# Regioselective Electrophilic Addition to Propargylic B(MIDA)s Enabled by *β*‐Boron Effect

**DOI:** 10.1002/advs.202304282

**Published:** 2023-08-26

**Authors:** Yin Li, Zhi‐Hao Chen, Shuang Lin, Yuan Liu, Jiasheng Qian, Qingjiang Li, Zhi‐Shu Huang, Honggen Wang

**Affiliations:** ^1^ Guangdong Key Laboratory of Chiral Molecule and Drug Discovery School of Pharmaceutical Sciences Sun Yat‐Sen University Guangzhou 510006 China

**Keywords:** electrophilic addition, hydration, internal alkyne, *β*‐boron effect, *β*‐difluoroalkylboron

## Abstract

Electrophilic addition reaction to alkynes is of fundamental importance in organic chemistry, yet the regiocontrol when reacting with unsymmetrical 1,2‐dialkyl substituted alkynes is often problematic. Herein, it is demonstrated that the rarely recognized *β*‐boron effect can confer a high level of site‐selectivity in several alkyne electrophilic addition reactions. A broad range of highly functionalized and complex organoborons are thus formed under simple reaction conditions starting from propargylic MIDA (*N*‐methyliminodiacetic acid) boronates. These products are demonstrated to be valuable building blocks in organic synthesis. In addition to the regiocontrol, this study also observes a drastic rate enhancement upon B(MIDA) substitution. Theoretical calculation reveals that the highest occupied molecular obital (HOMO) energy level of propargylic B(MIDA) is significantly raised by 0.3 eV, and the preferential electrophilic addition to the *γ* position is due to its higher HOMO orbital coefficient and more negative natural bond orbital (NBO) charge compared to the *β* position. This study demonstrates the potential of utilizing the *β*‐boron effect in stereoelectronic control of chemical transformations, which can inspire further research in this area.

## Graphical abstract

Electrophilic addition to alkynes is of fundamental importance, yet the regiocontrol is often problematic. Herein, the authors report that the rarely recognized *β*‐boron effect can confer a high level of site‐selectivity. The preferential electrophilic addition to the *γ* position is due to its higher highest occupied molecular orbital (HOMO) coefficient and more negative charge compared to the *β* position.

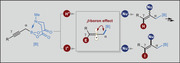

## 1 Introduction

**Scheme 1:**
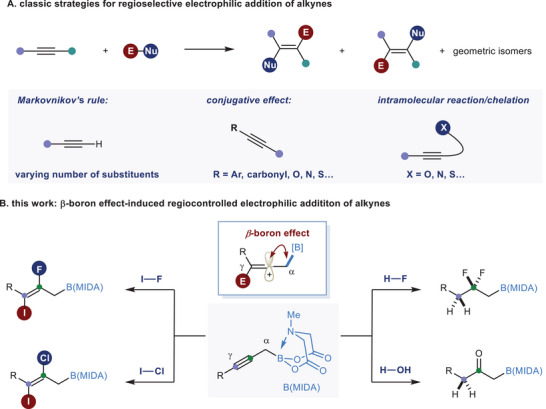
Regioselective electrophilic addition reaction to internal alkynes.

Electrophilic addition reaction to alkynes is of fundamental importance in organic chemistry.^[^ ^1^ ^]^ Alkynes are readily available and their triple bond allows for easy modifications, enabling the facile creation of diverse compounds from simple feedstocks.^[^ ^2^ ^]^ However, unsymmetrical alkynes pose a challenge in electrophilic addition reactions, as the addition may occur in two different directions, reducing the usefulness of the reaction (**Scheme** **1**A).^[^ ^1^, ^2^, ^3^ ^]^ To overcome this issue, a common strategy is to use terminal alkynes as substrates, following Markovnikov's rule.^[^ ^4^ ^]^ The underlying reason for this selectivity is the preferential formation of more stable carbocation intermediates that are involved in the mechanism, given that this step is often rate‐determining.^[^ ^5^ ^]^ In line with the same principle, the direct attachment of aryl,^[^ ^4^, ^6^ ^]^ carbonyl^[^ ^6^, ^7^ ^]^ or heteroatom^[^ ^8^ ^]^ to the triple bond could also perturb the electron distribution across the triple bond via conjugative effect. This can selectively deliver the product resulting from the conjugated carbocation intermediate. Introducing a directing group or a pendent nucleophile to the substrate is another common strategy used to achieve selectivity.^[^ ^9^ ^]^ The regioisomeric product derived from the minimal‐entropy‐penalty transition state typically predominates.^[^ ^10^ ^]^ However, achieving regiocontrol remains a challenging task for unsymmetrical dialkylalkynes that lack a strong electronic or steric bias.

Previous joint work from Yudin and us has demonstrated that the sp^3^‐B hybridized B(MIDA) (*N*‐methyliminodiacetic acid) moiety could exert an intriguing *β*‐boron effect, allowing for regioselective and stereospecific electrophilic addition to internal alkenes.^[^ ^11^ ^]^ This effect is analogous to the well‐known *β*‐silicon effect,^[^ ^12^ ^]^ but it shows distinct advantage in that the boryl moiety could be retained in the product.^[^ ^13^ ^]^ We thus hypothesized that the same effect might also be applicable to the electrophilic addition reaction of alkynes. However, due to the sluggish nature of the reactions of alkynes with electrophilic reagents compared to the corresponding reactions of alkenes, more severe conditions are often required^[^ ^1^, ^2^, ^3^ ^]^ These harsher conditions may lead to the decomposition of the boryl moiety and compromise the regioselectivity. In this paper, we present how the *β*‐boron effect can significantly enhance the reactivity of internal alkynes toward various types of electrophilic addition reactions. Furthermore, we demonstrate that a high level of regiocontrol can be achieved to produce synthetically challenging but useful organoboron products from simple propargylic B(MIDA)s (Scheme 1B).

## 2 Results and Discussion

### 2.1 Regioselective Dihydrofluorination

*β*‐Difluoroalkylborons, which contain the medicinally important CF_2_ moiety^[^ ^14^ ^]^ and a synthetically useful boron group,^[^ ^14^, ^15^ ^]^ are intriguing yet challenging synthons. Previous efforts toward their synthesis include iodoarene‐mediated difluorination reactions featuring 1,2‐aryl migration,^[^ ^16^ ^]^ 1,2‐B(MIDA) migration^[^ ^17^ ^]^ or 1,2‐hydrogen shift^[^ ^13d^ ^]^ under oxidative conditions, as well as Matteson‐type fluoroalkylation of (bromomethyl)pinacolborane, which has with limited scope. Direct dihydrofluorination of alkynes^[^ ^4^, ^18^ ^]^ has been recognized as one of the most atom‐economical and straightforward methods for gem‐difluorides synthesis. However, to avoid the potential regioselectivity problems, productive substrates typically include terminal alkynes, arylalkynes, and symmetric internal alkynes.

**Scheme 2:**
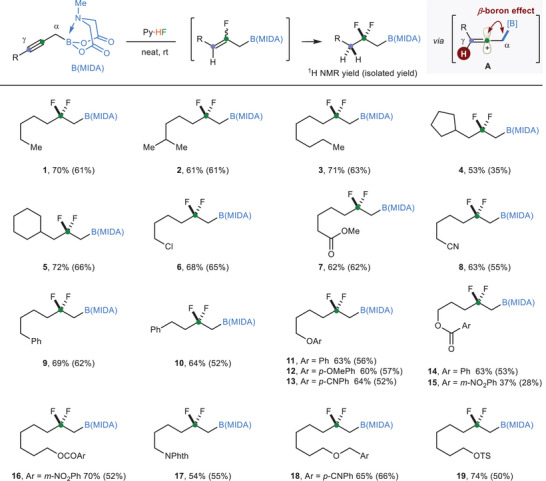
*β*‐Boron effect‐enabled regioselective dihydrofluorination of propargylic B(MIDA)s. Conditions: propargylic B(MIDA) (0.2 mmol), Py·HF (60.0 equiv), room temperature. Yields in parentheses were isolated yields. MIDA = *N*‐methyliminodiacetic acid; Py·HF = pyridine‐HF.

We first investigated the hydrofluorination reaction of propargylic B(MIDA)s and identified that in the presence of commercially available pyridine‐HF (Olah's reagent),^[^ ^19^ ^]^ hept‐2‐yn‐1‐yl B(MIDA) **S1** (See Supporting information) could be converted to *β*‐difluoroalkylboronate **1** in a regioselective manner, with a 61% isolated yield (70% ^1^H NMR yield) (**Scheme** **2**). No solvent or additive is required for transformation. The selectivity is believed to be due to the formation of vinyl carbocation A, which is stabilized by the adjacent boryl moiety. A wide variety of propargylic B(MIDA)s were then tested. In addition to linear propargylic B(MIDA)s (**1** and **3**), those with branched (**2**) or cyclic subsituents (**4** and **5**) were also competent substrates. Some commonly encountered functional groups such as chloro (**6**), ester (**7**, **14**, **15**, **16**), cyano (**8**), phenyl (**9** and **10**), ether (**11**–**13**), amide (**17**), and benzyl (**18**) were all well tolerated. The survival of OTs group in **19** provides opportunities for follow‐up transformation. The *tert*‐butyl‐substituted propargylic B(MIDA) also delivered the desired product, but we failed to isolate it due to some unknown impurities. The use of Bpin and BF_3_K congeners of **S1** predominately led to the decomposition of the starting materials.

### 2.2 Regioselective Hydration Reaction

*α*‐Boryl carbonyl compounds were previously thought to be thermodynamically unstable due to their tendency to undergo 1,3‐boron migration reactions.^[^ ^20^ ^]^ However, recent developments in sp^3^‐hybridized or tetra‐coordinated organoboron chemistry have led to the exploration of methods for their synthesis.^[^ ^13^, ^15^, ^21^ ^]^ Representative methods include the insertion reaction of borane with carbene precursors,^[^ ^21^, ^22^ ^]^ the free‐radical borylation reaction of alkenes,^[^ ^23^ ^]^ and the late‐stage structural modification reaction of several boron‐containing compounds.^[^ ^13^, ^24^ ^]^

**Scheme 3:**
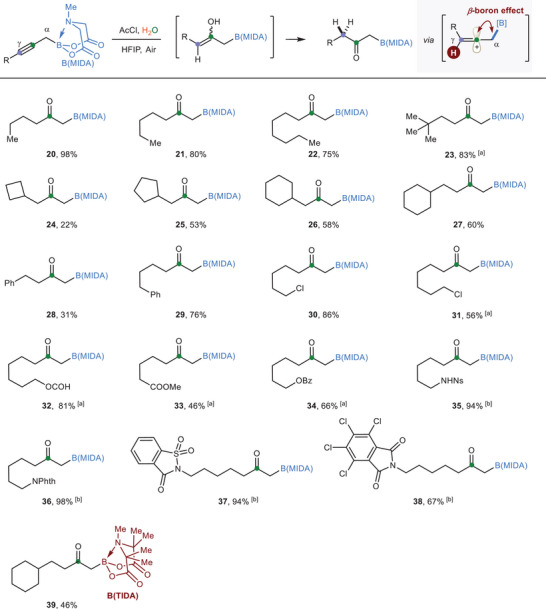
*β*‐Boron effect‐enabled regioselective hydration reaction of propargylic B(MIDA)s. Conditions: propargylic B(MIDA) (0.2 mmol), AcCl (15.0 equiv), H_2_O (10.0 equiv), HFIP (0.1 m), 30 °C. a) AcCl (5.0 equiv), H_2_O (2.5 equiv), 30 °C. b) AcCl (10.0 equiv), H_2_O (5.0 equiv), 60 °C; AcCl = acetyl chloride; HFIP = hexafluoroisopropanol.

The hydration reaction of alkynes is a textbook‐taught method for ketone synthesis. We recently uncovered a mild protocol for alkyne hydration reaction that eliminates the use of toxic mercury salts, precious metal catalysts, and strong Brønsted acids.^[^ ^25^ ^]^ The above success highlighted the potential of *β*‐boron effect in a regioselective alkyne hydration reaction. Indeed, after several trials, we found that in the presence of acetyl chloride (15.0 equiv) and H_2_O (10.0 equiv) in hexafluoroisopropanol (HFIP) at 30 °C, propargylic B(MIDA)s could be regioselective hydrated to the corresponding *α*‐boryl ketones (**Scheme** **3**). Notably, the B(MIDA) group remained unchanged in the reaction product. Similar to the dihydrofluorination reaction, a broad range of functional groups could be successfully tolerated. For substrates bearing labile ester functionality (**32**–**34**), reducing the loading of acetyl chloride to 5.0 equivalents was important for high yields. Moreover, a slight increase in reaction temperature to 60 °C was beneficial for substrates containing sulfonamide or amide groups (**35‐** **38**). The use of TIDA (tetramethyl *N*‐methyliminodiacetic acid) boronate, recently disclosed by Blair and Burke,^[^ ^15j^ ^]^ also furnished the corresponding *α*‐boryl ketone product in moderate yield (**39**), suggesting potential utility of these products as automated iterative building blocks.

### 2.3 Regioselective Iodoflurionation and Iodochlorination

**Scheme 4:**
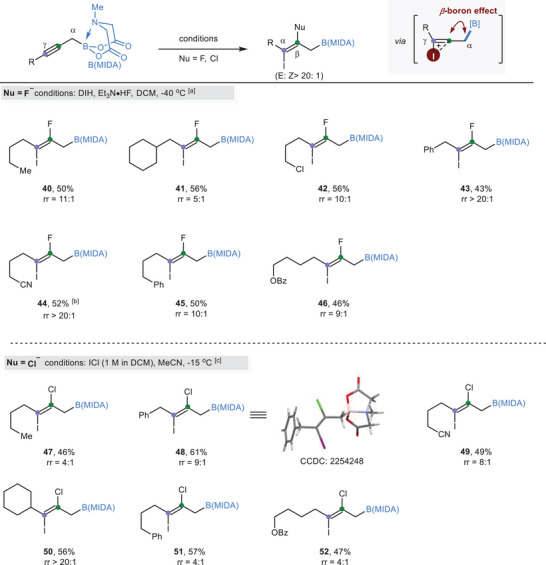
B(MIDA)‐guided regioselective iodoflurionation and iodochlorination of propargylic B(MIDA)s. a) Conditions: propargylic B(MIDA) (0.2 mmol), DIH (1.2 equiv), Et_3_N·HF (9.0 equiv), DCM (1.0 mL), −40 °C. b) DIH (1.7 equiv), Et_3_N·HF (14.0 equiv), DCM (1.0 mL), −40 °C. c) Conditions: propargylic B(MIDA) (0.2 mmol), ICl (2.0 equiv), MeCN (1.0 mL), −15 °C; DIH = 1,3‐diiodo‐5,5'‐dimethyl hydantoin; DCM = dichloromethane. Et_3_N·HF = triethylamine‐3HF.

To further test the robustness of the *β*‐boron effect and to introduce more structural complexity to the organoboron products, we turned our attention to the iodohalogenation reactions (**Scheme** **4**).^[^ ^4b^ ^]^ In the presence of electrophilic 1,3‐diiodo‐5,5'‐dimethyl hydantoin (DIH) and nucleophilic triethylamine‐3HF (Et_3_N·HF) in dichloromethane(DCM) at −40 °C, hept‐2‐yn‐1‐yl B(MIDA) **S1** was successfully converted to the iodofluorinated product **40** with good regioselectivity (rr = 11:1). As expected, the electrophilic iodine atom was preferentially attached to the more nucleophilic *γ* position. The reaction was trans‐selective, with only the *E* isomer observed. We then briefly examined the scope of the reaction and found that functional groups including chloro (**42**), phenyl (**43**, **45**), cyano (**44**), and ester (**46**) were well tolerated. Of note, the regioselectivity was sensitive to the electronic properties of the substituents. While electron‐rich substituent (Cy **41**) compromised the level of regioselectivity, electron‐withdrawing functional groups (Ph **43**, CN **44**) improved the regioselectivity. This phenomenon could be rationalized by the redistribution of electron‐density on the triple bond induced by the inductive effect.

We also attempted the iodochlorination reaction with I–Cl. This reaction is more challenging as I–Cl is highly electrophilic compared to DIH. Still, we achieved moderate levels of regioselectivities. Similarly, the introduction of electron‐withdrawing functional groups (Ph **48**, CN **49**) was beneficial for regioselectivity. Interestingly, a high regio‐ratio (20:1) was observed in secondary alkyl‐substituted alkyne (**50**). The *tert*‐butyl‐substituted propargylic B(MIDA) failed to give any desired products. Presumably, the compromised regioselectivity for iodohalogenation reactions is due to the too high reactivity of the initially formed cyclic iodonium ion toward nucleophilic ring‐opening.

### 2.4 Mechanistic Studies

**Scheme 5:**
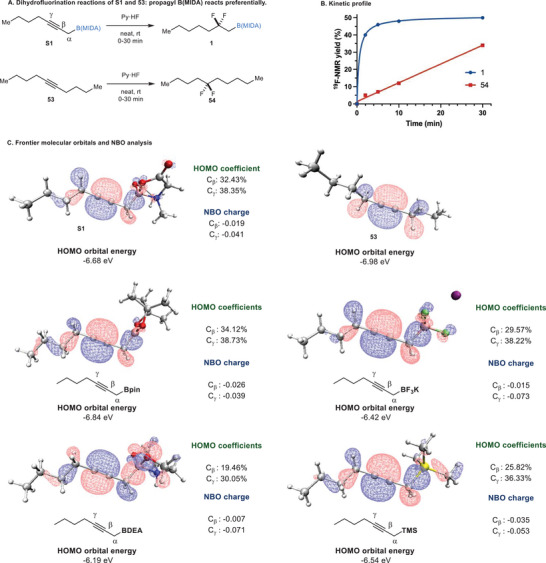
Mechanistic studies. HOMO = Highest Occupied Molecular Orbital; NBO = Natural Bond Orbital.

After demonstrating the effectiveness of *β*‐boron effect in regiocontrol, we were interested in understanding the magnitude of reactivity enhancement exerted by the B(MIDA)‐moiety. To investigate this, we measured and plotted the kinetics of the dihydrofluorination reactions of propargyl B(MIDA) **S1** and an internal alkyne **53 (** **Scheme** **5**A). We found that within 2 min, the yield of **1** had risen to 40%, compared to only 5% for **54**, indicating the reaction of **S1** is at least eight times faster (Scheme 5B).

Theoretical calculation showed the Highest Occupied Molecular Obital (HOMO) energy level of **S1** was indeed raised significantly by 0.3 eV upon B(MIDA) substitution. The preferential electrophilic addition at the *γ* position is consistent with the fact that this position has the highest HOMO orbital coefficient and more negative Nature Bond Orbital (NBO) charge compared to the *β* position (Scheme 5C).^[^ ^26^ ^]^ The calculations about other propargylic organoboron compounds and the propargylic silane were also conducted. It was found all these species have elevated HOMO energy. However, the energy level for sp^3^‐B species is generally higher than the sp^2^‐hybridized Bpin.

### 2.5 Synthetic Applications

**Scheme 6:**
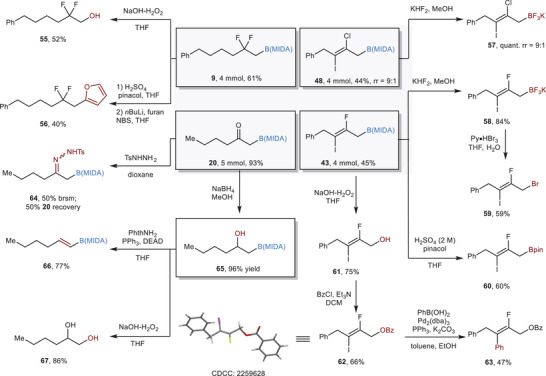
Synthetic transformations.

Finally, to demonstrate the robustness and utilities of our protocols, several transformations were conducted (**Scheme** **6**). All four protocols were amenable for gram‐scale synthesis with no obvious loss of efficacy. The gem‐difluorinated alkyl B(MIDA) **9** can be oxidized to the corresponding alcohol **55**, which represents another type of useful building block. Ligand exchange followed by an oxidative ligation reaction with furan‐2‐yl‐lithium furnished **56** in 40% yield over two steps.^[^ ^27^ ^]^ A condensation reaction of carbonyl group in **20** with sulfonhydrazide formed hydrazone **64** in 50% yield based on the recovery of the starting material. Reduction of the carbonyl group with NaBH_4_ in methanol was successful with no deborylation reaction being observed. The resulting alcohol **65** could be subjected to a dehydration reaction under Mitsunobu reaction conditions and oxidative reaction to deliver the alkenyl boronate **66** and diol product **67**, respectively. The iodination products (**48, 43)** could be easily transformed to their BF_3_K congeners (**57** and **58**) in high yields. Ligand exchange to form the pinacol boronic ester was also possible (**60**). The BF_3_K salt **58** could be converted to bromide **59** without difficulty. A fully substituted allyl alcohol was accessible upon the oxidation with H_2_O_2_. By protecting the hydroxyl group with BzCl, the resulting ester **62** ^[^ ^28^ ^]^ could undergo Suzuki–Miyaura coupling reaction to provide **63** in a stereoretentive manner.

## 3 Conclusion

In conclusion, controlling the regioselectivity of electrophilic addition to internal alkynes can be difficult. We showed herein that by taking advantage of the rarely recognized *β*‐boron effect, a high level of site‐selectivity for several alkynes electrophilic addition reactions was achieved. These reactions provide a straightforward method for synthesizing a broad range of highly functionalized and complex organoborons, which are otherwise difficult targets using alternative methods. We hope that our success in utilizing this chemistry will inspire further exploration of stereoelectronic control in chemical transformations using the *β*‐boron effect.

## Conflict of Interest

The authors declare no conflict of interest.

## Supporting information

Supporting Information

## Data Availability

The data that support the findings of this study are available in the supplementary material of this article.
