# Supplementary material for: Regioselective Electrophilic Addition to Propargylic B(MIDA)s Enabled by β‐Boron Effect
Source: Adv Sci (Weinh). 2023 Aug 26;10(30):2304282. doi: 10.1002/advs.202304282 (PMC10602563; doi:10.1002/advs.202304282)
Supplement: Supplementary file 1 — Supporting Information [file ADVS-10-2304282-s001.pdf]

ADVANCED SCIENCE
Open Access

Supporting Information

for *Adv. Sci.*, DOI 10.1002/advs.202304282

Regioselective Electrophilic Addition to Propargylic B(MIDA)s Enabled by β-Boron Effect

*Yin Li, Zhi-Hao Chen, Shuang Lin, Yuan Liu, Jiasheng Qian, Qingjiang Li, Zhi-Shu Huang and Honggen Wang**

Supplementary Information

Regioselective Electrophilic Addition to Propargylic B(MIDA)s Enabled by *β*-Boron Effect

Yin Li,[a,#] Zhi-Hao Chen,[a,#] Shuang Lin,[a] Yuan Liu,[a] Jiasheng Qian,[a] Qingjiang Li,[a] Zhi-Shu Huang[a] and Honggen Wang[a,*]

[a]Guangdong Key Laboratory of Chiral Molecule and Drug Discovery, School of Pharmaceutical Sciences, Sun Yat-Sen University, Guangzhou, 510006, China. E-mail: wanghg3@mail.sysu.edu.cn (H.W.)
[#]Y.L., and Z.C. contributed equally to this work.

Table of contents

1. General information 3
2. Preparation of starting materials 4
3. Characterization of the starting materials 6
4.General reaction development and optimization 10
5. General procedure for the synthesis of product 12
    5.1 General procedure A for the synthesis of β-difluorinated boronates 12
    5.2 General procedure B for the synthesis of α-boryl ketones 12
    5.3 General procedure C for the synthesis of β-fluoro-γ-iodinated boronates 12
    5.4 General procedure D for the synthesis of β-chloro-γ-iodinated boronates 13
6. Derivatization of the products 13
7. Limitation of substrates 18
8. Characterization of the products 18
9. Mechanistic experiments 36
    9.1 Competition experiment: propargyl B(MIDA) reacts preferentially 36
    9.2 Computational Studies 37
10. X-ray crystal structure data 46
11. References 49
12. NMR spectra of the starting materials and products 51

1. General information

Unless otherwise noted, all commercially available materials were used without further purification.

DIH (1,3-diiodo-5,5-dimethylhydantoin) (CAS 2232-12-4), was purchased from TCI and stored at -20 ^{o}C;

Py·HF (CAS 32001-55-1, 65%- 70% w/w) was purchased from Sigma-Aldrich and stored at 4 ^{o}C;

Et_3N·HF (CAS 73602-61-6) was purchased from Sigma-Aldrich and stored at 4 ^{o}C

NMR–spectra were recorded on Bruker AvanceIII–400M and Ascend^{TM} 500M in solvents as indicate. Chemical shifts (*δ*) are given in ppm relative to tetramethylsilane (*δ* = 0). The residual solvent signals were used as references. The following abbreviations were used to describe peak splitting patterns: s (singlet), d (doublet), t (triplet), q (quartet), septet (sept), m (multiplet), dd (doublet of doublets), dt (doublet of triplets), triplet of doublets (td). Coupling constants (*J*) were reported in hertz unit (Hz). High–resolution mass spectra (HRMS) were recorded on a Bruker VPEXII spectrometer with ESI mode unless otherwise stated.

Analytical thin layer chromatography was performed on Polygram SIL G/UV254 plates. Visualization was accomplished by UV light (254 nm), or $KMnO_4$ staining solutions followed by heating, also by Gas chromatograph-Mass spectrometer analysis (GC-MS) on Agilent Technologies 5977A MSD. Flash column chromatography was performed using silica gel (200–300 mesh).

No attempts were made to optimize yields for substrate synthesis or products derivatizations.

2. Preparation of starting materials

The starting materials were prepared according to literature procedures.[1] The spectroscopic data of known compounds were consistent with the reported values.

2.1 Synthesis of starting material S17

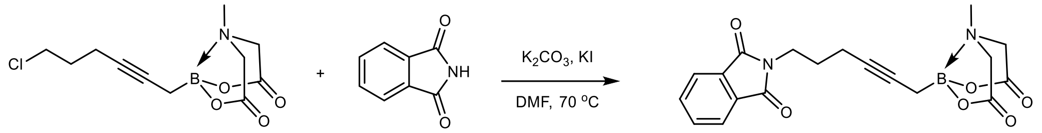

Under an argon atmosphere, a Schlenk tube equipped with a magnetic stir bar was charged with phthalimide (1.2 mmol, 1.2 equiv., 176 mg), K_2CO_3 (1.0 mmol, 1.0 equiv.) and KI (4 mg). Then **S6** (1.0 mmol, 1.0 equiv. 271 mg) in DMF (2 mL) was added to the reaction mixture. The resulting mixture was stirred at 70 °C for 24 h. After cooling to room temperature, the reaction mixture was diluted with ethyl acetate (50 mL) and water (25 mL). The organic phase was separated and the aqueous layer was extracted with ethyl acetate and acetone (20 mL + 20 mL) for three times. The combined organic layer was dried over anhydrous Na_2SO_4 and concentrated under reduced pressure. The resulting crude product was purified by flash chromatography on silica gel to afford the pure product **S-17** (48% yield). The spectroscopic data of known compounds were consistent with the reported values.[1]

2.2 Synthesis of starting material S15, S16, S19[2]

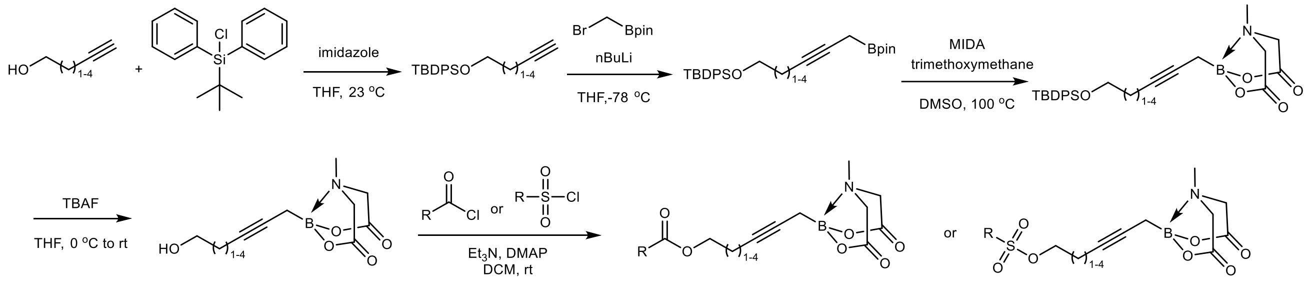

To a solution of alcohol (1.5 mL, 20 mmol, 1.0 equiv) and imidazole (3.404 g, 50 mmol, 2.5 equiv) in THF (40 mL), was added TBDPSCl (6.24 mL, 24 mmol, 1.2 equiv) dropwise at room temperature. The reaction mixture was stirred at for 4 h. After that, the reaction mixture was quenched with saturated NH_4Cl solution and was extracted with Et_2O. The combined organic layer was dried over anhydrous Na_2SO_4 and concentrated under reduced pressure. The resulting crude product was purified by flash chromatography on silica gel to afford the pure product.

To a stirred solution of THF (17 mL) containing terminal alkynes (17 mmol), *n*-butyl lithium in hexane (2.5 M, 6.12 mL, 15.3 mmol) was added dropwise at −78 °C. The resulting solution was stirred at the same temperature for 1 h, and then (bromomethyl)pinacolborane ($BrCH_2Bpin$, 3.76 g, 17 mmol) was added dropwise and the mixture was stirred at room temperature for 2 h. After that, Saturated NH_4Cl(aq.) was added to the reaction mixture at 0 °C and the solution was stirred for 20 min. The reaction mixture was mixed with water and the product was extracted repeatedly with dichloromethane (DCM). The organic phase was separated and the aqueous layer was extracted with DCM (30 mL) for three times. The combined organic layer was dried over anhydrous Na_2SO_4 and concentrated under reduced pressure. Anhydrous DMSO (40 mL) was added to

dissolve the solid (or viscous oil) which then *N*-methyliminodiacetic acid (MIDA, 6.0 equiv.) and $CH(OMe)_3$ (4.0 equiv.) was added. The resulting mixture was stirred at 100 °C until the propargyl boronic esters was used up by GC-MS monitoring. After cooling to room temperature, the reaction mixture was diluted with ethyl acetate (50 mL) and water (50 mL). The organic phase was separated and the aqueous layer was extracted with ethyl acetate (50 mL) for three times. The combined organic layer was dried over anhydrous Na_2SO_4 and concentrated under reduced pressure. The resulting crude product was purified by flash chromatography on silica gel with an appropriate solvent as eluent to afford the pure product.

To a stirred solution of THF (7.5 ml) containing pure product (2.0 mmol) of previous step, TBAF (1M in THF, 2 mL) was added at 0 °C. The reaction mixture was stirred at 0 °C for 30 min. Then the reaction mixture was allowed to warm to room temperature and stirred at that temperature for 4 h. The reaction mixture was diluted with ethyl acetate (20 mL) and water (20 mL). The organic phase was separated and the aqueous layer was extracted with ethyl acetate (20 mL) for three times. The combined organic layer was washed with brine for three times. The organic layer was dried over anhydrous Na_2SO_4 and concentrated under reduced pressure. The resulting crude product was purified by flash chromatography on silica gel (with DCM to PE:EA = 1:2 to PE:EA = 1:3 as eluent) to afford the pure product.

Under an argon atmosphere, a Schlenk tube equipped with a magnetic stir bar was charged with hydroxyl MIDA boronate (1.0 equiv.) and DCM (0.1 M). The reaction mixture was cooled to 0 °C. Et_3N (2.0 equiv.) was added to the reaction mixture. The resulting mixture was stirred at 0 °C for 30 min. acyl chloride (1.2 equiv.)/ sulfonyl chloride (1.2 equiv.), DMAP (0.1 equiv.) and DCM were added to the reaction mixture. Then the reaction mixture was allowed to warm to room temperature and stirred overnight. Until the complete consumption of hydroxyl MIDA boronate as monitored by TLC analysis. The reaction mixture was diluted with ethyl acetate and water. The organic phase was separated and the aqueous layer was extracted with ethyl acetate and acetone for three times. The combined organic layer was dried over anhydrous Na_2SO_4 and concentrated under reduced pressure. The resulting crude product was purified by flash chromatography on silica gel to afford the pure product.

2.3 Synthesis of starting material S35, S36, S37, S38

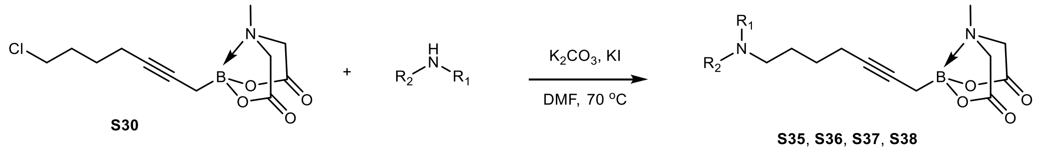

Under an argon atmosphere, a Schlenk tube equipped with a magnetic stir bar was charged with secondary amines (1.2 equiv.), K_2CO_3 (1.0 equiv.) and KI (3 mol%). Then **S30** (1.0 equiv.) in DMF (0.5 M) was added to the reaction mixture. The resulting mixture was stirred at 70 °C for 24 h. After cooling to room temperature, the reaction mixture was diluted with ethyl acetate and water. The organic phase was separated and the aqueous layer was extracted with ethyl acetate and acetone for three times. The combined organic layer was dried over anhydrous Na_2SO_4 and concentrated under reduced pressure. The resulting crude product was purified by flash chromatography on silica gel to afford the pure product.

3. Characterization of the starting materials

5-(6-methyl-4,8-dioxo-1,3,6,2-dioxazaborocan-2-yl)pent-3-yn-1-yl 3-nitrobenzoate (S15)

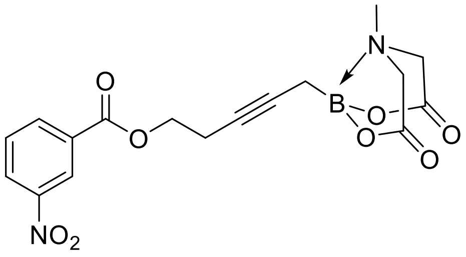

S15 was prepared according to literature procedure, and was obtained in 63% yield as a white solid after column chromatography (eluent = petroleum ether/ ethyl acetate 1:3 v/v). R_F = 0.40 (PE: ethyl acetate = 1:4). ^{1}H NMR (500 MHz, Acetone-d_6) δ 8.77 (t, *J* = 2.0 Hz, 1H), 8.51 (dd, *J* = 8.6, 2.4 Hz, 1H), 8.45 (dt, *J* = 7.8, 1.4 Hz, 1H), 7.87 (t, *J* = 8.0 Hz, 1H), 4.43 (t, *J* = 6.6 Hz, 2H), 4.23 (d, *J* = 16.8 Hz, 2H), 4.07 (d, *J* = 16.8 Hz, 2H), 3.25 (s, 3H), 2.68 (dq, *J* = 6.6, 3.8, 3.2 Hz, 2H), 1.68 – 1.61 (m, 2H). ^{11}B NMR (128 MHz, Acetone-d_6) δ 11.64. ^{13}C NMR (126 MHz, Acetone-d_6) δ 167.6, 164.1, 148.4, 135.3, 131.9, 130.4, 127.5, 123.9, 80.1, 75.3, 64.2, 62.4, 45.5, 18.9. **ESI-MS:** calcd for $C_{17}H_{17}N_2O_8BNa$ [M + Na]$^+$: 411.0971, found: 411.0974.

5-(6-methyl-4,8-dioxo-1,3,6,2-dioxazaborocan-2-yl)pent-3-yn-1-yl 3-nitrobenzoate (S16)

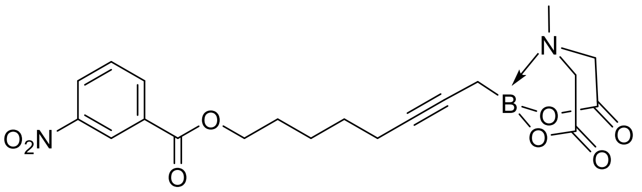

S16 was prepared according to literature procedure, and was obtained in 70% yield as a white solid after column chromatography (eluent = petroleum ether/ ethyl acetate 1:3 v/v). R_F = 0.39 (PE: ethyl acetate = 1:4). ^{1}H NMR (500 MHz, Acetonitrile-d_3) δ 8.71 (t, *J* = 2.0 Hz, 1H), 8.42 (ddd, *J* = 8.2, 2.5, 1.1 Hz, 1H), 8.36 (dt, *J* = 7.7, 1.4 Hz, 1H), 7.74 (t, *J* = 8.0 Hz, 1H), 4.34 (t, *J* = 6.6 Hz, 2H), 3.99 (d, *J* = 16.9 Hz, 2H), 3.86 (d, *J* = 17.0 Hz, 2H), 3.03 (s, 3H), 2.15 – 2.11 (m, 2H), 1.82 – 1.73 (m, 2H), 1.58 (s, 2H), 1.53 – 1.46 (m, 4H). ^{11}B NMR (160 MHz, Acetone-d_6) δ 11.59. ^{13}C NMR (126 MHz, Acetonitrile-d_3) δ 168.5, 165.0, 149.0, 135.7, 132.8, 130.8, 128.0, 124.5, 80.2, 78.6, 66.2, 63.0, 46.2, 29.0, 28.4, 25.6, 18.7. **ESI-MS:** calcd for $C_{20}H_{23}N_2O_8BNa$ [M + Na]$^+$: 453.1440, found: 453.1436.

5-(6-methyl-4,8-dioxo-1,3,6,2-dioxazaborocan-2-yl)pent-3-yn-1-yl 3-nitrobenzoate (S18)[3]

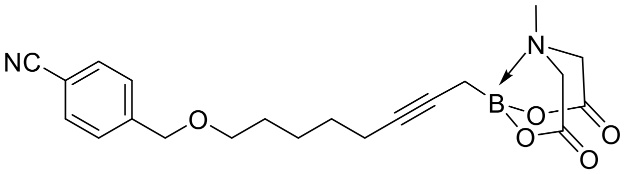

S18 was prepared according to literature procedure, and was obtained in 20% yield (two steps) as a white solid after column chromatography (eluent = petroleum ether/ ethyl acetate 1:3 v/v). R_F = 0.25 (PE: ethyl acetate = 1:4). ^{1}H NMR (500 MHz, Acetone-d_6) δ 7.75 (d, *J* = 8.3 Hz, 2H), 7.57 (d, *J* = 8.1 Hz, 2H), 4.60 (s, 2H), 4.25 (d, *J* = 16.8 Hz, 2H), 4.05 (d, *J* = 16.8 Hz, 2H), 3.53 (t, *J* = 6.4 Hz, 2H), 3.26 (s, 3H), 2.15 – 2.10 (m, 2H), 1.67 – 1.57 (m, 4H), 1.47 (hept, *J* = 2.2 Hz, 4H). ^{11}B NMR (128 MHz, Acetone-d_6) δ 11.55. ^{13}C NMR (126 MHz, Acetone-d_6) δ 167.6, 145.1, 132.0, 127.8, 118.5, 110.8, 79.3, 78.2, 71.3, 70.4, 62.5, 45.4, 25.4, 18.3. **ESI-MS:** calcd for $C_{21}H_{25}N_2O_5BNa$ [M + Na]$^+$: 419.1750, found: 419.1750.

5-(6-methyl-4,8-dioxo-1,3,6,2-dioxazaborocan-2-yl)pent-3-yn-1-yl 3-nitrobenzoate (S19)

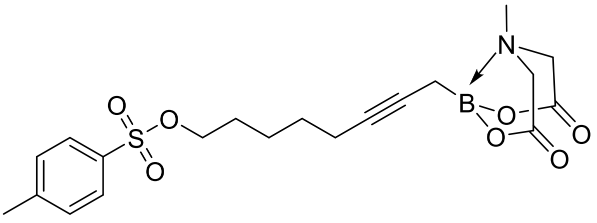

S19 was prepared according to literature procedure, and was obtained in 51% yield (two steps) as a white solid after column chromatography (eluent = petroleum ether/ ethyl acetate 1:3 v/v). R_F = 0.44 (PE: ethyl acetate = 1:4). ^{1}H NMR (500 MHz, Acetonitrile-d_3) δ 7.78 (d, *J* = 8.4 Hz, 2H), 7.44 (d, *J* = 7.8 Hz, 2H), 4.03 – 3.95 (m, 4H), 3.85 (d, *J* = 16.9 Hz, 2H), 3.02 (s, 3H), 2.45 (s, 3H), 2.07 – 2.02 (m, 2H), 1.62 – 1.54 (m, 4H), 1.40 – 1.24 (m, 4H). ^{11}B NMR (128 MHz, Acetone-d_6) δ 11.54. ^{13}C NMR (126 MHz, Acetonitrile-d_3) δ 168.5, 145.9, 133.6, 130.6, 128.3, 80.1, 78.6, 71.6, 63.0, 46.2, 28.7, 28.6, 25.0, 21.2, 18.6.

2-(7-chlorohept-2-yn-1-yl)-6-methyl-1,3,6,2-dioxazaborocane-4,8-dione (S30)

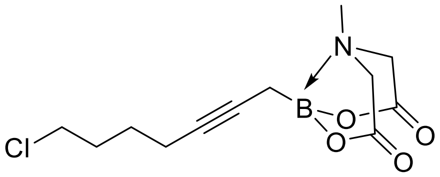

S30 was prepared according to literature procedure,[1] and was obtained in 35% yield (two steps) as a white solid after column chromatography (eluent = petroleum ether/ ethyl acetate 1:3 v/v). R_F = 0.60 (ethyl acetate). ^{1}H NMR (400 MHz, Acetonitrile-d_3) δ 4.00 (d, *J* = 16.9 Hz, 2H), 3.86 (d, *J* = 16.9 Hz, 2H), 3.59 (t, *J* = 6.6 Hz, 2H), 3.03 (s, 3H), 2.14 (tt, *J* = 7.1, 4.5, 2.6 Hz, 2H), 1.84 – 1.74 (m, 2H), 1.59 (s, 2H), 1.58 – 1.50 (m, 2H). ^{11}B NMR (128 MHz, CD_3CN) δ 11.6. ^{13}C NMR (101 MHz, CD_3CN) δ 168.8, 80.1, 79.2, 63.3, 46.6, 45.7, 32.4, 27.0, 18.5. **ESI-MS:** calcd for $C_{12}H_{17}BClNO_4Na$ [M + Na]$^+$: 308.0831, found: 308.0831.

2-(8-chlorooct-2-yn-1-yl)-6-methyl-1,3,6,2-dioxazaborocane-4,8-dione (S31)

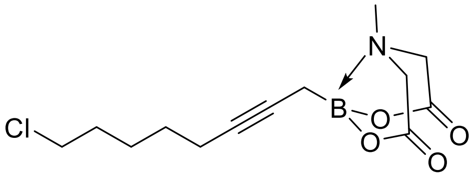

S31 was prepared according to literature procedure,[1] and was obtained in 30% yield (two steps) as a white solid after column chromatography (eluent = petroleum ether/ ethyl acetate 1:3 v/v). R_F = 0.62 (ethyl acetate). ^{1}H NMR (400 MHz, Acetonitrile-d_3) δ 4.00 (d, *J* = 16.9 Hz, 2H), 3.86 (d, *J* = 16.9 Hz, 2H), 3.58 (t, *J* = 6.7 Hz, 2H), 3.03 (s, 3H), 2.14 – 2.08 (m, 2H), 1.79 – 1.69 (m, 2H), 1.59 (t, *J* = 2.7 Hz, 2H), 1.47 – 1.42 (m, 2H). ^{11}B NMR (128 MHz, CD_3CN) δ 11.7. ^{13}C NMR (101 MHz, CD_3CN) δ 168.9, 80.6, 78.9, 63.4, 46.6, 46.1, 32.9, 29.1, 26.9, 19.1. **ESI-MS:** calcd for $C_{13}H_{19}BClNO_4Na$ [M + Na]$^+$: 322.0988, found: 322.0992.

8-(6-methyl-4,8-dioxo-1,3,6,2-dioxazaborocan-2-yl)oct-6-yn-1-yl formate (S32)

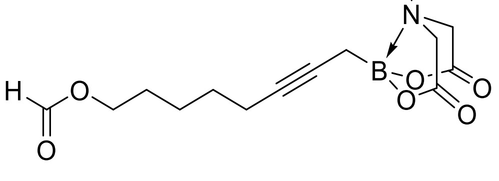

S-32 was prepared according to literature procedure,[1] and was obtained in 13% yield (two steps) as a white solid after column chromatography (eluent = petroleum ether/ ethyl acetate 0:1 v/v). R_F = 0.40 (ethyl acetate). ^{1}H NMR (500 MHz, Acetone-d_6) δ 8.12 (s, 1H), 4.26 (d, *J* = 16.8 Hz, 2H), 4.13 (t, *J* = 6.6 Hz, 2H), 4.05 (d, *J* = 16.8 Hz, 2H), 3.27 (s, 3H), 2.18 – 2.11 (m, 2H), 1.69 – 1.57 (m, 4H), 1.54 – 1.38 (m, 4H). ^{11}B NMR (128 MHz, Acetone-d_6) δ 11.6. ^{13}C NMR (126 MHz, Acetone-d_6) δ 168.6, 162.1, 80.1, 79.2, 64.2, 63.4, 46.4, 29.5, 29.0, 25.9, 19.2. **ESI-MS:** calcd for $C_{14}H_{20}BNO_6Na$ [M + Na]$^+$: 332.1276, found: 332.1279.

7-(6-methyl-4,8-dioxo-1,3,6,2-dioxazaborocan-2-yl)hept-5-yn-1-yl benzoate (S34)

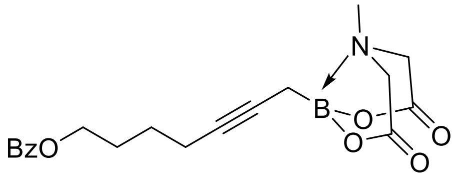

S34 was prepared according to literature procedure,[1] and was obtained in 60% yield (two steps) as a white solid after column chromatography (eluent = petroleum ether/ ethyl acetate 1:4 v/v). R_F = 0.52 (ethyl acetate). ^{1}H NMR (400 MHz, Acetone-*d*$_6$) δ 8.03 (d, *J* = 7.6 Hz, 2H), 7.63 (t, *J* = 7.2 Hz, 1H), 7.51 (t, *J* = 7.5 Hz, 2H), 4.32 (t, *J* = 6.4 Hz, 2H), 4.26 (d, *J* = 16.9 Hz, 2H), 4.06 (d, *J* = 16.9 Hz, 2H), 3.26 (s, 2H), 2.22 (t, *J* = 6.6 Hz, 3H), 1.90 – 1.79 (m, 2H), 1.63 (q, *J* = 12.7, 10.1 Hz, 5H). ^{11}B NMR (128 MHz, Acetone-*d*$_6$) δ 11.7. ^{13}C NMR (101 MHz, Acetone-*d*$_6$) δ 168.5, 166.7, 133.7, 131.3, 130.1, 129.3, 79.8, 79.4, 65.1, 63.3, 46.3, 28.6, 26.4, 18.9. **ESI-MS:** calcd for $C_{19}H_{22}BNO_6Na$ [M + Na]$^+$: 394.1432, found: 394.1434.

***N*-(7-(6-methyl-4,8-dioxo-1,3,6,2-dioxazaborocan-2-yl)hept-5-yn-1-yl)-4-nitrobenzenesulfonamide (S35)**

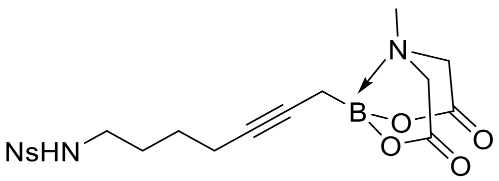

S35 was obtained in 40% yield as a white solid after column chromatography (eluent = petroleum ether/ ethyl acetate 1:4 v/v). R_F = 0.58 (ethyl acetate). ^{1}H NMR (400 MHz, Acetone-*d*$_6$) δ 8.44 (d, *J* = 8.7 Hz, 2H), 8.13 (d, *J* = 8.7 Hz, 2H), 4.27 (d, *J* = 16.9 Hz, 2H), 4.05 (d, *J* = 16.9 Hz, 2H), 3.25 (s, 3H), 3.04 – 2.94 (m, 2H), 2.90 (s, 3H), 2.12 – 2.08 (m, 2H), 1.65 – 1.50 (m, 4H), 1.49 – 1.39 (m, 2H). ^{13}C NMR (101 MHz, Acetone-*d*$_6$) δ 168.4, 150.4, 147.1, 128.8, 124.8, 79.4, 78.9, 63.0, 46.0, 43.1, 43.0, 29.1, 26.2, 18.3. ^{11}B NMR (128 MHz, Acetone-*d*$_6$) δ 11.8. **ESI-MS:** calcd for $C_{18}H_{22}BN_3O_8S$ Na [M + Na]$^+$: 474.1113, found: 474.1117.

2-(7-(1,3-dioxoisoindolin-2-yl)hept-2-yn-1-yl)-6-methyl-1,3,6,2-dioxazaborocane-4,8-dione (S36)

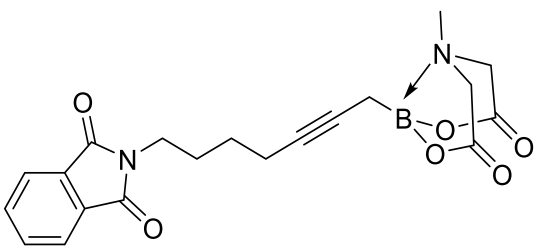

S36 was obtained in 34% yield as a white solid after column chromatography (eluent = petroleum ether/ ethyl acetate 1:4 v/v). R_F = 0.60 (ethyl acetate). ^{1}H NMR (400 MHz, Acetone-*d*$_6$) δ 7.71 (s, 4H), 4.13 (d, *J* = 16.8 Hz, 2H), 3.94 (d, *J* = 16.9 Hz, 2H), 3.52 (t, *J* = 7.1 Hz, 2H), 3.14 (s, 3H), 2.05 (t, *J* = 5.6 Hz, 2H), 1.61 (p, *J* = 7.2 Hz, 2H), 1.47 (s, 2H), 1.36 (p, *J* = 7.2 Hz, 2H). ^{13}C NMR (126 MHz, Acetone-*d*$_6$) δ 168.7, 168.4, 134.7, 132.9, 123.5, 79.6, 79.2, 63.2, 46.1, 37.7, 37.7, 28.2, 26.8, 18.5. ^{11}B NMR (128 MHz, Acetone-*d*$_6$) δ 11.7. **ESI-MS:** calcd for $C_{20}H_{21}BN_2O_6Na$ [M + Na]$^+$: 419.1385, found: 419.1388.

2-(7-(1,1-dioxido-3-oxobenzo[d]isothiazol-2(3H)-yl)hept-2-yn-1-yl)-6-methyl-1,3,6,2-dioxazaborocane-4,8-dione (S37)

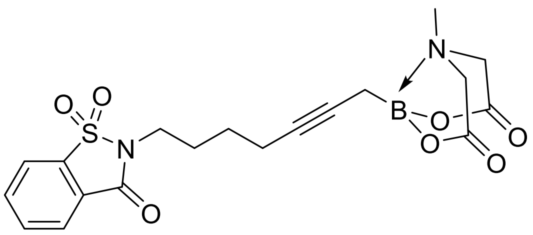

S37 was obtained in 32% yield as a white solid after column chromatography (eluent = petroleum ether/ ethyl acetate 1:4 v/v). R_F = 0.65 (ethyl acetate). ^{1}H NMR (400 MHz, Acetone-d_6) δ 8.27 – 7.87 (m, 4H), 4.26 (d, *J* = 16.8 Hz, 2H), 4.07 (d, *J* = 16.8 Hz, 2H), 3.77 (t, *J* = 7.2 Hz, 4H), 3.27 (s, 3H), 2.22 (tt, *J* = 7.0, 2.5 Hz, 2H), 1.96 – 1.85 (m, 2H), 1.67 – 1.53 (m, 4H). ^{13}C NMR (101 MHz, Acetone-d_6) δ 168.6, 159.7, 138.7, 136.3, 135.7, 128.1, 125.9, 121.9, 79.7, 79.6, 63.4, 46.4, 39.3, 28.5, 27.0, 18.8. ^{11}B NMR (128 MHz, Acetone-d_6) δ 11.7. **ESI-MS:** calcd for $C_{19}H_{21}BN_2O_7S$ Na [M + Na]$^+$: 455.1055, found: 455.1055.

6-methyl-2-(7-(4,5,6,7-tetrachloro-1,3-dioxoisoindolin-2-yl)hept-2-yn-1-yl)-1,3,6,2-dioxazaborocane-4,8-dione (S38)

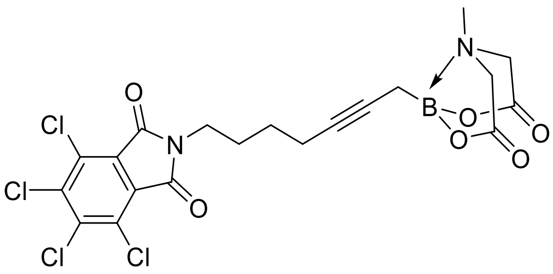

S38 was obtained in 20% yield as a yellow solid after column chromatography (eluent = petroleum ether/ ethyl acetate 1:4 v/v). R_F = 0.72 (ethyl acetate). ^{1}H NMR (400 MHz, DMSO-d_6) δ 4.27 (d, *J* = 17.1 Hz, 2H), 3.94 (d, *J* = 17.0 Hz, 2H), 3.56 (t, *J* = 6.9 Hz, 2H), 2.99 (s, 3H), 2.12 (t, *J* = 6.9 Hz, 2H), 1.68 – 1.56 (m, 2H), 1.52 (s, 2H), 1.44 – 1.33 (m, 2H). ^{13}C NMR (101 MHz, DMSO-d_6) δ 168.6, 163.5, 137.9, 128.5, 127.9, 78.8, 78.7, 62.2, 45.8, 37.8, 26.9, 25.8, 17.7. ^{11}B NMR (128 MHz, DMSO-d_6) δ 11.2. **ESI-MS:** calcd for $C_{20}H_{17}BCl_4N_2O_6$ Na [M + Na]$^+$: 554.9826, found: 554.9836.

4.General reaction development and optimization

Table S1. Reaction optimization for the synthesis of β-difluorinated boronates.

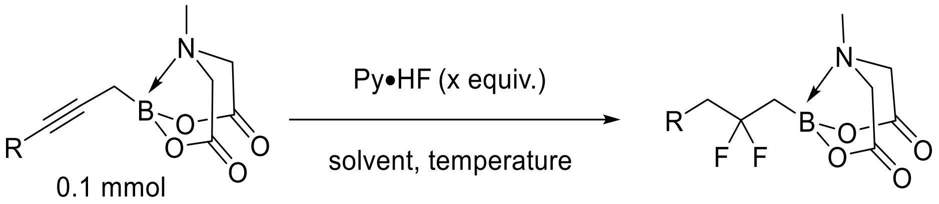

Entry	R	Nu⁻ (x equiv.)	solvent (y mL)	T (°C)	Yield (%) [a]
**1**	*n*Bu	Py•9HF (60.0)	neat	rt	66
**2**	*n*Bu	Py•9HF (100.0)	neat	rt	46
**3**	*n*Bu	Py•9HF (60.0)	DCM (0.5)	rt	62
**4**	*n*Bu	Py•9HF (60.0)	DCE (0.5)	rt	58
**5**	*n*Bu	Py•9HF (60.0)	$PhCF_3$ (0.5)	rt	54
**6**	*n*Bu	Py•9HF (60.0)	neat	0	65

[a]4-Iodoanisole as the internal standard to determine the ^{1}H-NMR yield.

Table S2. Reaction optimization for the synthesis of β-fluoro-γ-iodinated boronates.

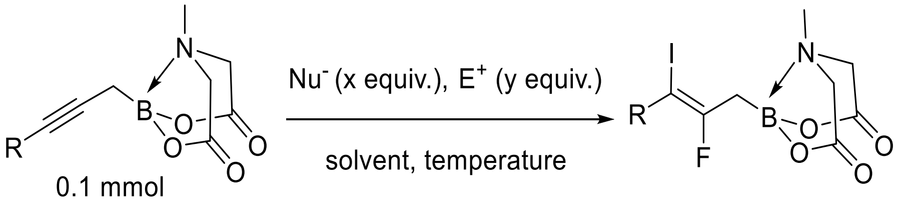

Entry	R	Nu⁻ (x equiv.)	E⁺ (y equiv.)	solvent (z mL)	T (°C)	Yield (%) [a]	Regio-ratio
**1**	*n*Bu	DIH (0.6)	$Et_3N{\cdot}3HF$ (3.0)	DCM	rt	28	7: 1
**2**	*n*Bu	DIH (0.6)	$Et_3N{\cdot}3HF$ (9.0)	DCM	rt	32	6: 1
**3**	*n*Bu	DIH (1.2)	$Et_3N{\cdot}3HF$ (3.0)	DCM	-20	36	9: 1
**4**	*n*Bu	DIH (1.2)	$Et_3N{\cdot}3HF$ (3.0)	DCM	-40	25	11:1
**5**	*n*Bu	DIH (1.2)	$Et_3N{\cdot}3HF$ (9.0)	DCM	-40	50	11:1
**6**	*n*Bu	DIH (1.5)	$Et_3N{\cdot}3HF$ (9.0)	DCM	-40	43	11:1
**7**	*n*Bu	DIH (2.0)	$Et_3N{\cdot}3HF$ (9.0)	DCM	-40	40	11:1

[a]4-Iodoanisole as the internal standard to determine the ^{1}H-NMR yield.

Table S3. Reaction optimization for the synthesis of β-chloro-γ-iodinated boronates.

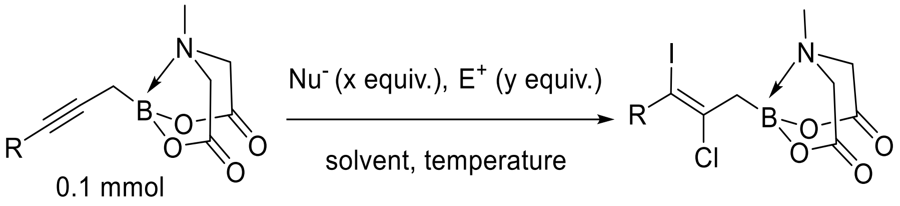

Entry	R	Nu⁻ (x equiv.)	E⁺ (y equiv.)	solvent (z mL)	T (°C)	Yield (%) [a]	Regio-ratio
**1**	*n*Bu	NIS (1.5)	LiCl (2.0)	DCM-HOAc	rt	80	1.3: 1
**2**	*n*Bu	NIS (1.5)	LiCl (2.0)	DCM-HOAc	-40	65	1.3: 1
**3**	*n*Bu	ICl (2.0)		DCM-HOAc	-15	91	2.4: 1
**4**	*n*Bu	ICl (2.0)		HOAc	-15	63	2.2: 1
**5**	*n*Bu	ICl (2.0)		MeCN (1.0)	0	37	2.6: 1
**6**	*n*Bu	ICl (2.0)		MeCN (1.0)	-15	48	3.6: 1
**7**	*n*Bu	ICl (2.0)		MeCN (0.5)	-15	52	3.7: 1

[a]4-Iodoanisole as the internal standard to determine the ^{1}H-NMR yield.

Table S4. Reaction optimization of α-boryl ketones.

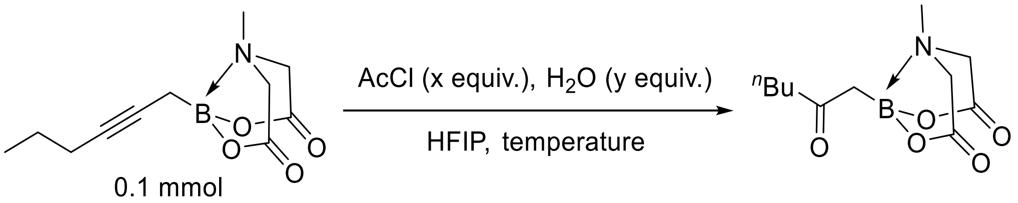

Entry	AcCl (x equiv.)	H_2O (y equiv.)	T (°C)	Yield (%) [a]
**1**	3.0	10.0	30	-
**2**	5.0	10.0	30	-
**3**	7.0	10.0	30	38%
**4**	9.0	10.0	30	55%
**5**	11.0	10.0	30	71%
**6**	15.0	10.0	30	92% (98%[b])
**7**	15.0	0.0	30	NR
**8**	15.0	10.0	0 °C	NR
**9**	15.0	10.0	-15 °C	NR
**10**	7.0	10.0	30	55%
**11**	7.0	15	30	<10%
**12**	7.0	20	30	11%
**13**	5.0	10.0	30	39%
**14**	5.0	5.0	30	76%
**15**	5.0	2.5	30	86%
**16**	5.0	1.2	30	86%

[a]4-Iodoanisole as the internal standard to determine the ^{1}H-NMR yield. [b]isolated yield.

5. General procedure for the synthesis of product

5.1 General procedure A for the synthesis of β-difluorinated boronates

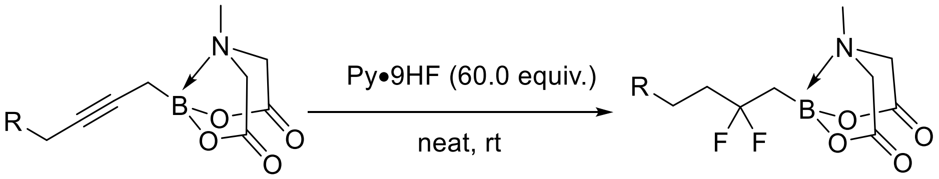

1-19

To a 10 mL of plastic tubing[4] charged with propargylic MIDA boronates (0.2 mmol, 1.0 equiv), was added Py·9HF (60.0 equiv) in one portion. The reaction was allowed to stir at room temperature for 3 h. The reaction mixture was quenched by slowly adding basic alumina suspended in dichloromethane (1.8 g). The suspension was filtered and washed with 25 mL of dichloromethane. The combined filtrate was concentrated in vacuo and purified by column chromatography to afford the pure product **1-19**.

5.2 General procedure B for the synthesis of α-boryl ketones

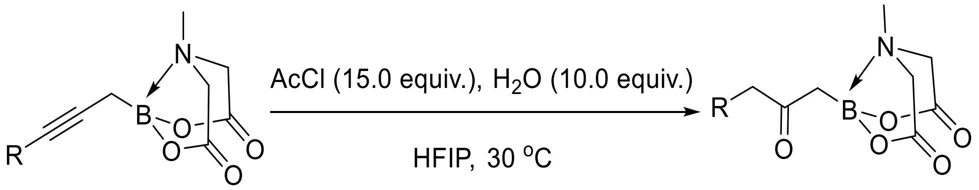

The propargylic MIDA boronates (0.2 mmol, 1.0 equiv.) were added to a 15 mL oven-dried pressure tube equipped with a stir bar. HFIP (2.0 mL) and H_2O (2.0 mmol, 10.0 equiv.) were added to the tube under air. The solution was stirred for 15 seconds and then add AcCl (3.0 mmol, 15.0 equiv.) was added following sealing the tube quickly with a thread plug. The solution was stirred at 30 °C until the propargyl boronic esters was used up by TLC monitoring. The solvent was removed in vacuo and the crude product was purified by flash column chromatography on silica with an eluent (PE / EtOAc from 1:2 to 1:4, v/v) to afford the pure product as a semi solid **20-39**.

5.3 General procedure C for the synthesis of β-fluoro-γ-iodinated boronates

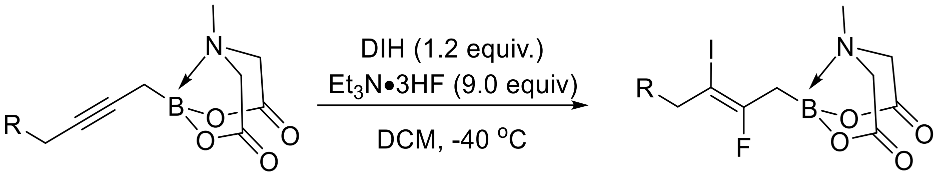

40-46

To a 10 mL of plastic tubing equipped with a stirring bar, were added propargylic MIDA boronates (0.3 mmol, 1.0 equiv), 1,3-diiodo-5,5-dimethylhydantoin/DIH (0.36 mmol, 1.2 equiv), CH_2Cl_2 (1.5 mL) and Et_3N·3HF (423 μL, 9.0 equiv). The solution was stirred at -40 °C overnight. The resulting mixture was quenched with saturated

$Na_2S_2O_3$ solution and then extracted with EtOAc. The organic phase was washed with saturated NaCl solution three times. The combined organic layer was dried over anhydrous Na_2SO_4 and concentrated under reduced pressure. The residue was purified by column chromatography to afford the pure product **40-46**.

5.4 General procedure D for the synthesis of β-chloro-γ-iodinated boronates

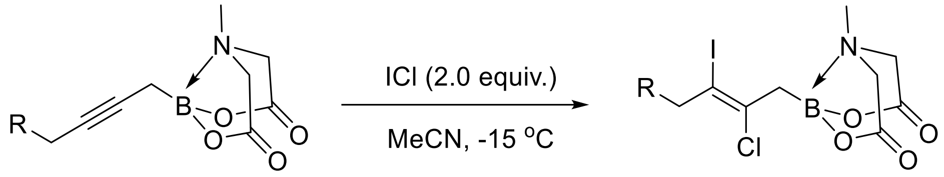

47-52

To a 15-mL screw cap vial equipped with a stir bar were added propargylic MIDA boronates (0.2 mmol, 1.0 equiv) and MeCN (1.0 mL). The mixture was cooled to -15 °C. ICl (2.0 equiv. 1.0 M in DCM) was added in one portion and the bottle was sealed. After stirring overnight, the resulting mixture was quenched with saturated $Na_2S_2O_3$ solution and then extracted with EtOAc for three times. The combined filtrate was concentrated in vacuo and purified by column chromatography to afford the pure product **47-52**.

6. Derivatization of the products

The compound **67** were prepared and its spectroscopic data was consistent with reported literature.[5]

6.1 Synthesis of compound **55**

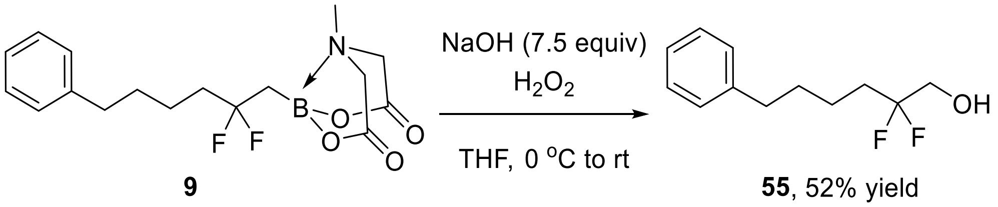

9 **55**, 52% yield

Under an argon atmosphere, a 15-mL Schlenk tube equipped with a stirring bar, were added alkyl difluorinated MIDA boronates **9** (0.2 mmol, 70.6 mg 1.0 equiv), and THF (2.0 mL). Under 0 °C, 3 M NaOH in H_2O (7.5 equiv, 60 mg, 0.5 mL) was added into the Schlenk tube dropwise, then 30% H_2O_2 (0.5 mL) was added dropwise. The solution was stirred at room temperature for 2 h, and the complete consumption of **9** as monitored by TLC analysis. The resulting mixture was quenched with saturated $Na_2S_2O_3$ solution under 0 °C and then extracted with Et_2O for three times. The combined organic layer was dried over anhydrous Na_2SO_4 and concentrated under reduced pressure. The crude residue was purified by flash column chromatography on silica gel to afford the product. (**55**, yield: 52%)

6.2 Synthesis of compound **56**

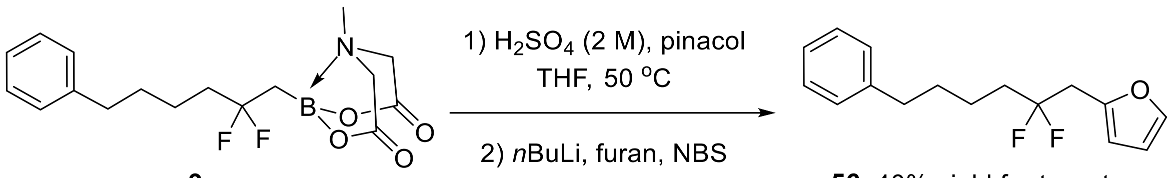

9 **56**, 40% yield for two steps

Under an argon atmosphere, a 15-mL Schlenk tube equipped with a stirring bar, were added alkyl difluorinated MIDA boronates **9** (0.2 mmol, 70.6 mg 1.0 equiv) and THF (1.5 mL), 2 M H_2SO_4 in H_2O (2.0 equiv, 22 μL, 0.2 mL) was added into mixture slowly, then pinacol (1.2 equiv, 29 mg) in THF (0.5 mL) was added subsequently. The solution was stirred at 50 °C overnight. After cooled to room temperature, the resulting mixture was quenched by H_2O and extracted with EtOAc for three times. The combined organic layer was dried over anhydrous Na_2SO_4 and concentrated under reduced pressure. The crude reaction mixture was used directly in the next step without further purification.

Under an argon atmosphere, a 15-mL Schlenk tube equipped with a stirring bar, were added furan (1.5 equiv, 22 μL) and THF (1.5 mL). The mixture was cooled to -78 °C, the *n*BuLi (1.5 equiv, 0.12 mL) was added dropwise. The solution was stirred at room temperature for 1 h. Then the mixture was cooled to -78 °C, crude reaction mixture of previous step in THF (1.5 mL) was added dropwise and stirred at -78 °C for 1 h. NBS (1.2 equiv, 54 mg) in THF (1.0 mL) was added dropwise and the mixture was stirred at -78 °C for 12 h. The resulting mixture was quenched with saturated $Na_2S_2O_3$ solution and then extracted with Et_2O for three times. The combined organic layer was dried over anhydrous Na_2SO_4 and concentrated under reduced pressure. The crude residue was purified by flash column chromatography on silica gel to afford the product. (**56**, yield: 40% for two steps)

6.3 Synthesis of compound **57**

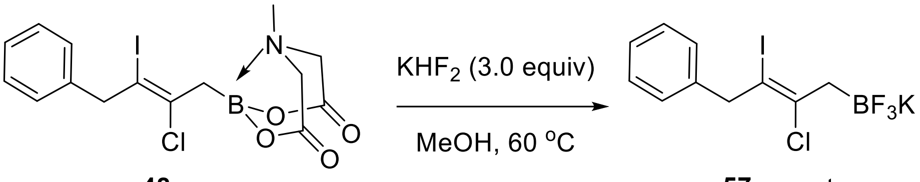

48 **57**, quant.

To a 15-mL Schlenk tube equipped with a stirring bar were added iodochlorinated alkyl MIDA boronates **48** (0.1 mmol, 44.7 mg 1.0 equiv), 3.0 M KHF_2 in H_2O (3.0 equiv. 24 mg, 0.1 mL) and MeOH (4.0 mL). The solution was stirred at 60 °C for 5 h. The resulting mixture was concentrated via rotary evaporator. After the evaporation of the solvent under vacuum, the obtained solid was triturated with acetone and filtered through a plug of Celite. The acetone solution was evaporated to yield a solid. The white solid was dissolved in acetone (3 mL) and precipitated with cold Et_2O (50 mL) to yield a white amorphous solid (**57**, yield: quant.)

6.4 Synthesis of compound **58**

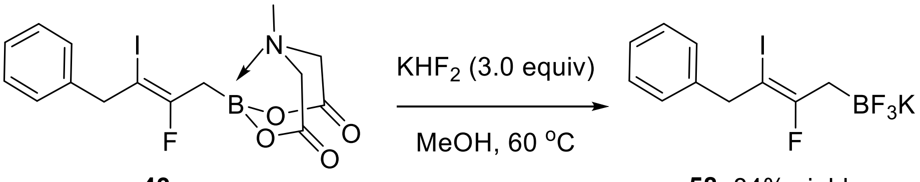

43 **58**, 84% yield

To a 15-mL Schlenk tube equipped with a stirring bar were added iodochlorinated alkyl MIDA boronates **48** (0.2 mmol, 86.2 mg 1.0 equiv), 3.0 M KHF_2 in H_2O (3.0 equiv. 47 mg, 0.2 mL) and MeOH (8.0 mL). The solution was stirred at 60 °C overnight. The resulting mixture was concentrated via rotary evaporator. After the evaporation of the solvent under vacuum, the obtained solid was triturated with acetone and filtered through a plug of Celite. The acetone solution was evaporated to yield a solid. The white solid was dissolved in acetone and precipitated with cold Et_2O to yield a white amorphous solid (**58**, yield: 84%)

6.5 Synthesis of compound **59**

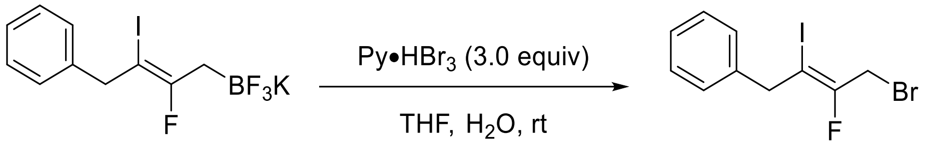

58

59, 50% yield

To a 15-mL Schlenk tube equipped with a stirring bar, were added compound **58** (0.084 mmol, 32 mg, 1.0 equiv), H_2O (0.5 mL) and THF (0.5 mL). Py·HBr_3 (3.0 equiv, 80.6 mg) was added into the reaction mixture. The solution was stirred at room temperature for 24 h. The resulting mixture was extracted with Et_2O for three times. The combined organic layer was dried over anhydrous Na_2SO_4 and concentrated under reduced pressure. The crude residue was purified by flash column chromatography on silica gel to afford the product. (**59**, yield: 59%)

6.6 Synthesis of compound **60**

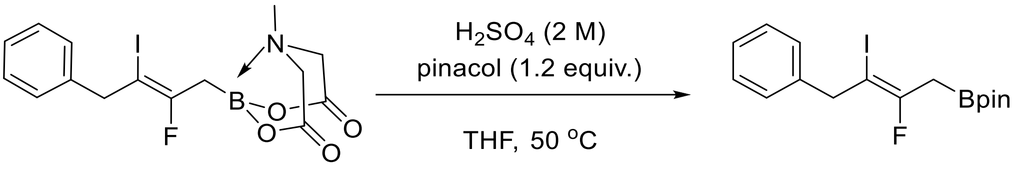

43

60, 60% yield

Under an argon atmosphere, a 15-mL Schlenk tube equipped with a stirring bar, were added alkyl iodofluorinated MIDA boronates **43** (0.2 mmol, 86.2 mg 1.0 equiv) and THF (1.5 mL), 2 M H_2SO_4 in H_2O (2.0 equiv, 22 μL, 0.2 mL) was added into mixture slowly, then pinacol (1.2 equiv, 29 mg) in THF (0.5 mL) was added subsequently. The solution was stirred at 50 °C overnight. After cooled to room temperature, the resulting mixture was quenched by H_2O and extracted with EtOAc for three times. The combined organic layer was dried over anhydrous Na_2SO_4 and concentrated under reduced pressure. The crude residue was purified by flash column chromatography on silica gel to afford the product. (**60**, yield: 60%)

6.7 Synthesis of compound **61**

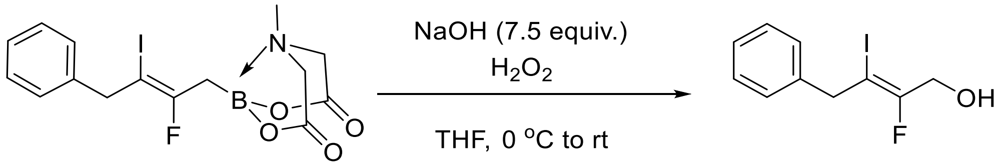

43

61, 75% yield

Under an argon atmosphere, a 15-mL Schlenk tube equipped with a stirring bar, were added alkyl iodofluorinated MIDA boronates **43** (0.2 mmol, 86.2 mg 1.0 equiv), and THF (2.0 mL). Under 0 °C, 3 M NaOH

in H_2O (7.5 equiv, 60 mg, 0.5 mL) was added into the Schlenk tube dropwise, then 30% H_2O_2 (0.5 mL) was added dropwise. The solution was stirred at room temperature for 4 h, and the complete consumption of **43** as monitored by TLC analysis. The resulting mixture was quenched with saturated $Na_2S_2O_3$ solution under 0 °C and then extracted with Et_2O for three times. The combined organic layer was dried over anhydrous Na_2SO_4 and concentrated under reduced pressure. The crude residue was purified by flash column chromatography on silica gel to afford the product. (**61**, yield: 75%)

6.8 Synthesis of compound **62**

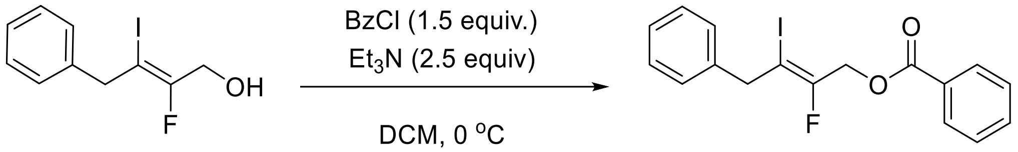

61 **62**, 66% yield

To a 15-mL Schlenk tube equipped with a stirring bar, were added (E)-2-fluoro-3-iodo-4-phenylbut-2-en-1-ol **61** (0.54 mmol, 158 mg 1.0 equiv), and DCM (2.7 mL). Under 0 °C, Et_3N (2.5 equiv, 137 mg) was added into the Schlenk tube dropwise, then BzCl (1.5 equiv. 114 mg) was added dropwise. The solution was stirred at 0 °C for 2 h, and the complete consumption of **61** as monitored by TLC analysis. The resulting mixture was quenched with 1 M HCl solution under 0 °C and then extracted with EtOAc for three times. The combined organic layer was dried over anhydrous Na_2SO_4 and concentrated under reduced pressure. The crude residue was purified by flash column chromatography on silica gel to afford the product. (**62**, yield: 66%)

6.9 Synthesis of compound **63**

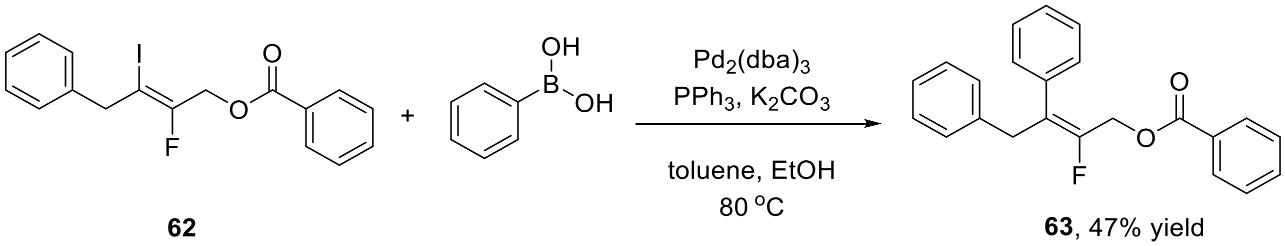

62 **63**, 47% yield

To a 15-mL Schlenk tube equipped with a stirring bar, were added compound **62** (0.1 mmol, 39.6 mg 1.0 equiv), phenylboronic acid (1.5 equiv. 18.3 mg), $Pd_2(dba)_3$ (0.05 equiv. 4.6 mg), PPh_3 (0.2 equiv. 5.3 mg), K_2CO_3 (3.0 equiv. 41.5 mg), EtOH (0.1 mL) and toluene (1.0 mL). The solution was stirred at 80 °C for 12 h, the resulting mixture was quenched by H_2O and extracted with EtOAc for three times. The combined organic layer was dried over anhydrous Na_2SO_4 and concentrated under reduced pressure. The crude residue was purified by flash column chromatography on silica gel to afford the product. (**63**, yield: 47%)

6.10 Synthesis of compound **64**

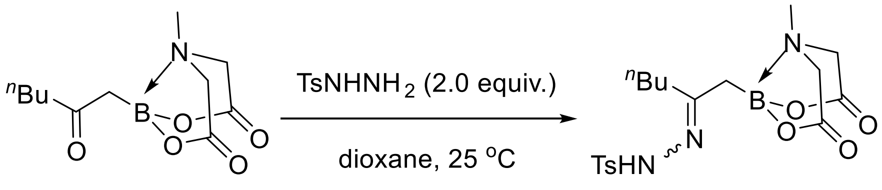

20 **64**, 25%

To a 15-mL screw cap vial equipped with a stirring bar, MIDA boronates **20** (25.5 mg, 0.1 mmol, $TsNHNH_2$

(37.2 mg, 0.2 mmol) and dioxane (1.0 mL) were added. The solution was stirred at 25 °C for 5 h. Removed the solvent in vacuo and the crude residue was purified by flash column chromatography on silica gel to afford the product. The compound **20** could be recovered in more than 50% yield. (**64**, yield: 25%)

6.11 Synthesis of compound **65**

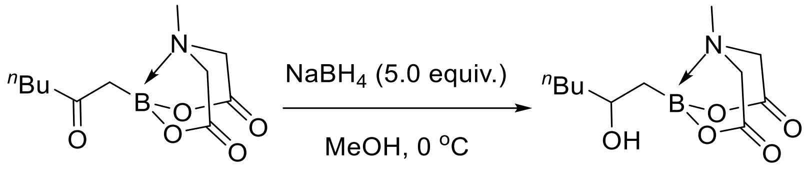

20 **65**, 96%

To a 15-mL screw cap vial equipped with a stirring bar, MIDA boronates **20** (0.2 mmol, 51.0 mg 1.0 equiv) and MeOH (2.0 mL) were added. Under 0 °C, $NaBH_4$ (38.0 mg, 5.0 equiv.) was added and the solution was stirred at 0 °C until the consumption of **20** was complete (monitored by TLC analysis). The resulting mixture was quenched with H_2O under 0 °C and then extracted with DCM for three times. The combined organic layer was dried over anhydrous Na_2SO_4 and concentrated under reduced pressure. The crude residue was purified by flash column chromatography on silica gel to afford the product. (**65**, yield: 96%)

6.12 Synthesis of compound **66**

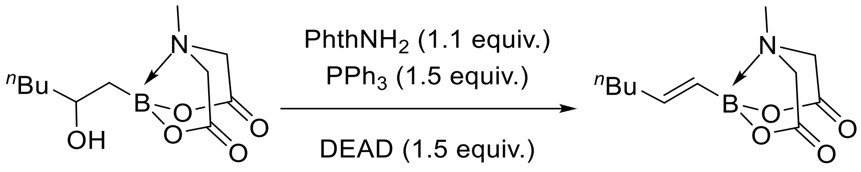

65 **66**, 77%

To a 15-mL screw cap vial equipped with a stirring bar, MIDA boronates **65** (25.7 mg, 0.1 mmol, PPh_3 (40.0 mg, 0.15 mmol), $PhthNH_2$ (17.0 mg, 0.11 mmol) and THF (1.0 mL) were added. Under 0 °C, DEAD (26.0 mg, 5.0 equiv.) was added and the solution was stirred at 0 °C until the consumption of **65** was complete (monitored by TLC analysis). After removed the solvent in vacuo, the crude residue was purified by flash column chromatography on silica gel to afford the product. (**66**, yield: 77%)

6.13 Synthesis of compound **67**

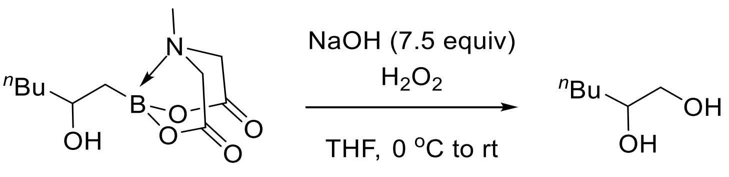

65 **67,** 86%

To a 15-mL screw cap vial equipped with a stirring bar, were added MIDA boronates **65** (0.1 mmol, 25.7 mg 1.0 equiv), and THF (1.0 mL). Under 0 °C, 3 M NaOH in H_2O (7.5 equiv, 30.0 mg, 0.25 mL) was added into the screw cap vial dropwise, then 30% H_2O_2 (0.25 mL) was added dropwise. The solution was stirred at room temperature for 2 h, and the complete consumption of **67** as monitored by TLC analysis. The resulting mixture was quenched with saturated $Na_2S_2O_3$ solution under 0 °C and then extracted with Et_2O for three times. The combined organic layer was dried over anhydrous Na_2SO_4 and concentrated under reduced pressure. The crude

residue was purified by flash column chromatography on silica gel to afford the product. (**67**, yield: 86%)

7. Limitation of substrates

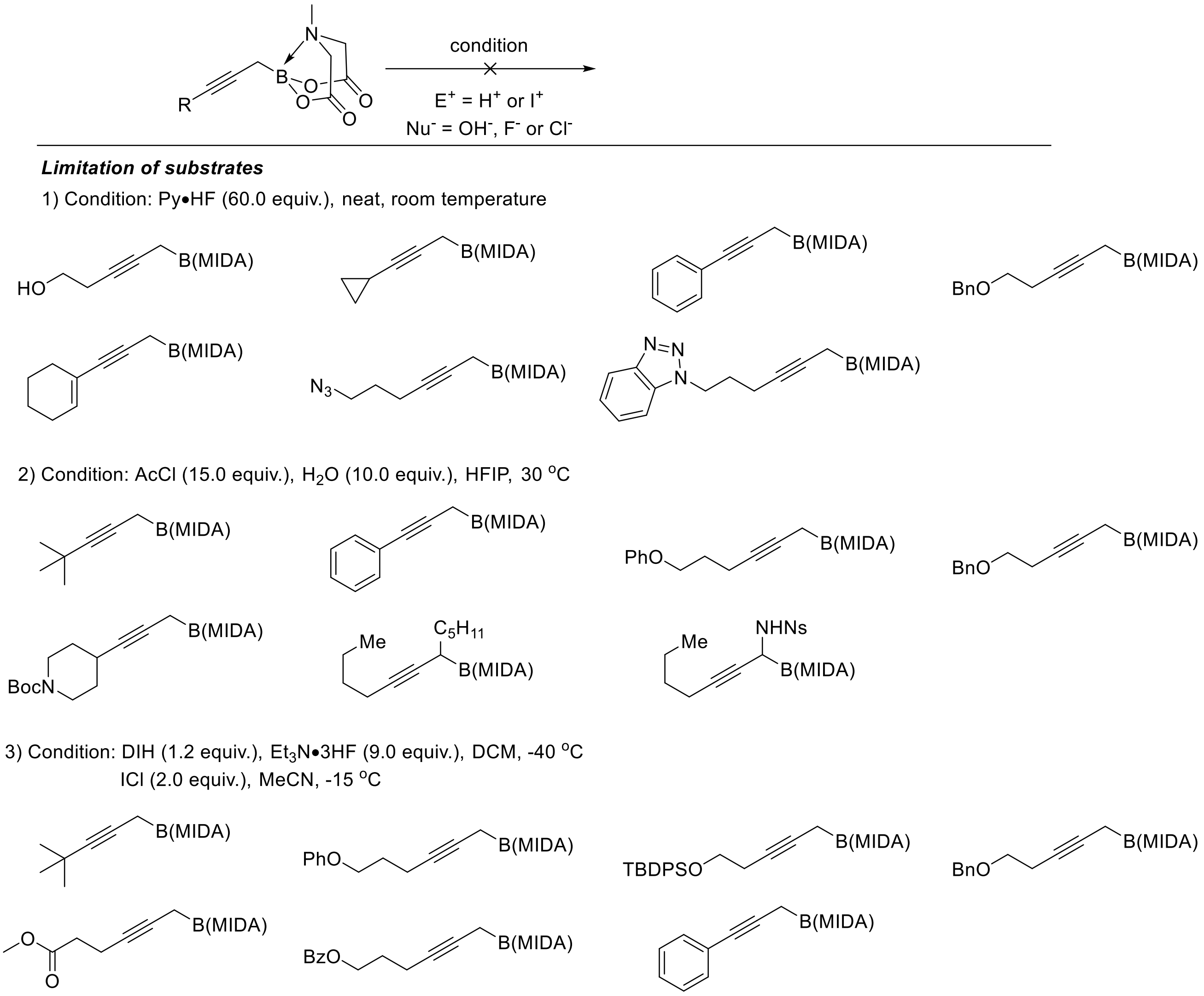

Figure S1. Limitation of substrates

8. Characterization of the products

2-(2,2-difluoroheptyl)-6-methyl-1,3,6,2-dioxazaborocane-4,8-dione (1)

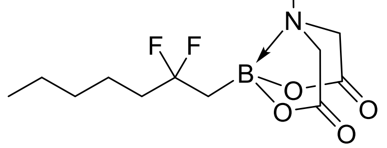

Following the general procedure **A**, the product **1** was obtained in 61% yield as a white solid after column chromatography (eluent = petroleum ether/ ethyl acetate 1:3 v/v). R_F = 0.30 (PE: ethyl acetate = 1:4). ^{1}H NMR (400 MHz, Acetone-d_6) δ 4.24 (d, *J* = 17.0 Hz, 1H), 4.03 (d, *J* = 16.9 Hz, 1H), 3.16 (s,

2H), 2.02 – 1.85 (m, 1H), 1.59 – 1.42 (m, 2H), 1.40 – 1.25 (m, 2H), 0.91 (t, 1H). ^{11}B NMR (128 MHz, Acetone-d_6) δ 11.40. ^{19}F NMR (376 MHz, Acetone-d_6) δ -84.89. ^{13}C NMR (101 MHz, Acetone-d_6) δ 167.6, 127.1 (t, J = 238.3 Hz), 61.9, 45.8, 37.9 (t, J = 25.9 Hz), 31.4, 24.3 – 21.1 (m), 13.3. **ESI-MS:** calcd for $C_{12}H_{20}NO_4BF_2Na$ [M + Na]$^+$: 314.1346, found: 314.1352.

2-(2,2-difluoro-6-methylheptyl)-6-methyl-1,3,6,2-dioxazaborocane-4,8-dione (2)

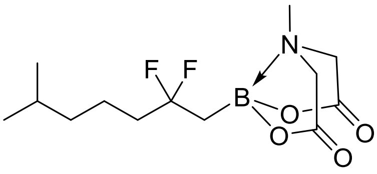

Following the general procedure **A**, the product **2** was obtained in 61% yield as a white solid after column chromatography (eluent = petroleum ether/ ethyl acetate 1:3 v/v). R_F = 0.34 (PE: ethyl acetate = 1:4). ^1H NMR (400 MHz, Acetone-d_6) δ 4.24 (d, J = 16.9 Hz, 2H), 4.03 (d, J = 16.8 Hz, 2H), 3.16 (s, 3H), 2.00 – 1.82 (m, 2H), 1.61 – 1.41 (m, 5H), 1.27 – 1.18 (m, 2H), 0.90 (s, 3H), 0.88 (s, 3H). ^{11}B NMR (128 MHz, Acetone-d_6) δ 11.39. ^{19}F NMR (376 MHz, Acetone-d_6) δ -84.87. ^{13}C NMR (126 MHz, Acetone-d_6) δ 167.6, 126.1 (t), 61.9, 45.8, 38.5, 38.1 (t, J = 26.0 Hz), 27.6, 21.9, 20.4 (t, J = 4.7 Hz). **ESI-MS:** calcd for $C_{13}H_{22}NO_4BF_2Na$ [M + Na]$^+$: 328.1503, found: 328.1507.

2-(2,2-difluorononyl)-6-methyl-1,3,6,2-dioxazaborocane-4,8-dione (3)

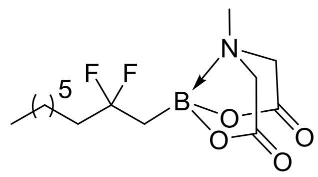

Following the general procedure **A**, the product **3** was obtained in 63% yield as a white solid after column chromatography (eluent = petroleum ether/ ethyl acetate 1:3 v/v). R_F = 0.38 (PE: ethyl acetate = 1:4). ^1H NMR (400 MHz, Acetone-d_6) δ 4.25 (d, J = 16.9 Hz, 2H), 4.04 (d, J = 16.9 Hz, 2H), 3.17 (s, 3H), 2.04 – 1.86 (m, 2H), 1.63 – 1.41 (m, 4H), 1.38 – 1.24 (m, 8H), 0.89 (t, 3H). ^{11}B NMR (128 MHz, Acetonitrile-d_3) δ 11.43. ^{19}F NMR (376 MHz, Acetone-d_6) δ -84.87. ^{13}C NMR (101 MHz, Acetonitrile-d_3) δ 168.6, 128.1 (t, J = 238.1 Hz), 62.6, 46.8, 38.8 (t, J = 25.9 Hz), 32.2, 29.8, 29.6, 23.3 (t, J = 4.8 Hz), 23.1, 14.2. **ESI-MS:** calcd for $C_{14}H_{24}NO_4BF_2Na$ [M + Na]$^+$: 342.1659, found: 342.1656.

2-(3-cyclopentyl-2,2-difluoropropyl)-6-methyl-1,3,6,2-dioxazaborocane-4,8-dione (4)

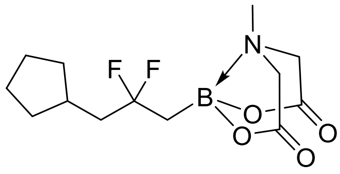

Following the general procedure **A**, the product **4** was obtained in 35% yield as a white solid after column chromatography (eluent = petroleum ether/ ethyl acetate 1:3 v/v). R_F = 0.40 (PE: ethyl acetate = 1:4). ^1H NMR (500 MHz, DMSO-d_6) δ 4.21 (d, J = 16.9 Hz, 2H), 3.96 (d, J = 17.0 Hz, 2H), 2.86 (s, 3H), 2.02 – 1.85 (m, 3H), 1.80 (dq, J = 10.0, 6.0 Hz, 2H), 1.59 (q, J = 7.0 Hz, 2H), 1.52 – 1.35 (m, 4H), 1.16 – 1.04 (m, 2H). ^{11}B NMR (128 MHz, Acetone-d_6) δ 11.41. ^{19}F NMR (376 MHz, Acetone-d_6) δ -83.37. ^{13}C NMR (126 MHz, Acetone-d_6) δ 167.6, 127.2 (t, J = 238.8 Hz), 61.9, 45.9, 43.5 (t, J = 25.2 Hz), 34.8 (t, J = 3.4 Hz), 33.1, 24.5. **ESI-MS:** calcd for $C_{13}H_{20}NO_4BF_2Na$ [M + Na]$^+$: 326.1346, found: 326.1349.

2-(3-cyclohexyl-2,2-difluoropropyl)-6-methyl-1,3,6,2-dioxazaborocane-4,8-dione (5)

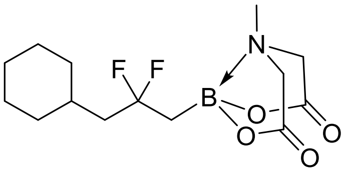

Following the general procedure **A**, the product **5** was obtained in 66% yield as a white solid after column chromatography (eluent = petroleum ether/ ethyl acetate 1:3 v/v). R_F = 0.36 (PE: ethyl acetate = 1:4). ^1H NMR (500 MHz, Acetonitrile-d_3) δ 3.96 (d, J = 17.0 Hz, 2H), 3.79 (d, J = 17.0 Hz, 2H), 2.89 (s,

3H), 1.87 – 1.73 (m, 4H), 1.72 – 1.57 (m, 4H), 1.46 (t, J = 19.6 Hz, 2H), 1.36 – 1.22 (m, 2H), 1.22 – 1.11 (m, 1H), 1.08 – 0.94 (m, 2H). ^{11}B NMR (160 MHz, Acetonitrile-d_3) δ 11.24. ^{19}F NMR (376 MHz, Acetonitrile-d_3) δ -82.42. ^{13}C NMR (101 MHz, DMSO-d_6) δ 169.1, 128.0 (t), 62.0, 46.2, 45.1 (t, J = 24.3 Hz), 33.9, 32.6, 26.2, 26.2. **ESI-MS:** calcd for $C_{14}H_{22}NO_4BF_2Na$ [M + Na]$^+$: 340.1503, found: 340.1507.

2-(6-chloro-2,2-difluorohexyl)-6-methyl-1,3,6,2-dioxazaborocane-4,8-dione (6)

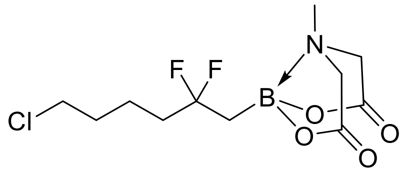

Following the general procedure **A**, the product **6** was obtained in 65% yield as a white solid after column chromatography (eluent = petroleum ether/ ethyl acetate 1:3 v/v). R_F = 0.41 (PE: ethyl acetate = 1:4). ^{1}H NMR (500 MHz, Acetone-d_6) δ 4.25 (dd, J = 16.9, 1.7 Hz, 2H), 4.04 (dd, J = 16.9, 1.8 Hz, 2H), 3.64 (t, J = 6.5 Hz, 2H), 3.16 (s, 3H), 2.04 – 1.93 (m, 2H), 1.83 (p, J = 7.0 Hz, 2H), 1.64 (h, J = 7.3 Hz, 2H), 1.54 (t, J = 19.0 Hz, 2H). ^{11}B NMR (128 MHz, Acetone-d_6) δ 11.34. ^{19}F NMR (376 MHz, Acetonitrile-d_3) δ -85.16. ^{13}C NMR (126 MHz, Acetone-d_6) δ 167.6, 126.9 (t, J = 238.5 Hz), 61.9, 45.9, 44.6, 37.1 (t, J = 26.2 Hz), 32.2, 20.0 (t, J = 4.9 Hz). The spectroscopic data of known compounds were consistent with the reported values.[6]

methyl 6,6-difluoro-7-(6-methyl-4,8-dioxo-1,3,6,2-dioxazaborocan-2-yl)heptanoate (7)

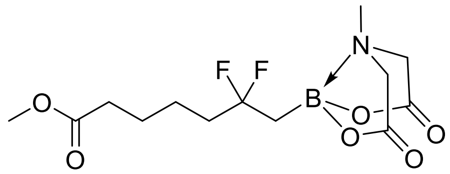

Following the general procedure **A**, the product **7** was obtained in 62% yield as a white solid after column chromatography (eluent = petroleum ether/ ethyl acetate 1:3 v/v). R_F = 0.45 (PE: ethyl acetate = 1:4). ^{1}H NMR (500 MHz, Acetone-d_6) δ 4.24 (d, J = 16.9 Hz, 2H), 4.03 (d, J = 16.9 Hz, 2H), 3.61 (s, 3H), 3.15 (s, 3H), 2.33 (t, J = 7.4 Hz, 2H), 2.02 – 1.89 (m, 2H), 1.64 (p, J = 7.5 Hz, 2H), 1.58 – 1.42 (m, 4H). ^{11}B NMR (160 MHz, Acetone-d_6) δ 11.42. ^{19}F NMR (376 MHz, Acetone-d_6) δ -84.94. ^{13}C NMR (126 MHz, Acetone-d_6) δ 173.1, 167.6, 127.0 (t, J = 238.5 Hz), 61.9, 50.6, 45.8, 37.6 (t, J = 26.1 Hz), 33.2, 24.5, 22.1 (t, J = 4.8 Hz). **ESI-MS:** calcd for $C_{13}H_{20}NO_6BF_2Na$ [M + Na]$^+$: 358.1242, found: 358.1242.

6,6-difluoro-7-(6-methyl-4,8-dioxo-1,3,6,2-dioxazaborocan-2-yl)heptanenitrile (8)

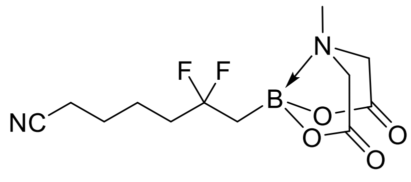

Following the general procedure **A**, the product **8** was obtained in 55% yield as a white solid after column chromatography (eluent = petroleum ether/ ethyl acetate 1:3 v/v). R_F = 0.24 (PE: ethyl acetate = 1:4). ^{1}H NMR (500 MHz, Acetone-d_6) δ 4.25 (d, J = 16.9 Hz, 2H), 4.04 (d, J = 16.9 Hz, 2H), 3.17 (s, 3H), 2.51 (t, J = 6.9 Hz, 2H), 2.04 – 1.93 (m, 2H), 1.71 (p, J = 7.1 Hz, 2H), 1.64 (q, J = 8.5 Hz, 2H), 1.54 (t, J = 19.0 Hz, 2H). ^{11}B NMR (160 MHz, Acetone-d_6) δ 11.31. ^{19}F NMR (376 MHz, Acetone-d_6) δ -85.15. ^{13}C NMR (126 MHz, Acetone-d_6) δ 167.6, 126.8 (t, J = 238.7 Hz), 119.6, 61.9, 45.9, 37.0 (t, J = 26.3 Hz), 25.1, 21.8 (t, J = 4.8 Hz), 16.2. **ESI-MS:** calcd for $C_{12}H_{17}N_2O_4BFNa$ [M + Na]$^+$: 325.1142, found: 325.1146.

2-(2,2-difluoro-6-phenylhexyl)-6-methyl-1,3,6,2-dioxazaborocane-4,8-dione (9)

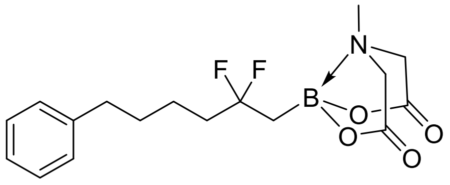

Following the general procedure **A**, the product **9** was obtained in 62% yield as a white solid after column chromatography (eluent = petroleum ether/ ethyl acetate 1:3 v/v). R_F = 0.35 (PE: ethyl acetate = 1:4). ^{1}H NMR (500 MHz, Acetone-d_6) δ 7.29 – 7.24 (m, 2H), 7.24 – 7.20 (m, 2H), 7.20 – 7.11 (m, 1H), 4.24 (d, J = 16.9 Hz, 2H), 4.02 (d, J = 16.9 Hz, 2H), 3.15 (s, 3H), 2.64 (t, 2H), 2.06 – 1.92 (m, 2H), 1.67 (p, J = 7.5 Hz, 2H), 1.58 – 1.44 (m, 4H). ^{11}B NMR (160 MHz, Acetone-d_6) δ 11.30. ^{19}F NMR (376 MHz, Acetone-d_6) δ -84.97. ^{13}C NMR (126 MHz, Acetone-d_6) δ 167.6, 142.4, 128.3, 128.2, 127.1 (t, J = 238.4 Hz), 125.6, 61.9, 45.9, 37.8 (t, J = 26.0 Hz), 35.4, 31.2, 22.2 (t, J = 4.7 Hz). **ESI-MS:** calcd for $C_{17}H_{22}NO_4BF_2Na$ [M + Na]$^+$: 376.1503, found: 376.1503.

2-(2,2-difluoro-4-phenylbutyl)-6-methyl-1,3,6,2-dioxazaborocane-4,8-dione (10)

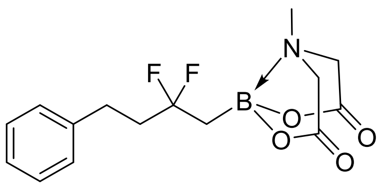

Following the general procedure **A**, the product **10** was obtained in 52% yield as a white solid after column chromatography (eluent = petroleum ether/ ethyl acetate 1:3 v/v). R_F = 0.38 (PE: ethyl acetate = 1:4). ^{1}H NMR (400 MHz, Acetone-d_6) δ 7.17 (d, J = 5.6 Hz, 4H), 7.12 – 7.03 (m, 1H), 4.15 (d, J = 16.9 Hz, 2H), 3.95 (d, J = 16.9 Hz, 2H), 3.07 (s, 3H), 2.70 – 2.64 (m, 2H), 2.23 – 2.06 (m, 2H), 1.49 (t, J = 18.9 Hz, 2H). ^{11}B NMR (128 MHz, Acetone-d_6) δ 11.21. ^{19}F+ NMR (376 MHz, Acetone-d_6) δ -84.94. ^{13}C NMR (126 MHz, Acetonitrile-d_3) δ 168.4, 141.8, 129.1, 128.9, 127.3 (t, J = 238.9 Hz), 126.6, 62.4, 46.6, 40.6 (t, J = 26.0 Hz), 29.3 (t, J = 5.3 Hz). **ESI-MS:** calcd for $C_{15}H_{18}NO_4BF_2Na$ [M + Na]$^+$: 348.1190, found: 348.1188.

2-(2,2-difluoro-6-phenoxyhexyl)-6-methyl-1,3,6,2-dioxazaborocane-4,8-dione (11)

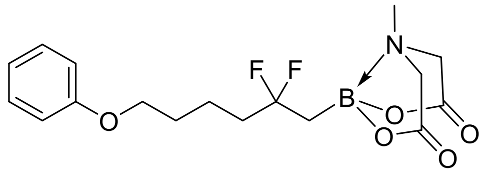

Following the general procedure **A**, the product **11** was obtained in 56% yield as a white solid after column chromatography (eluent = petroleum ether/ ethyl acetate 1:3 v/v). R_F = 0.40 (PE: ethyl acetate = 1:4). ^{1}H NMR (500 MHz, DMSO-d_6) δ 7.35 – 7.16 (m, 2H), 6.92 (dd, J = 12.8, 7.5 Hz, 3H), 4.22 (d, J = 17.2 Hz, 2H), 4.04 – 3.88 (m, 4H), 2.87 (s, 3H), 2.05 – 1.85 (m, 2H), 1.75 (p, J = 6.9 Hz, 2H), 1.57 (p, J = 8.1, 7.6 Hz, 2H), 1.43 (t, J = 19.1 Hz, 2H). ^{11}B NMR (128 MHz, Acetone-d_6) δ 11.43. ^{19}F NMR (376 MHz, Acetone-d_6) δ -85.02. ^{13}C NMR (126 MHz, DMSO-d_6) δ 169.2, 159.1, 129.9, 127.7 (t, J = 238.6 Hz), 120.9, 114.9, 67.5, 62.0, 46.2, 37.8 (t, J = 26.1 Hz), 28.9, 19.5 (t, J = 4.3 Hz). **ESI-MS:** calcd for $C_{17}H_{22}NO_5BF_2Na$ [M + Na]$^+$: 392.1452, found: 392.1451.

2-(2,2-difluoro-6-(4-methoxyphenoxy)hexyl)-6-methyl-1,3,6,2-dioxazaborocane-4,8-dione (12)

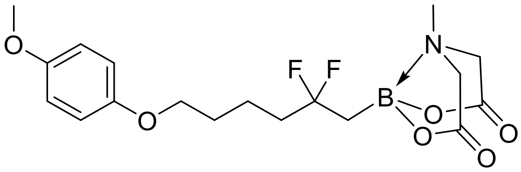

Following the general procedure **A**, the product **12** was obtained in 57% yield as a white solid after column chromatography (eluent = petroleum ether/ ethyl acetate 1:3 v/v). R_F = 0.41 (PE: ethyl acetate = 1:4). ^{1}H NMR (500 MHz, DMSO-d_6) δ 6.89 – 6.81 (m, 4H), 4.22 (d, J = 17.0 Hz, 2H), 3.97 (d, J = 17.0 Hz, 2H), 3.90 (t, J = 6.4 Hz, 2H), 3.69 (s, 3H), 2.87 (s, 3H), 1.94

(tt, J = 16.6, 8.0 Hz, 2H), 1.71 (p, J = 6.7 Hz, 2H), 1.61 – 1.51 (m, 2H), 1.43 (t, J = 19.2 Hz, 2H). ^{11}B NMR (128 MHz, Acetonitrile-d_3) δ 11.20. ^{19}F NMR (376 MHz, Acetonitrile-d_3) δ -85.68. ^{13}C NMR (101 MHz, Acetonitrile-d_3) δ 168.4, 154.4, 153.7, 127.8 (t, J = 238.3 Hz), 116.0, 115.1, 68.6, 62.4, 55.8, 46.6, 38.3 (t, J = 26.0 Hz), 29.3, 19.8 (t, J = 4.9 Hz). **ESI-MS:** calcd for $C_{18}H_{24}NO_6BF_2Na$ [M + Na]$^+$: 422.1558, found: 422.1554.

4-((5,5-difluoro-6-(6-methyl-4,8-dioxo-1,3,6,2-dioxazaborocan-2-yl)hexyl)oxy)benzonitrile (13)

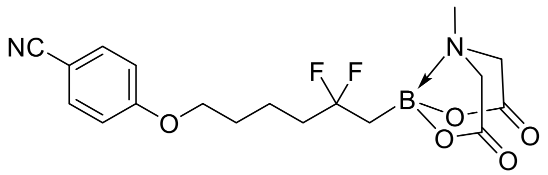

Following the general procedure **A**, the product **13** was obtained in 52% yield as a white solid after column chromatography (eluent = petroleum ether/ ethyl acetate 1:3 v/v). R_F = 0.25 (PE: ethyl acetate = 1:4). ^{1}H NMR (500 MHz, DMSO-d_6) δ 7.75 (d, J = 8.8 Hz, 2H), 7.11 (d, J = 8.8 Hz, 2H), 4.22 (d, J = 17.0 Hz, 2H), 4.08 (t, J = 6.4 Hz, 2H), 3.97 (d, J = 17.0 Hz, 2H), 2.87 (s, 3H), 2.02 – 1.88 (m, 2H), 1.77 (p, J = 6.8 Hz, 2H), 1.63 – 1.52 (m, 2H), 1.43 (t, J = 19.2 Hz, 2H). ^{11}B NMR (128 MHz, Acetonitrile-d_3) δ 11.21. ^{19}F NMR (376 MHz, Acetonitrile-d_3) δ -85.64. ^{13}C NMR (126 MHz, Acetonitrile-d_3) δ 168.4, 163.1, 134.7, 129.8 – 125.3 (m), 119.7, 115.9, 103.9, 68.7, 62.4, 46.6, 38.2 (t, J = 26.0 Hz), 28.9, 19.7 (t, J = 5.0 Hz). **ESI-MS:** calcd for $C_{18}H_{21}N_2O_5BF_2Na$ [M + Na]$^+$: 417.1405, found: 417.1404.

4,4-difluoro-5-(6-methyl-4,8-dioxo-1,3,6,2-dioxazaborocan-2-yl)pentyl benzoate (14)

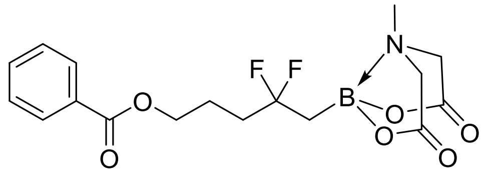

Following the general procedure **A**, the product **14** was obtained in 53% yield as a white solid after column chromatography (eluent = petroleum ether/ ethyl acetate 1:3 v/v). R_F = 0.35 (PE: ethyl acetate = 1:4). ^{1}H NMR (400 MHz, Acetone-d_6) δ 8.11 – 7.96 (m, 2H), 7.66 – 7.59 (m, 1H), 7.51 (t, J = 7.7 Hz, 2H), 4.36 (t, J = 6.4 Hz, 2H), 4.26 (d, J = 17.0 Hz, 2H), 4.05 (d, J = 16.9 Hz, 2H), 3.18 (s, 3H), 2.26 – 2.10 (m, 2H), 2.02 – 1.93 (m, 2H), 1.59 (t, J = 19.0 Hz, 2H). ^{11}B NMR (128 MHz, Acetone-d_6) δ 11.16. ^{19}F NMR (376 MHz, Acetone-d_6) δ -85.32. ^{13}C NMR (101 MHz, Acetone-d_6) δ 167.6, 165.8, 132.9, 130.5, 129.4, 128.5, 126.9 (t, J = 238.7 Hz), 64.1, 61.9, 45.9, 34.6 (t, J = 26.4 Hz), 22.2 (t, J = 4.9 Hz). **ESI-MS:** calcd for $C_{17}H_{20}NO_6BF_2Na$ [M + Na]$^+$: 406.1245, found: 406.1245.

4,4-difluoro-5-(6-methyl-4,8-dioxo-1,3,6,2-dioxazaborocan-2-yl)pentyl 3-nitrobenzoate (15)

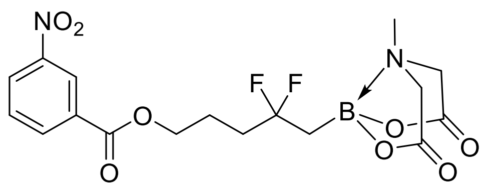

Following the general procedure **A**, the product **15** was obtained in 28% yield as a white solid after column chromatography (eluent = petroleum ether/ ethyl acetate 1:3 v/v). R_F = 0.26 (PE: ethyl acetate = 1:4). ^{1}H NMR (500 MHz, DMSO-d_6) δ 8.65 (t, J = 2.0 Hz, 1H), 8.50 (ddd, J = 8.2, 2.4, 1.1 Hz, 1H), 8.41 (dt, J = 7.8, 1.4 Hz, 1H), 7.84 (t, J = 8.0 Hz, 1H), 4.38 (t, J = 6.4 Hz, 2H), 4.22 (d, J = 17.0 Hz, 2H), 3.98 (d, J = 17.0 Hz, 2H), 2.88 (s, 3H), 2.17 – 2.00 (m, 2H), 1.95 – 1.86 (m, 2H), 1.48 (t, J = 19.1 Hz, 2H). ^{11}B NMR (160 MHz, Acetone-d_6) δ 11.18. ^{19}F NMR (471 MHz, Acetone-d_6) δ -85.10. ^{13}C NMR (126 MHz, Acetone-d_6) δ 167.6, 164.1, 148.5, 135.3, 132.1, 130.3, 127.4, 126.8 (t, J = 238.7 Hz), 123.9, 65.0, 61.9, 45.9, 34.5 (t, J = 26.5 Hz), 22.1 (t, J = 4.8 Hz). **ESI-MS:** calcd for $C_{17}H_{19}N_2O_8BF_2Na$ [M + Na]$^+$: 451.1096, found: 451.1098.

7,7-difluoro-8-(6-methyl-4,8-dioxo-1,3,6,2-dioxazaborocan-2-yl)octyl 3-nitrobenzoate (16)

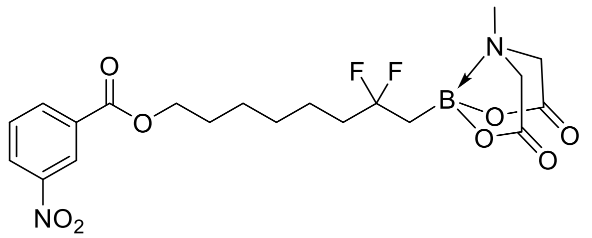

Following the general procedure **B**, the product **16** was obtained in 52% yield as a white solid after column chromatography (eluent = petroleum ether/ ethyl acetate 1:3 v/v). R_F = 0.30 (PE: ethyl acetate = 1:4). ^{1}H NMR (500 MHz, DMSO-d_6) δ 8.62 (t, *J* = 2.0 Hz, 1H), 8.50 (dd, *J* = 8.2, 2.4 Hz, 1H), 8.41 – 8.34 (m, 1H), 7.84 (t, *J* = 8.0 Hz, 1H), 4.34 (t, *J* = 6.6 Hz, 2H), 4.21 (d, *J* = 17.0 Hz, 2H), 3.97 (d, *J* = 17.1 Hz, 2H), 2.86 (s, 3H), 1.94 – 1.82 (m, 2H), 1.76 (p, *J* = 6.7 Hz, 2H), 1.51 – 1.32 (m, 8H). ^{11}B NMR (128 MHz, Acetone-d_6) δ 11.18. ^{19}F NMR (376 MHz, Acetone-d_6) δ -84.76. ^{13}C NMR (101 MHz, Acetonitrile-d_3) δ 168.4, 165.0, 149.0, 135.7, 132.8, 130.8, 127.9, 127.9 (t, *J* = 238.1 Hz), 124.5, 66.3, 62.4, 46.6, 38.5 (t, *J* = 26.0 Hz), 29.2, 28.7, 26.0, 22.9 (t, *J* = 4.7 Hz). **ESI-MS:** calcd for $C_{20}H_{25}N_2O_8BF_2Na$ [M + Na]$^+$: 493.1565, found: 493.1565.

2-(6-(1,3-dioxoisoindolin-2-yl)-2,2-difluorohexyl)-6-methyl-1,3,6,2-dioxazaborocane-4,8-dione (17)

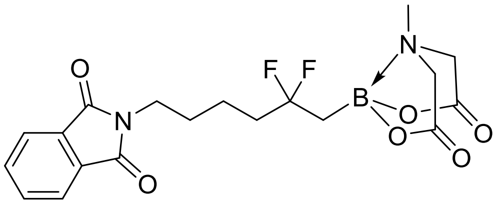

Following the general procedure **A**, the product **17** was obtained in 55% yield as a white solid after column chromatography (eluent = petroleum ether/ ethyl acetate 1:3 v/v). R_F = 0.20 (PE: ethyl acetate = 1:4). ^{1}H NMR (400 MHz, Acetone-d_6) δ 8.06 – 7.73 (m, 4H), 4.23 (d, *J* = 16.9 Hz, 2H), 4.02 (d, *J* = 16.9 Hz, 2H), 3.68 (t, *J* = 7.1 Hz, 2H), 3.15 (s, 3H), 2.04 – 1.93 (m, 2H), 1.73 (p, *J* = 7.3 Hz, 2H), 1.61 – 1.44 (m, 4H). ^{11}B NMR (128 MHz, Acetone-d_6) δ 11.18. ^{19}F NMR (376 MHz, Acetone-d_6) δ -85.28. ^{13}C NMR (101 MHz, Acetone-d_6) δ 167.9, 167.6, 134.1, 132.3, 126.9 (t, *J* = 238.5 Hz), 122.8, 61.9, 45.9, 37.5 (t, *J* = 26.2 Hz), 37.4, 28.2, 20.0 (t, *J* = 4.8 Hz). **ESI-MS:** calcd for $C_{19}H_{21}N_2O_6BF_2Na$ [M + Na]$^+$: 445.1354, found: 445.1352.

4-(((7,7-difluoro-8-(6-methyl-4,8-dioxo-1,3,6,2-dioxazaborocan-2-yl)octyl)oxy)methyl)benzonitrile (18)

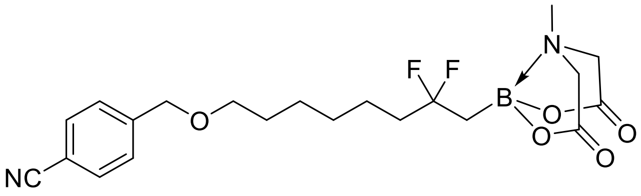

Following the general procedure **A**, the product **18** was obtained in 66% yield as a white solid after column chromatography (eluent = petroleum ether/ ethyl acetate 1:3 v/v). R_F = 0.21 (PE: ethyl acetate = 1:4). ^{1}H NMR (400 MHz, Acetone-d_6) δ 7.75 (d, *J* = 8.3 Hz, 2H), 7.56 (d, *J* = 7.8 Hz, 2H), 4.60 (s, 2H), 4.24 (d, *J* = 16.9 Hz, 2H), 4.03 (d, *J* = 16.9 Hz, 2H), 3.53 (t, *J* = 6.4 Hz, 2H), 3.16 (s, 3H), 2.01 – 1.86 (m, 2H), 1.70 – 1.58 (m, 2H), 1.59 – 1.33 (m, 8H). ^{11}B NMR (128 MHz, Acetone-d_6) δ 11.35. ^{19}F NMR (376 MHz, Acetone-d_6) δ -84.75. ^{13}C NMR (126 MHz, Acetonitrile-d_3) δ 168.4, 145.6, 132.8, 128.4, 127.9 (t, *J* = 238.1 Hz), 119.4, 111.3, 71.9, 71.0, 62.4, 46.6, 38.5 (t, *J* = 25.9 Hz), 29.8, 29.4, 26.3, 23.0 (t, *J* = 4.7 Hz). **ESI-MS:** calcd for $C_{21}H_{27}N_2O_5BF_2Na$ [M + Na]$^+$: 459.1874, found: 459.1873.

7,7-difluoro-8-(6-methyl-4,8-dioxo-1,3,6,2-dioxazaborocan-2-yl)octyl 4-methylbenzenesulfonate (19)

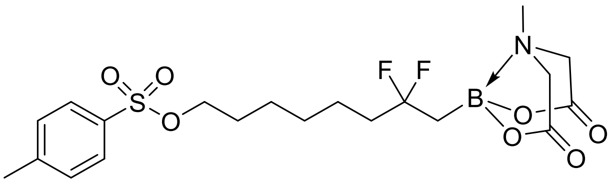

Following the general procedure **A**, the product **19** was obtained in 50% yield as a white solid after column chromatography (eluent = petroleum ether/ ethyl acetate 1:3 v/v). R_F = 0.34 (PE: ethyl acetate = 1:4). ^{1}H NMR (500 MHz, Acetone-d_6) δ 7.81 (d, *J* = 8.3 Hz, 2H), 7.49

(d, J = 8.0 Hz, 2H), 4.24 (d, J = 16.9 Hz, 2H), 4.08 – 4.00 (m, 4H), 3.15 (s, 3H), 2.46 (s, 3H), 1.97 – 1.82 (m, 2H), 1.69 – 1.59 (m, 2H), 1.51 (t, J = 19.0 Hz, 2H), 1.46 – 1.38 (m, 2H), 1.36 – 1.23 (m, 4H). ^{1}B NMR (160 MHz, Acetone-d_6) δ 11.20. ^{19}F NMR (471 MHz, Acetone-d_6) δ -84.79. ^{13}C NMR (101 MHz, Acetone-d_6) δ 168.4, 145.9, 133.6, 130.6, 128.3, 127.8 (t, J = 238.1 Hz), 71.6, 62.4, 46.6, 38.4 (t, J = 26.0 Hz), 28.8, 25.4, 22.8 (t, J = 4.8 Hz), 21.2. **ESI-MS:** calcd for $C_{20}H_{28}NO_7BF_2Na$ [M + Na]$^+$: 498.1541, found: 498.1544.

6-methyl-2-(2-oxohexyl)-1,3,6,2-dioxazaborocane-4,8-dione (20)

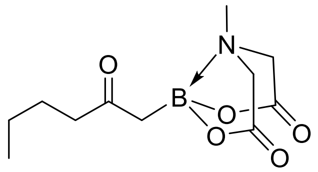

Following the general procedure **B**, the product **20** was obtained in 98% yield as a semi solid after column chromatography (eluent = petroleum ether/ ethyl acetate 1:3 v/v). R_F = 0.46 (ethyl acetate). ^{1}H NMR (400 MHz, Acetonitrile-d_3) δ 3.99 (d, J = 17.0 Hz, 2H), 3.89 (d, J = 17.0 Hz, 2H), 2.98 (s, 3H), 2.49 (t, J = 7.3 Hz, 2H), 2.20 (s, 2H), 1.55 – 1.43 (m, 2H), 1.37 – 1.23 (m, 2H), 0.91 (t, J = 7.3 Hz, 3H). ^{13}C NMR (126 MHz, CD_3CN) δ 213.9, 168.9, 63.2, 63.1, 47.3, 45.0, 26.5, 22.8, 14.1. ^{11}B NMR (128 MHz, Acetone-d_6) δ 11.4. **ESI-MS:** calcd for $C_{11}H_{18}BNO_5Na$ [M + Na]$^+$: 278.1170, found: 278.1170.

6-methyl-2-(2-oxoheptyl)-1,3,6,2-dioxazaborocane-4,8-dione (21)

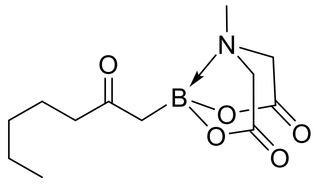

Following the general procedure **B**, the product **21** was obtained in 80% yield as a semi solid after column chromatography (eluent = petroleum ether/ ethyl acetate 1:3 v/v). R_F = 0.46 (ethyl acetate). ^{1}H NMR (500 MHz, Acetone-d_6) δ 4.22 (d, J = 16.9 Hz, 2H), 4.04 (d, J = 16.9 Hz, 2H), 3.20 (d, J = 1.8 Hz, 3H), 2.48 (t, J = 7.3 Hz, 2H), 2.21 (s, 2H), 1.50 (p, J = 7.3 Hz, 2H), 1.35 – 1.21 (m, 5H), 0.87 (t, J = 7.1 Hz, 3H). ^{13}C NMR (101 MHz, $CDCl_3$) δ 214.8, 168.0, 62.6, 46.7, 45.6, 31.3, 23.4, 22.6, 14.0. ^{11}B NMR (128 MHz, $CDCl_3$) δ 12.2. **ESI-MS:** calcd for $C_{12}H_{20}BNO_5Na$ [M + Na]$^+$: 292.1327, found: 292.1323.

6-methyl-2-(2-oxononyl)-1,3,6,2-dioxazaborocane-4,8-dione (22)

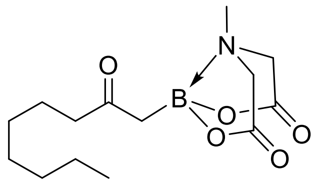

Following the general procedure **B**, the product **22** was obtained in 75% yield as a semi solid after column chromatography (eluent = petroleum ether/ ethyl acetate 1:3 v/v). R_F = 0.50 (ethyl acetate). ^{1}H NMR (400 MHz, Chloroform-d) δ 4.00 (d, J = 16.7 Hz, 2H), 3.93 (d, J = 16.6 Hz, 2H), 3.09 (s, 3H), 2.49 (t, J = 7.4 Hz, 2H), 2.23 (s, 2H), 1.50 (p, J = 7.1 Hz, 2H), 1.28 – 1.23 (m, 8H), 0.87 (t, J = 6.8 Hz, 3H). ^{13}C NMR (101 MHz, Chloroform-d) δ 214.8, 168.0, 62.6, 46.7, 45.6, 31.8, 29.2, 29.2, 23.8, 22.7, 14.2. ^{11}B NMR (128 MHz, DMSO-d_6) δ 7.3. **ESI-MS:** calcd for $C_{14}H_{24}BNO_5Na$ [M + Na]$^+$: 320.1640, found: 320.1640.

2-(5,5-dimethyl-2-oxohexyl)-6-methyl-1,3,6,2-dioxazaborocane-4,8-dione (23)

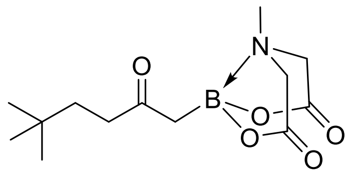

Following the general procedure **B** (5.0 equiv AcCl, 2.5 equiv H_2O), the product **23** was obtained in 83% yield as a semi solid after column chromatography (eluent = petroleum ether/ ethyl acetate 1:3 v/v). R_F = 0.63 (ethyl acetate). ^{1}H NMR (400 MHz, Acetone-d_6) δ 4.22 (d, J = 16.8 Hz, 2H), 4.04 (d, J = 16.8 Hz, 2H), 3.20 (s, 3H), 2.55 – 2.41 (m, 2H), 2.25 (s, 2H), 1.49 – 1.39 (m, 2H), 0.88

(s, 9H). ^{13}C NMR (101 MHz, Acetone-*d*6) δ 213.5, 168.6, 63.1, 47.2, 41.0, 38.0, 30.4, 29.5. ^{11}B NMR (128 MHz, Acetone-*d*6) δ 11.4. **ESI-MS:** calcd for $C_{13}H_{22}BNO_5Na$ [M + Na]$^+$: 306.1483, found: 306.1480.

2-(3-cyclobutyl-2-oxopropyl)-6-methyl-1,3,6,2-dioxazaborocane-4,8-dione (24)

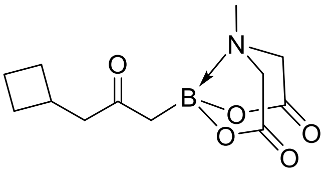

Following the general procedure **B**, the product **24** was obtained in 22% yield as a semi solid after column chromatography (eluent = petroleum ether/ ethyl acetate 1:3 v/v). R_F = 0.49 (ethyl acetate). ^{1}H NMR (500 MHz, Chloroform-*d*) δ 3.91 (d, *J* = 16.4 Hz, 2H), 3.86 (d, *J* = 16.4 Hz, 2H), 3.10 (s, 3H), 2.62 (d, *J* = 3.0 Hz, 2H), 2.21 (s, 2H), 2.14 – 2.05 (m, 2H), 1.94 – 1.76 (m, 2H), 1.66 – 1.56 (m, 2H). ^{13}C NMR (101 MHz, Acetone-*d*6) δ 212.5, 168.5, 63.0, 52.5, 47.1, 32.3, 29.0, 19.3. ^{11}B NMR (128 MHz, Acetone-*d*6) δ 11.4. **ESI-MS:** calcd for $C_{12}H_{18}BNO_5Na$ [M + Na]$^+$: 290.1170, found: 290.1172.

2-(3-cyclopentyl-2-oxopropyl)-6-methyl-1,3,6,2-dioxazaborocane-4,8-dione (25)

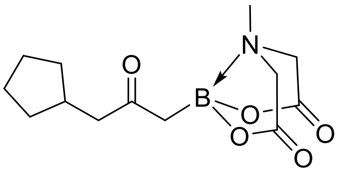

Following the general procedure **B**, the product **25** was obtained in 53% yield as a semi solid after column chromatography (eluent = petroleum ether/ ethyl acetate 1:3 v/v). R_F = 0.50 (ethyl acetate). ^{1}H NMR (400 MHz, Acetone-*d*6) δ 4.21 (d, *J* = 16.8 Hz, 2H), 4.04 (d, *J* = 16.8 Hz, 2H), 3.21 (s, 3H), 2.51 (d, *J* = 7.1 Hz, 2H), 2.30 – 2.11 (m, 1H), 1.83 – 1.73 (m, 2H), 1.63 – 1.48 (m, 4H), 1.32-1.29 (m, 2H). ^{13}C NMR (126 MHz, $CDCl_3$) δ 215.1, 167.0, 62.7, 52.2, 46.7, 35.4, 32.6, 25.1. **ESI-MS:** calcd for $C_{13}H_{20}BNO_5Na$ [M + Na]$^+$: 304.1327, found: 304.1330.

2-(3-cyclohexyl-2-oxopropyl)-6-methyl-1,3,6,2-dioxazaborocane-4,8-dione (26)

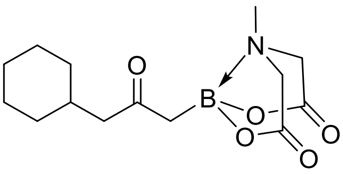

Following the general procedure **B**, the product **26** was obtained in 58% yield as a semi solid after column chromatography (eluent = petroleum ether/ ethyl acetate 1:3 v/v). R_F = 0.40 (Petroleum ether/ethyl acetate=1:4). ^{1}H NMR (400 MHz, Acetone-*d*6) δ 4.21 (d, *J* = 16.9 Hz, 2H), 4.04 (d, *J* = 16.9 Hz, 2H), 3.20 (s, 3H), 2.36 (d, *J* = 6.7 Hz, 2H), 2.20 (s, 2H), 1.85 – 1.70 (m, 1H), 1.69 – 1.60 (m, 5H), 1.26 – 1.10 (m, 3H), 0.96 – 0.87 (m, 2H). ^{13}C NMR (101 MHz, Acetone-*d*6) δ 211.8, 167.6, 62.2, 52.2, 46.2, 33.4, 32.9, 26.1, 26.0. ^{11}B NMR (128 MHz, Acetone-*d*6) δ 11.5. **ESI-MS:** calcd for $C_{14}H_{22}BNO_5Na$ [M + Na]$^+$: 318.1483, found: 318.1483.

2-(4-cyclohexyl-2-oxobutyl)-6-methyl-1,3,6,2-dioxazaborocane-4,8-dione (27)

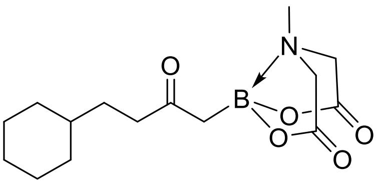

Following the general procedure **B**, the product **27** was obtained in 60% yield as a semi solid after column chromatography (eluent = petroleum ether/ ethyl acetate 1:3 v/v). R_F = 0.50 (ethyl acetate). ^{1}H NMR (400 MHz, Acetone-*d*6) δ 4.21 (d, *J* = 16.8 Hz, 2H), 4.03 (d, *J* = 16.8 Hz, 2H), 3.20 (s, 3H), 2.58 – 2.26 (m, 2H), 2.22 (s, 2H), 1.77 – 1.54 (m, 5H), 1.47 – 1.33 (m, 2H), 1.26 – 1.13 (m, 4H), 0.95 – 0.76 (m, 2H). ^{13}C NMR (126 MHz, $CDCl_3$) δ 215.1, 167.9, 62.7, 46.7, 43.2, 37.2, 33.2, 31.2, 26.6, 26.3. ^{11}B NMR (128 MHz, $CDCl_3$) δ 12.2. **ESI-MS:** calcd for $C_{15}H_{24}BNO_5Na$ [M + Na]$^+$: 332.1640, found: 332.1636.

6-methyl-2-(2-oxo-4-phenylbutyl)-1,3,6,2-dioxazaborocane-4,8-dione (28)

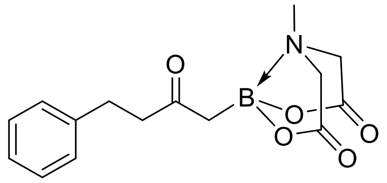

Following the general procedure **B**, the product **28** was obtained in 31% yield as a semi solid after column chromatography (eluent = petroleum ether/ ethyl acetate 1:4 v/v). R_F = 0.37(Petroleum ether/ethyl acetate=1:4). ^{1}H NMR (400 MHz, Chloroform-*d*) δ 7.25 – 7.21 (m, 4H), 7.17 – 7.13 (m, 1H), 4.23 (d, *J* = 16.9 Hz, 2H), 4.04 (d, *J* = 16.9 Hz, 2H), 3.16 (s, 3H), 2.82 – 2.81 (m, 4H), 2.25 (s, 2H). ^{13}C NMR (101 MHz, $CDCl_3$) δ 213.4, 167.6, 141.0, 128.6, 128.5, 126.2, 62.6, 46.7, 46.7, 29.7. ^{11}B NMR (128 MHz, $CDCl_3$) δ 11.7. **ESI-MS:** calcd for $C_{15}H_{18}BNO_5Na$ [M + Na]$^+$: 326.1170, found: 326.1173.

6-methyl-2-(2-oxo-4-phenylbutyl)-1,3,6,2-dioxazaborocane-4,8-dione (29)

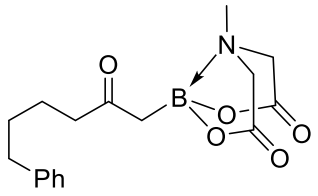

Following the general procedure **B**, the product **29** was obtained in 76% yield as a semi solid after column chromatography (eluent = petroleum ether/ ethyl acetate 1:3 v/v). R_F = 0.57 (ethyl acetate). ^{1}H NMR (400 MHz, Acetone-d_6) δ 7.32 – 7.07 (m, 5H), 4.22 (d, *J* = 16.9 Hz, 2H), 4.04 (d, *J* = 16.9 Hz, 2H), 3.19 (s, 3H), 2.61 (t, *J* = 7.2 Hz, 2H), 2.53 (t, *J* = 6.9 Hz, 2H), 2.22 (s, 2H), 1.62-1.51 (m, 4H). ^{13}C NMR (101 MHz, $CDCl_3$) δ 214.2, 168.1, 142.3, 128.5, 128.4, 125.8, 62.5, 46.7, 45.2, 35.8, 30.9, 23.8, 23.4. ^{11}B NMR (128 MHz, $CDCl_3$) δ 12.6. **ESI-MS:** calcd for $C_{17}H_{22}BNO_5Na$ [M + Na]$^+$: 354.1483, found: 354.1481.

2-(7-chloro-2-oxoheptyl)-6-methyl-1,3,6,2-dioxazaborocane-4,8-dione (30)

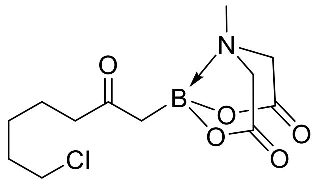

Following the general procedure **B**, the product **30** was obtained in 86% yield as a semi solid after column chromatography (eluent = petroleum ether/ ethyl acetate 1:3 v/v). R_F = 0.50 (ethyl acetate). ^{1}H NMR (500 MHz, Chloroform-*d*) δ 4.00 (d, *J* = 16.8 Hz, 2H), 3.89 (d, *J* = 16.7 Hz, 2H), 3.50 (t, *J* = 6.6 Hz, 2H), 3.05 (s, 3H), 2.49 (t, *J* = 7.2 Hz, 2H), 2.20 (s, 2H), 1.80 – 1.67 (m, 2H), 1.58 – 1.46 (m, 2H), 1.42 – 1.32 (m, 2H). ^{13}C NMR (126 MHz, $CDCl_3$) δ 214.0, 168.2, 62.5, 46.8, 45.1, 45.0, 32.4, 29.7, 26.3, 22.9. ^{11}B NMR (128 MHz, $CDCl_3$) δ 11.9. **ESI-MS:** calcd for $C_{12}H_{19}BClNO_5Na$ [M + Na]$^+$: 326.0937, found: 326.0937.

2-(8-chloro-2-oxooctyl)-6-methyl-1,3,6,2-dioxazaborocane-4,8-dione (31)

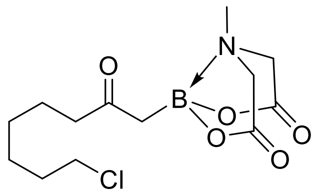

Following the general procedure **B** (5.0 equiv AcCl, 2.5 equiv H_2O), the product **31** was obtained in 56% yield as a semi solid after column chromatography (eluent = petroleum ether/ ethyl acetate 1:3 v/v). R_F = 0.52 (ethyl acetate). ^{1}H NMR (400 MHz, Chloroform-*d*) δ 4.01 – 3.86 (m, 4H), 3.53 (t, *J* = 6.0 Hz, 2H), 3.11 (s, 3H), 2.51 (t, *J* = 6.6 Hz, 2H), 2.25 (s, 2H), 1.83 – 1.71 (m, 2H), 1.57 – 1.49 (m, 2H), 1.48 – 1.38 (m, 2H), 1.34 – 1.27 (m, 2H). ^{13}C NMR (101 MHz, $CDCl_3$) δ 214.5, 167.6, 62.7, 46.8, 45.4, 45.2, 32.5, 28.4, 26.7, 23.5. ^{11}B NMR (128 MHz, $CDCl_3$) δ 11.8. **ESI-MS:** calcd for $C_{13}H_{21}BClNO_5Na$ [M + Na]$^+$: 340.1093, found: 340.1098.

8-(6-methyl-4,8-dioxo-1,3,6,2-dioxazaborocan-2-yl)-7-oxooctyl formate (32)

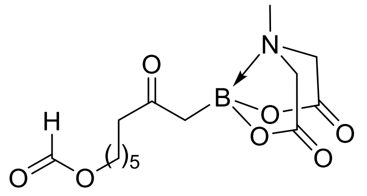

Following the general procedure **B** (5.0 equiv AcCl, 2.5 equiv H_2O), the product **32** was obtained in 81% yield as a semi solid after column chromatography (eluent = petroleum ether/ ethyl acetate 1:4 v/v). R_F = 0.38 (ethyl acetate). ^1H NMR (400 MHz, Chloroform-*d*) δ 8.03 (s, 1H), 4.12 (t, *J* = 6.4 Hz, 2H), 3.96 (d, *J* = 16.8 Hz, 2H), 3.89 (d, *J* = 16.6 Hz, 2H), 3.08 (s, 3H), 2.48 (t, *J* = 7.0 Hz, 2H), 2.21 (s, 2H), 1.69 – 1.57 (m, 2H), 1.54 – 1.45 (m, 2H), 1.39 – 1.20 (m, 4H). ^{13}C NMR (101 MHz, $CDCl_3$) δ 214.5, 167.8, 161.4, 64.0, 62.6, 46.7, 45.3, 28.6, 28.3, 25.7, 23.4. ^{11}B NMR (128 MHz, $CDCl_3$) δ 12.0. **ESI-MS:** calcd for $C_{14}H_{22}BNO_7Na$ $[M + Na]^+$: 350.1381, found: 350.1381.

methyl 7-(6-methyl-4,8-dioxo-1,3,6,2-dioxazaborocan-2-yl)-6-oxoheptanoate (33)

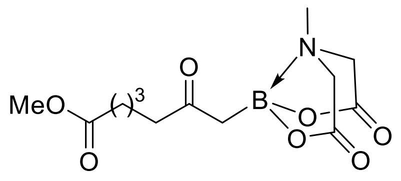

Following the general procedure **B** (5.0 equiv AcCl, 2.5 equiv H_2O), the product **33** was obtained in 46% yield as a semi solid after column chromatography (eluent = petroleum ether/ ethyl acetate 1:4 v/v). R_F = 0.50 (ethyl acetate). ^1H NMR (400 MHz, Chloroform-*d*) δ 3.92 (s, 4H), 3.65 (s, 3H), 3.10 (s, 3H), 2.52 (t, *J* = 6.6 Hz, 2H), 2.30 (t, *J* = 6.8 Hz, 2H), 2.24 (s, 2H), 1.64 – 1.49 (m, 4H). ^{13}C NMR (101 MHz, $CDCl_3$) δ 214.2, 174.1, 167.5, 62.7, 51.7, 46.8, 45.1, 33.9, 24.3, 23.1. ^{11}B NMR (128 MHz, $CDCl_3$) δ 11.7. **ESI-MS:** calcd for $C_{13}H_{20}BNO_7$ Na $[M + Na]^+$: 336.1225, found: 336.1224.

7-(6-methyl-4,8-dioxo-1,3,6,2-dioxazaborocan-2-yl)-6-oxoheptyl benzoate (34)

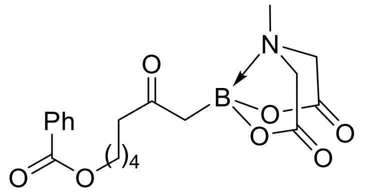

Following the general procedure **B** (5.0 equiv AcCl, 2.5 equiv H_2O), the product **34** was obtained in 66% yield as a semi solid after column chromatography (eluent = petroleum ether/ ethyl acetate 1:4 v/v). R_F = 0.41 (ethyl acetate). ^1H NMR (400 MHz, Chloroform-*d*) δ 8.01 (d, *J* = 7.7 Hz, 2H), 7.54 (t, *J* = 7.4 Hz, 1H), 7.42 (t, *J* = 7.6 Hz, 2H), 4.28 (t, *J* = 6.5 Hz, 2H), 3.97 – 3.91 (m, 4H), 3.07 (s, 3H), 2.52 (t, *J* = 7.1 Hz, 2H), 2.22 (s, 2H), 1.75 (p, *J* = 6.8 Hz, 2H), 1.62 – 1.51 (m, 2H), 1.44 – 1.35 (m, 2H). ^{13}C NMR (101 MHz, $CDCl_3$) δ 214.3, 167.8, 166.8, 133.0, 130.5, 129.6, 128.5, 64.9, 62.6, 46.7, 45.3, 28.6, 25.6, 23.3. ^{11}B NMR (128 MHz, $CDCl_3$) δ 11.8. **ESI-MS:** calcd for $C_{19}H_{24}BNO_7Na$ $[M + Na]^+$: 412.1538, found: 412.1538.

***N*-(7-(6-methyl-4,8-dioxo-1,3,6,2-dioxazaborocan-2-yl)-6-oxoheptyl)-4-nitrobenzenesulfonamide (35)**

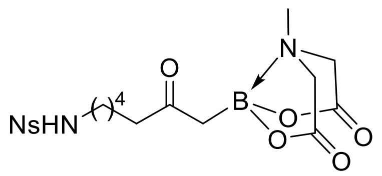

Following the general procedure **B** (5.0 equiv AcCl, 2.5 equiv H_2O, 60 °C), the product **35** was obtained in 94% yield as a white solid after column chromatography (eluent = petroleum ether/ ethyl acetate 1:4 v/v). R_F = 0.35 (ethyl acetate). ^1H NMR (400 MHz, Acetone-d_6) δ 8.43 (d, *J* = 8.3 Hz, 2H), 8.13 (d, *J* = 8.1 Hz, 2H), 6.85 (s, 1H), 4.23 (d, *J* = 16.9 Hz, 2H), 4.05 (d, *J* = 17.0 Hz, 2H), 3.19 (s, 3H), 3.01 – 2.93 (m, 2H), 2.44 (t, *J* = 7.0 Hz, 2H), 2.21 (s, 2H), 1.46 (dt, *J* = 14.7, 7.3 Hz, 4H), 1.26 – 1.19 (m, 2H). ^{13}C NMR (101 MHz, Acetone-d_6) δ 212.8, 168.3, 150.5, 147.3, 128.8, 124.8, 62.8, 62.7, 62.7, 46.8, 44.5, 43.4, 43.3, 26.3, 23.3. ^{11}B NMR (128 MHz, Acetone-d_6) δ 11.4. **ESI-MS:** calcd for $C_{18}H_{24}BN_3O_9S$ Na $[M + Na]^+$: 492.1218, found: 492.1217.

2-(7-(1,3-dioxoisoindolin-2-yl)-2-oxoheptyl)-6-methyl-1,3,6,2-dioxazaborocane-4,8-dione (36)

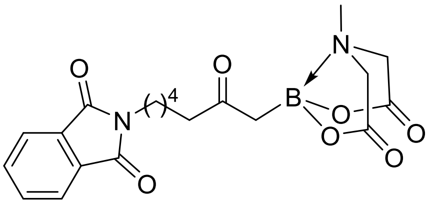

Following the general procedure **B** (5.0 equiv AcCl, 2.5 equiv H_2O, 60 °C), the product **36** was obtained in 98% yield as a semi solid after column chromatography (eluent = petroleum ether/ ethyl acetate 1:4 v/v). R_F = 0.35 (ethyl acetate). ^{1}H NMR (500 MHz, Acetone-*d*$_6$) δ 7.89 – 7.79 (m, 4H), 4.22 (d, *J* = 16.8 Hz, 1H), 4.04 (d, *J* = 16.8 Hz, 1H), 3.64 (t, *J* = 7.2 Hz, 2H), 3.19 (s, 2H), 2.50 (t, *J* = 7.2 Hz, 3H), 2.21 (s, 1H), 1.66 (p, *J* = 7.4 Hz, 2H), 1.55 (p, *J* = 7.4 Hz, 2H), 1.37 – 1.30 (m, 2H). ^{13}C NMR (101 MHz, Acetone-*d*$_6$) δ 212.9, 168.8, 168.5, 134.8, 133.1, 123.6, 63.0, 47.1, 45.0, 38.2, 29.0, 27.0, 23.9. ^{11}B NMR (128 MHz, Acetone-*d*$_6$) δ 11.4. **ESI-MS:** calcd for $C_{20}H_{23}BN_2O_7$ Na [M + Na]$^+$: 437.1490, found: 437.1491.

2-(7-(1,1-dioxido-3-oxobenzo[d]isothiazol-2(3H)-yl)-2-oxoheptyl)-6-methyl-1,3,6,2-dioxazaborocane-4,8-dione (37)

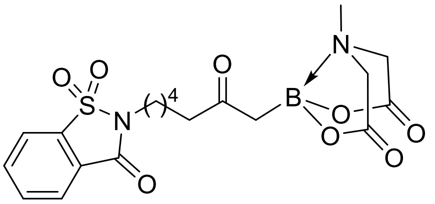

Following the general procedure **B** (5.0 equiv AcCl, 2.5 equiv H_2O, 60 °C), the product **37** was obtained in 94% yield as a semi solid after column chromatography (eluent = petroleum ether/ ethyl acetate 1:4 v/v). R_F = 0.38 (ethyl acetate). ^{1}H NMR (400 MHz, Chloroform-*d*) δ 8.13 – 7.68 (m, 4H), 3.91 (s, 4H), 3.74 (t, *J* = 7.6 Hz, 2H), 3.10 (s, 3H), 2.52 (t, *J* = 7.4 Hz, 2H), 2.23 (s, 2H), 1.82 (p, *J* = 7.7, 7.1 Hz, 2H), 1.57 (p, *J* = 7.5 Hz, 2H), 1.43 – 1.32 (m, 2H). ^{13}C NMR (101 MHz, $CDCl_3$) δ 214.3, 167.5, 159.1, 137.7, 134.8, 134.5, 127.5, 125.3, 121.0, 62.7, 46.8, 45.2, 39.3, 28.3, 26.2, 23.0. ^{11}B NMR (128 MHz, $CDCl_3$) δ 11.8. **ESI-MS:** calcd for $C_{19}H_{23}BN_2O_8S$ Na [M + Na]$^+$: 473.1160, found: 473.1155.

6-methyl-2-(2-oxo-7-(4,5,6,7-tetrachloro-1,3-dioxo-2,3-dihydro-1H-inden-2-yl)heptyl)-1,3,6,2-dioxazaborocane-4,8-dione (38)

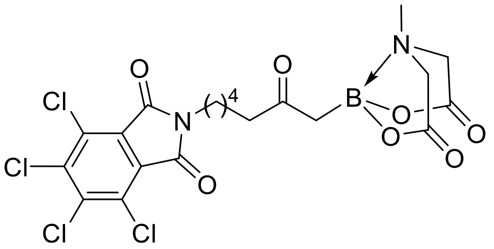

Following the general procedure **B** (5.0 equiv AcCl, 2.5 equiv H_2O, 60 °C), the product **38** was obtained in 67% yield as a yellow solid after column chromatography (eluent = petroleum ether/ ethyl acetate 1:3 v/v). R_F = 0.48 (ethyl acetate). ^{1}H NMR (400 MHz, DMSO-*d*$_6$) δ 4.23 (d, *J* = 17.0 Hz, 2H), 3.98 (d, *J* = 17.0 Hz, 2H), 3.54 (t, *J* = 6.8 Hz, 2H), 2.90 (s, 3H), 2.42 (t, *J* = 7.0 Hz, 2H), 2.11 (s, 2H), 1.63 – 1.50 (m, 2H), 1.45 (p, *J* = 7.1, 6.6 Hz, 2H), 1.28 – 1.24 (m, 2H). ^{13}C NMR (101 MHz, DMSO-*d*$_6$) δ 211.4, 168.6, 163.4, 138.0, 128.4, 128.0, 61.8, 46.2, 43.3, 38.1, 27.5, 25.7, 22.9. ^{11}B NMR (128 MHz, DMSO-*d*$_6$) δ 11.4. **ESI-MS:** calcd for $C_{21}H_{20}BCl_4NO_7Na$ [M + Na]$^+$: 571.9979, found: 571.9984.

2-(4-cyclohexyl-2-oxobutyl)-5,5,6,7,7-pentamethyl-1,3,6,2-dioxazaborocane-4,8-dione (39)

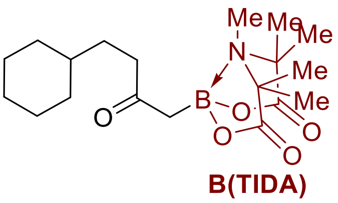

Following the general procedure **B**, the product **39** was obtained in 46% yield as a semi solid after column chromatography (eluent = petroleum ether/ ethyl acetate 1:3 v/v). R_F = 0.30 (ethyl acetate). ^{1}H NMR (400 MHz, Chloroform-*d*) δ 2.61 (s, 3H), 2.55 (t, *J* = 7.7 Hz, 2H), 2.24 (s, 2H), 1.72 (s, 6H), 1.70 – 1.64 (m, 4H), 1.60 (s, 6H), 1.43 (q, *J* = 7.3 Hz, 2H), 1.30 – 1.06 (m, 5H), 0.93 – 0.81 (m,

2H). ^{13}C NMR (126 MHz, $CDCl_3$) δ 212.9, 173.5, 42.2, 37.3, 36.7, 33.3, 31.5, 26.7, 26.4. ^{11}B NMR (128 MHz, $CDCl_3$) δ 9.4. **ESI-MS:** calcd for $C_{19}H_{32}BNO_5$ Na [M + Na]$^+$: 388.2266, found: 388.2261.

(E)-2-(2-fluoro-3-iodohept-2-en-1-yl)-6-methyl-1,3,6,2-dioxazaborocane-4,8-dione (40)

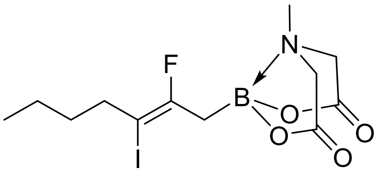

Following the general procedure **C**, the product **40** was obtained in 50% yield (rr = 11: 1) as a lightly yellow solid after column chromatography (eluent = petroleum ether/ ethyl acetate 1:3 v/v). R_F = 0.35 (PE: ethyl acetate = 1:4). ^{1}H NMR (400 MHz, Acetone-d_6) δ 4.27 (d, J = 17.0 Hz, 2H), 4.05 (d, J = 16.9 Hz, 2H), 3.20 (s, 3H), 2.45 (td, J = 7.2, 2.8 Hz, 2H), 2.26 (d, J = 25.2 Hz, 2H), 1.50 – 1.36 (m, 2H), 1.36 – 1.27 (m, 2H), 0.90 (t, J = 7.3 Hz, 3H). ^{11}B NMR (128 MHz, Acetone-d_6) δ 11.58. ^{19}F NMR (376 MHz, Acetone-d_6) δ -87.78. ^{13}C NMR (101 MHz, Acetone-d_6) δ 167.5, 157.9 (d, J = 254.1 Hz), 82.6 (d, J = 33.7 Hz), 62.0 (d, J = 1.4 Hz), 45.9 (d, J = 2.8 Hz), 34.5 (d, J = 7.2 Hz), 31.1 (d, J = 1.8 Hz), 21.1, 13.2. **ESI-MS:** calcd for $C_{12}H_{18}NO_4BFINa$ [M + Na]$^+$: 420.0251, found: 420.0250.

(E)-2-(4-cyclohexyl-2-fluoro-3-iodobut-2-en-1-yl)-6-methyl-1,3,6,2-dioxazaborocane-4,8-dione (41)

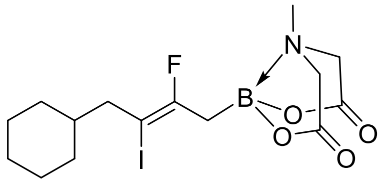

Following the general procedure **C**, the product **41** was obtained in 56% yield (rr = 5: 1) as a lightly yellow solid after column chromatography (eluent = petroleum ether/ ethyl acetate 1:3 v/v). R_F = 0.38 (PE: ethyl acetate = 1:4). ^{1}H NMR (500 MHz, Acetone-d_6) δ 4.26 (d, J = 16.9 Hz, 2H), 4.04 (d, J = 16.9 Hz, 2H), 3.20 (s, 3H), 2.33 (dd, J = 7.2, 2.9 Hz, 2H), 2.28 (d, J = 25.3 Hz, 2H), 1.75 – 1.60 (m, 6H), 1.58 – 1.45 (m, 1H), 1.34 – 1.12 (m, 4H). ^{11}B NMR (128 MHz, Acetone-d_6) δ 11.60. ^{19}F NMR (376 MHz, Acetone-d_6) δ -86.49. ^{13}C NMR (126 MHz, Acetone-d_6) δ 167.5, 158.4 (d, J = 254.1 Hz), 81.1 (d, J = 34.3 Hz), 62.0, 45.9 (d, J = 2.7 Hz), 42.2 (d, J = 6.1 Hz), 37.2 (d, J = 1.9 Hz), 32.1, 26.3, 26.0. **ESI-MS:** calcd for $C_{15}H_{22}NO_4BFINa$ [M + Na]$^+$: 460.0564, found: 460.0564.

2-(2-iodo-5-phenylpentyl)-6-methyl-1,3,6,2-dioxazaborocane-4,8-dione (42)

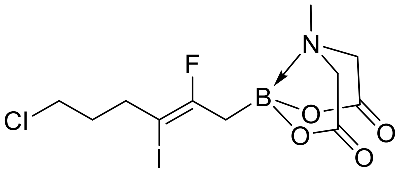

Following the general procedure **C**, the product **42** was obtained in 56% yield (rr = 10: 1) as a lightly yellow solid after column chromatography (eluent = petroleum ether/ ethyl acetate 1:3 v/v). R_F = 0.36 (PE: ethyl acetate = 1:4). ^{1}H NMR (500 MHz, Acetone-d_6) δ 4.28 (d, J = 16.9 Hz, 2H), 4.06 (d, J = 16.9 Hz, 2H), 3.60 (t, J = 6.6 Hz, 2H), 3.21 (s, 3H), 2.63 (td, J = 7.1, 2.7 Hz, 2H), 2.27 (d, J = 25.7 Hz, 2H), 1.93 (p, J = 6.8 Hz, 2H). ^{11}B NMR (128 MHz, Acetone-d_6) δ 11.49. ^{19}F NMR (376 MHz, Acetone-d_6) δ -85.99. ^{13}C NMR (126 MHz, Acetone-d_6) δ 167.5, 158.9 (d, J = 256.1 Hz), 80.1 (d, J = 33.2 Hz), 62.0, 46.0 (d, J = 2.7 Hz), 43.3, 32.3 (d, J = 7.7 Hz), 32.1 (d, J = 1.8 Hz). **ESI-MS:** calcd for $C_{11}H_{15}NO_4BFClINa$ [M + Na]$^+$: 439.9704, found: 436.9705.

(E)-2-(2-fluoro-3-iodo-4-phenylbut-2-en-1-yl)-6-methyl-1,3,6,2-dioxazaborocane-4,8-dione (43)

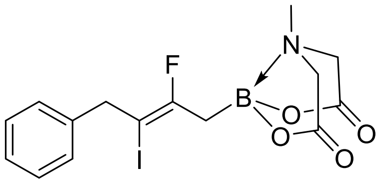

Following the general procedure **C**, the product **43** was obtained in 43% yield (rr > 20: 1) as a lightly yellow solid after column chromatography (eluent = petroleum ether/ ethyl acetate 1:3 v/v). R_F = 0.40 (PE: ethyl acetate = 1:4). ^{1}H NMR (400 MHz, Acetone-d_6) δ 7.33 – 7.27 (m, 1H), 7.26 – 7.20 (m, 2H), 4.30 (d, *J* = 16.9 Hz, 1H), 4.09 (d, *J* = 16.9 Hz, 1H), 3.86 (d, *J* = 2.7 Hz, 1H), 3.23 (s, 1H), 2.34 (d, *J* = 25.6 Hz, 1H). ^{11}B NMR (128 MHz, Acetone-d_6) δ 11.45. ^{11}B NMR (128 MHz, Acetone-d_6) δ 11.45. ^{13}C NMR (101 MHz, Acetone-d_6) δ 167.5, 158.8 (d, *J* = 256.1 Hz), 138.9 (d, *J* = 2.1 Hz), 128.6, 128.4, 126.4, 80.3 (d, *J* = 32.3 Hz), 62.1, 46.0 (d, *J* = 2.4 Hz), 41.0 (d, *J* = 7.8 Hz). **ESI-MS:** calcd for $C_{15}H_{16}NO_4BFINa$ $[M + Na]^+$: 454.0094, found: 454.0091.

(E)-6-fluoro-5-iodo-7-(6-methyl-4,8-dioxo-1,3,6,2-dioxazaborocan-2-yl)hept-5-enenitrile (44)

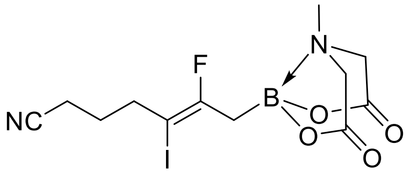

Following the general procedure **C** (1.7 equiv. DIH, 14.0 equiv. $Et_3N·HF$), the product **44** was obtained in 56% yield (rr > 20: 1) as a lightly yellow solid after column chromatography (eluent = petroleum ether/ ethyl acetate 1:3 v/v). R_F = 0.25 (PE: ethyl acetate = 1:4). ^{1}H NMR (500 MHz, Acetone-d_6) δ 4.29 (d, *J* = 16.9 Hz, 2H), 4.07 (d, *J* = 16.9 Hz, 2H), 3.21 (s, 3H), 2.61 (td, *J* = 7.1, 2.7 Hz, 2H), 2.45 (t, *J* = 7.2 Hz, 2H), 2.28 (d, *J* = 25.7 Hz, 2H), 1.81 (p, *J* = 7.2 Hz, 2H). ^{11}B NMR (128 MHz, Acetone-d_6) δ 11.50. ^{19}F NMR (376 MHz, Acetone-d_6) δ -85.44. ^{13}C NMR (126 MHz, Acetone-d_6) δ 167.5, 159.4 (d, *J* = 256.1 Hz), 119.4, 79.4 (d, *J* = 33.0 Hz), 62.0, 46.0 (d, *J* = 2.7 Hz), 33.6 (d, *J* = 7.6 Hz), 25.0 (d, *J* = 1.9 Hz), 14.8. **ESI-MS:** calcd for $C_{12}H_{15}N_2O_4BFINa$ $[M + Na]^+$: 431.0047, found: 431.0045.

(E)-2-(2-fluoro-3-iodo-6-phenylhex-2-en-1-yl)-6-methyl-1,3,6,2-dioxazaborocane-4,8-dione (45)

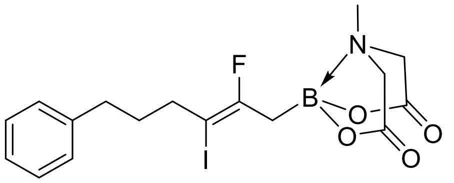

Following the general procedure **C**, the product **45** was obtained in 50% yield (rr = 11: 1) as a lightly yellow solid after column chromatography (eluent = petroleum ether/ ethyl acetate 1:3 v/v). R_F = 0.37 (PE: ethyl acetate = 1:4). ^{1}H NMR (500 MHz, Acetone-d_6) δ 7.30 – 7.21 (m, 4H), 7.18 – 7.13 (m, 1H), 4.27 (d, *J* = 16.9 Hz, 2H), 4.06 (d, *J* = 16.9 Hz, 2H), 3.20 (s, 3H), 2.61 (dd, *J* = 9.0, 6.7 Hz, 2H), 2.49 (td, *J* = 7.2, 2.7 Hz, 2H), 2.28 (d, *J* = 25.4 Hz, 2H), 1.78 (p, *J* = 7.4 Hz, 2H). ^{11}B NMR (128 MHz, Acetone-d_6) δ 11.43. ^{19}F NMR (376 MHz, Acetone-d_6) δ -86.87. ^{13}C NMR (126 MHz, Acetone-d_6) δ 167.5, 158.2 (d, *J* = 254.7 Hz), 142.2, 128.4, 128.2, 125.7, 82.3 (d, *J* = 33.6 Hz), 62.0, 46.0 (d, *J* = 2.4 Hz), 34.4 (d, *J* = 7.2 Hz), 34.1, 31.0 (d, *J* = 1.7 Hz). **ESI-MS:** calcd for $C_{17}H_{20}NO_4BFINa$ $[M + Na]^+$: 482.0407, found: 482.0405.

(E)-6-fluoro-5-iodo-7-(6-methyl-4,8-dioxo-1,3,6,2-dioxazaborocan-2-yl)hept-5-en-1-yl benzoate (46)

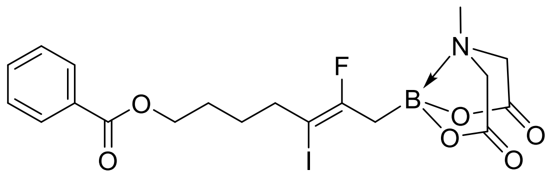

Following the general procedure **C**, the product **46** was obtained in 46% yield (rr = 9: 1) as a lightly yellow solid after column chromatography (eluent = petroleum ether/ ethyl acetate 1:3 v/v). R_F = 0.30 (PE: ethyl acetate = 1:4). ^{1}H NMR (500 MHz, Acetone-d_6) δ 8.10 – 8.01 (m, 2H), 7.66 – 7.59 (m, 1H), 7.51 (t, *J* = 7.8 Hz, 2H), 4.33 (t, *J* = 6.4 Hz, 2H), 4.28 (d, *J* = 16.9 Hz, 2H), 4.06 (d, *J* = 16.9 Hz, 2H), 3.20 (s, 3H), 2.55 (td, *J* = 7.1, 2.9 Hz, 2H), 2.28 (d, *J* = 25.3 Hz, 2H), 1.78 (dq, *J* = 8.9, 6.6 Hz, 2H), 1.70 – 1.57 (m, 2H). ^{11}B NMR (128 MHz, Acetone-d_6) δ 11.46. ^{19}F

NMR (376 MHz, Acetone-d_6) δ -87.07. ^{13}C NMR (126 MHz, Acetone-d_6) δ 167.5, 165.9, 158.4 (d, J = 254.5 Hz), 132.9, 130.6, 129.3, 128.5, 82.0 (d, J = 33.7 Hz), 64.3, 62.1, 46.0 (d, J = 2.6 Hz), 34.3 (d, J = 7.3 Hz), 27.1, 25.3 (d, J = 1.3 Hz). **ESI-MS:** calcd for $C_{19}H_{22}NO_4BFINa$ [M + Na]$^+$: 540.0462, found: 540.0460.

(E)-2-(2-chloro-3-iodohept-2-en-1-yl)-6-methyl-1,3,6,2-dioxazaborocane-4,8-dione (47)

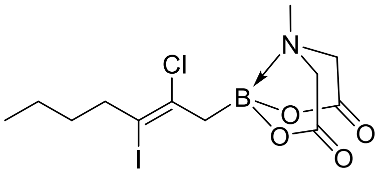

Following the general procedure **D**, the product **47** was obtained in 46% yield (rr = 4: 1) as a white solid after column chromatography (eluent = petroleum ether/ ethyl acetate 1:3 v/v). R_F = 0.40 (PE: ethyl acetate = 1:4). ^{1}H NMR (500 MHz, Acetone-d_6) δ 4.26 (d, J = 16.9 Hz, 2H), 4.08 (d, J = 16.9 Hz, 2H), 3.21 (s, 3H), 2.67 (t, 2H), 2.40 (s, 2H), 1.54 – 1.47 (m, 2H), 1.40 – 1.33 (m, 2H), 0.92 (t, J = 7.3 Hz, 3H). ^{11}B NMR (128 MHz, Acetone-d_6) δ 11.52. ^{13}C NMR (126 MHz, Acetone-d_6) δ 167.5, 129.7, 97.8, 62.0, 45.8, 41.4, 30.6, 21.2, 13.4. **ESI-MS:** calcd for $C_{12}H_{18}NO_4BClINa$ [M + Na]$^+$: 435.9955, found: 435.9952.

(E)-2-(2-chloro-3-iodo-4-phenylbut-2-en-1-yl)-6-methyl-1,3,6,2-dioxazaborocane-4,8-dione (48)

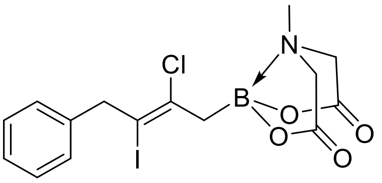

Following the general procedure **D**, the product **48** was obtained in 61% yield (rr = 9: 1) as a white solid after column chromatography (eluent = petroleum ether/ ethyl acetate 1:3 v/v). R_F = 0.37 (PE: ethyl acetate = 1:4). ^{1}H NMR (500 MHz, Acetone-d_6) δ 7.37 – 7.20 (m, 3H), 4.29 (d, J = 16.8 Hz, 1H), 4.15 – 4.01 (m, 2H), 3.24 (s, 1H), 2.51 (s, 1H). ^{11}B NMR (128 MHz, Acetone-d_6) δ 11.61. ^{13}C NMR (126 MHz, Acetone-d_6) δ 167.5, 138.1, 131.5, 128.5, 128.4, 126.5, 95.5, 62.0, 47.4, 45.9. **ESI-MS:** calcd for $C_{15}H_{16}NO_4BClINa$ [M + Na]$^+$: 469.9799, found: 469.9800.

(E)-6-chloro-5-iodo-7-(6-methyl-4,8-dioxo-1,3,6,2-dioxazaborocan-2-yl)hept-5-enenitrile (49)

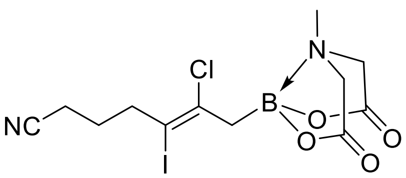

Following the general procedure **D**, the product **49** was obtained in 49% yield (rr = 8: 1) as a white solid after column chromatography (eluent = petroleum ether/ ethyl acetate 1:3 v/v). R_F = 0.26 (PE: ethyl acetate = 1:4). ^{1}H NMR (500 MHz, Acetonitrile-d_3) δ 4.00 (d, J = 17.0 Hz, 1H), 3.85 (d, J = 17.0 Hz, 1H), 2.94 (s, 1H), 2.80 (t, J = 7.2 Hz, 1H), 2.41 (t, J = 7.2 Hz, 1H), 2.35 (s, 1H), 1.85 (p, J = 7.2 Hz, 1H). ^{11}B NMR (128 MHz, Acetone-d_6) δ 11.47. ^{13}C NMR (126 MHz, Acetone-d_6) δ 167.5, 131.7, 119.5, 94.9, 62.0, 45.8, 40.4, 24.8, 14.8. **ESI-MS:** calcd for $C_{12}H_{15}N_2O_4BClINa$ [M + Na]$^+$: 446.9751, found: 446.9753.

(E)-2-(2-chloro-3-cyclohexyl-3-iodoallyl)-6-methyl-1,3,6,2-dioxazaborocane-4,8-dione (50)

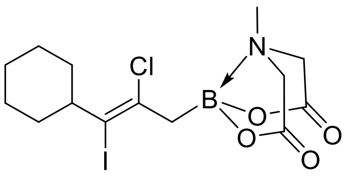

Following the general procedure **D**, the product **50** was obtained in 56% yield (rr > 20: 1) as a white solid after column chromatography (eluent = petroleum ether/ ethyl acetate 1:3 v/v). R_F = 0.34 (PE: ethyl acetate = 1:4). ^{1}H NMR (500 MHz, Acetone-d_6) δ 4.25 (d, J = 16.8 Hz, 2H), 4.08 (d, J = 16.9 Hz, 2H), 3.22 (s, 3H), 2.44 (s, 2H), 1.77 (dt, J = 12.9, 3.4 Hz, 2H), 1.72 – 1.63 (m, 1H), 1.57 – 1.48 (m, 2H), 1.48 – 1.25 (m, 6H). ^{11}B NMR (128 MHz, Acetone-d_6) δ 11.55. ^{13}C NMR (126 MHz, Acetone-d_6) δ 167.5, 127.4, 108.9, 62.0, 45.8, 44.7, 33.1, 25.5, 25.4. **ESI-MS:** calcd for $C_{14}H_{20}NO_4BClINa$ [M + Na]$^+$: 462.0112, found: 462.0112.

(E)-2-(2-chloro-3-iodo-6-phenylhex-2-en-1-yl)-6-methyl-1,3,6,2-dioxazaborocane-4,8-dione (51)

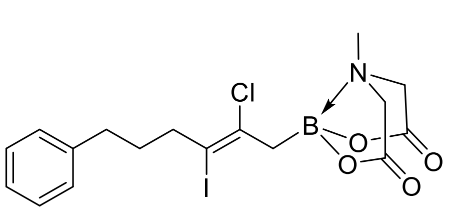

Following the general procedure **D**, the product **51** was obtained in 57% yield (rr = 4: 1) as a white solid after column chromatography (eluent = petroleum ether/ ethyl acetate 1:3 v/v). R_F = 0.36 (PE: ethyl acetate = 1:4). ^{1}H NMR (500 MHz, Acetone-d_6) δ 7.28 – 7.25 (m, 4H), 7.19 – 7.14 (m, 1H), 4.26 (d, *J* = 16.9 Hz, 2H), 4.09 (d, *J* = 16.8 Hz, 2H), 3.21 (s, 3H), 2.73 (t, *J* = 7.4 Hz, 2H), 2.69 – 2.63 (m, 2H), 2.43 (s, 2H), 1.89 – 1.82 (m, 2H). ^{11}B NMR (128 MHz, Acetone-d_6) δ 11.33. ^{13}C NMR (126 MHz, Acetone-d_6) δ 167.5, 142.2, 130.2, 128.4, 128.2, 125.7, 97.4, 62.0, 45.8, 41.3, 34.1, 30.6. **ESI-MS:** calcd for $C_{17}H_{20}NO_4BClINa$ [M + Na]$^+$: 498.0112, found: 498.0112.

(E)-6-chloro-5-iodo-7-(6-methyl-4,8-dioxo-1,3,6,2-dioxazaborocan-2-yl)hept-5-en-1-yl benzoate (52)

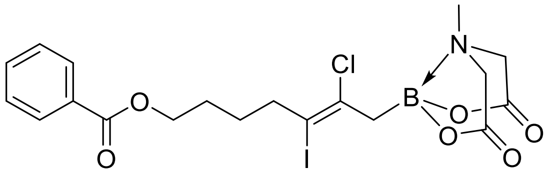

Following the general procedure **D**, the product **52** was obtained in 47% yield (rr = 4: 1) as a white solid after column chromatography (eluent = petroleum ether/ ethyl acetate 1:3 v/v). R_F = 0.34 (PE: ethyl acetate = 1:4). ^{1}H NMR (500 MHz, Acetone-d_6) δ 8.09 – 8.03 (m, 2H), 7.66 – 7.60 (m, 1H), 7.52 (t, *J* = 7.8 Hz, 2H), 4.36 (t, *J* = 6.3 Hz, 2H), 4.27 (d, *J* = 17.0 Hz, 2H), 4.09 (d, *J* = 16.9 Hz, 2H), 3.22 (s, 3H), 2.78 (t, *J* = 7.2 Hz, 2H), 2.43 (s, 2H), 1.87 – 1.80 (m, 2H), 1.78 – 1.71 (m, 2H). ^{11}B NMR (128 MHz, Acetone-d_6) δ 11.21. ^{13}C NMR (126 MHz, Acetone-d_6) δ 167.5, 165.9, 132.9, 130.6, 130.3, 129.3, 128.5, 97.3, 64.3, 62.0, 45.8, 41.1, 27.0, 25.0. **ESI-MS:** calcd for $C_{19}H_{22}NO_6BClINa$ [M + Na]$^+$: 556.0166, found: 556.0166.

2,2-difluoro-6-phenylhexan-1-ol (55)

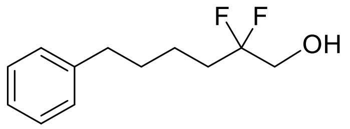

The product **55** was obtained in 52% yield as a colorless liquid after column chromatography (eluent = petroleum ether / ethyl acetate 10:1 v/v). R_F = 0.40 (petroleum ether: ethyl acetate = 8:1). ^{1}H NMR (400 MHz, Chloroform-*d*) δ 7.34 – 7.25 (m, 1H), 7.22 – 7.16 (m, 1H), 3.83 – 3.66 (m, 1H), 2.65 (t, *J* = 7.6 Hz, 1H), 2.04 (s, 1H), 2.02 – 1.83 (m, 1H), 1.69 (p, *J* = 7.5 Hz, 1H), 1.61 – 1.48 (m, 1H). ^{19}F NMR (376 MHz, Chloroform-*d*) δ -108.51. ^{13}C NMR (101 MHz, Chloroform-*d*) δ 141.1, 127.3, 127.3, 124.8, 122.2 (t, *J* = 241.6 Hz), 63.0 (t, *J* = 32.0 Hz), 34.6, 32.1 (t, *J* = 24.0 Hz), 30.2, 20.5 (t, *J* = 4.6 Hz). **EI-MS:** calcd for $C_{12}H_{16}F_2O$: 214.1169, found: 214.1167.

2-(2,2-difluoro-6-phenylhexyl)furan (56)

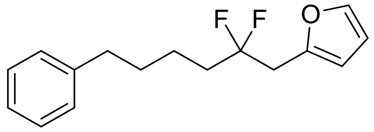

The product **56** was obtained in 40% yield (for two steps) as a colorless liquid after column chromatography (eluent = petroleum ether). R_F = 0.30 (petroleum ether). ^{1}H NMR (400 MHz, Chloroform-*d*) δ 7.35 (s, 1H), 7.27 (t, *J* = 7.7 Hz, 2H), 7.17 (t, *J* = 9.2 Hz, 3H), 6.33 (d, *J* = 2.7 Hz, 1H), 6.19 (d, *J* = 3.0 Hz, 1H), 3.18 (t, *J* = 14.7 Hz, 2H), 2.61 (t, *J* = 7.6 Hz, 2H), 1.93 – 1.73 (m, 2H), 1.65 (p, *J* = 7.6, 7.2 Hz, 2H), 1.55 (d, *J* = 7.6 Hz, 2H). ^{19}F NMR (376 MHz, Chloroform-*d*) δ -95.98. ^{13}C NMR (101 MHz, Chloroform-*d*) δ 147.8, 142.1, 128.4, 128.3, 125.8, 123.3 (t, *J* = 242.5 Hz), 110.6, 108.9, 35.8 (t, *J* = 29.0 Hz), 35.6 (t, *J* = 24.5 Hz), 31.1, 29.7, 21.8. **EI-MS:** calcd for $C_{16}H_{18}F_2O$: 264.1326, found: 264.1323.

(E)-(2-chloro-3-iodo-4-phenylbut-2-en-1-yl)trifluoro-l4-borane, potassium salt (57)

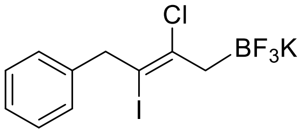

The product **57** was obtained in quant. (rr = 9: 1) as a white solid. ^{1}H NMR (400 MHz, Acetone-*d*6) δ 7.27 (d, *J* = 4.5 Hz, 4H), 7.23 – 7.17 (m, 1H), 4.06 (s, 2H), 1.99 – 1.92 (m, 2H). ^{11}B NMR (128 MHz, Acetone-*d*6) δ 7.04 – 0.97 (q). ^{19}F NMR (376 MHz, Acetone-*d*6) δ -136.97 – -139.59 (m). ^{13}C NMR (101 MHz, Acetone-*d*6) δ 139.0, 137.8, 128.5, 128.2, 126.1, 89.2, 47.6. **ESI-MS:** calcd for $C_{10}H_9BF_3ClIKNa$ [M + Na]$^+$:420.9013, found: 420.9009.

(E)-trifluoro(2-fluoro-3-iodo-4-phenylbut-2-en-1-yl)-l4-borane, potassium salt (58)

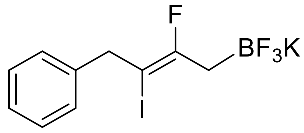

The product **58** was obtained in 84% yield as a white solid. ^{1}H NMR (400 MHz, Acetone-*d*6) δ 7.57 – 6.73 (m, 2H), 3.81 (d, *J* = 2.5 Hz, 1H), 1.83 – 1.66 (m, 1H). ^{11}B NMR (128 MHz, Acetone-*d*6) δ 3.71 (q, *J* = 57.1, 56.5 Hz). ^{19}F NMR (376 MHz, Acetone-*d*6) δ -86.04 (q, *J* = 2.9 Hz), -138.67 (dd, *J* = 108.4, 39.1 Hz). ^{13}C NMR (101 MHz, Acetone-*d*6) δ 164.6 (d, *J* = 258.3 Hz), 139.9 (d, *J* = 2.2 Hz), 128.5, 128.1, 126.0, 74.7 (d, *J* = 36.2 Hz), 41.2 (d, *J* = 8.3 Hz). **ESI-MS:** calcd for $C_{10}H_9BF_4I$ [M - K]$^-$: 342.9784, found: 342.9780.

(E)-(4-bromo-3-fluoro-2-iodobut-2-en-1-yl)benzene (59)

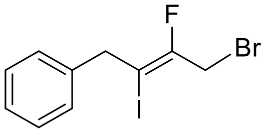

The product **59** was obtained in 59% yield as a colorless liquid after column chromatography (eluent = petroleum ether). R_F = 0.60 (petroleum ether). ^{1}H NMR (500 MHz, Chloroform-*d*) δ 7.33 (t, *J* = 7.2 Hz, 3H), 7.30 – 7.27 (m, 1H), 7.24 – 7.19 (m, 2H), 4.28 (d, *J* = 21.9 Hz, 2H), 3.87 (d, *J* = 3.5 Hz, 3H). ^{19}F NMR (376 MHz, Chloroform-*d*) δ -108.15. ^{13}C NMR (126 MHz, Chloroform-*d*) δ 152.7 (d, *J* = 252.9 Hz), 136.6 (d, *J* = 2.4 Hz), 128.8, 128.7, 127.1, 111.8 (d, *J* = 36.8 Hz), 39.0 (d, *J* = 3.6 Hz), 25.9 (d, *J* = 31.5 Hz). **EI-MS:** calcd for $C_{10}H_9IBrF$: 353.8916, found: 353.8908.

(E)-2-(2-fluoro-3-iodo-4-phenylbut-2-en-1-yl)-4,4,5,5-tetramethyl-1,3,2-dioxaborolane (60)

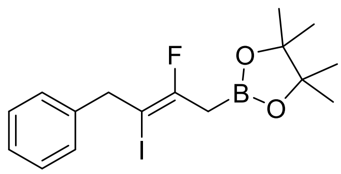

The product **60** was obtained in 60% yield as a colorless liquid after column chromatography (eluent = petroleum ether / ethyl acetate 50:1 v/v). R_F = 0.70 (petroleum ether: ethyl acetate = 10:1). ^{1}H NMR (400 MHz, Chloroform-*d*) δ 7.29 (dt, *J* = 7.6, 1.7 Hz, 1H), 7.25 – 7.19 (m, 2H), 3.85 (d, *J* = 2.6 Hz, 1H), 2.35 (d, *J* = 24.2 Hz, 1H), 1.27 (s, 6H). ^{11}B NMR (128 MHz, Chloroform-*d*) δ 31.76. ^{19}F NMR (376 MHz, Chloroform-*d*) δ -83.80. ^{13}C NMR (101 MHz, Chloroform-*d*) δ 155.9 (d, *J* = 260.1 Hz), 137.9 (d, *J* = 2.3 Hz), 127.7, 127.3, 125.5, 83.0, 79.6 (d, *J* = 31.9 Hz), 40.1 (d, *J* = 6.7 Hz), 23.8. **EI-MS:** calcd for $C_{16}H_{21}BFIO_2$: 402.0663, found: 402.0658.

(E)-2-fluoro-3-iodo-4-phenylbut-2-en-1-ol (61)

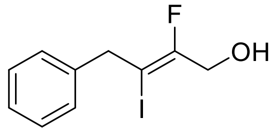

The product **61** was obtained in 75% yield as a colorless liquid after column chromatography (eluent = petroleum ether / ethyl acetate 10:1 v/v). R_F = 0.20 (petroleum ether: ethyl acetate = 9:1). ^{1}H NMR (500 MHz, Chloroform-*d*) δ 7.33 (dd, *J*

= 8.0, 6.4 Hz, 2H), 7.30 – 7.27 (m, 1H), 7.24 – 7.20 (m, 2H), 4.57 (dd, *J* = 19.9, 5.4 Hz, 2H), 3.91 (d, *J* = 2.8 Hz, 2H), 1.98 (t, *J* = 6.5 Hz, 1H). ^{19}F NMR (376 MHz, Chloroform-*d*) δ -100.75. ^{13}C NMR (126 MHz, Chloroform-*d*) δ 155.6 (d, *J* = 263.5 Hz), 137.7 (d, *J* = 2.4 Hz), 128.9, 128.6, 127.0, 84.1 (d, *J* = 28.7 Hz), 62.8 (d, *J* = 29.9 Hz), 41.3 (d, *J* = 6.3 Hz). **EI-MS:** calcd for $C_{10}H_{10}FIO$: 291.9760, found: 291.9753.

(E)-2-fluoro-3-iodo-4-phenylbut-2-en-1-yl benzoate (62)

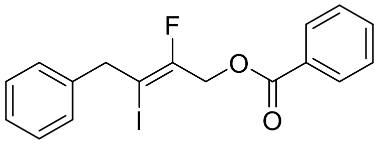

The product **62** was obtained in 66% yield as a colorless solid after column chromatography (eluent = petroleum ether / ethyl acetate 50:1 v/v). R_F = 0.50 (petroleum ether: ethyl acetate = 9:1). ^{1}H NMR (500 MHz, Chloroform-*d*) δ 8.01 (dd, *J* = 8.1, 1.5 Hz, 2H), 7.50 (t, *J* = 7.4 Hz, 1H), 7.38 (t, *J* = 7.7 Hz, 2H), 7.25 (t, *J* = 7.2 Hz, 2H), 7.22 – 7.17 (m, 1H), 7.17 – 7.09 (m, 2H), 5.18 (d, *J* = 19.3 Hz, 2H), 3.87 (d, *J* = 2.8 Hz, 2H). ^{19}F NMR (376 MHz, Chloroform-*d*) δ -98.3. ^{13}C NMR (126 MHz, Chloroform-*d*) δ 166.0, 152.4 (d, *J* = 263.2 Hz), 137.5 (d, *J* = 2.4 Hz), 133.4, 129.9, 129.4, 128.9, 128.7, 128.5, 127.1, 87.2 (d, *J* = 27.6 Hz), 63.9 (d, *J* = 29.4 Hz), 41.6 (d, *J* = 5.9 Hz).

(E)-2-fluoro-3,4-diphenylbut-2-en-1-yl benzoate (63)

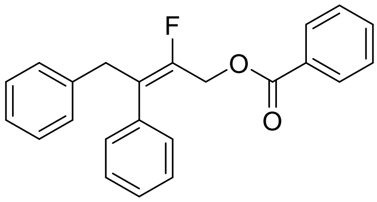

The product **63** was obtained in 47% yield as a colorless liquid after column chromatography (eluent = petroleum ether / ethyl acetate 50:1 v/v). R_F = 0.40 (petroleum ether: ethyl acetate = 9:1). ^{1}H NMR (500 MHz, Chloroform-*d*) δ 8.19 – 8.07 (m, 2H), 7.61 (t, *J* = 7.4 Hz, 1H), 7.49 (t, *J* = 7.7 Hz, 2H), 7.33 – 7.23 (m, 5H), 7.20 (t, *J* = 7.3 Hz, 1H), 7.18 – 7.09 (m, 4H), 4.89 (d, *J* = 21.2 Hz, 2H), 3.88 (d, *J* = 3.0 Hz, 2H). ^{19}F NMR (376 MHz, Chloroform-*d*) δ -115.63. ^{13}C NMR (101 MHz, Chloroform-*d*) δ 165.0, 150.4 (d, *J* = 253.8 Hz), 137.2 (d, *J* = 2.3 Hz), 135.7 (d, *J* = 7.0 Hz), 132.1, 128.8, 127.8, 127.4, 127.3, 126.8, 125.2, 124.4 (d, *J* = 16.8 Hz), 59.7 (d, *J* = 29.4 Hz), 35.5 (d, *J* = 5.5 Hz). **ESI-MS:** calcd for $C_{23}H_{19}FO_2Na$ $[M + Na]^+$: 369.1262, found: 369.1260.

4-methyl-*N'*-(1-(6-methyl-4,8-dioxo-1,3,6,2-dioxazaborocan-2-yl)hexan-2-ylidene)benzenesulfonohydrazide (64)

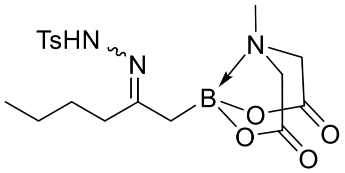

The product **64** was obtained in 25% yield as a semi solid after column chromatography (eluent = petroleum ether/ ethyl acetate 1:2 v/v). R_F = 0.70 (ethyl acetate). ^{1}H NMR (400 MHz, Chloroform-*d*) δ 7.74 (d, *J* = 8.1 Hz, 2H), 7.57 (s, 1H), 7.33 (d, *J* = 7.9 Hz, 2H), 4.01 (d, *J* = 16.4 Hz, 2H), 3.72 (d, *J* = 16.4 Hz, 2H), 3.04 (s, 3H), 2.43 (s, 3H), 2.16 (t, *J* = 10.0 Hz, 2H), 1.88 (s, 2H), 1.43 – 1.27 (m, 4H), 0.88 (t, *J* = 7.1 Hz, 3H). ^{13}C NMR (101 MHz, $CDCl_3$) δ 167.5, 163.2, 144.6, 135.7, 130.1, 127.7, 63.2, 46.1, 31.8, 27.2, 22.9, 21.8, 13.9. ^{11}B NMR (128 MHz, $CDCl_3$) δ 12.4. **ESI-MS:** calcd for $C_{18}H_{26}BN_3O_6S$ Na $[M + Na]^+$: 446.1528, found: 446.1536.

2-(2-hydroxyhexyl)-6-methyl-1,3,6,2-dioxazaborocane-4,8-dione (65)

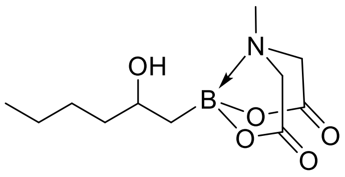 The product **65** was obtained in 96% yield as a semi solid after column chromatography (eluent = ethyl acetate / acetone 10:1 v/v). R_F = 0.15 (ethyl acetate). ^{1}H NMR (400 MHz, Acetone-d_6) δ 4.20 – 3.91 (m, 4H), 3.81 – 3.69 (m, 1H), 3.55 (s, 1H), 3.15 (s, 3H), 1.53 – 1.45 (m,2H), 1.35 – 1.28 (m, 2H), 0.99 – 0.76 (m, 5H). ^{13}C NMR (101 MHz, Acetone-d_6) δ 169.6, 168.7, 69.5, 63.2, 62.7, 46.7, 43.9, 19.6, 14.5. ^{11}B NMR (128 MHz, Acetone-d_6) δ 13.0. **ESI-MS:** calcd for $C_{11}H_{20}BNO_5Na$ [M + Na]$^+$: 280.1327, found: 280.1329.

(*E*)-2-(hex-1-en-1-yl)-6-methyl-1,3,6,2-dioxazaborocane-4,8-dione (66)

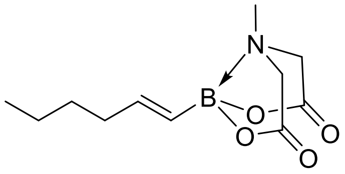 The product **66** was obtained in 77% yield as a white solid after column chromatography (eluent = petroleum ether/ ethyl acetate 1:3 v/v). RF = 0.66 (ethyl acetate). ^{1}H NMR (400 MHz, DMSO-d_6) δ 5.94 (dt, *J* = 17.6, 6.4 Hz, 1H), 5.37 (d, *J* = 17.6 Hz, 1H), 4.18 (d, *J* = 17.1 Hz, 2H), 3.95 (d, *J* = 17.0 Hz, 2H), 3.33 (s, 1H), 2.73 (s, 3H), 2.07 (q, *J* = 7.5, 7.0 Hz, 2H), 1.43 – 1.23 (m, 6H), 0.87 (t, *J* = 7.2 Hz, 3H). ^{13}C NMR (101 MHz, DMSO-d_6) δ 169.2, 144.2, 61.2, 46.7, 34.5, 30.5, 21.7, 13.8. ^{11}B NMR (128 MHz, DMSO-d_6) δ 12.3. **ESI-MS:** calcd for $C_{11}H_{18}BNO_4Na$ [M + Na]$^+$: 262.1221, found: 262.1219.

hexane-1,2-diol (67)

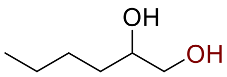 The product **67** was obtained in 86% yield as a pale yellow liquid column chromatography (eluent = petroleum ether/ ethyl acetate 2:1 v/v). RF = 0.75 (petroleum ether: ethyl acetate = 1:1). ^{1}H NMR (400 MHz, Chloroform-*d*) δ 3.79 – 3.64 (m, 2H), 3.53 – 3.36 (m, 1H), 1.36 – 1.22 (m, 6H), 0.91 (t, *J* = 6.6 Hz, 3H). Data in concordance with literature.[5]

9. Mechanistic experiments

9.1 Competition experiment: propargyl B(MIDA) reacts preferentially

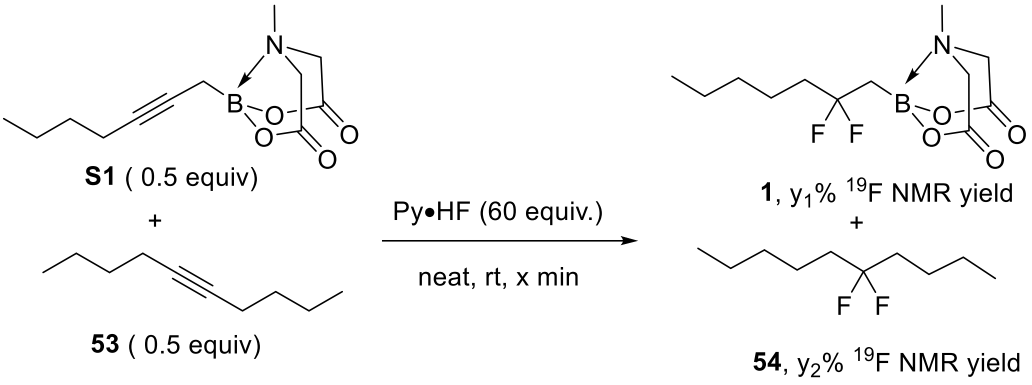

To a 10 mL of plastic tubing charged with propargylic MIDA boronates **S1** (0.05 mmol, 0.5 equiv.), compound **53** (0.05 mmol, 0.5 equiv.), was added Py·9HF (60.0 equiv) in one portion. The reaction was allowed to stir at room temperature for x min. The reaction mixture was quenched by slowly adding basic alumina suspended in dichloromethane (0.9 g). The suspension was filtered and washed with 25 mL of dichloromethane. The combined organic layer was dried over anhydrous Na_2SO_4 and concentrated under reduced pressure. The internal standard ((trifluoromethyl)benzene) was added and used acetone-d_6 as the solvent to determine the ^{19}F-NMR yield.

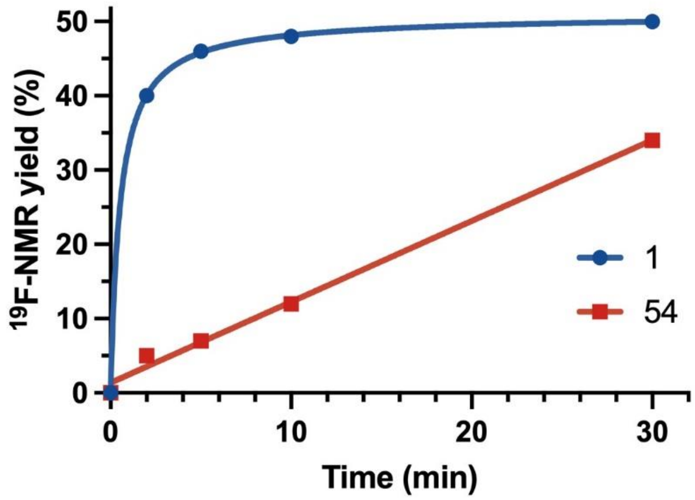

Figure S2. Competition experiment of difluorination reaction.

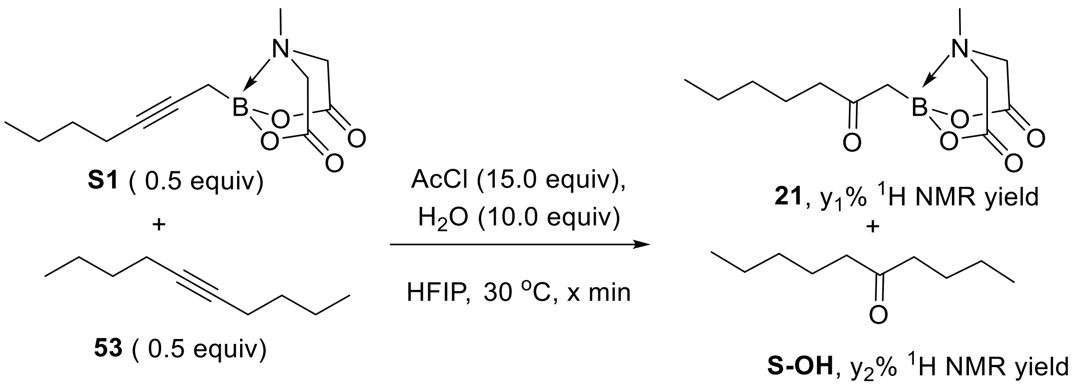

The MIDA boronates **S1** (0.05 mmol, 0.5 equiv.), compound **53** (0.05 mmol, 0.5 equiv.) were added to a 15 mL oven-dried pressure tube equipped with a stir bar. HFIP (2.0 mL) and H_2O (2.0 mmol, 10.0 equiv.) were added to the tube under air. The solution was stirred for 15 seconds and then add AcCl (3.0 mmol, 15.0 equiv.) was added following sealing the tube quickly with a thread plug. The solution was stirred at 30 °C for x min. The solvent was removed in vacuo and the internal standard ((4-Iodoanisole) was added and used acetone-d_6 as the solvent to determine the ^{1}H-NMR yield.

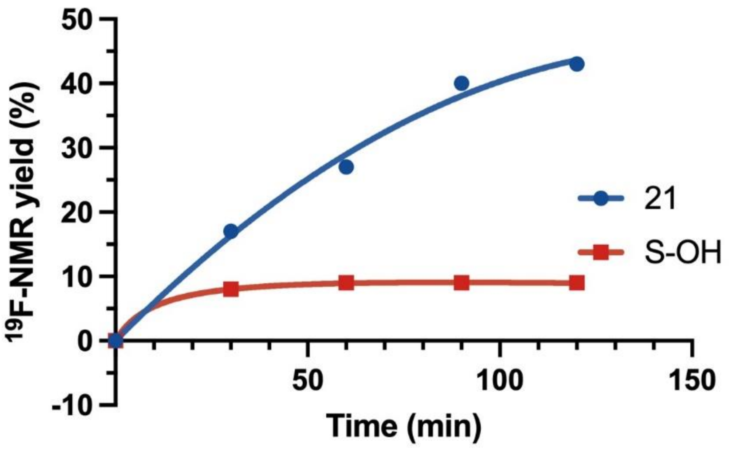

Figure S3. Competition experiment of hydration reaction.

9.2 Computational Studies

9.2.1 Computational details

The DFT calculations were performed by Gaussian 09 programs[7]. Geometries of the minimum energy structures were optimized at the M06[8] level of theory with the 6-31G(d, p) basis using SMD[9] solvation model (solvent = Dichloromethane). After optimizing the structure, we obtained the wavefuction, and the electronic structure analysis was performed with the Multiwfn (Multifunctional Wavefunction Analyzer)[10] including orbital composition analysis with Mulliken partition[11]. Visualization of HOMO orbitals and optimized structures was carried out by VMD (Visual Molecular Dynamics)[12] software.

9.2.2 Cartesian Coordinates and Energies of the Optimized Structures

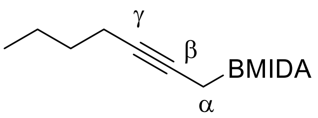

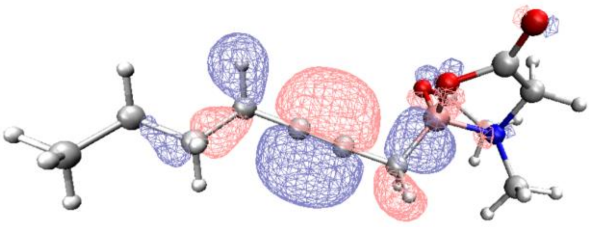

HOMO

Zero-point correction= 0.297487 (Hartree/Particle)
Thermal correction to Energy= 0.315663
Thermal correction to Enthalpy= 0.316607
Thermal correction to Gibbs Free Energy= 0.249673
Sum of electronic and zero-point Energies= -847.921523
Sum of electronic and thermal Energies= -847.903347
Sum of electronic and thermal Enthalpies= -847.902403
Sum of electronic and thermal Free Energies= -847.969337

Orbital 67 is HOMO, energy: -0.245687 a.u. -6.685491 eV
Orbital 68 is LUMO, energy: 0.011578 a.u. 0.315066 eV
HOMO-LUMO gap: 0.257266 a.u. 7.000557 eV 675.451204 kJ/mol

Orbital 67 is HOMO
Composition of each atom:
Atom 22($C_β$): 32.43329%
Atom 23($C_γ$): 38.35015%

NBO charge:
Atom 22($C_β$): -0.019
Atom 23($C_γ$): -0.041

O	0.44323800	9.20697200	6.86804500
O	-0.95231100	9.45732000	8.60963300
O	0.41896800	5.03759100	5.14162900
O	0.98375200	7.13105000	5.72620700
N	-1.10942400	8.21688700	5.28377300
C	-0.70537300	9.03561500	7.50820700
C	1.24988100	9.34409200	4.44329400
C	-1.66278100	8.21494700	6.66707600
C	0.17835800	6.21748300	5.20614000
C	-1.79508900	9.22553100	4.44573600
C	-1.08606400	6.86873500	4.67484900
B	0.49517300	8.51710100	5.58321300
H	1.12672100	8.84448300	3.46890600

H	0.80634000	10.34528300	4.33788900
H	-1.68512300	7.19341400	7.06268000
H	-2.67903100	8.61655600	6.69341800
H	-2.84763200	8.94975500	4.32998600
H	-1.31488000	9.26998100	3.46584300
H	-1.72372600	10.20193600	4.93450500
H	-1.00288100	6.96892600	3.58616200
H	-1.98466000	6.29121600	4.90898000
C	2.66606700	9.46218600	4.76291500
C	3.83758900	9.52652400	5.06660700
C	5.25050200	9.62978200	5.41463500
H	5.81331200	8.83268600	4.90513400
H	5.38079700	9.44203600	6.49134800
C	5.86745700	10.98411900	5.06301100
H	5.74668000	11.17087600	3.98533600
H	5.31037700	11.78199600	5.57656600
C	7.33856600	11.05923800	5.43785600
H	7.88380200	10.25225100	4.92489400
H	7.44696800	10.85970000	6.51483200
C	7.95856800	12.40228900	5.09567600
H	9.01828500	12.44581400	5.37136800
H	7.88682800	12.60792800	4.01974600
H	7.44514800	13.21907300	5.61947600

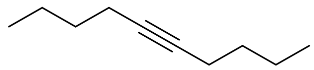

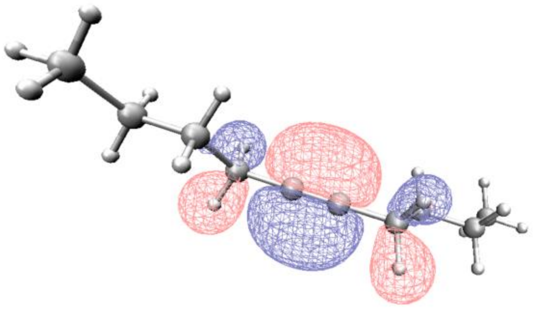

HOMO

Zero-point correction=	0.254500 (Hartree/Particle)
Thermal correction to Energy=	0.267680
Thermal correction to Enthalpy=	0.268624
Thermal correction to Gibbs Free Energy=	0.213608
Sum of electronic and zero-point Energies=	-391.310675
Sum of electronic and thermal Energies=	-391.297495
Sum of electronic and thermal Enthalpies=	-391.296551
Sum of electronic and thermal Free Energies=	-391.351567

Orbital 39 is HOMO, energy: -0.256556 a.u. -6.981251 eV

Orbital 40 is LUMO, energy: 0.067661 a.u. 1.841151 eV

HOMO-LUMO gap: 0.324217 a.u. 8.822402 eV 851.232608 kJ/mol

C	1.27940900	9.30697400	4.46473800
H	1.15429500	8.81247900	3.48946700
H	0.83651200	10.30788800	4.34968300
C	2.69942400	9.43062800	4.77521000
C	3.87275000	9.50342800	5.06799700
C	5.28723700	9.61968900	5.40445500
H	5.85628300	8.83531100	4.88264300
H	5.42683400	9.42316900	6.47840500
C	5.88259400	10.98626100	5.06223100
H	5.75402600	11.18045200	3.98696100
H	5.31683100	11.77160000	5.58533900
C	7.35418300	11.07781800	5.43119400
H	7.90709600	10.27997400	4.91233800
H	7.46961200	10.87505200	6.50684900
C	7.95576100	12.42998700	5.09201700
H	9.01575500	12.48573000	5.36422200
H	7.87763700	12.63849400	4.01714300
H	7.43405400	13.23845300	5.62050200
C	0.50318000	8.53002300	5.52908000
H	0.94396700	7.52802600	5.64055700
H	0.62612200	9.02746300	6.50279100
C	-0.97472500	8.40839800	5.19531800
H	-1.08585300	7.91683500	4.21674100
H	-1.40335900	9.41539100	5.07771400
C	-1.74460500	7.63752800	6.25272800
H	-2.80907400	7.55545900	6.00588900
H	-1.34972200	6.61940700	6.36449500
H	-1.66728800	8.12692000	7.23230000

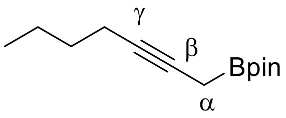

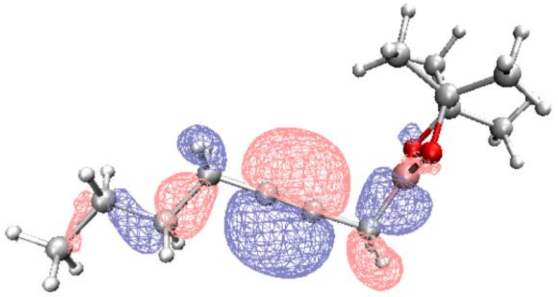

HOMO

Zero-point correction= 0.340902 (Hartree/Particle)
Thermal correction to Energy= 0.359595
Thermal correction to Enthalpy= 0.360539

Thermal correction to Gibbs Free Energy= 0.293433
Sum of electronic and zero-point Energies= -683.822357
Sum of electronic and thermal Energies= -683.803664
Sum of electronic and thermal Enthalpies= -683.802720
Sum of electronic and thermal Free Energies= -683.869826

Orbital 61 is HOMO, energy: -0.251304 a.u. -6.838327 eV
Orbital 62 is LUMO, energy: 0.046805 a.u. 1.273630 eV
HOMO-LUMO gap: 0.298109 a.u. 8.111957 eV 782.685023 kJ/mol

Orbital 61 is HOMO
Composition of each atom:
Atom 4(C_β): 34.11766 %
Atom 5(C_γ): 38.72647 %

NBO charge:
Atom 4(C_β): -0.026
Atom 5(C_γ): -0.039

C	1.59898900	7.80166300	2.73216400
H	0.89778200	7.55678800	3.54016900
H	1.78392100	6.87960200	2.16538000
C	2.84852800	8.32036700	3.27890700
C	3.87491400	8.81404700	3.69452600
C	5.12024400	9.36116400	4.22197900
H	4.90550200	10.26873300	4.80644000
H	5.76025800	9.68930900	3.38882200
C	5.89486000	8.37153000	5.09340500
H	5.25840700	8.04974300	5.93127200
H	6.11248300	7.46550400	4.50828900
C	7.18911200	8.96352100	5.62701700
H	6.96023400	9.87374500	6.20207900
H	7.81353100	9.28897300	4.78100000
C	7.96074900	7.98413900	6.49331100
H	8.89217400	8.41902300	6.87320300
H	7.36458000	7.66845300	7.35931500
H	8.22280800	7.07957500	5.92931200
C	0.93057900	10.37247500	0.07153400
C	-0.23534700	10.66076900	1.06549300
B	1.00564500	8.91750200	1.79105300
C	2.12710600	11.28969600	0.26937300
H	2.96798900	10.90499400	-0.31846800
H	1.91398800	12.31069500	-0.06700200
H	2.43811100	11.32384200	1.32111100

C	0.53390500	10.33497400	-1.38708700
H	0.12969500	11.30489000	-1.70242200
H	1.41547800	10.12218400	-2.00257500
H	-0.21365700	9.56228200	-1.58926000
C	-0.39193300	12.11139300	1.46463200
H	-0.60391500	12.73247700	0.58543900
H	-1.23402100	12.21368600	2.15871600
H	0.50311000	12.49902100	1.96001800
C	-1.56973500	10.09775000	0.60468100
H	-2.28068000	10.14508000	1.43710800
H	-1.98282100	10.67621300	-0.22960900
H	-1.48175300	9.05041100	0.29235200
O	0.16026600	9.89490700	2.23234000
O	1.36032900	9.04797500	0.47810500

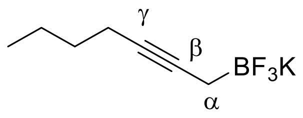

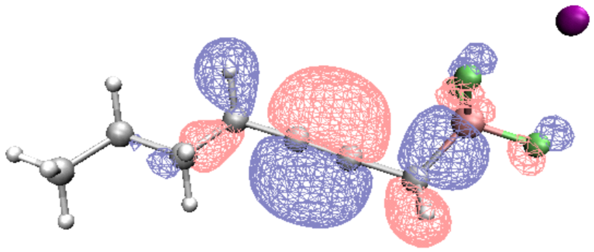

HOMO

Orbital 52 is HOMO, energy: -0.235984 a.u. -6.421449 eV

Orbital 53 is LUMO, energy: -0.056151 a.u. -1.527956 eV

HOMO-LUMO gap: 0.179833 a.u. 4.893493 eV 472.150380 kJ/mol

Orbital 52 is HOMO

Composition of each atom:

Atom 5($C_β$): 29.57052%

Atom 6($C_γ$): 38.22098%

NBO charge:

Atom 5($C_β$): -0.015

Atom 6($C_γ$): -0.073

C	1.28914800	9.28462400	4.46710300
B	0.43097800	8.55006700	5.60944300
H	1.15921100	8.73976200	3.51994800
H	0.86553600	10.28601300	4.29806000
C	2.70389000	9.39412900	4.78502000
C	3.87861800	9.46375700	5.08011800
C	5.29683800	9.58730200	5.40262000

H	5.86874100	8.82238100	4.85463600
H	5.46142200	9.37024700	6.46943200
C	5.87195000	10.96726200	5.07945600
H	5.71611800	11.18461000	4.01207300
H	5.30882100	11.73413100	5.63241800
C	7.35036700	11.07072800	5.41619300
H	7.90214000	10.29516400	4.86326100
H	7.49488100	10.84124200	6.48299900
C	7.92480500	12.44012400	5.09909900
H	8.99088500	12.50444300	5.34466400
H	7.81488500	12.67665100	4.03275200
H	7.40595500	13.22647200	5.66267600
F	0.86184900	7.22539200	5.87879600
F	0.45634400	9.23319000	6.85707700
F	-0.94398600	8.44883600	5.24860600
K	-1.22303300	7.33604800	7.58782400

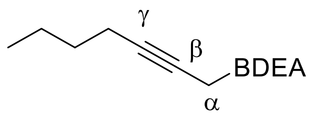

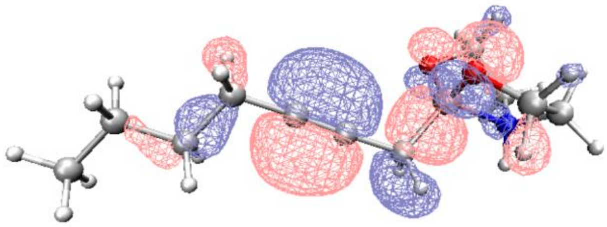

HOMO

Orbital 57 is HOMO, energy: -0.227563 a.u. -6.192304 eV

Orbital 58 is LUMO, energy: 0.071530 a.u. 1.946437 eV

HOMO-LUMO gap: 0.299093 a.u. 8.138741 eV 785.269287 kJ/mol

Orbital 57 is HOMO

Composition of each atom:

Atom 8($C_β$): 19.45754%

Atom 9($C_γ$): 30.05451%

NBO charge:

Atom 8($C_β$): -0.026

Atom 9($C_γ$): -0.039

O	0.45530300	8.23316400	7.11167500
O	1.12899400	7.05687700	5.09322800
N	-1.07023700	8.12340800	5.22813900
C	1.21248000	9.60736900	5.03867500

B	0.59109700	8.25253400	5.67035900
H	1.03892700	9.61436100	3.95062700
H	0.68808700	10.48950600	5.44006000
C	2.63654900	9.71823000	5.31023100
C	3.82249400	9.77255700	5.56100400
C	5.25168200	9.88219100	5.83666100
H	5.75436800	8.93500300	5.58706700
H	5.41083400	10.02329900	6.91697300
C	5.92858500	11.02433700	5.07790000
H	5.78306100	10.87781400	3.99691800
H	5.42788900	11.97213600	5.32723400
C	7.41293700	11.12957700	5.38784600
H	7.90207000	10.17361800	5.14572300
H	7.54676100	11.27109100	6.47129100
C	8.08591700	12.26109800	4.63158000
H	9.15504800	12.32939200	4.86246900
H	7.98860500	12.12360700	3.54680600
H	7.63000600	13.22831400	4.88015600
H	-1.37974800	8.89019600	4.63293700
C	-0.77585900	8.77624100	7.47723100
H	-0.79217200	9.87811600	7.37221500
H	-1.00783400	8.54536700	8.52487000
C	-1.78908500	8.15368500	6.52683100
H	-2.73398100	8.69787500	6.44449500
H	-2.00059700	7.12004700	6.82147600
C	-1.10283600	6.83606300	4.49141100
H	-0.97042800	7.06078200	3.42820400
H	-2.05519800	6.31503500	4.63514200
C	0.11615000	6.09327400	5.02166300
H	0.40916800	5.27352200	4.35405800
H	-0.10312500	5.65276000	6.01099700

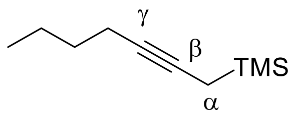

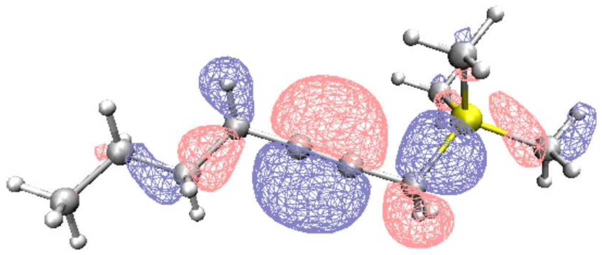

HOMO

Orbital 47 is HOMO, energy: -0.240343 a.u. -6.540062 eV

Orbital 48 is LUMO, energy: 0.069529 a.u. 1.891992 eV

HOMO-LUMO gap: 0.309872 a.u. 8.432054 eV 813.569715 kJ/mol

Orbital 47 is HOMO

Composition of each atom:

Atom 4(C_β): 25.81903%

Atom 5(C_γ): 36.32968%

NBO charge:

Atom 4(C_β): -0.035

Atom 5(C_γ): -0.053

C	1.62151900	8.16823800	3.12631100
H	0.89168700	8.13419200	3.94872400
H	1.65201500	7.15675000	2.69475800
C	2.93136700	8.56522800	3.60832400
C	4.02498700	8.95439400	3.96298200
C	5.35001000	9.35669200	4.42420000
H	5.26597900	10.25505400	5.05455900
H	5.96702200	9.65670200	3.56333400
C	6.07483300	8.25933000	5.20443100
H	5.46504800	7.96840000	6.07295300
H	6.15706800	7.36096600	4.57442100
C	7.45660800	8.69257900	5.66682500
H	7.36373500	9.59603400	6.28869100
H	8.05455700	8.98876500	4.79139600
C	8.17643200	7.60286600	6.44101300
H	9.16977800	7.92473400	6.77354600
H	7.60652400	7.31030100	7.33245700
H	8.30708700	6.70259800	5.82671500
Si	0.97133300	9.36986400	1.79517500
C	2.19557200	9.40991600	0.37343200
H	1.85100500	10.07599800	-0.42729100
H	3.17643600	9.76957200	0.70822300
H	2.33452100	8.41130200	-0.05970800
C	0.80063300	11.07431200	2.56147000
H	0.41030500	11.79866200	1.83586800
H	0.11612300	11.05855500	3.41889000
H	1.77092100	11.44527500	2.91462100
C	-0.69579000	8.72743100	1.21780100
H	-1.40498700	8.65931500	2.05226300
H	-1.13541500	9.38725300	0.45966100
H	-0.60204300	7.72753100	0.77573900

10. X-ray crystal structure data

Crystal structure data for 48:

Experimental

Single crystals of $C_{15}H_{16}BClINO_4$ **48** were colorless crystal. A suitable crystal was selected on a XtaLAB Synergy R, DW system, HyPix diffractometer. The crystal was kept at 100.00(10) K during data collection. Using Olex2 [1], the structure was solved with the SHELXT [2] structure solution program using Intrinsic Phasing and refined with the SHELXL [3] refinement package using Least Squares minimisation.

1. Dolomanov, O.V., Bourhis, L.J., Gildea, R.J, Howard, J.A.K. & Puschmann, H. (2009), J. Appl. Cryst. 42, 339-341.
2. Sheldrick, G.M. (2015). Acta Cryst. A71, 3-8.
3. Sheldrick, G.M. (2015). Acta Cryst. C71, 3-8.

Crystal structure determination of 48

Crystal Data for $C_{15}H_{16}BClINO_4$ (M =447.45 g/mol): monoclinic, space group P21/c (no. 14), a = 11.4048(4) Å, b = 10.7909(5) Å, c = 13.7249(5) Å, β = 94.575(4)°, V = 1683.71(12) Å^3, Z = 4, T = 100.00(10) K, μ(Cu Kα) = 16.566 mm^{-1}, Dcalc = 1.765 g/cm^3, 10495 reflections measured (7.776° ≤ 2Θ ≤ 151.04°), 3291 unique (R_{int} = 0.0798, R_{sigma} = 0.0798) which were used in all calculations. The final R_1 was 0.0650 (I > 2σ(I)) and wR_2 was 0.1878 (all data).

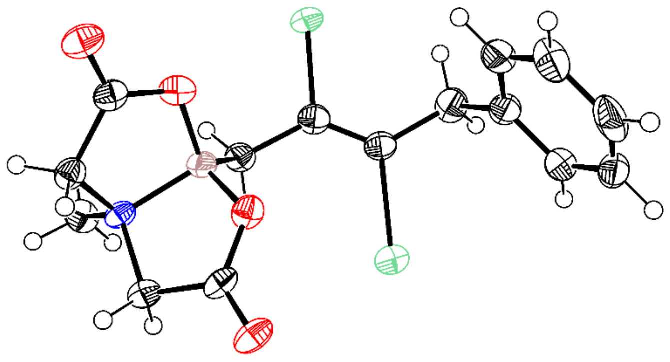

Figure S4. Absolute configuration of 48 (CCDC 2254248).

Table S5. Crystal data and structure refinement for 48.

Identification code	liy_230313_auto
Empirical formula	$C_{15}H_{16}BClINO_4$
Formula weight	447.45
Temperature/K	100.00(10)
Crystal system	monoclinic
Space group	$P2_1/c$

a/Å	11.4048(4)
b/Å	10.7909(5)
c/Å	13.7249(5)
α/°	90
β/°	94.575(4)
γ/°	90
Volume/Å^3	1683.71(12)
Z	4
ρ_{calc}mg/mm^3	1.765
μ/mm^{-1}	16.566
F(000)	880.0
Crystal size/mm^3	0.1 × 0.1 × 0.05
2Θ range for data collection	7.776 to 151.04°
Index ranges	-14 ≤ h ≤ 14, -9 ≤ k ≤ 13, -17 ≤ l ≤ 14
Reflections collected	10495
Independent reflections	3291[R(int) = 0.0798]
Data/restraints/parameters	3291/0/209
Goodness-of-fit on F^2	1.036
Final R indexes [I>=2σ (I)]	R_1 = 0.0650, wR_2 = 0.1742
Final R indexes [all data]	R_1 = 0.0796, wR_2 = 0.1878
Largest diff. peak/hole / e Å^{-3}	1.72/-0.87

Crystal structure data for 62:

Experimental

Single crystals of $C_{17}H_{14}FIO_2$ **62** were colorless crystal. A suitable crystal was selected on a XtaLAB Synergy R, DW system, HyPix diffractometer. The crystal was kept at 100.00(10) K during data collection. Using Olex2 [1], the structure was solved with the SHELXT [2] structure solution program using Intrinsic Phasing and refined with the SHELXL [3] refinement package using Least Squares minimisation.

1. Dolomanov, O.V., Bourhis, L.J., Gildea, R.J, Howard, J.A.K. & Puschmann, H. (2009), J. Appl. Cryst. 42, 339-341.
2. Sheldrick, G.M. (2015). Acta Cryst. A71, 3-8.
3. Sheldrick, G.M. (2015). Acta Cryst. C71, 3-8.

Crystal structure determination of 62

Crystal Data for $C_{17}H_{14}FIO_2$ (M =396.18 g/mol): triclinic, space group P-1 (no. 2), a = 6.04220(10) Å, b = 8.46130(10) Å, α = 101.8730(10)°, β = 99.9370(10)°, γ = 100.9850(10)°, V = 743.993(16) Å^3, Z = 2, T = 100.00(10) K, μ(Cu Kα) = 17.028 mm^{-1}, Dcalc = 1.768 g/cm^3, 29504 reflections measured (5.962° ≤ 2Θ ≤ 157.066°), 3072 unique (R_{int} = 0.0493, R_{sigma} = 0.0217) which were used in all calculations. The final R_1 was 0.0251 (I > 2σ(I)) and wR_2 was 0.0667 (all data).

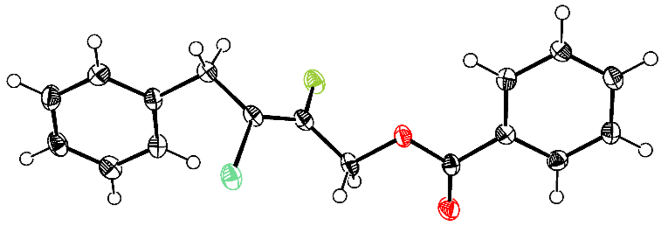

Figure S5. Absolute configuration of 62 (CCDC 2259628).

Table S6. Crystal data and structure refinement for 62.

Identification code	liy_230426_auto
Empirical formula	$C_{17}H_{14}FIO_2$
Formula weight	396.18
Temperature/K	100.00(10)
Crystal system	triclinic
Space group	P-1
a/Å	6.04220(10)
b/Å	8.46130(10)
c/Å	15.53020(10)
α/°	101.8730(10)
β/°	99.9370(10)
γ/°	100.9850(10)
Volume/Å^3	743.993(16)
Z	2
ρcalcg/cm^3	1.768
μ/mm^{-1}	17.028
F(000)	388.0
Crystal size/mm^3	0.1 × 0.1 × 0.05
Radiation	Cu Kα (λ = 1.54184)
2Θ range for data collection/°	5.962 to 157.066
Index ranges	$-7 \le h \le 7$, $-10 \le k \le 10$, $-19 \le l \le 19$
Reflections collected	29504
Independent reflections	3072 [R_{int} = 0.0493, R_{sigma} = 0.0217]
Data/restraints/parameters	3072/0/191
Goodness-of-fit on F^2	1.136
Final R indexes [I>=2σ (I)]	R_1 = 0.0251, wR_2 = 0.0663
Final R indexes [all data]	R_1 = 0.0260, wR_2 = 0.0667
Largest diff. peak/hole / e Å^{-3}	0.72/-0.92

11. References

1. Liu Y., Chen Z.-H., Li Y., Qian J., Li Q. & Wang H. Boryl-Dictated Site-Selective Intermolecular Allylic and Propargylic C–H Amination. *J. Am. Chem. Soc.* 144, 14380-14387 (2022).

2. Hassan H., Pirenne V., Wissing M., Khiar C., Hussain A., Robert F. & Landais Y. Free-Radical Carbocyanation of Olefins. *Chemistry – A European Journal.* 23, 4651-4658 (2017).

3. Qian H., Yu X., Zhang J. & Sun J. Organocatalytic Enantioselective Synthesis of 2,3-Allenoates by Intermolecular Addition of Nitroalkanes to Activated Enynes. *J. Am. Chem. Soc.* 135, 18020-18023 (2013).

4. Li Y., Fan W.-X., Luo S., Trofimova A., Liu Y., Xue J.-H., Yang L., Li Q., Wang H. & Yudin A. K. β-Boron Effect Enables Regioselective and Stereospecific Electrophilic Addition to Alkenes. *J. Am. Chem. Soc.* 145, 7548-7558 (2023).

5. Deadman B. J., Gian S., Lee V. E. Y., Adrio L. A., Hellgardt K. & Hii K. K. On-demand, in situ, generation of ammonium caroate (peroxymonosulfate) for the dihydroxylation of alkenes to vicinal diols. *Green Chem.* 24, 5570-5578 (2022).

6. Lv W.-X., Li Y., Cai Y.-H., Tan D.-H., Li Z., Li J.-L., Li Q. & Wang H. Hypervalent iodine-mediated β-difluoroalkylboron synthesis via an unusual 1,2-hydrogen shift enabled by boron substitution. *Chem. Sci.* 13, 2981-2984 (2022).

7. Frisch M. J., Trucks G. W., Schlegel H. B., Scuseria G. E., Robb M. A., Cheeseman J. R., Scalmani G., Barone V., Petersson G. A., Nakatsuji H., Li X., Caricato M., Marenich A. V., Bloino J., Janesko B. G., Gomperts R., Mennucci B., Hratchian H. P., Ortiz J. V., Izmaylov A. F., Sonnenberg J. L., Williams, Ding F., Lipparini F., Egidi F., Goings J., Peng B., Petrone A., Henderson T., Ranasinghe D., Zakrzewski V. G., Gao J., Rega N., Zheng G., Liang W., Hada M., Ehara M., Toyota K., Fukuda R., Hasegawa J., Ishida M., Nakajima T., Honda Y., Kitao O., Nakai H., Vreven T., Throssell K., Montgomery Jr. J. A., Peralta J. E., Ogliaro F., Bearpark M. J., Heyd J. J., Brothers E. N., Kudin K. N., Staroverov V. N., Keith T. A., Kobayashi R., Normand J., Raghavachari K., Rendell A. P., Burant J. C., Iyengar S. S., Tomasi J., Cossi M., Millam J. M., Klene M., Adamo C., Cammi R., Ochterski J. W., Martin R. L., Morokuma K., Farkas O., Foresman J. B. & Fox D. J. in *Gaussian 09 Rev. D.01*, *Vol.* Wallingford, CT, **2013**.

8. Zhao Y. & Truhlar D. G. The M06 suite of density functionals for main group thermochemistry, thermochemical kinetics, noncovalent interactions, excited states, and transition elements: two new functionals and systematic testing of four M06-class functionals and 12 other functionals. *Theor. Chem. Acc.* 120, 215-241 (2008).

9. Marenich A. V., Cramer C. J. & Truhlar D. G. Universal Solvation Model Based on Solute Electron Density and on a Continuum Model of the Solvent Defined by the Bulk Dielectric Constant and Atomic Surface Tensions. *The Journal of Physical Chemistry B.* 113, 6378-6396 (2009).

10. Lu T. & Chen F. Multiwfn: A multifunctional wavefunction analyzer.

Journal of Computational Chemistry. 33, 580-592 (2012).
11. Lu Tian C. F.-W. Calculation of Molecular Orbital Composition. *Acta Chim. Sinica.* 69, 2393-2406 (2011).
12. Humphrey W., Dalke A. & Schulten K. VMD: Visual molecular dynamics. *Journal of Molecular Graphics.* 14, 33-38 (1996).

12. NMR spectra of the starting materials and products

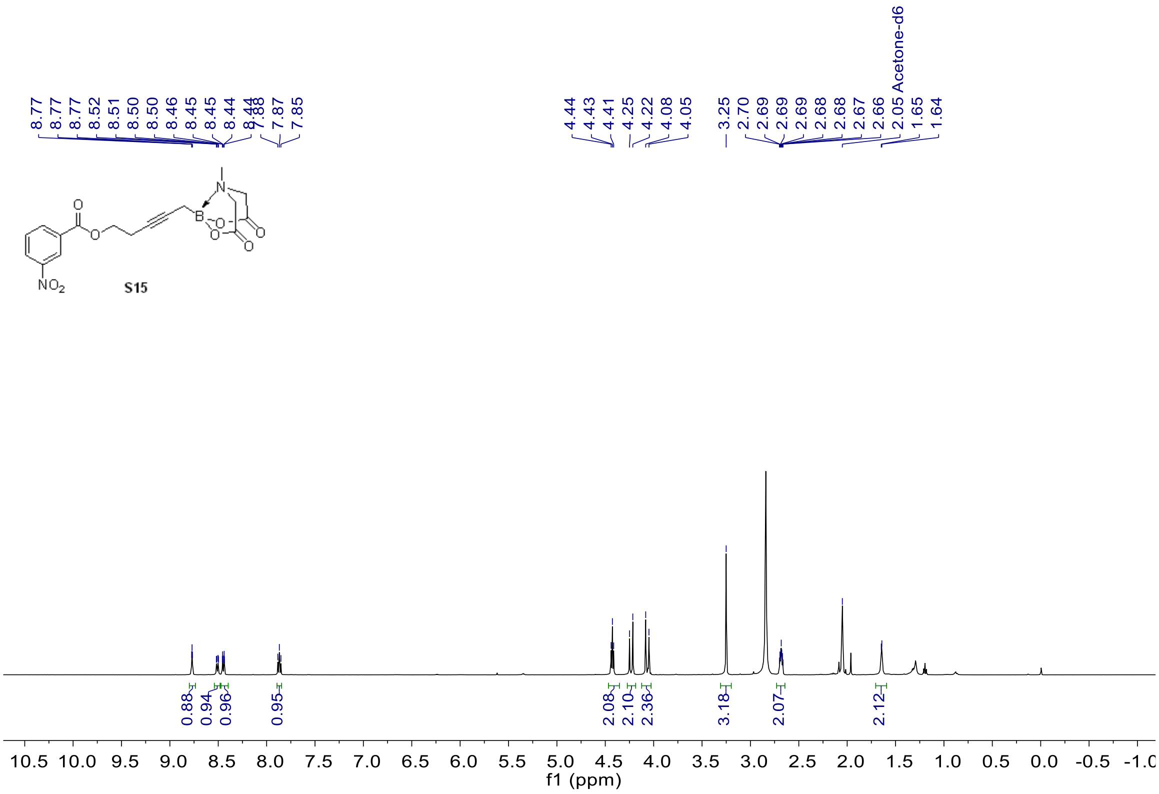

S15

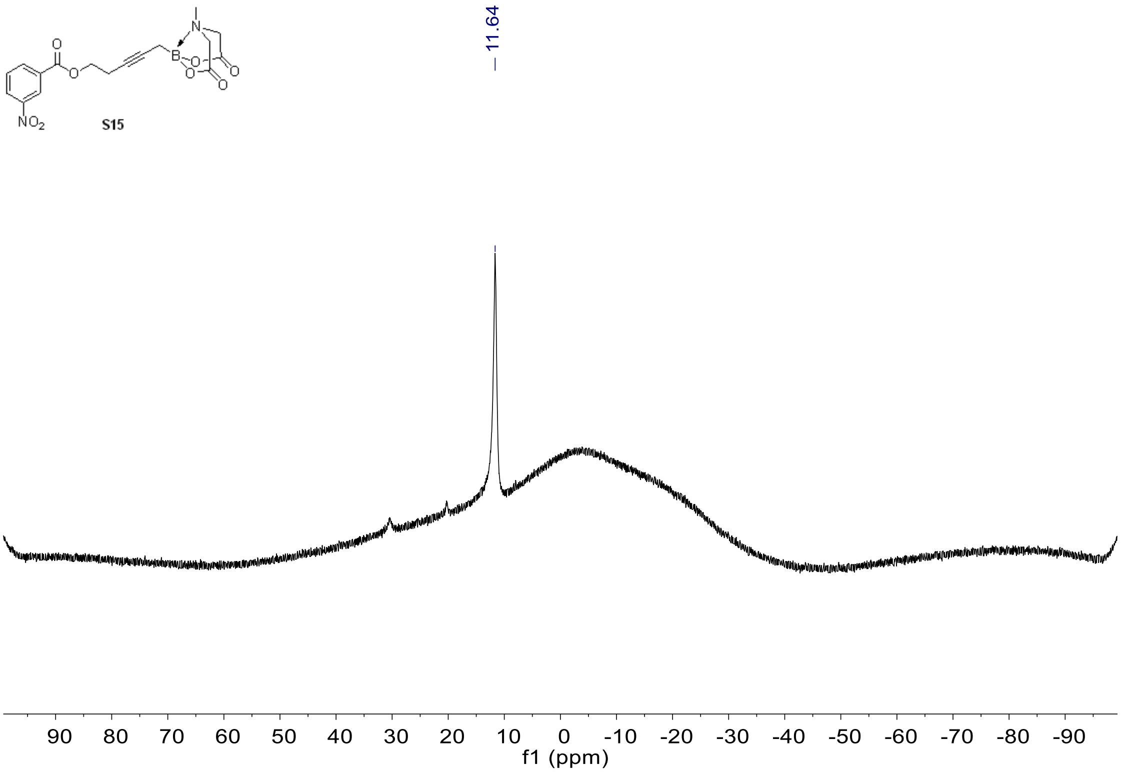

S15

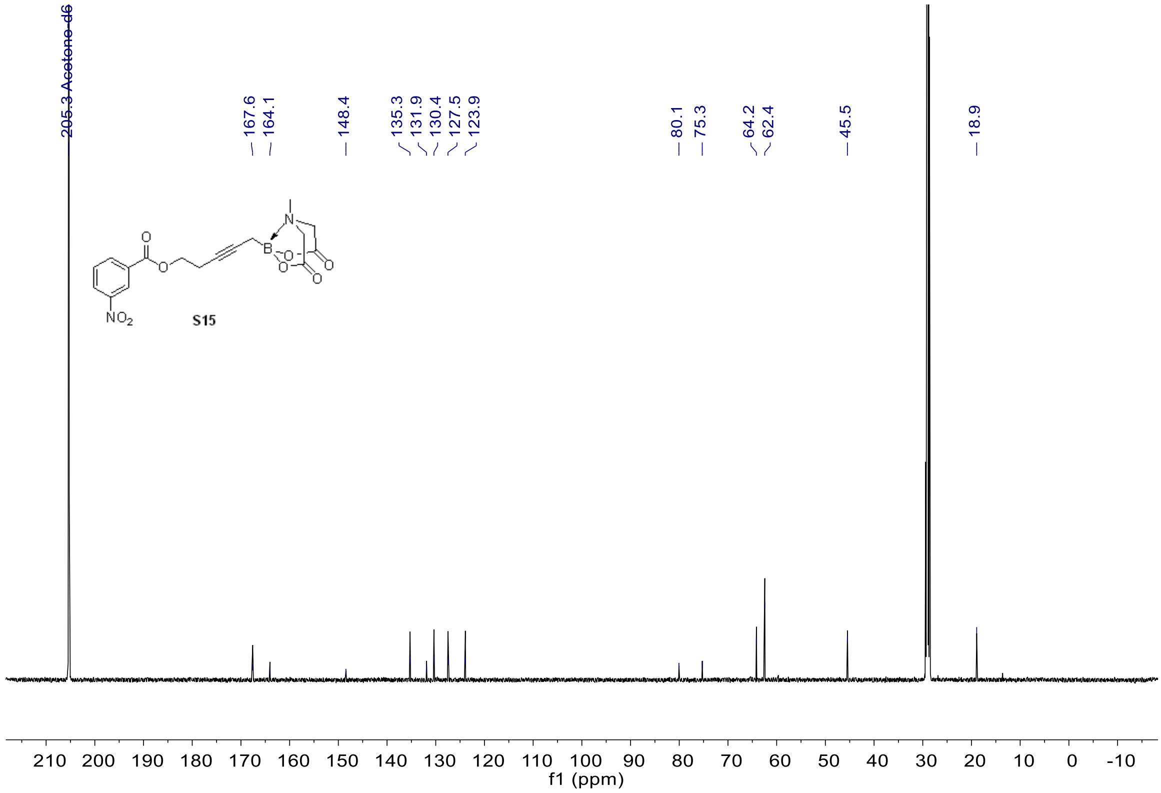
205.3 Acetone-d6
167.6
164.1
148.4
135.3
131.9
130.4
127.5
123.9
80.1
75.3
64.2
62.4
45.5
18.9
S15
210 200 190 180 170 160 150 140 130 120 110 100 90 80 70 60 50 40 30 20 10 0 -10
f1 (ppm)

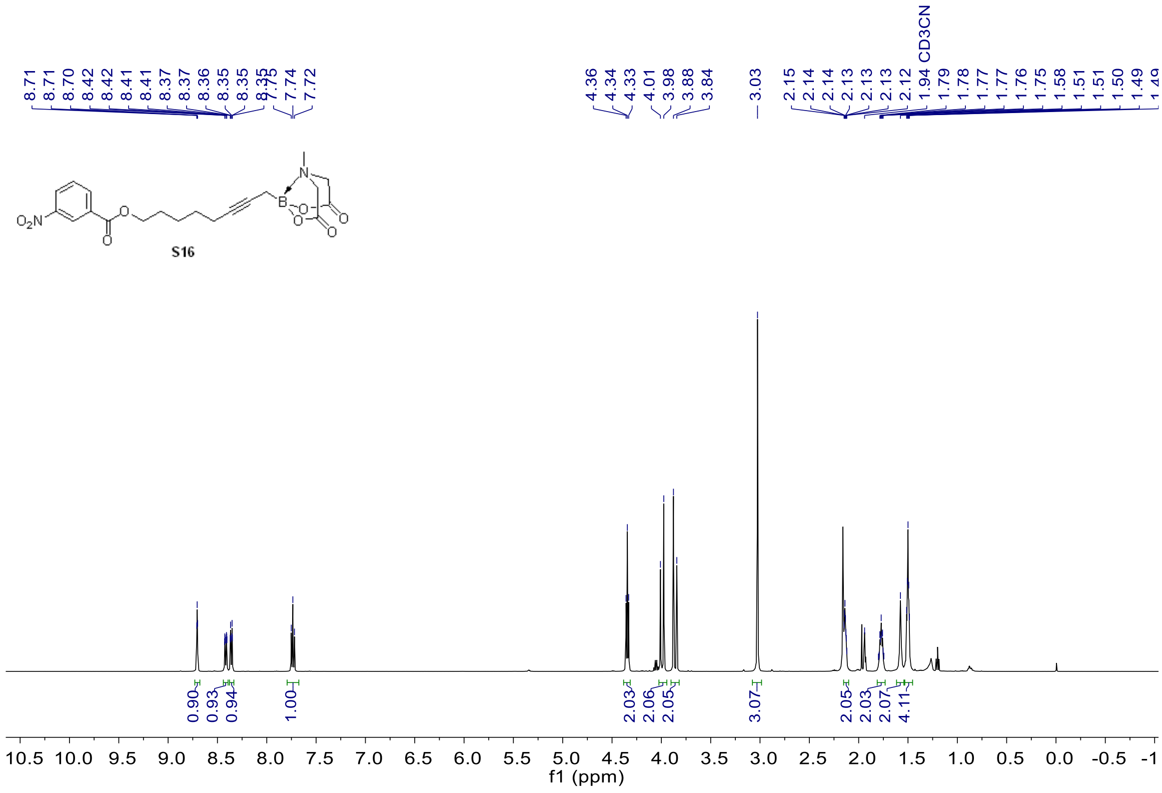
8.71 8.71 8.70 8.42 8.42 8.41 8.37 8.37 8.36 8.35 8.35 7.75 7.74 7.72
4.36 4.34 4.33 4.01 3.98 3.88 3.84
3.03
2.15 2.14 2.14 2.13 2.13 2.13 2.12 1.94 CD3CN 1.79 1.78 1.77 1.77 1.76 1.75 1.58 1.51 1.51 1.50 1.49
S16
0.90 0.93 0.94 1.00 2.03 2.06 2.05 3.07 2.05 2.03 2.07 4.11
10.5 10.0 9.5 9.0 8.5 8.0 7.5 7.0 6.5 6.0 5.5 5.0 4.5 4.0 3.5 3.0 2.5 2.0 1.5 1.0 0.5 0.0 -0.5 -1
f1 (ppm)

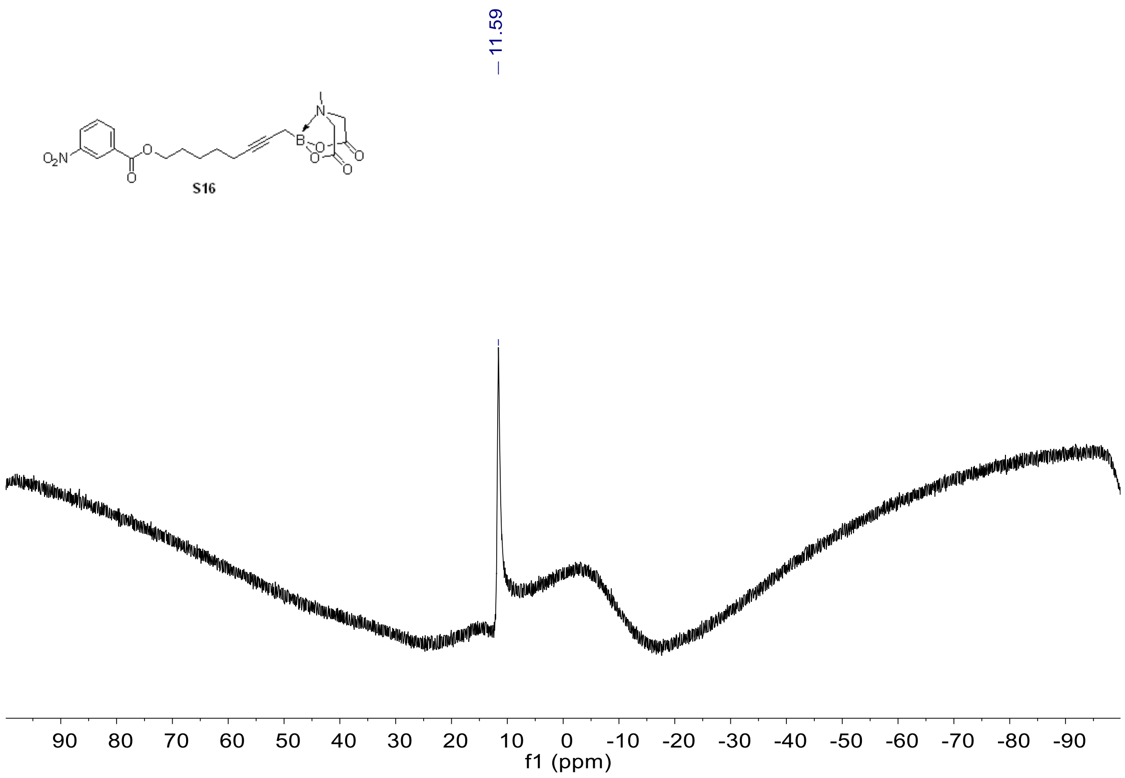

11.59
O2N
O
O
N
B
O
O
O
O
S16
90 80 70 60 50 40 30 20 10 0 -10 -20 -30 -40 -50 -60 -70 -80 -90
f1 (ppm)

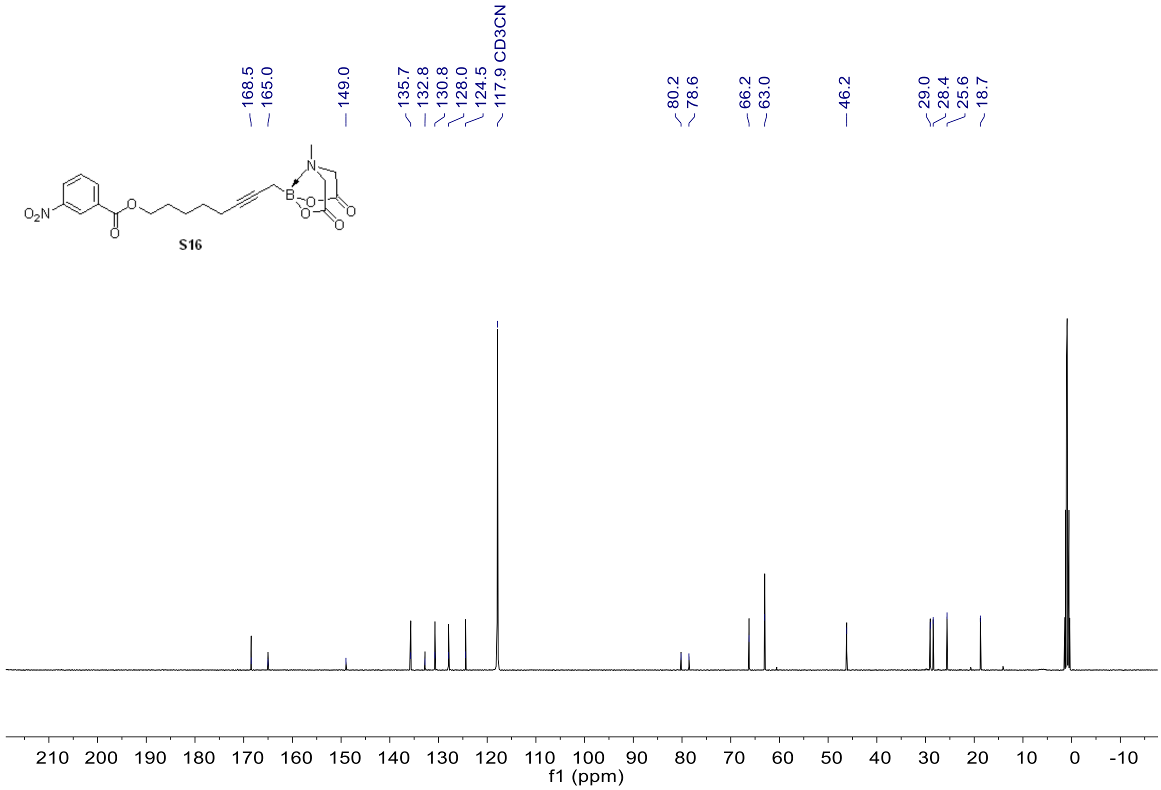

168.5
165.0
149.0
135.7
132.8
130.8
128.0
124.5
117.9 CD3CN
80.2
78.6
66.2
63.0
46.2
29.0
28.4
25.6
18.7
S16
210 200 190 180 170 160 150 140 130 120 110 100 90 80 70 60 50 40 30 20 10 0 -10
f1 (ppm)

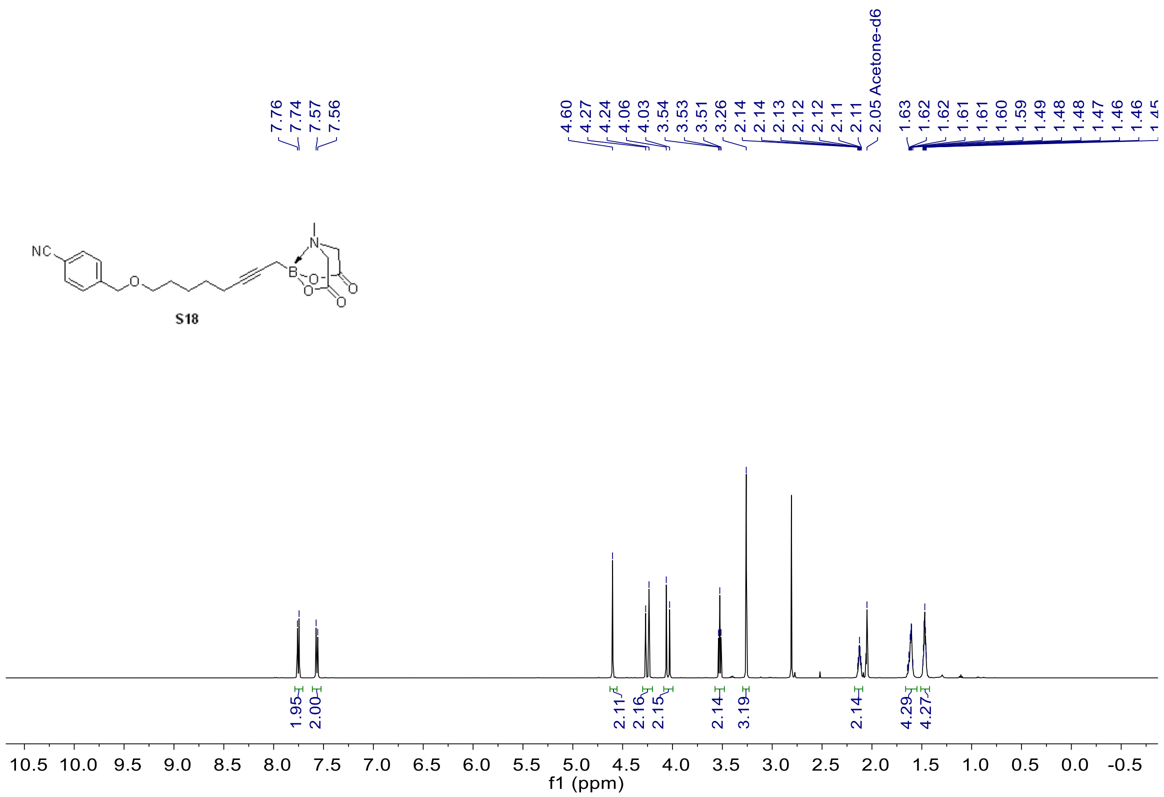
7.76
7.74
7.57
7.56
4.60
4.27
4.24
4.06
4.03
3.54
3.53
3.51
3.26
2.14
2.14
2.13
2.12
2.12
2.11
2.11
2.05 Acetone-d6
1.63
1.62
1.62
1.61
1.61
1.60
1.59
1.49
1.48
1.48
1.47
1.46
1.46
[illegible]
NC
O
N
B
O
O
O
O
S18
1.95
2.00
2.11
2.16
2.15
2.14
3.19
2.14
4.29
4.27
10.5 10.0 9.5 9.0 8.5 8.0 7.5 7.0 6.5 6.0 5.5 5.0 4.5 4.0 3.5 3.0 2.5 2.0 1.5 1.0 0.5 0.0 -0.5
f1 (ppm)

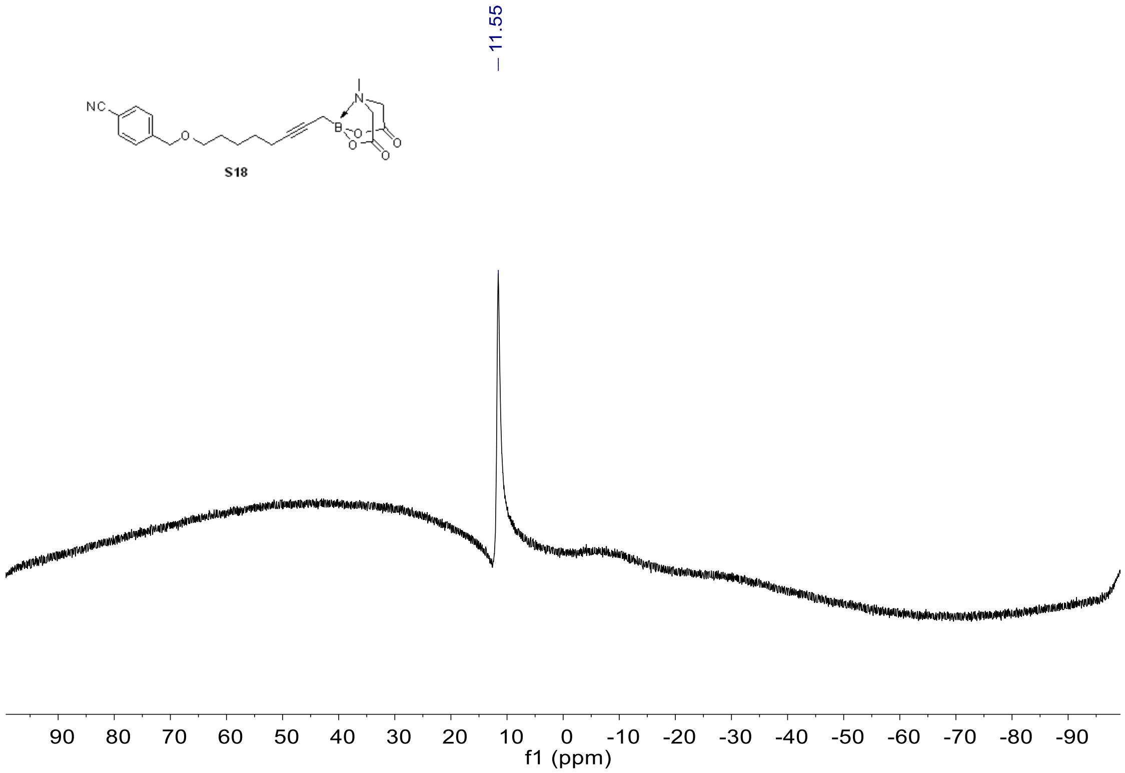
11.55
NC
O
N
B
O
O
O
O
S18
90 80 70 60 50 40 30 20 10 0 -10 -20 -30 -40 -50 -60 -70 -80 -90
f1 (ppm)

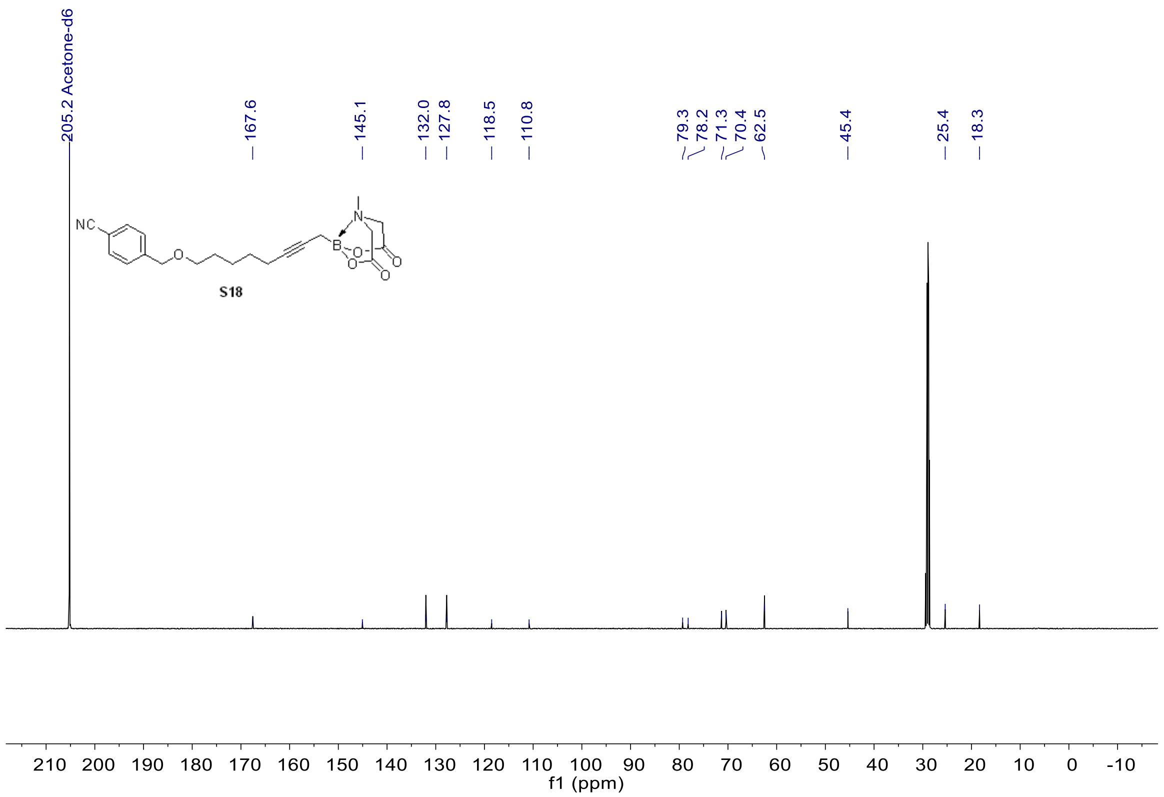
205.2 Acetone-d6
167.6
145.1
132.0
127.8
118.5
110.8
79.3
78.2
71.3
70.4
62.5
45.4
25.4
18.3
NC
O
B
N
O
O
O
O
S18
210 200 190 180 170 160 150 140 130 120 110 100 90 80 70 60 50 40 30 20 10 0 -10
f1 (ppm)

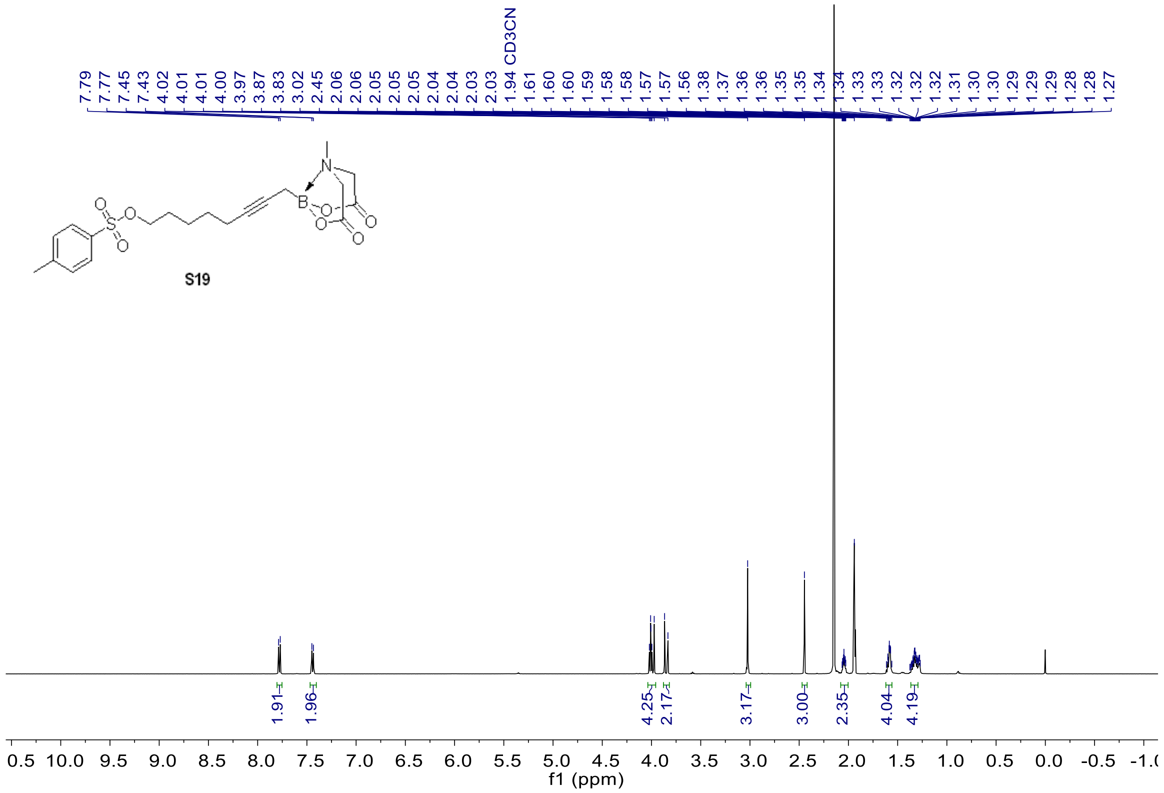
CD3CN
7.79
7.77
7.45
7.43
4.02
4.01
4.00
3.97
3.87
3.83
3.02
2.45
2.06
2.06
2.05
2.05
2.05
2.04
2.04
2.03
1.94
1.61
1.60
1.60
1.59
1.58
1.58
1.57
1.57
1.56
1.38
1.37
1.36
1.36
1.35
1.35
1.34
1.34
1.33
1.33
1.32
1.32
1.32
1.31
1.30
1.30
1.29
1.29
1.29
1.28
1.28
1.27
S19
1.91
1.96
4.25
2.17
3.17
3.00
2.35
4.04
4.19
0.5 10.0 9.5 9.0 8.5 8.0 7.5 7.0 6.5 6.0 5.5 5.0 4.5 4.0 3.5 3.0 2.5 2.0 1.5 1.0 0.5 0.0 -0.5
f1 (ppm)

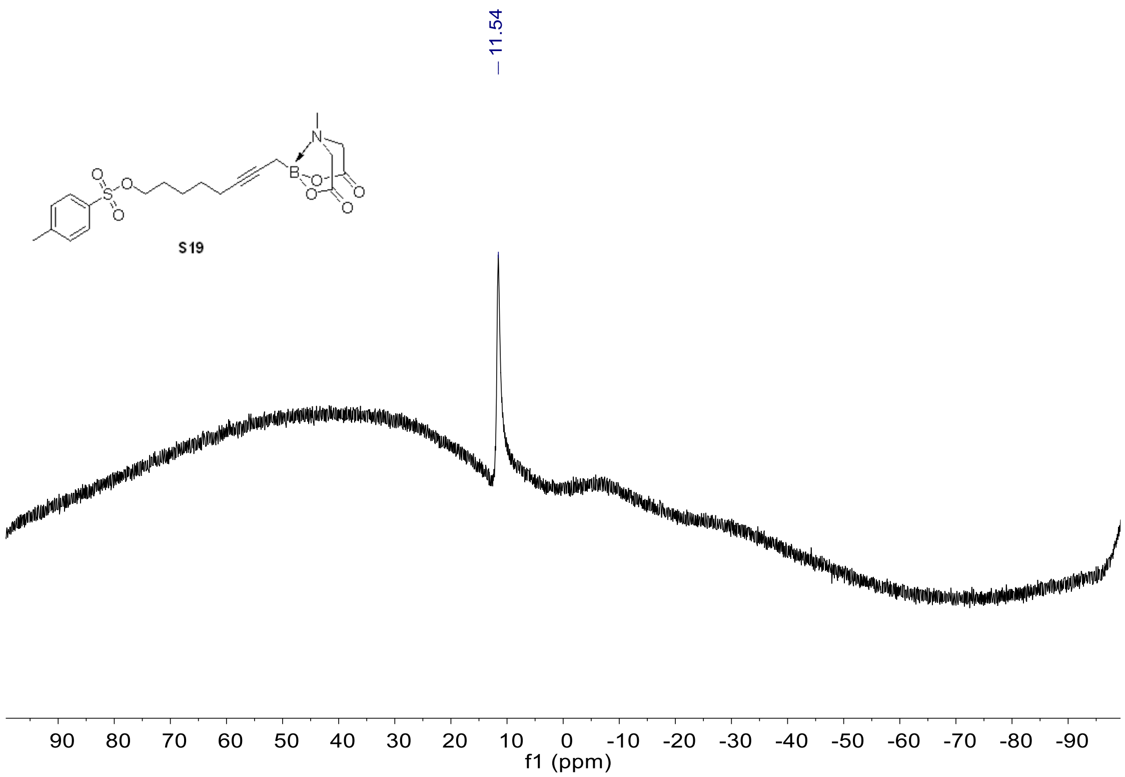
11.54
S19
f1 (ppm)

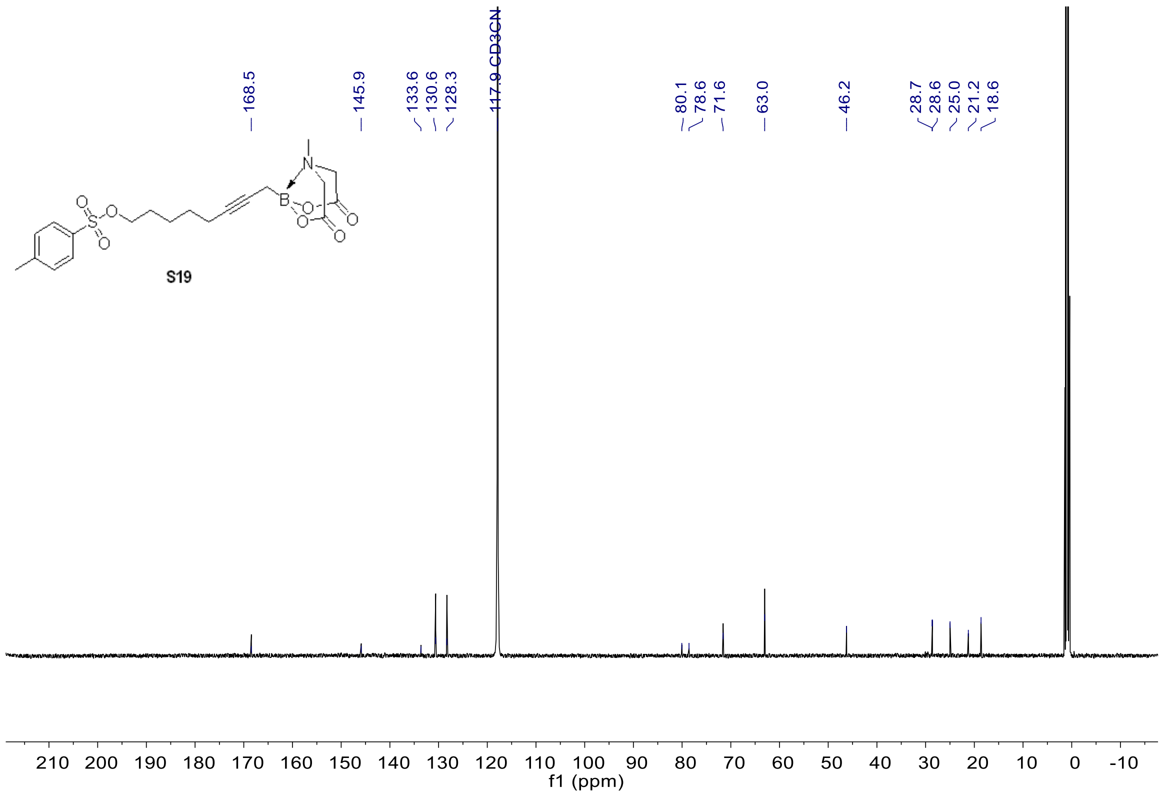
168.5
145.9
133.6
130.6
128.3
117.9 CD3CN
80.1
78.6
71.6
63.0
46.2
28.7
28.6
25.0
21.2
18.6
S19
f1 (ppm)

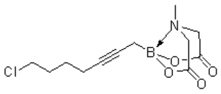

S30

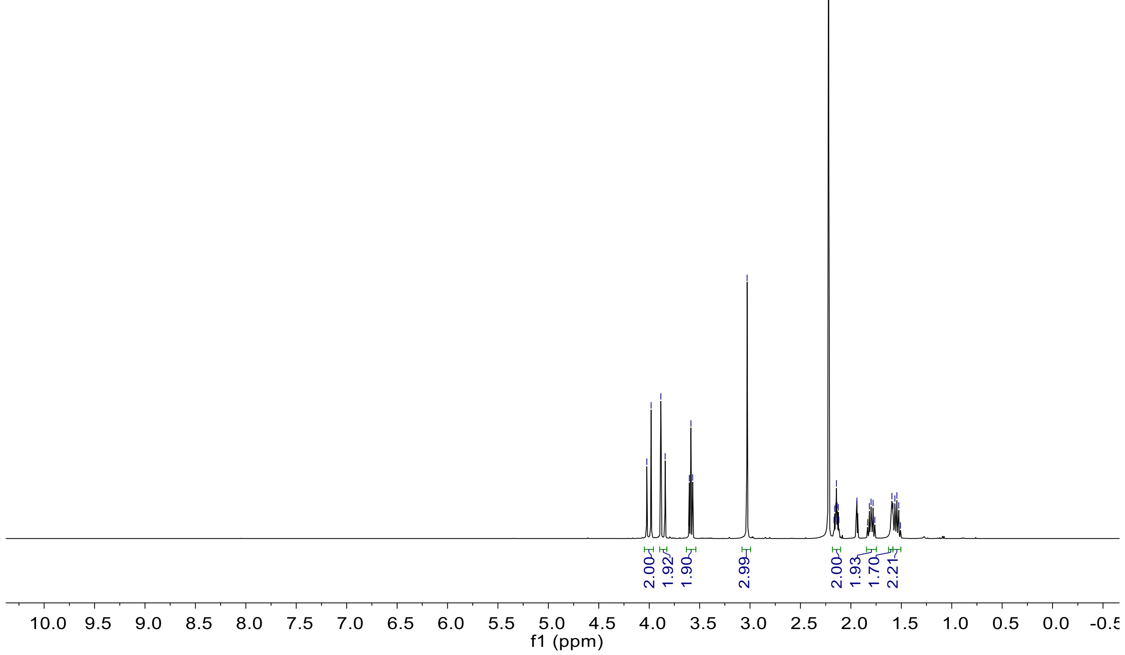

4.02
3.98
3.88
3.84
3.60
3.59
3.57
3.03
2.17
2.16
2.15
2.15
2.14
2.14
2.13
2.13
2.12
1.94 CD3CN
1.82
1.80
1.78
1.76
1.59
1.58
1.56
1.54
2.00
1.92
1.90
2.99
2.00
1.93
1.70
2.21
10.0 9.5 9.0 8.5 8.0 7.5 7.0 6.5 6.0 5.5 5.0 4.5 4.0 3.5 3.0 2.5 2.0 1.5 1.0 0.5 0.0
f1 (ppm)

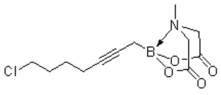

S30

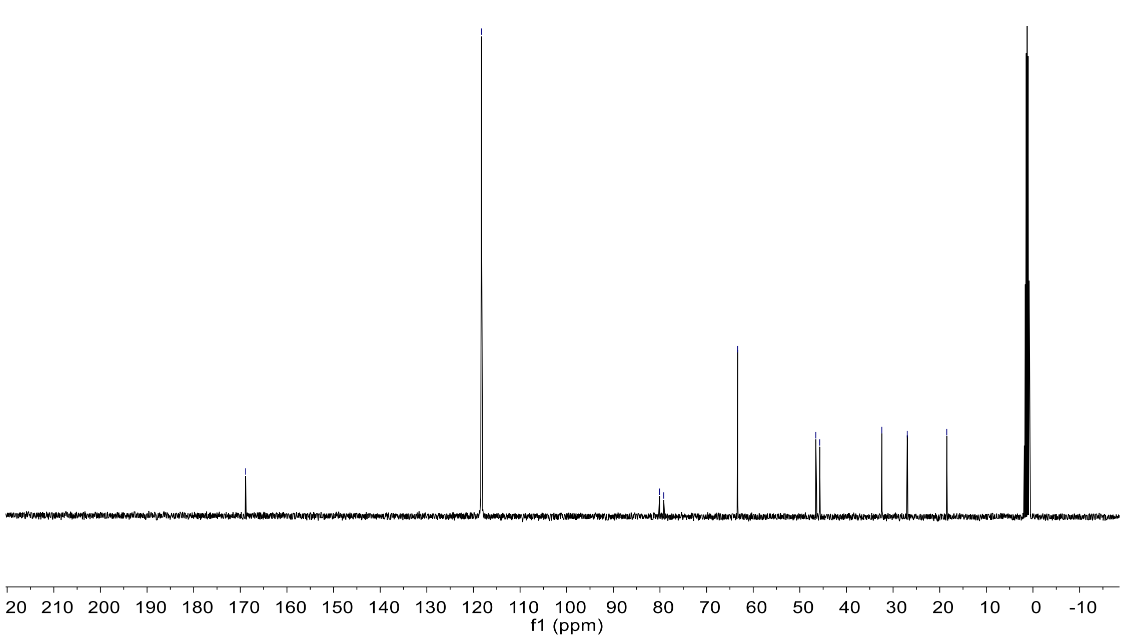

168.85
118.26 CD3CN
80.09
79.19
63.34
46.57
45.74
32.41
26.97
18.48
210 200 190 180 170 160 150 140 130 120 110 100 90 80 70 60 50 40 30 20 10 0 -10
f1 (ppm)

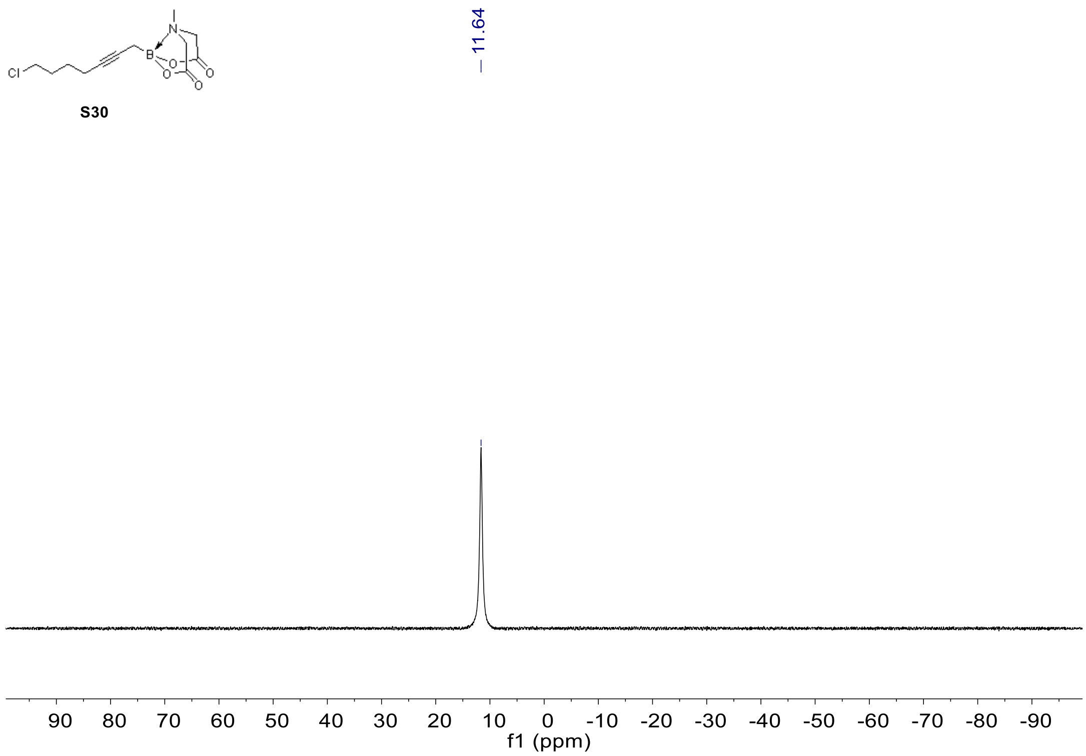

Cl
B
N
O
S30
11.64
90
80
70
60
50
40
30
20
10
0
-10
-20
-30
-40
-50
-60
-70
-80
-90
f1 (ppm)

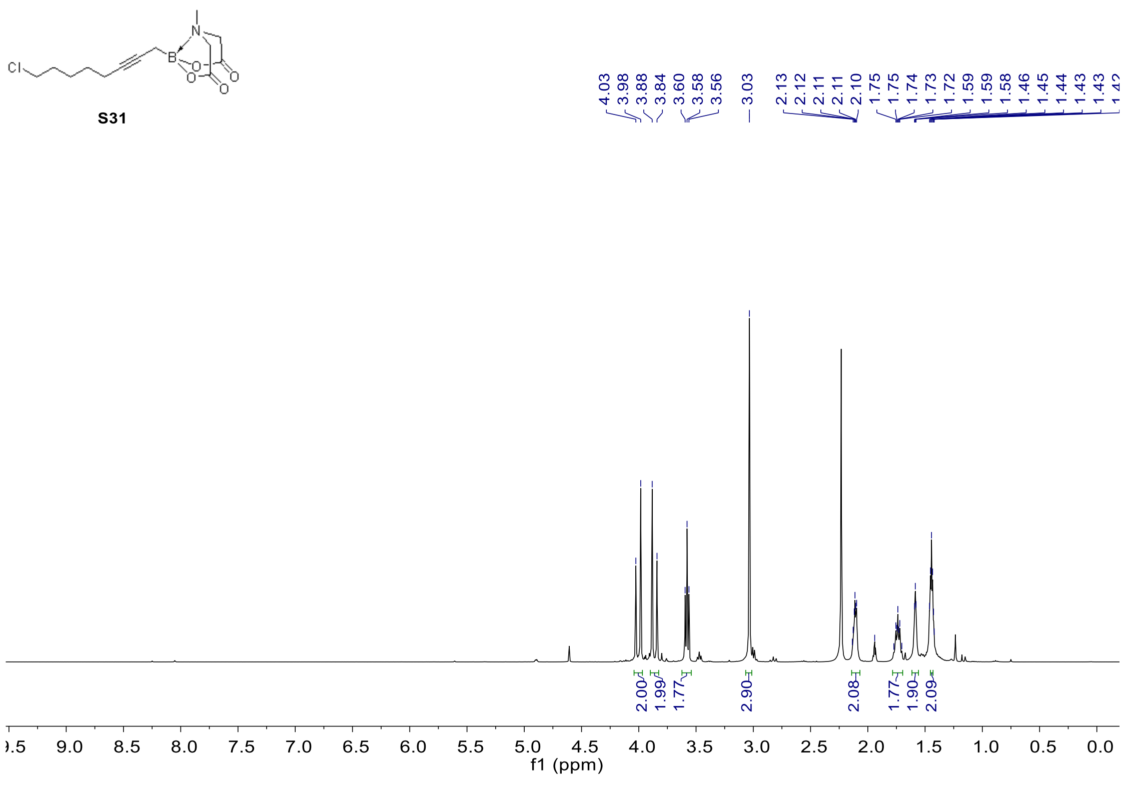

Cl
B
N
O
S31
4.03
3.98
3.88
3.84
3.60
3.58
3.56
3.03
2.13
2.12
2.11
2.11
2.10
1.75
1.75
1.74
1.73
1.72
1.59
1.58
1.46
1.45
1.44
1.43
1.43
2.00
1.99
1.77
2.90
2.08
1.77
1.90
2.09
9.0
8.5
8.0
7.5
7.0
6.5
6.0
5.5
5.0
4.5
4.0
3.5
3.0
2.5
2.0
1.5
1.0
0.5
0.0
f1 (ppm)

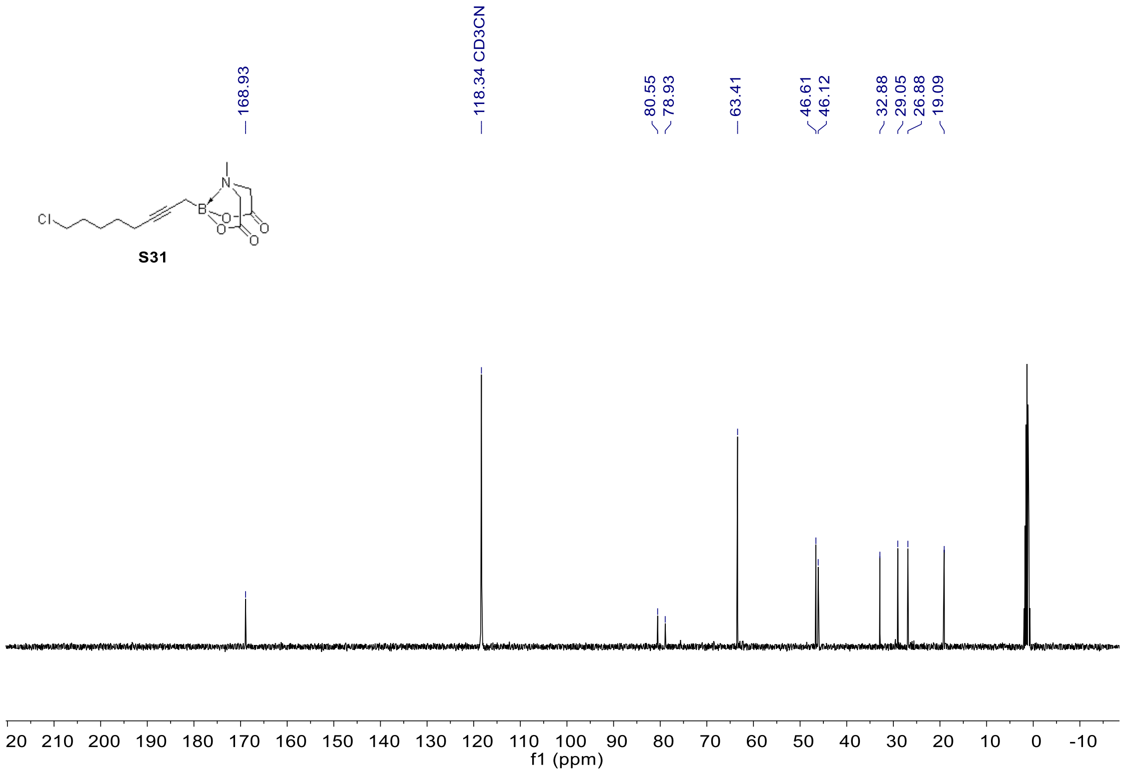
168.93
118.34 CD3CN
80.55
78.93
63.41
46.61
46.12
32.88
29.05
26.88
19.09
S31
20 210 200 190 180 170 160 150 140 130 120 110 100 90 80 70 60 50 40 30 20 10 0 -10
f1 (ppm)

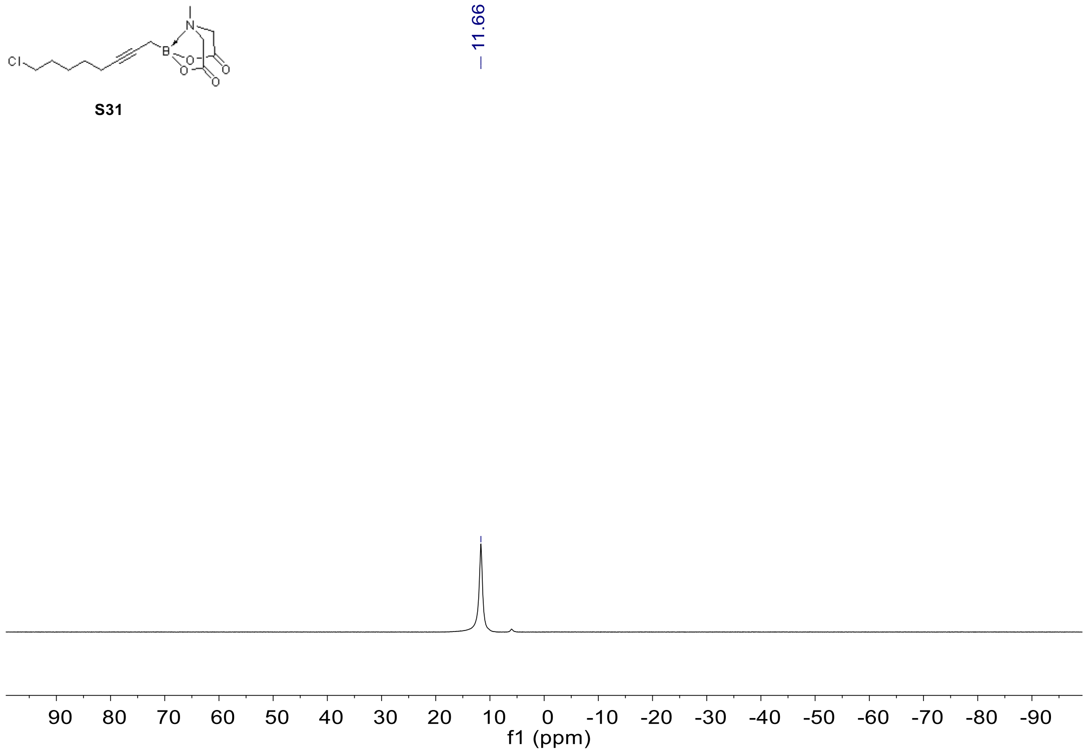
11.66
S31
90 80 70 60 50 40 30 20 10 0 -10 -20 -30 -40 -50 -60 -70 -80 -90
f1 (ppm)

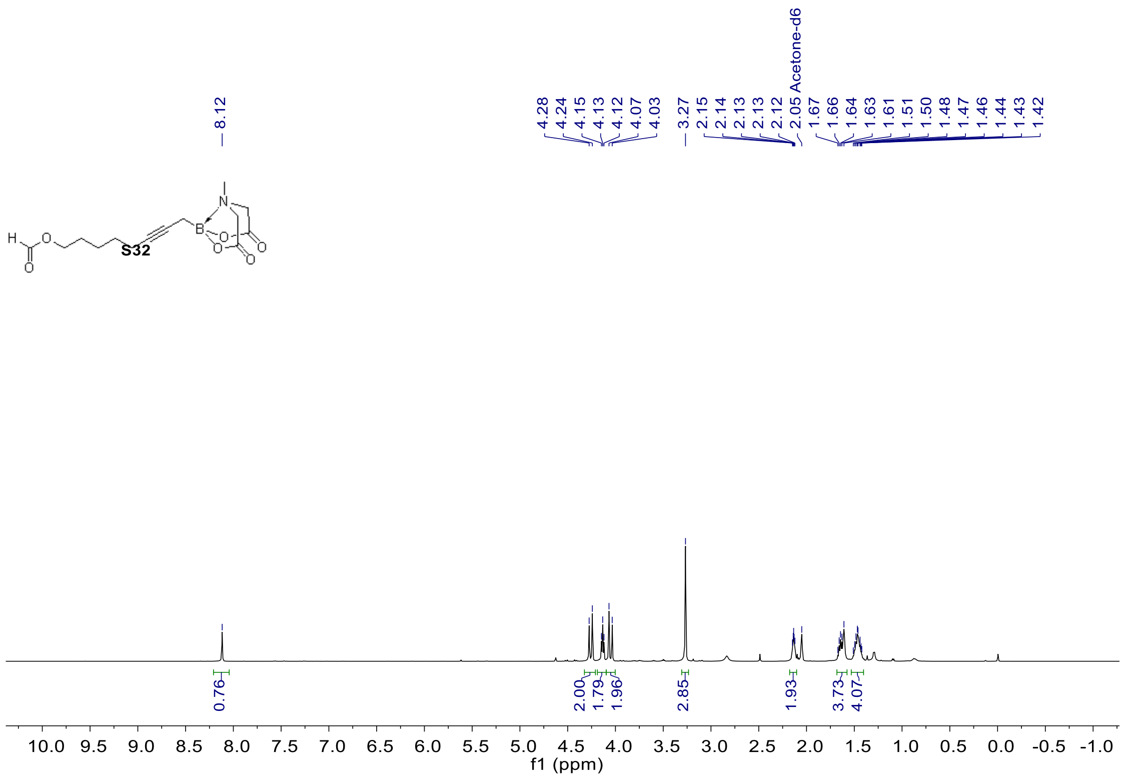

S32
8.12
4.28
4.24
4.15
4.13
4.12
4.07
4.03
3.27
2.15
2.14
2.13
2.13
2.12
2.05 Acetone-d6
1.67
1.66
1.64
1.63
1.61
1.51
1.50
1.48
1.47
1.46
1.44
1.43
1.42
0.76
2.00
1.79
1.96
2.85
1.93
3.73
4.07
f1 (ppm)

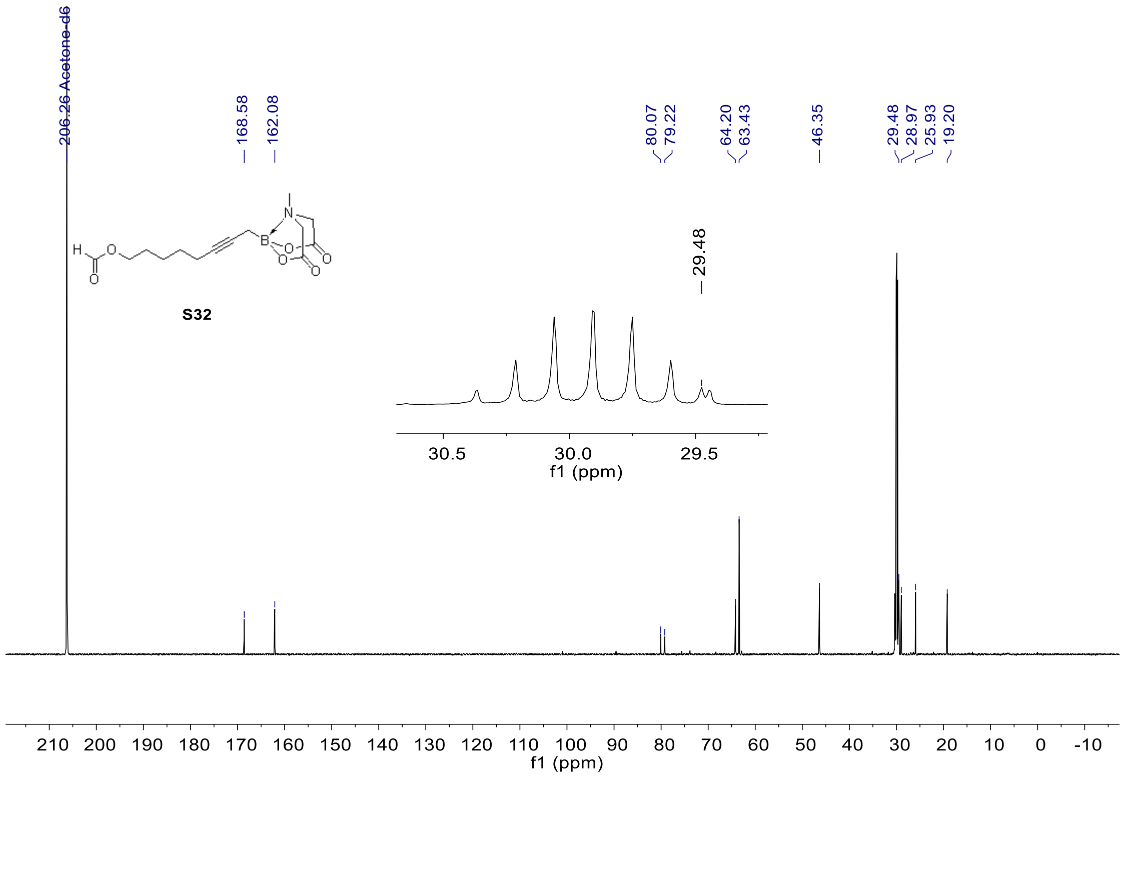

206.26 Acetone-d6
168.58
162.08
80.07
79.22
64.20
63.43
46.35
29.48
28.97
25.93
19.20
S32
f1 (ppm)

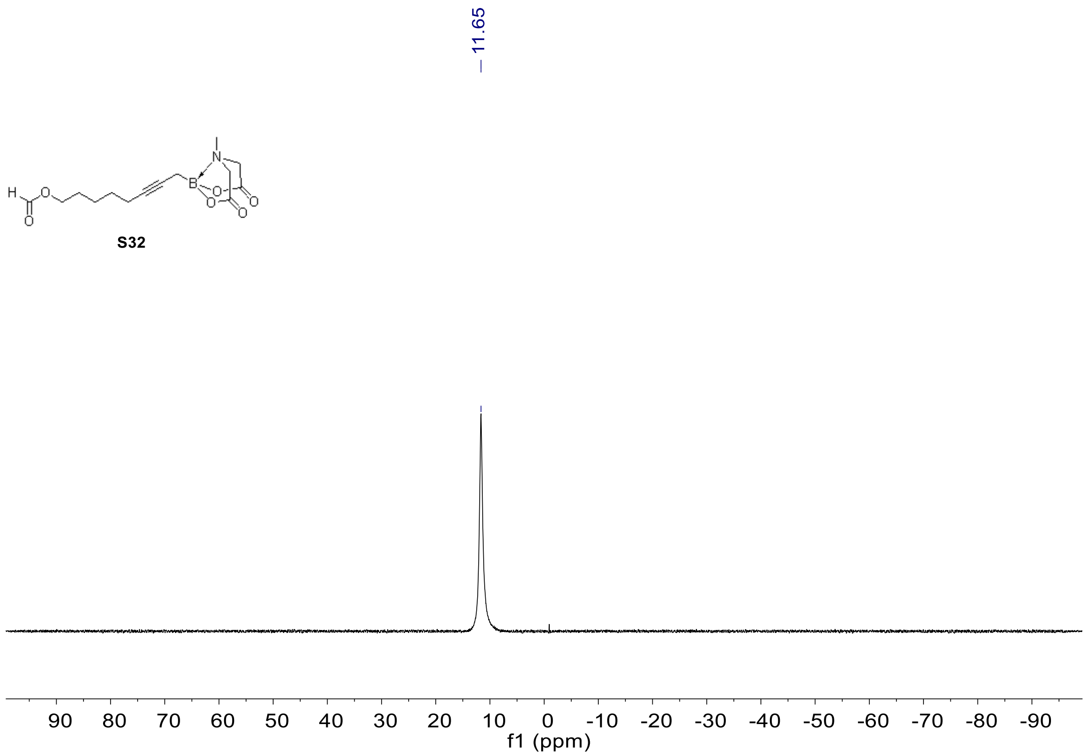
11.65
f1 (ppm)

S32

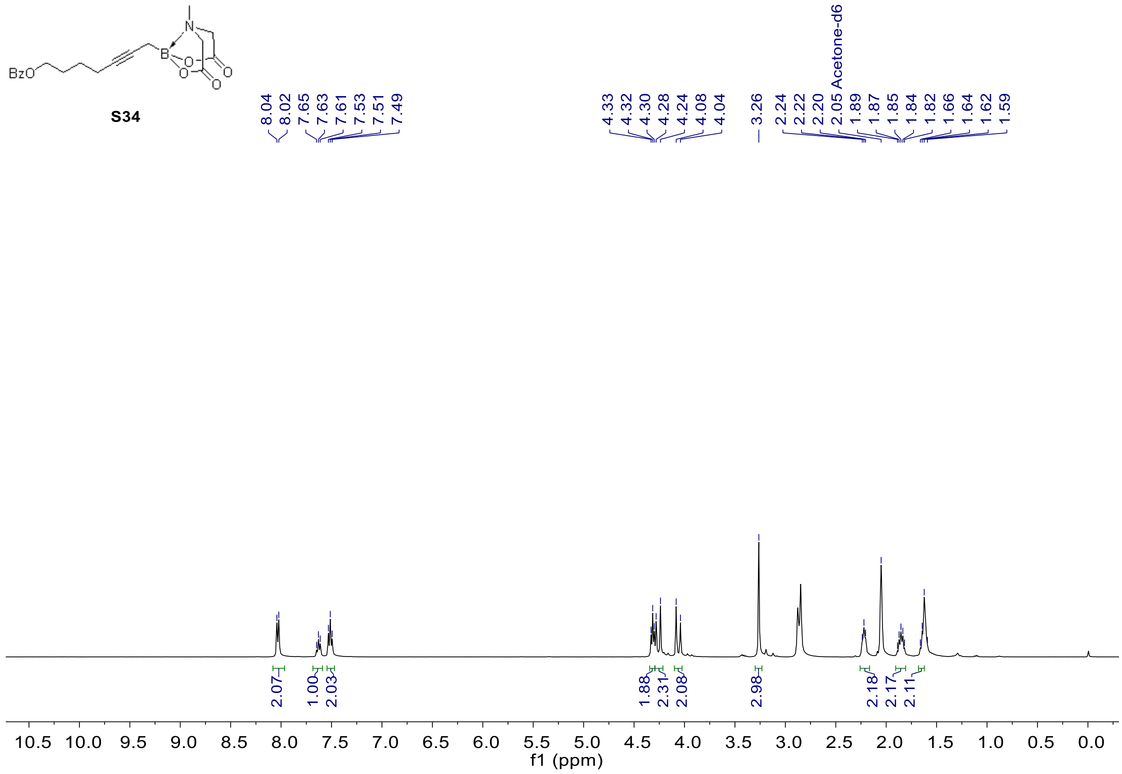
BzO
8.04 8.02 7.65 7.63 7.61 7.53 7.51 7.49
4.33 4.32 4.30 4.28 4.24 4.08 4.04
3.26
2.24 2.22 2.20 2.05 Acetone-d6 1.89 1.87 1.85 1.84 1.82 1.66 1.64 1.62 1.59
2.07 1.00 2.03 1.88 2.31 2.08 2.98 2.18 2.17 2.11
f1 (ppm)

S34

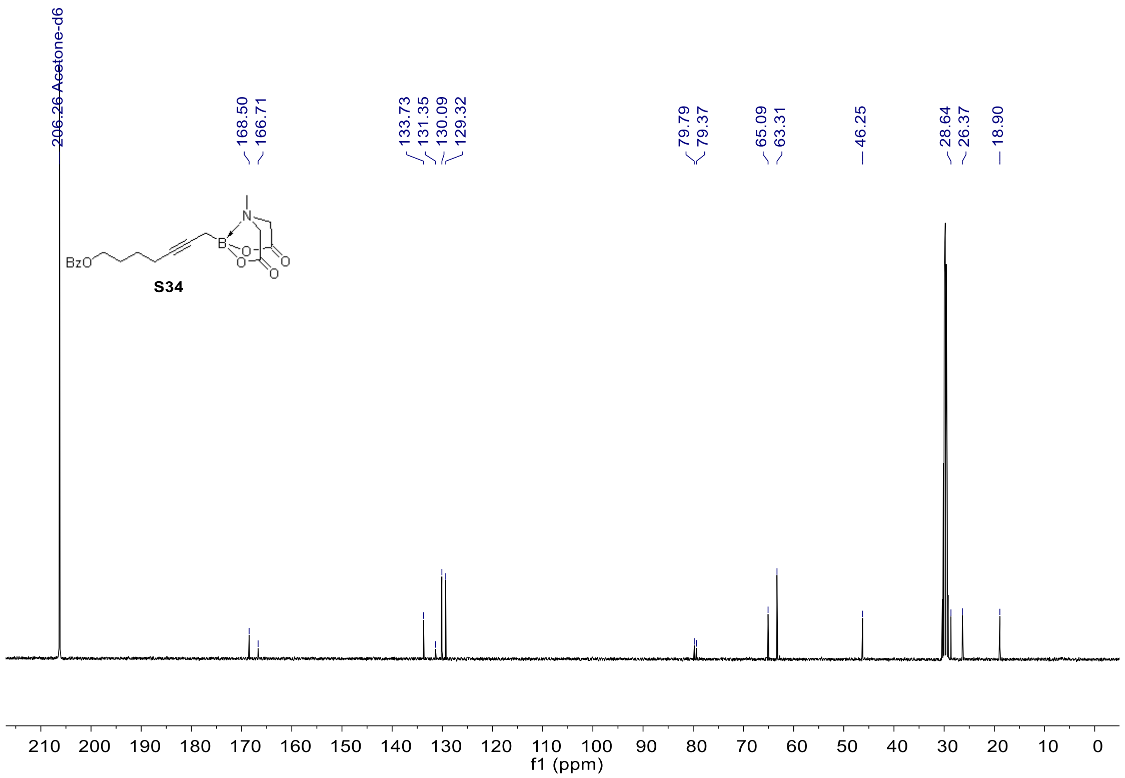
206.26 Acetone-d6
168.50
166.71
133.73
131.35
130.09
129.32
79.79
79.37
65.09
63.31
46.25
28.64
26.37
18.90
BzO
B
N
O
S34
210 200 190 180 170 160 150 140 130 120 110 100 90 80 70 60 50 40 30 20 10 0
f1 (ppm)

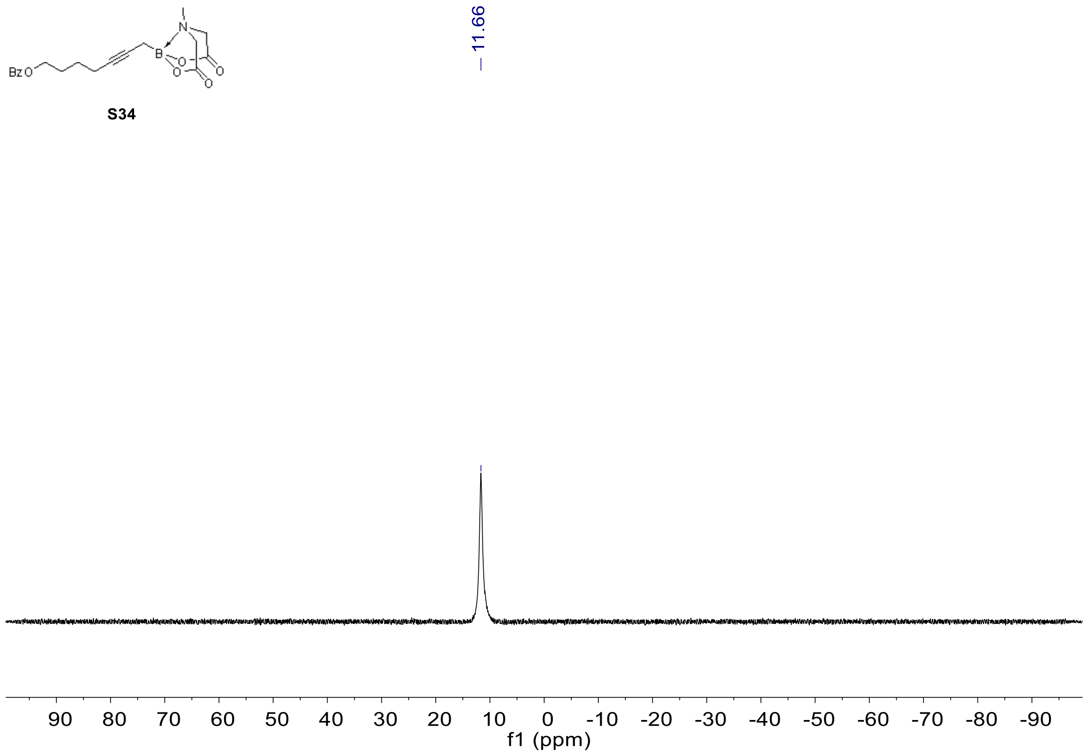
11.66
BzO
B
N
O
S34
90 80 70 60 50 40 30 20 10 0 -10 -20 -30 -40 -50 -60 -70 -80 -90
f1 (ppm)

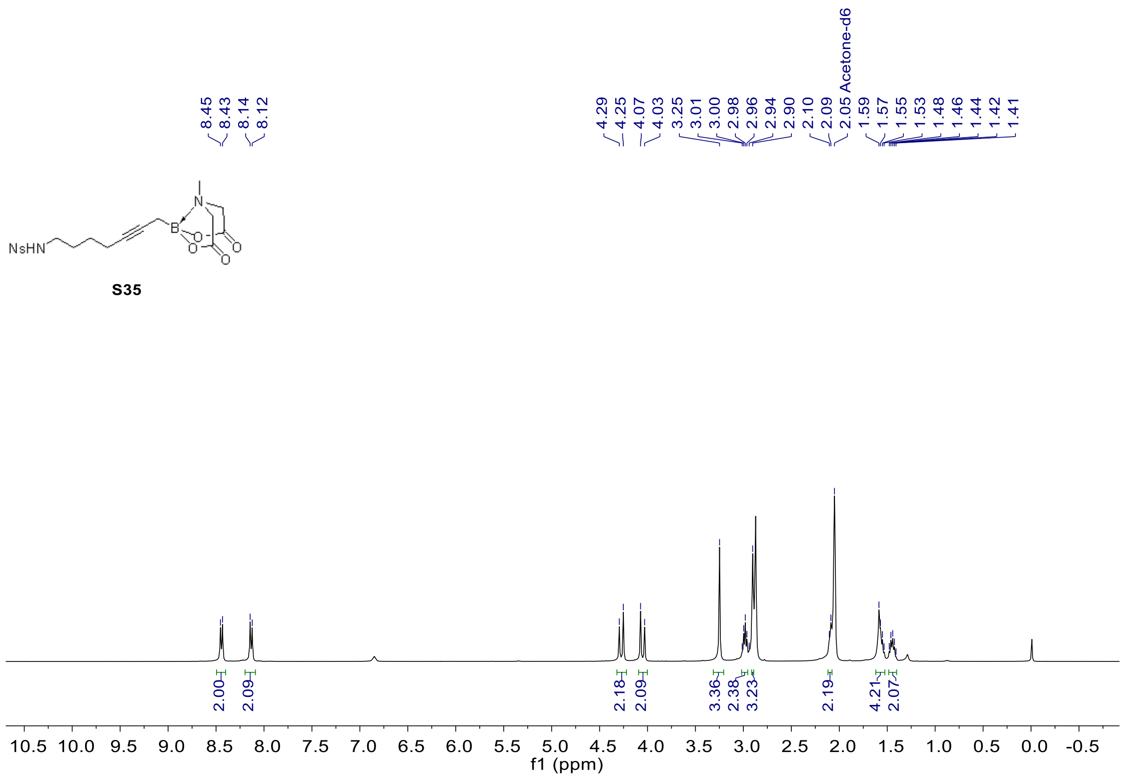
8.45
8.43
8.14
8.12
4.29
4.25
4.07
4.03
3.25
3.01
3.00
2.98
2.96
2.94
2.90
2.10
2.09
2.05 Acetone-d6
1.59
1.57
1.55
1.53
1.48
1.46
1.44
1.42
1.41
NsHN
N
B
O
S35
2.00
2.09
2.18
2.09
3.36
2.38
3.23
2.19
4.21
2.07
10.5 10.0 9.5 9.0 8.5 8.0 7.5 7.0 6.5 6.0 5.5 5.0 4.5 4.0 3.5 3.0 2.5 2.0 1.5 1.0 0.5 0.0 -0.5
f1 (ppm)

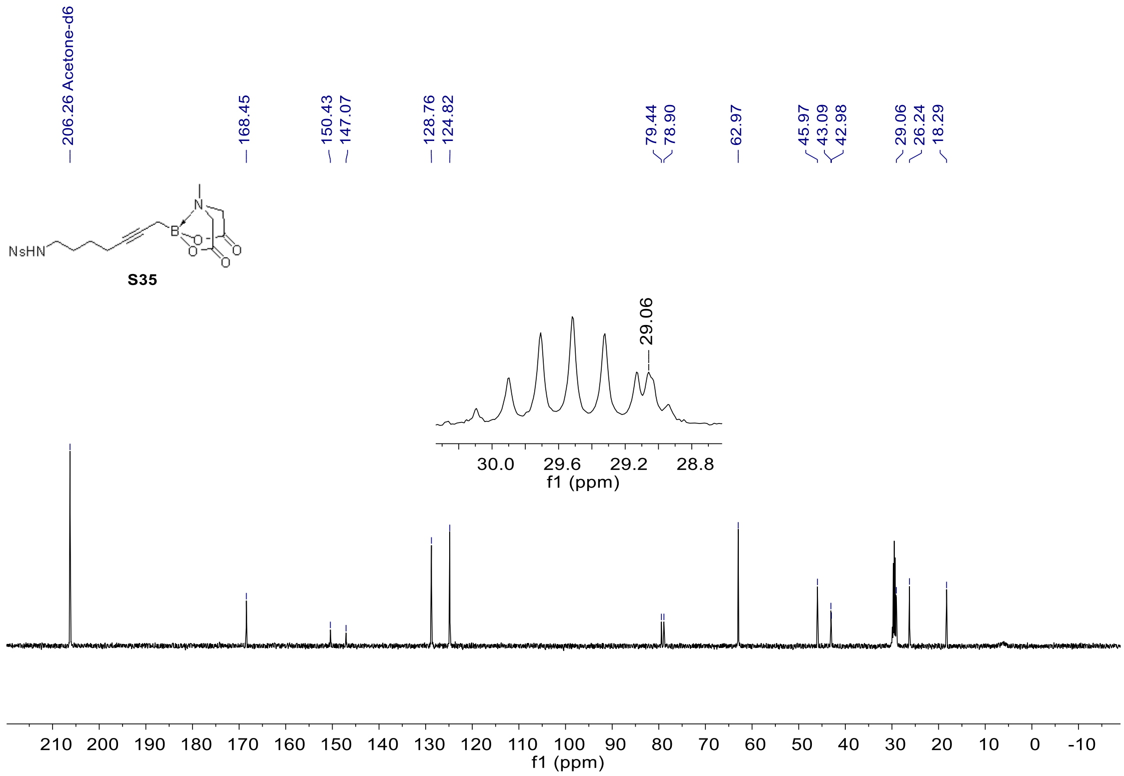
206.26 Acetone-d6
168.45
150.43
147.07
128.76
124.82
79.44
78.90
62.97
45.97
43.09
42.98
29.06
26.24
18.29
NsHN
N
B
O
S35
29.06
30.0 29.6 29.2 28.8
f1 (ppm)
210 200 190 180 170 160 150 140 130 120 110 100 90 80 70 60 50 40 30 20 10 0 -10
f1 (ppm)

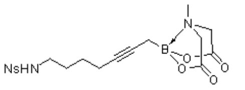
NsHN
B
N
O
O
O
O

S35

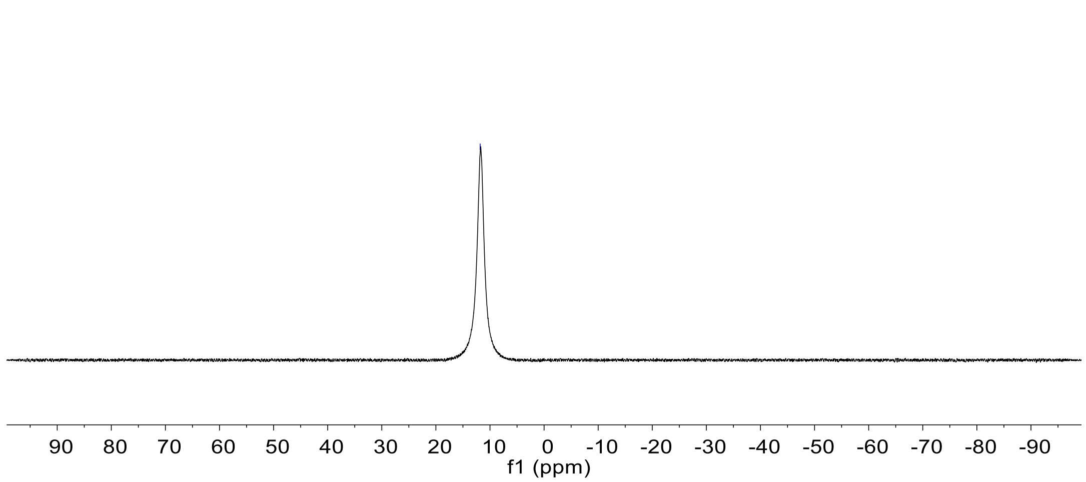
11.82
90 80 70 60 50 40 30 20 10 0 -10 -20 -30 -40 -50 -60 -70 -80 -90
f1 (ppm)

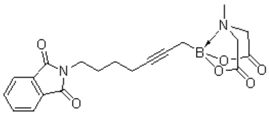
O
N
O
B
N
O
O
O
O

S36

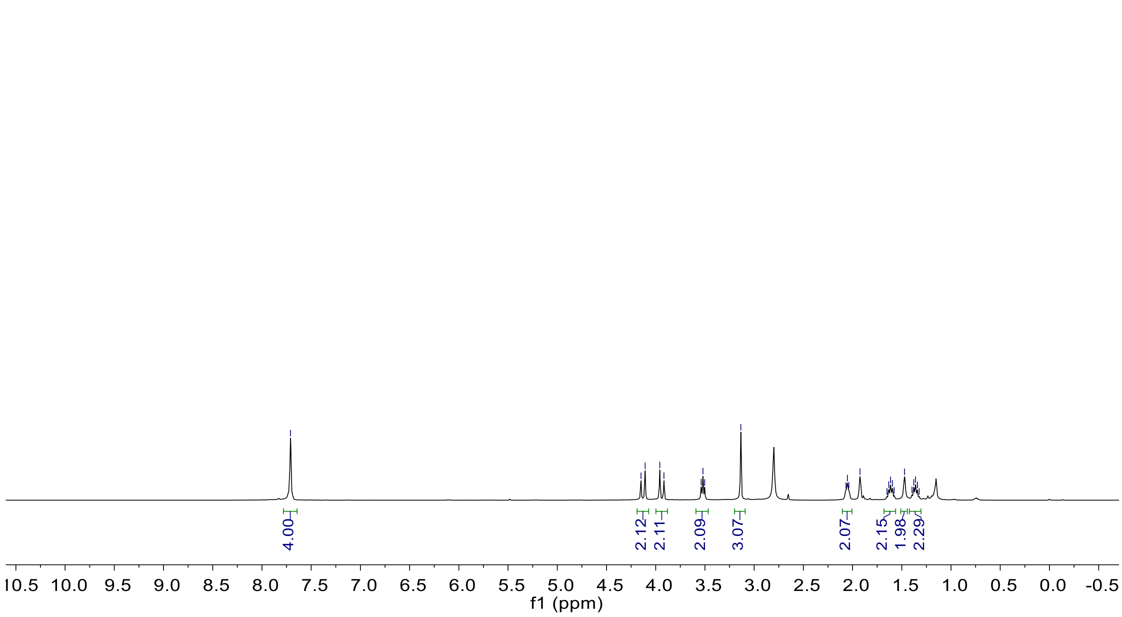
7.71
4.15 4.11 3.96 3.92 3.54 3.52 3.50 3.14 2.07 2.05 2.04 1.92 Acetone-d6 1.65 1.63 1.61 1.60 1.58 1.47 1.40 1.38 1.36 1.34 1.32
4.00
2.12 2.11 2.09 3.07 2.07 2.15 1.98 2.29
10.5 10.0 9.5 9.0 8.5 8.0 7.5 7.0 6.5 6.0 5.5 5.0 4.5 4.0 3.5 3.0 2.5 2.0 1.5 1.0 0.5 0.0 -0.5
f1 (ppm)

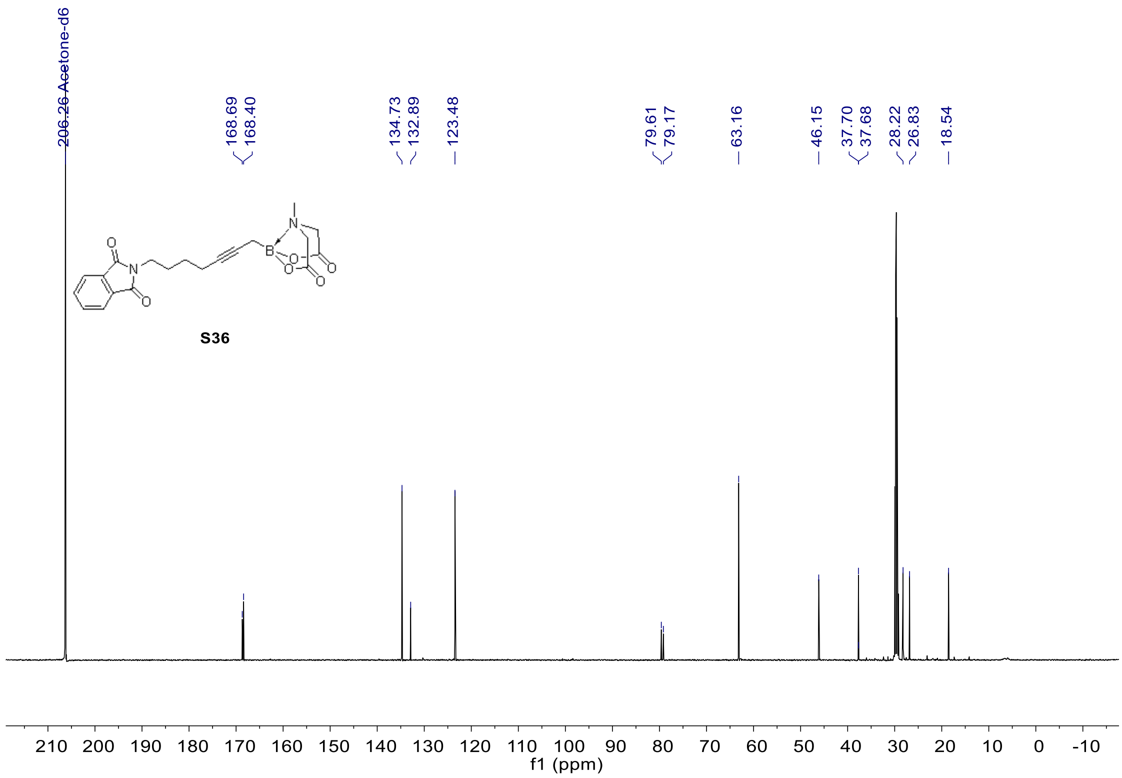
206.26 Acetone-d6
168.69
168.40
134.73
132.89
123.48
79.61
79.17
63.16
46.15
37.70
37.68
28.22
26.83
18.54
S36
210 200 190 180 170 160 150 140 130 120 110 100 90 80 70 60 50 40 30 20 10 0 -10
f1 (ppm)

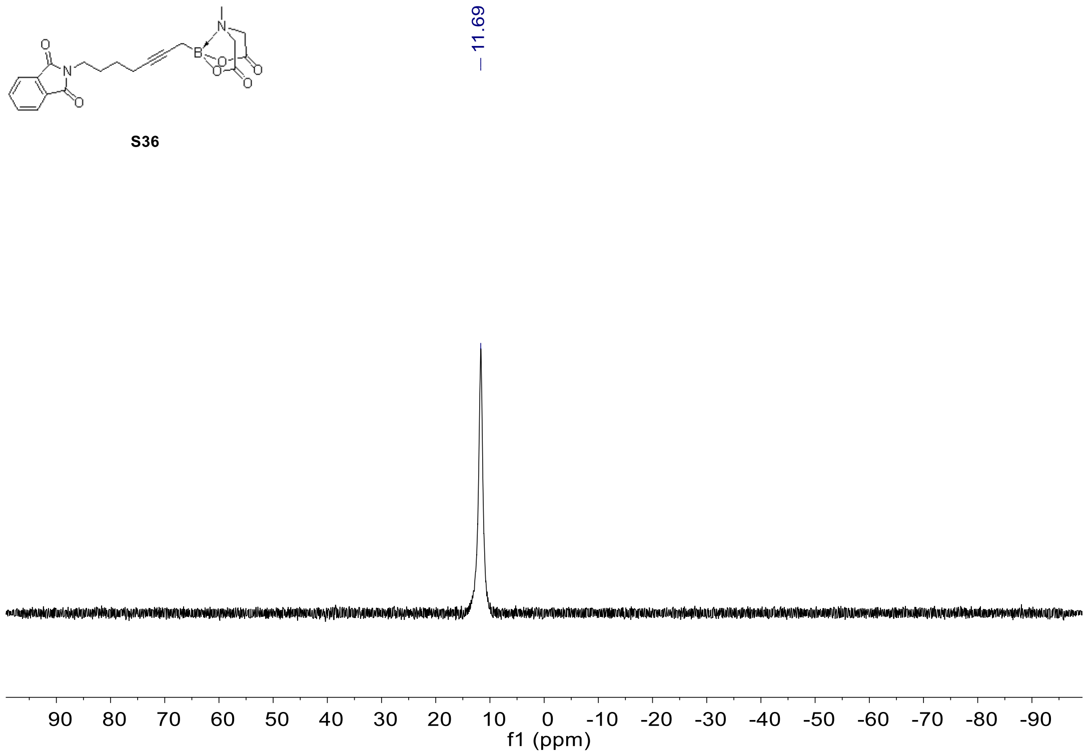
11.69
S36
90 80 70 60 50 40 30 20 10 0 -10 -20 -30 -40 -50 -60 -70 -80 -90
f1 (ppm)

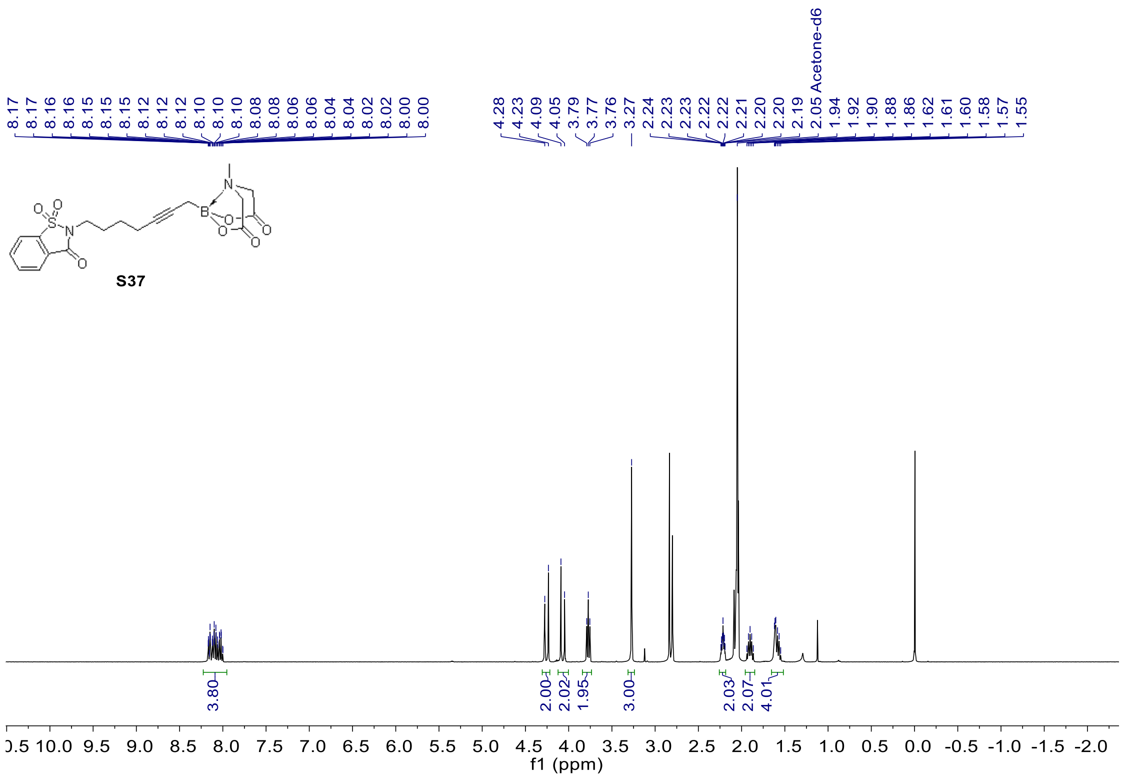
8.17 8.17 8.16 8.16 8.15 8.15 8.12 8.12 8.12 8.10 8.10 8.08 8.08 8.06 8.04 8.04 8.02 8.02 8.00 8.00
4.28 4.23 4.09 4.05 3.79 3.77 3.76 3.27 2.24 2.23 2.23 2.22 2.22 2.21 2.20 2.20 2.19 2.05 Acetone-d6 1.94 1.92 1.90 1.88 1.86 1.62 1.61 1.60 1.58 1.57 1.55
S37
3.80
2.00 2.02 1.95 3.00 2.03 2.07 4.01
f1 (ppm)

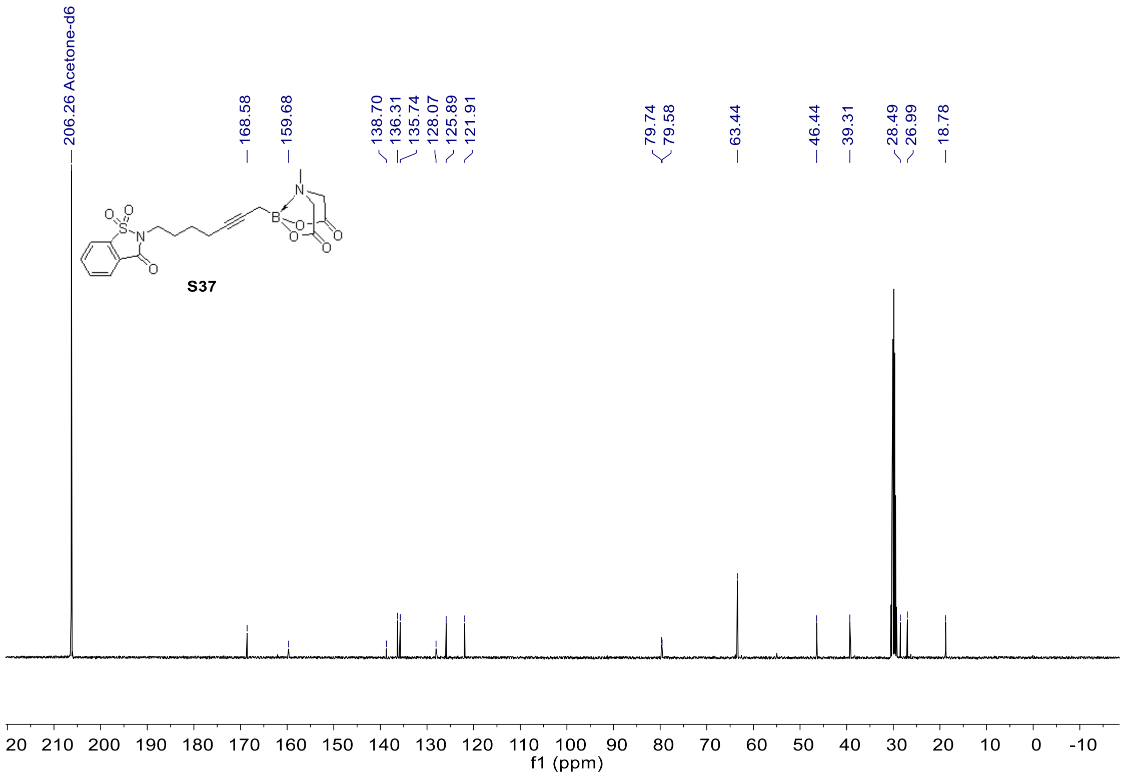
206.26 Acetone-d6
168.58 159.68 138.70 136.31 135.74 128.07 125.89 121.91 79.74 79.58 63.44 46.44 39.31 28.49 26.99 18.78
S37
f1 (ppm)

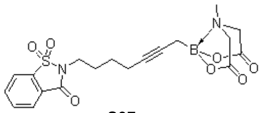

S37

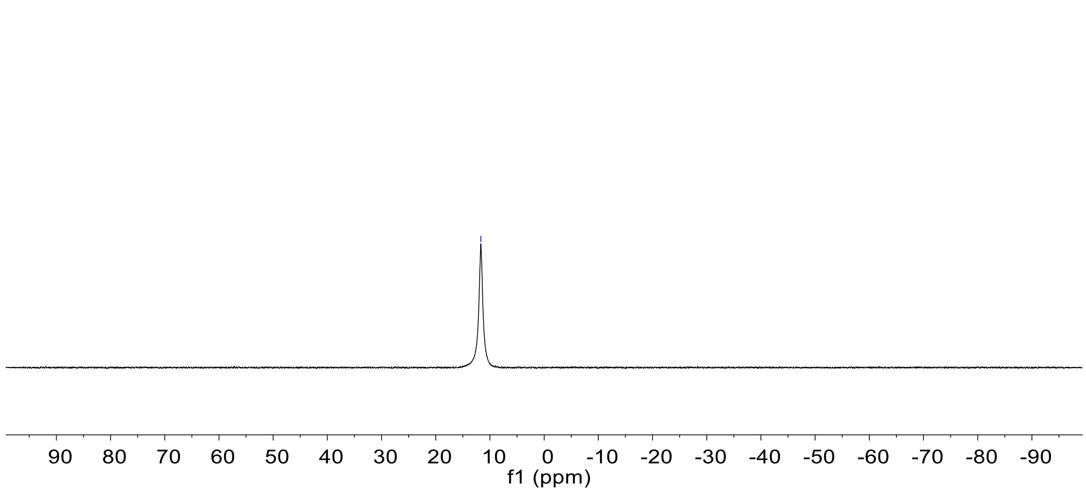

11.67
90 80 70 60 50 40 30 20 10 0 -10 -20 -30 -40 -50 -60 -70 -80 -90
f1 (ppm)

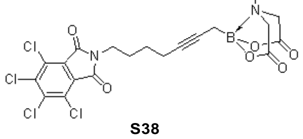

S38

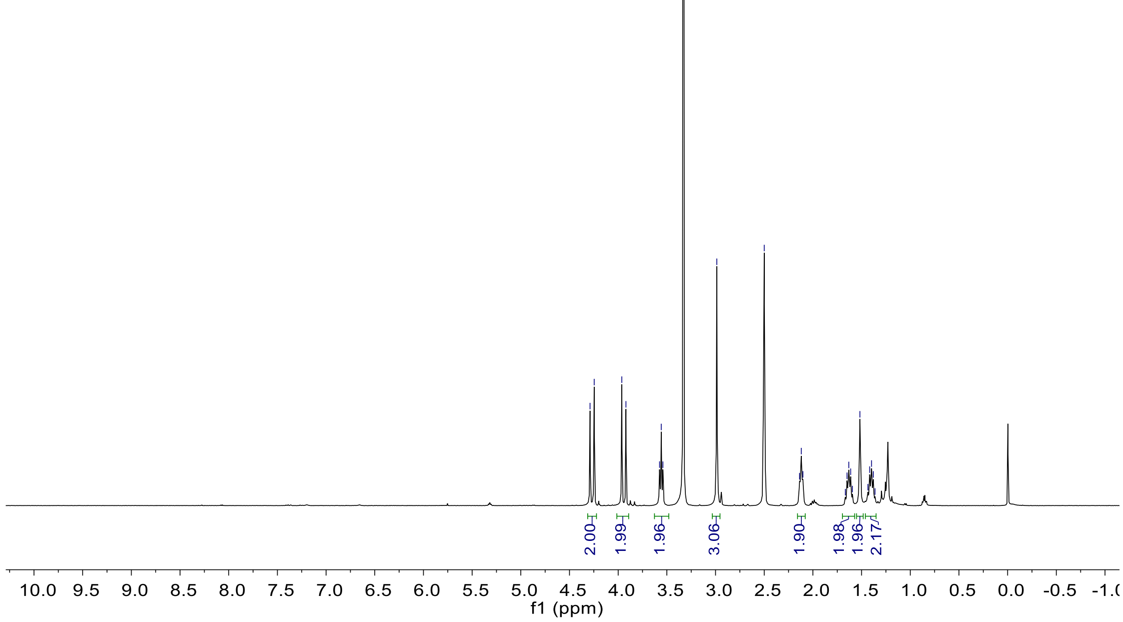

4.29
4.25
3.96
3.92
3.57
3.56
3.54
2.99
2.50 DMSO-d6
2.14
2.12
2.10
1.67
1.65
1.63
1.61
1.59
1.52
1.43
1.42
1.40
1.38
1.36
2.00
1.99
1.96
3.06
1.90
1.98
1.96
2.17
10.0 9.5 9.0 8.5 8.0 7.5 7.0 6.5 6.0 5.5 5.0 4.5 4.0 3.5 3.0 2.5 2.0 1.5 1.0 0.5 0.0 -0.5
f1 (ppm)

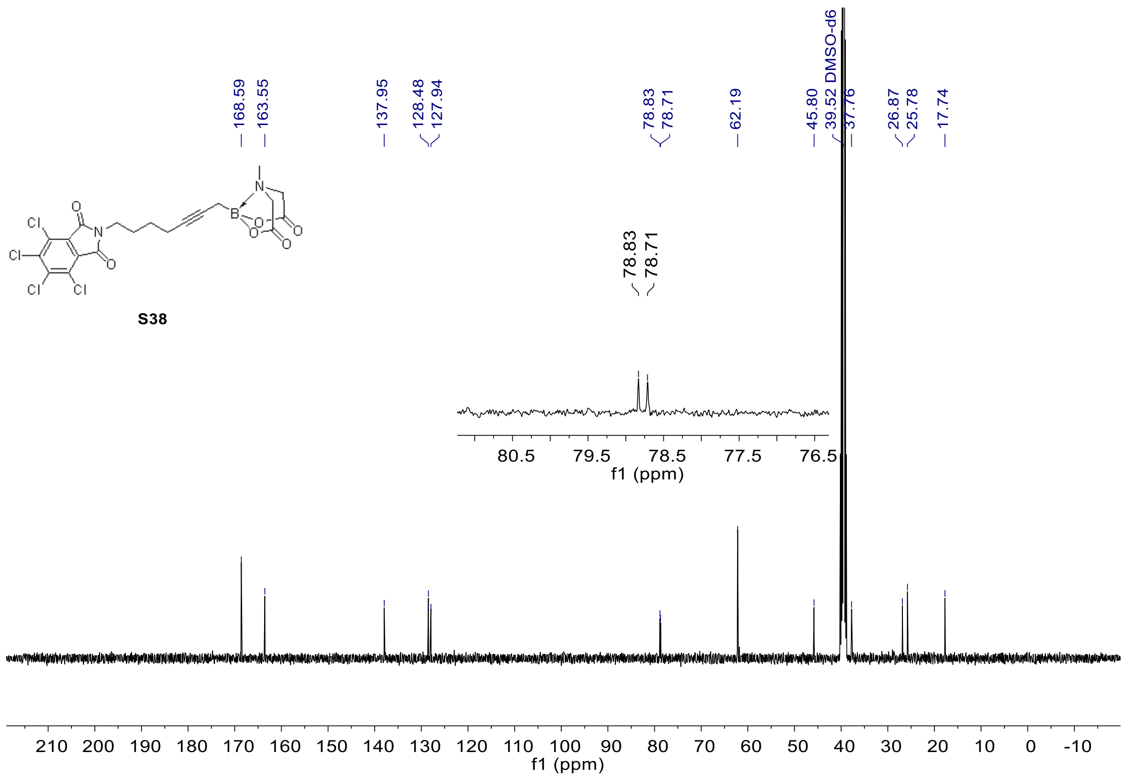
168.59
163.55
137.95
128.48
127.94
78.83
78.71
62.19
45.80
39.52 DMSO-d6
37.76
26.87
25.78
17.74
S38
80.5
79.5
78.5
77.5
76.5
f1 (ppm)
210 200 190 180 170 160 150 140 130 120 110 100 90 80 70 60 50 40 30 20 10 0 -10
f1 (ppm)

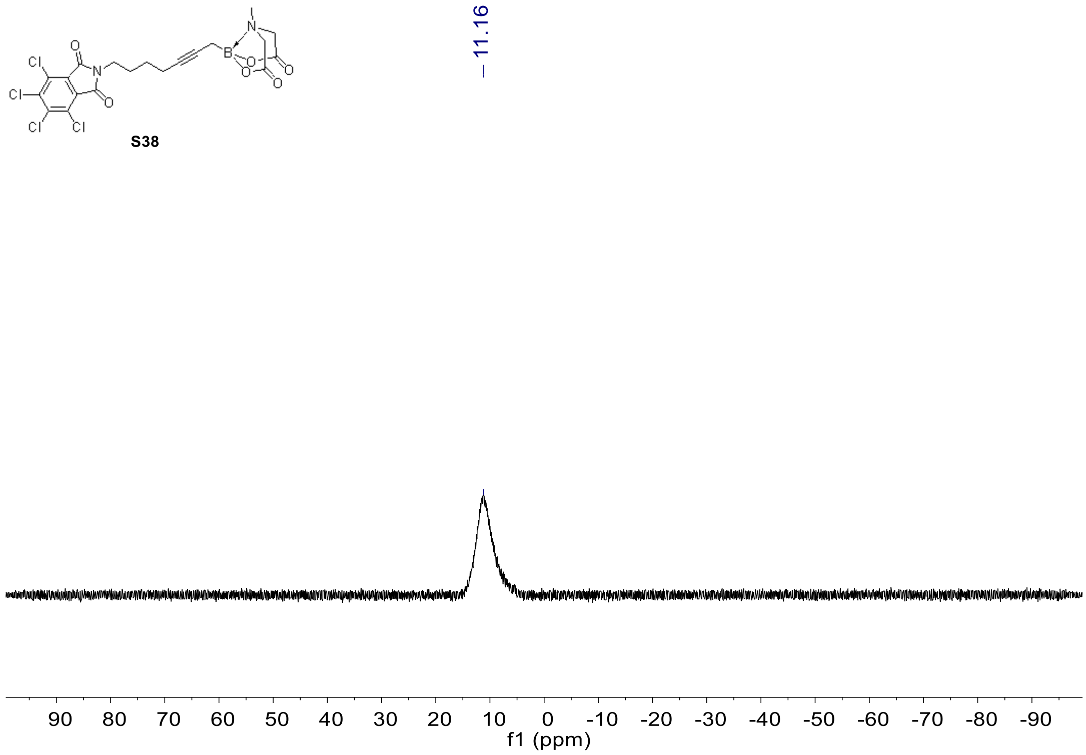
11.16
S38
90 80 70 60 50 40 30 20 10 0 -10 -20 -30 -40 -50 -60 -70 -80 -90
f1 (ppm)

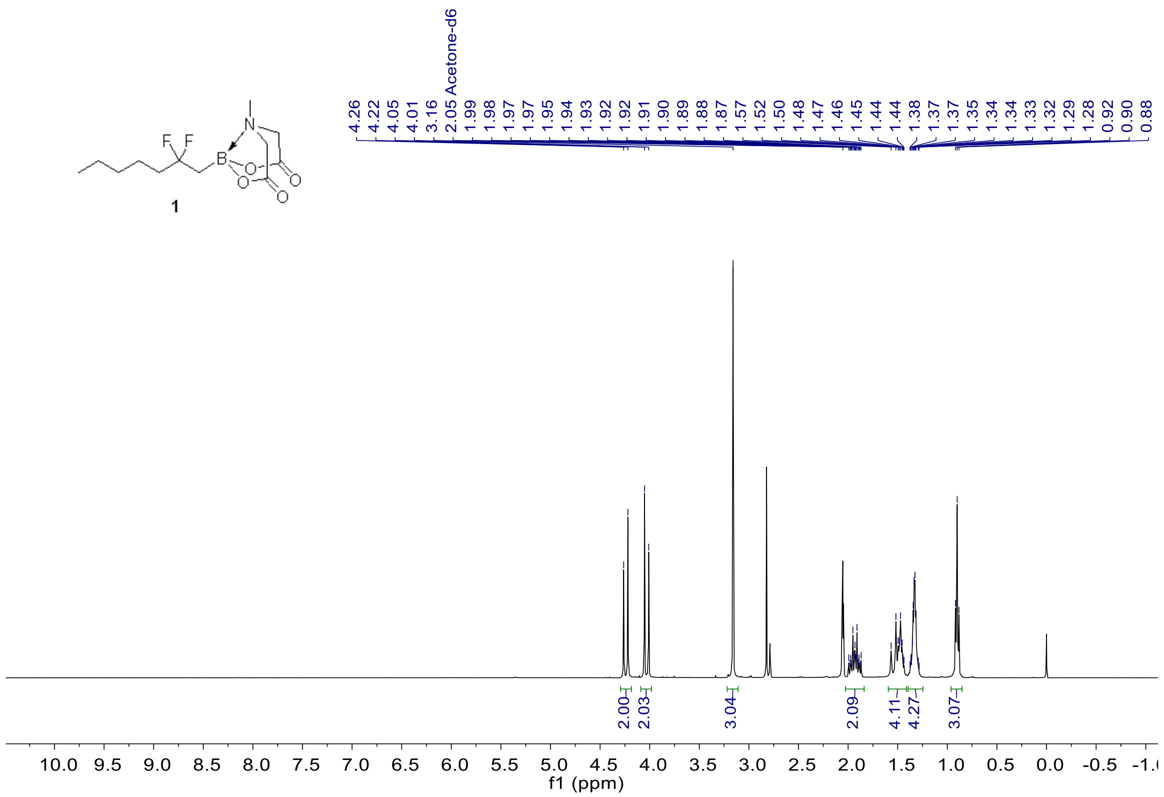

4.26 4.22 4.05 4.01 3.16 2.05 Acetone-d6 1.99 1.98 1.97 1.97 1.95 1.94 1.93 1.92 1.92 1.91 1.90 1.89 1.88 1.87 1.57 1.52 1.50 1.48 1.47 1.46 1.45 1.44 1.44 1.38 1.37 1.37 1.35 1.34 1.33 1.32 1.29 1.28 0.92 0.90 0.88
F F
B
N
O
1
2.00 2.03 3.04 2.09 4.11 4.27 3.07
f1 (ppm)

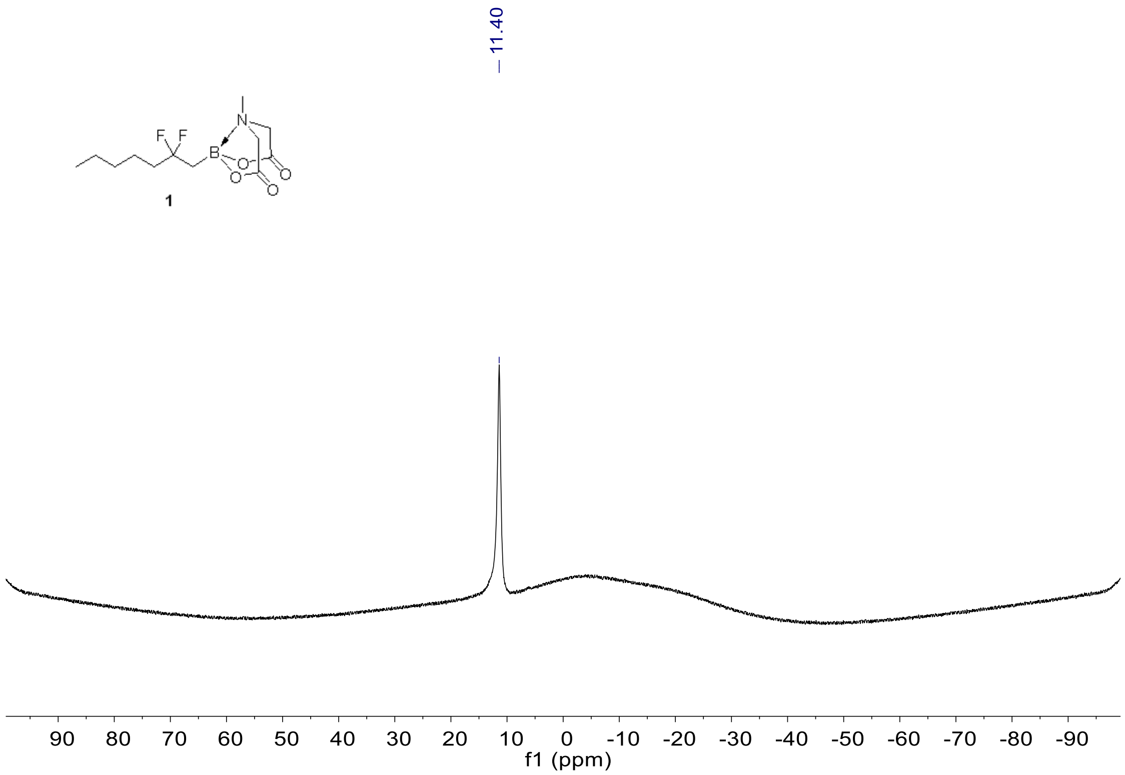

11.40
F F
B
N
O
1
f1 (ppm)

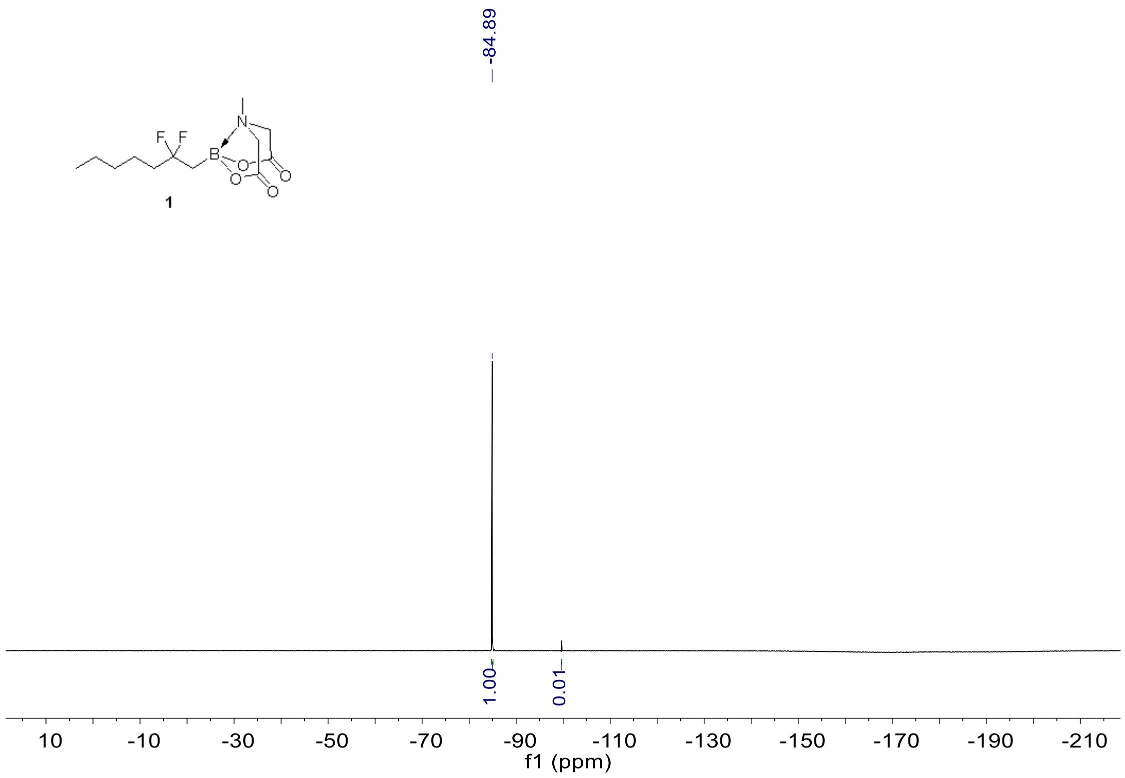
-84.89
F F
B
N
O
O
O
O
1
1.00
0.01
10 -10 -30 -50 -70 -90 -110 -130 -150 -170 -190 -210
f1 (ppm)

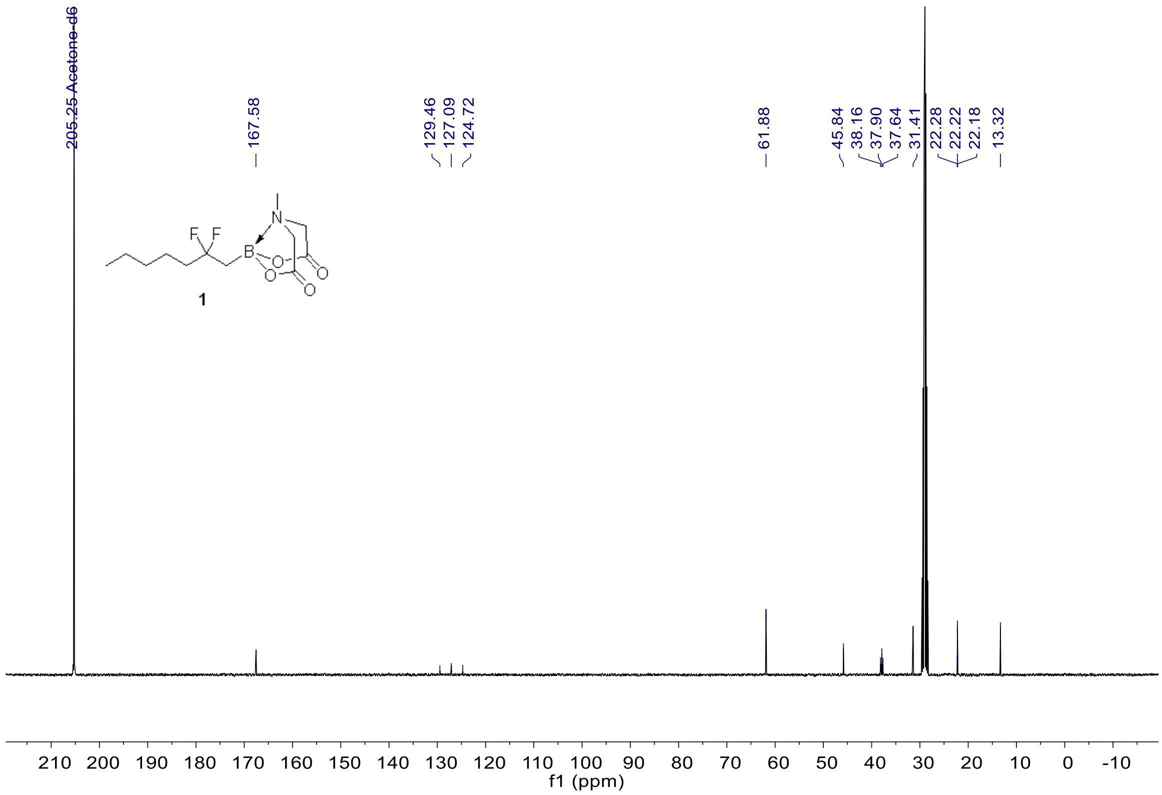
205.25 Acetone-d6
167.58
129.46
127.09
124.72
61.88
45.84
38.16
37.90
37.64
31.41
22.28
22.22
22.18
13.32
F F
B
N
O
O
O
O
1
210 200 190 180 170 160 150 140 130 120 110 100 90 80 70 60 50 40 30 20 10 0 -10
f1 (ppm)

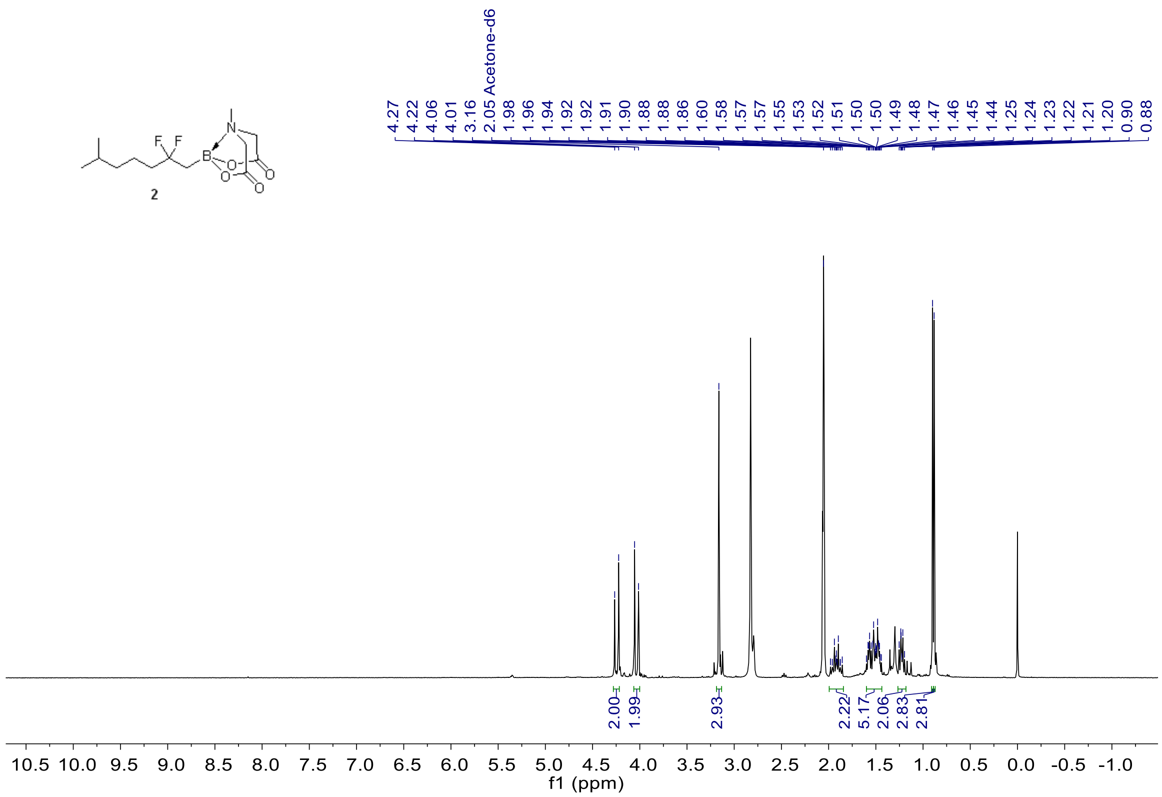
F F
N
B
O
O
O
O
2
4.27
4.22
4.06
4.01
3.16
2.05 Acetone-d6
1.98
1.96
1.94
1.92
1.92
1.91
1.90
1.88
1.86
1.60
1.58
1.57
1.57
1.55
1.53
1.52
1.51
1.50
1.50
1.49
1.48
1.47
1.46
1.45
1.44
1.25
1.24
1.23
1.22
1.21
1.20
0.90
0.88
2.00
1.99
2.93
2.22
5.17
2.06
2.83
2.81
10.5 10.0 9.5 9.0 8.5 8.0 7.5 7.0 6.5 6.0 5.5 5.0 4.5 4.0 3.5 3.0 2.5 2.0 1.5 1.0 0.5 0.0 -0.5 -1.0
f1 (ppm)

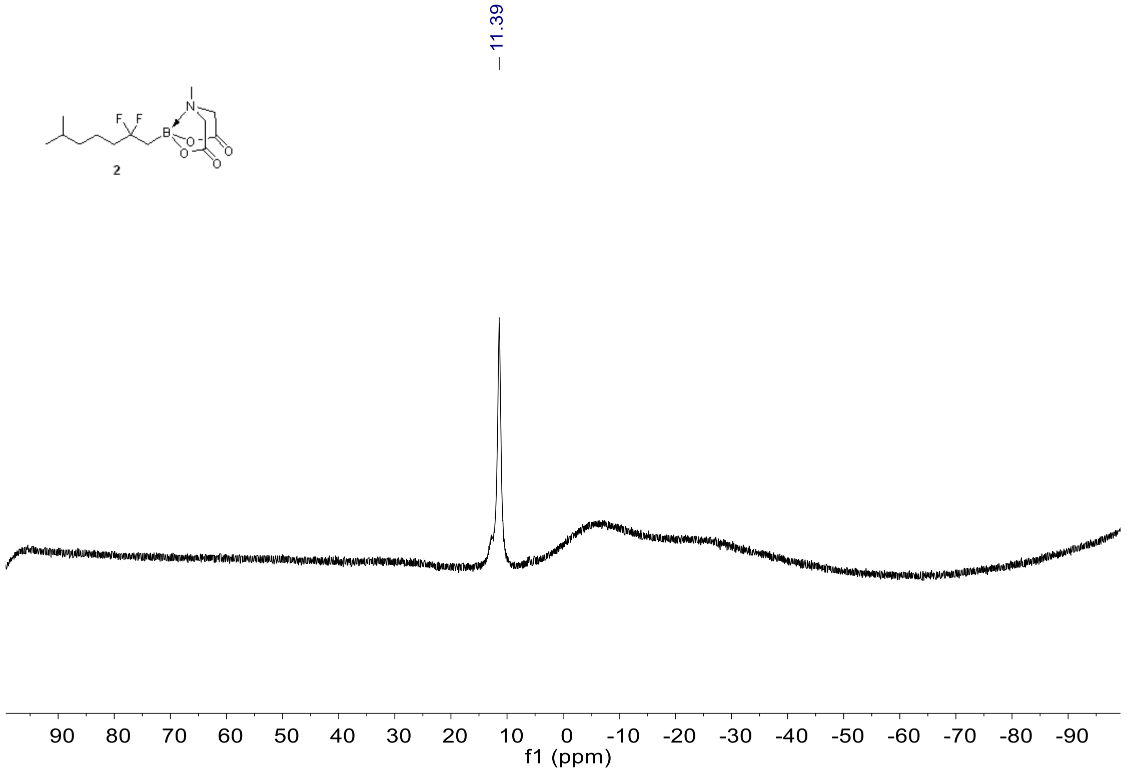
F F
N
B
O
O
O
O
2
11.39
90 80 70 60 50 40 30 20 10 0 -10 -20 -30 -40 -50 -60 -70 -80 -90
f1 (ppm)

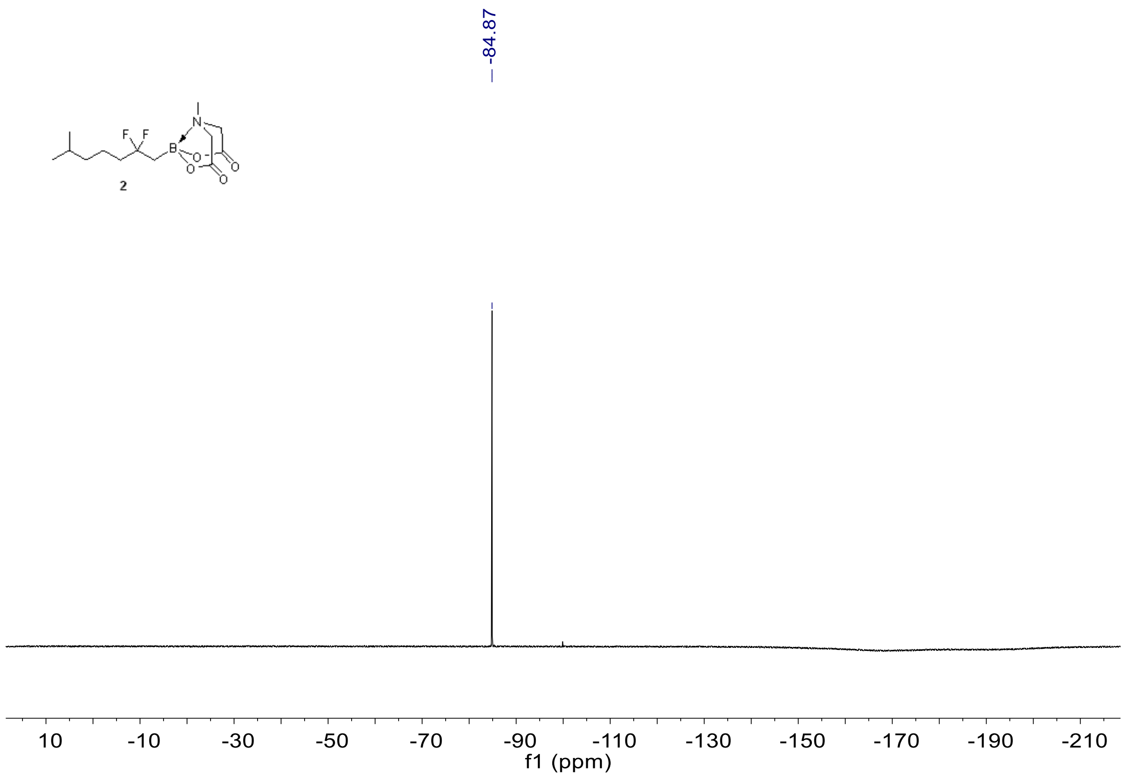
-84.87
2
10 -10 -30 -50 -70 -90 -110 -130 -150 -170 -190 -210
f1 (ppm)

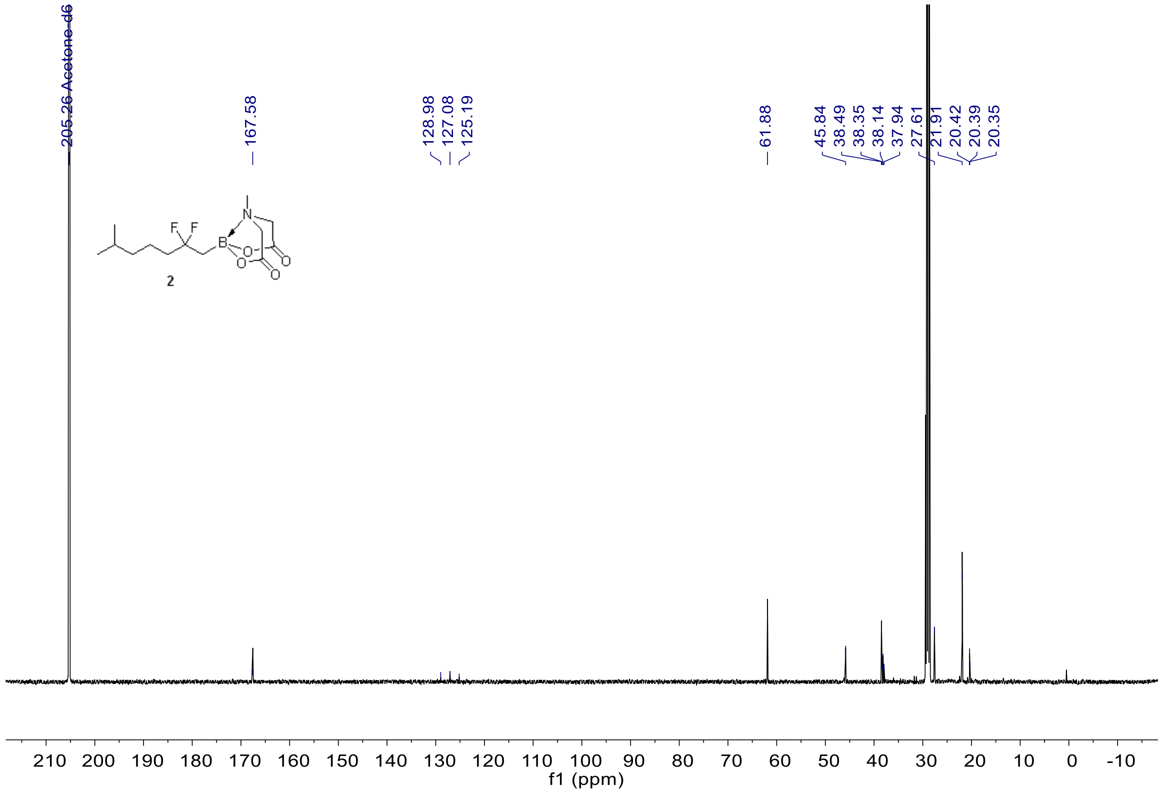
205.26 Acetone-d6
167.58
128.98
127.08
125.19
61.88
45.84
38.49
38.35
38.14
37.94
27.61
21.91
20.42
20.39
20.35
2
210 200 190 180 170 160 150 140 130 120 110 100 90 80 70 60 50 40 30 20 10 0 -10
f1 (ppm)

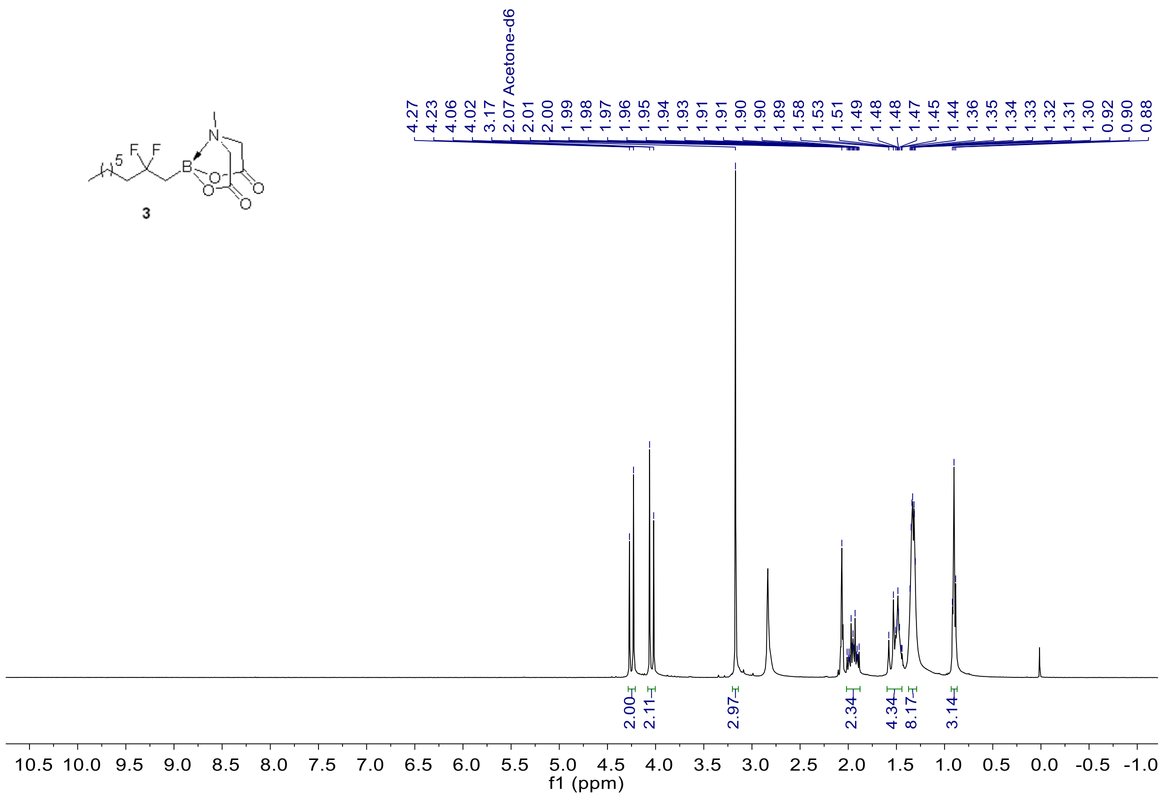
4.27
4.23
4.06
4.02
3.17
2.07 Acetone-d6
2.01
2.00
1.99
1.98
1.97
1.96
1.95
1.94
1.93
1.91
1.91
1.90
1.90
1.89
1.58
1.53
1.51
1.49
1.48
1.48
1.47
1.45
1.44
1.36
1.35
1.34
1.33
1.32
1.31
1.30
0.92
0.90
0.88
3
2.00
2.11
2.97
2.34
4.34
8.17
3.14
10.5 10.0 9.5 9.0 8.5 8.0 7.5 7.0 6.5 6.0 5.5 5.0 4.5 4.0 3.5 3.0 2.5 2.0 1.5 1.0 0.5 0.0 -0.5 -1.0
f1 (ppm)

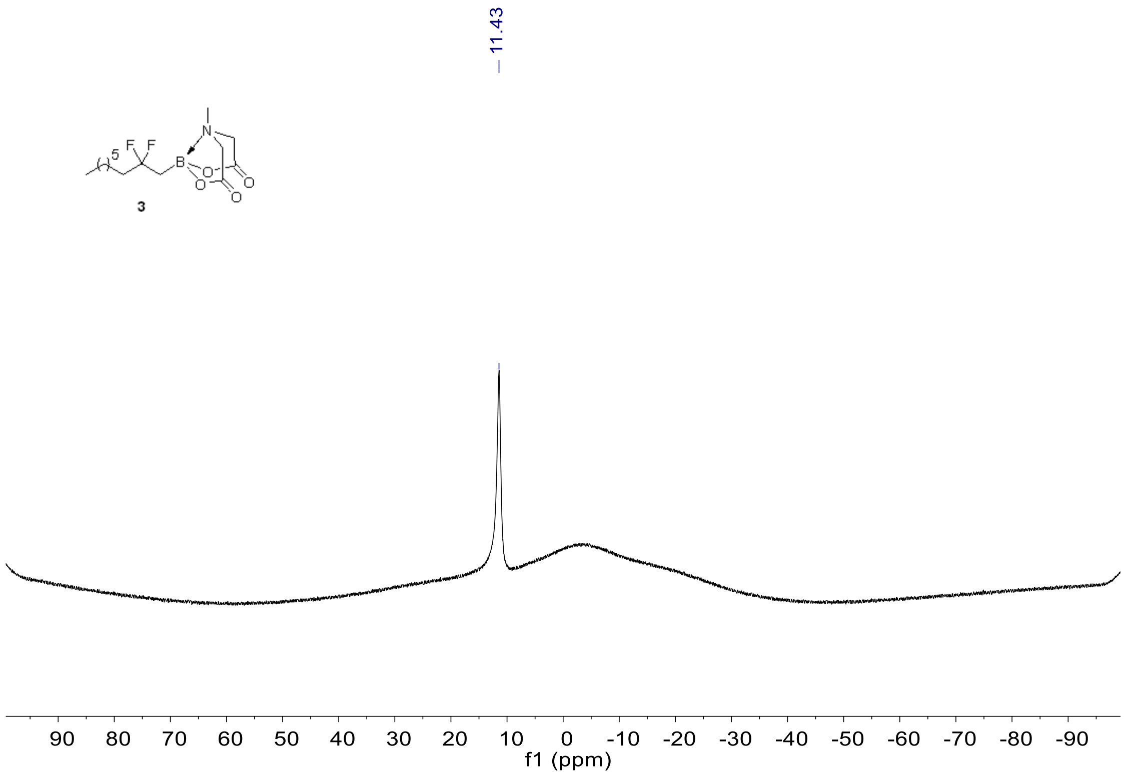
11.43
3
90 80 70 60 50 40 30 20 10 0 -10 -20 -30 -40 -50 -60 -70 -80 -90
f1 (ppm)

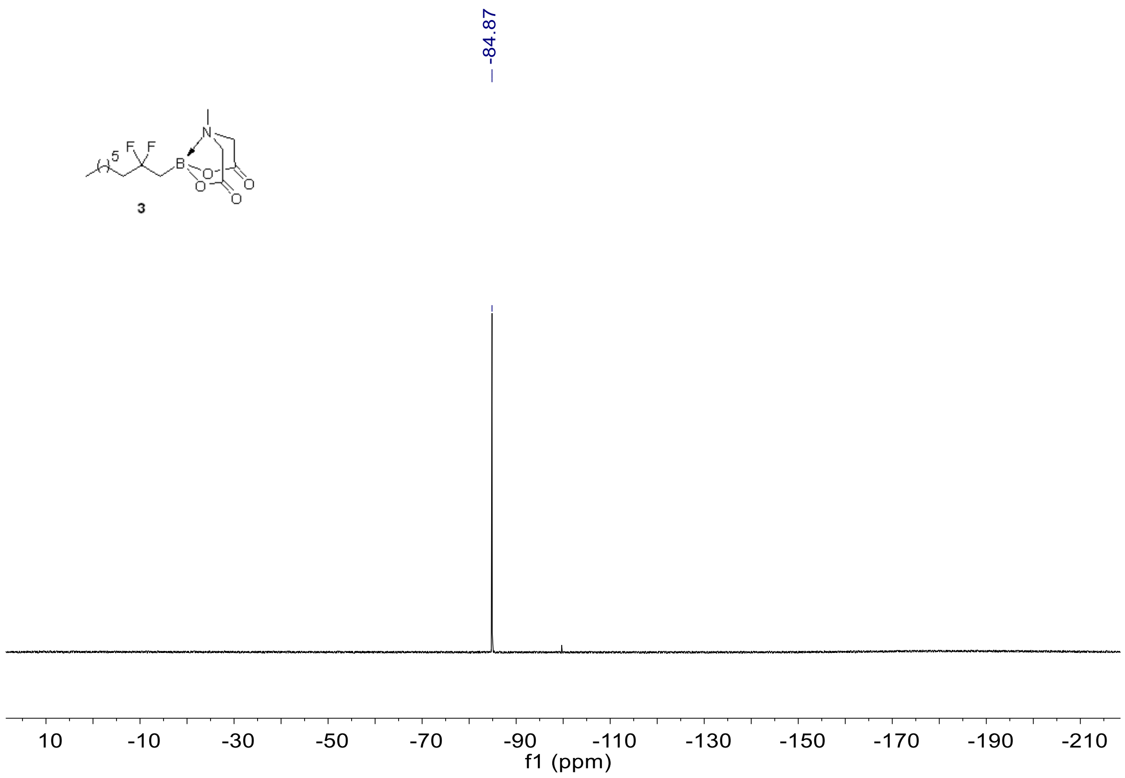

-84.87
F F
B
N
O
O
O
O
5
3
10 -10 -30 -50 -70 -90 -110 -130 -150 -170 -190 -210
f1 (ppm)

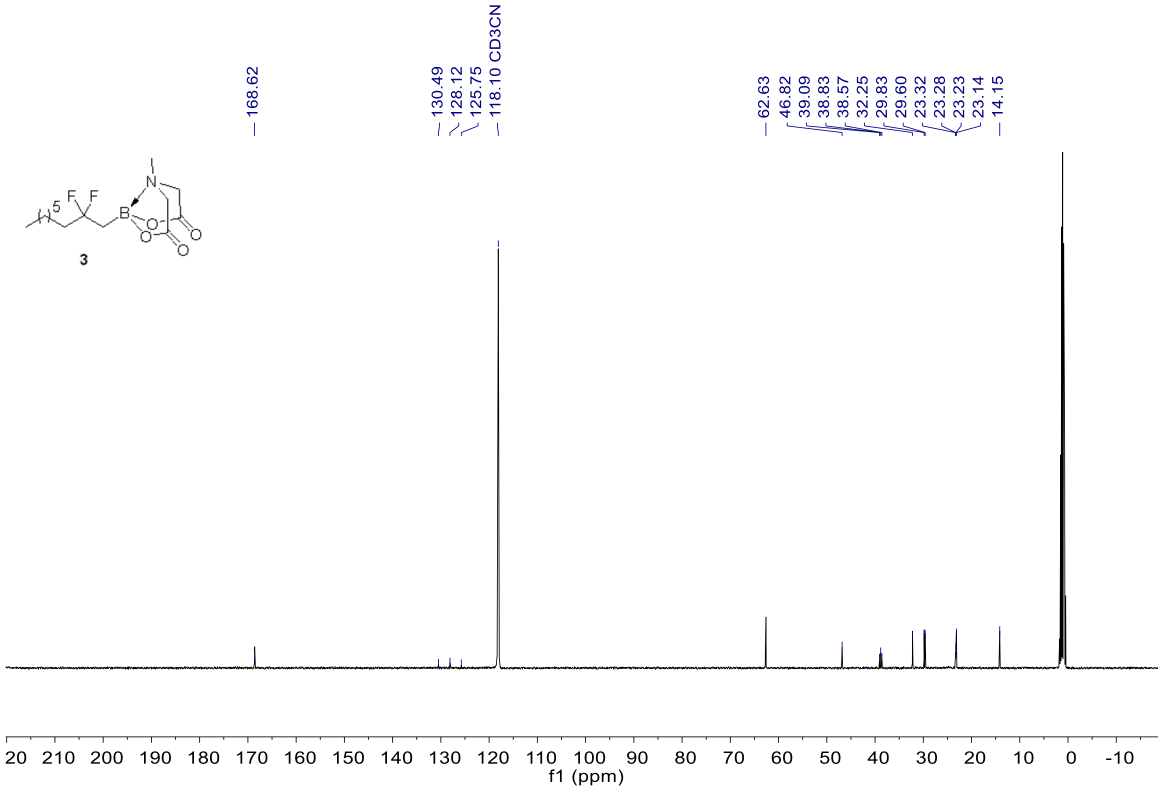

168.62
130.49
128.12
125.75
118.10 CD3CN
62.63
46.82
39.09
38.83
38.57
32.25
29.83
29.60
23.32
23.28
23.23
23.14
14.15
F F
B
N
O
O
O
O
5
3
20 210 200 190 180 170 160 150 140 130 120 110 100 90 80 70 60 50 40 30 20 10 0 -10
f1 (ppm)

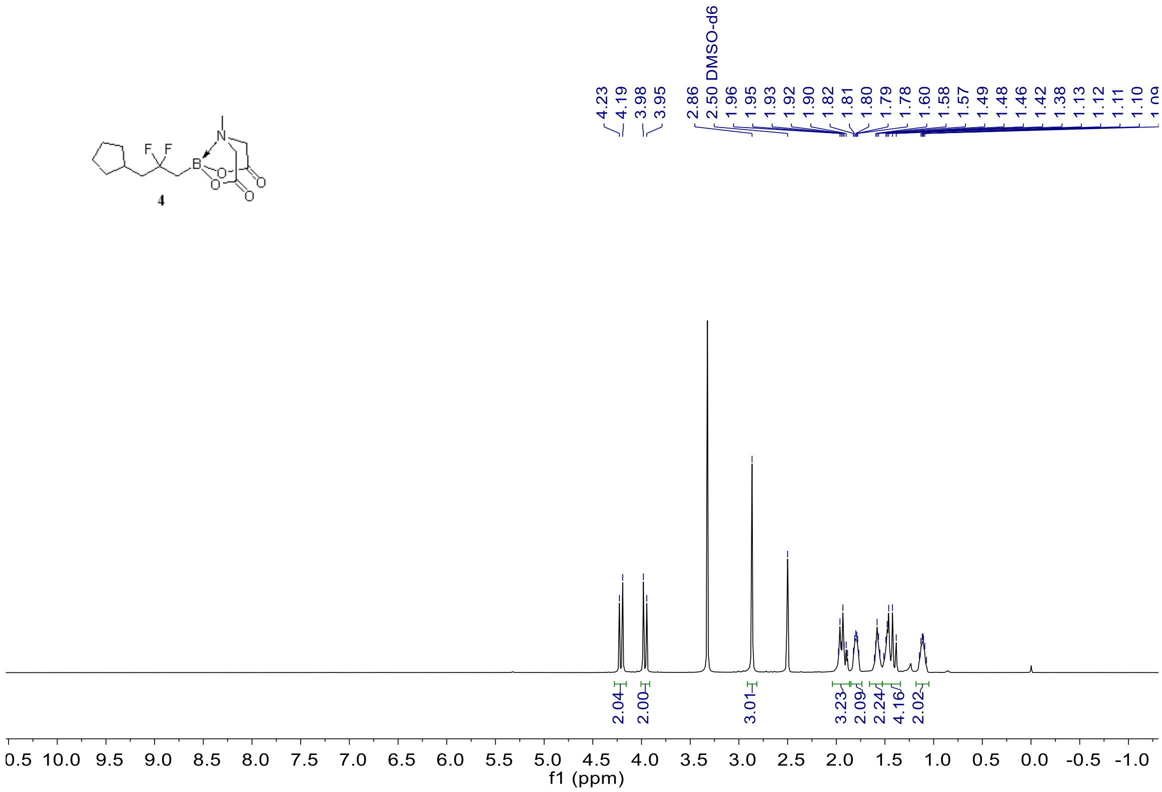
4.23
4.19
3.98
3.95
2.86
2.50 DMSO-d6
1.96
1.95
1.93
1.92
1.90
1.82
1.81
1.80
1.79
1.78
1.60
1.58
1.57
1.49
1.48
1.46
1.42
1.38
1.13
1.12
1.11
1.10
2.04
2.00
3.01
3.23
2.09
2.24
4.16
2.02
f1 (ppm)

4

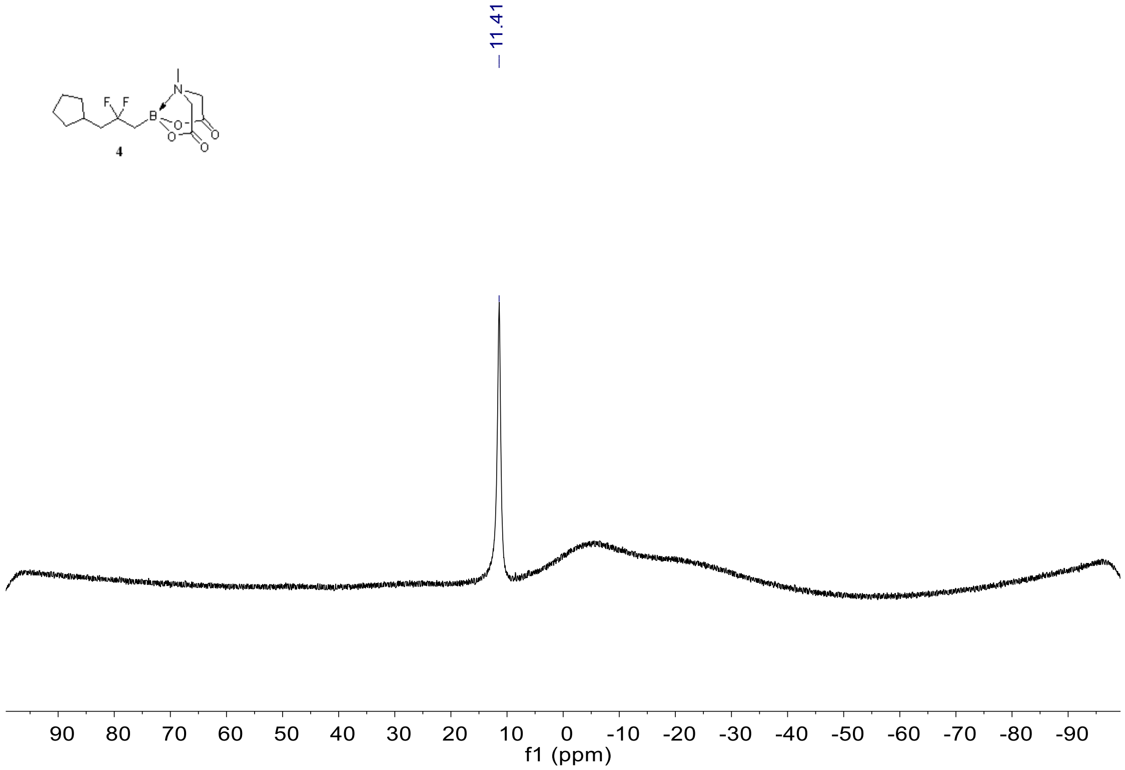
11.41
f1 (ppm)

4

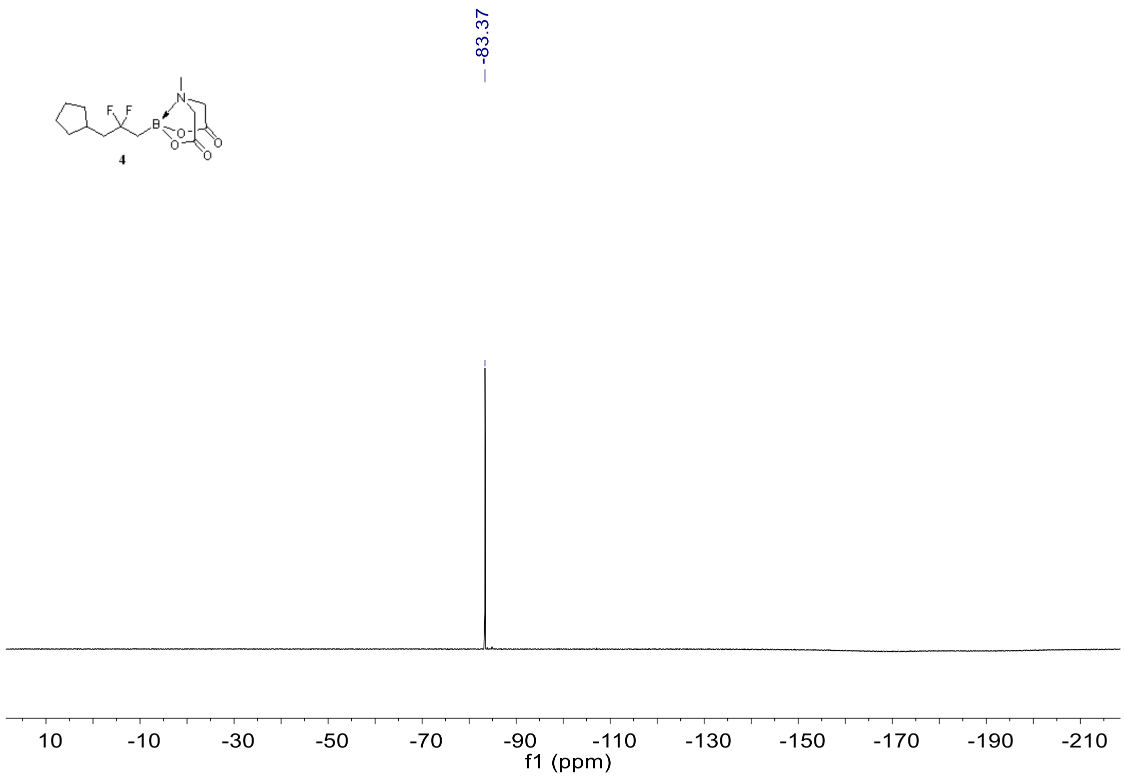
-83.37
F F
B
N
O
O
O
O
4
10 -10 -30 -50 -70 -90 -110 -130 -150 -170 -190 -210
f1 (ppm)

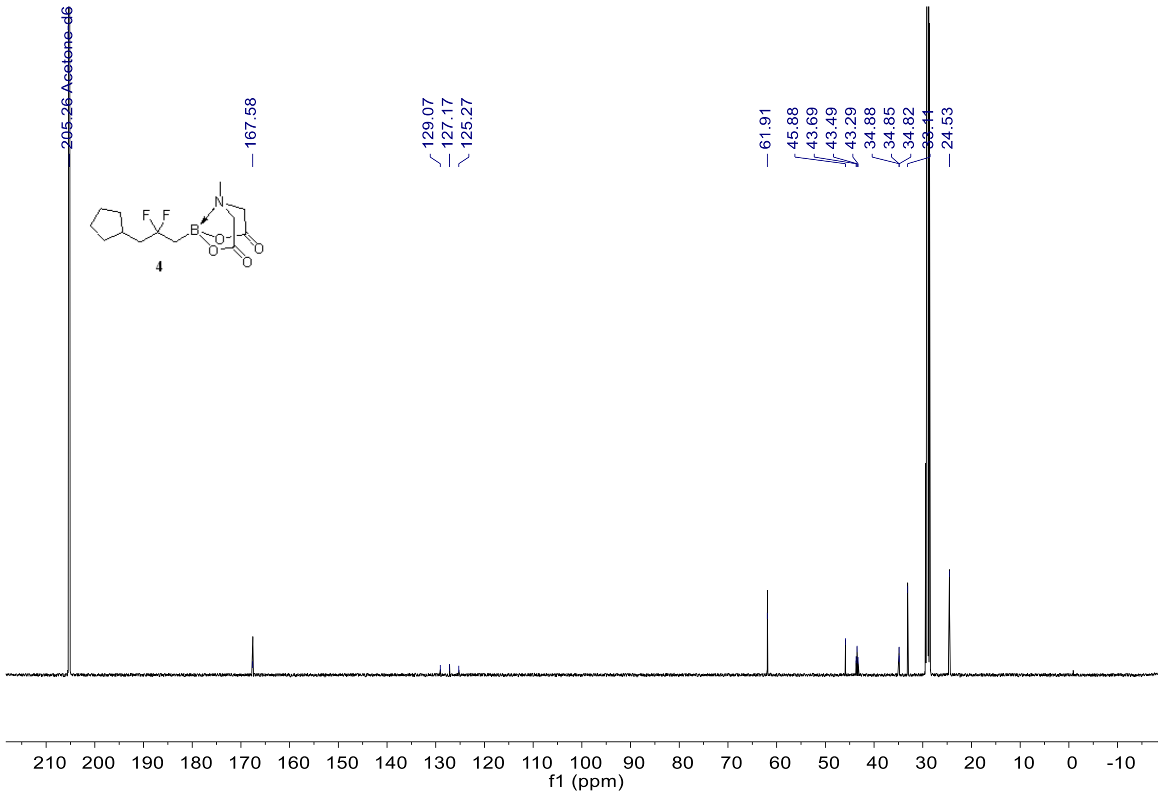
205.26 Acetone-d6
167.58
129.07
127.17
125.27
61.91
45.88
43.69
43.49
43.29
34.88
34.85
34.82
33.11
24.53
F F
B
N
O
O
O
O
4
210 200 190 180 170 160 150 140 130 120 110 100 90 80 70 60 50 40 30 20 10 0 -10
f1 (ppm)

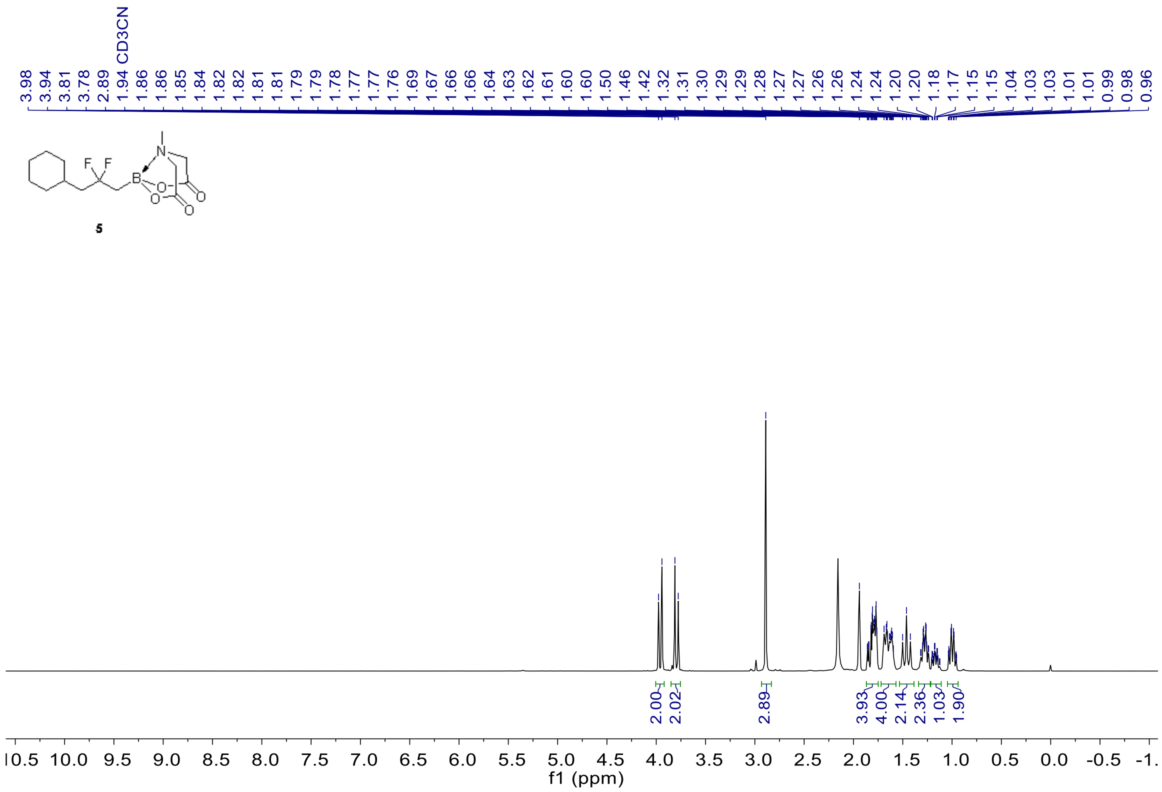
3.98 3.94 3.81 3.78 2.89 1.94 CD3CN 1.86 1.86 1.85 1.84 1.82 1.82 1.81 1.81 1.79 1.79 1.78 1.77 1.77 1.76 1.69 1.67 1.66 1.66 1.64 1.63 1.62 1.61 1.60 1.60 1.50 1.46 1.42 1.32 1.31 1.30 1.29 1.29 1.28 1.27 1.27 1.26 1.26 1.24 1.24 1.20 1.20 1.18 1.17 1.15 1.15 1.04 1.03 1.03 1.01 1.01 0.99 0.98 0.96
5
2.00 2.02 2.89 3.93 4.00 2.14 2.36 1.03 1.90
10.5 10.0 9.5 9.0 8.5 8.0 7.5 7.0 6.5 6.0 5.5 5.0 4.5 4.0 3.5 3.0 2.5 2.0 1.5 1.0 0.5 0.0 -0.5 -1.
f1 (ppm)

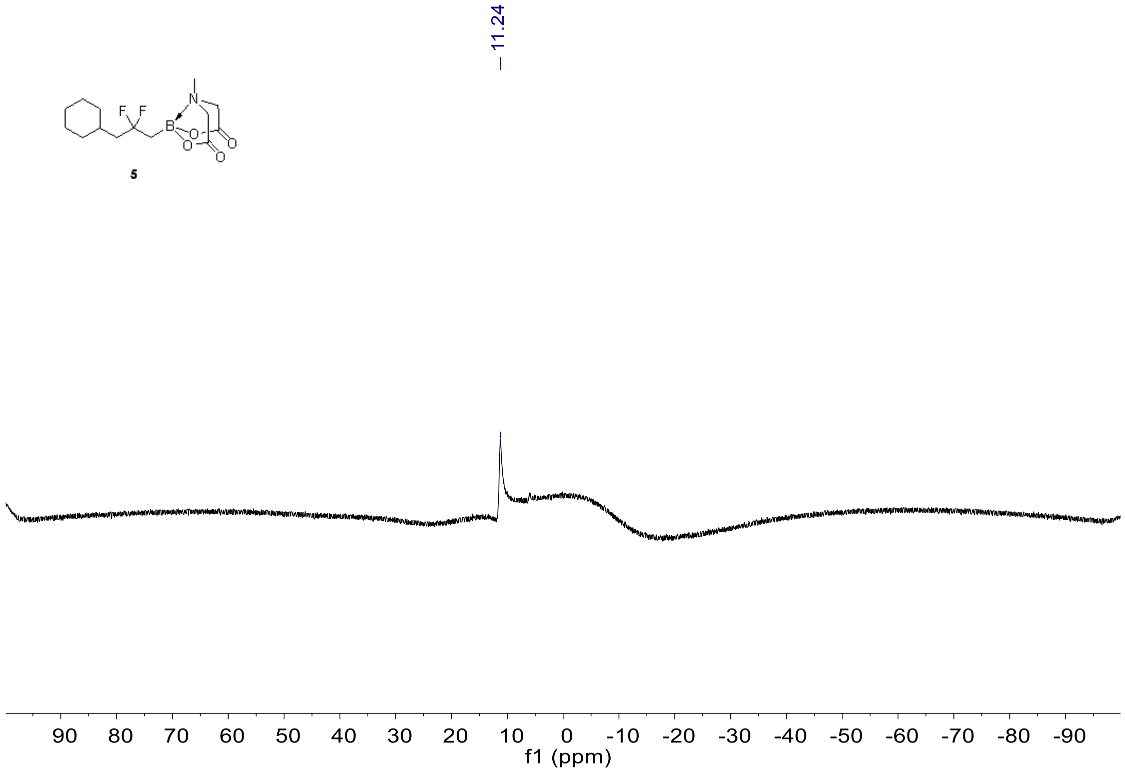
11.24
5
90 80 70 60 50 40 30 20 10 0 -10 -20 -30 -40 -50 -60 -70 -80 -90
f1 (ppm)

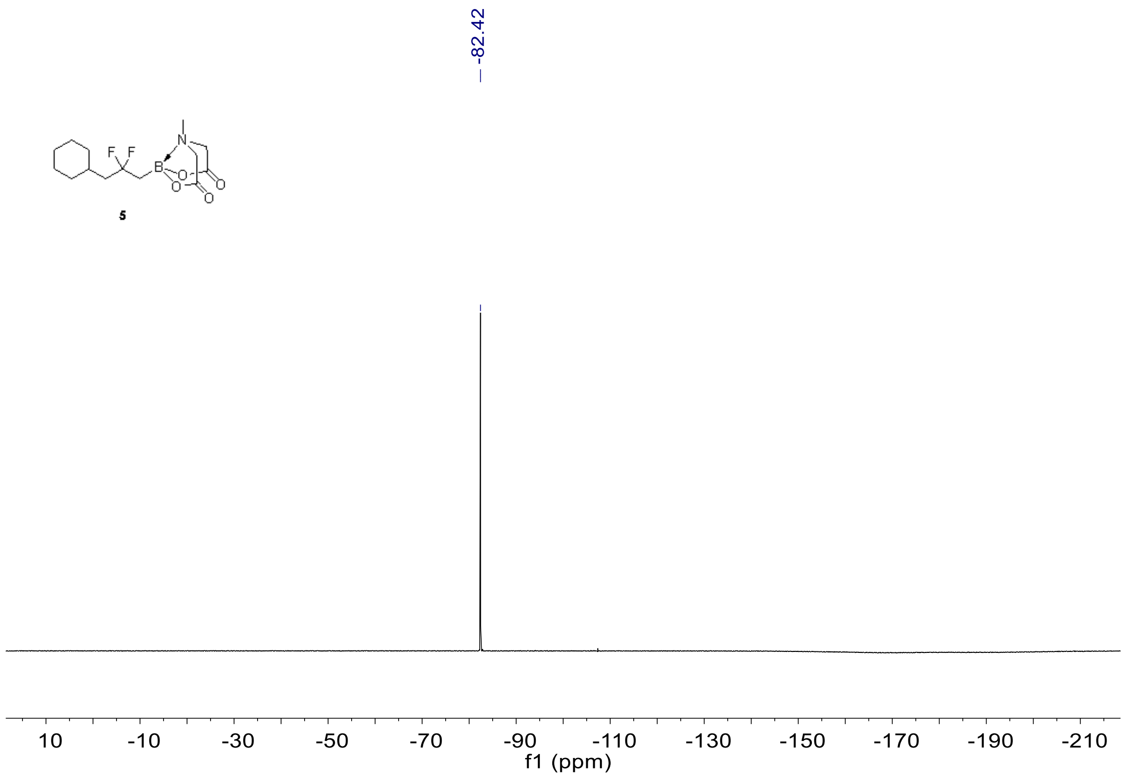

-82.42
F F
N
B
O
O
O
O
5
10 -10 -30 -50 -70 -90 -110 -130 -150 -170 -190 -210
f1 (ppm)

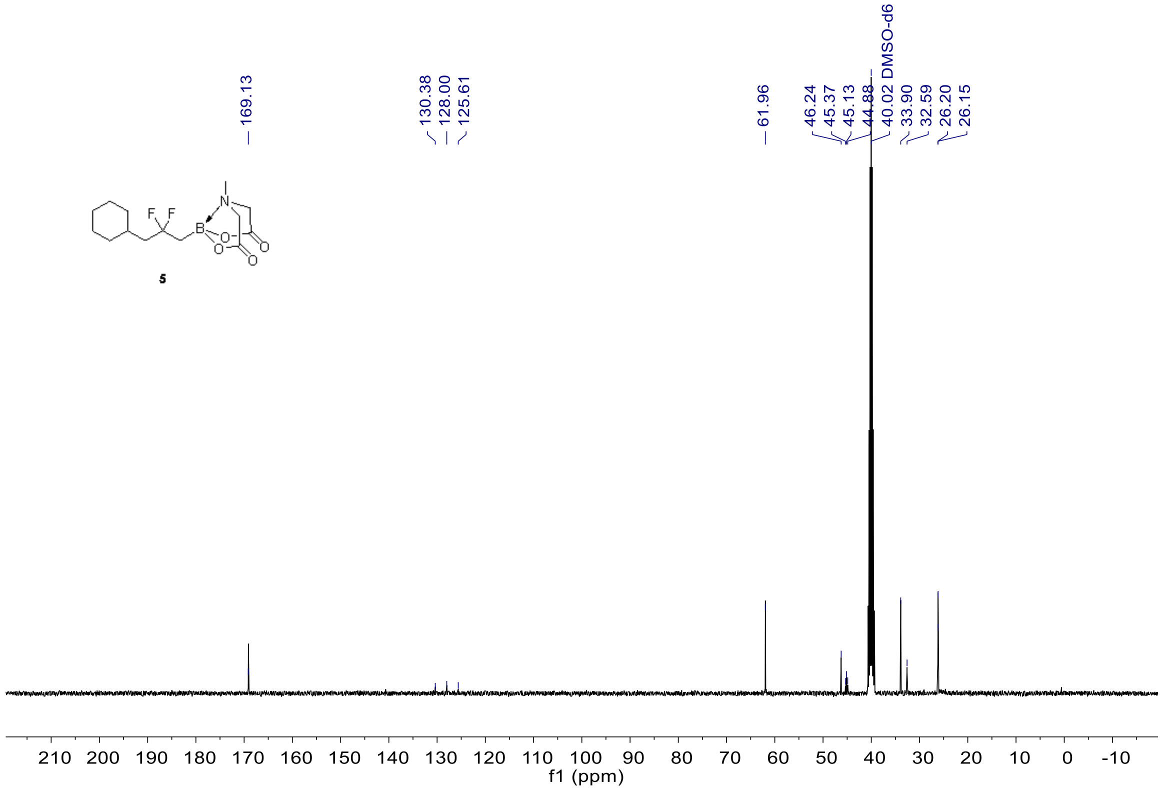

169.13
130.38
128.00
125.61
61.96
46.24
45.37
45.13
44.88
40.02 DMSO-d6
33.90
32.59
26.20
26.15
F F
N
B
O
O
O
O
5
210 200 190 180 170 160 150 140 130 120 110 100 90 80 70 60 50 40 30 20 10 0 -10
f1 (ppm)

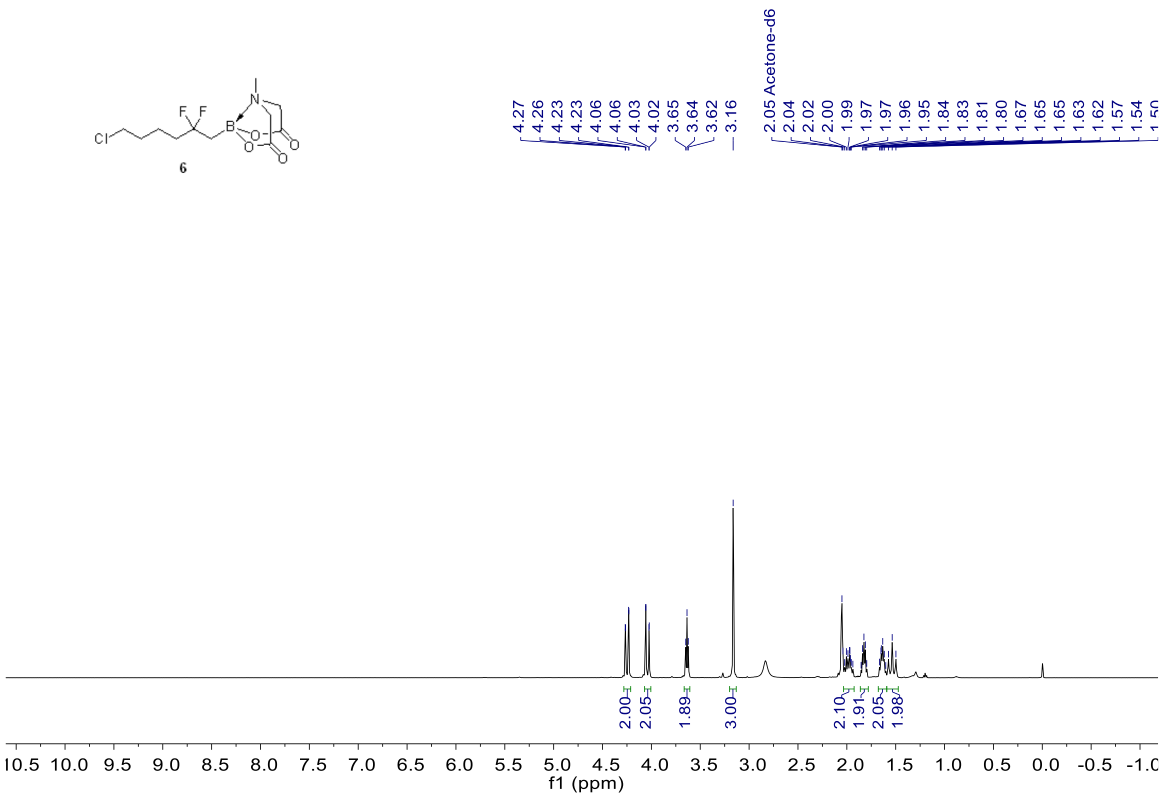

Cl
F F
N
B
O
O
O
O
6
4.27
4.26
4.23
4.23
4.06
4.06
4.03
4.02
3.65
3.64
3.62
3.16
2.05 Acetone-d6
2.04
2.02
2.00
1.99
1.97
1.97
1.96
1.95
1.84
1.83
1.81
1.80
1.67
1.65
1.65
1.63
1.62
1.57
1.54
2.00
2.05
1.89
3.00
2.10
1.91
2.05
1.98
10.5 10.0 9.5 9.0 8.5 8.0 7.5 7.0 6.5 6.0 5.5 5.0 4.5 4.0 3.5 3.0 2.5 2.0 1.5 1.0 0.5 0.0 -0.5
f1 (ppm)

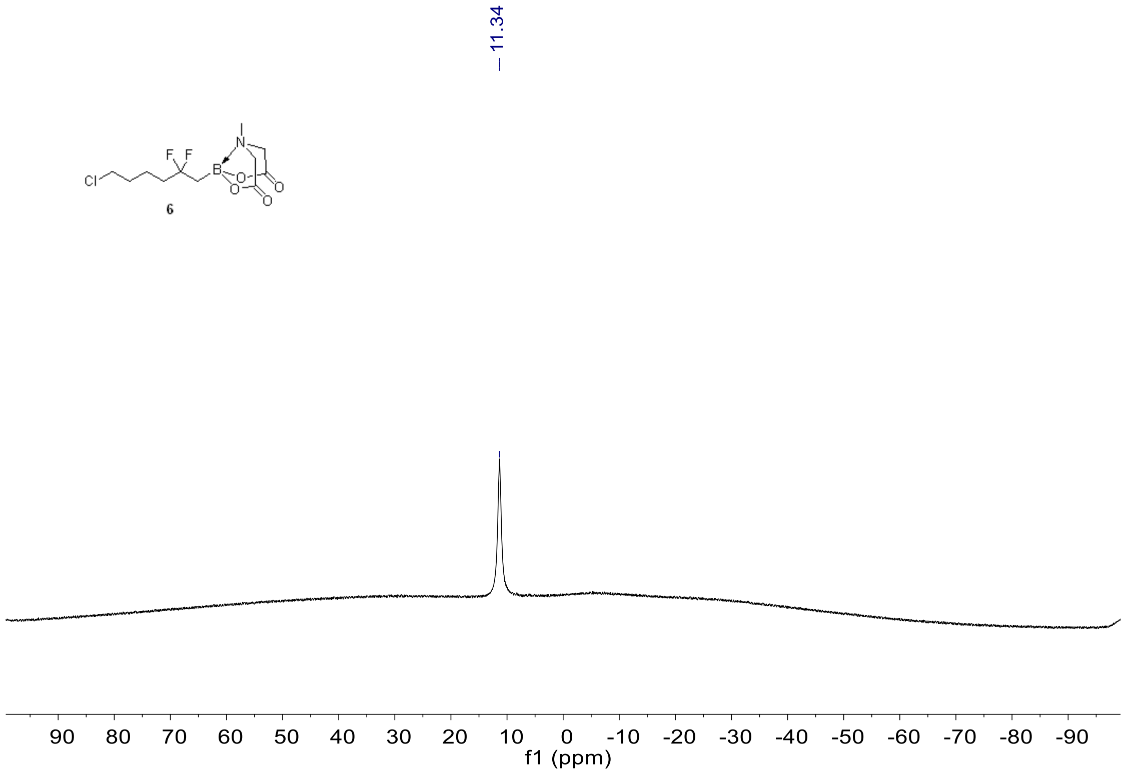

11.34
Cl
F F
N
B
O
O
O
O
6
90 80 70 60 50 40 30 20 10 0 -10 -20 -30 -40 -50 -60 -70 -80 -90
f1 (ppm)

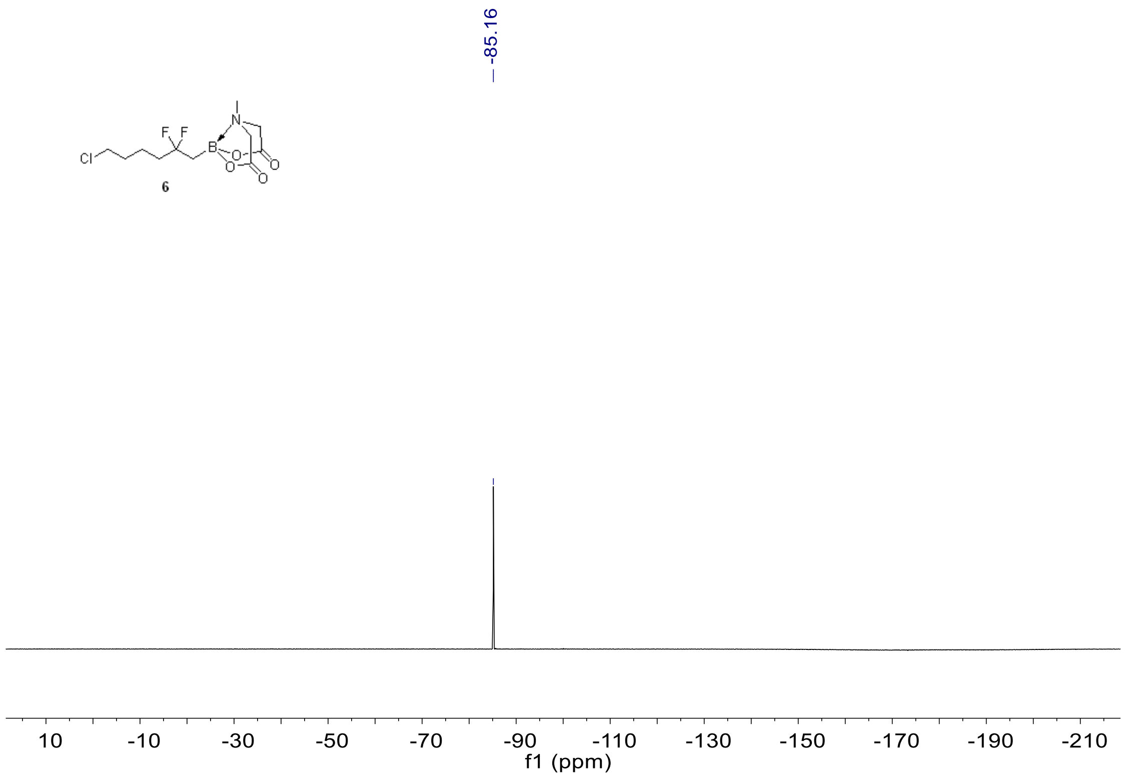

-85.16
Cl
F F
B
N
O
6
10 -10 -30 -50 -70 -90 -110 -130 -150 -170 -190 -210
f1 (ppm)

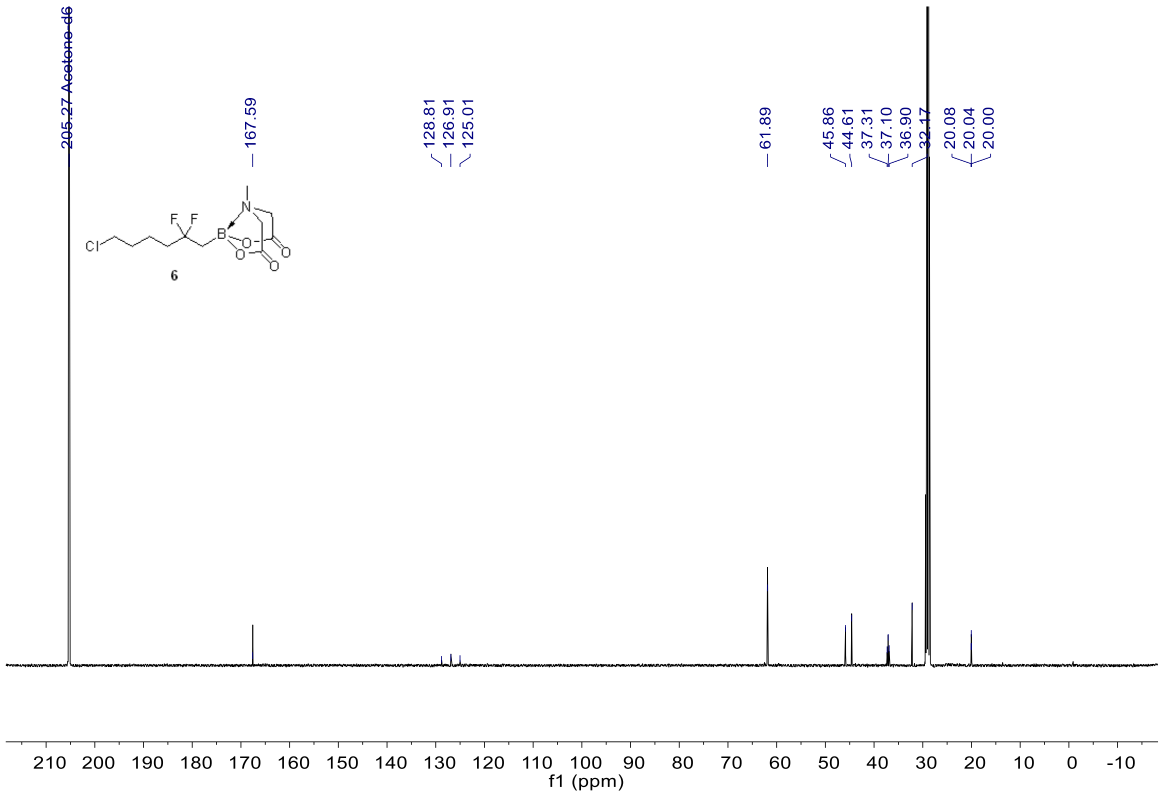

205.27 Acetone-d6
167.59
128.81
126.91
125.01
61.89
45.86
44.61
37.31
37.10
36.90
32.17
20.08
20.04
20.00
Cl
F F
B
N
O
6
210 200 190 180 170 160 150 140 130 120 110 100 90 80 70 60 50 40 30 20 10 0 -10
f1 (ppm)

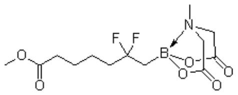

7

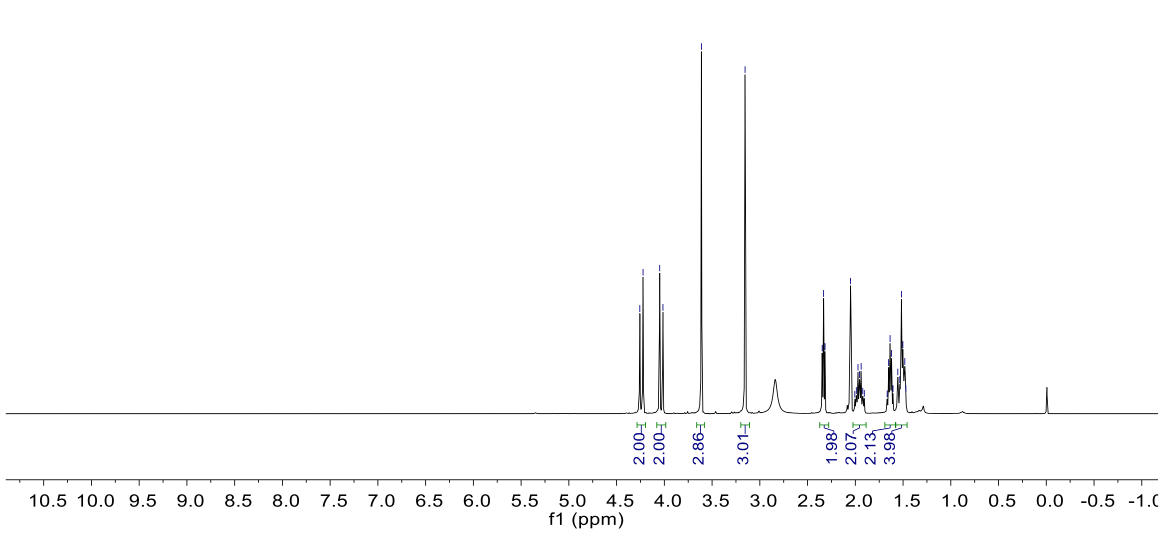
4.26
4.22
4.05
4.01
3.61
3.15
2.35
2.33
2.32
2.05 Acetone-d6
1.99
1.97
1.96
1.94
1.92
1.91
1.65
1.64
1.62
1.61
1.55
1.53
1.52
1.50
1.48
1.47
2.00
2.00
2.86
3.01
1.98
2.07
2.13
3.98
10.5 10.0 9.5 9.0 8.5 8.0 7.5 7.0 6.5 6.0 5.5 5.0 4.5 4.0 3.5 3.0 2.5 2.0 1.5 1.0 0.5 0.0 -0.5 -1.0
f1 (ppm)

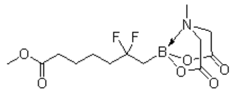

7

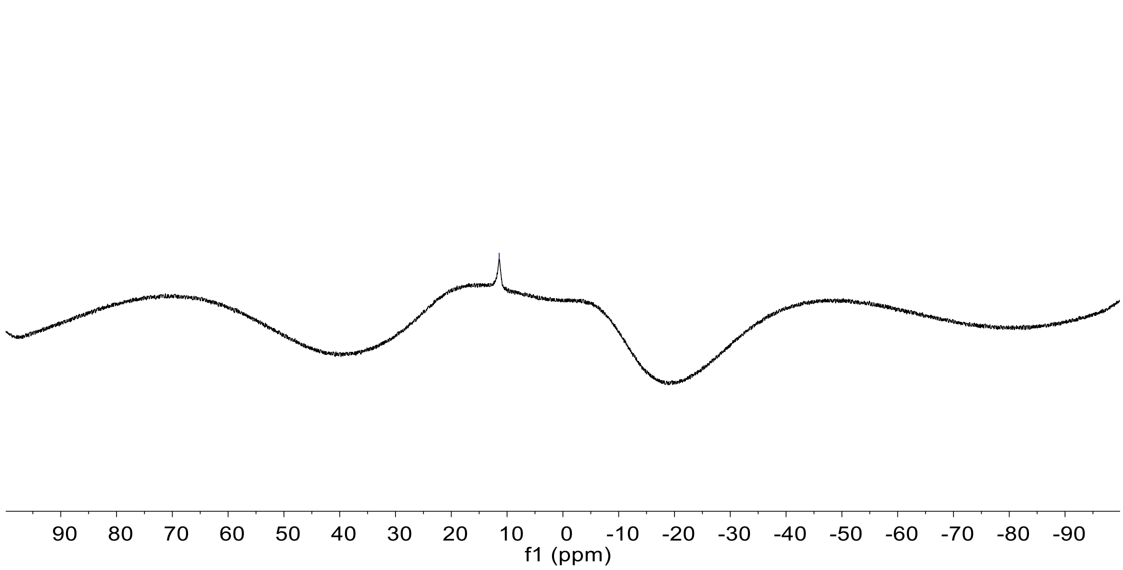
11.42
90 80 70 60 50 40 30 20 10 0 -10 -20 -30 -40 -50 -60 -70 -80 -90
f1 (ppm)

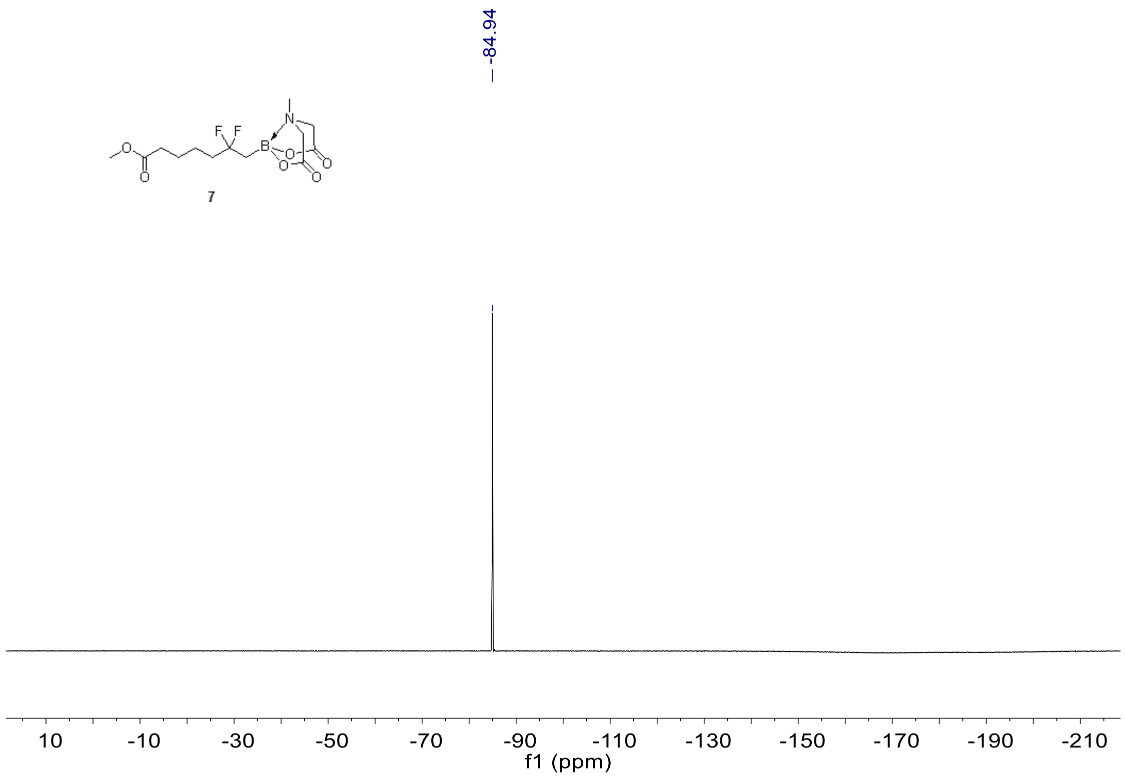

-84.94
F F
N
B
O
O
O
O
O
O
7
10
-10
-30
-50
-70
-90
-110
-130
-150
-170
-190
-210
f1 (ppm)

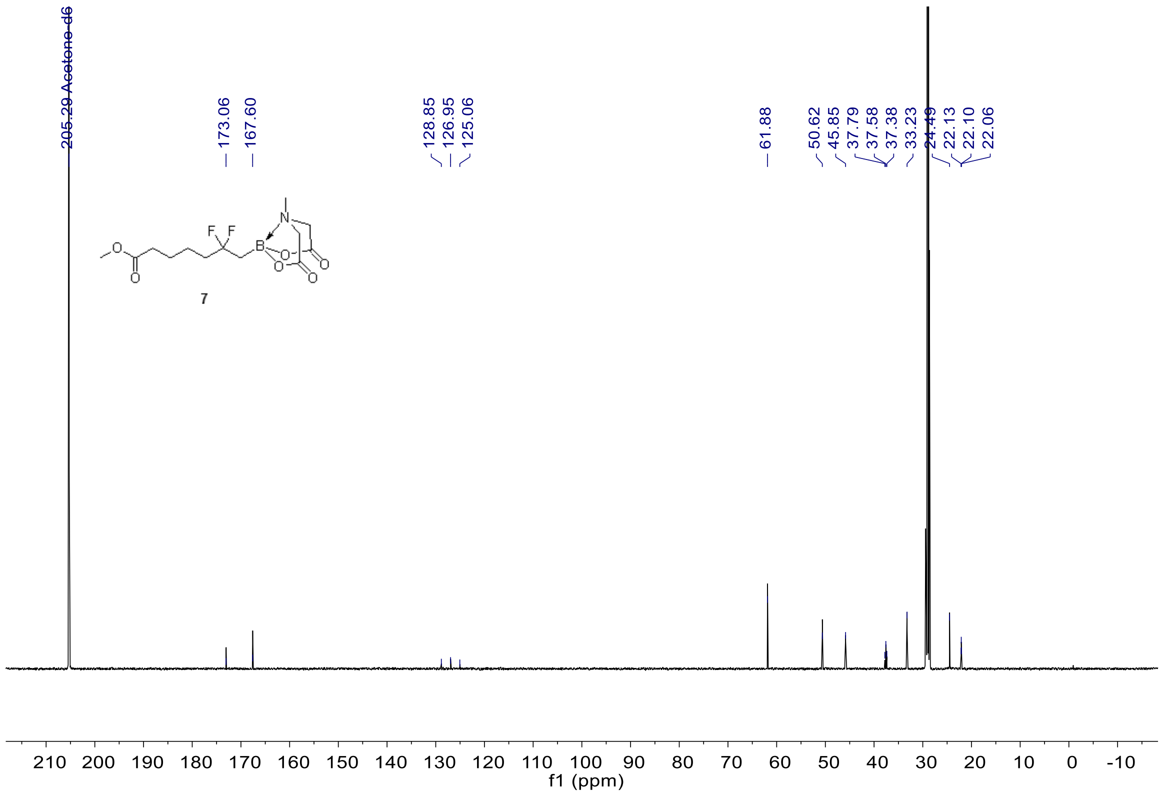

205.29 Acetone-d6
173.06
167.60
128.85
126.95
125.06
61.88
50.62
45.85
37.79
37.58
37.38
33.23
24.49
22.13
22.10
22.06
F F
N
B
O
O
O
O
O
O
7
210 200 190 180 170 160 150 140 130 120 110 100 90 80 70 60 50 40 30 20 10 0 -10
f1 (ppm)

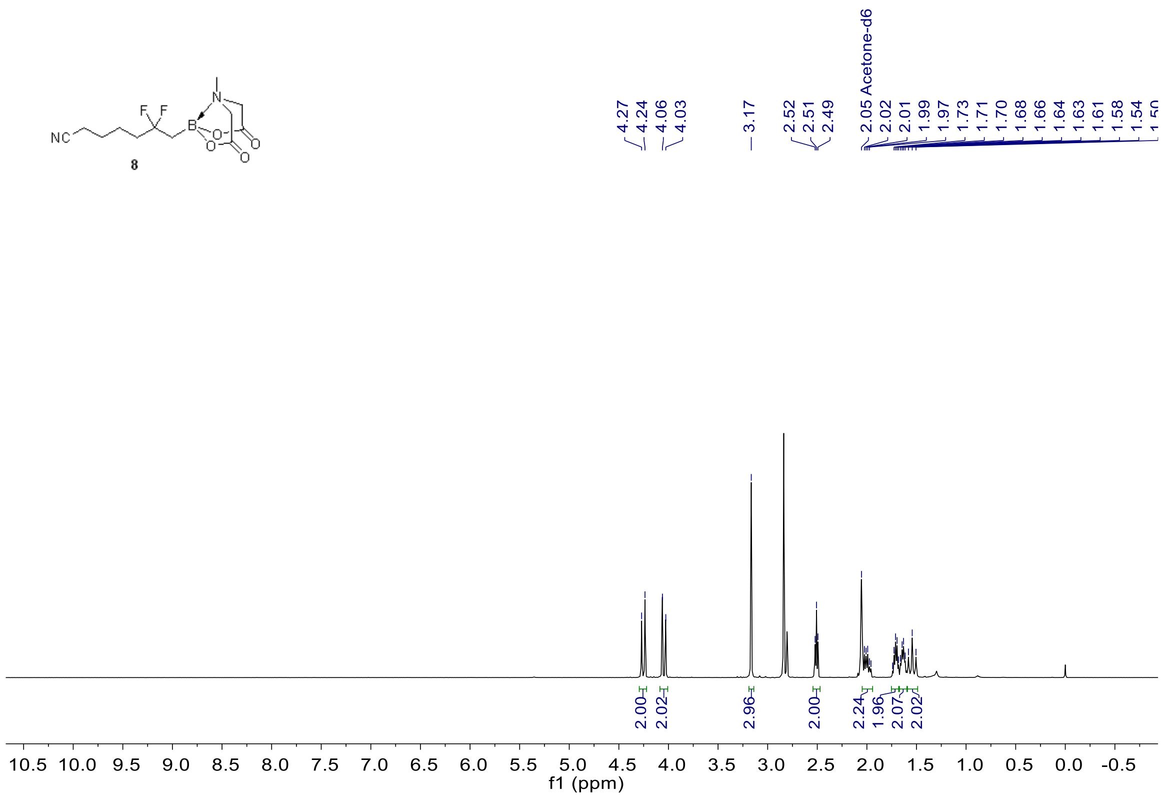
NC
F F
B
N
O
8
4.27
4.24
4.06
4.03
3.17
2.52
2.51
2.49
2.05 Acetone-d6
2.02
2.01
1.99
1.97
1.73
1.71
1.70
1.68
1.66
1.64
1.63
1.61
1.58
1.54
2.00
2.02
2.96
2.00
2.24
1.96
2.07
2.02
10.5 10.0 9.5 9.0 8.5 8.0 7.5 7.0 6.5 6.0 5.5 5.0 4.5 4.0 3.5 3.0 2.5 2.0 1.5 1.0 0.5 0.0 -0.5
f1 (ppm)

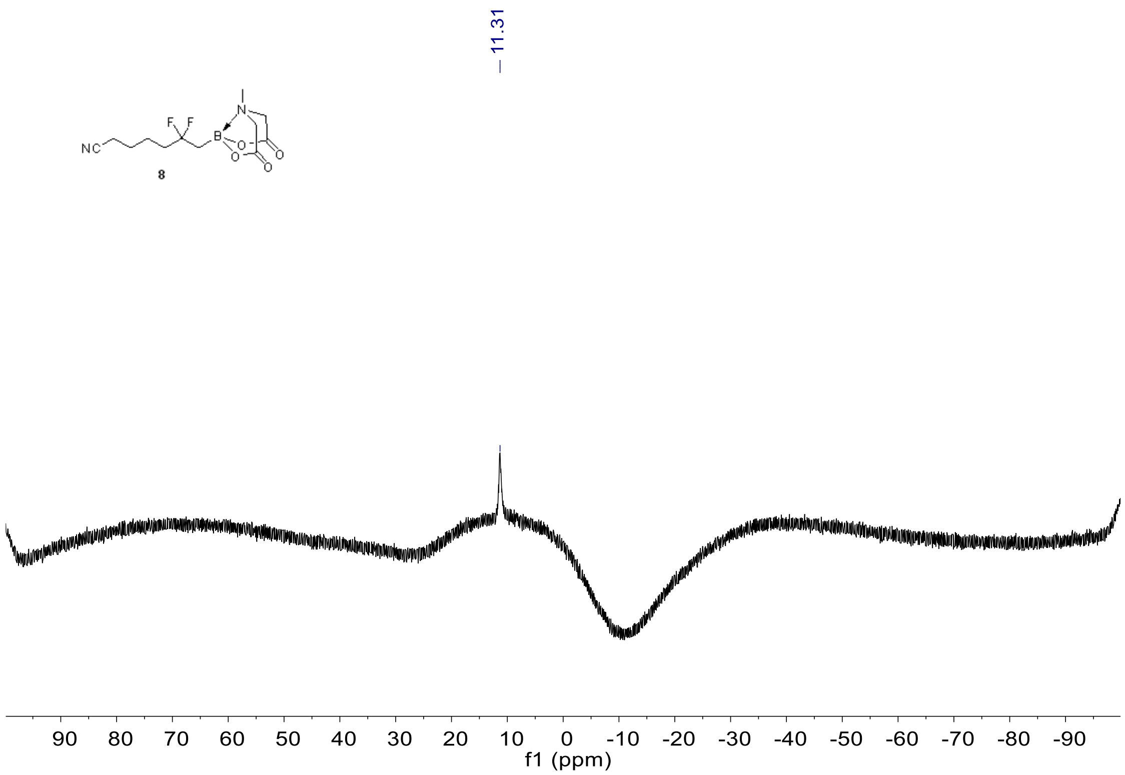
11.31
NC
F F
B
N
O
8
90 80 70 60 50 40 30 20 10 0 -10 -20 -30 -40 -50 -60 -70 -80 -90
f1 (ppm)

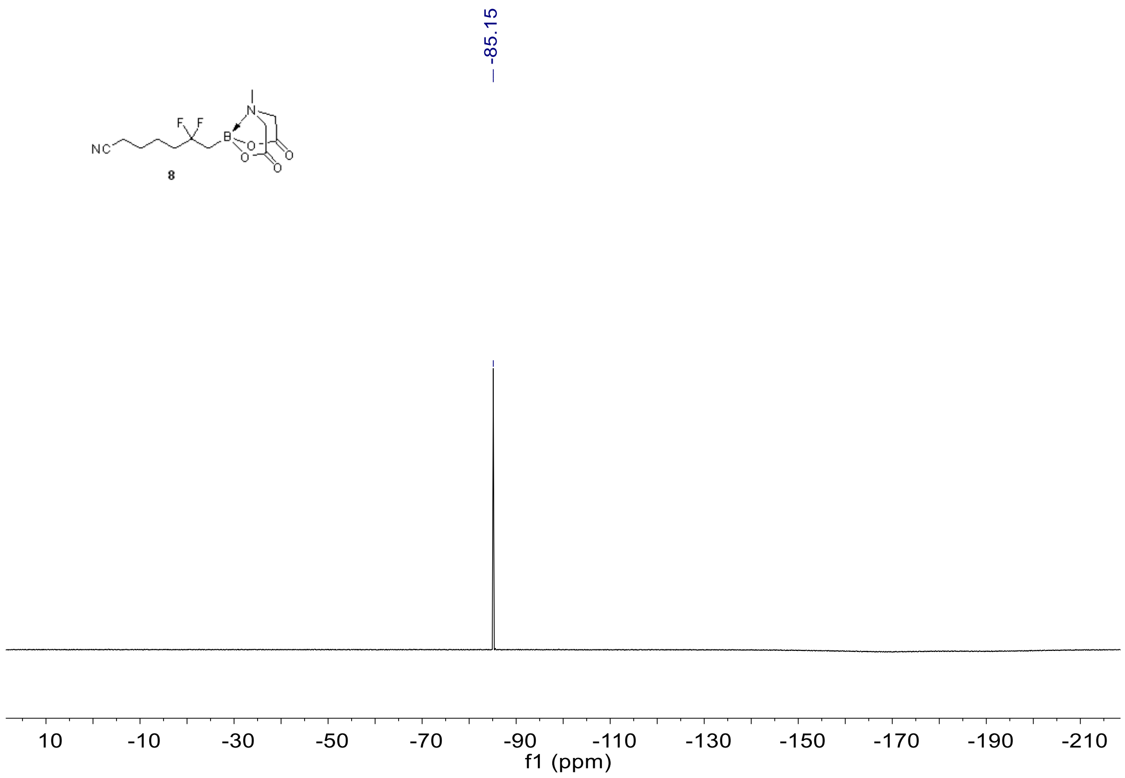

-85.15
NC
F F
B
N
O
8
10 -10 -30 -50 -70 -90 -110 -130 -150 -170 -190 -210
f1 (ppm)

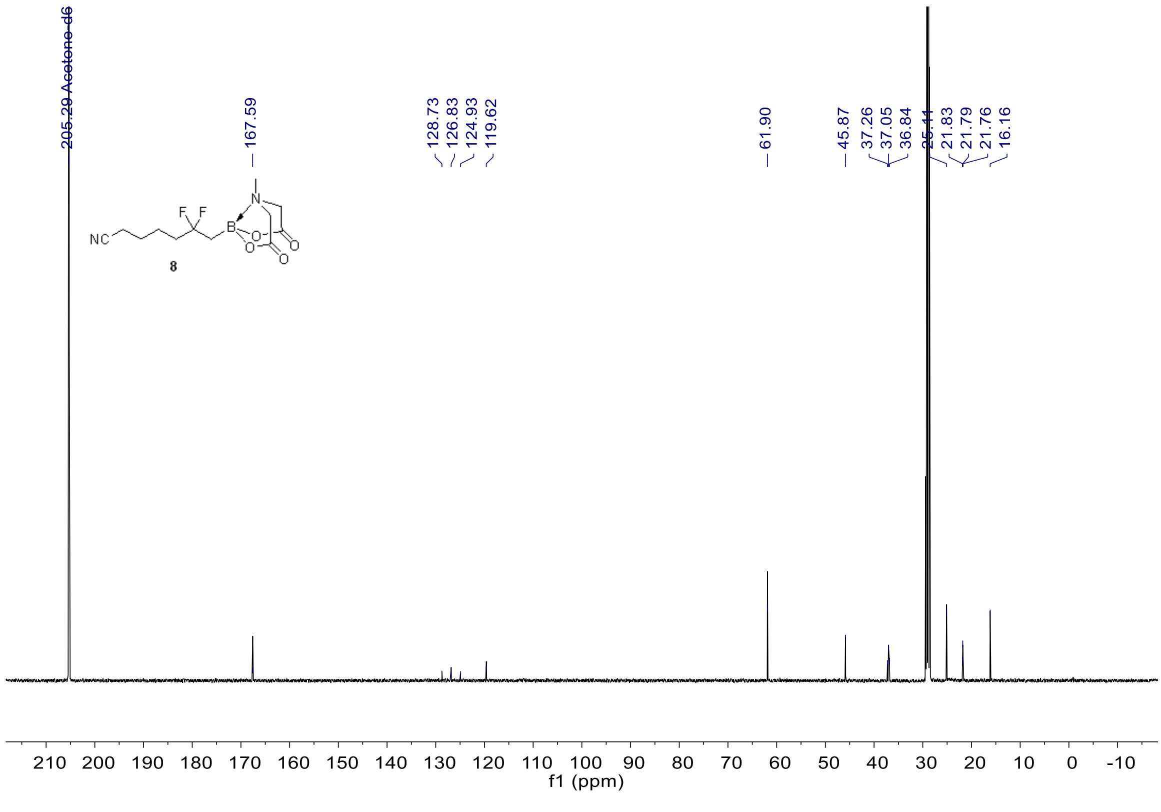

205.29 Acetone-d6
167.59
128.73
126.83
124.93
119.62
61.90
45.87
37.26
37.05
36.84
29.44
21.83
21.79
21.76
16.16
NC
F F
B
N
O
8
210 200 190 180 170 160 150 140 130 120 110 100 90 80 70 60 50 40 30 20 10 0 -10
f1 (ppm)

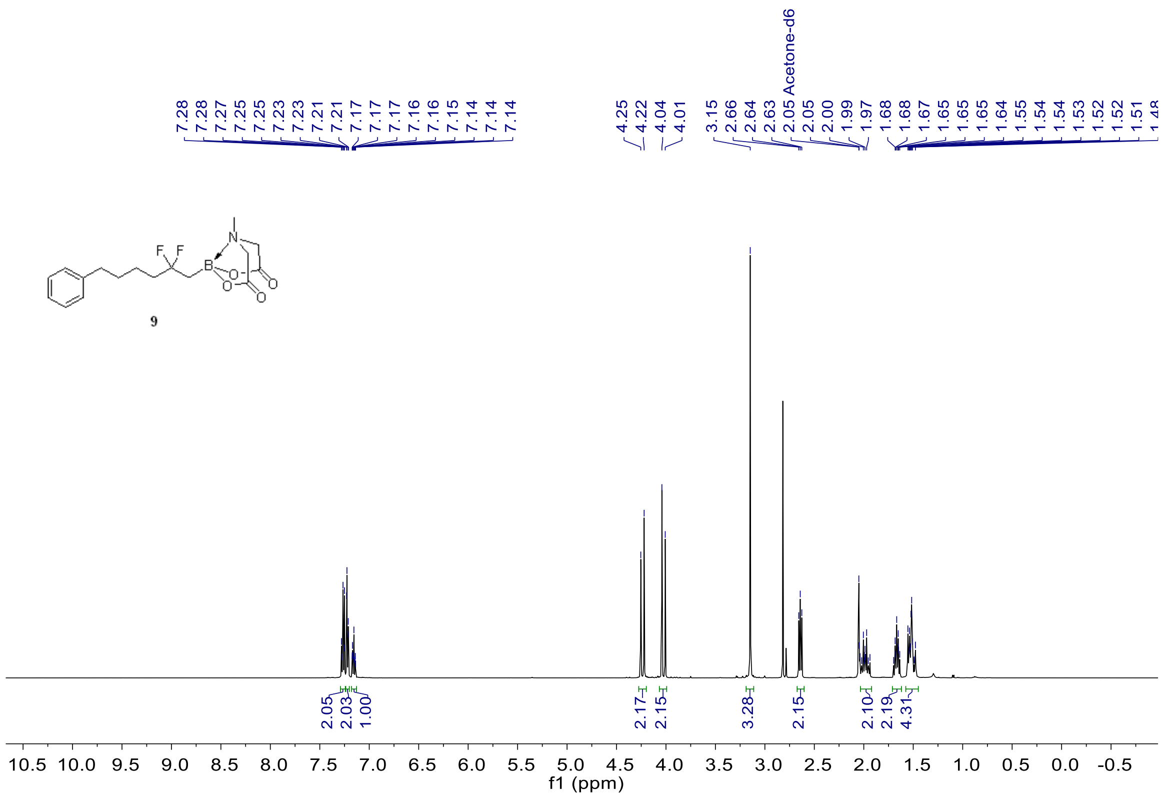

7.28 7.28 7.27 7.25 7.25 7.23 7.23 7.21 7.21 7.17 7.17 7.17 7.16 7.16 7.15 7.14 7.14 7.14
4.25 4.22 4.04 4.01
3.15 2.66 2.64 2.63 2.05 Acetone-d6 2.05 2.00 1.99 1.97 1.68 1.68 1.67 1.65 1.65 1.64 1.55 1.54 1.54 1.53 1.52 1.52 1.51
F F
N
B
O
O
O
O
9
2.05 2.03 1.00
2.17 2.15
3.28
2.15
2.10 2.19 4.31
10.5 10.0 9.5 9.0 8.5 8.0 7.5 7.0 6.5 6.0 5.5 5.0 4.5 4.0 3.5 3.0 2.5 2.0 1.5 1.0 0.5 0.0 -0.5
f1 (ppm)

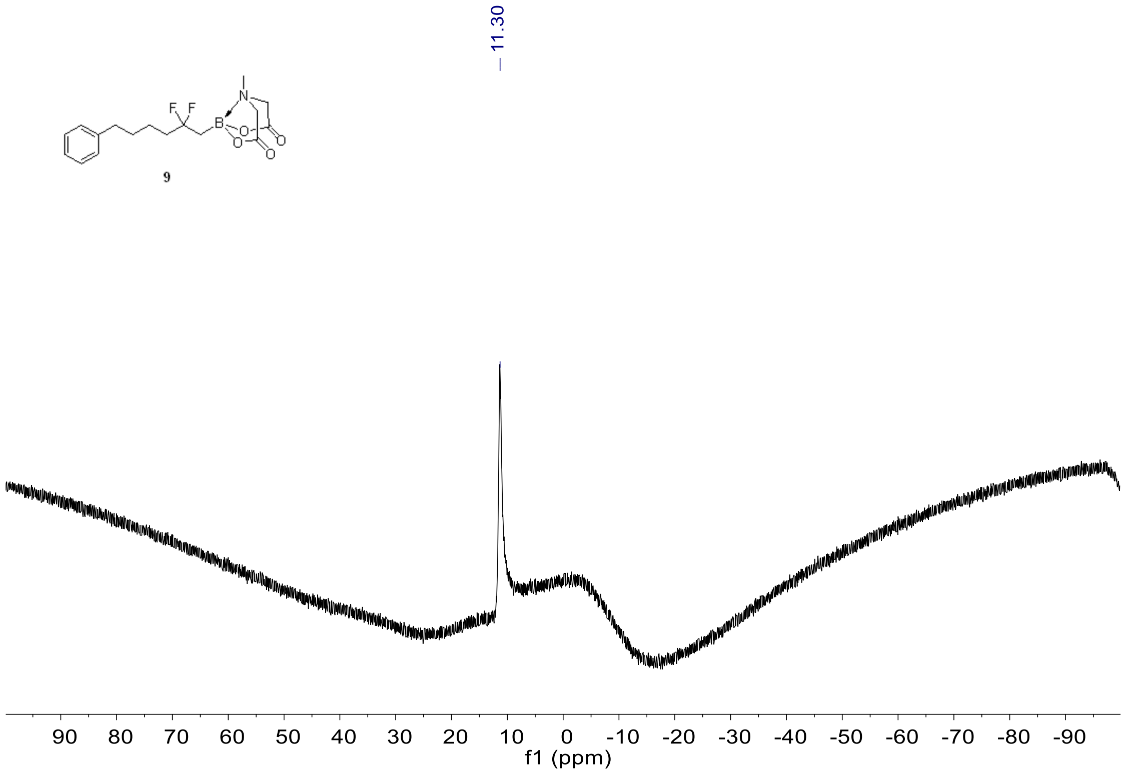

11.30
F F
N
B
O
O
O
O
9
90 80 70 60 50 40 30 20 10 0 -10 -20 -30 -40 -50 -60 -70 -80 -90
f1 (ppm)

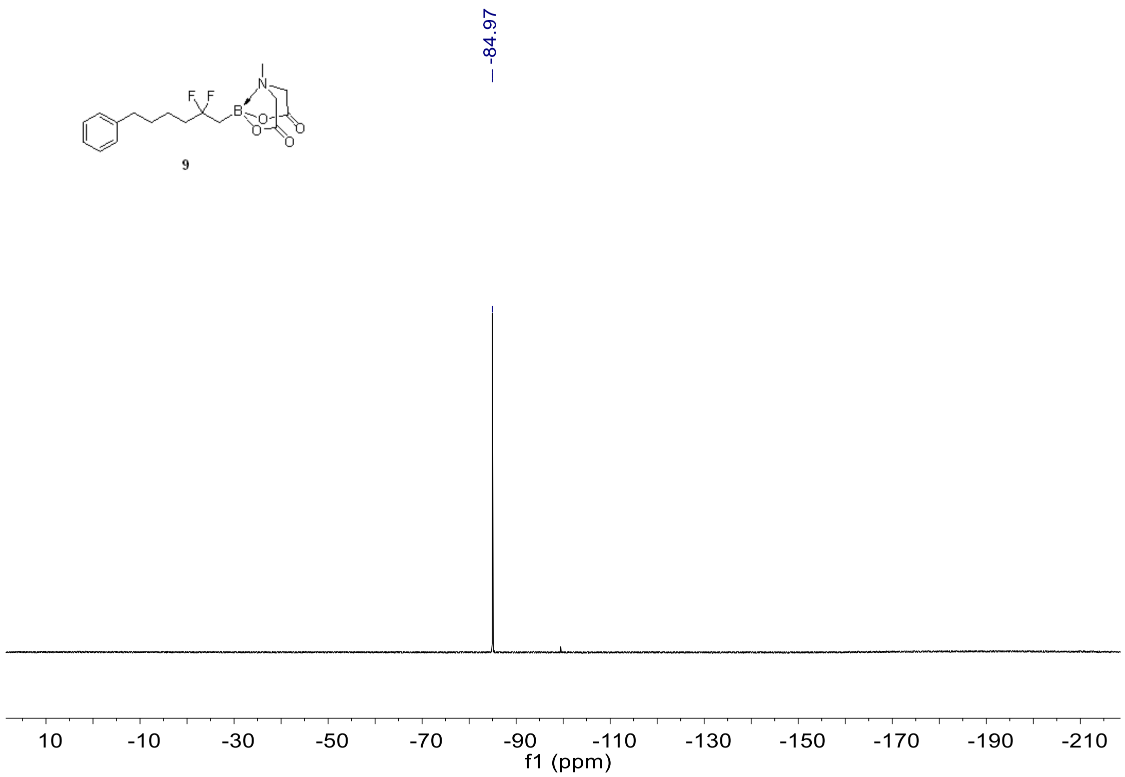

-84.97
F F
B
N
O
9
10 -10 -30 -50 -70 -90 -110 -130 -150 -170 -190 -210
f1 (ppm)

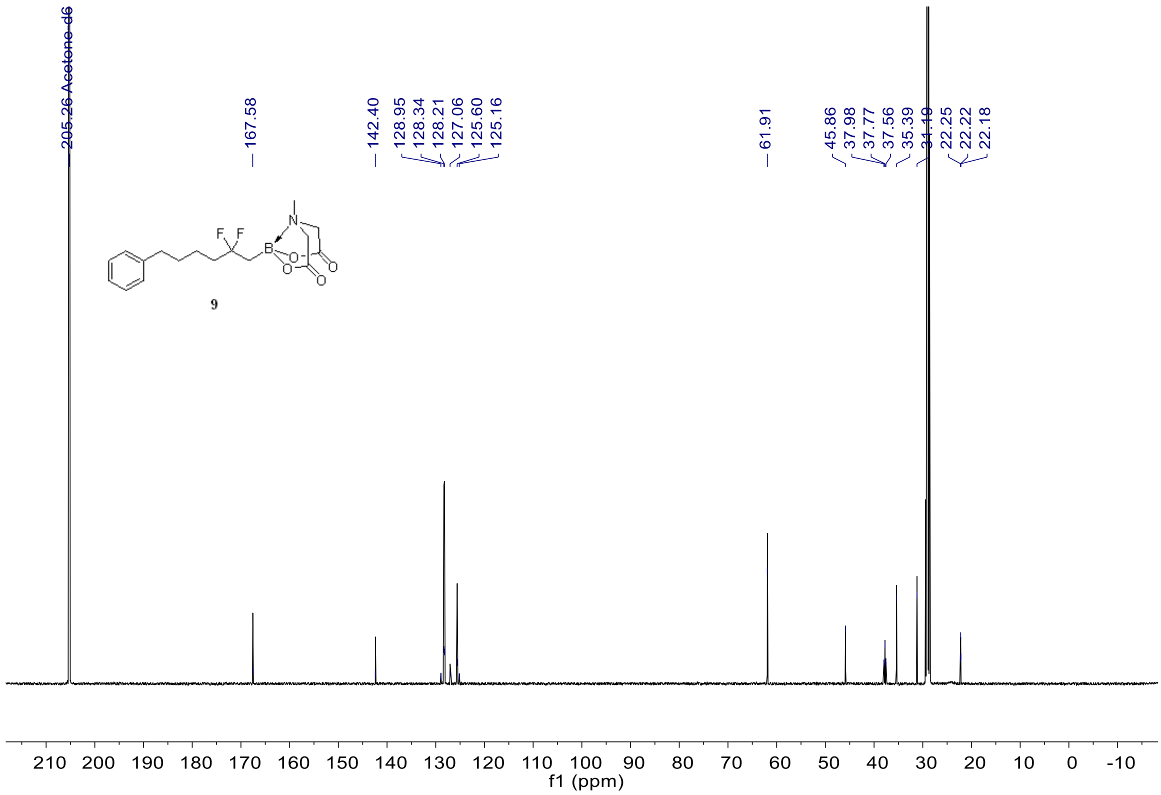

205.26 Acetone-d6
167.58
142.40
128.95
128.34
128.21
127.06
125.60
125.16
61.91
45.86
37.98
37.77
37.56
35.39
31.19
22.25
22.22
22.18
F F
B
N
O
9
210 200 190 180 170 160 150 140 130 120 110 100 90 80 70 60 50 40 30 20 10 0 -10
f1 (ppm)

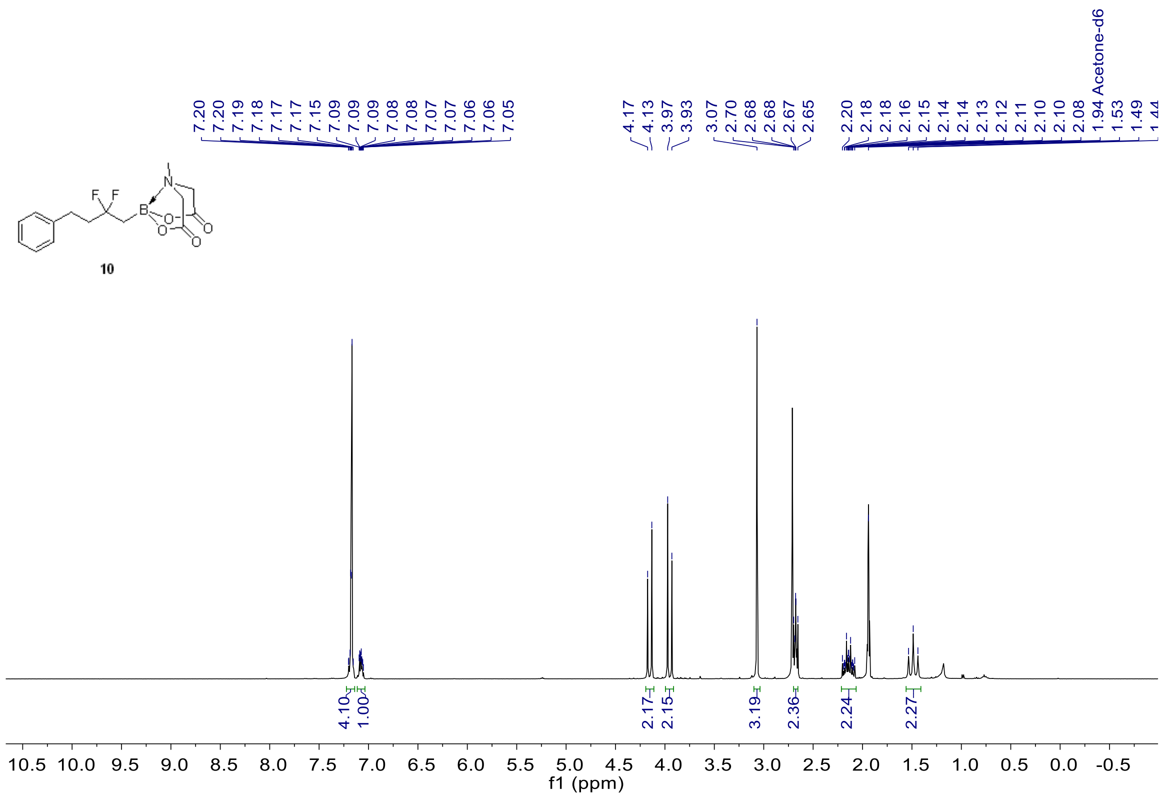
7.20 7.20 7.19 7.18 7.17 7.17 7.15 7.09 7.09 7.08 7.08 7.07 7.07 7.06 7.06 7.05
4.17 4.13 3.97 3.93
3.07 2.70 2.68 2.68 2.67 2.65
2.20 2.18 2.18 2.16 2.15 2.14 2.14 2.13 2.12 2.11 2.10 2.10 2.08 1.94 Acetone-d6 1.53 1.49 1.44
F F B N O O O O
10
4.10 1.00 2.17 2.15 3.19 2.36 2.24 2.27
10.5 10.0 9.5 9.0 8.5 8.0 7.5 7.0 6.5 6.0 5.5 5.0 4.5 4.0 3.5 3.0 2.5 2.0 1.5 1.0 0.5 0.0 -0.5
f1 (ppm)

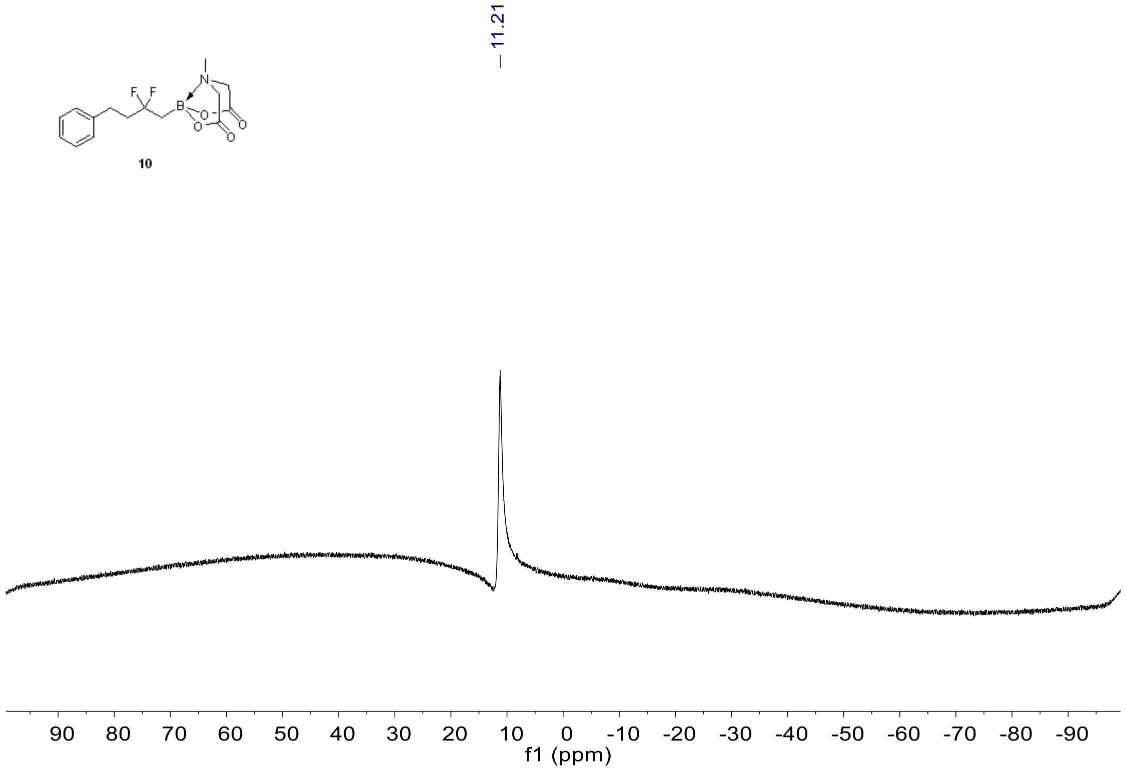
11.21
F F B N O O O O
10
90 80 70 60 50 40 30 20 10 0 -10 -20 -30 -40 -50 -60 -70 -80 -90
f1 (ppm)

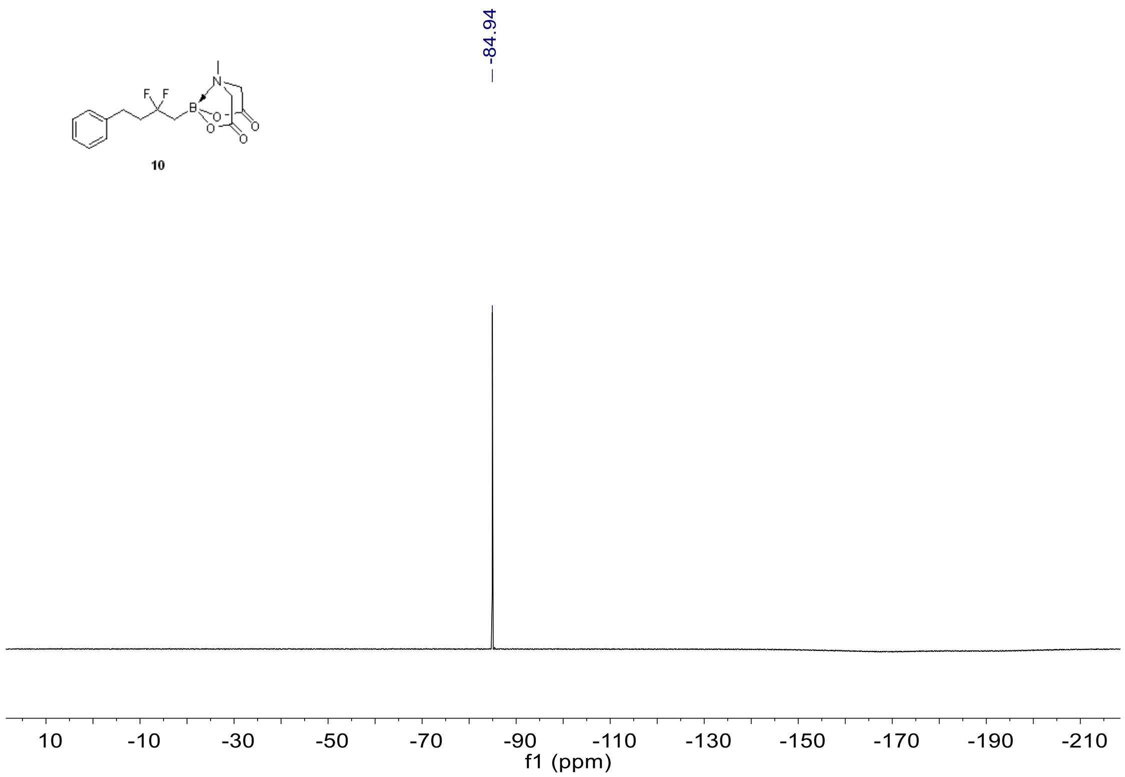

-84.94
10
10 -10 -30 -50 -70 -90 -110 -130 -150 -170 -190 -210
f1 (ppm)

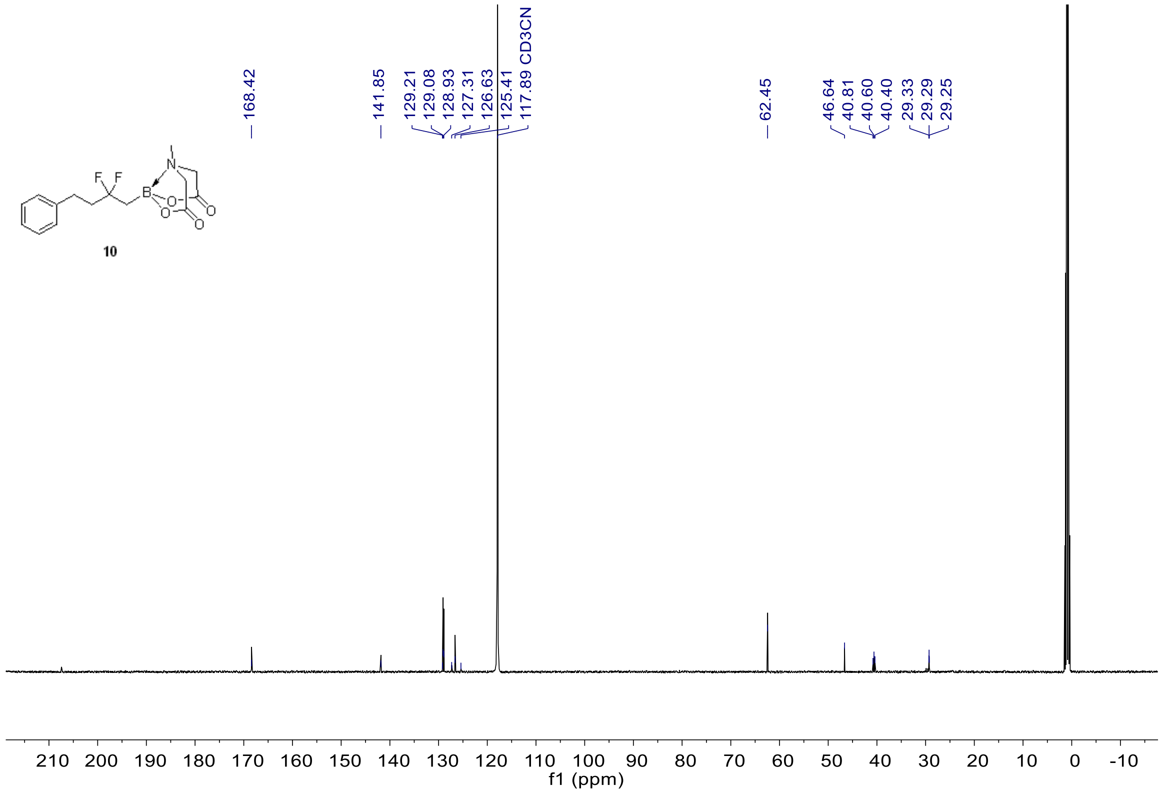

168.42
141.85
129.21
129.08
128.93
127.31
126.63
125.41
117.89 CD3CN
62.45
46.64
40.81
40.60
40.40
29.33
29.29
29.25
10
210 200 190 180 170 160 150 140 130 120 110 100 90 80 70 60 50 40 30 20 10 0 -10
f1 (ppm)

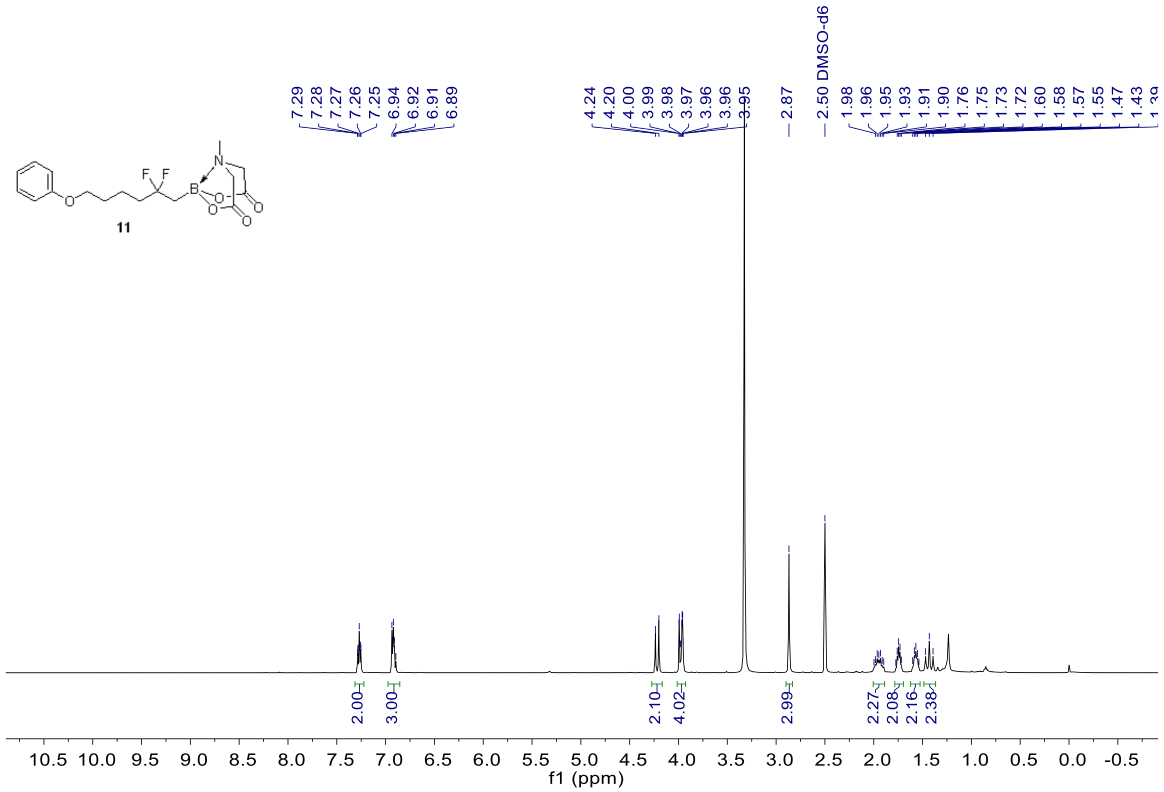
7.29 7.28 7.27 7.26 7.25 6.94 6.92 6.91 6.89
4.24 4.20 4.00 3.99 3.98 3.97 3.96 3.96 3.95
2.87
2.50 DMSO-d6
1.98 1.96 1.95 1.93 1.91 1.90 1.76 1.75 1.73 1.72 1.60 1.58 1.57 1.55 1.47 1.43
2.00 3.00 2.10 4.02 2.99 2.27 2.08 2.16 2.38
10.5 10.0 9.5 9.0 8.5 8.0 7.5 7.0 6.5 6.0 5.5 5.0 4.5 4.0 3.5 3.0 2.5 2.0 1.5 1.0 0.5 0.0 -0.5
f1 (ppm)
F F
B
N
O
O
O
O
O
11

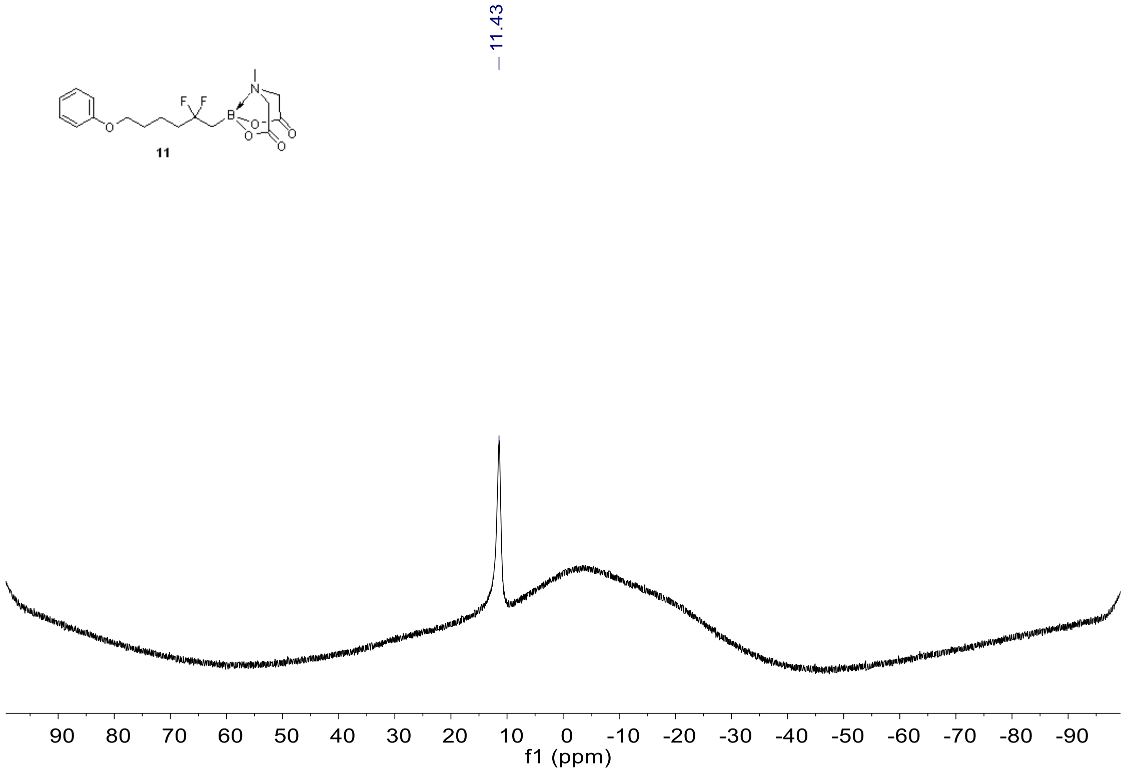
11.43
90 80 70 60 50 40 30 20 10 0 -10 -20 -30 -40 -50 -60 -70 -80 -90
f1 (ppm)
F F
B
N
O
O
O
O
O
11

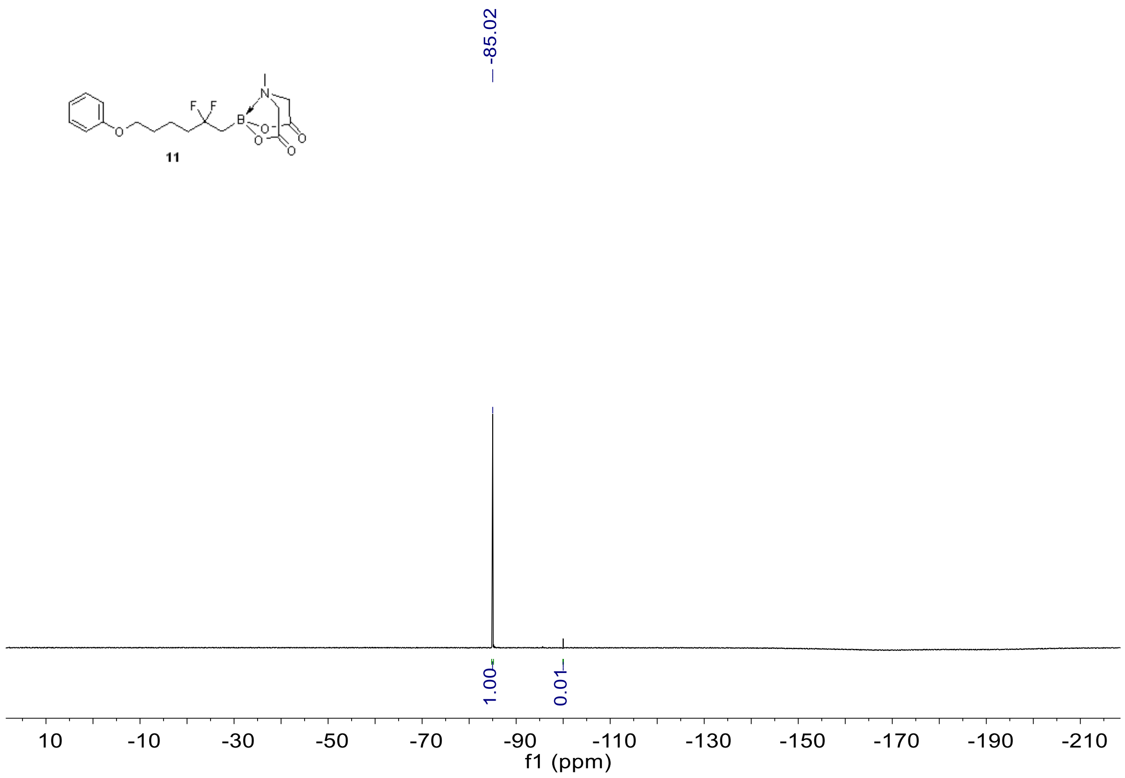
-85.02
11
1.00
0.01
10 -10 -30 -50 -70 -90 -110 -130 -150 -170 -190 -210
f1 (ppm)

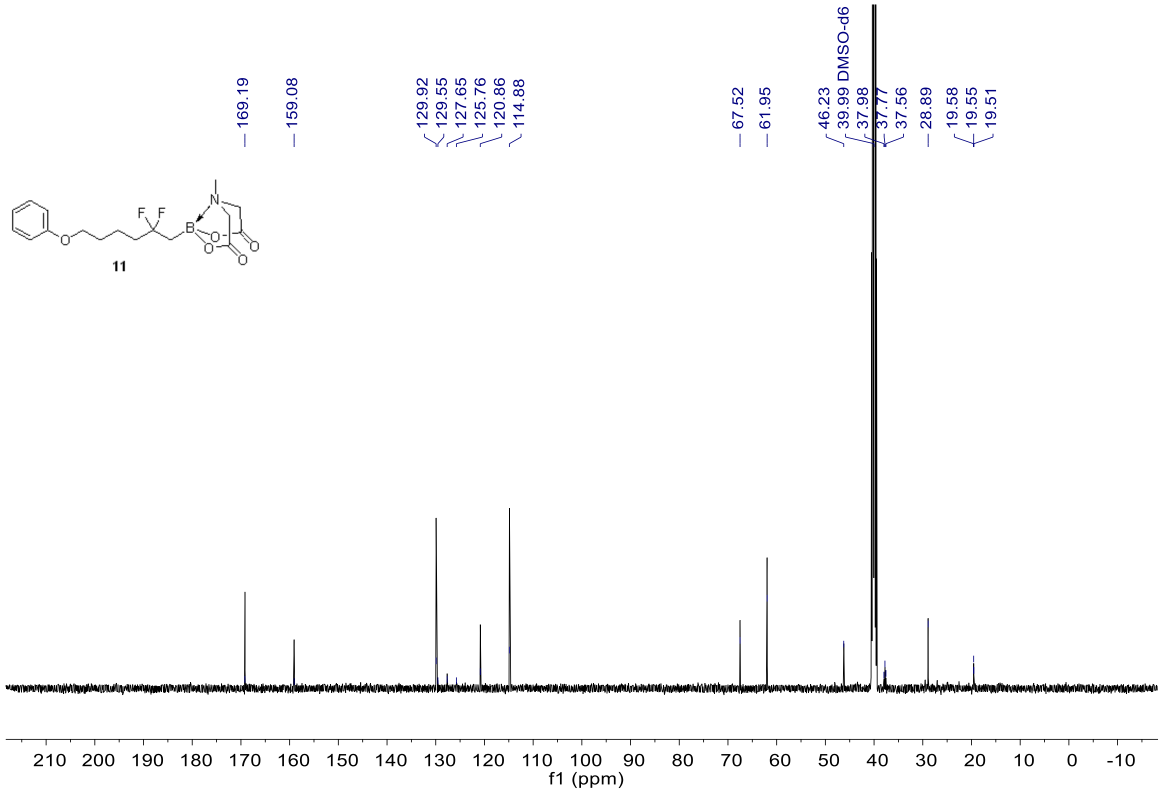
169.19
159.08
129.92
129.55
127.65
125.76
120.86
114.88
67.52
61.95
46.23
39.99 DMSO-d6
37.98
37.77
37.56
28.89
19.58
19.55
19.51
11
210 200 190 180 170 160 150 140 130 120 110 100 90 80 70 60 50 40 30 20 10 0 -10
f1 (ppm)

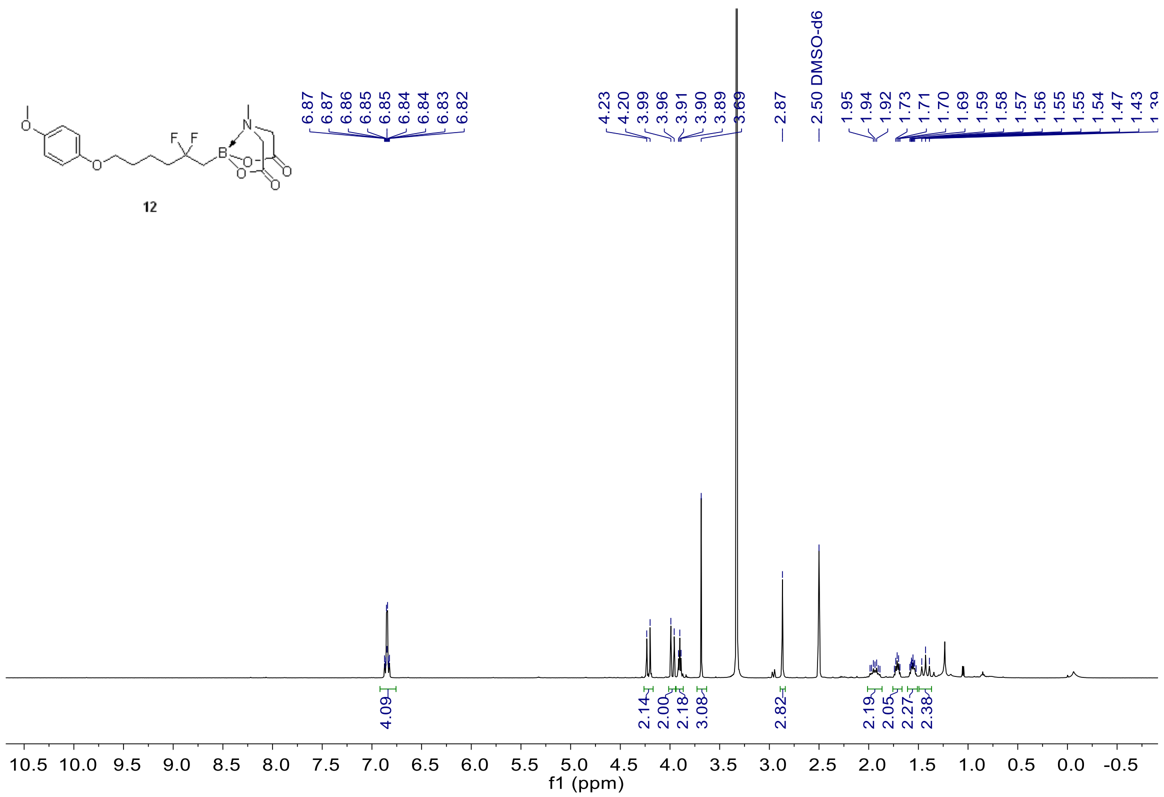
12
6.87
6.87
6.86
6.85
6.85
6.84
6.84
6.83
6.82
4.23
4.20
3.99
3.96
3.91
3.90
3.89
3.69
2.87
2.50 DMSO-d6
1.95
1.94
1.92
1.73
1.71
1.70
1.69
1.59
1.58
1.57
1.56
1.55
1.55
1.54
1.47
1.43
4.09
2.14
2.00
2.18
3.08
2.82
2.19
2.05
2.27
2.38
10.5 10.0 9.5 9.0 8.5 8.0 7.5 7.0 6.5 6.0 5.5 5.0 4.5 4.0 3.5 3.0 2.5 2.0 1.5 1.0 0.5 0.0 -0.5
f1 (ppm)

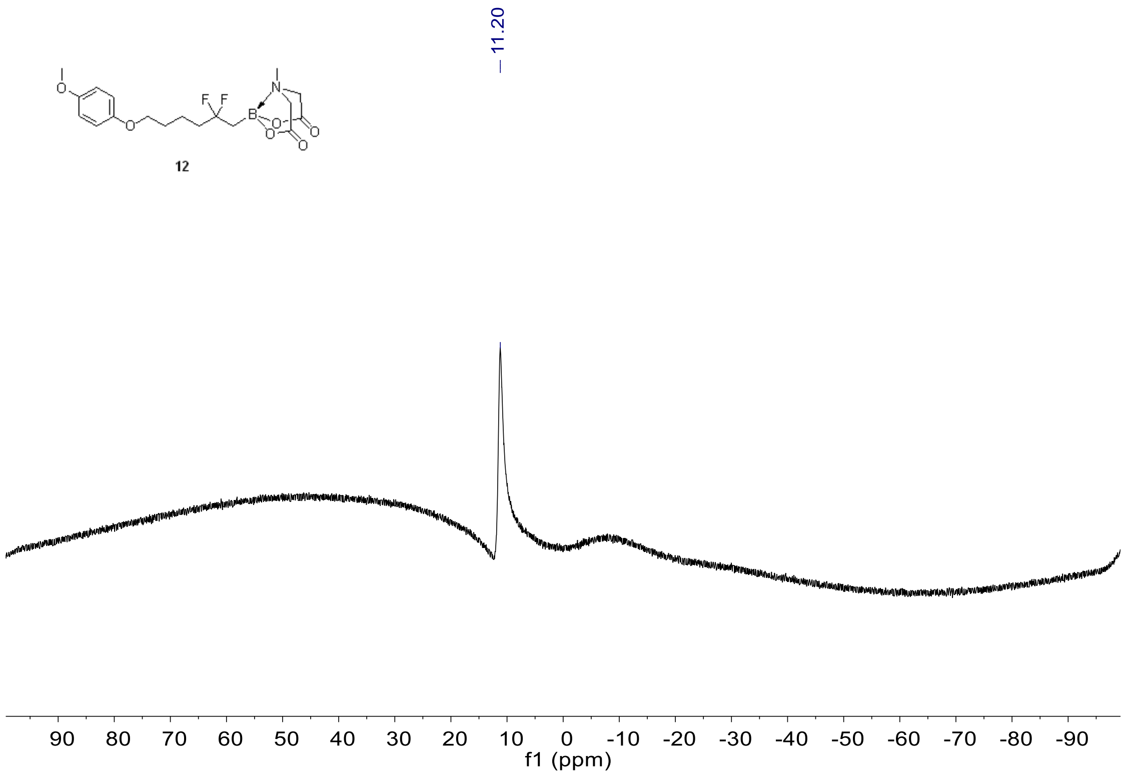
11.20
12
90 80 70 60 50 40 30 20 10 0 -10 -20 -30 -40 -50 -60 -70 -80 -90
f1 (ppm)

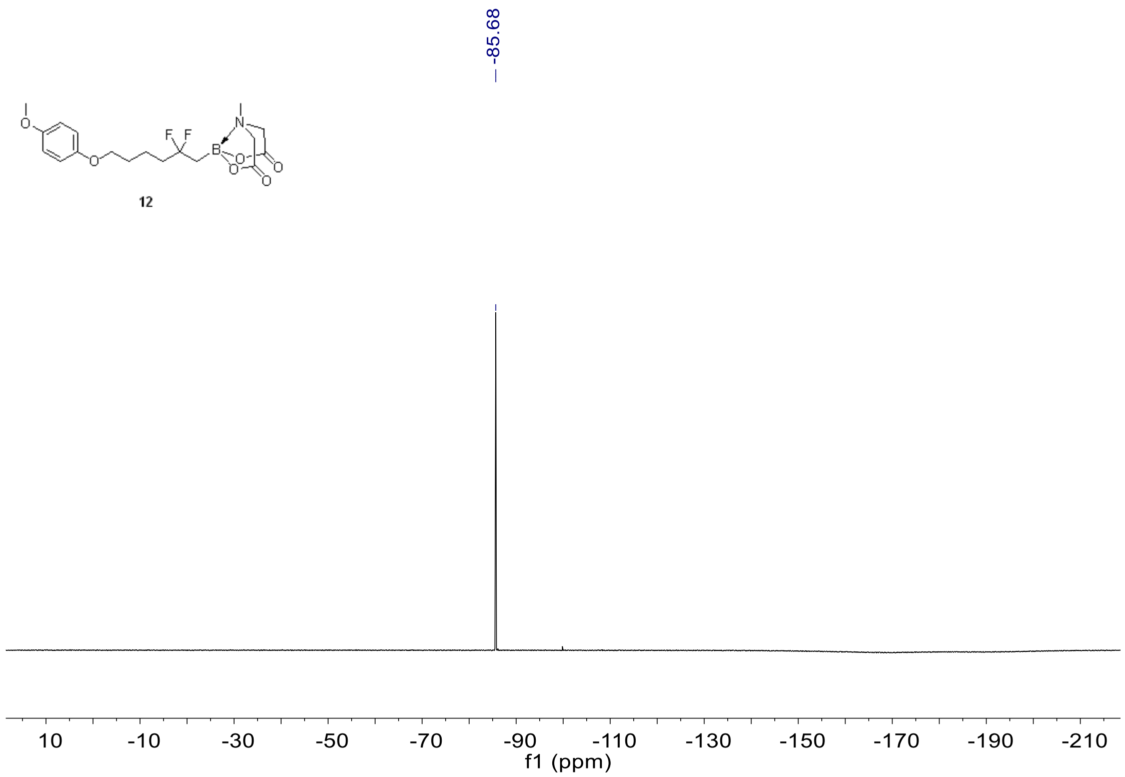
-85.68
F F
B
N
O
12
10 -10 -30 -50 -70 -90 -110 -130 -150 -170 -190 -210
f1 (ppm)

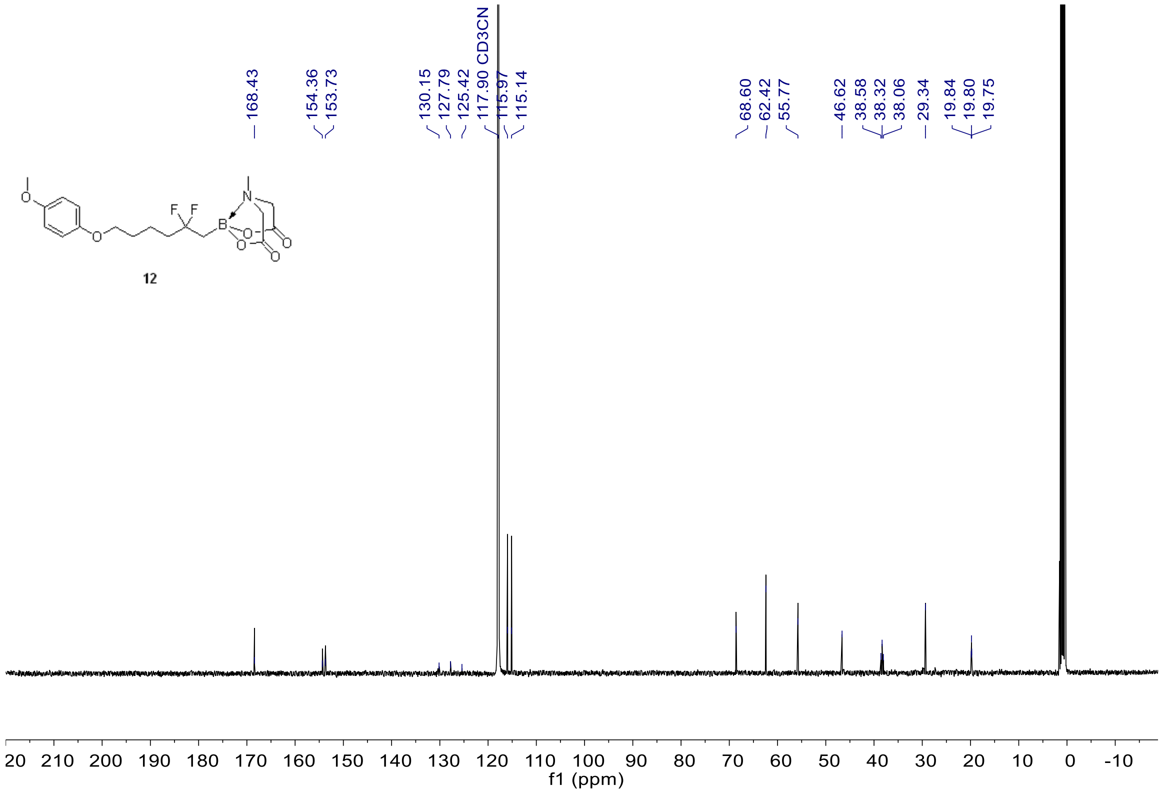
168.43
154.36
153.73
130.15
127.79
125.42
117.90 CD3CN
115.97
115.14
68.60
62.42
55.77
46.62
38.58
38.32
38.06
29.34
19.84
19.80
19.75
F F
B
N
O
12
20 210 200 190 180 170 160 150 140 130 120 110 100 90 80 70 60 50 40 30 20 10 0 -10
f1 (ppm)

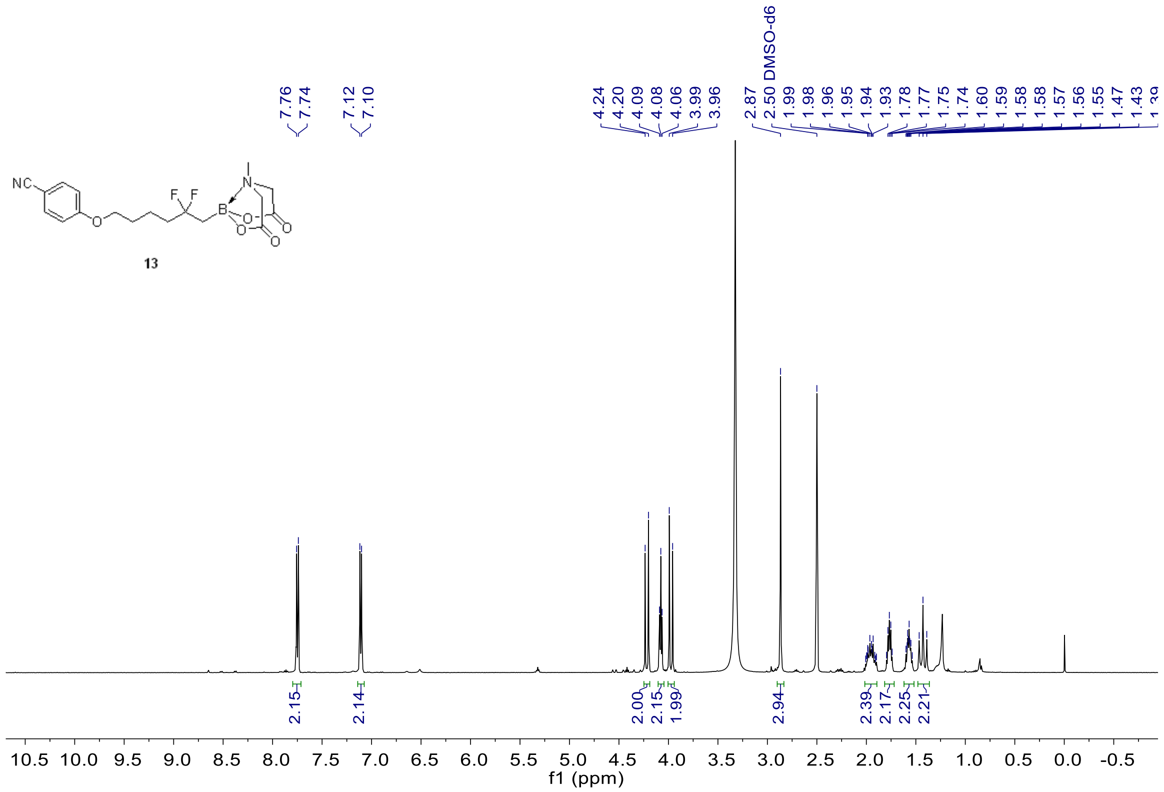
7.76
7.74
7.12
7.10
4.24
4.20
4.09
4.08
4.06
3.99
3.96
2.87
2.50 DMSO-d6
1.99
1.98
1.96
1.95
1.94
1.93
1.78
1.77
1.75
1.74
1.60
1.59
1.58
1.58
1.57
1.56
1.55
1.47
1.43
NC
F F
B
N
O
O
O
O
O
13
2.15
2.14
2.00
2.15
1.99
2.94
2.39
2.17
2.25
2.21
10.5 10.0 9.5 9.0 8.5 8.0 7.5 7.0 6.5 6.0 5.5 5.0 4.5 4.0 3.5 3.0 2.5 2.0 1.5 1.0 0.5 0.0 -0.5
f1 (ppm)

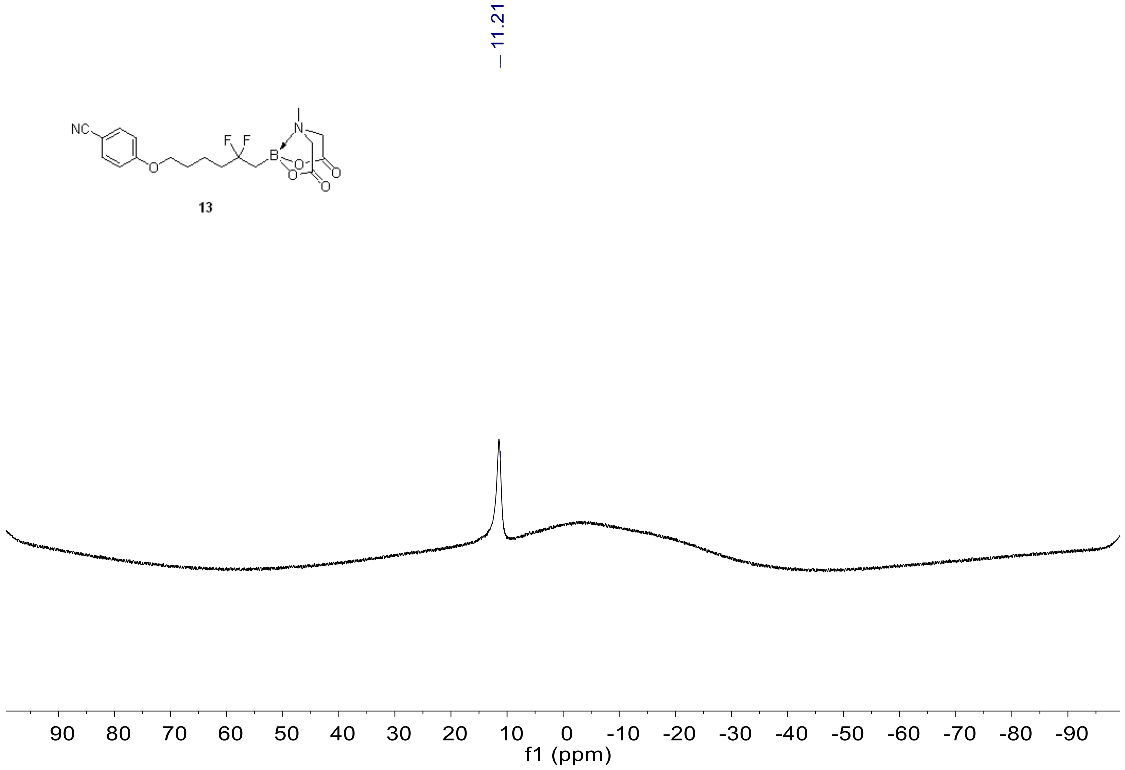
11.21
NC
F F
B
N
O
O
O
O
O
13
90 80 70 60 50 40 30 20 10 0 -10 -20 -30 -40 -50 -60 -70 -80 -90
f1 (ppm)

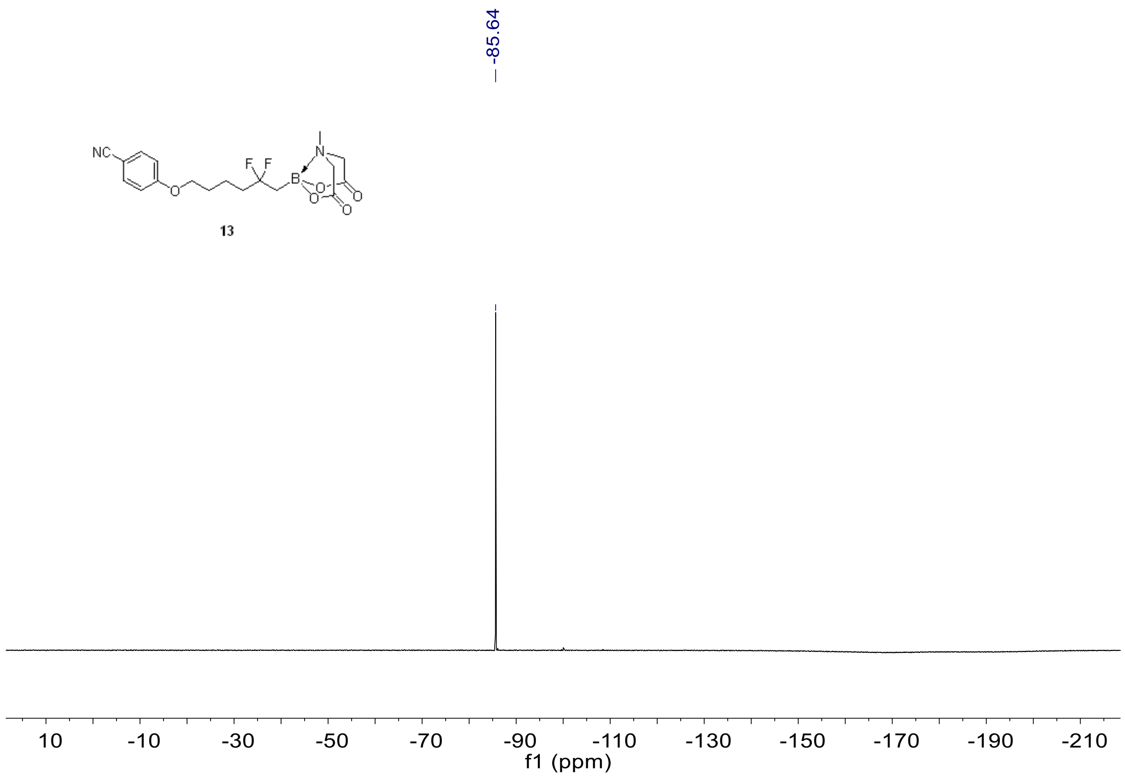
-85.64
NC
F F
B
N
O
13
10 -10 -30 -50 -70 -90 -110 -130 -150 -170 -190 -210
f1 (ppm)

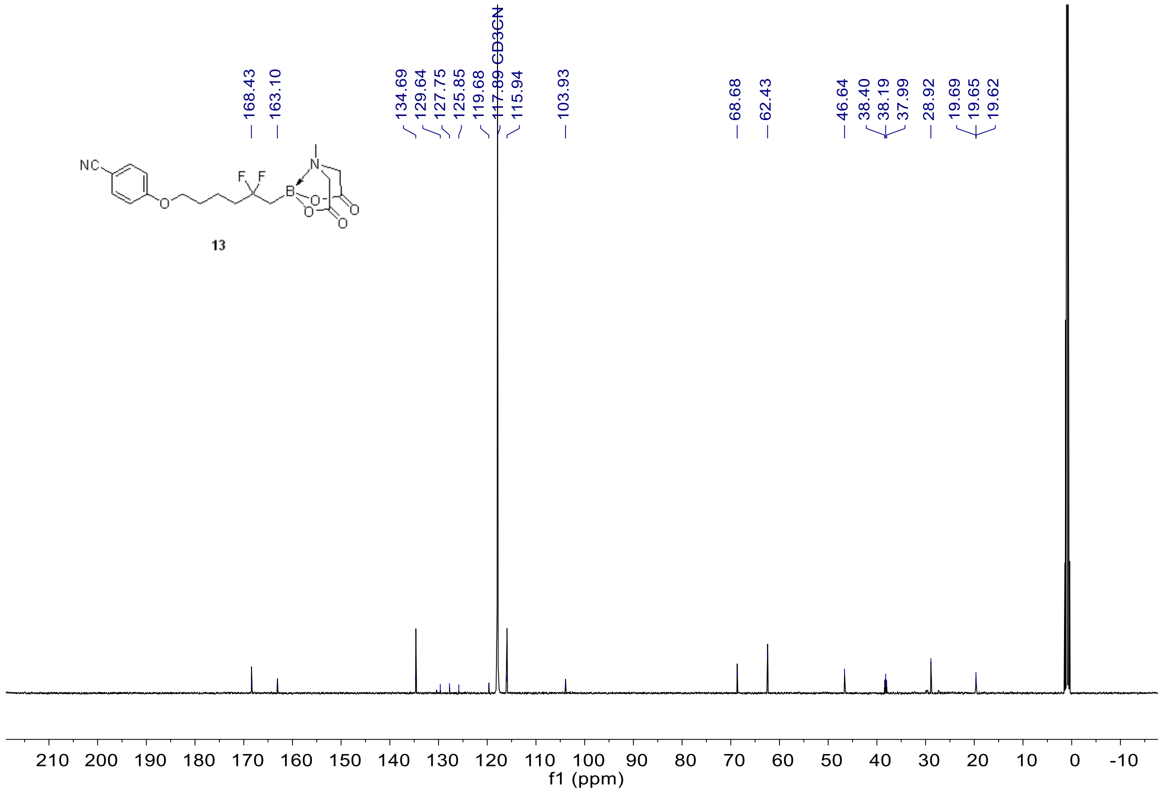
168.43
163.10
134.69
129.64
127.75
125.85
119.68
117.89 CD3CN
115.94
103.93
68.68
62.43
46.64
38.40
38.19
37.99
28.92
19.69
19.65
19.62
NC
F F
B
N
O
13
210 200 190 180 170 160 150 140 130 120 110 100 90 80 70 60 50 40 30 20 10 0 -10
f1 (ppm)

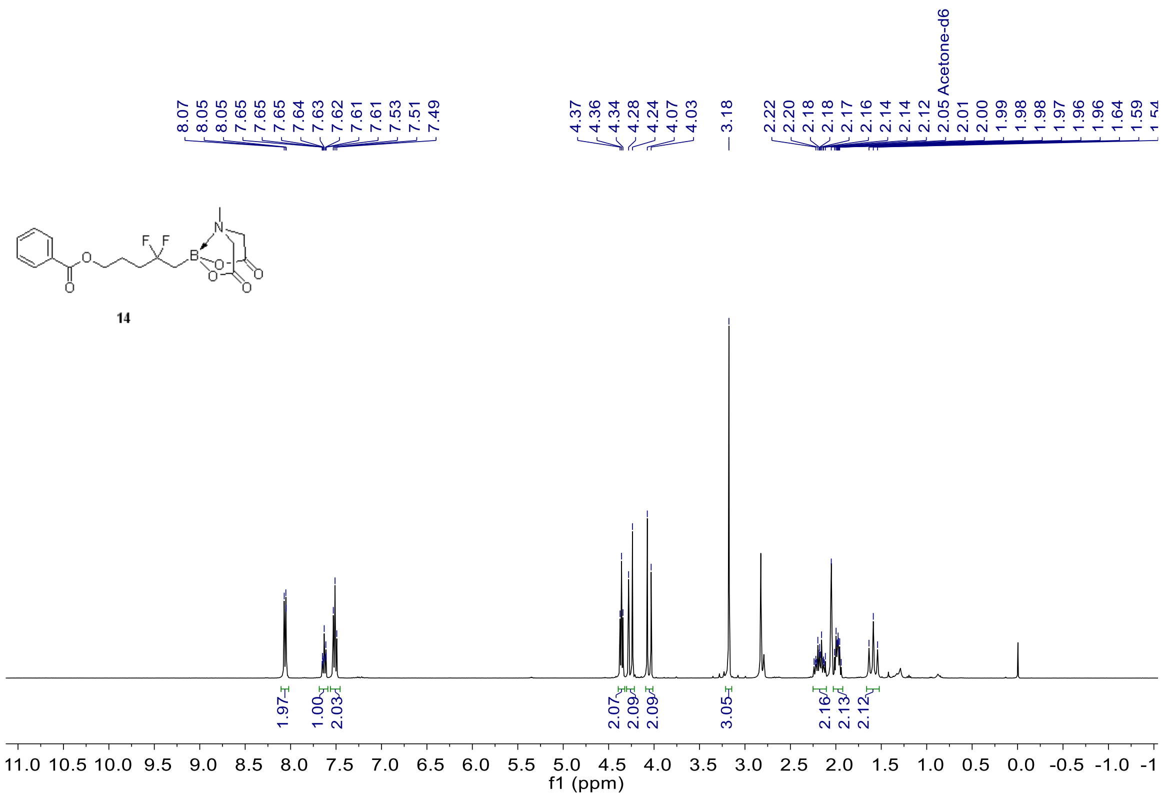
8.07 8.05 8.05 7.65 7.65 7.65 7.64 7.63 7.62 7.61 7.61 7.53 7.51 7.49
4.37 4.36 4.34 4.28 4.24 4.07 4.03
3.18
2.22 2.20 2.18 2.18 2.17 2.16 2.14 2.12 2.05 Acetone-d6 2.01 2.00 1.99 1.98 1.98 1.97 1.96 1.96 1.64 1.59 1.54
14
1.97 1.00 2.03 2.07 2.09 2.09 3.05 2.16 2.13 2.12
11.0 10.5 10.0 9.5 9.0 8.5 8.0 7.5 7.0 6.5 6.0 5.5 5.0 4.5 4.0 3.5 3.0 2.5 2.0 1.5 1.0 0.5 0.0 -0.5 -1.0 -1
f1 (ppm)

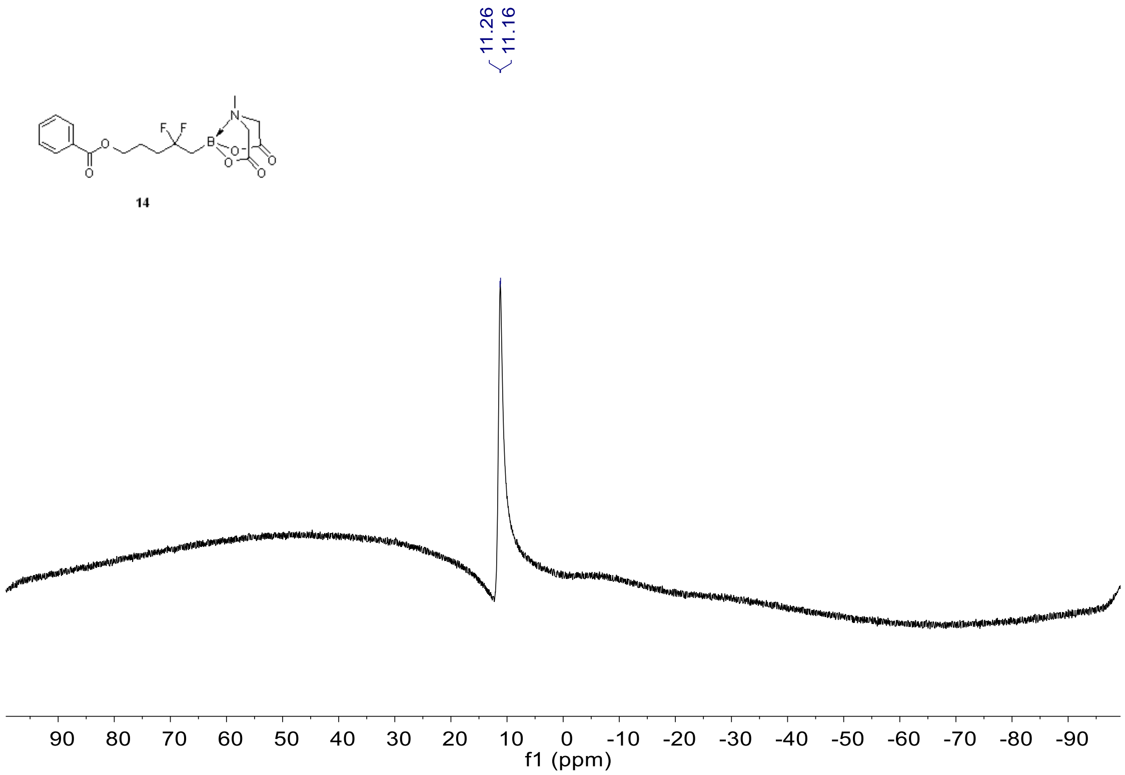
11.26
11.16
14
90 80 70 60 50 40 30 20 10 0 -10 -20 -30 -40 -50 -60 -70 -80 -90
f1 (ppm)

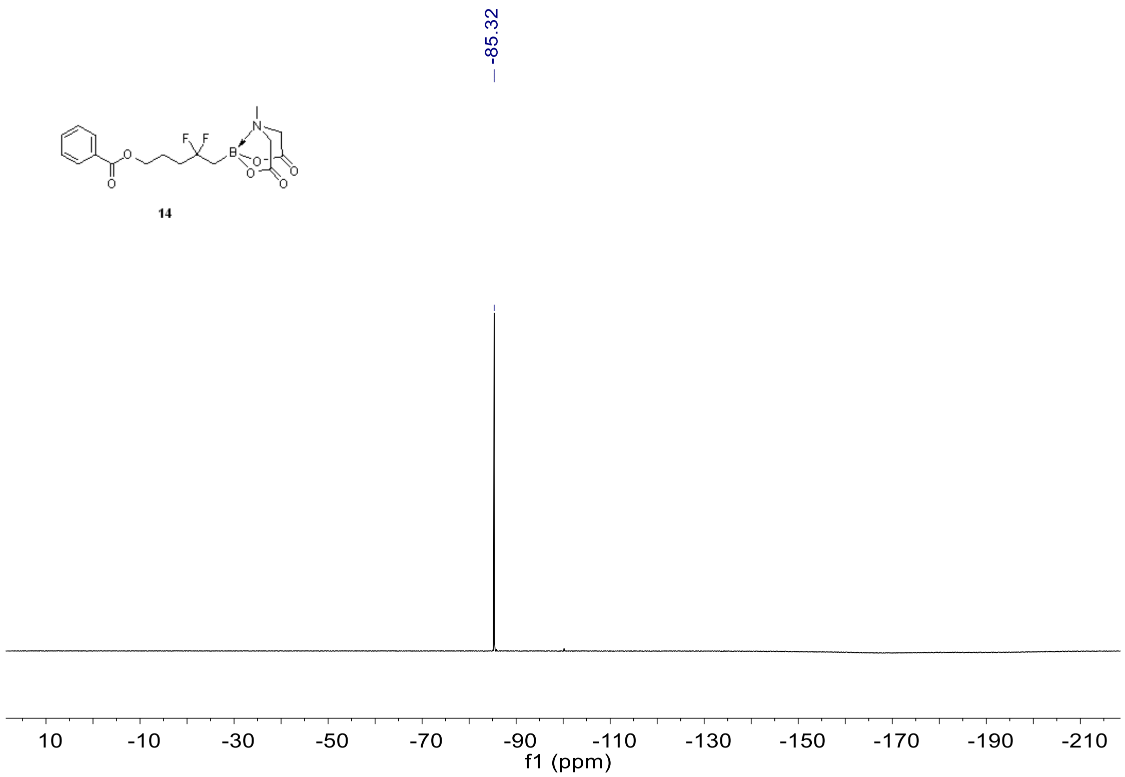

-85.32
14
10 -10 -30 -50 -70 -90 -110 -130 -150 -170 -190 -210
f1 (ppm)

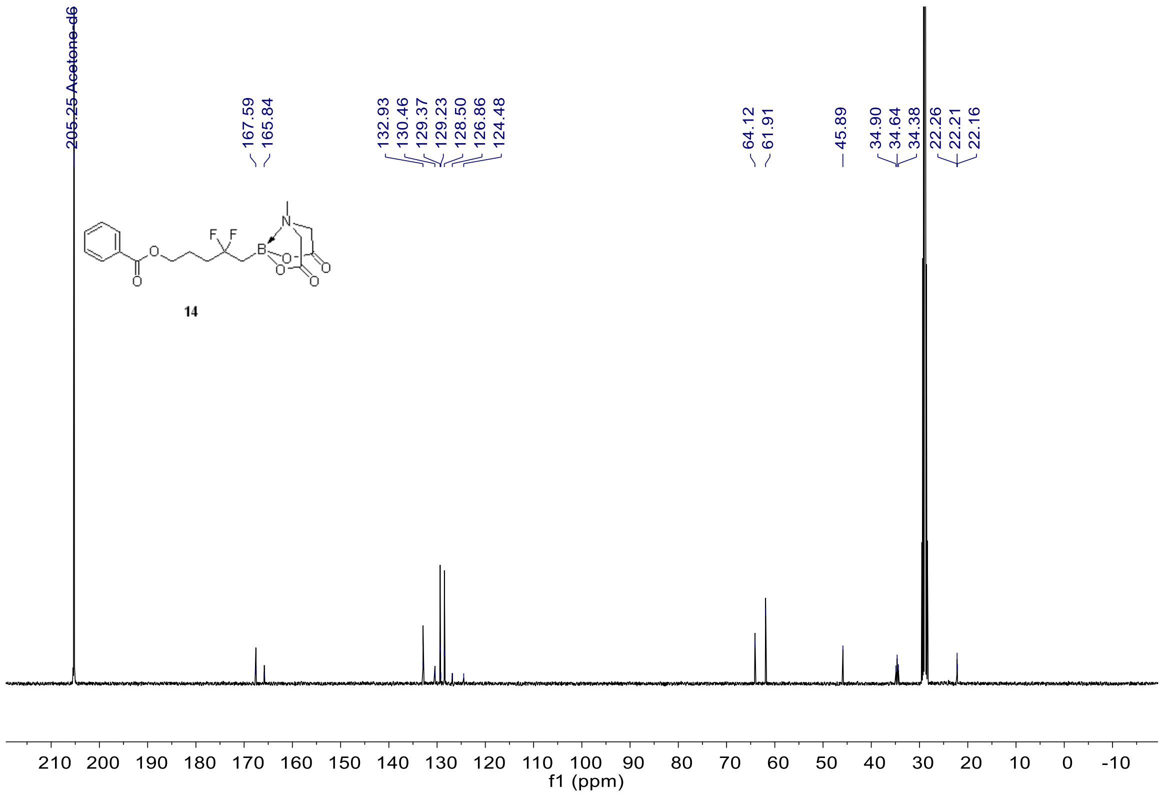

205.25 Acetone-d6
167.59
165.84
132.93
130.46
129.37
129.23
128.50
126.86
124.48
64.12
61.91
45.89
34.90
34.64
34.38
22.26
22.21
22.16
14
210 200 190 180 170 160 150 140 130 120 110 100 90 80 70 60 50 40 30 20 10 0 -10
f1 (ppm)

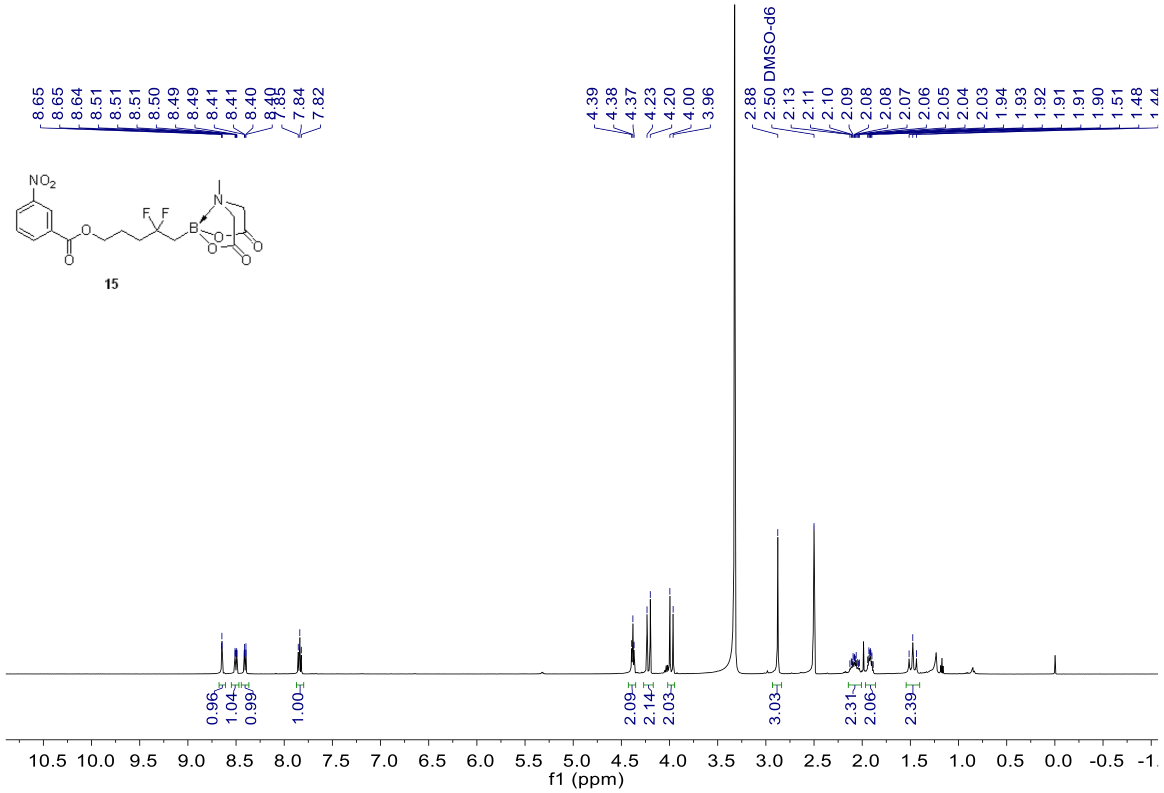

8.65
8.65
8.64
8.51
8.51
8.50
8.49
8.49
8.41
8.41
8.40
8.40
7.85
7.84
7.82
4.39
4.38
4.37
4.23
4.20
4.00
3.96
2.88
2.50 DMSO-d6
2.13
2.11
2.10
2.09
2.08
2.07
2.06
2.05
2.04
2.03
1.94
1.93
1.92
1.91
1.91
1.90
1.51
1.48
NO2
F F
B
N
O
O
O
O
O
15
0.96
1.04
0.99
1.00
2.09
2.14
2.03
3.03
2.31
2.06
2.39
10.5 10.0 9.5 9.0 8.5 8.0 7.5 7.0 6.5 6.0 5.5 5.0 4.5 4.0 3.5 3.0 2.5 2.0 1.5 1.0 0.5 0.0 -0.5
f1 (ppm)

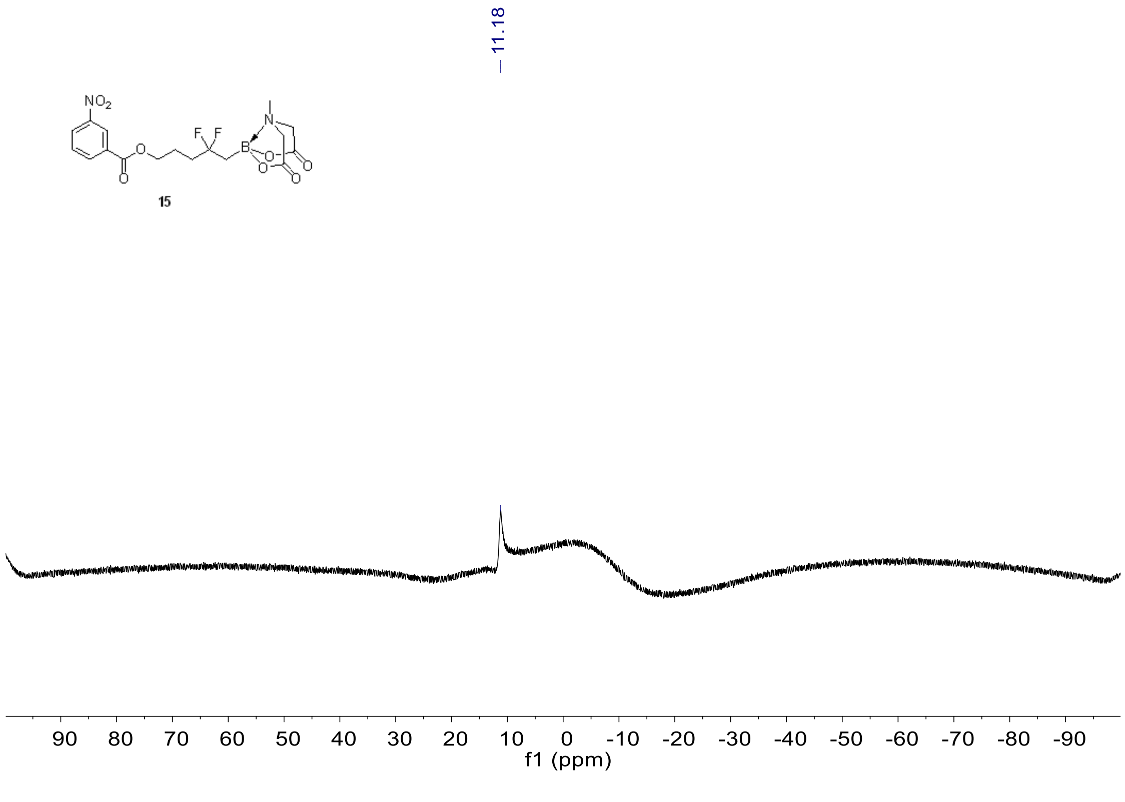

11.18
NO2
F F
B
N
O
O
O
O
O
15
90 80 70 60 50 40 30 20 10 0 -10 -20 -30 -40 -50 -60 -70 -80 -90
f1 (ppm)

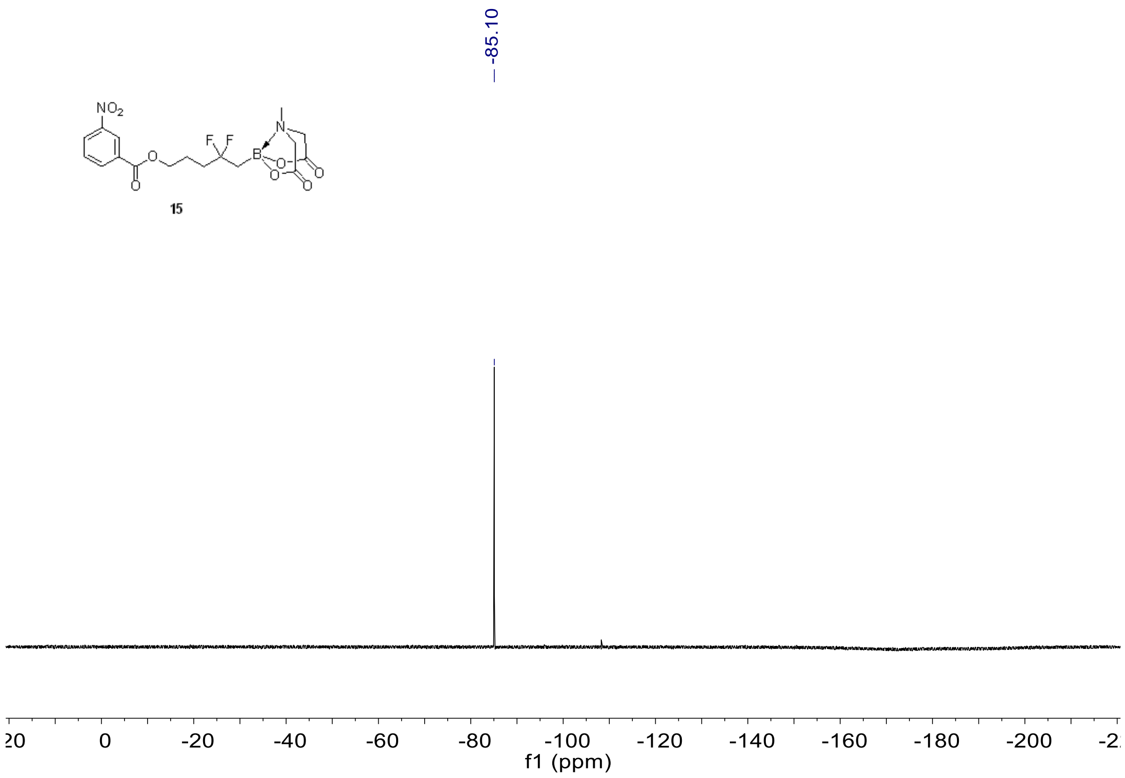

-85.10
NO2
F F
N
B
O
O
O
O
O
15
20
0
-20
-40
-60
-80
-100
-120
-140
-160
-180
-200
f1 (ppm)

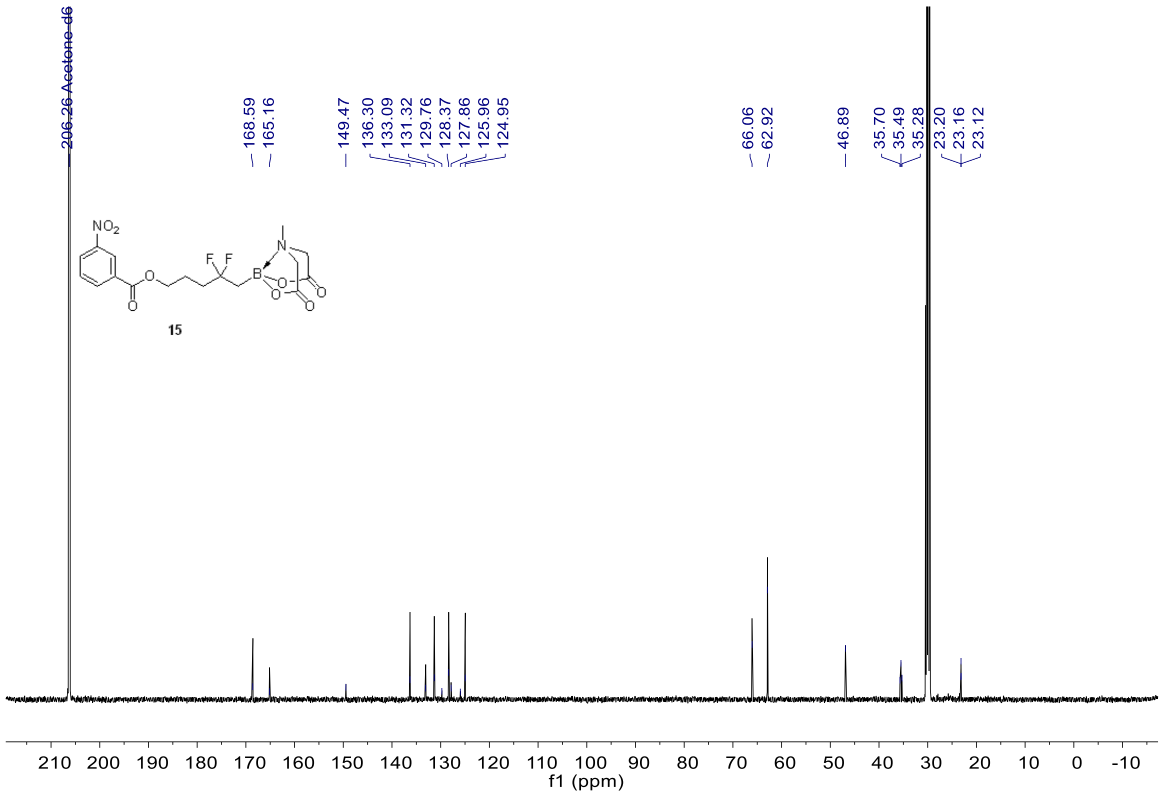

206.26 Acetone-d6
168.59
165.16
149.47
136.30
133.09
131.32
129.76
128.37
127.86
125.96
124.95
66.06
62.92
46.89
35.70
35.49
35.28
23.20
23.16
23.12
NO2
F F
N
B
O
O
O
O
O
15
210 200 190 180 170 160 150 140 130 120 110 100 90 80 70 60 50 40 30 20 10 0 -10
f1 (ppm)

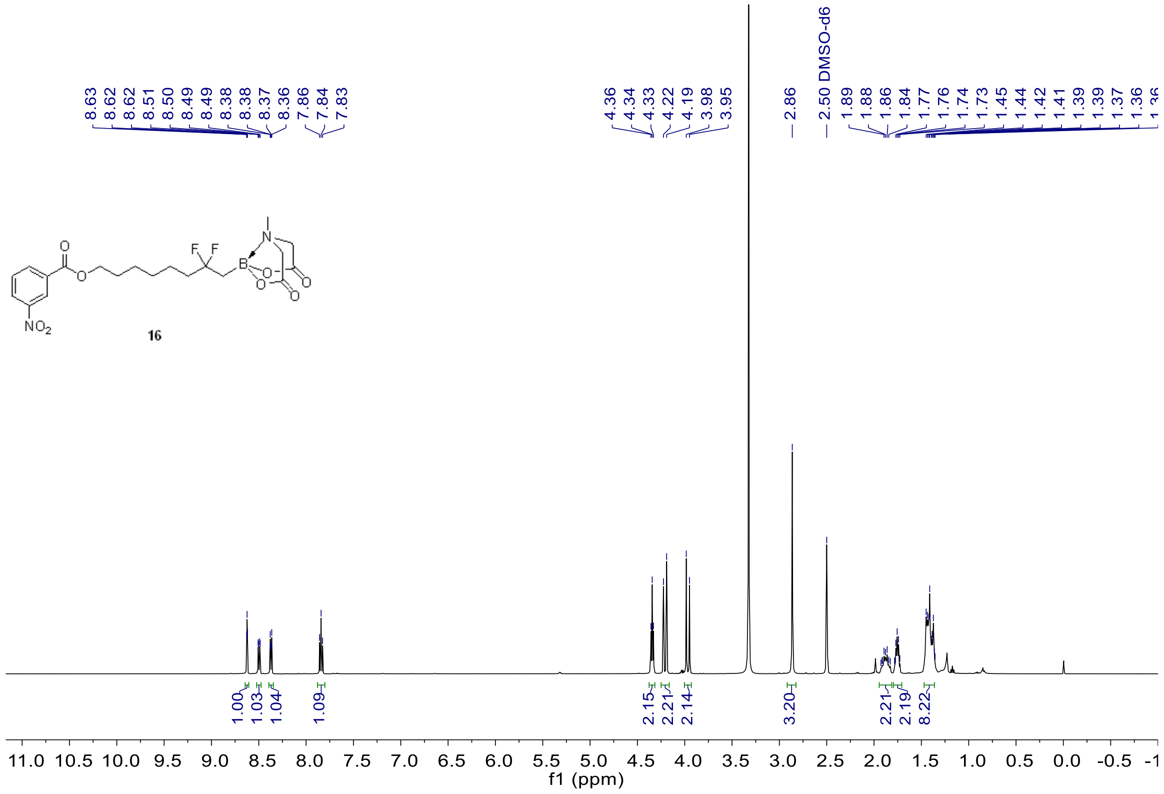
8.63
8.62
8.62
8.51
8.50
8.49
8.49
8.38
8.38
8.37
8.36
7.86
7.84
7.83
4.36
4.34
4.33
4.22
4.19
3.98
3.95
2.86
2.50 DMSO-d6
1.89
1.88
1.86
1.84
1.77
1.76
1.74
1.73
1.45
1.44
1.42
1.41
1.39
1.37
1.36
NO2
16
1.00
1.03
1.04
1.09
2.15
2.21
2.14
3.20
2.21
2.19
8.22
11.0 10.5 10.0 9.5 9.0 8.5 8.0 7.5 7.0 6.5 6.0 5.5 5.0 4.5 4.0 3.5 3.0 2.5 2.0 1.5 1.0 0.5 0.0 -0.5
f1 (ppm)

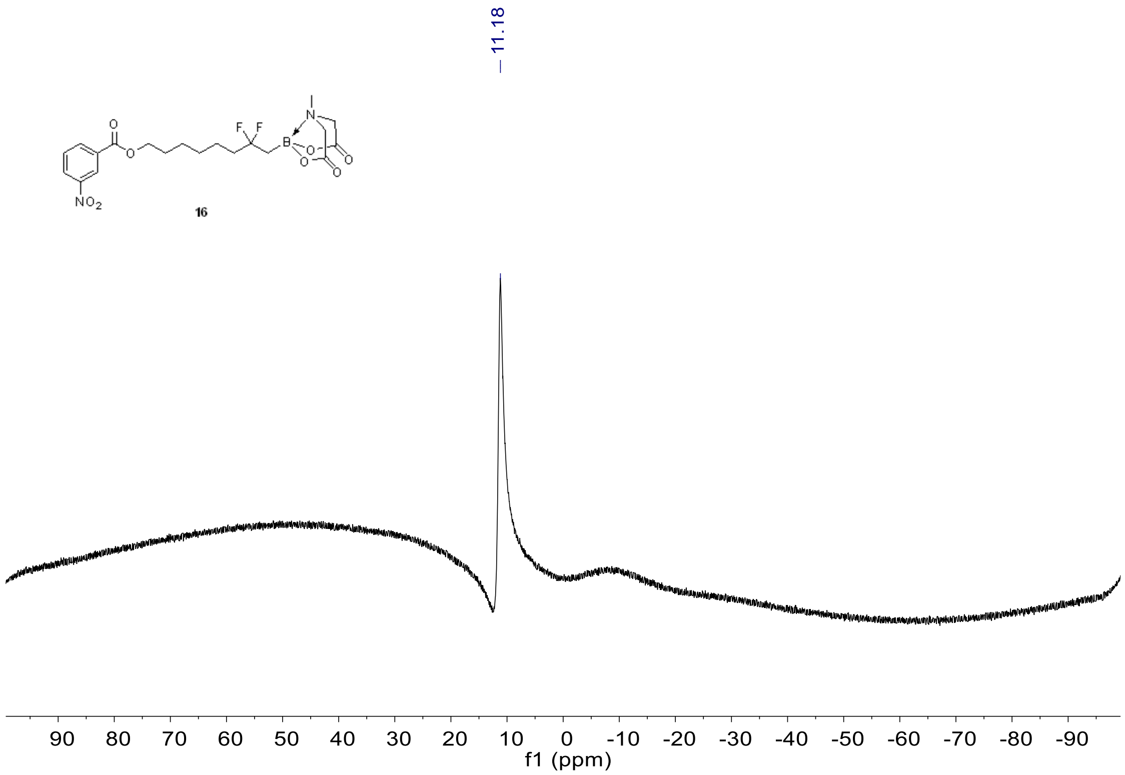
11.18
NO2
16
90 80 70 60 50 40 30 20 10 0 -10 -20 -30 -40 -50 -60 -70 -80 -90
f1 (ppm)

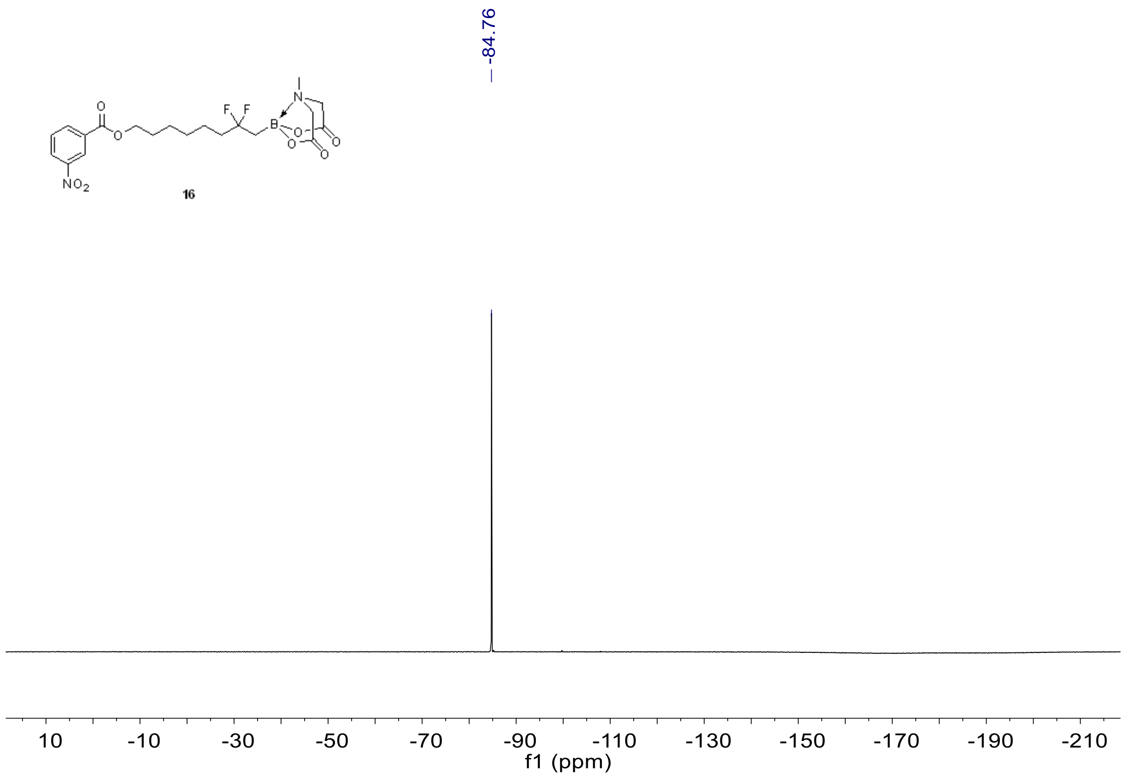

-84.76
NO2
16
10 -10 -30 -50 -70 -90 -110 -130 -150 -170 -190 -210
f1 (ppm)

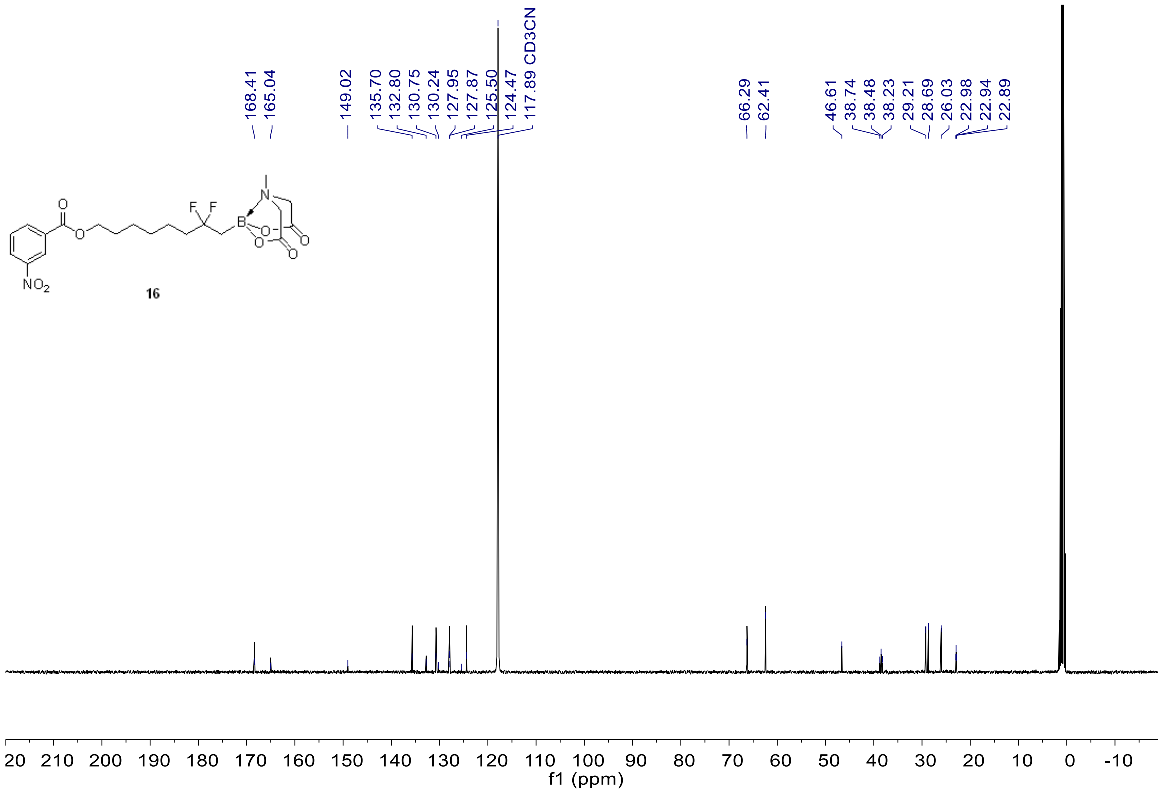

168.41
165.04
149.02
135.70
132.80
130.75
130.24
127.95
127.87
125.50
124.47
117.89 CD3CN
66.29
62.41
46.61
38.74
38.48
38.23
29.21
28.69
26.03
22.98
22.94
22.89
NO2
16
20 210 200 190 180 170 160 150 140 130 120 110 100 90 80 70 60 50 40 30 20 10 0 -10
f1 (ppm)

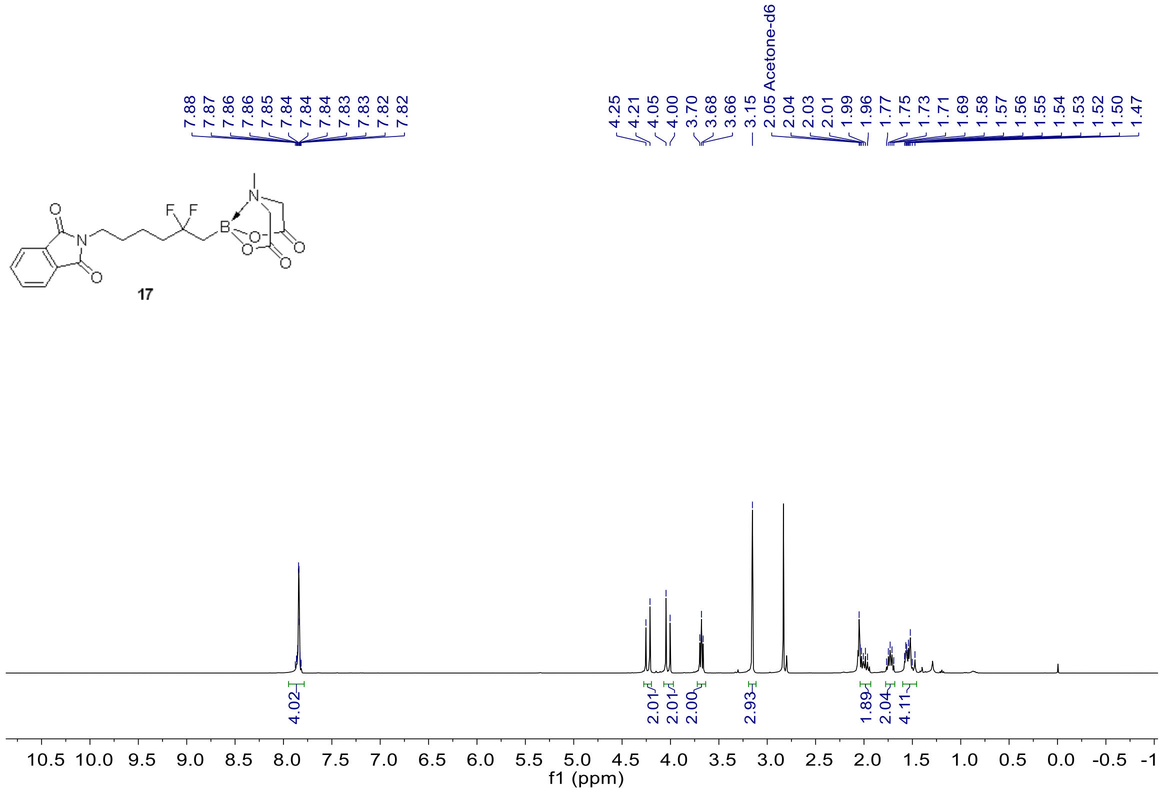
7.88 7.87 7.86 7.86 7.85 7.84 7.84 7.84 7.83 7.83 7.82 7.82
4.25 4.21 4.05 4.00 3.70 3.68 3.66 3.15 2.05 Acetone-d6 2.04 2.03 2.01 1.99 1.96 1.77 1.75 1.73 1.71 1.69 1.58 1.57 1.56 1.55 1.54 1.53 1.52 1.50 1.47
17
4.02
2.01
2.01
2.00
2.93
1.89
2.04
4.11
10.5 10.0 9.5 9.0 8.5 8.0 7.5 7.0 6.5 6.0 5.5 5.0 4.5 4.0 3.5 3.0 2.5 2.0 1.5 1.0 0.5 0.0 -0.5 -1
f1 (ppm)

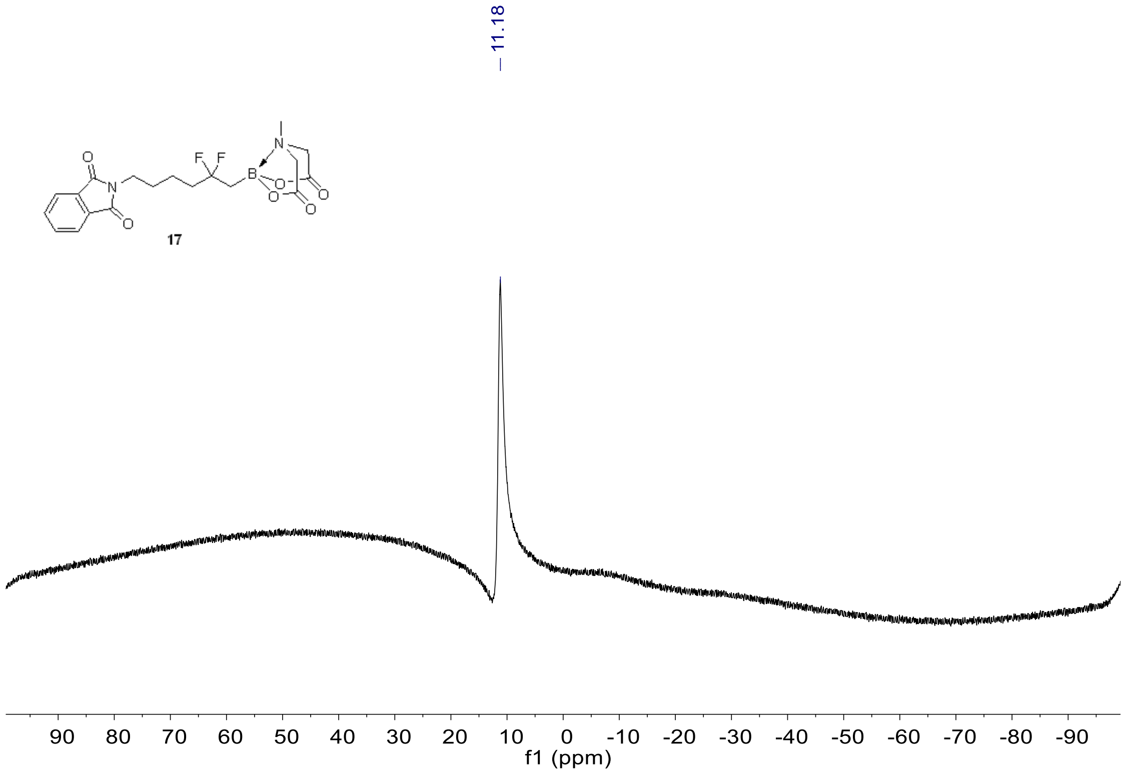
11.18
17
90 80 70 60 50 40 30 20 10 0 -10 -20 -30 -40 -50 -60 -70 -80 -90
f1 (ppm)

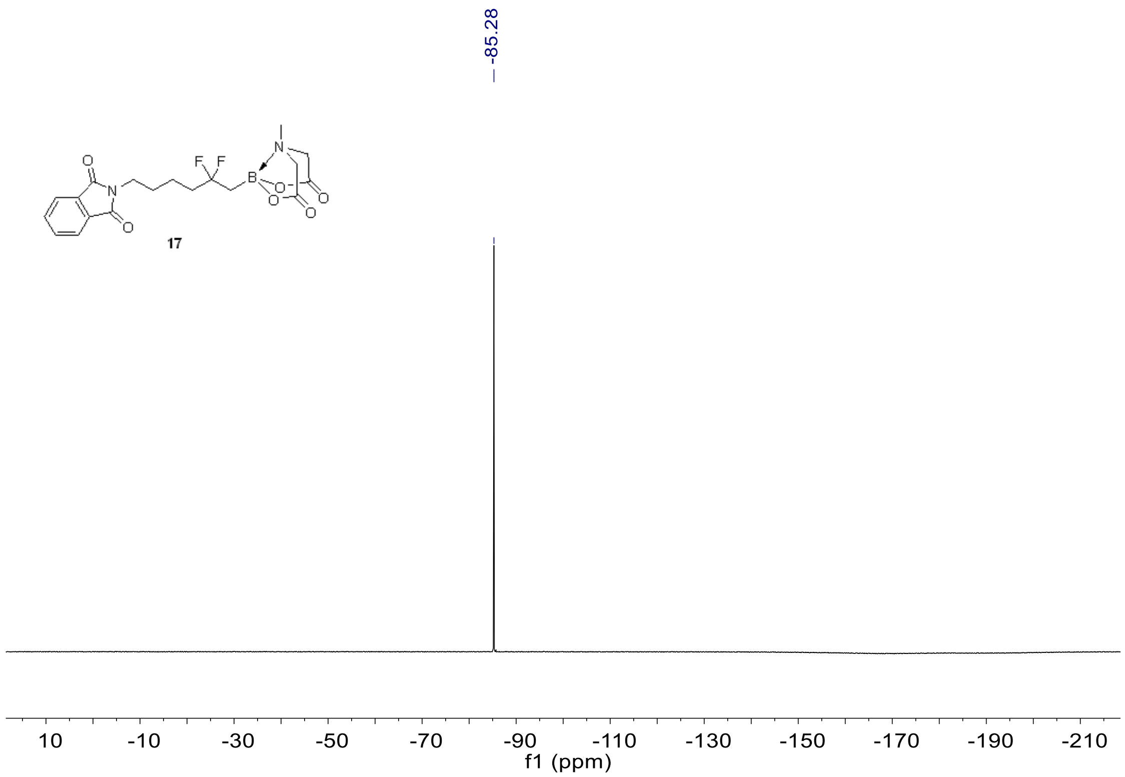
-85.28
17
10 -10 -30 -50 -70 -90 -110 -130 -150 -170 -190 -210
f1 (ppm)

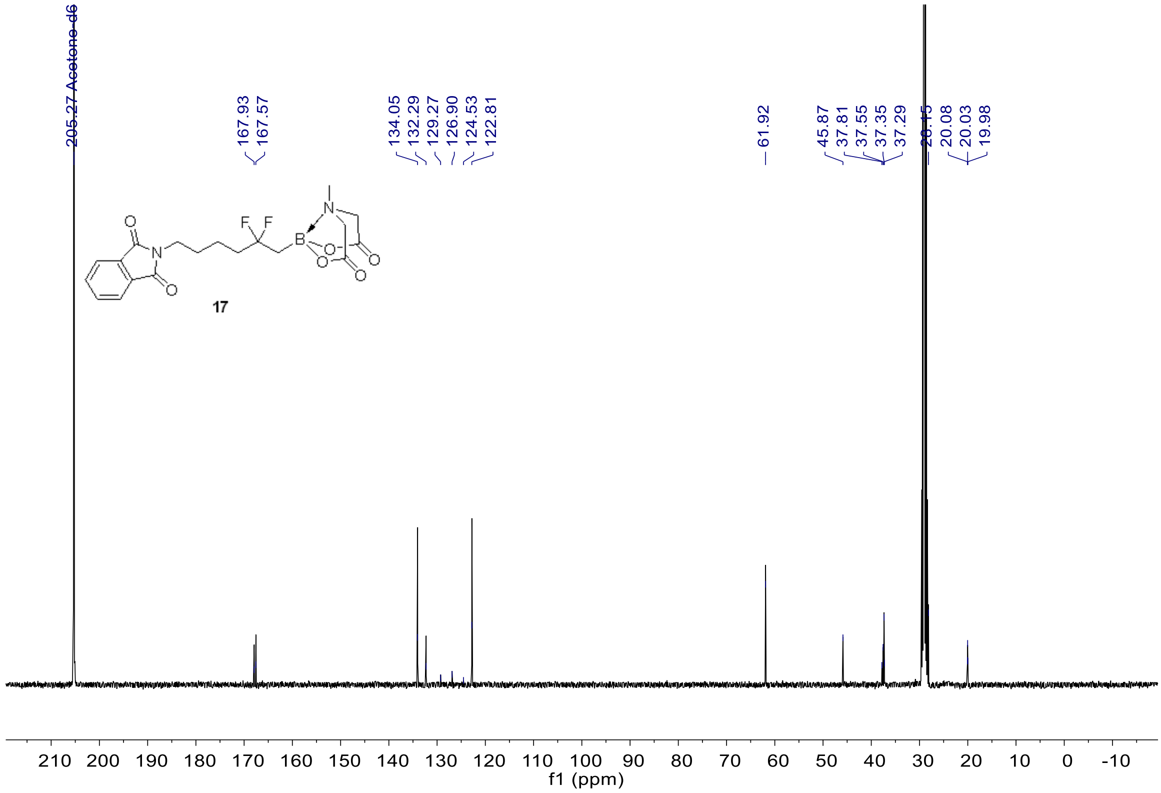
205.27 Acetone-d6
167.93
167.57
134.05
132.29
129.27
126.90
124.53
122.81
61.92
45.87
37.81
37.55
37.35
37.29
20.08
20.03
19.98
17
210 200 190 180 170 160 150 140 130 120 110 100 90 80 70 60 50 40 30 20 10 0 -10
f1 (ppm)

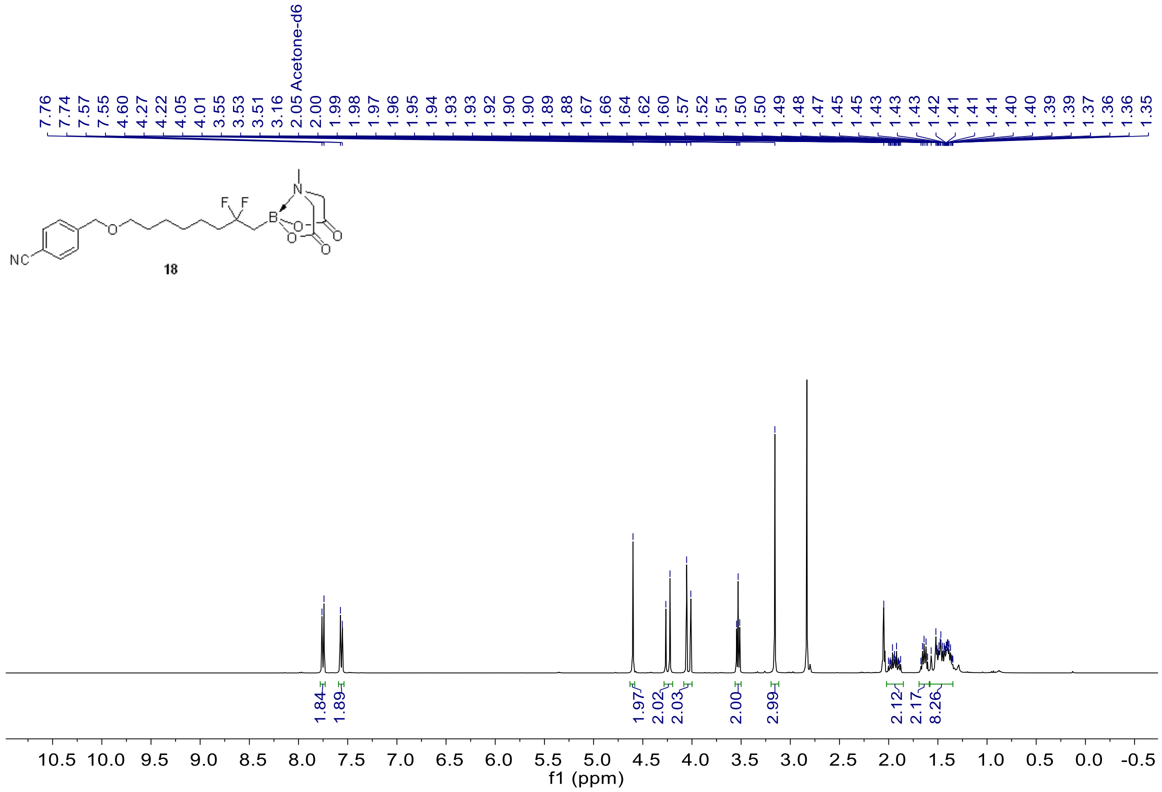

7.76 7.74 7.57 7.55 4.60 4.27 4.22 4.05 4.01 3.55 3.53 3.51 3.16 2.05 Acetone-d6 2.00 1.99 1.98 1.97 1.96 1.95 1.94 1.93 1.93 1.92 1.90 1.90 1.89 1.88 1.67 1.66 1.64 1.62 1.60 1.57 1.52 1.51 1.50 1.50 1.49 1.48 1.47 1.45 1.45 1.43 1.43 1.43 1.42 1.41 1.41 1.41 1.40 1.40 1.39 1.39 1.37 1.36 1.35
NC
F F
B
N
O
18
1.84 1.89 1.97 2.02 2.03 2.00 2.99 2.12 2.17 8.26
10.5 10.0 9.5 9.0 8.5 8.0 7.5 7.0 6.5 6.0 5.5 5.0 4.5 4.0 3.5 3.0 2.5 2.0 1.5 1.0 0.5 0.0 -0.5
f1 (ppm)

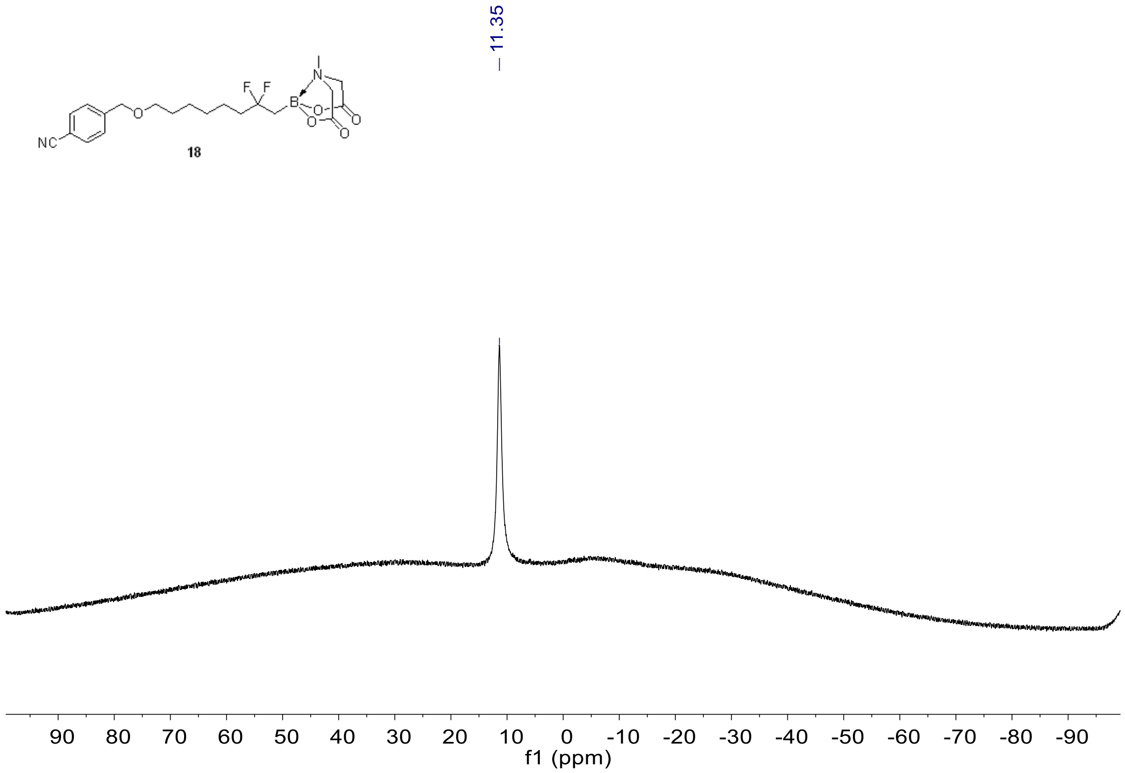

11.35
NC
F F
B
N
O
18
90 80 70 60 50 40 30 20 10 0 -10 -20 -30 -40 -50 -60 -70 -80 -90
f1 (ppm)

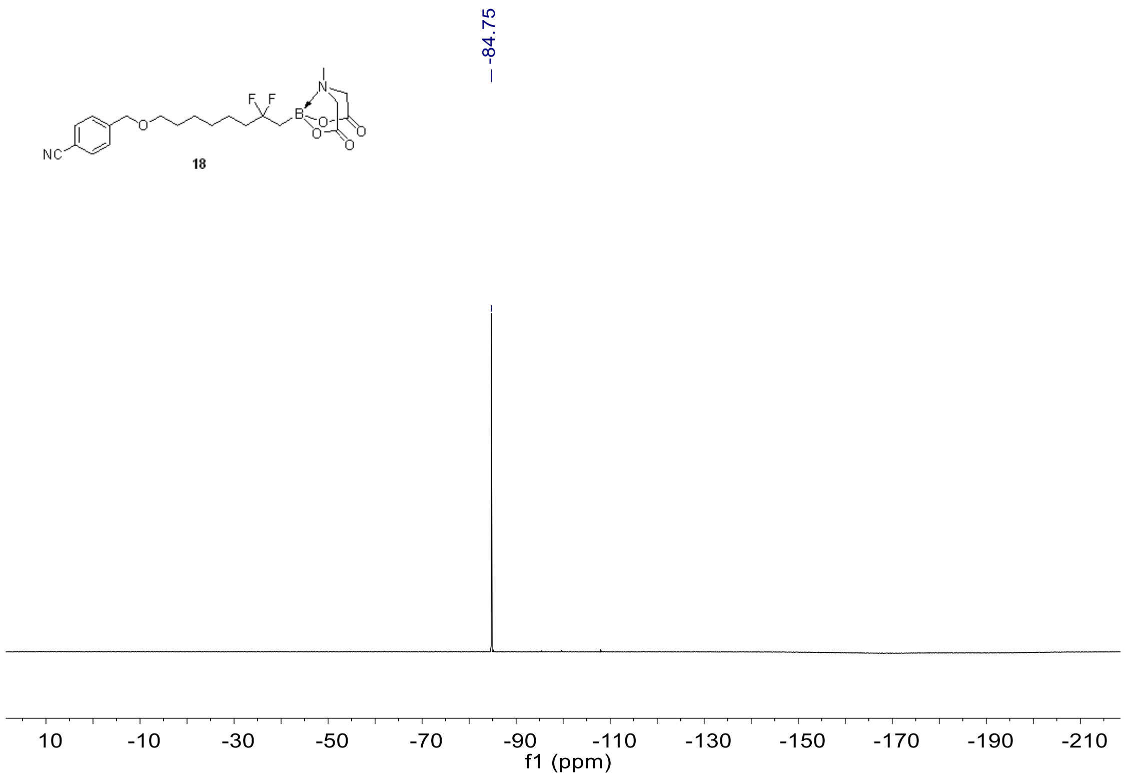

-84.75
NC
F F
N
B
O
O
O
O
18
10 -10 -30 -50 -70 -90 -110 -130 -150 -170 -190 -210
f1 (ppm)

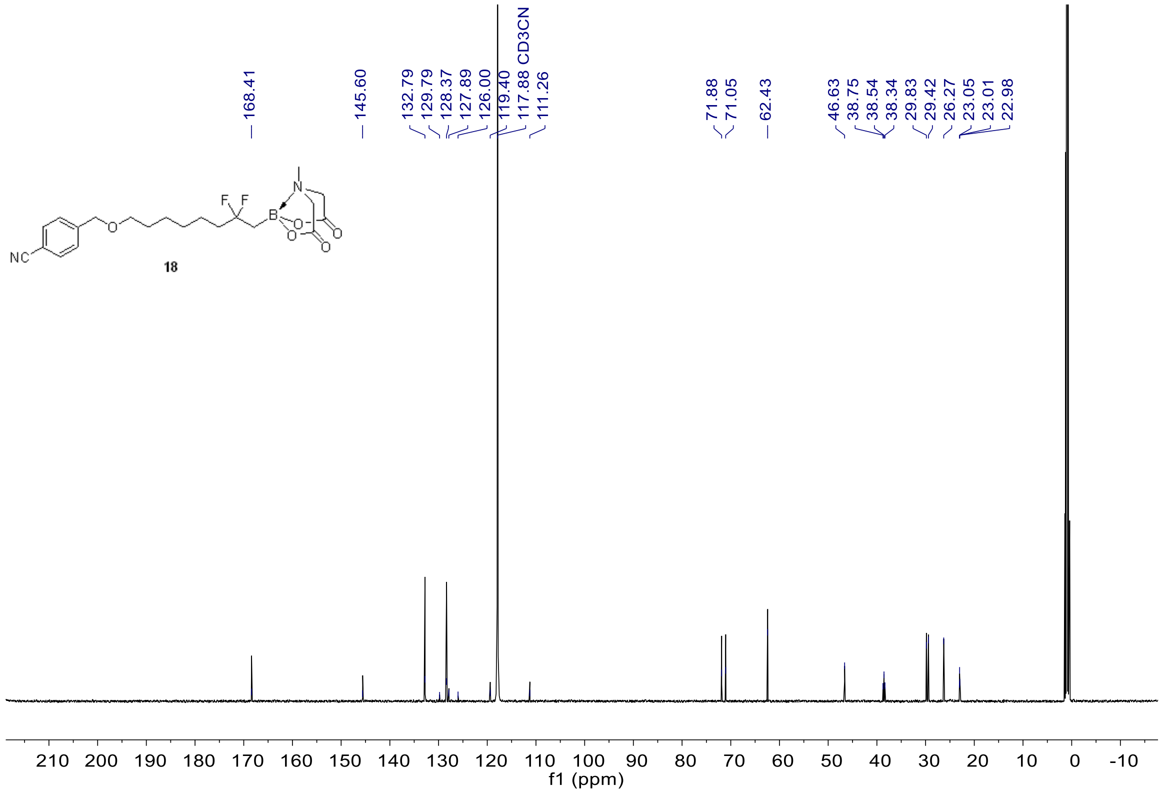

168.41
145.60
132.79
129.79
128.37
127.89
126.00
119.40
117.88 CD3CN
111.26
71.88
71.05
62.43
46.63
38.75
38.54
38.34
29.83
29.42
26.27
23.05
23.01
22.98
NC
F F
N
B
O
O
O
O
18
210 200 190 180 170 160 150 140 130 120 110 100 90 80 70 60 50 40 30 20 10 0 -10
f1 (ppm)

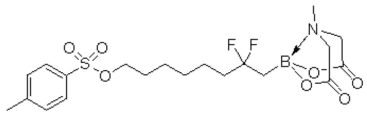

19

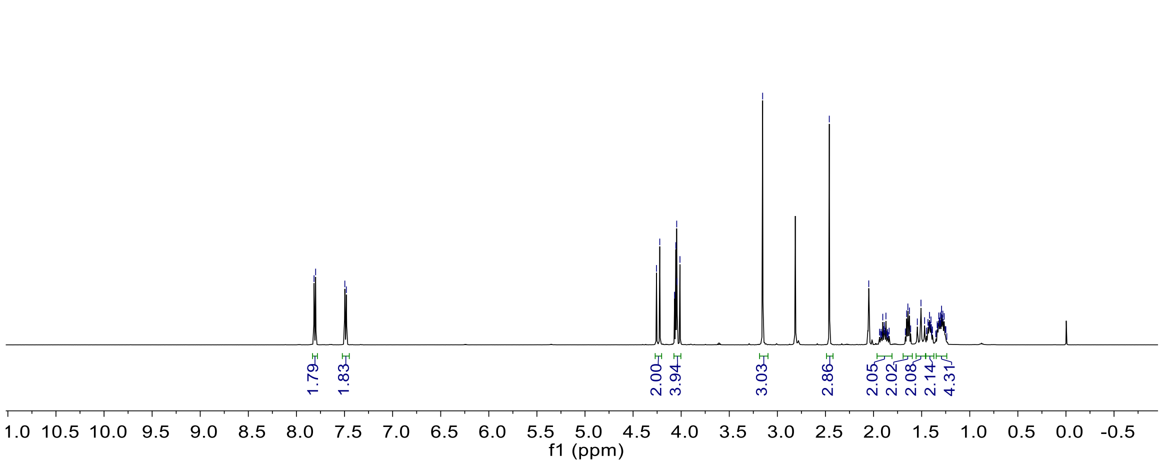

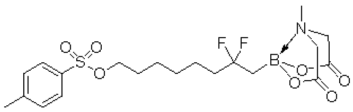

19

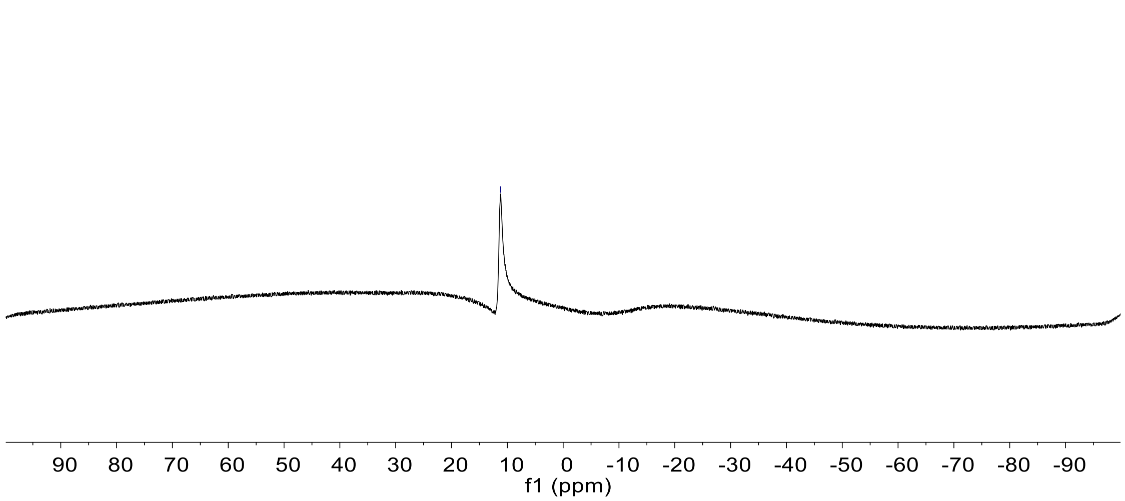

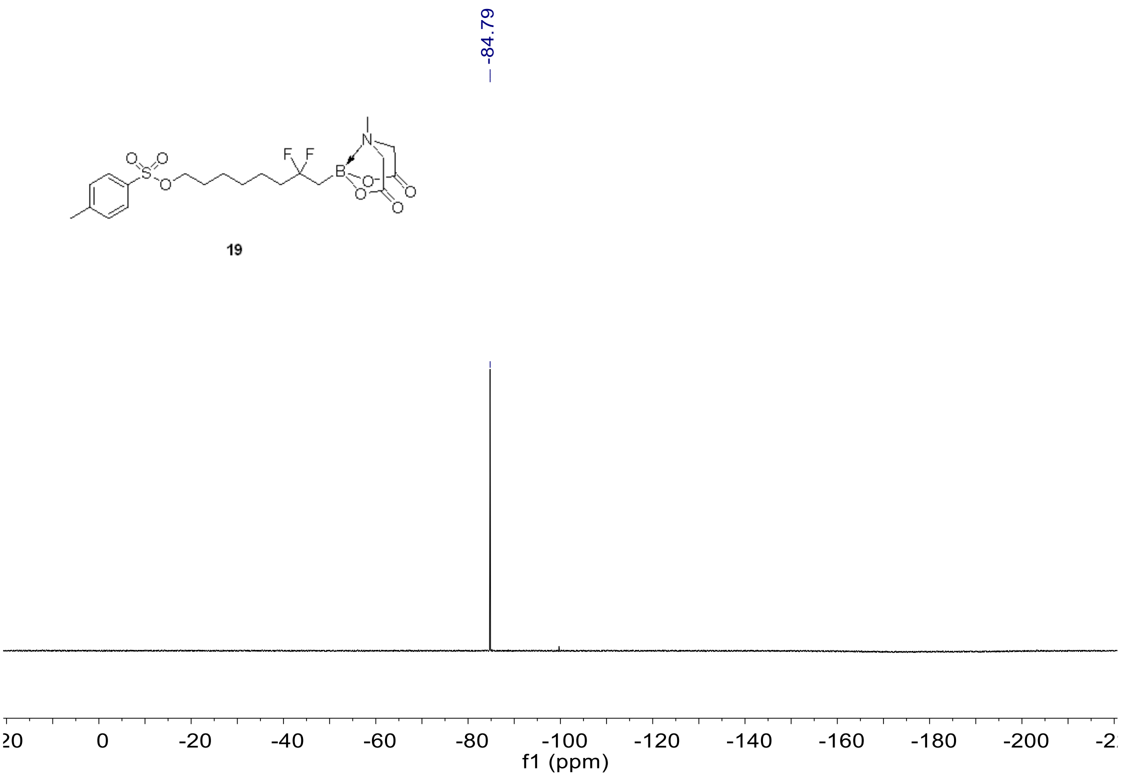

-84.79
20
0
-20
-40
-60
-80
-100
-120
-140
-160
-180
-200
-2
f1 (ppm)

19

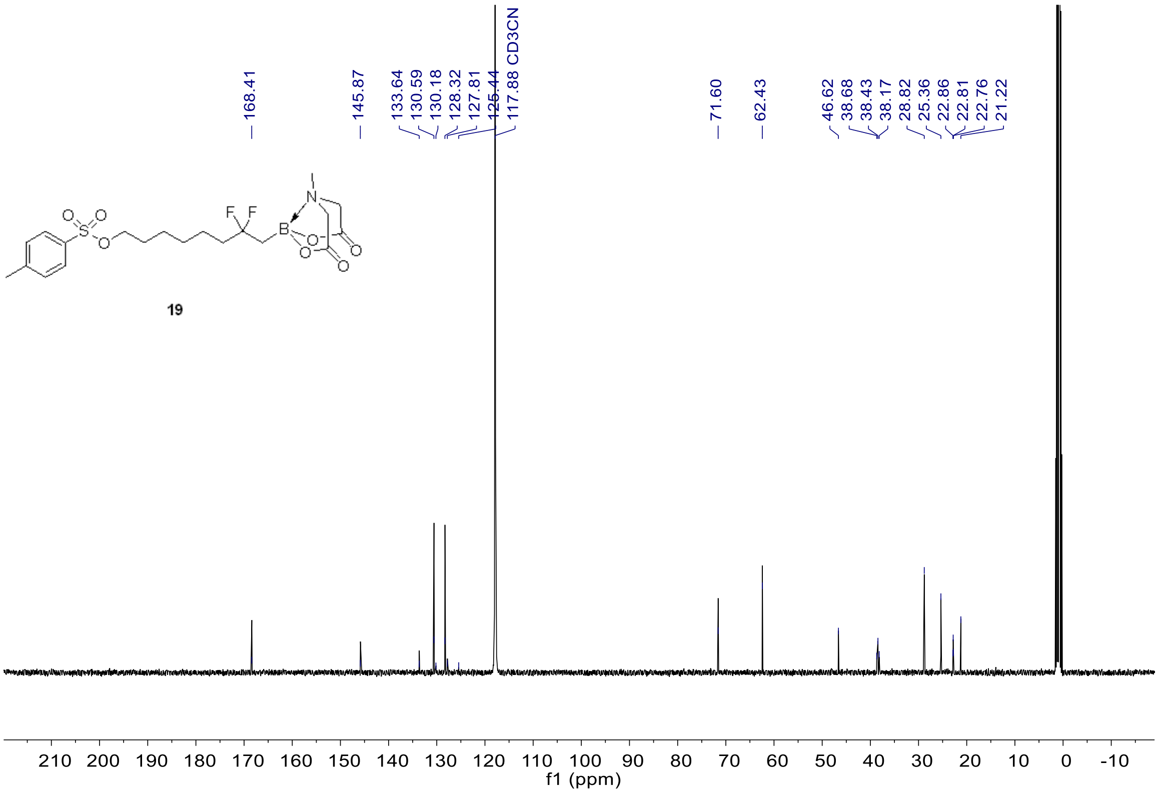

168.41
145.87
133.64
130.59
130.18
128.32
127.81
125.44
117.88 CD3CN
71.60
62.43
46.62
38.68
38.43
38.17
28.82
25.36
22.86
22.81
22.76
21.22
210 200 190 180 170 160 150 140 130 120 110 100 90 80 70 60 50 40 30 20 10 0 -10
f1 (ppm)

19

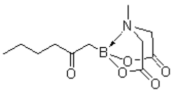

20

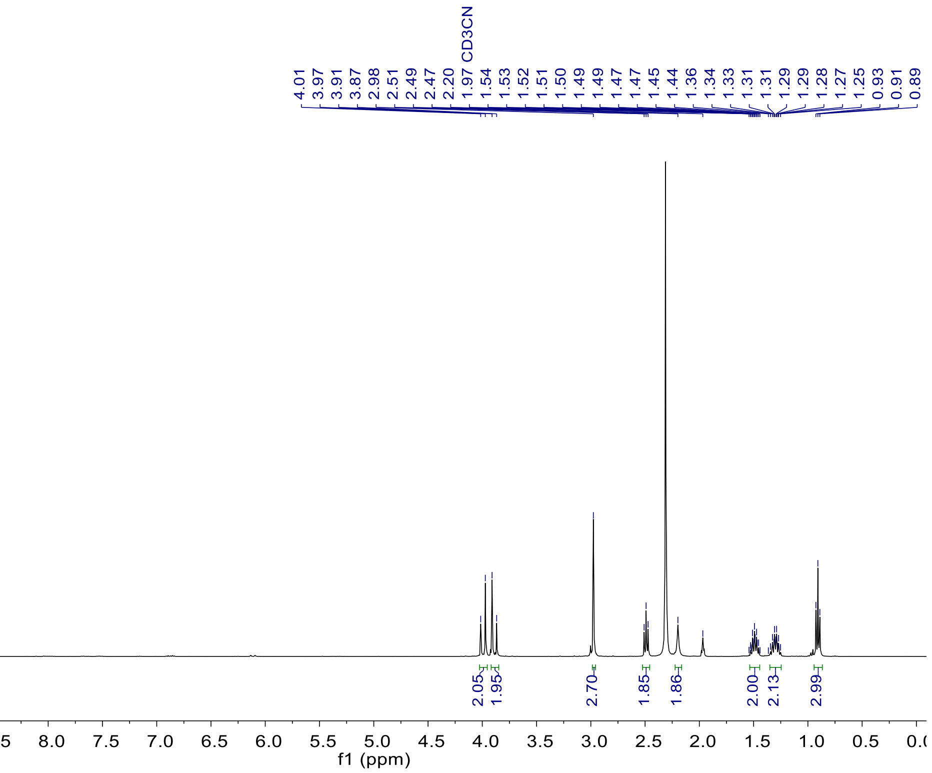
CD3CN
4.01 3.97 3.91 3.87 2.98 2.51 2.49 2.47 2.20 1.97 1.54 1.53 1.52 1.51 1.50 1.49 1.49 1.47 1.47 1.45 1.44 1.36 1.34 1.33 1.31 1.31 1.29 1.29 1.28 1.27 1.25 0.93 0.91 0.89
2.05 1.95 2.70 1.85 1.86 2.00 2.13 2.99
10.0 9.5 9.0 8.5 8.0 7.5 7.0 6.5 6.0 5.5 5.0 4.5 4.0 3.5 3.0 2.5 2.0 1.5 1.0 0.5 0.(
f1 (ppm)

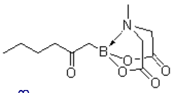

20

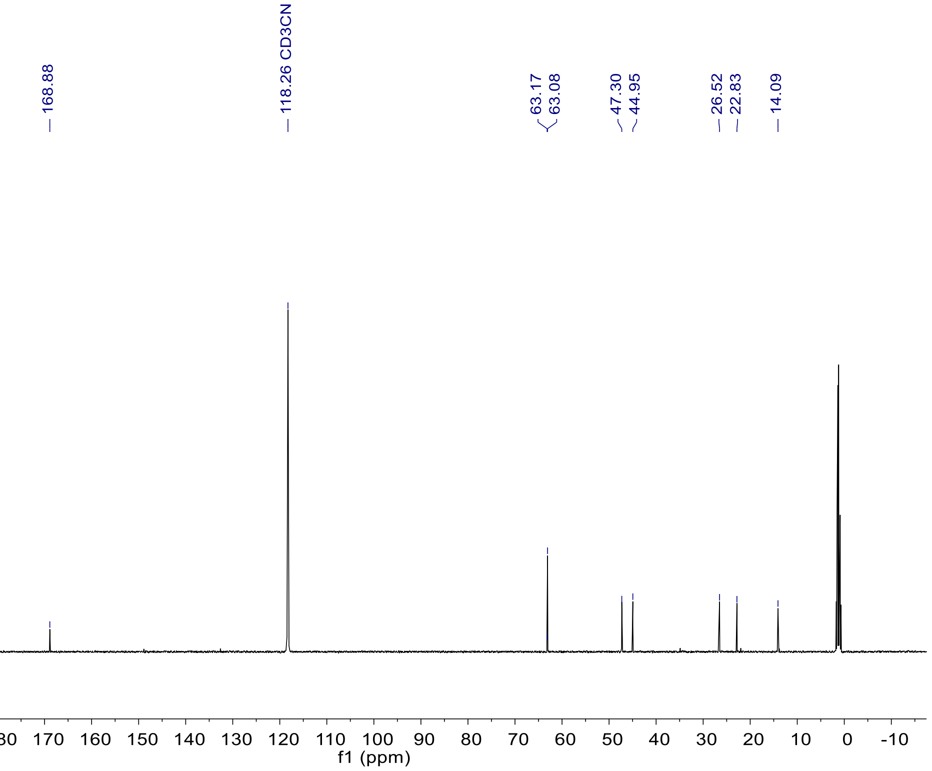
213
168.88
118.26 CD3CN
63.17 63.08
47.30 44.95
26.52 22.83
14.09
210 200 190 180 170 160 150 140 130 120 110 100 90 80 70 60 50 40 30 20 10 0 -10
f1 (ppm)

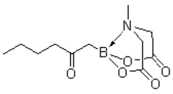

20

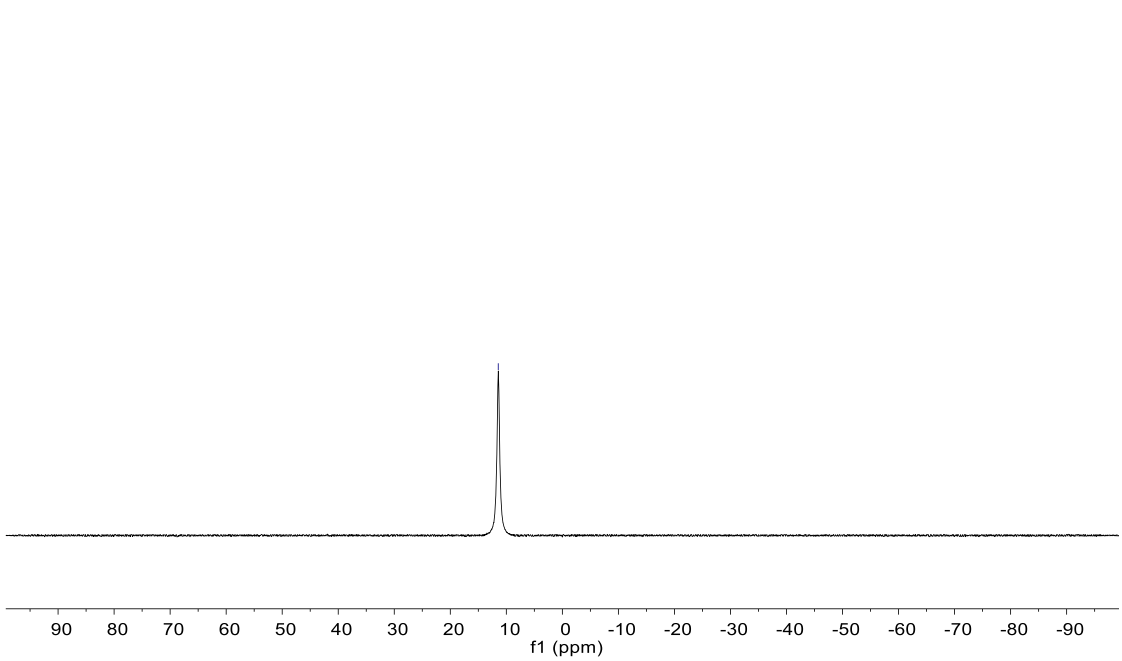

11.44
90 80 70 60 50 40 30 20 10 0 -10 -20 -30 -40 -50 -60 -70 -80 -90
f1 (ppm)

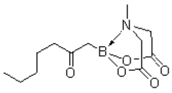

21

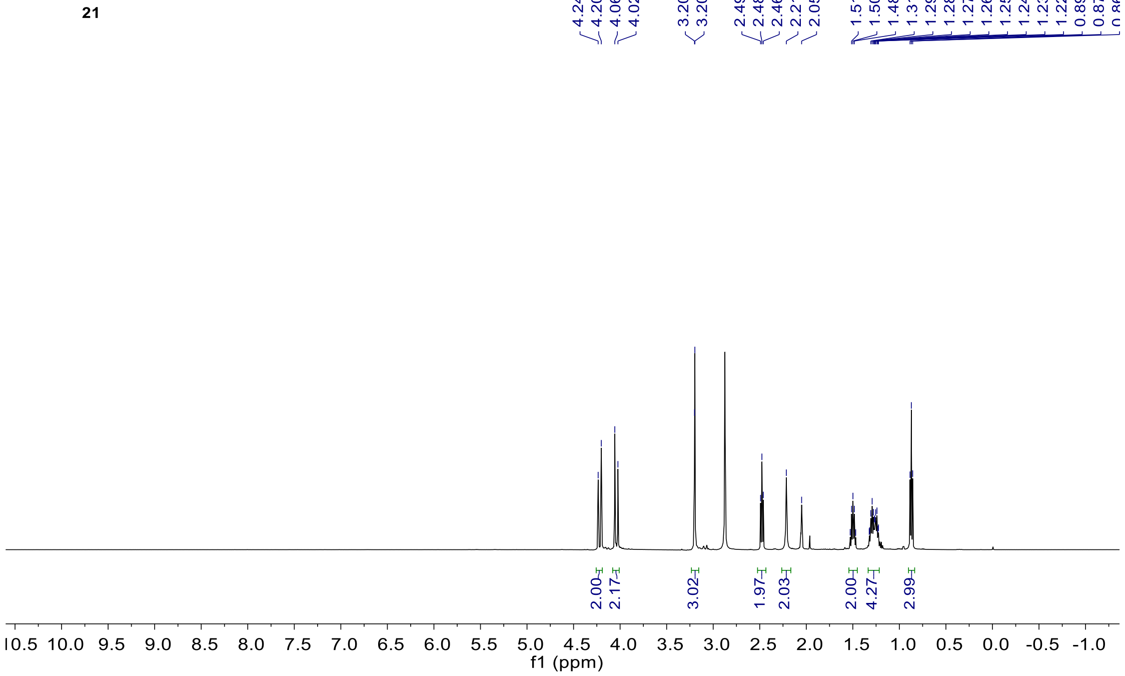

4.24
4.20
4.06
4.02
3.20
3.20
2.49
2.48
2.46
2.21
2.05 Acetone-d6
1.51
1.50
1.48
1.31
1.29
1.28
1.27
1.26
1.25
1.24
1.23
1.22
0.89
0.87
2.00
2.17
3.02
1.97
2.03
2.00
4.27
2.99
10.5 10.0 9.5 9.0 8.5 8.0 7.5 7.0 6.5 6.0 5.5 5.0 4.5 4.0 3.5 3.0 2.5 2.0 1.5 1.0 0.5 0.0 -0.5 -1.0
f1 (ppm)

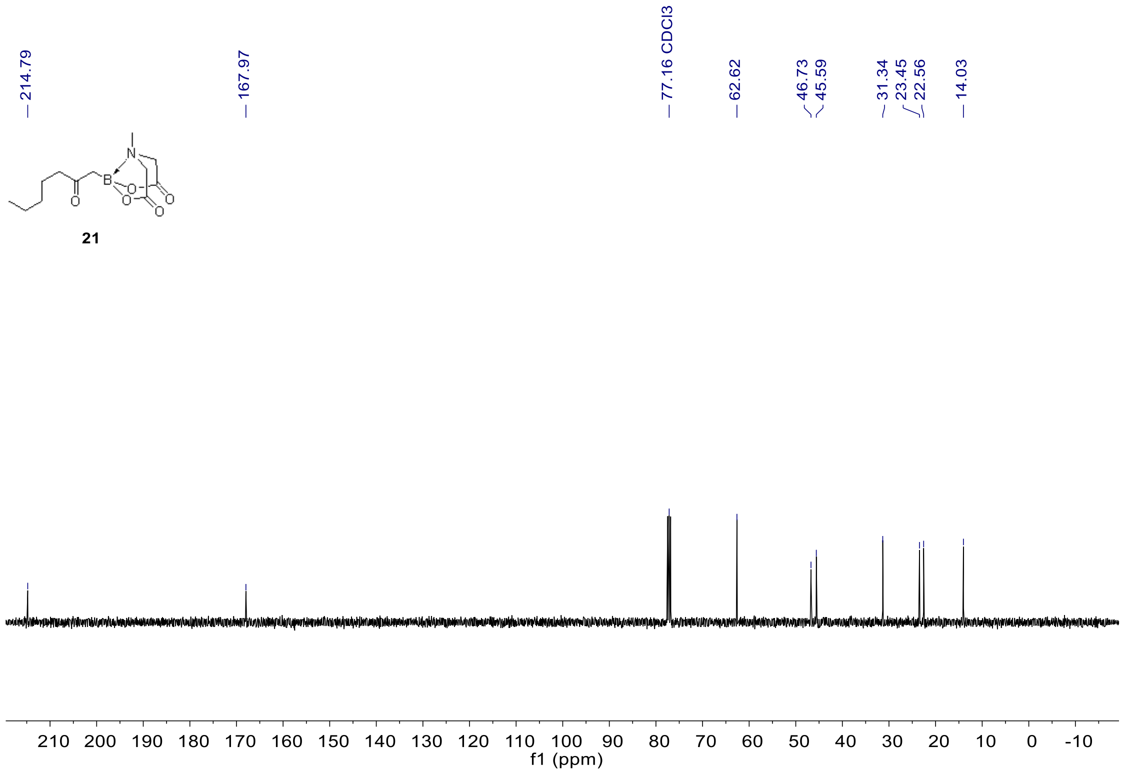
214.79
167.97
77.16 CDCl3
62.62
46.73
45.59
31.34
23.45
22.56
14.03
210 200 190 180 170 160 150 140 130 120 110 100 90 80 70 60 50 40 30 20 10 0 -10
f1 (ppm)

21

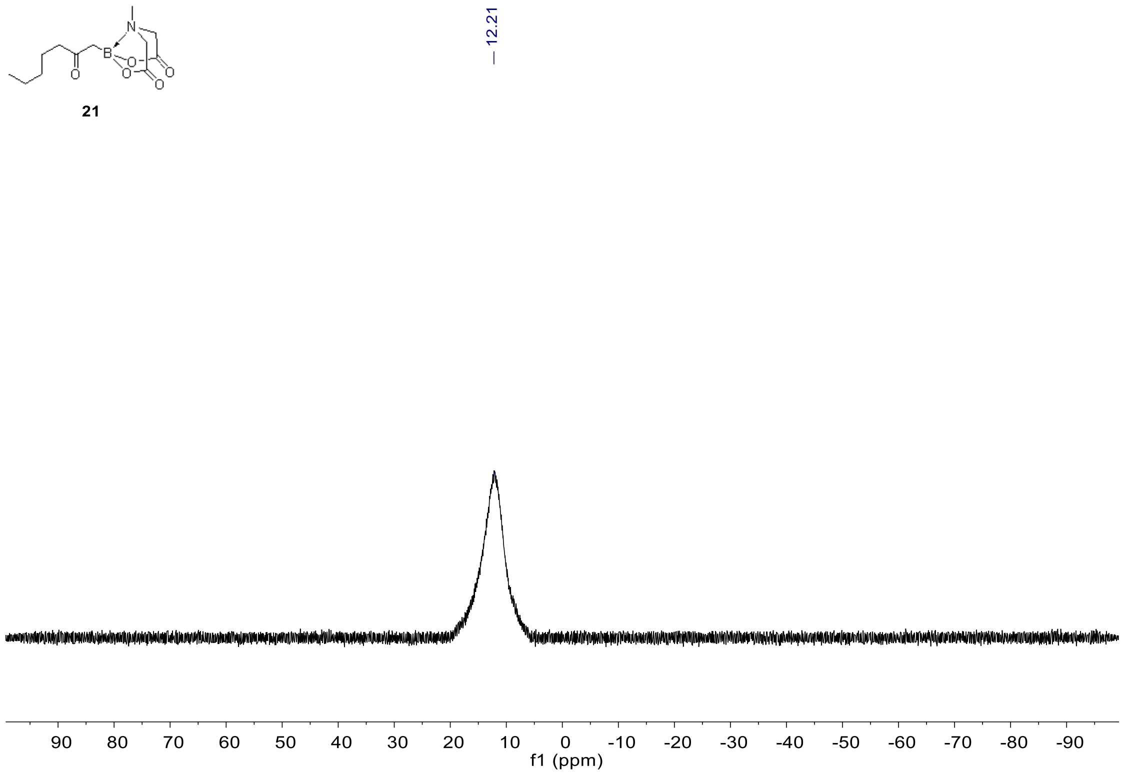
12.21
90 80 70 60 50 40 30 20 10 0 -10 -20 -30 -40 -50 -60 -70 -80 -90
f1 (ppm)

21

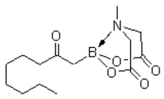

22

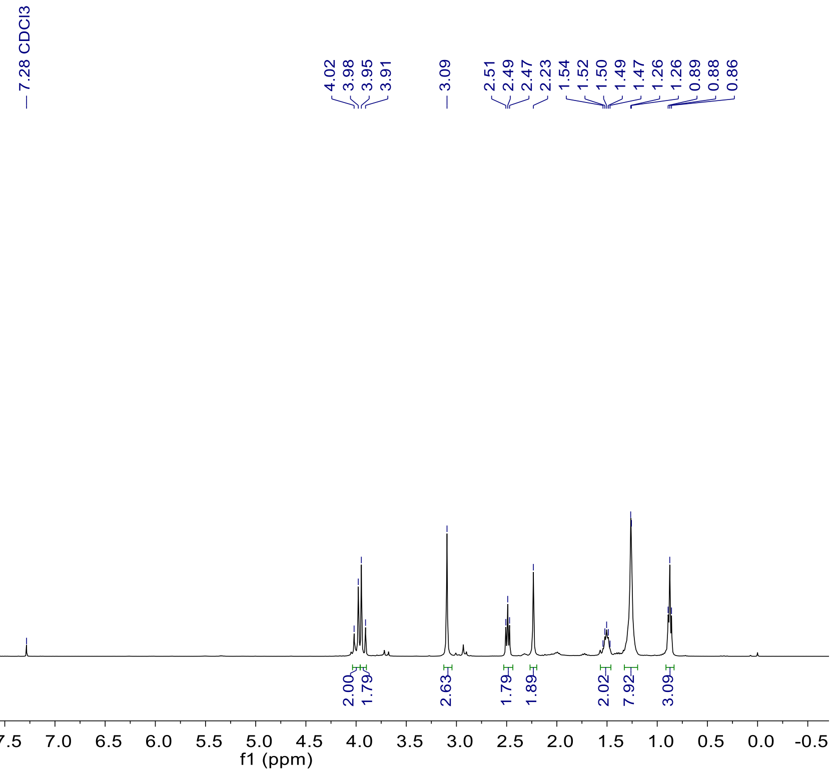

7.28 CDCl3
4.02 3.98 3.95 3.91
3.09
2.51 2.49 2.47 2.23 1.54 1.52 1.50 1.49 1.47 1.26 1.26 0.89 0.88 0.86
2.00 1.79 2.63 1.79 1.89 2.02 7.92 3.09
10.0 9.5 9.0 8.5 8.0 7.5 7.0 6.5 6.0 5.5 5.0 4.5 4.0 3.5 3.0 2.5 2.0 1.5 1.0 0.5 0.0 -0.5
f1 (ppm)

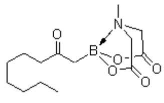

22

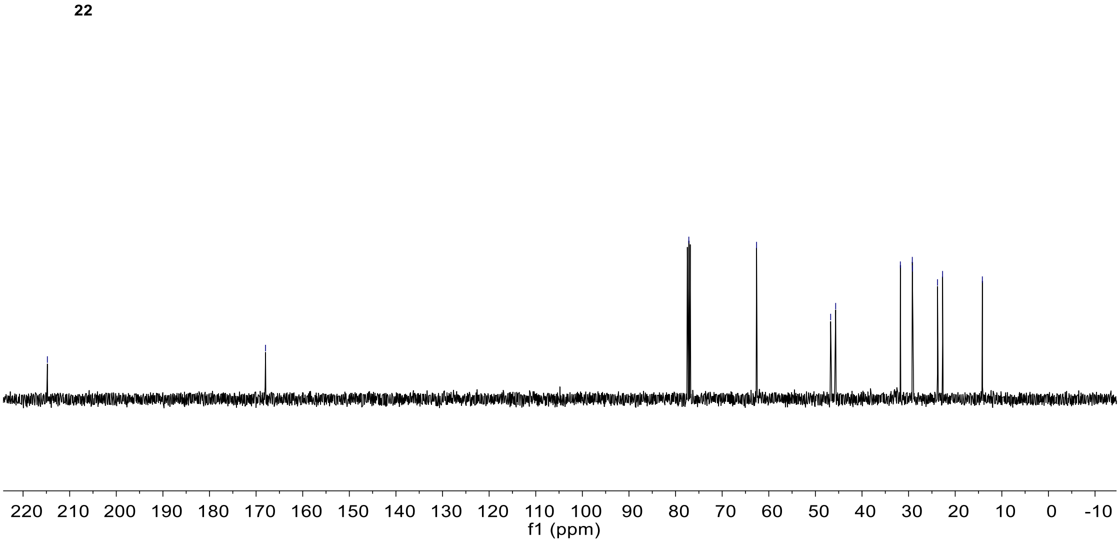

214.79
167.98
77.16 CDCl3
62.62
46.73 45.65
31.77 29.21 29.17 23.80 22.70
14.18
220 210 200 190 180 170 160 150 140 130 120 110 100 90 80 70 60 50 40 30 20 10 0 -10
f1 (ppm)

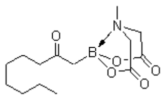

22

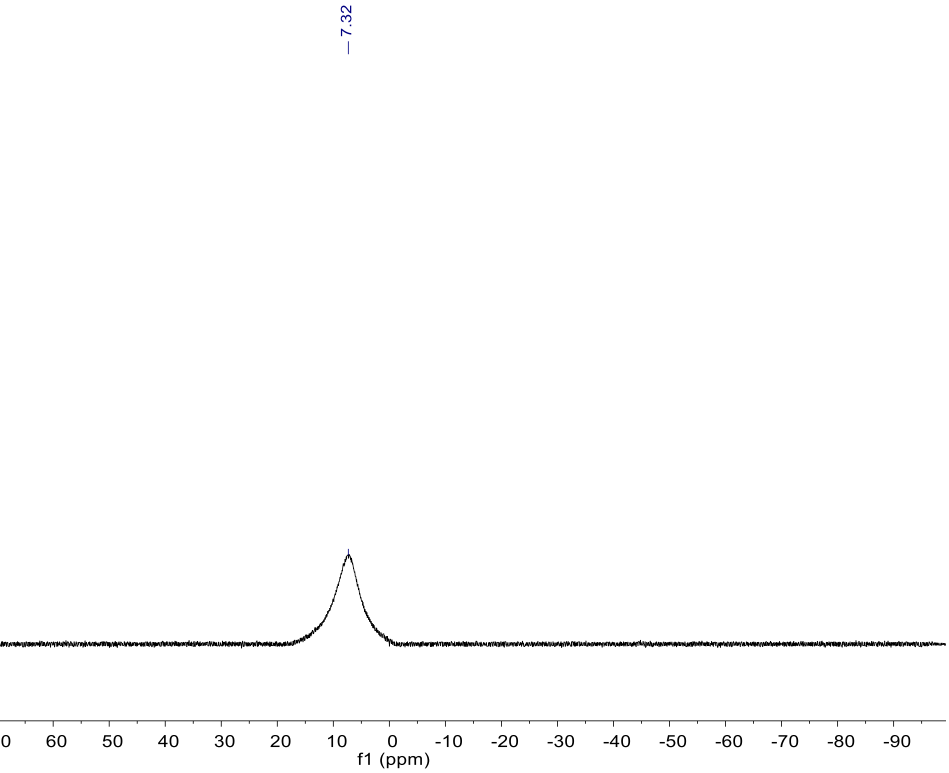

7.32
90 80 70 60 50 40 30 20 10 0 -10 -20 -30 -40 -50 -60 -70 -80 -90
f1 (ppm)

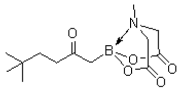

23

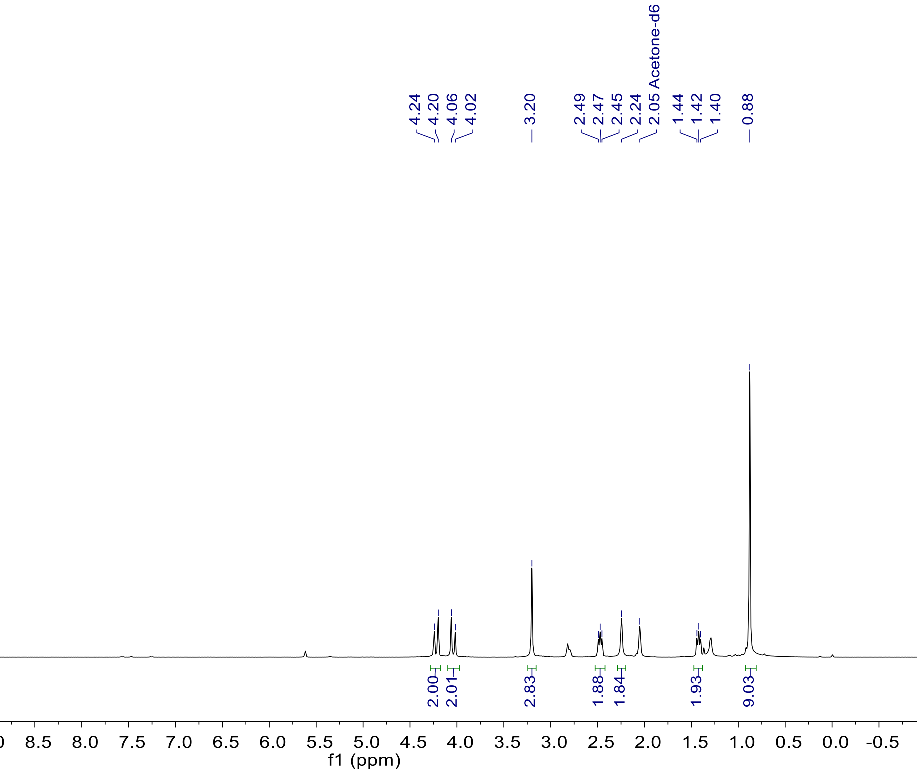

4.24
4.20
4.06
4.02
3.20
2.49
2.47
2.45
2.24
2.05 Acetone-d6
1.44
1.42
1.40
0.88
2.00
2.01
2.83
1.88
1.84
1.93
9.03
10.5 10.0 9.5 9.0 8.5 8.0 7.5 7.0 6.5 6.0 5.5 5.0 4.5 4.0 3.5 3.0 2.5 2.0 1.5 1.0 0.5 0.0 -0.5
f1 (ppm)

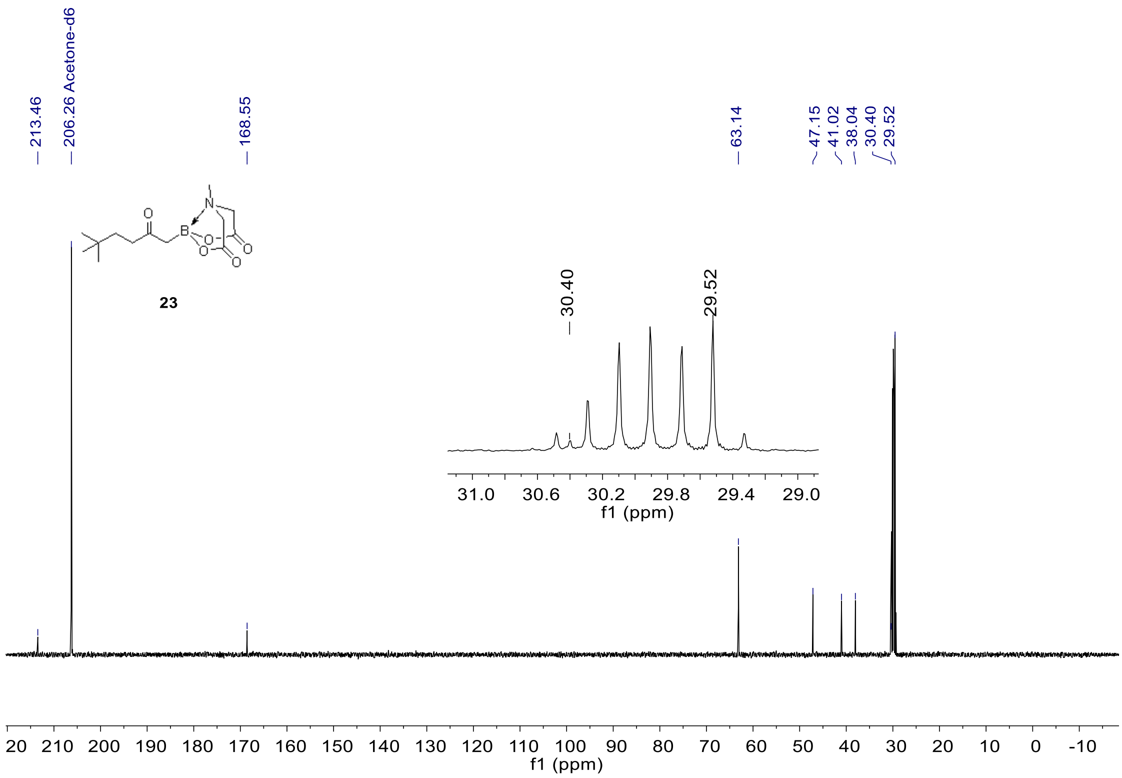

213.46
206.26 Acetone-d6
168.55
63.14
47.15
41.02
38.04
30.40
29.52
23
30.40
29.52
31.0 30.6 30.2 29.8 29.4 29.0
f1 (ppm)
20 210 200 190 180 170 160 150 140 130 120 110 100 90 80 70 60 50 40 30 20 10 0 -10
f1 (ppm)

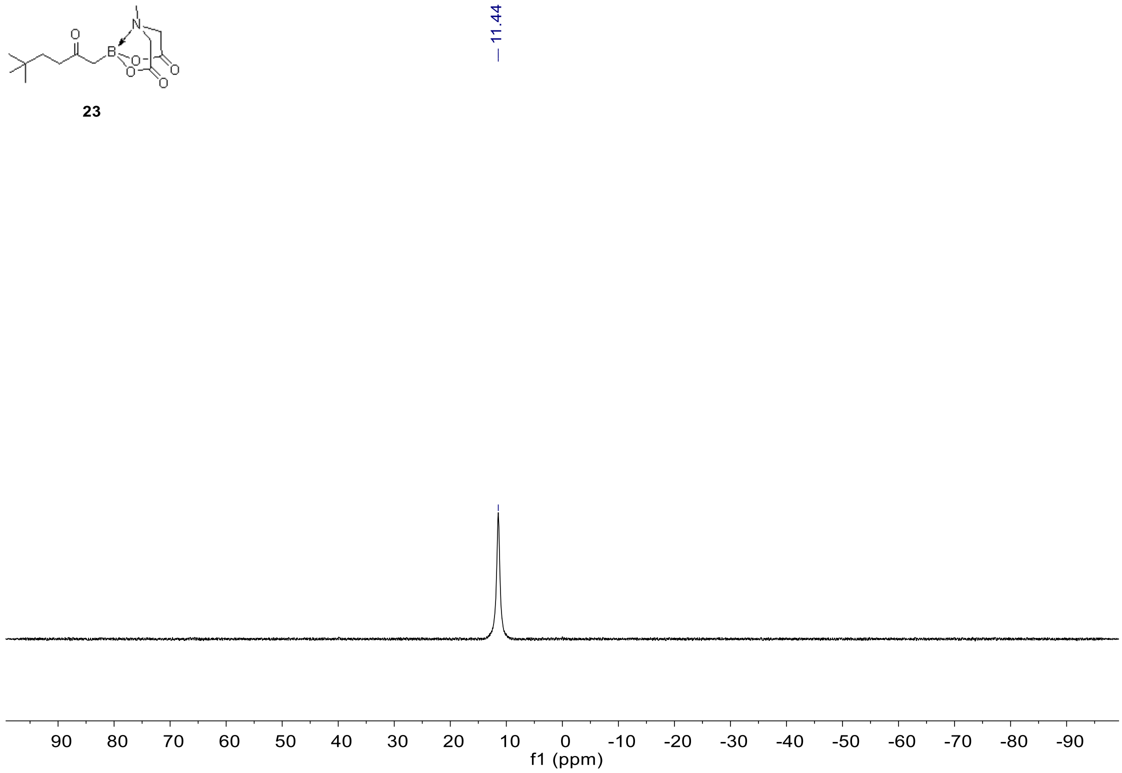

11.44
23
90 80 70 60 50 40 30 20 10 0 -10 -20 -30 -40 -50 -60 -70 -80 -90
f1 (ppm)

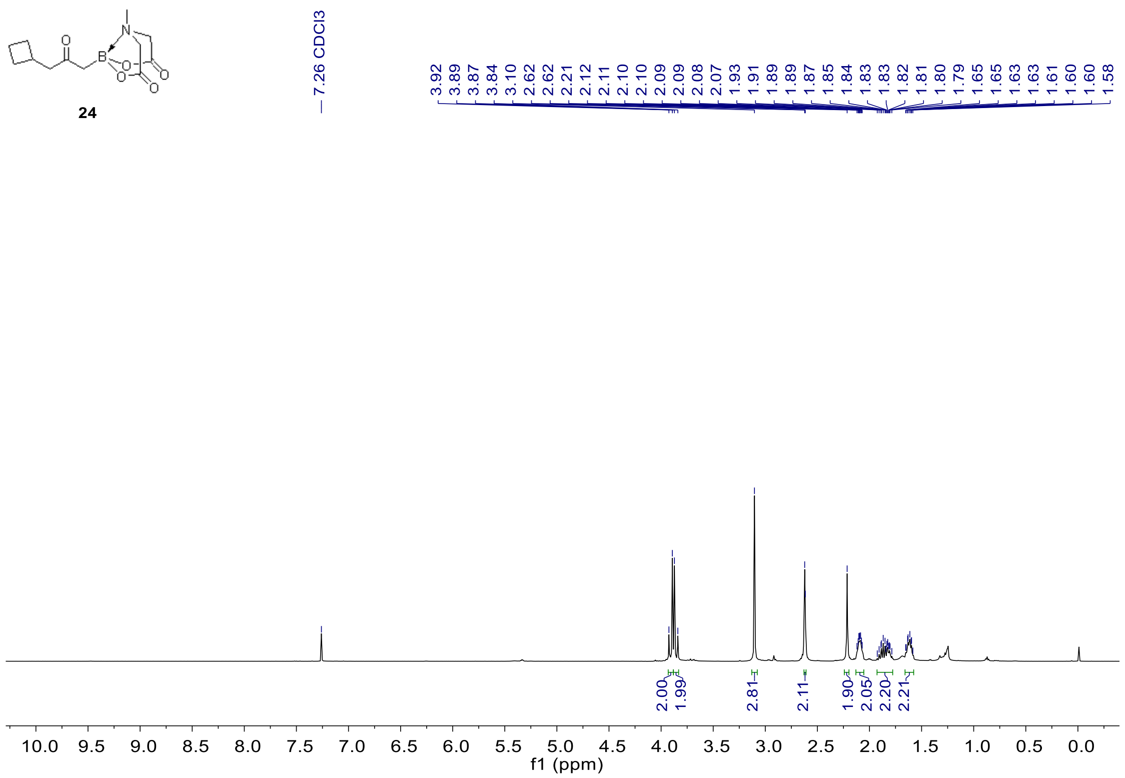
24
7.26 CDCl3
3.92 3.89 3.87 3.84 3.10 2.62 2.62 2.21 2.12 2.11 2.10 2.10 2.09 2.09 2.08 2.07 1.93 1.91 1.89 1.89 1.87 1.85 1.84 1.83 1.83 1.82 1.81 1.80 1.79 1.65 1.65 1.63 1.63 1.61 1.60 1.60 1.58
2.00 1.99 2.81 2.11 1.90 2.05 2.20 2.21
10.0 9.5 9.0 8.5 8.0 7.5 7.0 6.5 6.0 5.5 5.0 4.5 4.0 3.5 3.0 2.5 2.0 1.5 1.0 0.5 0.0
f1 (ppm)

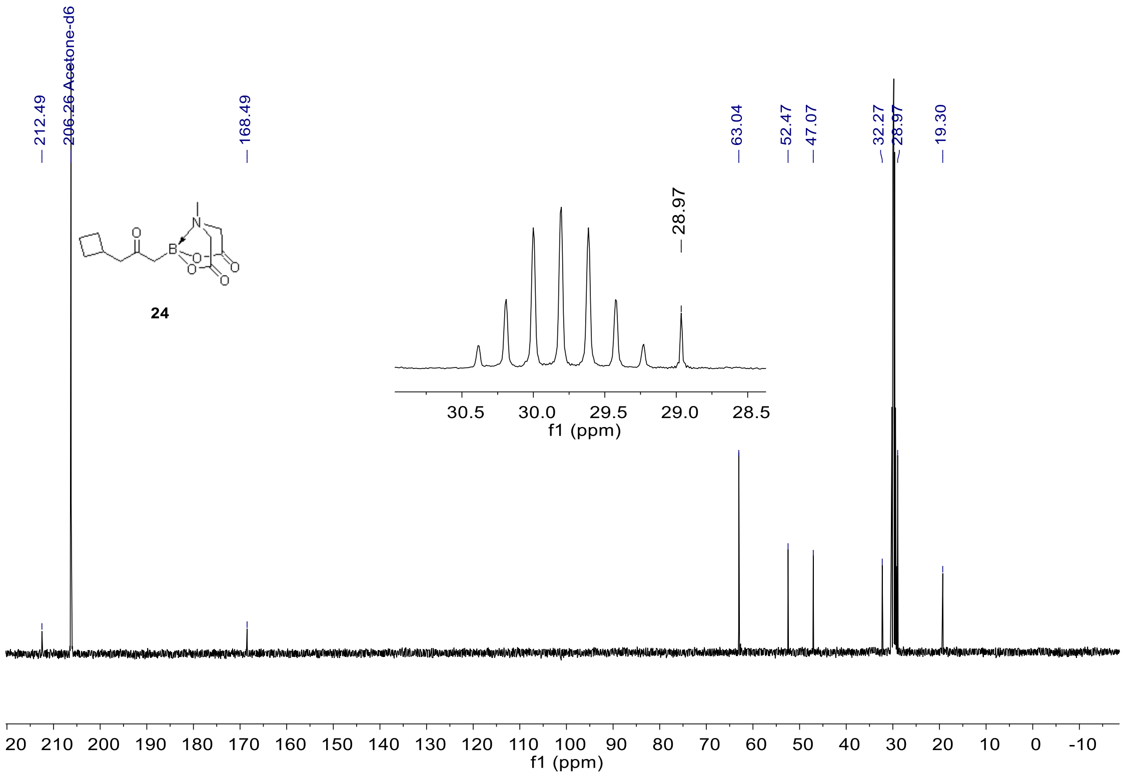
212.49
206.26 Acetone-d6
168.49
63.04
52.47
47.07
32.27
28.97
19.30
24
28.97
30.5 30.0 29.5 29.0 28.5
f1 (ppm)
20 210 200 190 180 170 160 150 140 130 120 110 100 90 80 70 60 50 40 30 20 10 0 -10
f1 (ppm)

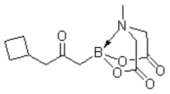

24

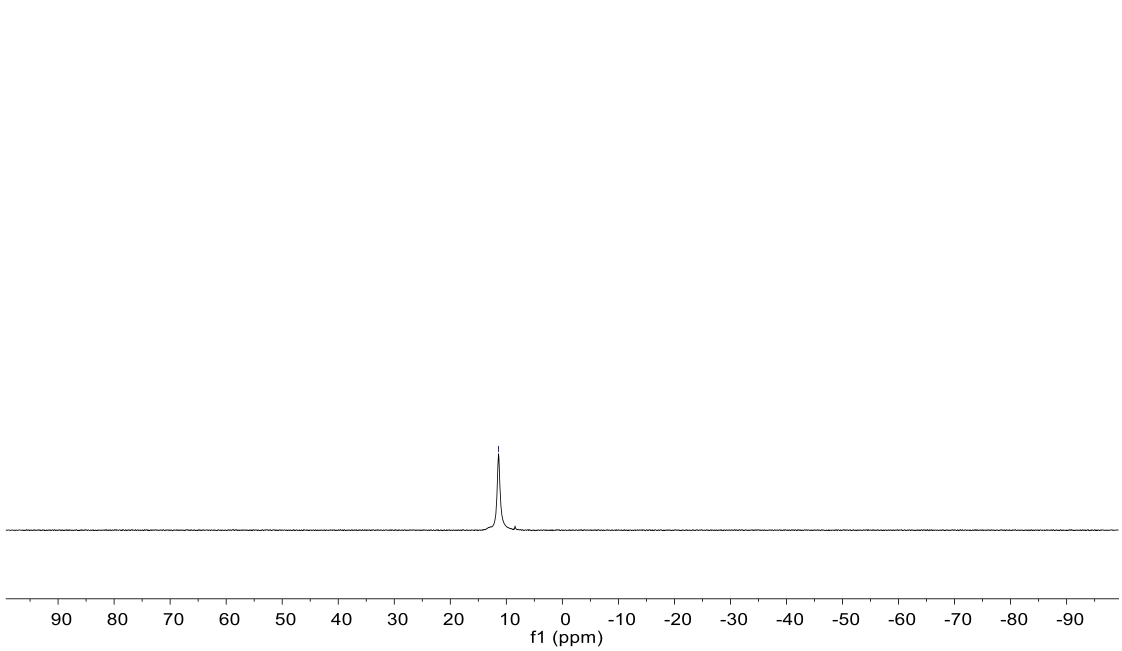

11.39
90 80 70 60 50 40 30 20 10 0 -10 -20 -30 -40 -50 -60 -70 -80 -90
f1 (ppm)

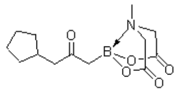

25

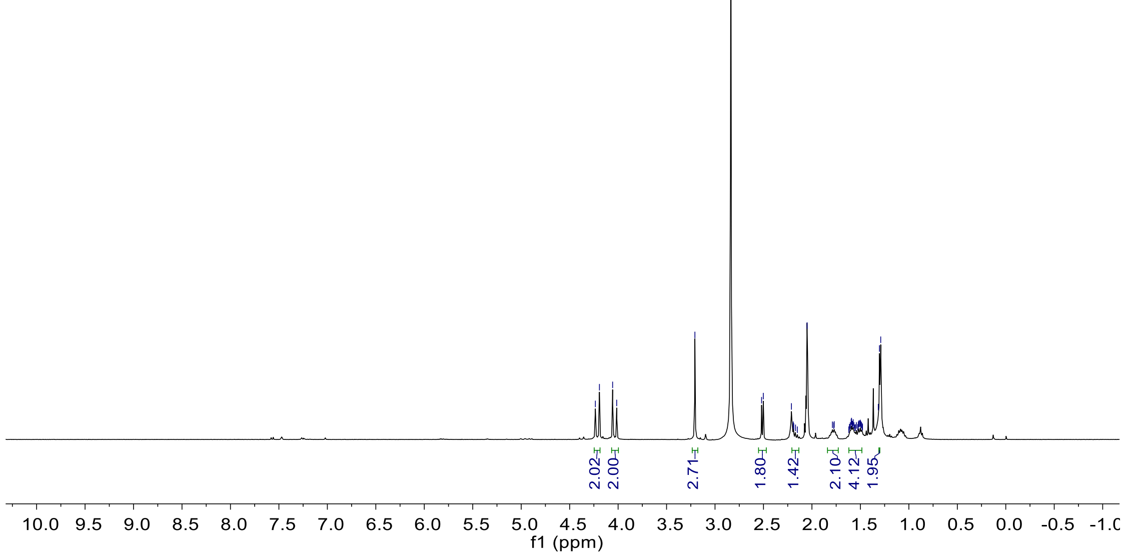

4.23 4.19 4.06 4.01 3.21 2.52 2.50 2.21 2.19 2.19 2.17 2.15 2.05 Acetone-d6 1.79 1.77 1.77 1.62 1.61 1.60 1.60 1.59 1.59 1.58 1.57 1.57 1.56 1.55 1.54 1.53 1.52 1.52 1.51 1.50 1.50 1.49 1.48 1.48 1.32 1.30 1.29
2.02 2.00 2.71 1.80 1.42 2.10 4.12 1.95
10.0 9.5 9.0 8.5 8.0 7.5 7.0 6.5 6.0 5.5 5.0 4.5 4.0 3.5 3.0 2.5 2.0 1.5 1.0 0.5 0.0 -0.5 -1.0
f1 (ppm)

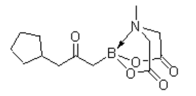

25

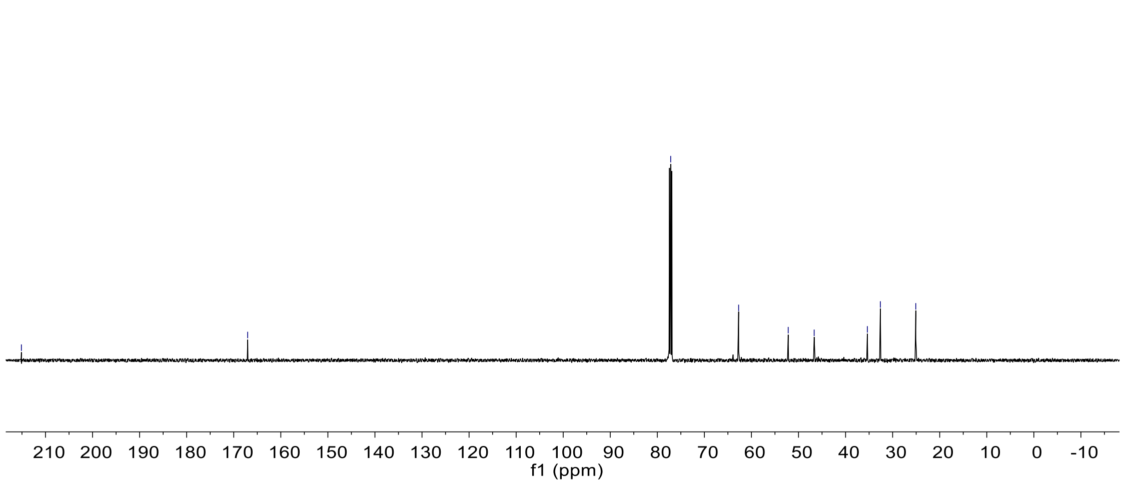

215.12
167.04
77.16 CDCl3
62.72
52.19
46.67
35.37
32.61
25.08
210 200 190 180 170 160 150 140 130 120 110 100 90 80 70 60 50 40 30 20 10 0 -10
f1 (ppm)

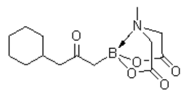

26

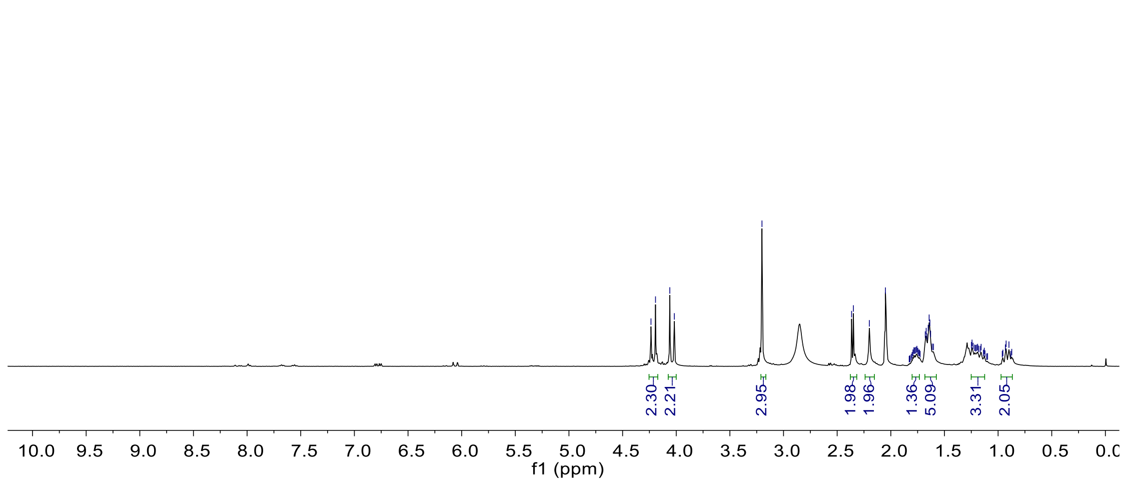

4.24
4.19
4.06
4.02
3.20
2.37
2.35
2.20
2.05 Acetone-d6
1.83
1.82
1.81
1.80
1.79
1.78
1.77
1.76
1.75
1.74
1.73
1.73
1.68
1.67
1.66
1.65
1.64
1.64
1.63
1.61
1.60
1.25
1.24
1.23
1.22
1.21
1.20
1.19
1.18
1.17
1.16
1.14
1.13
1.12
1.11
1.10
0.96
0.96
0.93
0.93
0.90
0.88
0.87
2.30
2.21
2.95
1.98
1.96
1.36
5.09
3.31
2.05
10.0 9.5 9.0 8.5 8.0 7.5 7.0 6.5 6.0 5.5 5.0 4.5 4.0 3.5 3.0 2.5 2.0 1.5 1.0 0.5 0.0
f1 (ppm)

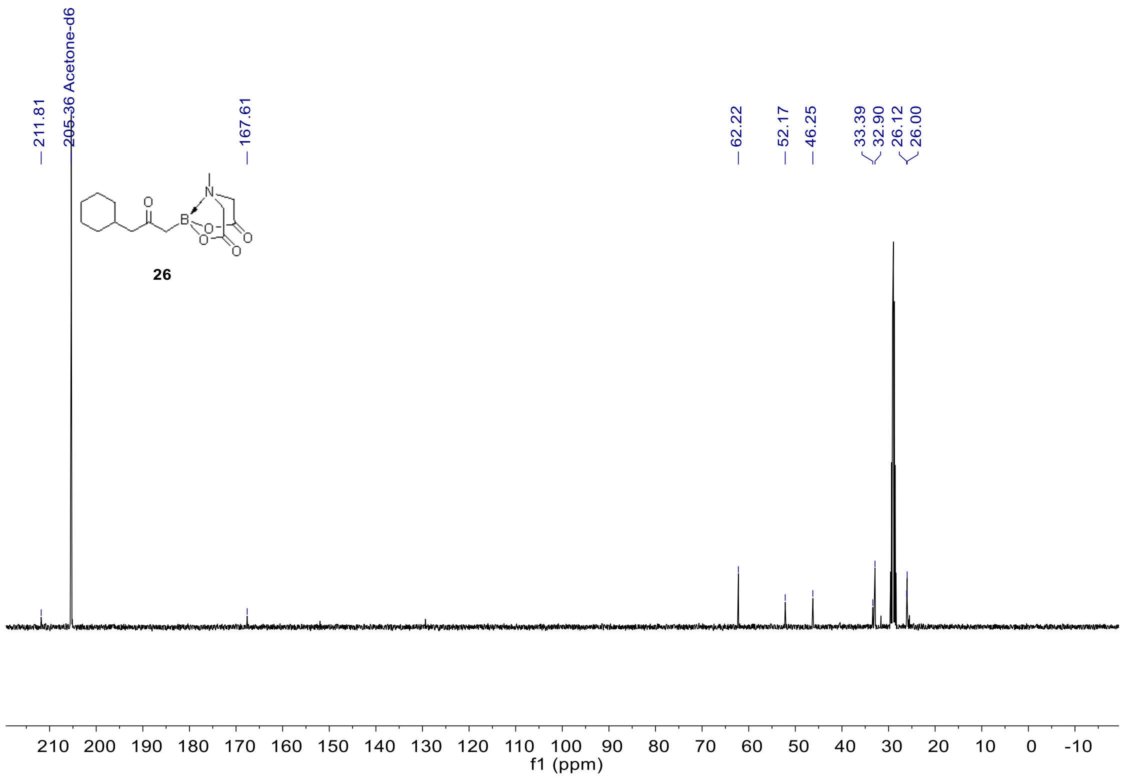
211.81
205.36 Acetone-d6
167.61
62.22
52.17
46.25
33.39
32.90
26.12
26.00
26
210 200 190 180 170 160 150 140 130 120 110 100 90 80 70 60 50 40 30 20 10 0 -10
f1 (ppm)

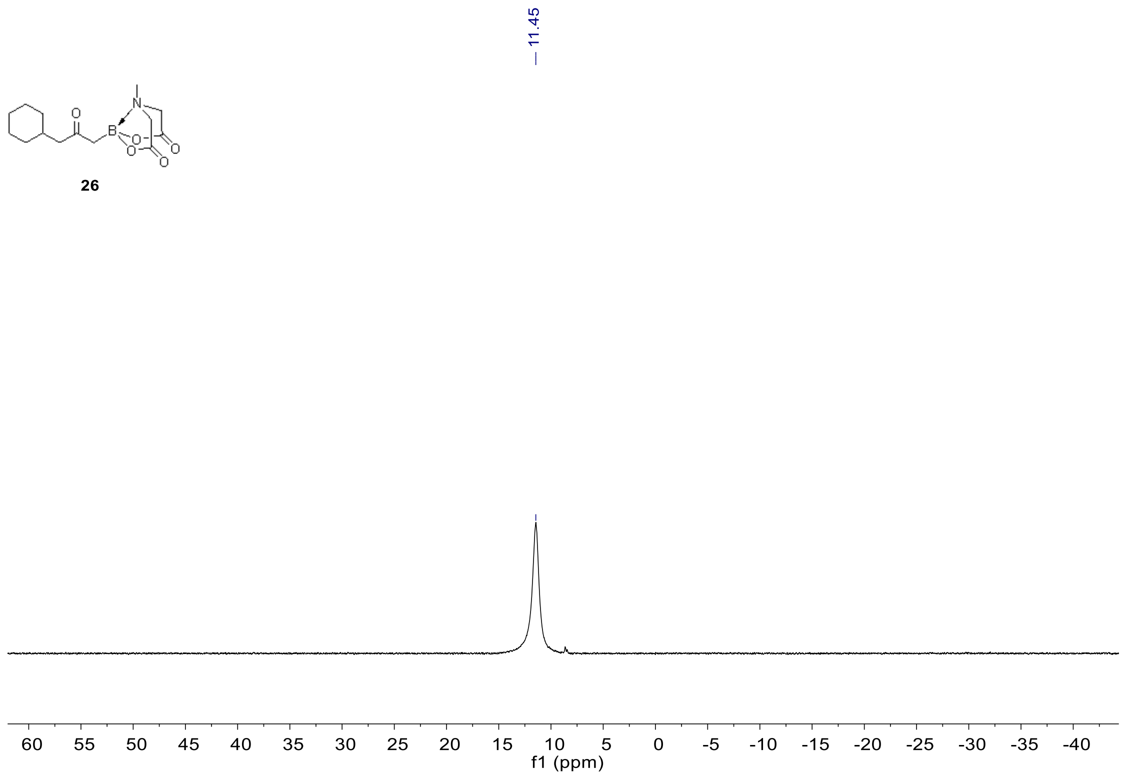
11.45
26
60 55 50 45 40 35 30 25 20 15 10 5 0 -5 -10 -15 -20 -25 -30 -35 -40
f1 (ppm)

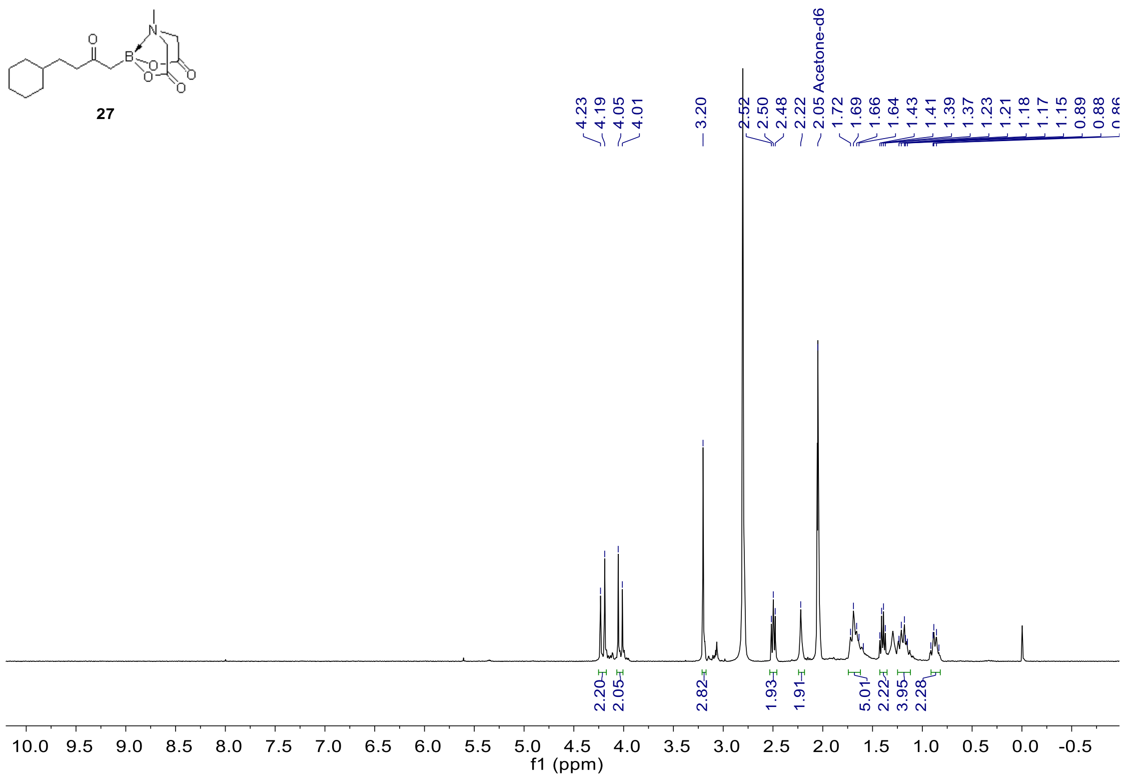
27
4.23
4.19
4.05
4.01
3.20
2.52
2.50
2.48
2.22
2.05 Acetone-d6
1.72
1.69
1.66
1.64
1.43
1.41
1.39
1.37
1.23
1.21
1.18
1.17
1.15
0.89
0.88
2.20
2.05
2.82
1.93
1.91
5.01
2.22
3.95
2.28
10.0 9.5 9.0 8.5 8.0 7.5 7.0 6.5 6.0 5.5 5.0 4.5 4.0 3.5 3.0 2.5 2.0 1.5 1.0 0.5 0.0 -0.5
f1 (ppm)

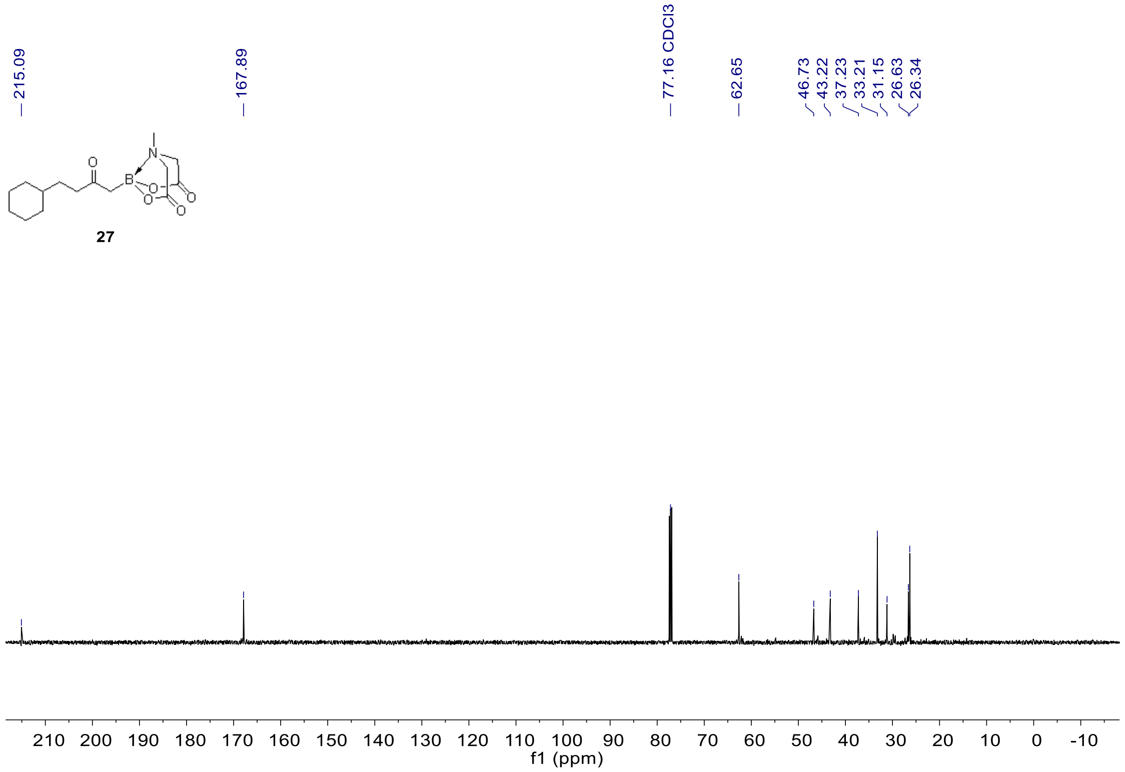
215.09
167.89
77.16 CDCl3
62.65
46.73
43.22
37.23
33.21
31.15
26.63
26.34
27
210 200 190 180 170 160 150 140 130 120 110 100 90 80 70 60 50 40 30 20 10 0 -10
f1 (ppm)

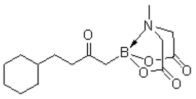

27

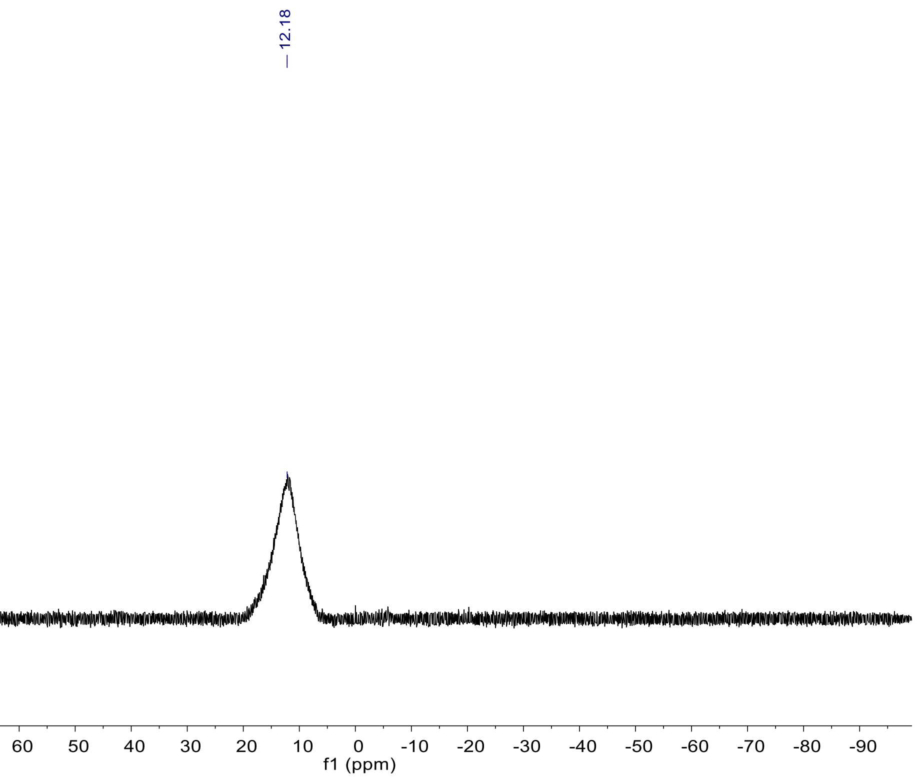
12.18
90
80
70
60
50
40
30
20
10
0
-10
-20
-30
-40
-50
-60
-70
-80
-90
f1 (ppm)

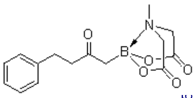

28

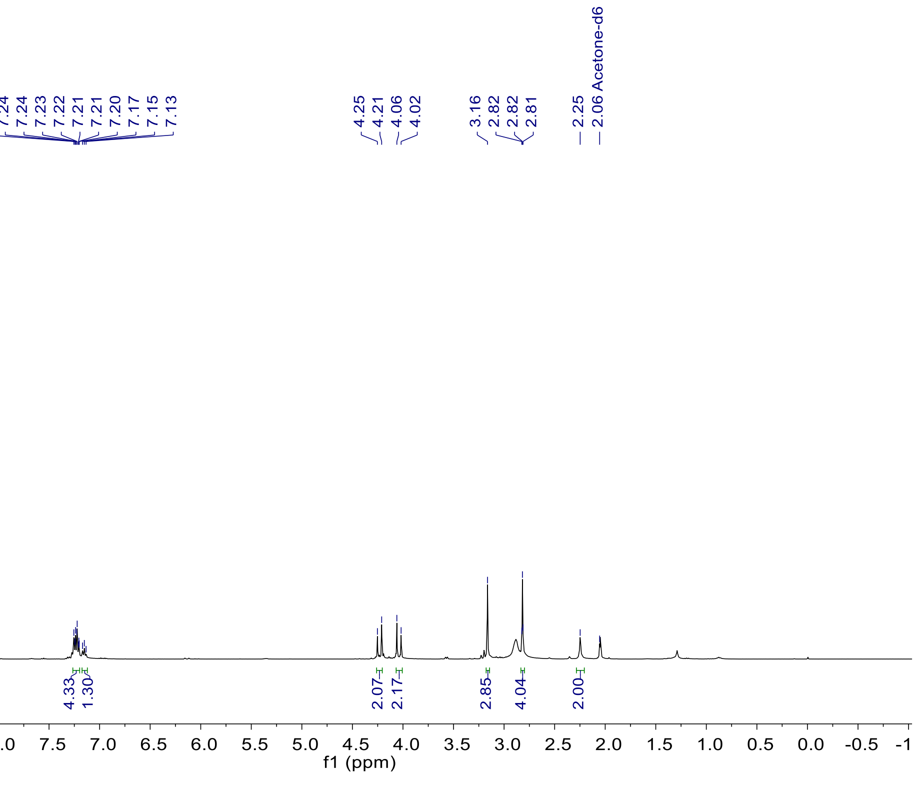
7.25 7.24 7.24 7.23 7.22 7.21 7.21 7.20 7.17 7.15 7.13
4.25 4.21 4.06 4.02
3.16 2.82 2.82 2.81
2.25
2.06 Acetone-d6
4.33 1.30
2.07 2.17
2.85
4.04
2.00
9.5 9.0 8.5 8.0 7.5 7.0 6.5 6.0 5.5 5.0 4.5 4.0 3.5 3.0 2.5 2.0 1.5 1.0 0.5 0.0 -0.5 -1
f1 (ppm)

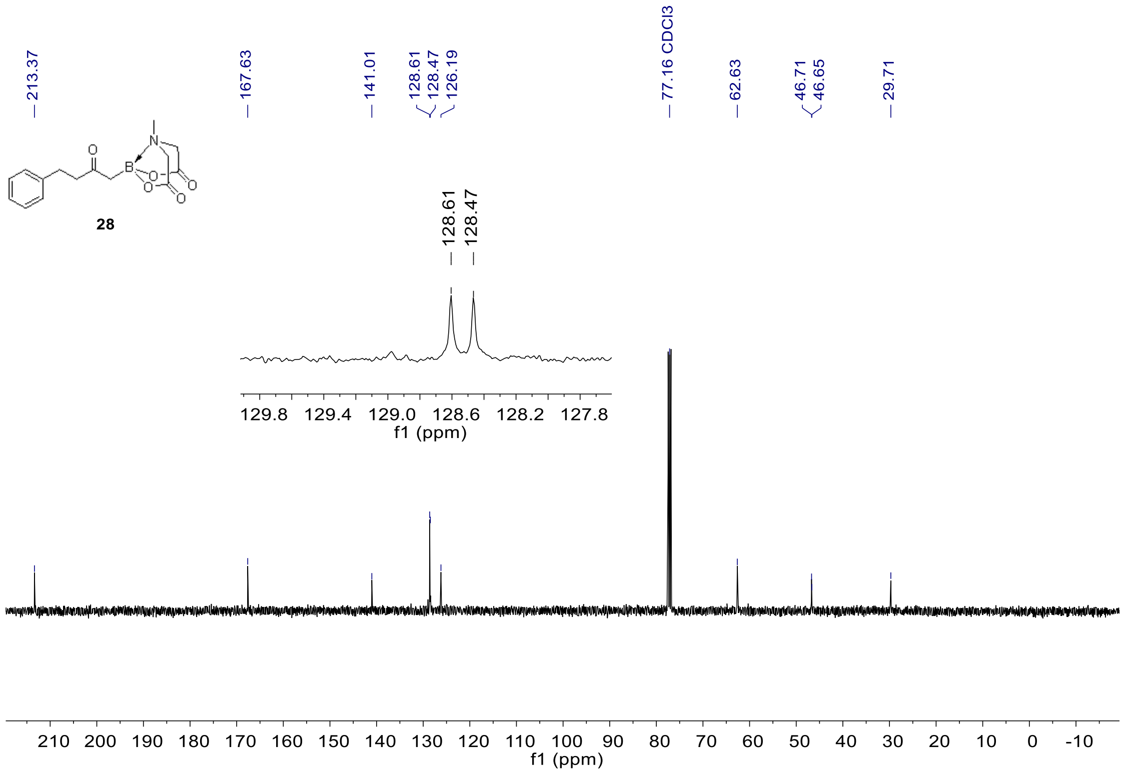
213.37
167.63
141.01
128.61
128.47
126.19
77.16 CDCl3
62.63
46.71
46.65
29.71
128.61
128.47
129.8 129.4 129.0 128.6 128.2 127.8
f1 (ppm)
210 200 190 180 170 160 150 140 130 120 110 100 90 80 70 60 50 40 30 20 10 0 -10
f1 (ppm)

28

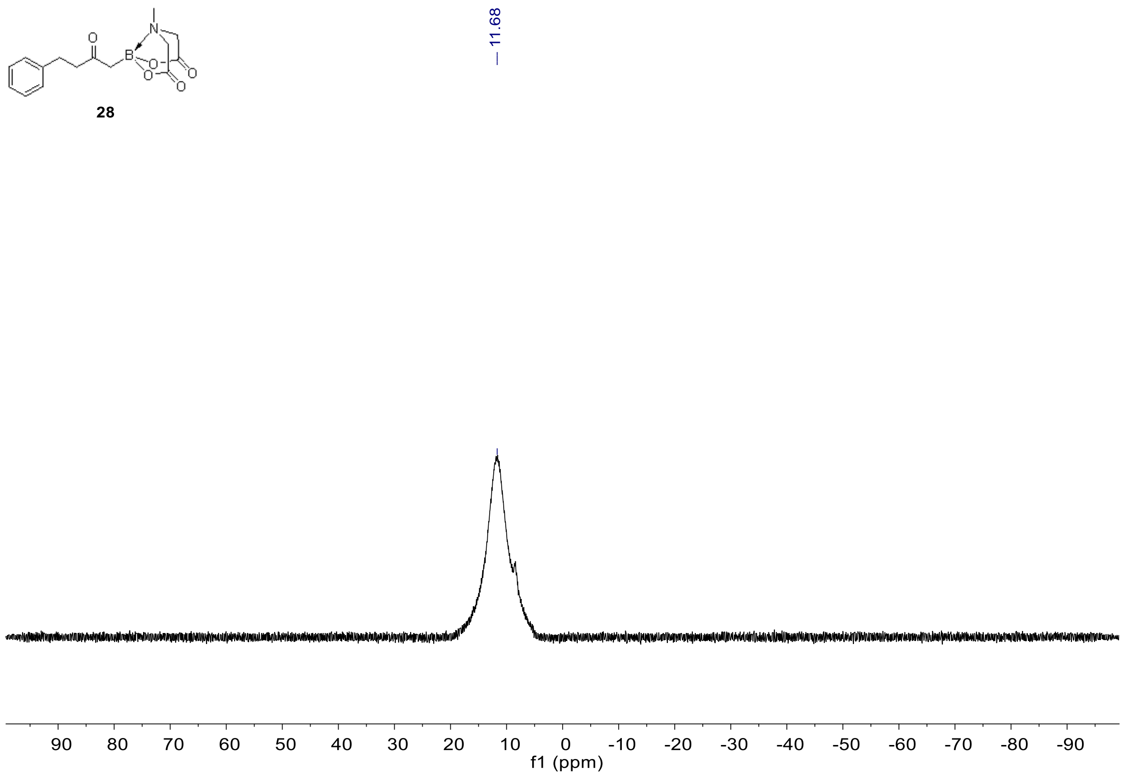
11.68
90 80 70 60 50 40 30 20 10 0 -10 -20 -30 -40 -50 -60 -70 -80 -90
f1 (ppm)

28

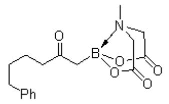

29

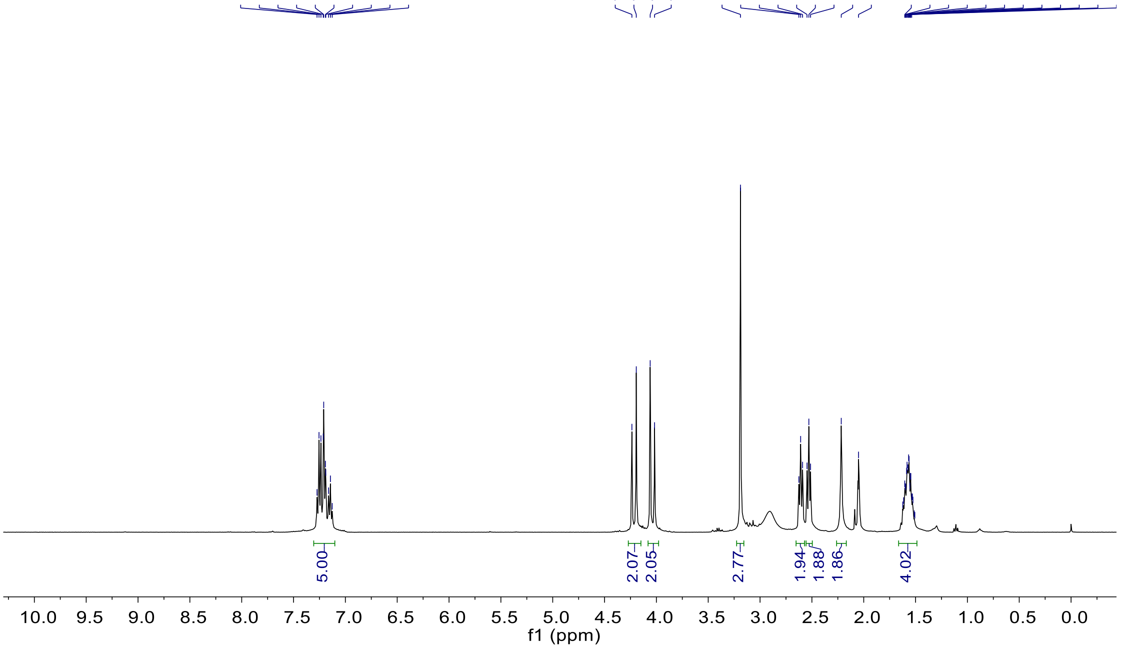
7.27
7.25
7.24
7.21
7.21
7.19
7.19
7.16
7.14
7.13
4.24
4.19
4.06
4.02
3.19
2.63
2.61
2.59
2.55
2.53
2.51
2.22
2.05 Acetone-d6
1.60
1.60
1.59
1.59
1.58
1.57
1.57
1.56
1.56
1.55
1.55
5.00
2.07
2.05
2.77
1.94
1.88
1.86
4.02
10.0 9.5 9.0 8.5 8.0 7.5 7.0 6.5 6.0 5.5 5.0 4.5 4.0 3.5 3.0 2.5 2.0 1.5 1.0 0.5 0.0
f1 (ppm)

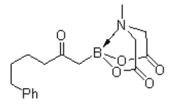

29

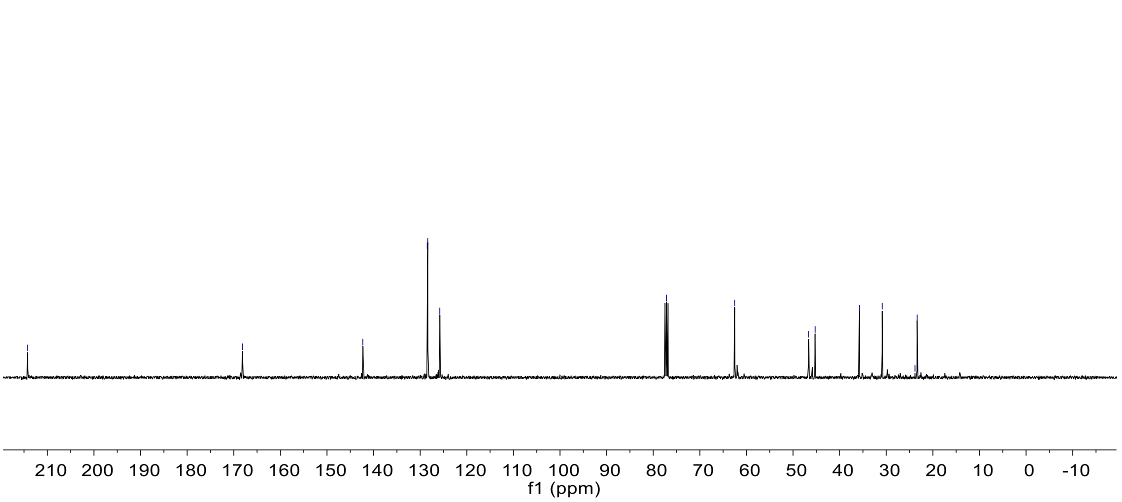
214.22
168.13
142.32
128.48
128.38
125.80
77.16 CDCl3
62.52
46.66
45.25
35.76
30.86
23.83
23.36
210 200 190 180 170 160 150 140 130 120 110 100 90 80 70 60 50 40 30 20 10 0 -10
f1 (ppm)

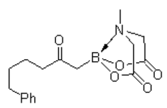

29

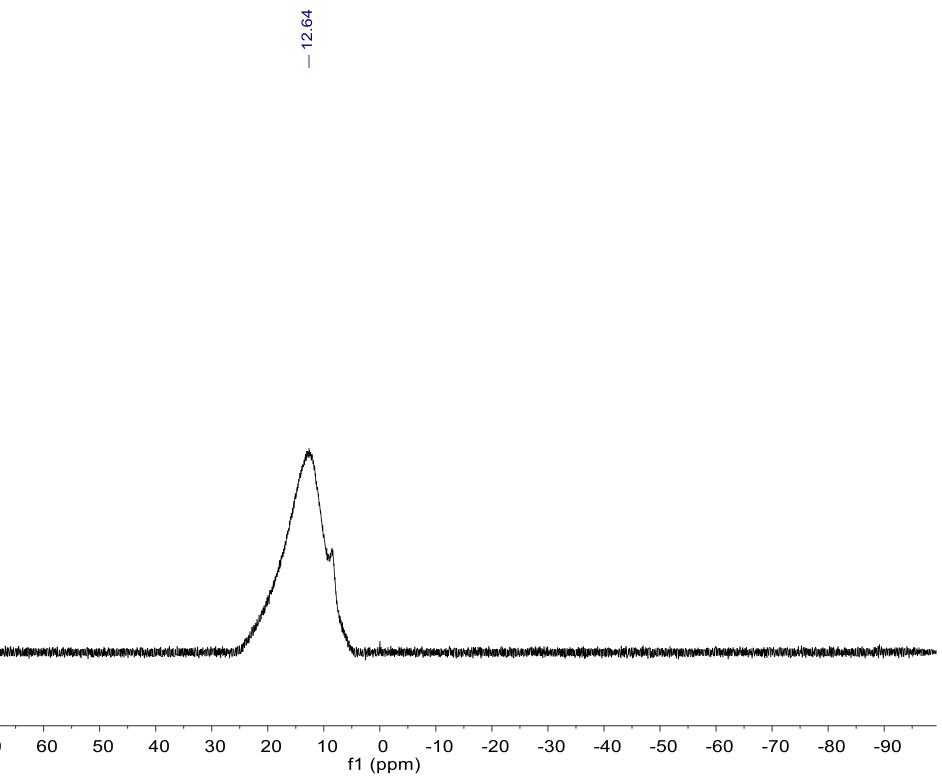

— 12.64
90 80 70 60 50 40 30 20 10 0 -10 -20 -30 -40 -50 -60 -70 -80 -90
f1 (ppm)

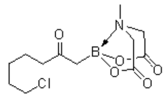

30

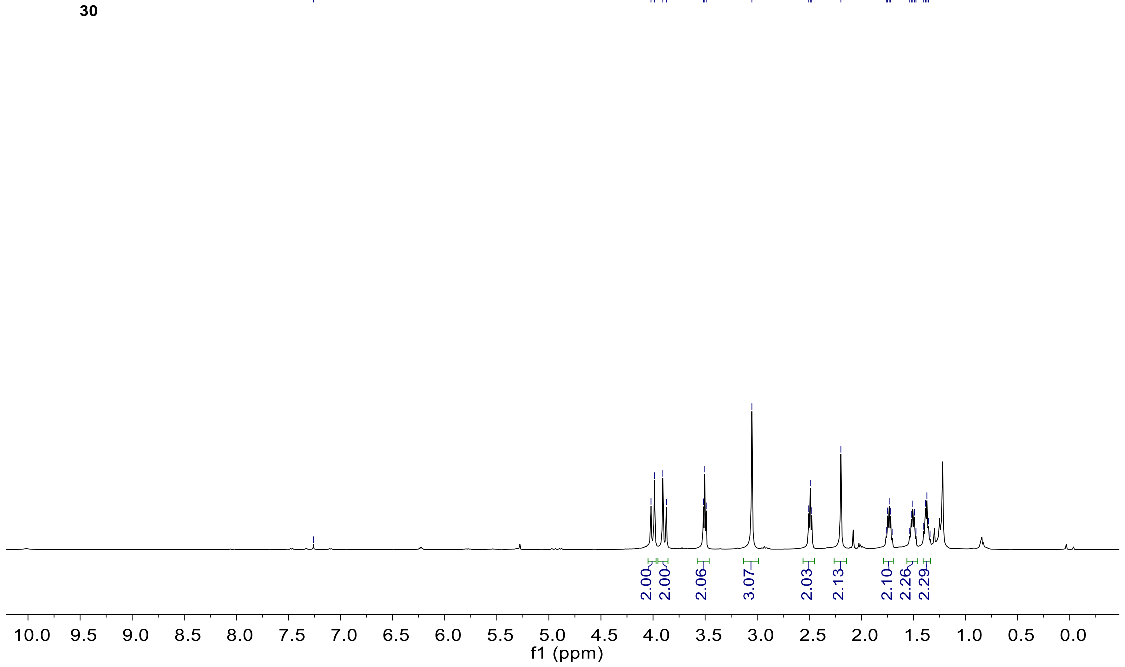

— 7.26 CDCl3
4.02 3.99 3.91 3.87 3.52 3.50 3.49 3.05
2.51 2.49 2.48 2.20
1.76 1.75 1.73 1.72 1.54 1.52 1.51 1.49 1.48 1.40 1.38 1.37
2.00 2.00 2.06 3.07 2.03 2.13 2.10 2.26 2.29
10.0 9.5 9.0 8.5 8.0 7.5 7.0 6.5 6.0 5.5 5.0 4.5 4.0 3.5 3.0 2.5 2.0 1.5 1.0 0.5 0.0
f1 (ppm)

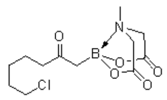

30

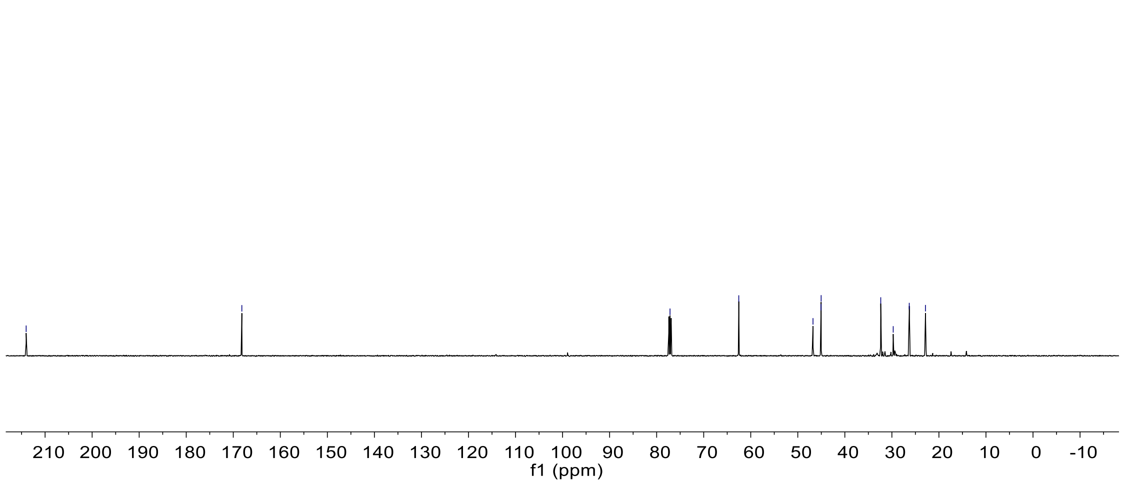

214.02
168.18
77.16 CDCl3
62.54
46.77
45.07
45.03
32.37
29.71
26.30
22.86
210 200 190 180 170 160 150 140 130 120 110 100 90 80 70 60 50 40 30 20 10 0 -10
f1 (ppm)

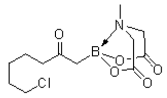

30

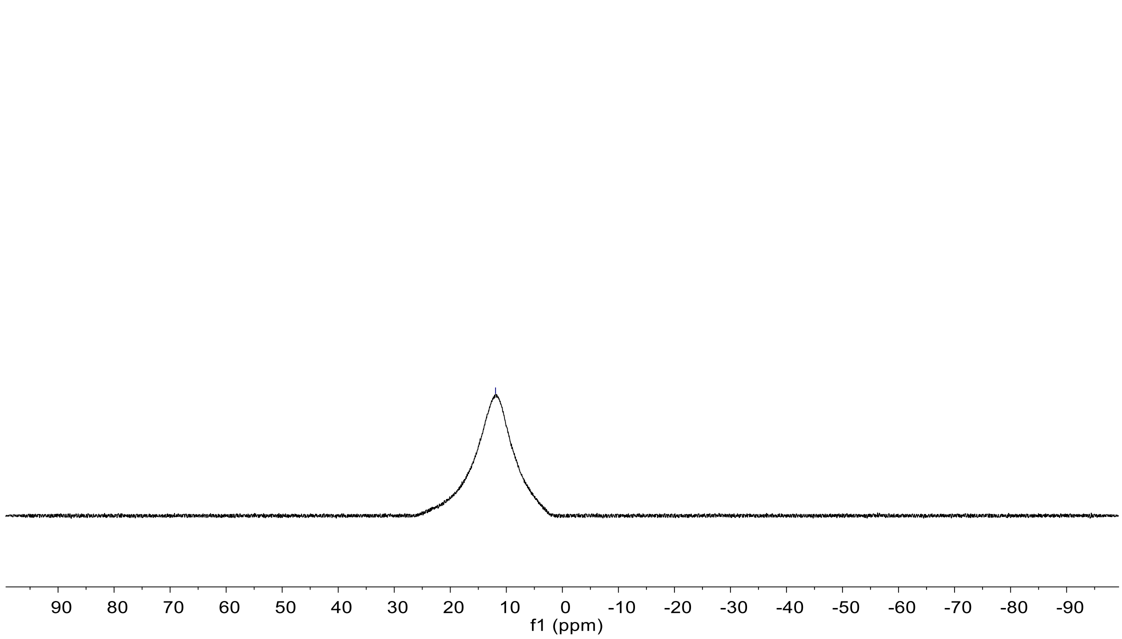

11.92
90 80 70 60 50 40 30 20 10 0 -10 -20 -30 -40 -50 -60 -70 -80 -90
f1 (ppm)

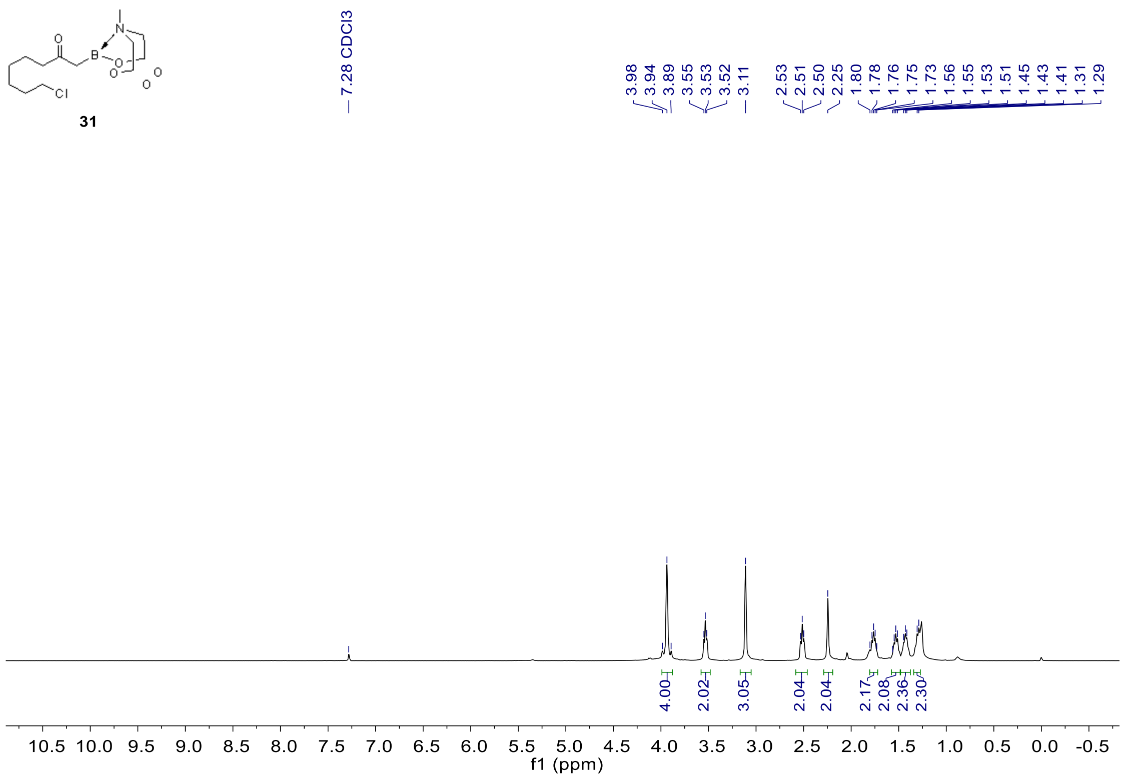
7.28 CDCl3
3.98
3.94
3.89
3.55
3.53
3.52
3.11
2.53
2.51
2.50
2.25
1.80
1.78
1.76
1.75
1.73
1.56
1.55
1.53
1.51
1.45
1.43
1.41
1.31
1.29
31
4.00
2.02
3.05
2.04
2.04
2.17
2.08
2.36
2.30
10.5 10.0 9.5 9.0 8.5 8.0 7.5 7.0 6.5 6.0 5.5 5.0 4.5 4.0 3.5 3.0 2.5 2.0 1.5 1.0 0.5 0.0 -0.5
f1 (ppm)

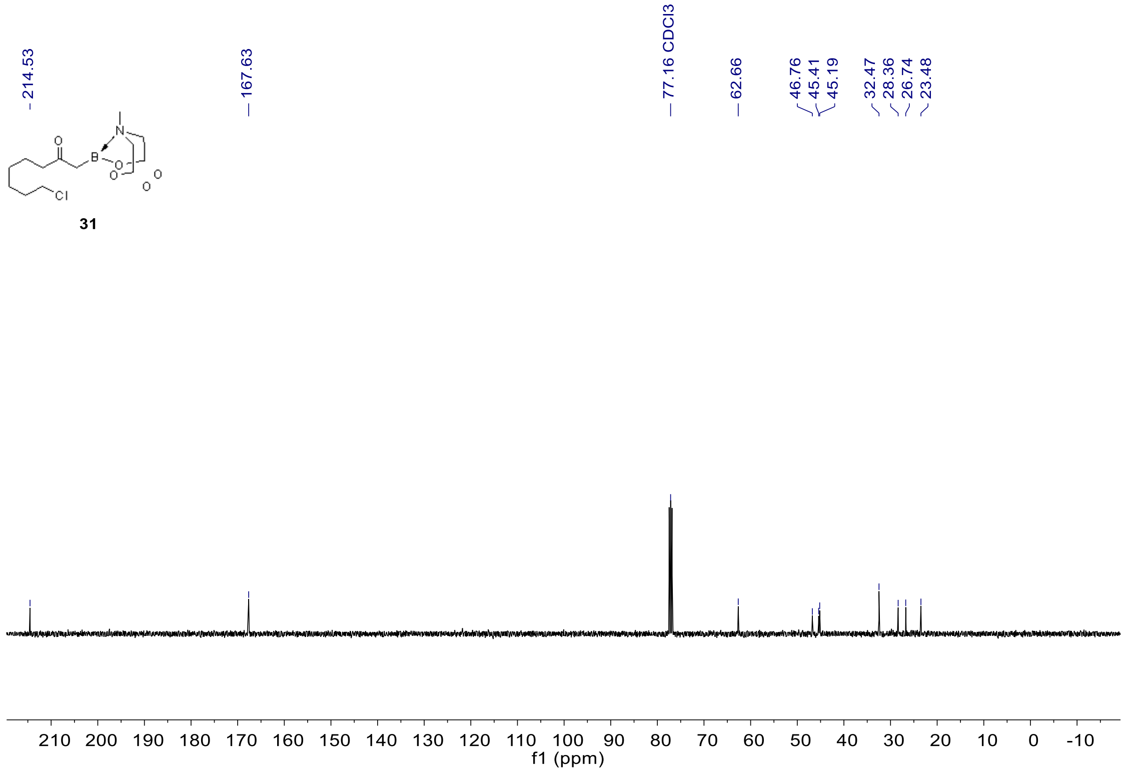
214.53
167.63
77.16 CDCl3
62.66
46.76
45.41
45.19
32.47
28.36
26.74
23.48
31
210 200 190 180 170 160 150 140 130 120 110 100 90 80 70 60 50 40 30 20 10 0 -10
f1 (ppm)

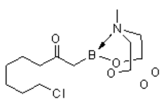

31

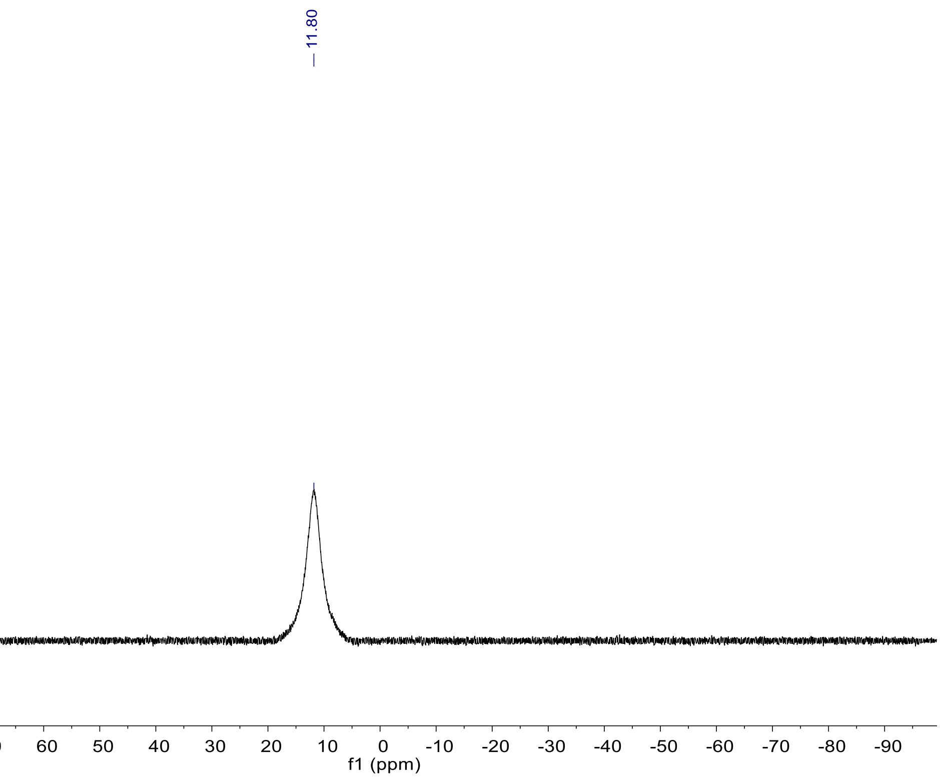

11.80
90 80 70 60 50 40 30 20 10 0 -10 -20 -30 -40 -50 -60 -70 -80 -90
f1 (ppm)

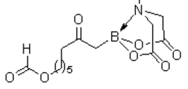

32

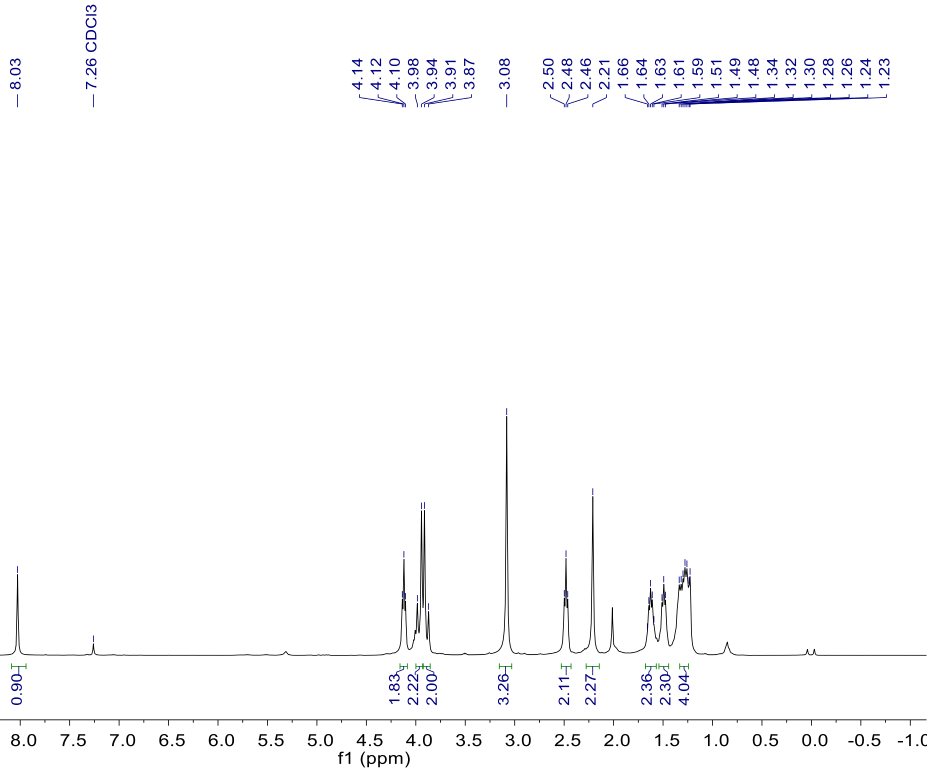

8.03
7.26 CDCl3
4.14 4.12 4.10 3.98 3.94 3.91 3.87
3.08
2.50 2.48 2.46 2.21 1.66 1.64 1.63 1.61 1.59 1.51 1.49 1.48 1.34 1.32 1.30 1.28 1.26 1.24 1.23
0.90 1.83 2.22 2.00 3.26 2.11 2.27 2.36 2.30 4.04
10.0 9.5 9.0 8.5 8.0 7.5 7.0 6.5 6.0 5.5 5.0 4.5 4.0 3.5 3.0 2.5 2.0 1.5 1.0 0.5 0.0 -0.5 -1.0
f1 (ppm)

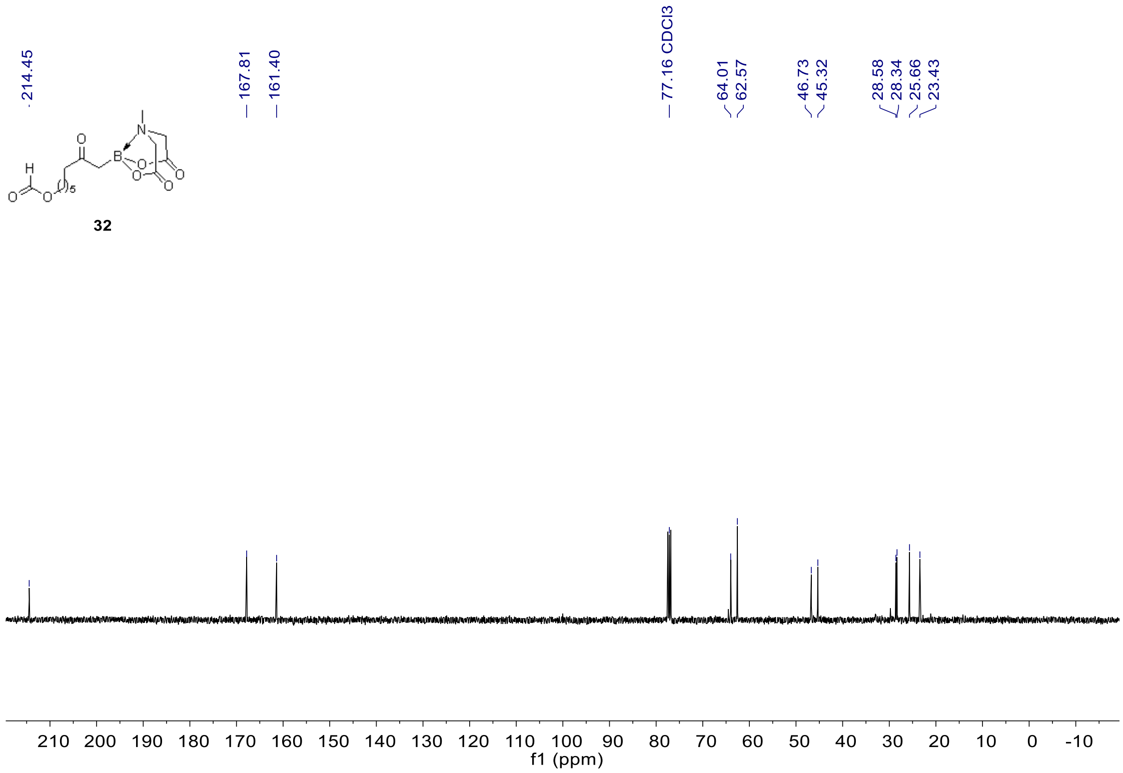
214.45
167.81
161.40
77.16 CDCl3
64.01
62.57
46.73
45.32
28.58
28.34
25.66
23.43
32
210 200 190 180 170 160 150 140 130 120 110 100 90 80 70 60 50 40 30 20 10 0 -10
f1 (ppm)

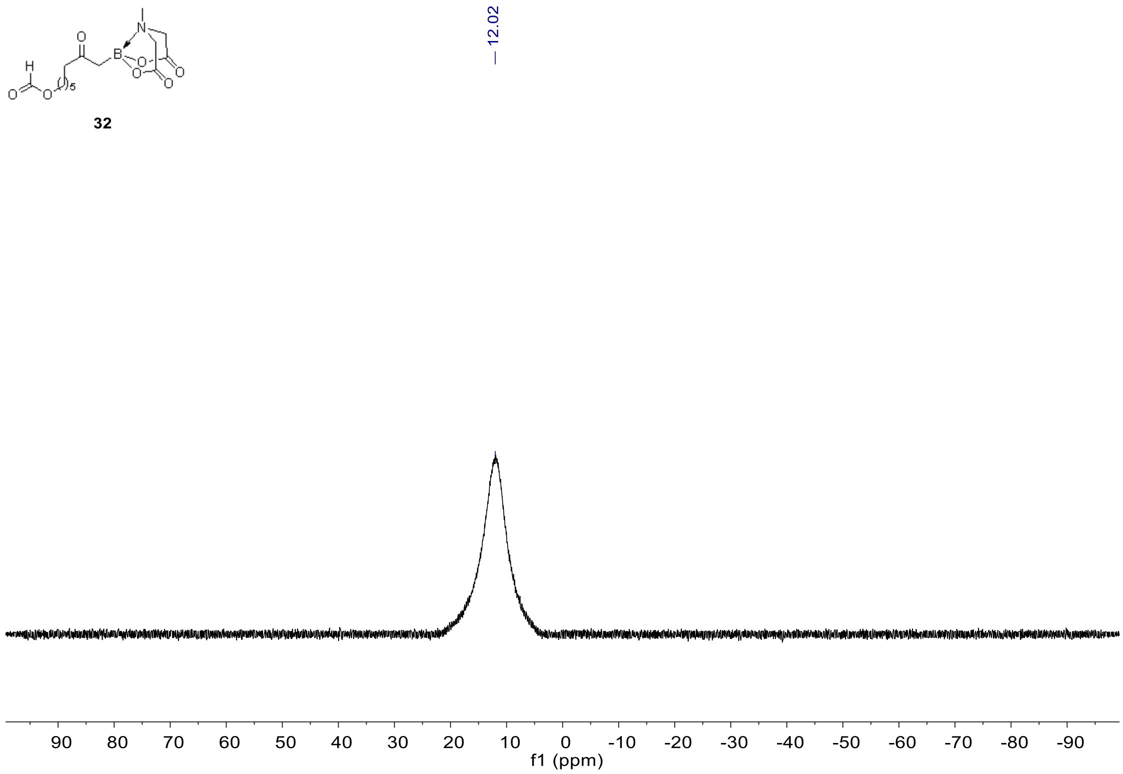
12.02
32
90 80 70 60 50 40 30 20 10 0 -10 -20 -30 -40 -50 -60 -70 -80 -90
f1 (ppm)

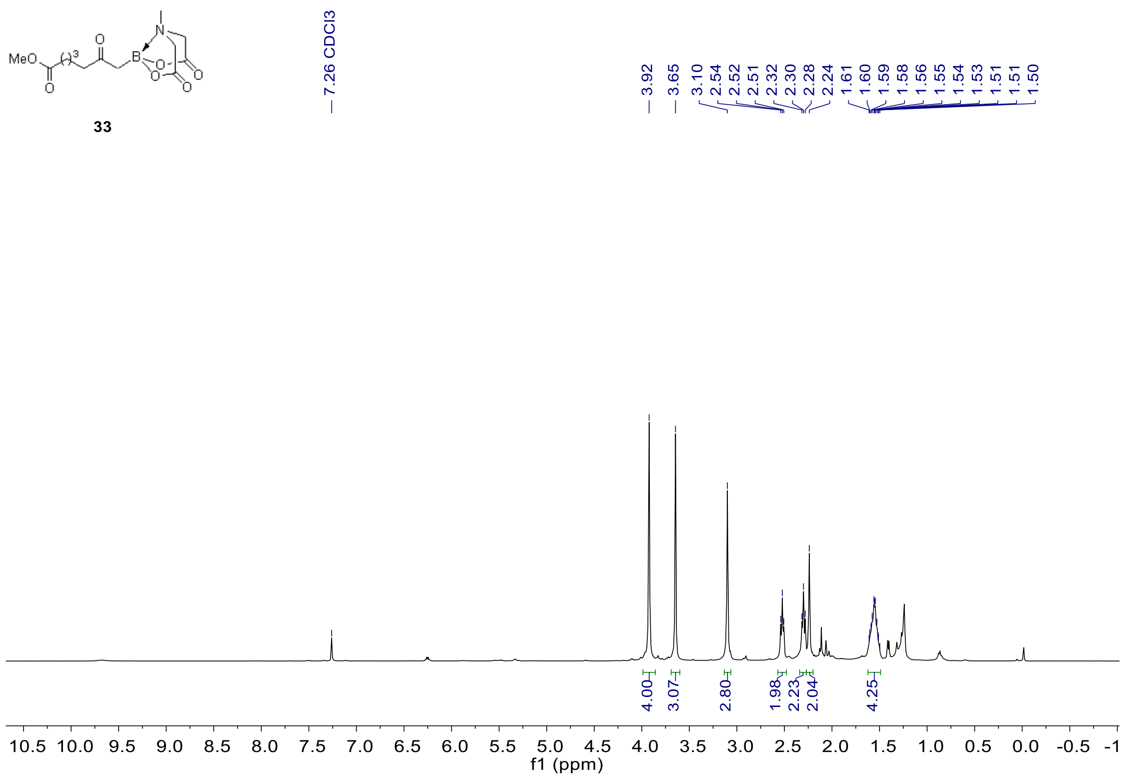

MeO
3
O
B
N
O
33
7.26 CDCl3
3.92
3.65
3.10
2.54
2.52
2.51
2.32
2.30
2.28
2.24
1.61
1.60
1.59
1.58
1.56
1.55
1.54
1.53
1.51
1.51
1.50
4.00
3.07
2.80
1.98
2.23
2.04
4.25
10.5 10.0 9.5 9.0 8.5 8.0 7.5 7.0 6.5 6.0 5.5 5.0 4.5 4.0 3.5 3.0 2.5 2.0 1.5 1.0 0.5 0.0 -0.5 -1
f1 (ppm)

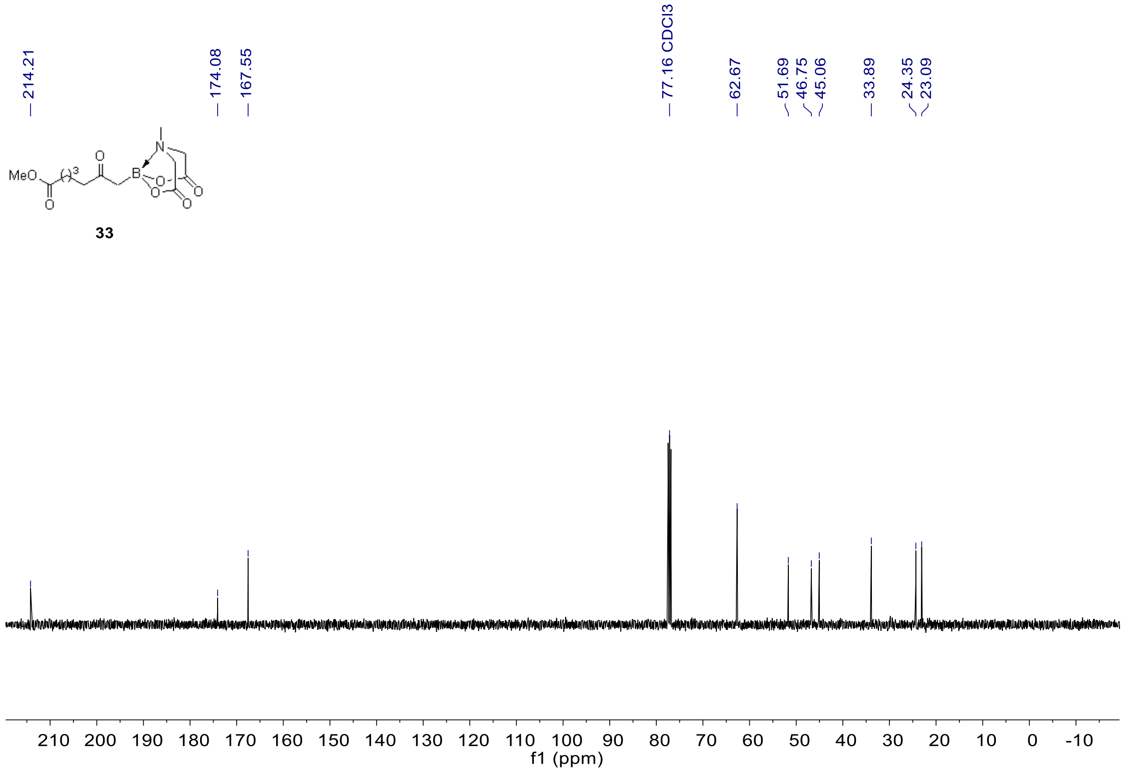

214.21
174.08
167.55
77.16 CDCl3
62.67
51.69
46.75
45.06
33.89
24.35
23.09
MeO
3
O
B
N
O
33
210 200 190 180 170 160 150 140 130 120 110 100 90 80 70 60 50 40 30 20 10 0 -10
f1 (ppm)

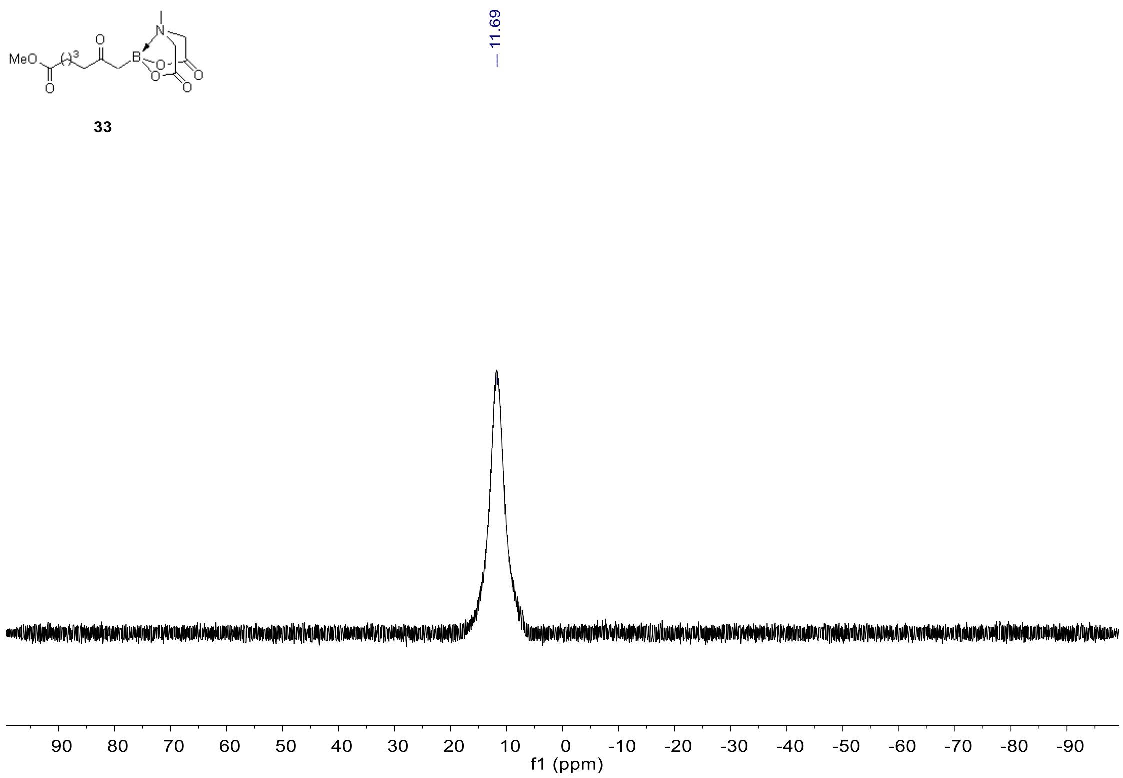
MeO
33
— 11.69
90 80 70 60 50 40 30 20 10 0 -10 -20 -30 -40 -50 -60 -70 -80 -90
f1 (ppm)

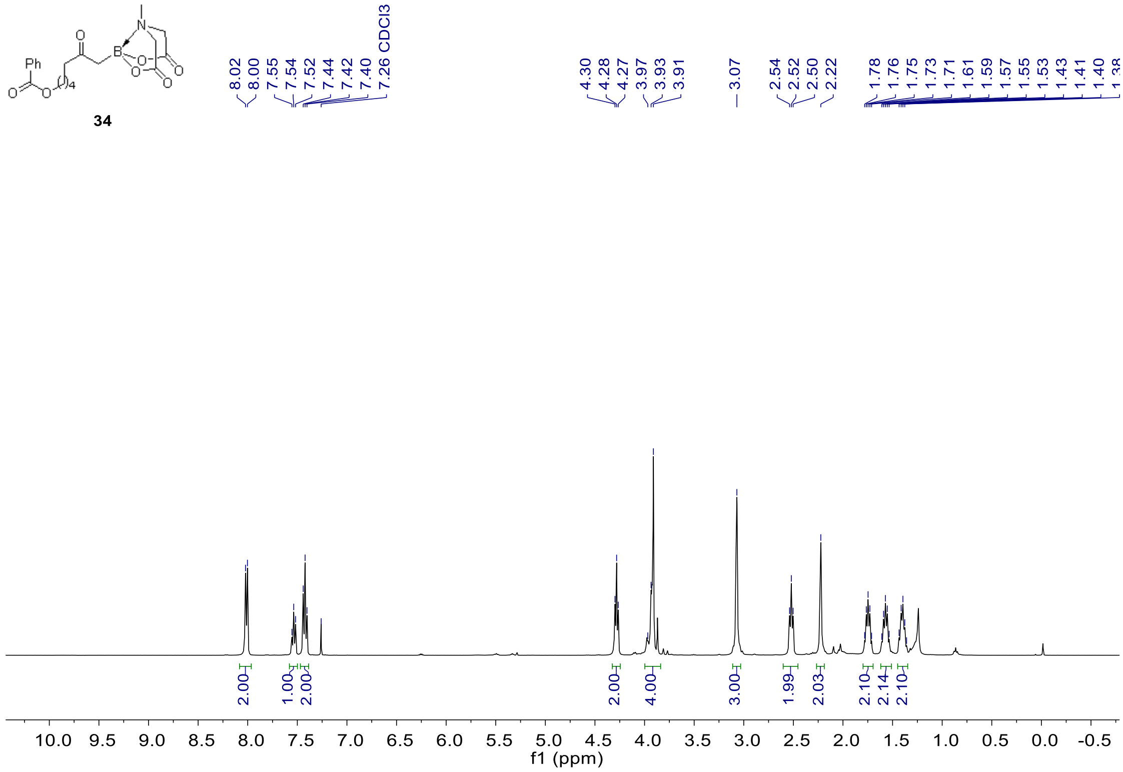
Ph
34
8.02 8.00 7.55 7.54 7.52 7.44 7.42 7.40 7.26 CDCl3
4.30 4.28 4.27 3.97 3.93 3.91
3.07
2.54 2.52 2.50 2.22
1.78 1.76 1.75 1.73 1.71 1.61 1.59 1.57 1.55 1.53 1.43 1.41 1.40
2.00 1.00 2.00 2.00 4.00 3.00 1.99 2.03 2.10 2.14 2.10
10.0 9.5 9.0 8.5 8.0 7.5 7.0 6.5 6.0 5.5 5.0 4.5 4.0 3.5 3.0 2.5 2.0 1.5 1.0 0.5 0.0 -0.5
f1 (ppm)

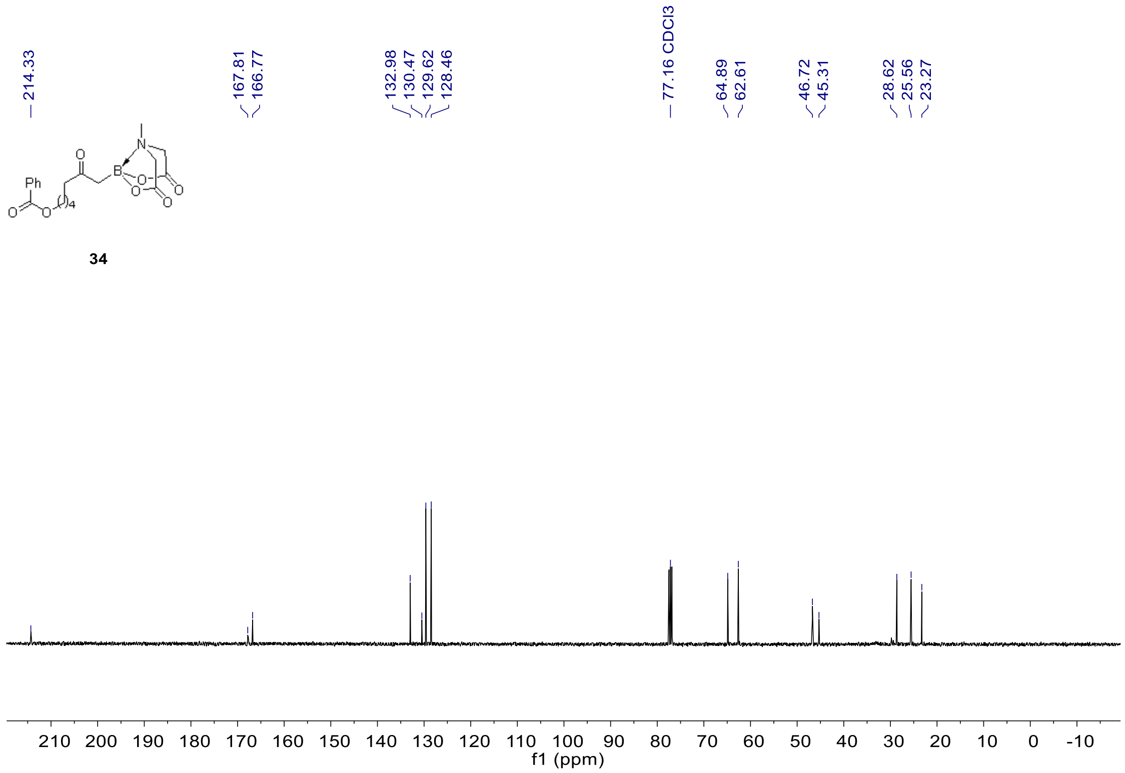
214.33
167.81
166.77
132.98
130.47
129.62
128.46
77.16 CDCl3
64.89
62.61
46.72
45.31
28.62
25.56
23.27
Ph
O
O
O
B
N
O
O
O
O
4
210 200 190 180 170 160 150 140 130 120 110 100 90 80 70 60 50 40 30 20 10 0 -10
f1 (ppm)

34

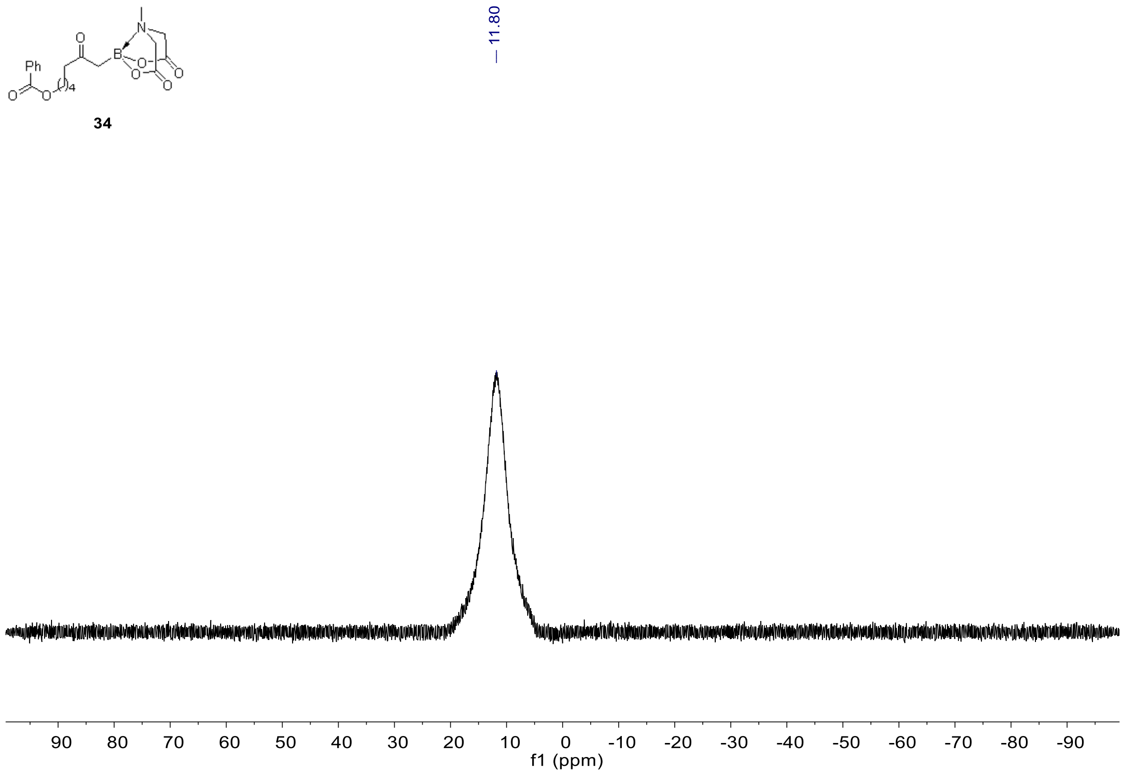
11.80
Ph
O
O
O
B
N
O
O
O
O
4
90 80 70 60 50 40 30 20 10 0 -10 -20 -30 -40 -50 -60 -70 -80 -90
f1 (ppm)

34

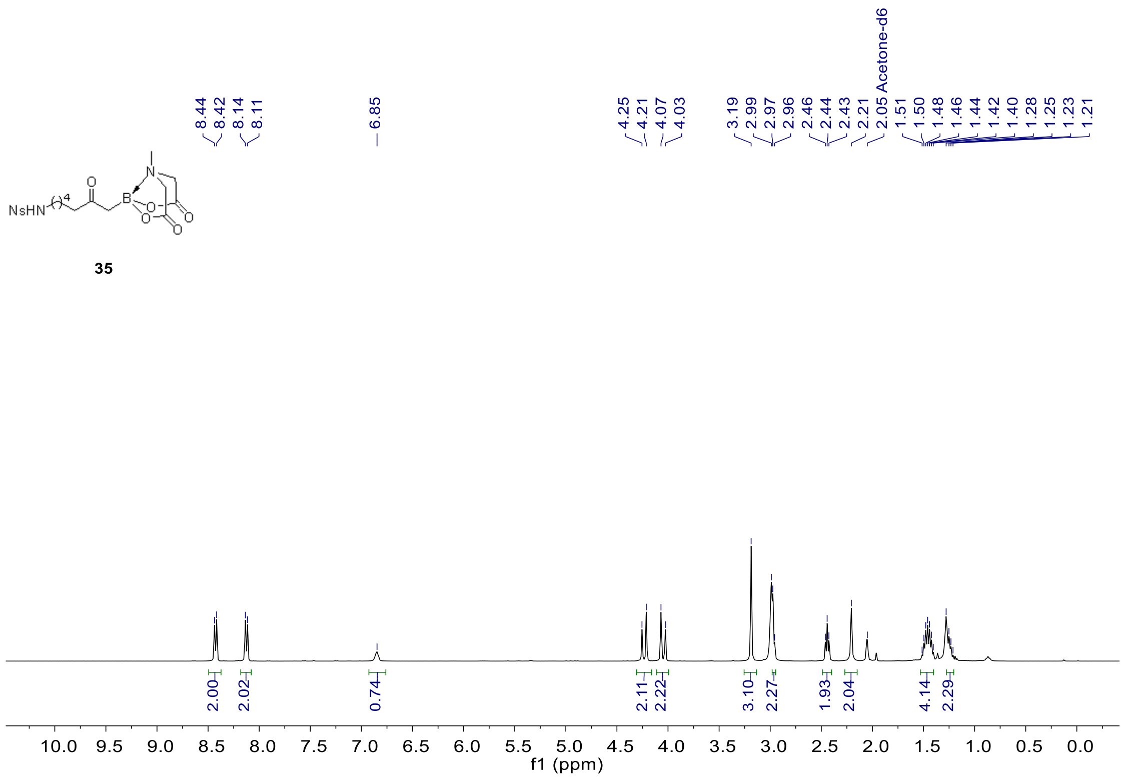
8.44
8.42
8.14
8.11
6.85
4.25
4.21
4.07
4.03
3.19
2.99
2.97
2.96
2.46
2.44
2.43
2.21
2.05 Acetone-d6
1.51
1.50
1.48
1.46
1.44
1.42
1.40
1.28
1.25
1.23
1.21
NsHN
4
O
B
N
O
O
O
O
35
2.00
2.02
0.74
2.11
2.22
3.10
2.27
1.93
2.04
4.14
2.29
f1 (ppm)

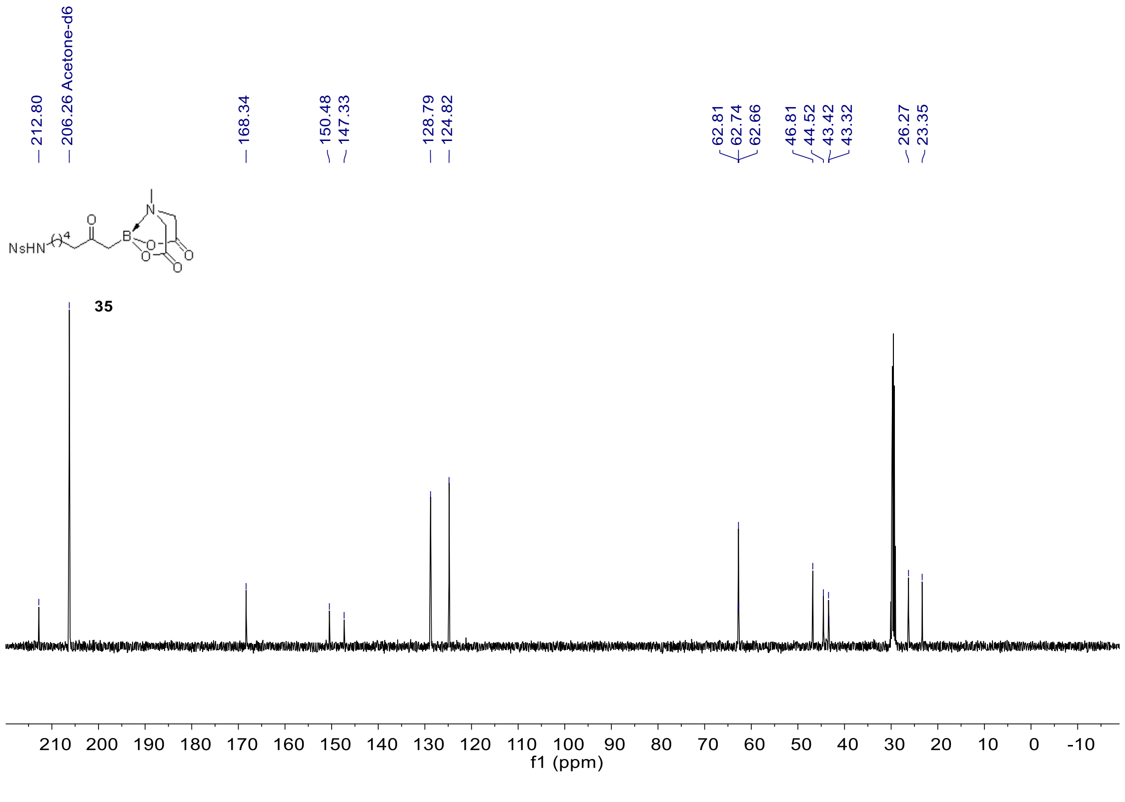
212.80
206.26 Acetone-d6
168.34
150.48
147.33
128.79
124.82
62.81
62.74
62.66
46.81
44.52
43.42
43.32
26.27
23.35
NsHN
4
O
B
N
O
O
O
O
35
f1 (ppm)

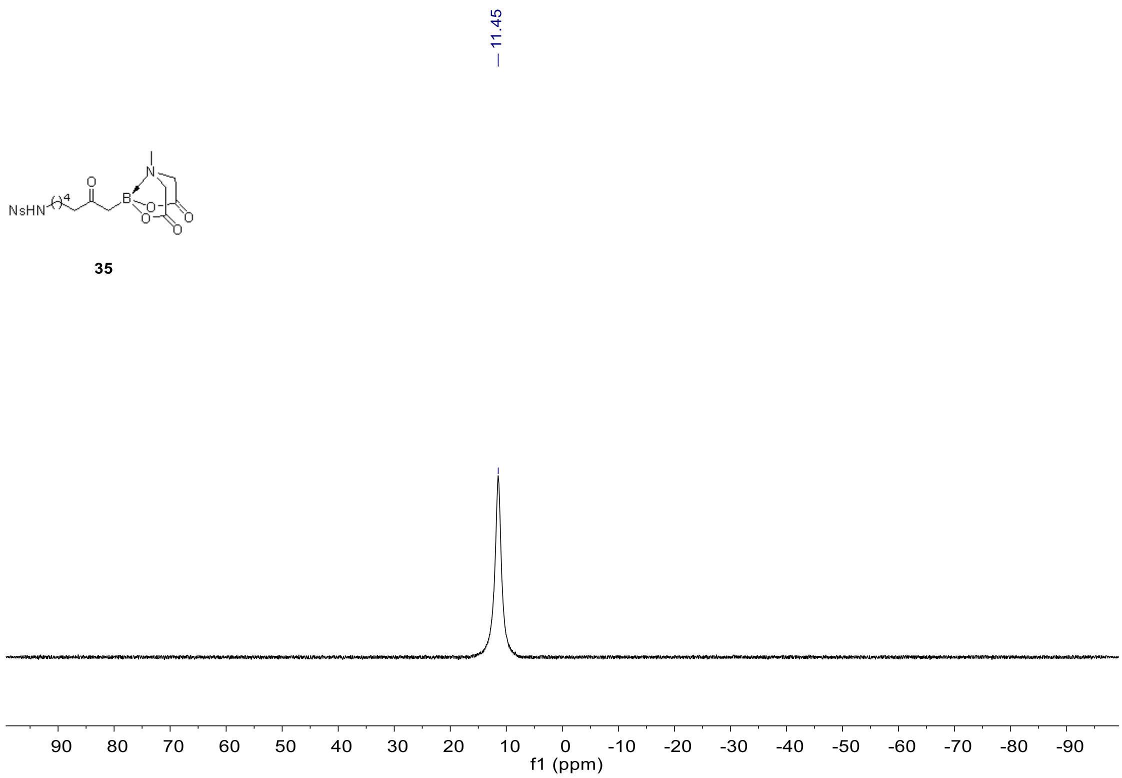

11.45
NsHN
90 80 70 60 50 40 30 20 10 0 -10 -20 -30 -40 -50 -60 -70 -80 -90
f1 (ppm)

35

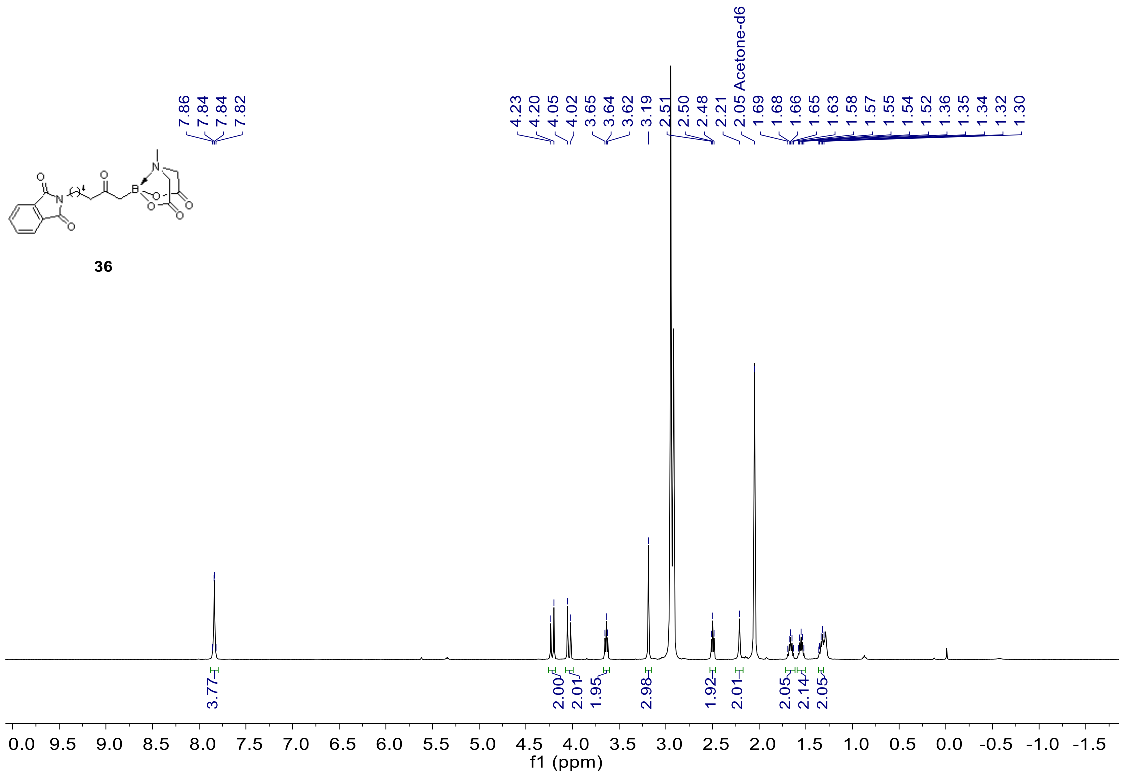

7.86 7.84 7.84 7.82
4.23 4.20 4.05 4.02 3.65 3.64 3.62 3.19 2.51 2.50 2.48 2.21 2.05 Acetone-d6 1.69 1.68 1.66 1.65 1.63 1.58 1.57 1.55 1.54 1.52 1.36 1.35 1.34 1.32 1.30
3.77 2.00 2.01 1.95 2.98 1.92 2.01 2.05 2.14 2.05
0.0 9.5 9.0 8.5 8.0 7.5 7.0 6.5 6.0 5.5 5.0 4.5 4.0 3.5 3.0 2.5 2.0 1.5 1.0 0.5 0.0 -0.5 -1.0 -1.5
f1 (ppm)

36

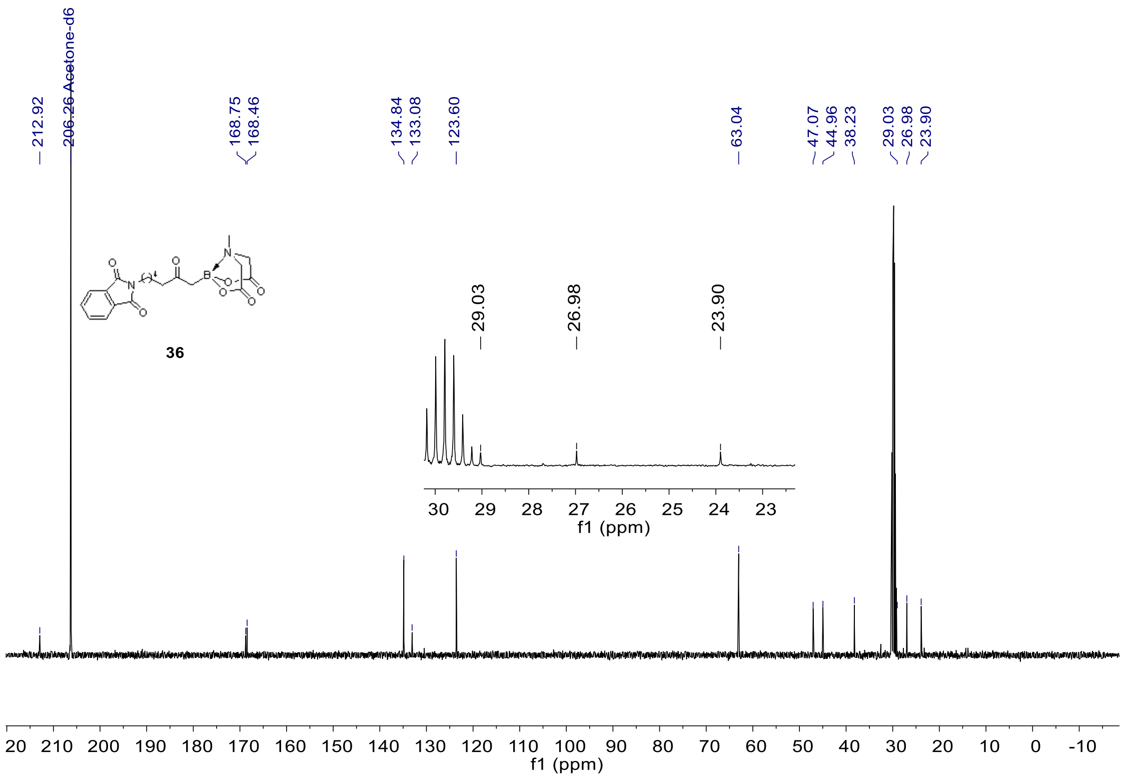

212.92
206.26 Acetone-d6
168.75
168.46
134.84
133.08
123.60
63.04
47.07
44.96
38.23
29.03
26.98
23.90
36
29.03
26.98
23.90
f1 (ppm)
f1 (ppm)

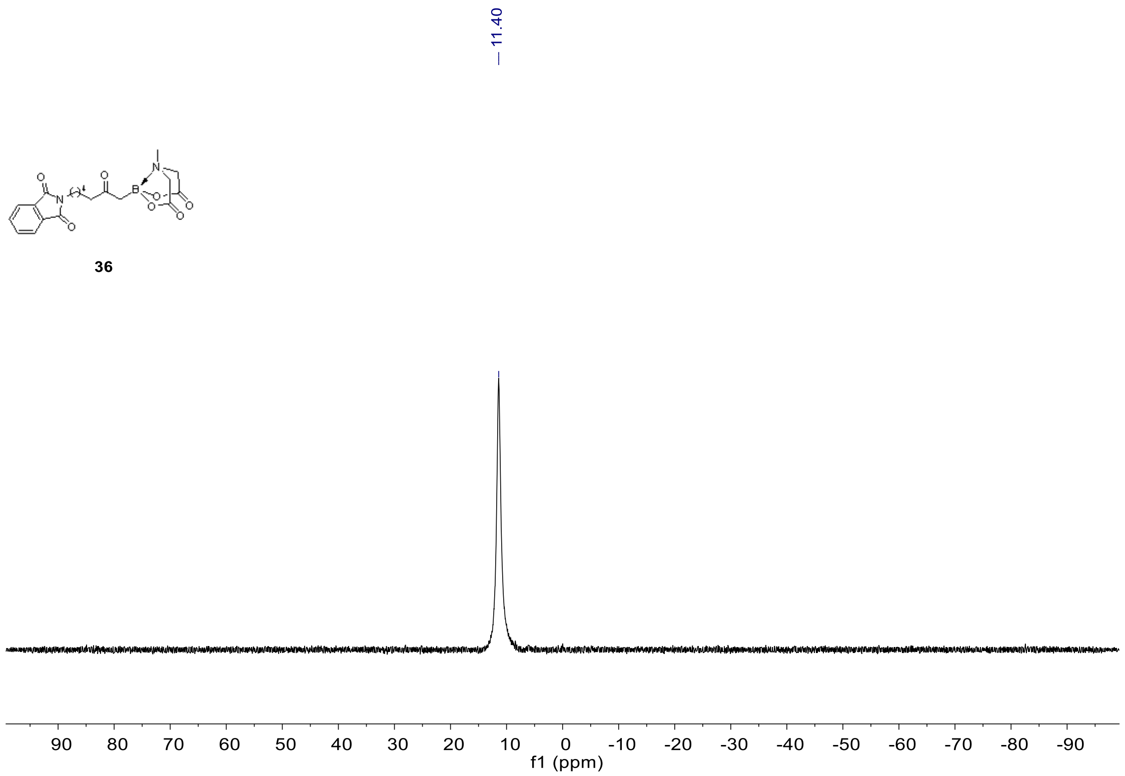

11.40
36
f1 (ppm)

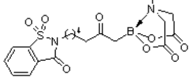

37

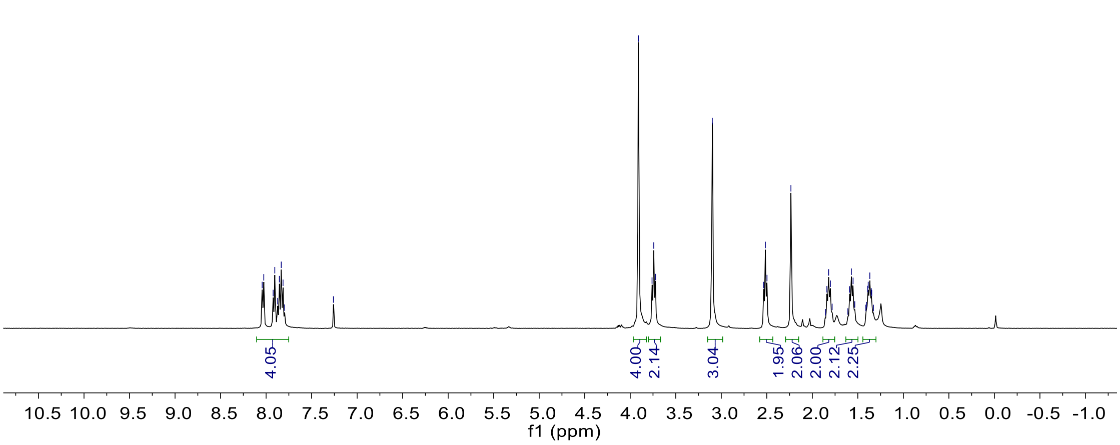
8.04 8.03 7.92 7.90 7.87 7.85 7.83 7.81 7.80 7.26 CDCl3
3.91 3.76 3.74 3.72
3.10
2.53 2.52 2.50 2.23 1.84 1.82 1.80 1.78 1.61 1.59 1.57 1.55 1.53 1.41 1.41 1.39 1.39 1.37 1.35 1.35
4.05
4.00 2.14
3.04
1.95 2.06 2.00 2.12 2.25
10.5 10.0 9.5 9.0 8.5 8.0 7.5 7.0 6.5 6.0 5.5 5.0 4.5 4.0 3.5 3.0 2.5 2.0 1.5 1.0 0.5 0.0 -0.5 -1.0
f1 (ppm)

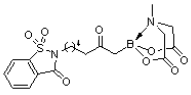

37

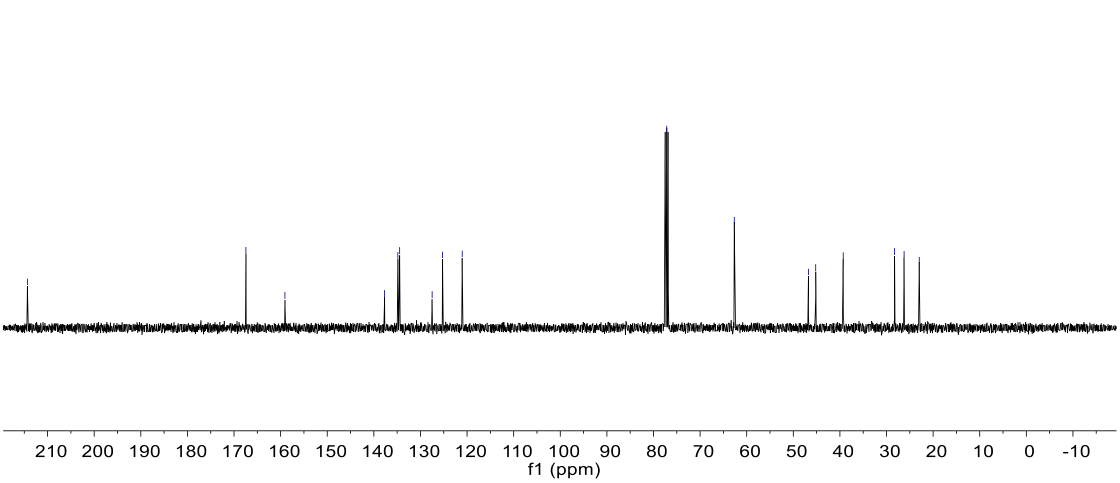
214.33
167.45
159.08
137.69 134.85 134.46 127.49 125.26 121.04
77.16 CDCl3
62.67
46.76 45.19 39.28
28.27 26.23 22.98
210 200 190 180 170 160 150 140 130 120 110 100 90 80 70 60 50 40 30 20 10 0 -10
f1 (ppm)

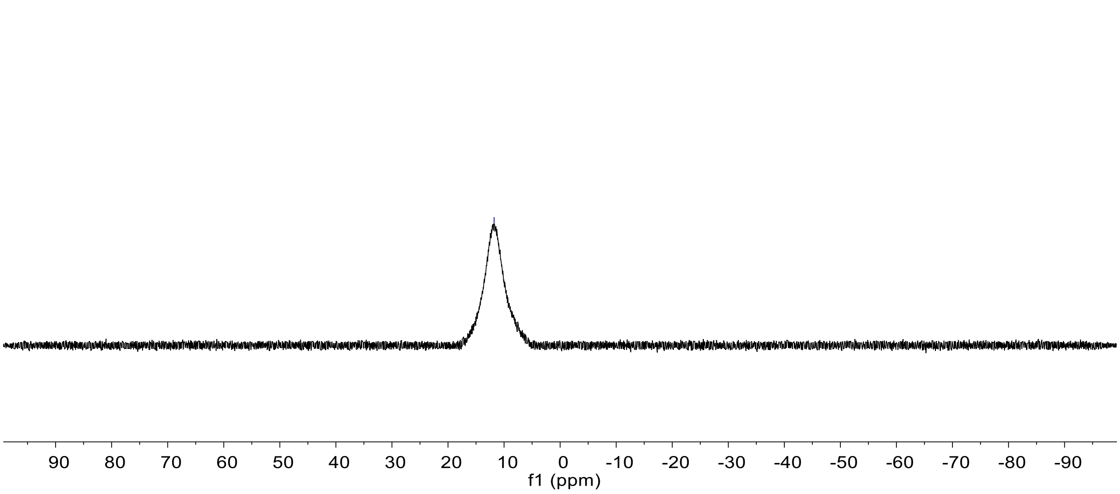
11.78
90 80 70 60 50 40 30 20 10 0 -10 -20 -30 -40 -50 -60 -70 -80 -90
f1 (ppm)

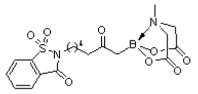

37

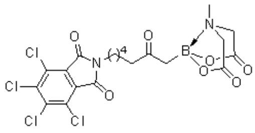

38

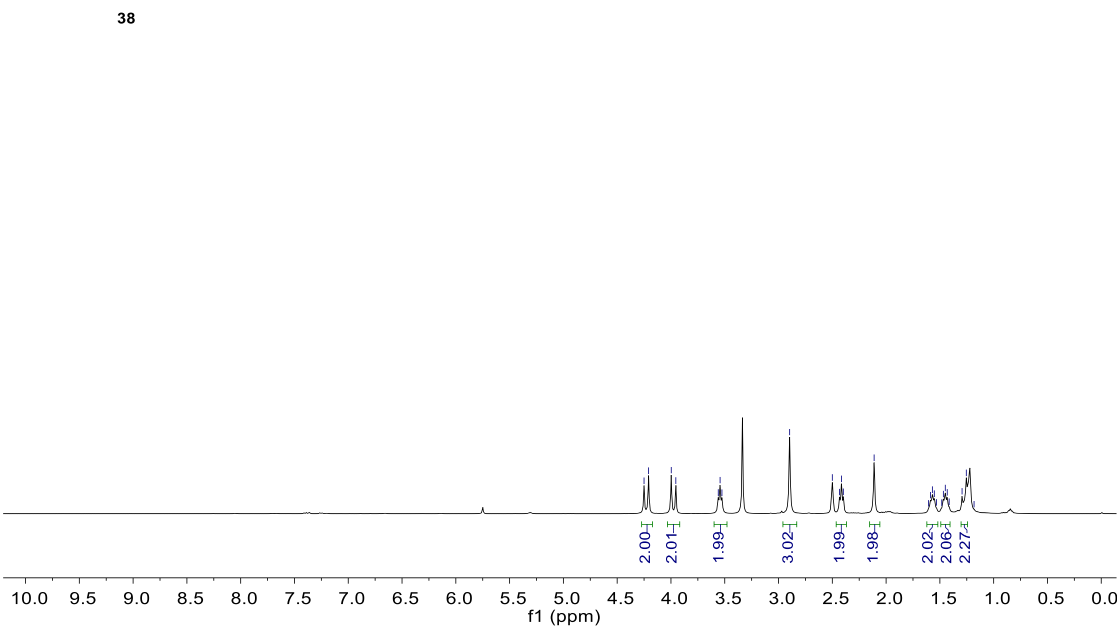
4.25
4.21
4.00
3.95
3.56
3.54
3.53
2.90
2.50 DMSO-d6
2.43
2.42
2.40
2.11
1.59
1.57
1.55
1.53
1.48
1.47
1.45
1.43
1.41
1.29
1.25
2.00
2.01
1.99
3.02
1.99
1.98
2.02
2.06
2.27
10.0 9.5 9.0 8.5 8.0 7.5 7.0 6.5 6.0 5.5 5.0 4.5 4.0 3.5 3.0 2.5 2.0 1.5 1.0 0.5 0.0
f1 (ppm)

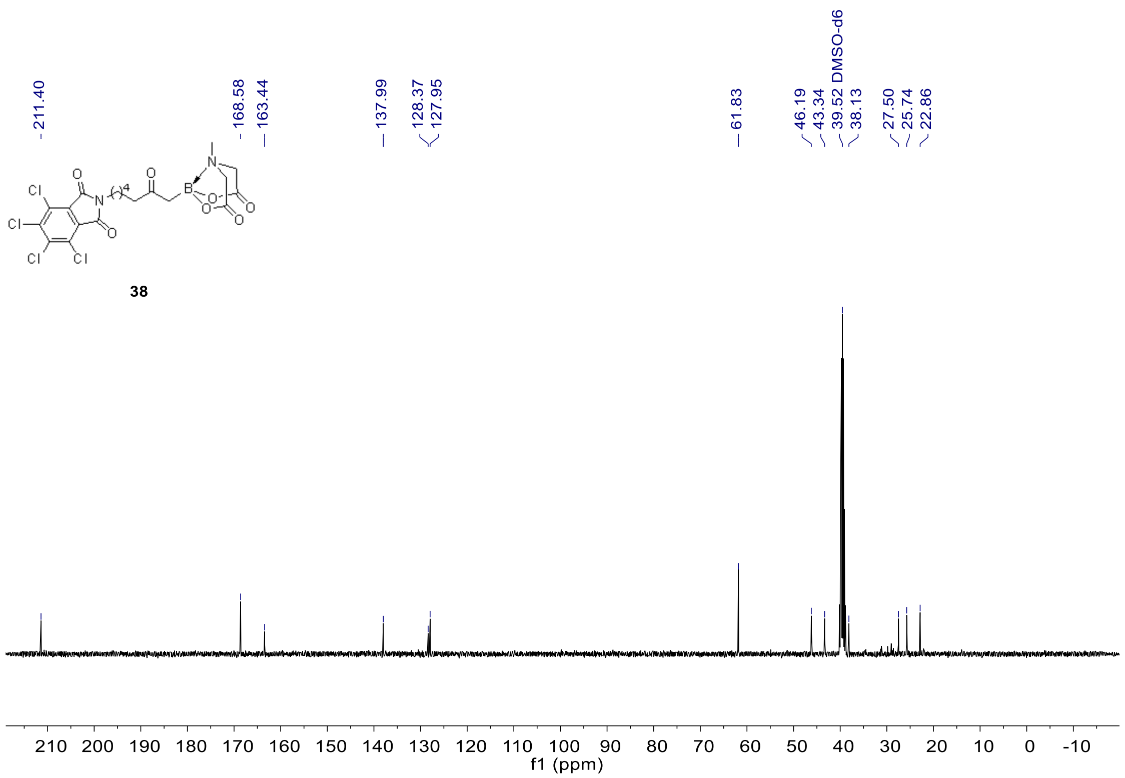
211.40
168.58
163.44
137.99
128.37
127.95
61.83
46.19
43.34
39.52 DMSO-d6
38.13
27.50
25.74
22.86
f1 (ppm)

38

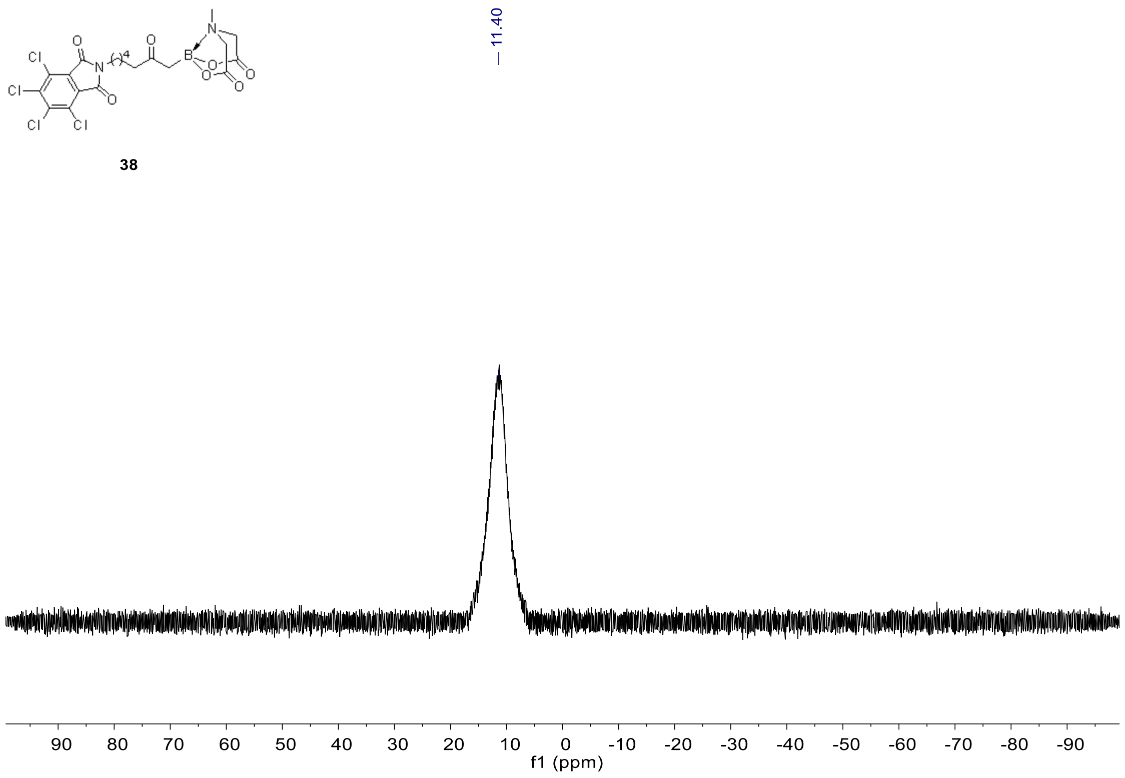
11.40
f1 (ppm)

38

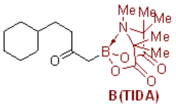
Me Me Me
N
B
O
O
Me
Me
O
O
B(TIDA)

39

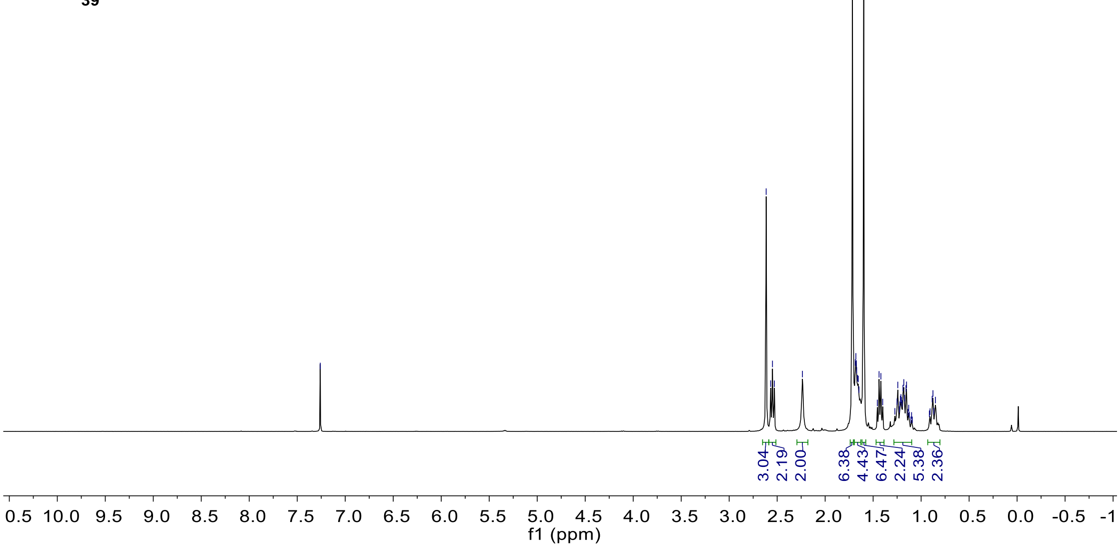
7.26 CDCl3
2.61
2.57
2.55
2.53
2.24
1.72
1.69
1.68
1.67
1.67
1.66
1.66
1.65
1.60
1.46
1.44
1.42
1.40
1.27
1.25
1.24
1.23
1.22
1.22
1.21
1.20
1.20
1.19
1.18
1.17
1.16
1.15
1.14
1.13
1.12
1.11
1.10
1.09
0.91
0.91
0.89
0.88
0.87
0.85
3.04
2.19
2.00
6.38
4.43
6.47
2.24
5.38
2.36
0.5 10.0 9.5 9.0 8.5 8.0 7.5 7.0 6.5 6.0 5.5 5.0 4.5 4.0 3.5 3.0 2.5 2.0 1.5 1.0 0.5 0.0 -0.5 -1
f1 (ppm)

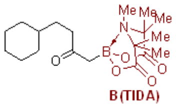
Me Me Me
N
B
O
O
Me
Me
O
O
B(TIDA)

39

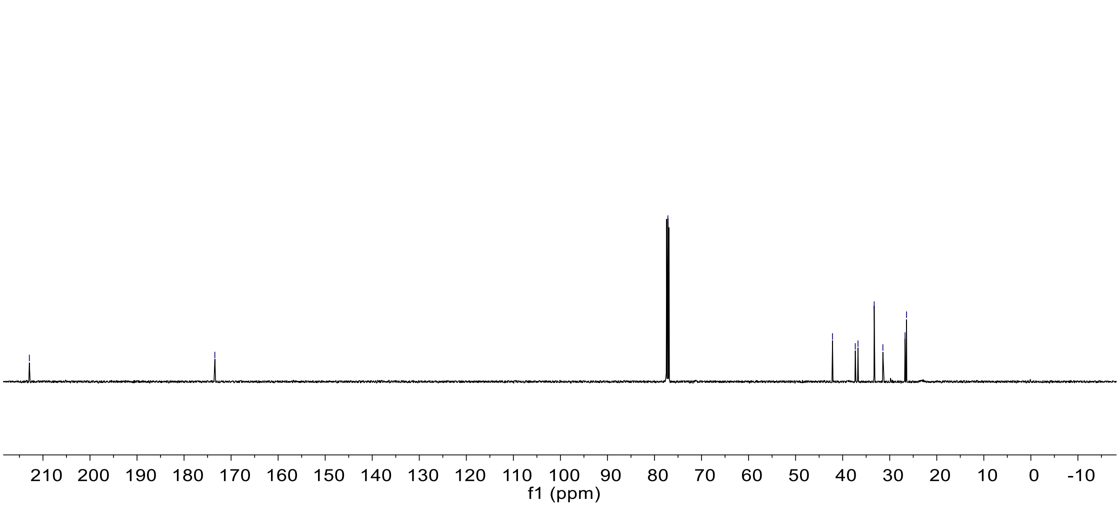
212.89
173.46
77.16 CDCl3
42.16
37.32
36.74
33.30
31.45
26.73
26.42
210 200 190 180 170 160 150 140 130 120 110 100 90 80 70 60 50 40 30 20 10 0 -10
f1 (ppm)

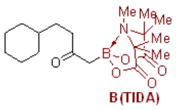

39

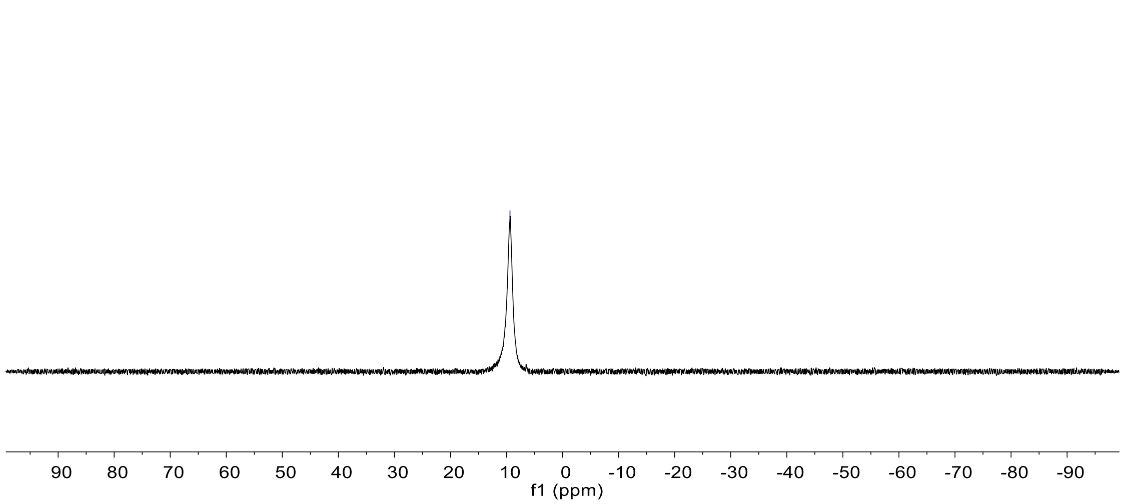

9.39
90 80 70 60 50 40 30 20 10 0 -10 -20 -30 -40 -50 -60 -70 -80 -90
f1 (ppm)

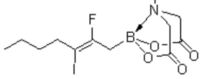

40

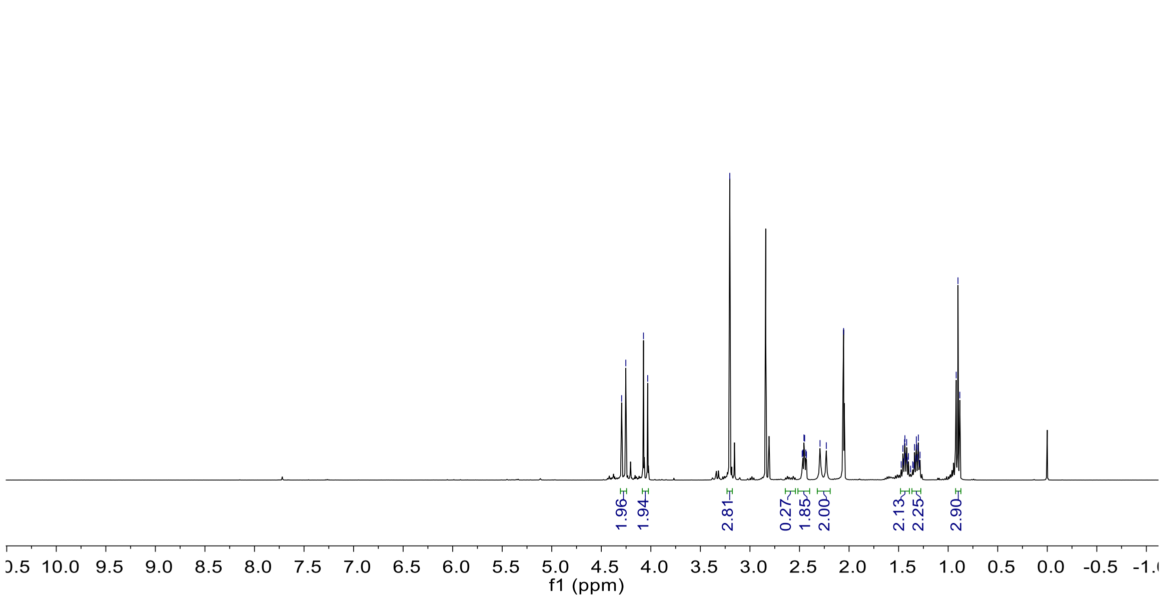

4.30 4.25 4.07 4.03 3.20 2.47 2.47 2.46 2.45 2.44 2.43 2.29 2.23 2.05 Acetone-d6 1.47 1.46 1.45 1.44 1.44 1.43 1.42 1.41 1.40 1.38 1.36 1.35 1.34 1.33 1.32 1.32 1.31 1.30 1.30 1.29 1.28 0.92 0.90 0.88
1.96 1.94 2.81 0.27 1.85 2.00 2.13 2.25 2.90
0.5 10.0 9.5 9.0 8.5 8.0 7.5 7.0 6.5 6.0 5.5 5.0 4.5 4.0 3.5 3.0 2.5 2.0 1.5 1.0 0.5 0.0 -0.5 -1.0
f1 (ppm)

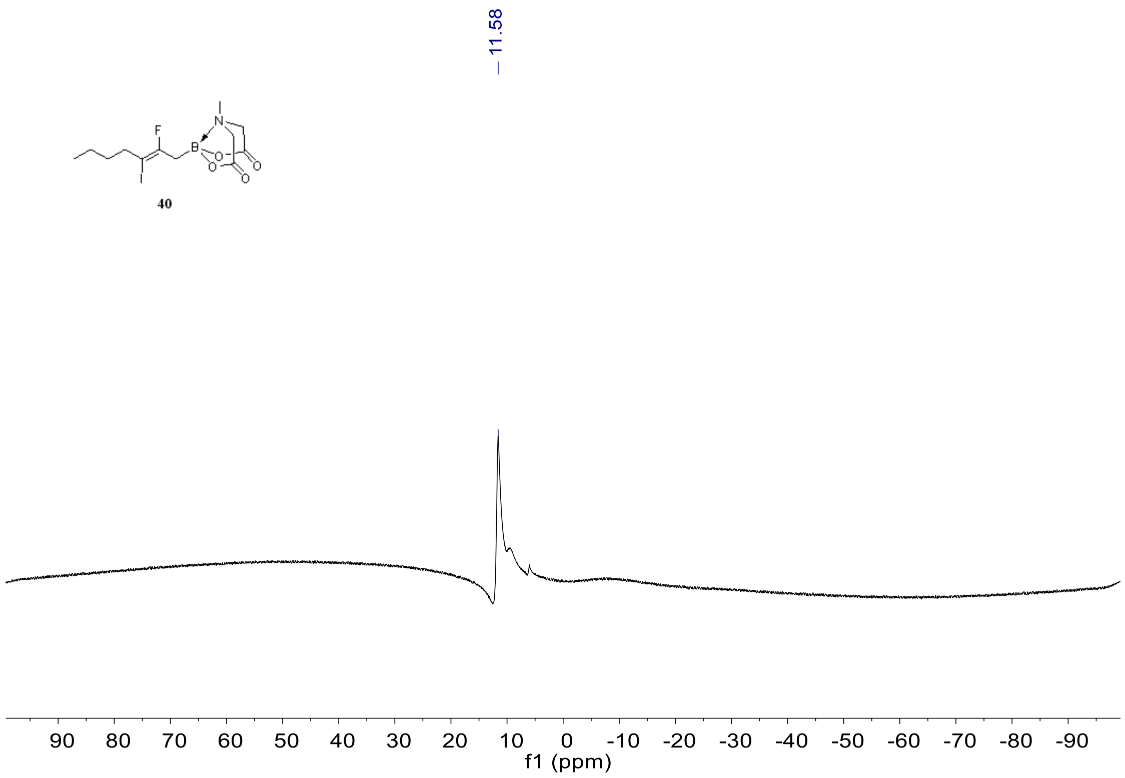
11.58
F
N
B
O
O
O
O
I
40
90 80 70 60 50 40 30 20 10 0 -10 -20 -30 -40 -50 -60 -70 -80 -90
f1 (ppm)

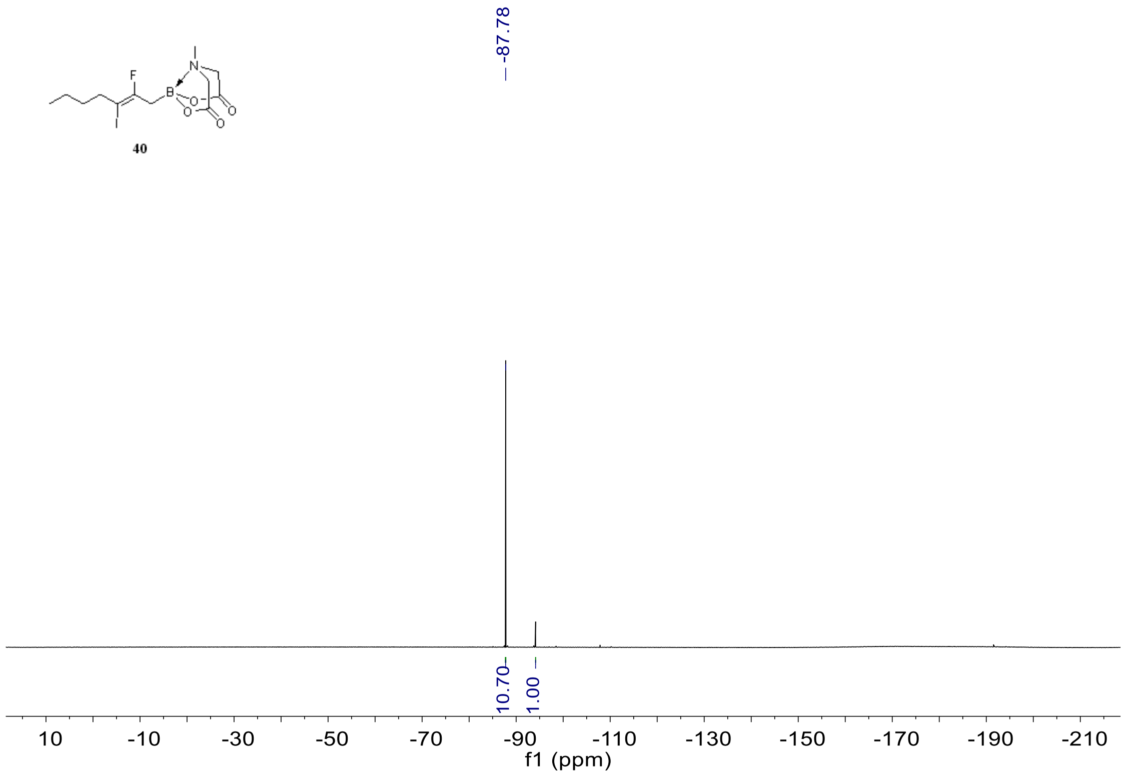
-87.78
F
N
B
O
O
O
O
I
40
10.70
1.00
10 -10 -30 -50 -70 -90 -110 -130 -150 -170 -190 -210
f1 (ppm)

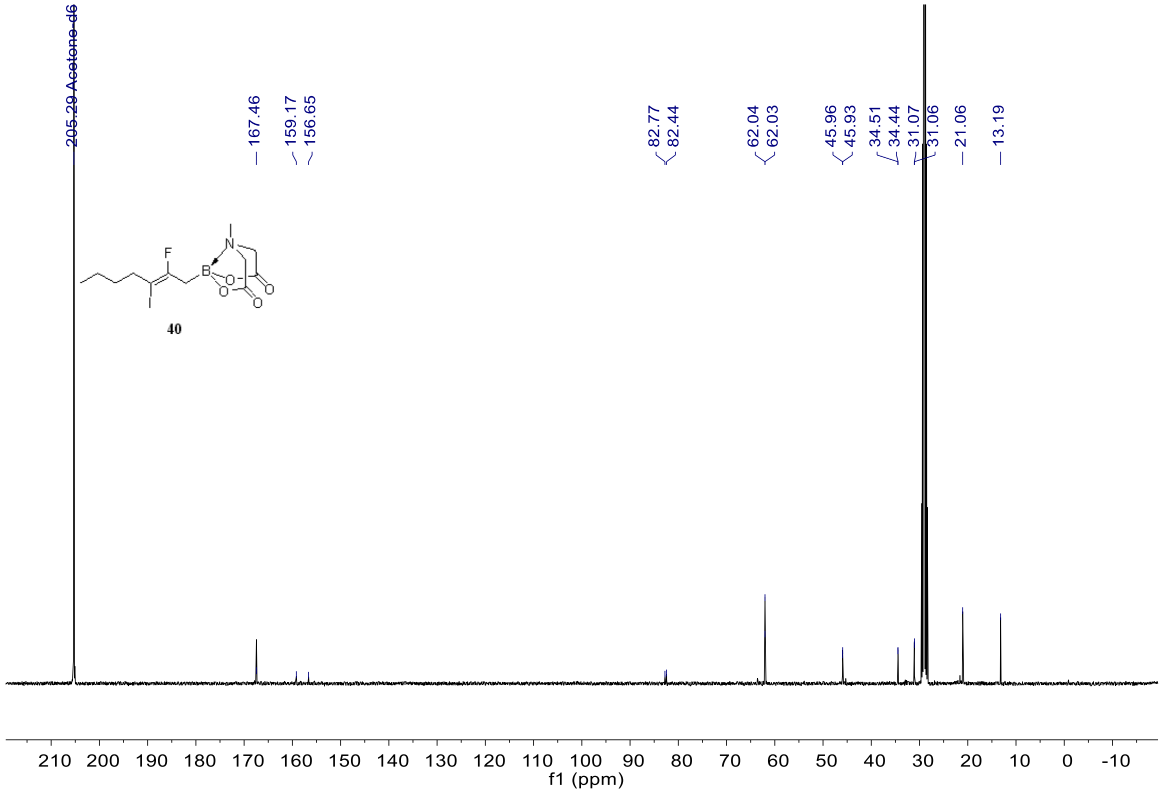

205.29 Acetone-d6
167.46
159.17
156.65
82.77
82.44
62.04
62.03
45.96
45.93
34.51
34.44
31.07
31.06
21.06
13.19
40
210 200 190 180 170 160 150 140 130 120 110 100 90 80 70 60 50 40 30 20 10 0 -10
f1 (ppm)

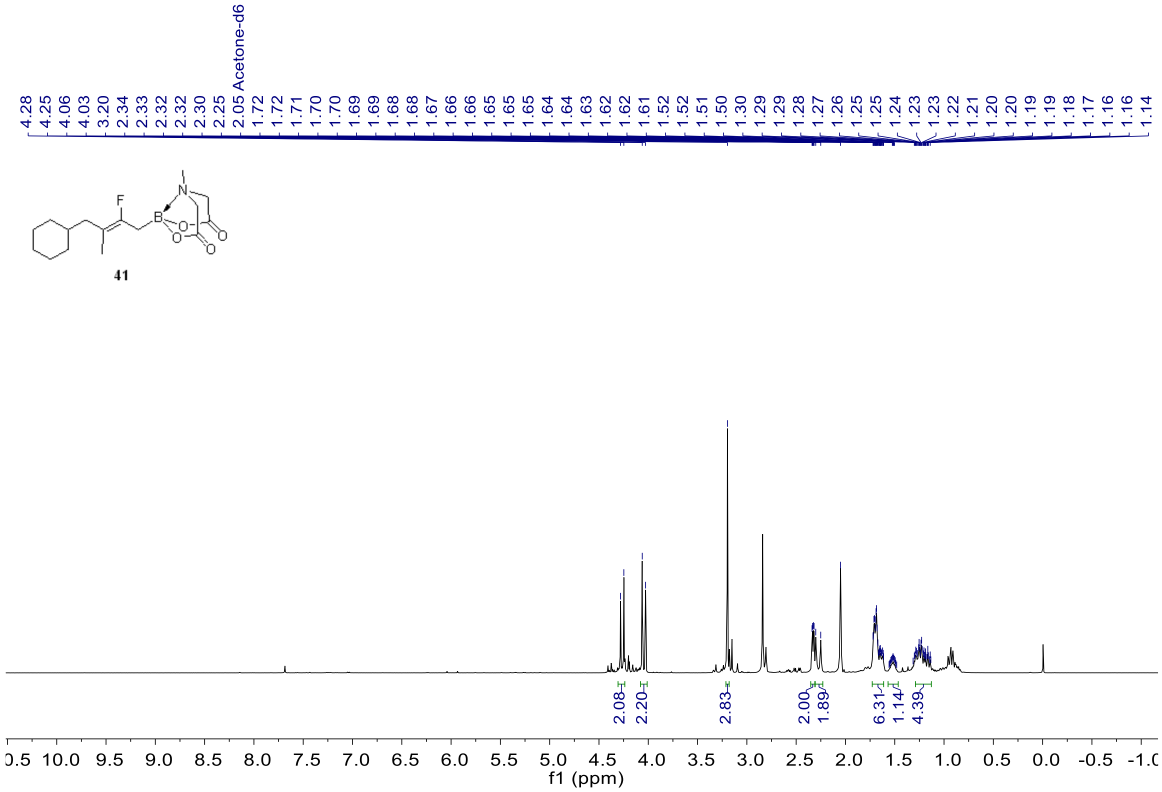

4.28 4.25 4.06 4.03 3.20 2.34 2.33 2.32 2.32 2.30 2.25 2.05 Acetone-d6 1.72 1.72 1.71 1.70 1.69 1.69 1.68 1.68 1.67 1.66 1.66 1.65 1.65 1.65 1.64 1.64 1.63 1.62 1.62 1.61 1.52 1.52 1.51 1.50 1.30 1.29 1.29 1.28 1.27 1.26 1.25 1.25 1.24 1.23 1.23 1.22 1.21 1.20 1.20 1.19 1.19 1.18 1.17 1.16 1.14
41
2.08 2.20 2.83 2.00 1.89 6.31 1.14 4.39
0.5 10.0 9.5 9.0 8.5 8.0 7.5 7.0 6.5 6.0 5.5 5.0 4.5 4.0 3.5 3.0 2.5 2.0 1.5 1.0 0.5 0.0 -0.5 -1.0
f1 (ppm)

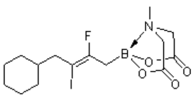

41

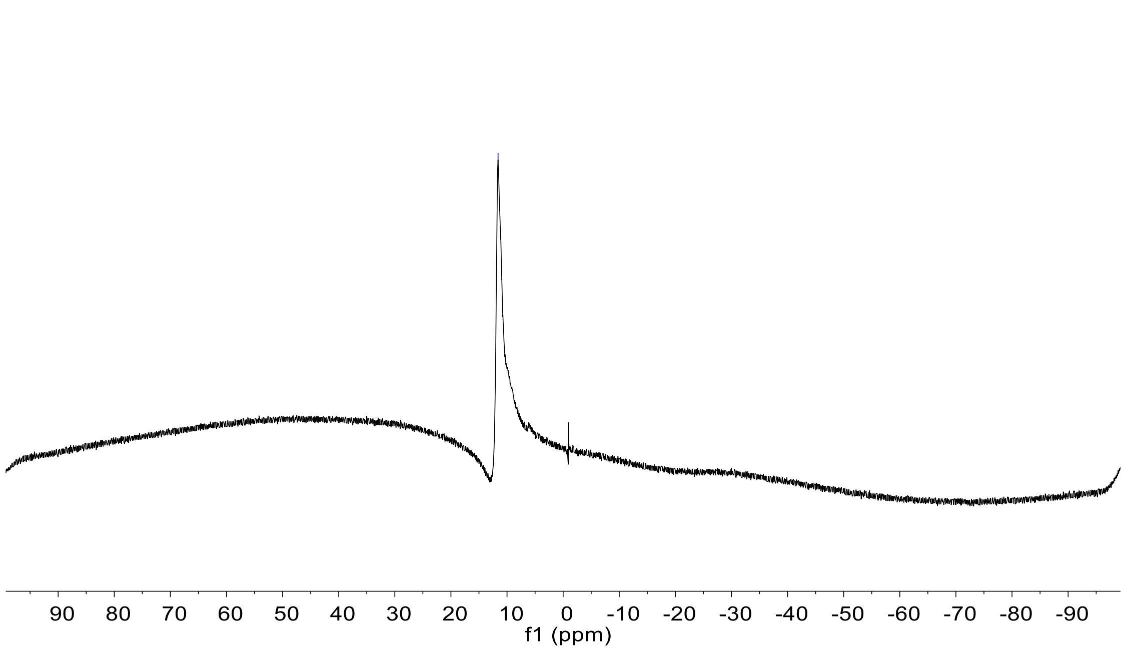
11.60
90 80 70 60 50 40 30 20 10 0 -10 -20 -30 -40 -50 -60 -70 -80 -90
f1 (ppm)

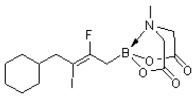

41

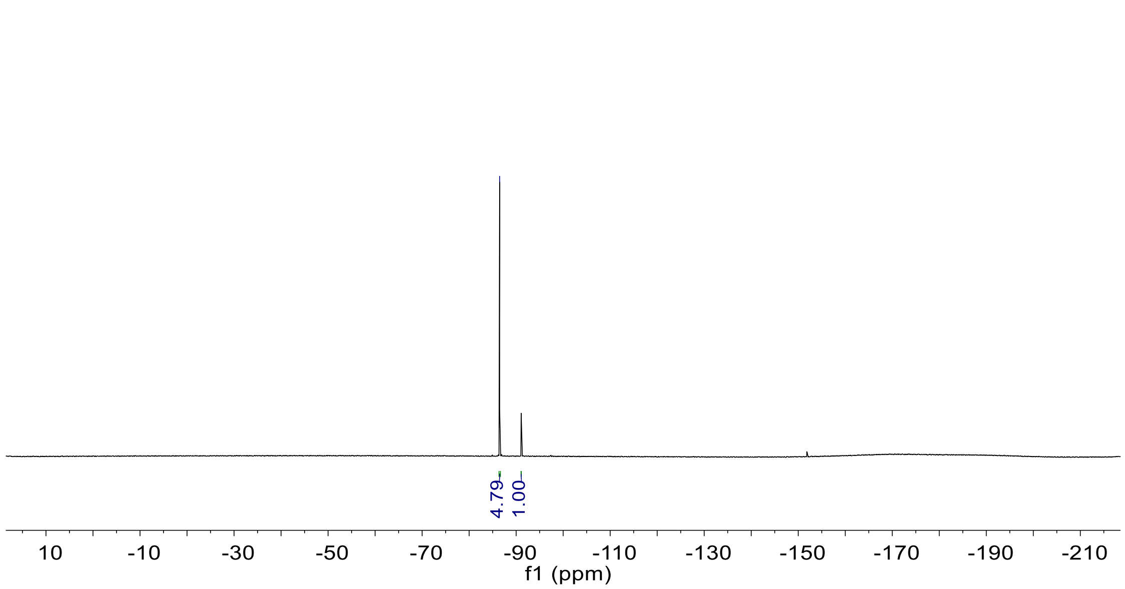
-86.49
4.79
1.00
10 -10 -30 -50 -70 -90 -110 -130 -150 -170 -190 -210
f1 (ppm)

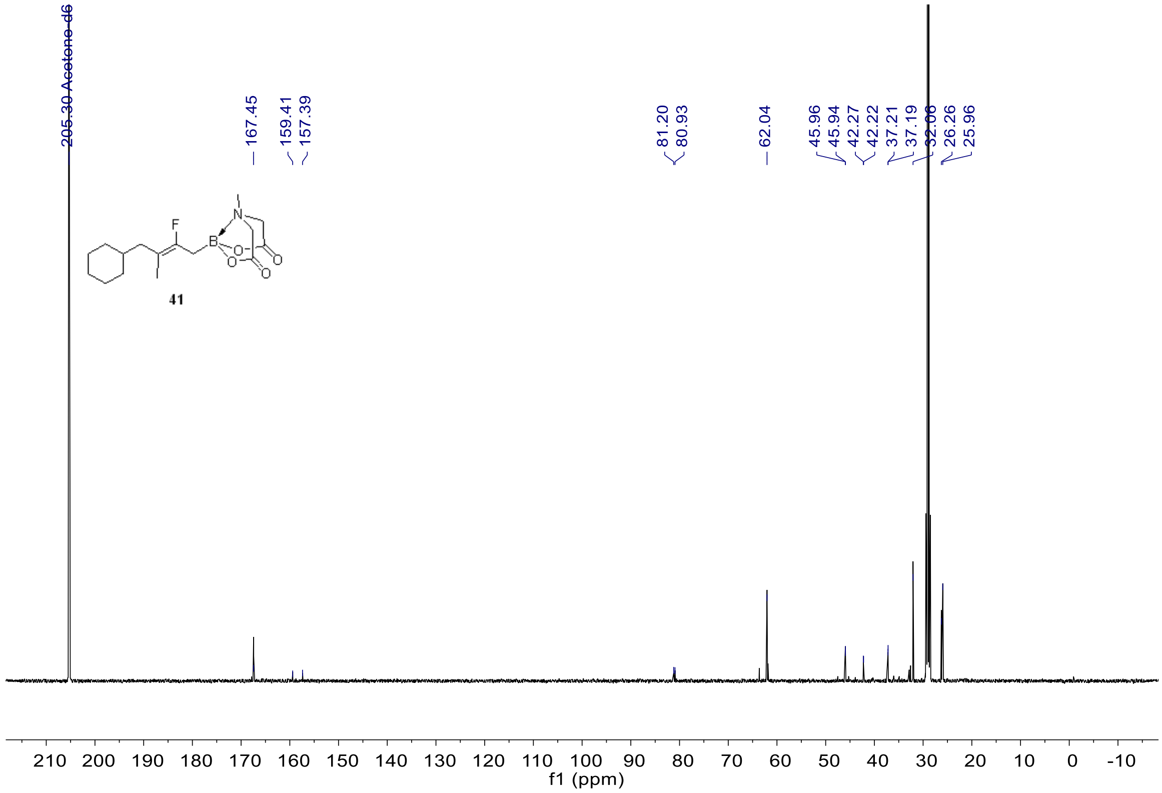
205.30 Acetone-d6
167.45
159.41
157.39
81.20
80.93
62.04
45.96
45.94
42.27
42.22
37.21
37.19
32.06
26.26
25.96
F
N
B
O
O
O
O
41
210 200 190 180 170 160 150 140 130 120 110 100 90 80 70 60 50 40 30 20 10 0 -10
f1 (ppm)

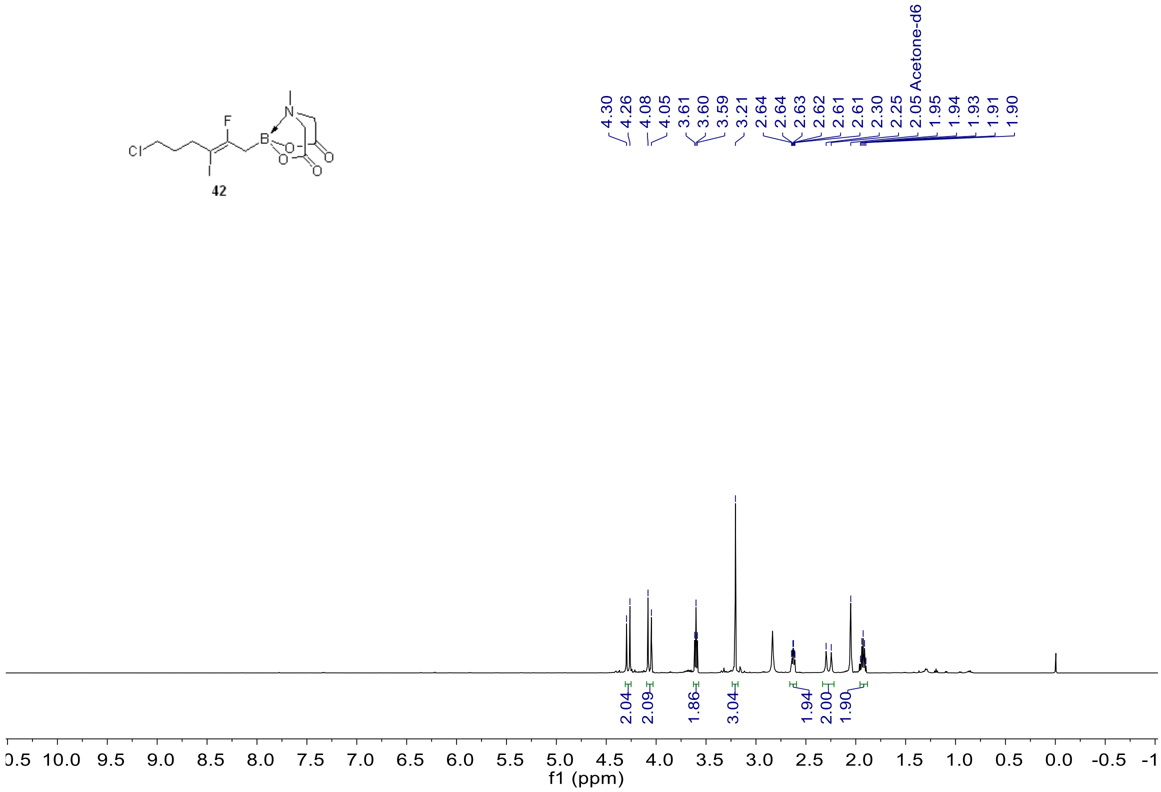
4.30
4.26
4.08
4.05
3.61
3.60
3.59
3.21
2.64
2.64
2.63
2.62
2.61
2.61
2.30
2.25
2.05 Acetone-d6
1.95
1.94
1.93
1.91
1.90
Cl
F
N
B
O
O
O
O
I
42
2.04
2.09
1.86
3.04
1.94
2.00
1.90
0.5 10.0 9.5 9.0 8.5 8.0 7.5 7.0 6.5 6.0 5.5 5.0 4.5 4.0 3.5 3.0 2.5 2.0 1.5 1.0 0.5 0.0 -0.5 -1
f1 (ppm)

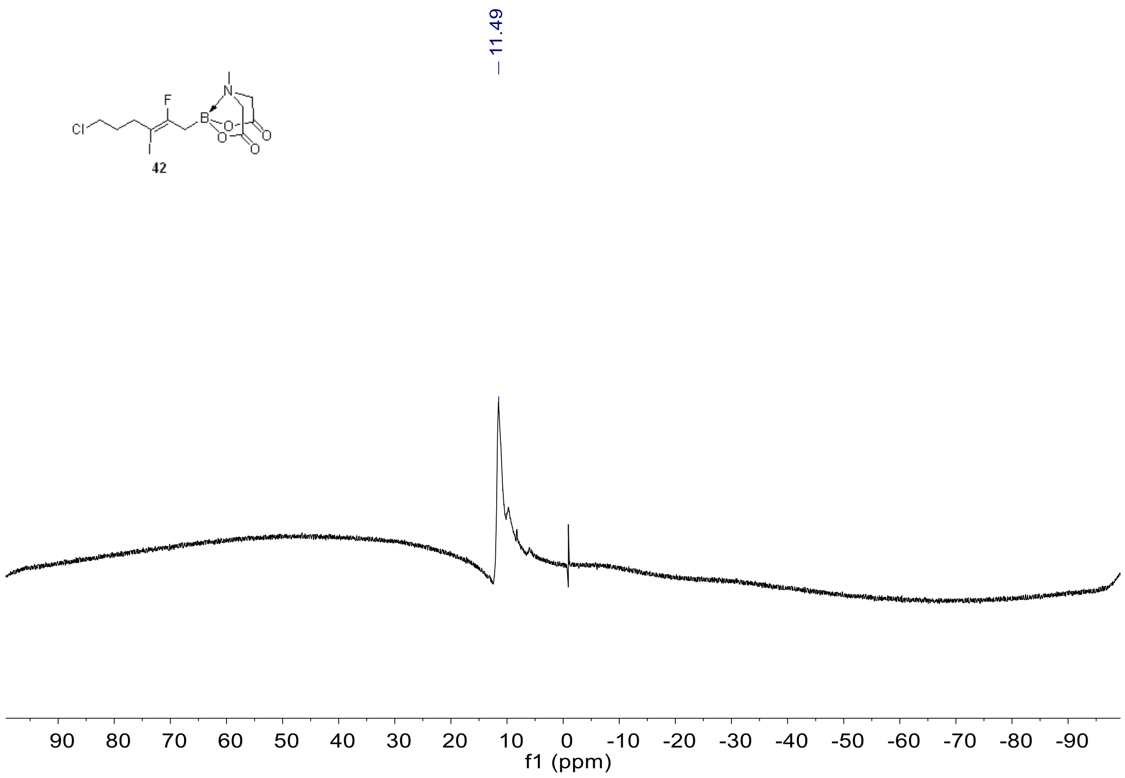

11.49
Cl
F
N
B
O
O
O
O
I
42
90
80
70
60
50
40
30
20
10
0
-10
-20
-30
-40
-50
-60
-70
-80
-90
f1 (ppm)

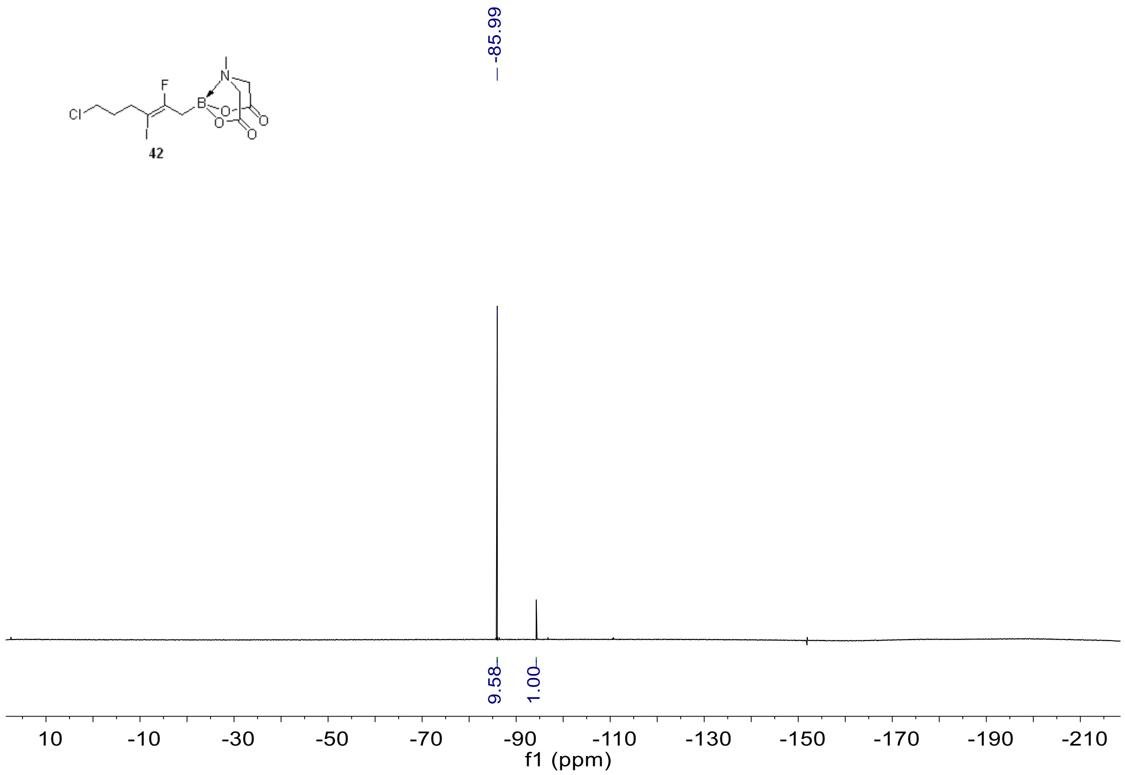

-85.99
Cl
F
N
B
O
O
O
O
I
42
9.58
1.00
10
-10
-30
-50
-70
-90
-110
-130
-150
-170
-190
-210
f1 (ppm)

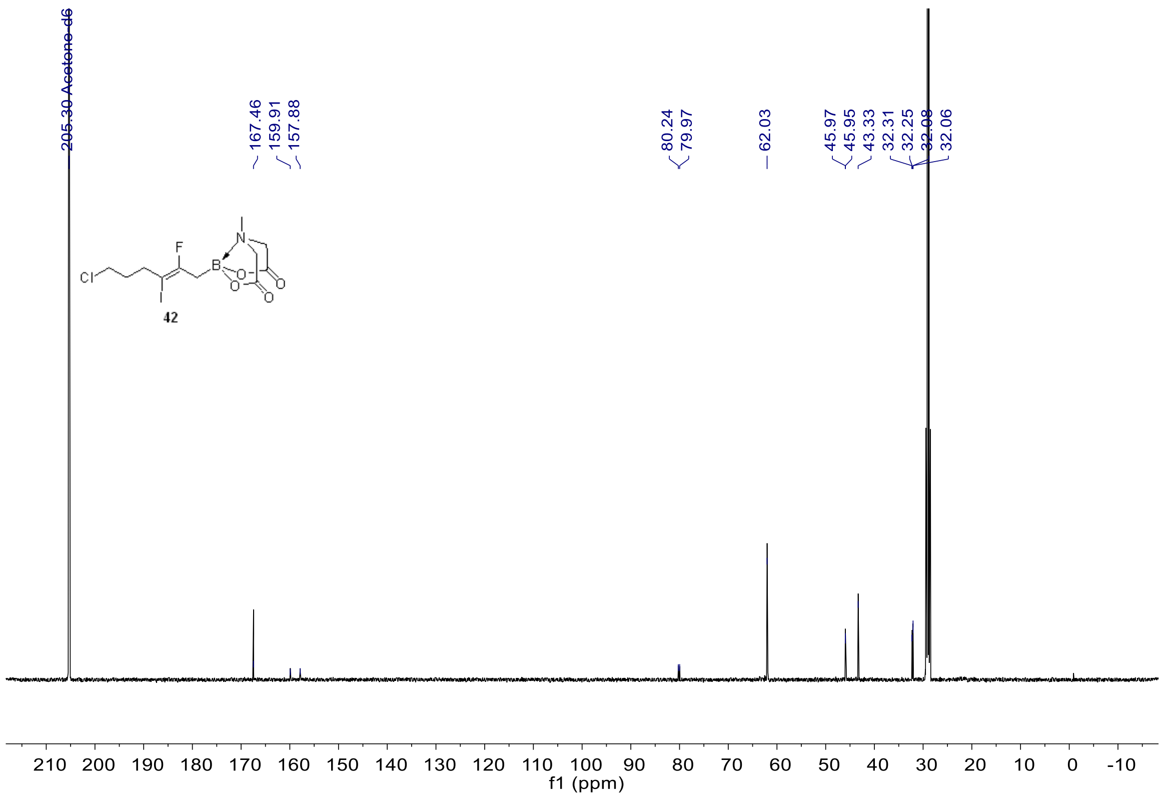
205.30 Acetone-d6
167.46
159.91
157.88
80.24
79.97
62.03
45.97
45.95
43.33
32.31
32.25
32.08
32.06
Cl
F
I
N
B
O
42
210 200 190 180 170 160 150 140 130 120 110 100 90 80 70 60 50 40 30 20 10 0 -10
f1 (ppm)

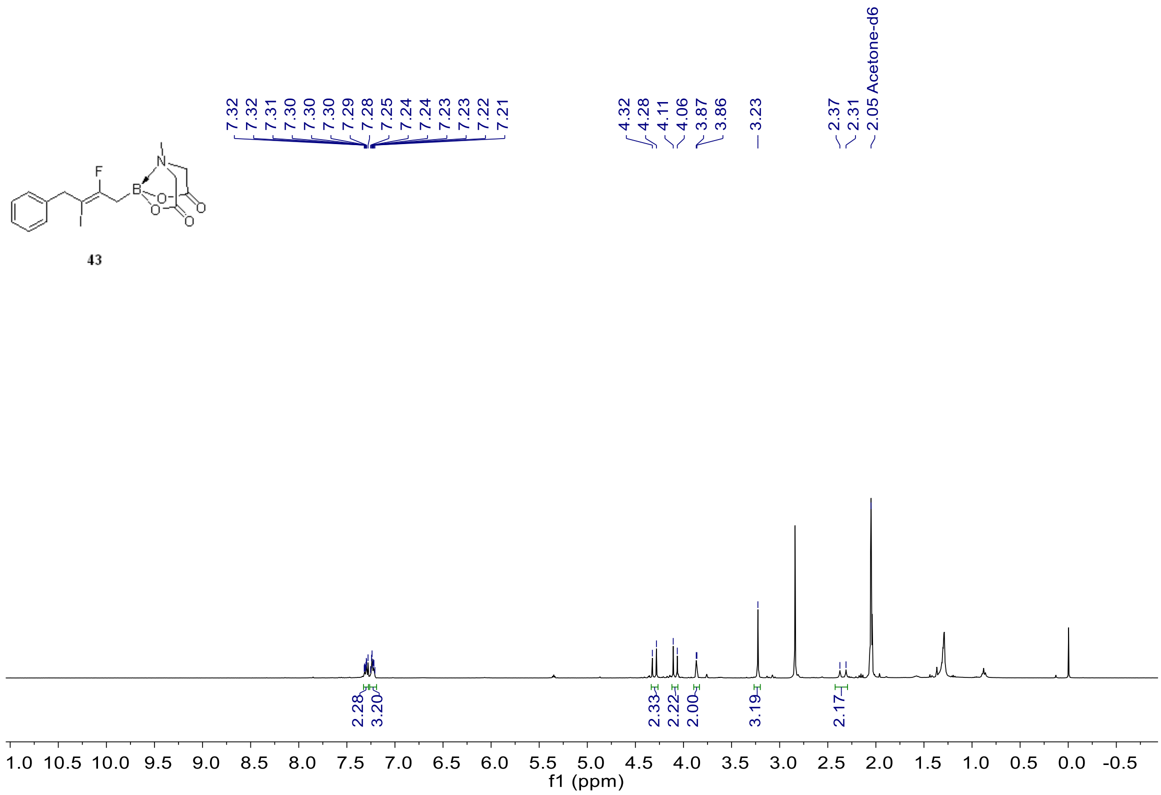
7.32
7.32
7.31
7.30
7.30
7.30
7.29
7.28
7.25
7.24
7.24
7.23
7.23
7.22
7.21
4.32
4.28
4.11
4.06
3.87
3.86
3.23
2.37
2.31
2.05 Acetone-d6
F
I
N
B
O
43
2.28
3.20
2.33
2.22
2.00
3.19
2.17
1.0 10.5 10.0 9.5 9.0 8.5 8.0 7.5 7.0 6.5 6.0 5.5 5.0 4.5 4.0 3.5 3.0 2.5 2.0 1.5 1.0 0.5 0.0 -0.5
f1 (ppm)

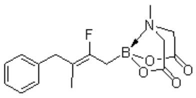

43

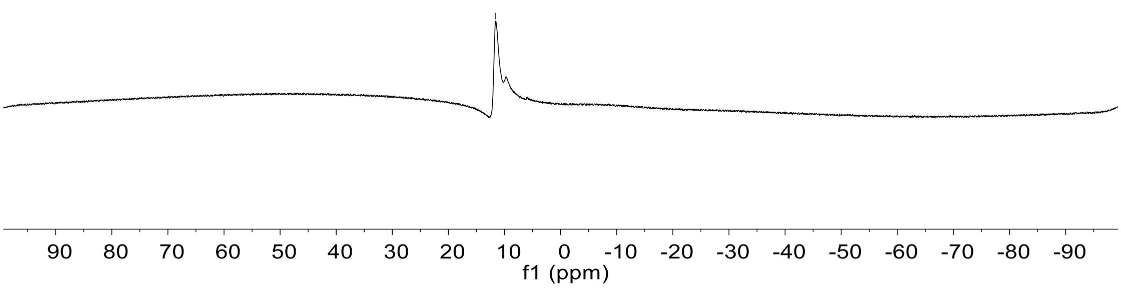

11.57
90 80 70 60 50 40 30 20 10 0 -10 -20 -30 -40 -50 -60 -70 -80 -90
f1 (ppm)

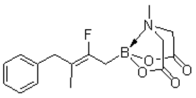

43

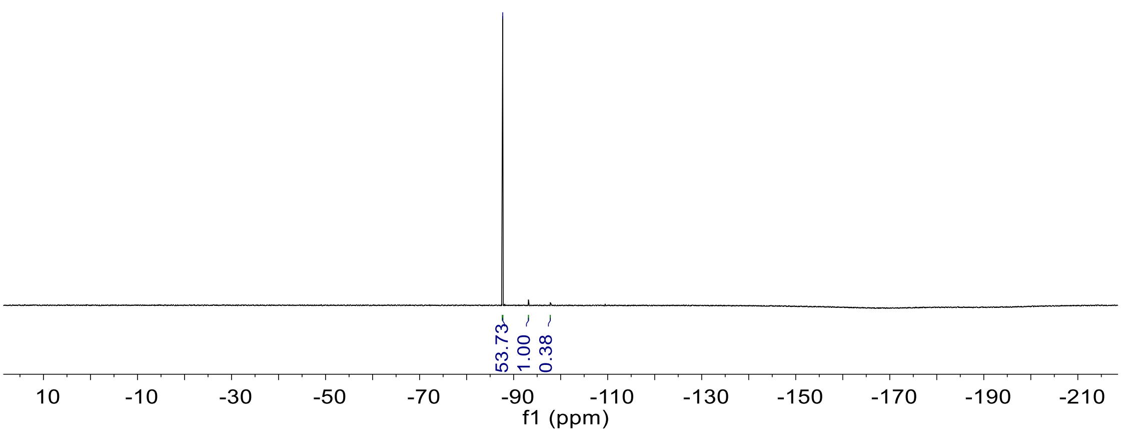

-87.68
53.73
1.00
0.38
10 -10 -30 -50 -70 -90 -110 -130 -150 -170 -190 -210
f1 (ppm)

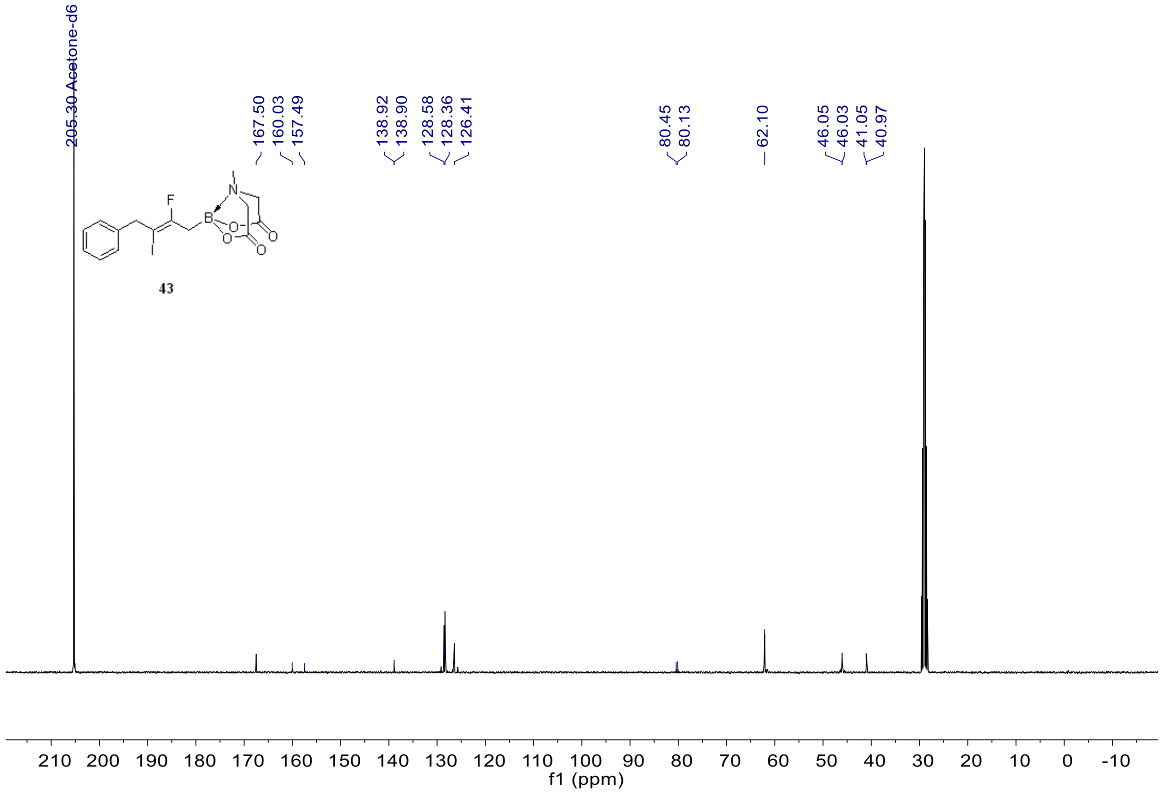
205.30 Acetone-d6
167.50
160.03
157.49
138.92
138.90
128.58
128.36
126.41
80.45
80.13
62.10
46.05
46.03
41.05
40.97
F
N
B
O
I
43
210 200 190 180 170 160 150 140 130 120 110 100 90 80 70 60 50 40 30 20 10 0 -10
f1 (ppm)

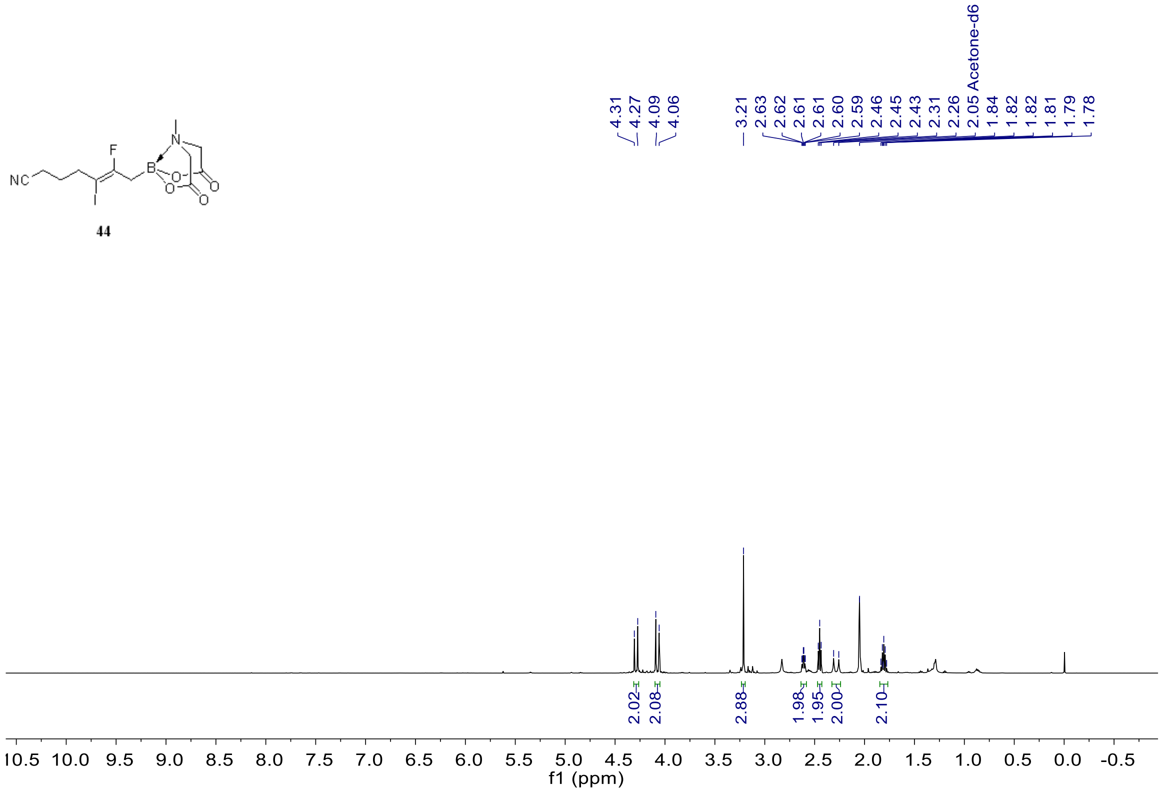
4.31
4.27
4.09
4.06
3.21
2.63
2.62
2.61
2.61
2.60
2.59
2.46
2.45
2.43
2.31
2.26
2.05 Acetone-d6
1.84
1.82
1.82
1.81
1.79
1.78
NC
F
N
B
O
I
44
2.02
2.08
2.88
1.98
1.95
2.00
2.10
10.5 10.0 9.5 9.0 8.5 8.0 7.5 7.0 6.5 6.0 5.5 5.0 4.5 4.0 3.5 3.0 2.5 2.0 1.5 1.0 0.5 0.0 -0.5
f1 (ppm)

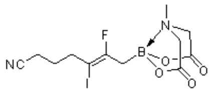

44

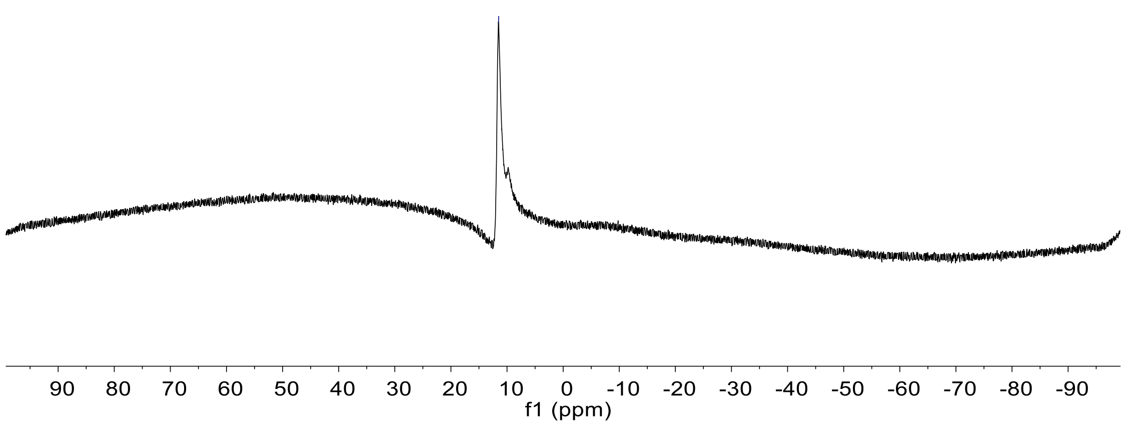
11.50
90 80 70 60 50 40 30 20 10 0 -10 -20 -30 -40 -50 -60 -70 -80 -90
f1 (ppm)

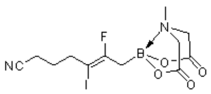

44

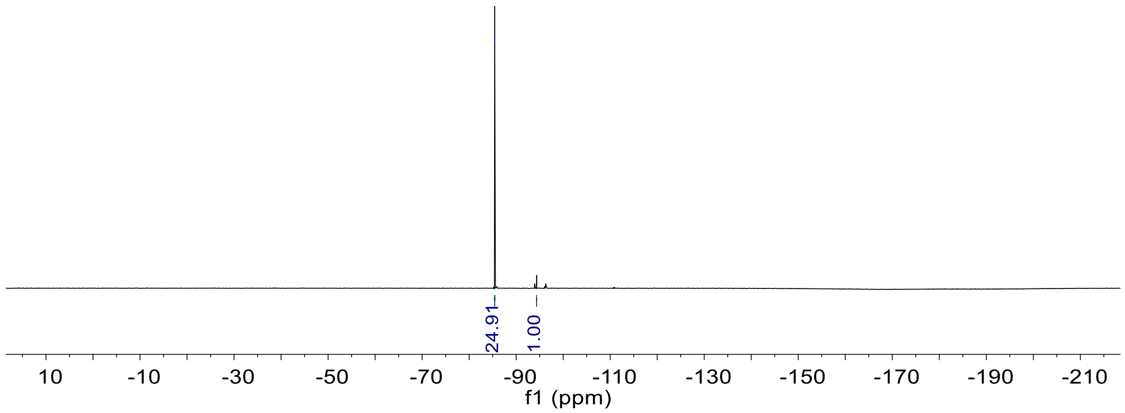
-85.44
24.91
1.00
10 -10 -30 -50 -70 -90 -110 -130 -150 -170 -190 -210
f1 (ppm)

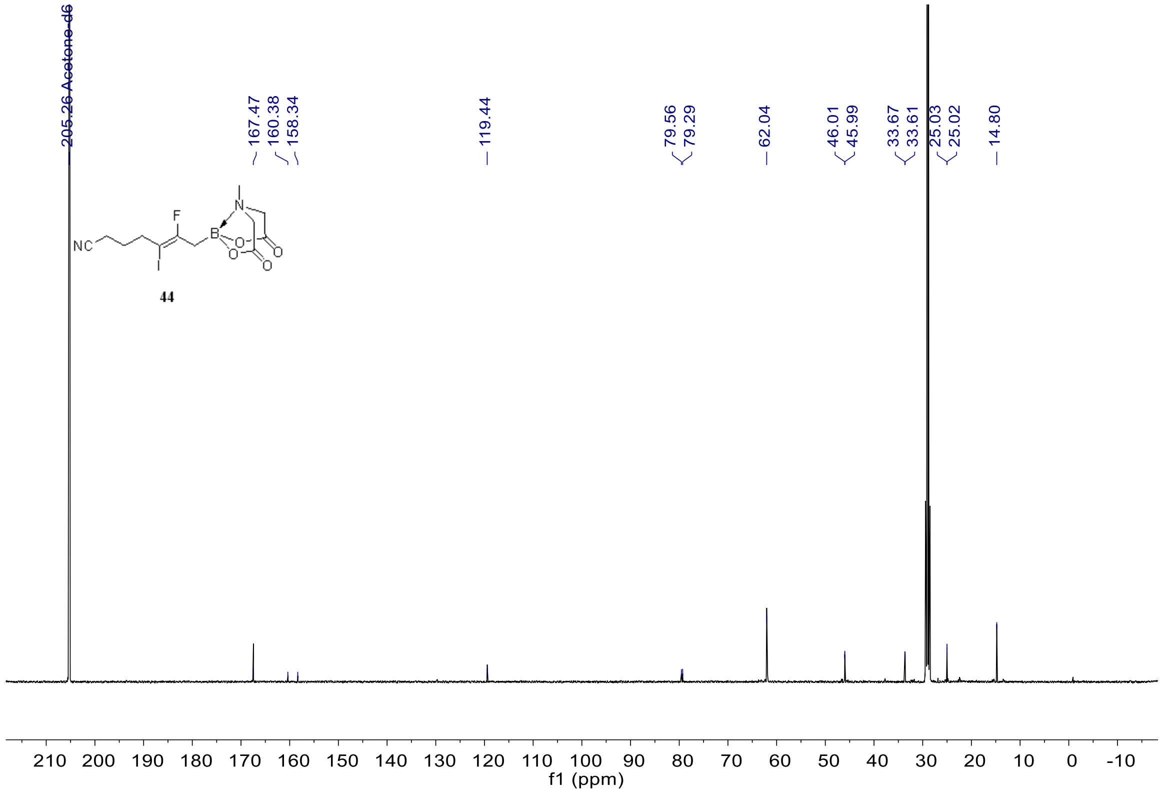
205.26 Acetone-d6
167.47
160.38
158.34
119.44
79.56
79.29
62.04
46.01
45.99
33.67
33.61
25.03
25.02
14.80
NC
F
B
N
O
I
44
210 200 190 180 170 160 150 140 130 120 110 100 90 80 70 60 50 40 30 20 10 0 -10
f1 (ppm)

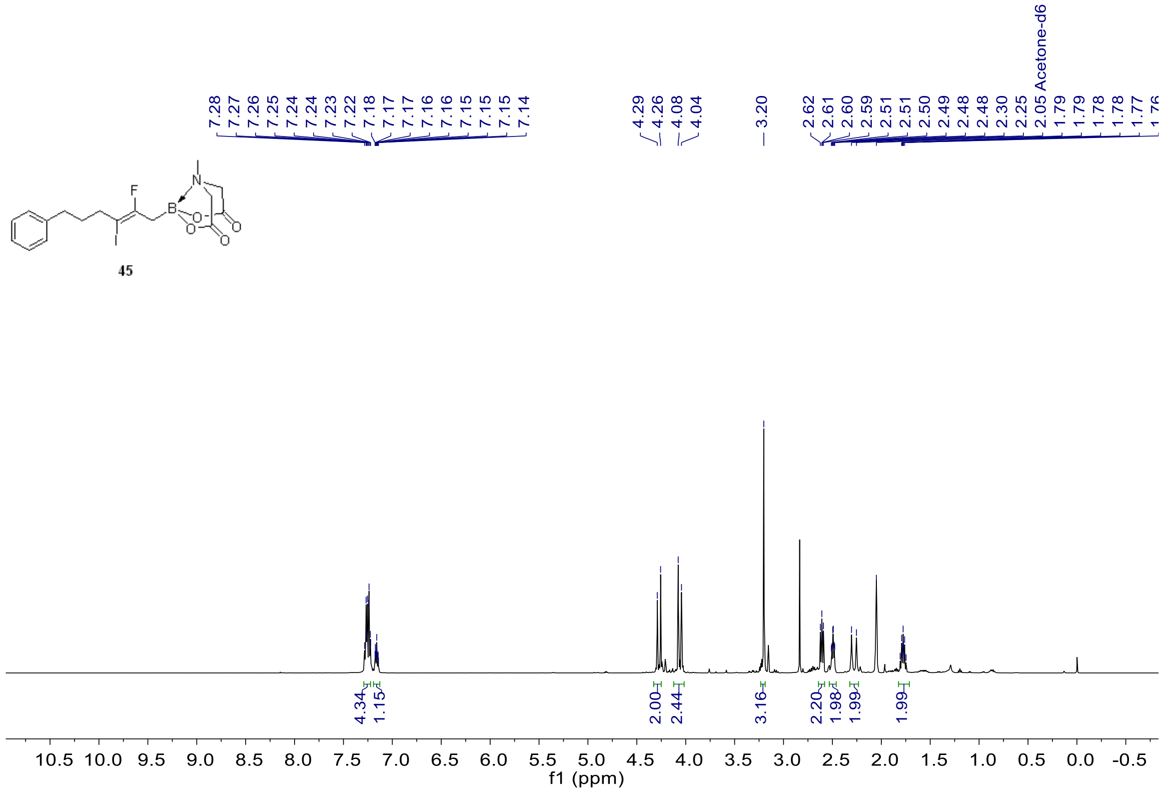
7.28
7.27
7.26
7.25
7.24
7.24
7.23
7.22
7.18
7.17
7.17
7.16
7.16
7.15
7.15
7.15
7.14
4.29
4.26
4.08
4.04
3.20
2.62
2.61
2.60
2.59
2.51
2.51
2.50
2.49
2.48
2.48
2.30
2.25
2.05 Acetone-d6
1.79
1.79
1.78
1.78
1.77
F
B
N
O
I
45
4.34
1.15
2.00
2.44
3.16
2.20
1.98
1.99
1.99
10.5 10.0 9.5 9.0 8.5 8.0 7.5 7.0 6.5 6.0 5.5 5.0 4.5 4.0 3.5 3.0 2.5 2.0 1.5 1.0 0.5 0.0 -0.5
f1 (ppm)

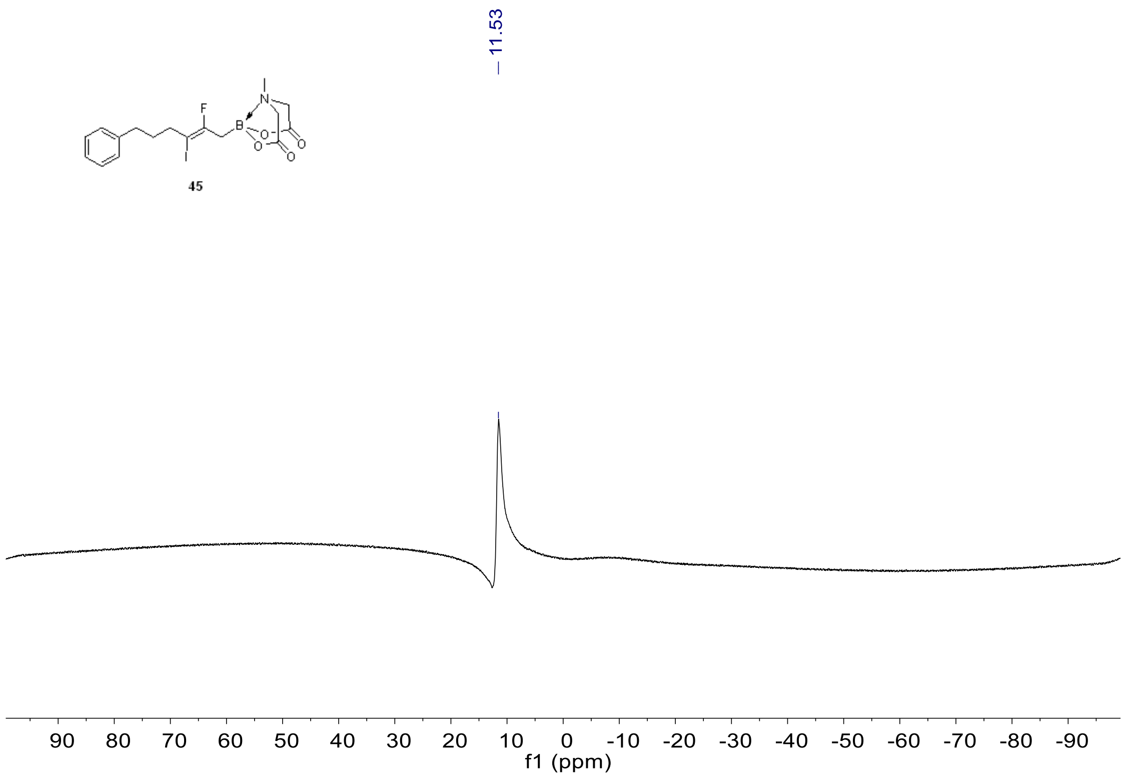
11.53
F
N
B
O
O
O
O
I
90 80 70 60 50 40 30 20 10 0 -10 -20 -30 -40 -50 -60 -70 -80 -90
f1 (ppm)

45

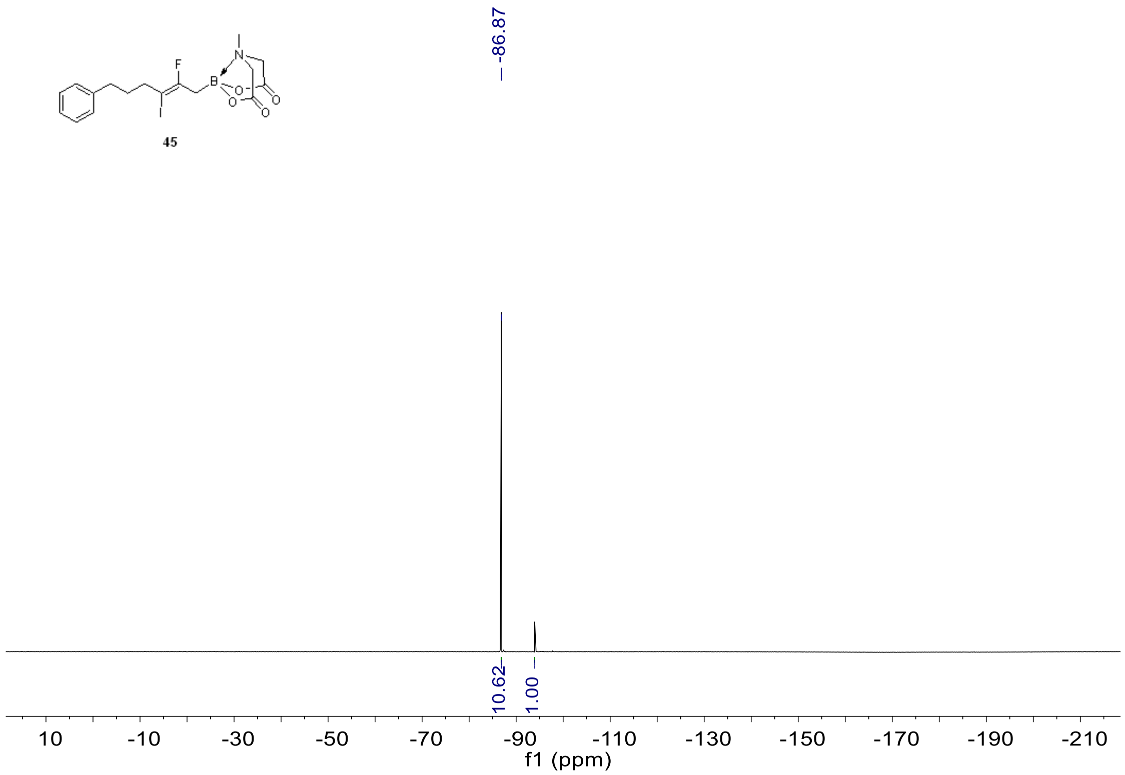
-86.87
F
N
B
O
O
O
O
I
10.62
1.00
10 -10 -30 -50 -70 -90 -110 -130 -150 -170 -190 -210
f1 (ppm)

45

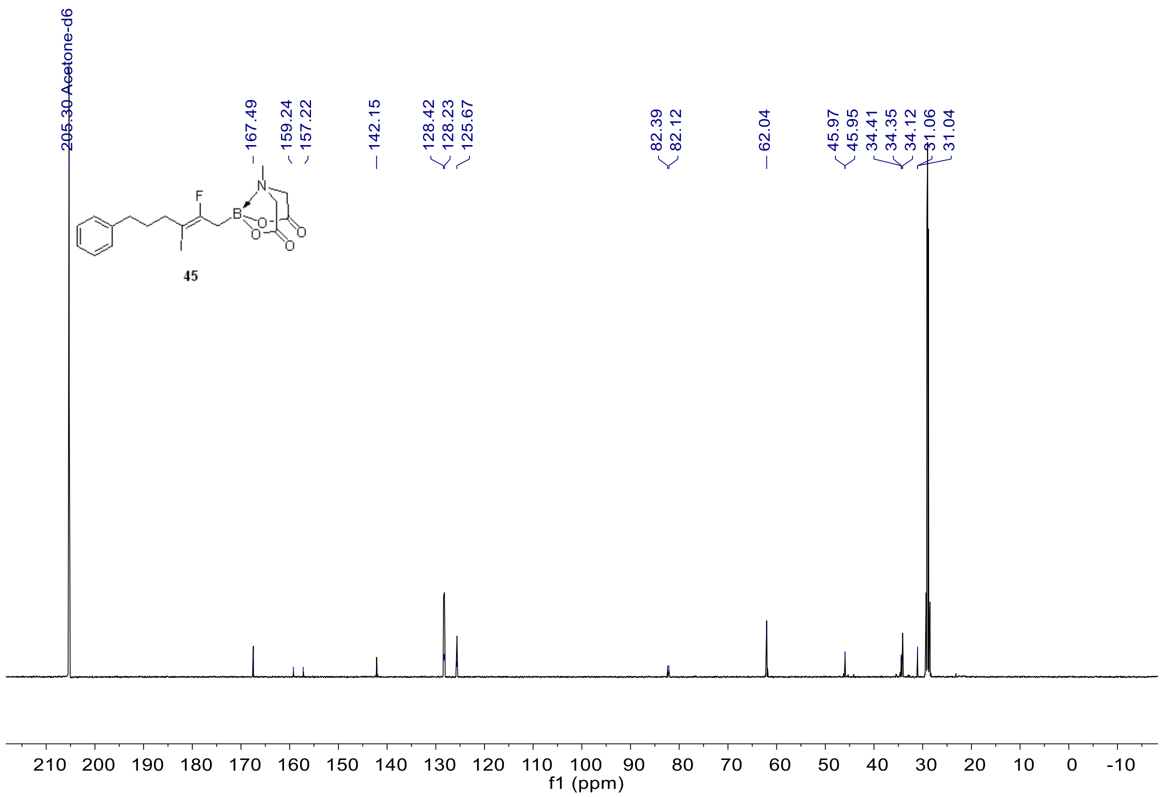
205.30 Acetone-d6
167.49
159.24
157.22
142.15
128.42
128.23
125.67
82.39
82.12
62.04
45.97
45.95
34.41
34.35
34.12
31.06
31.04
F
N
B
O
I
45
210 200 190 180 170 160 150 140 130 120 110 100 90 80 70 60 50 40 30 20 10 0 -10
f1 (ppm)

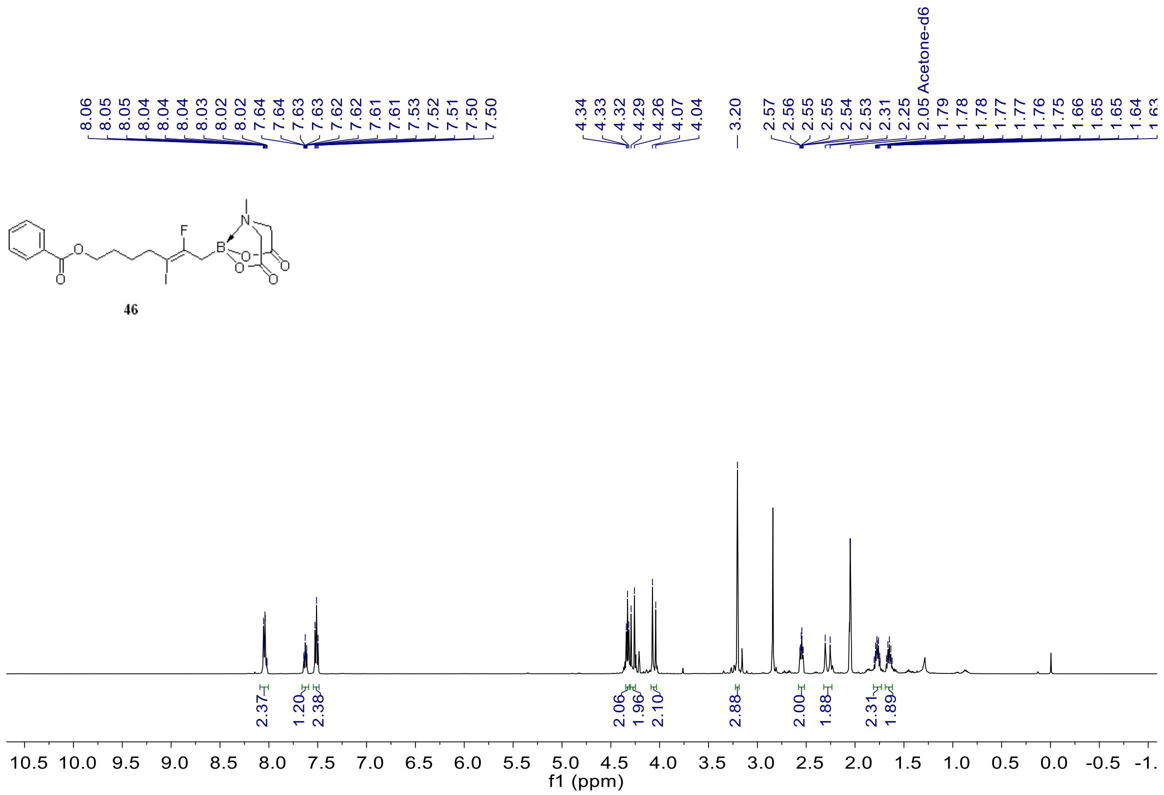
8.06
8.05
8.05
8.04
8.04
8.03
8.02
8.02
7.64
7.64
7.63
7.63
7.62
7.62
7.61
7.61
7.53
7.52
7.51
7.50
7.50
4.34
4.33
4.32
4.29
4.26
4.07
4.04
3.20
2.57
2.56
2.55
2.55
2.54
2.53
2.31
2.25
2.05 Acetone-d6
1.79
1.78
1.78
1.77
1.77
1.76
1.75
1.66
1.65
1.65
1.64
F
N
B
O
I
46
2.37
1.20
2.38
2.06
1.96
2.10
2.88
2.00
1.88
2.31
1.89
10.5 10.0 9.5 9.0 8.5 8.0 7.5 7.0 6.5 6.0 5.5 5.0 4.5 4.0 3.5 3.0 2.5 2.0 1.5 1.0 0.5 0.0 -0.5
f1 (ppm)

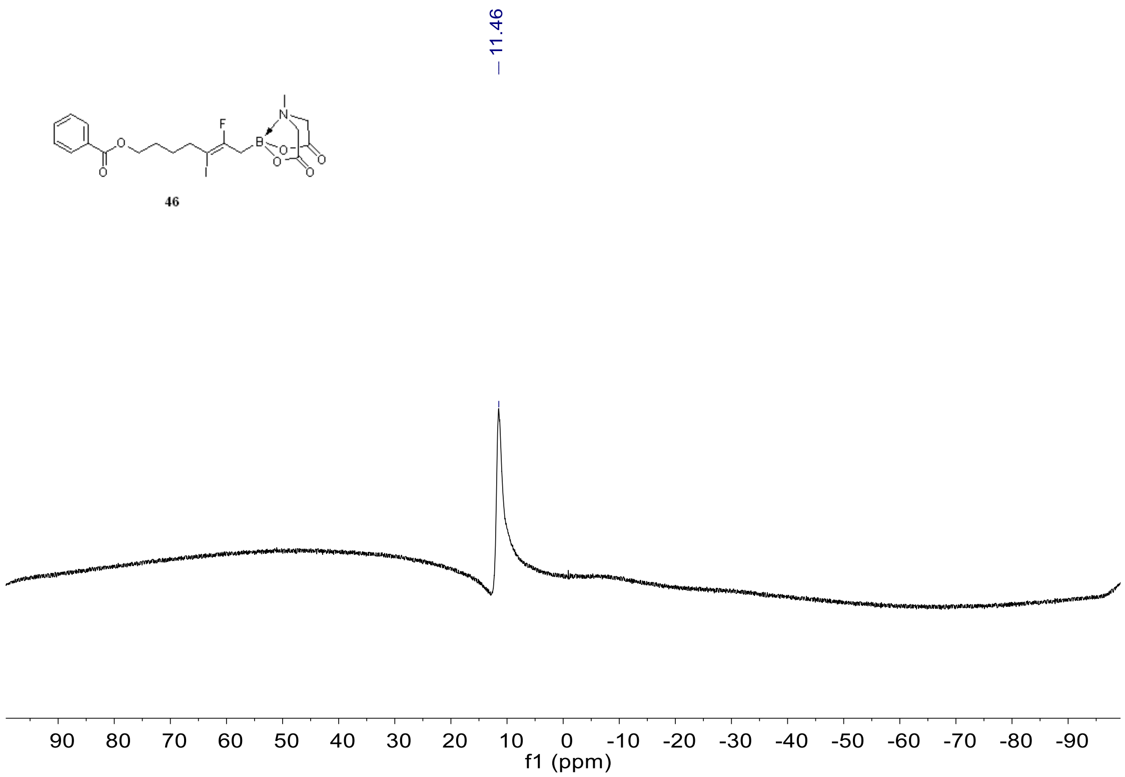

11.46
46
90 80 70 60 50 40 30 20 10 0 -10 -20 -30 -40 -50 -60 -70 -80 -90
f1 (ppm)

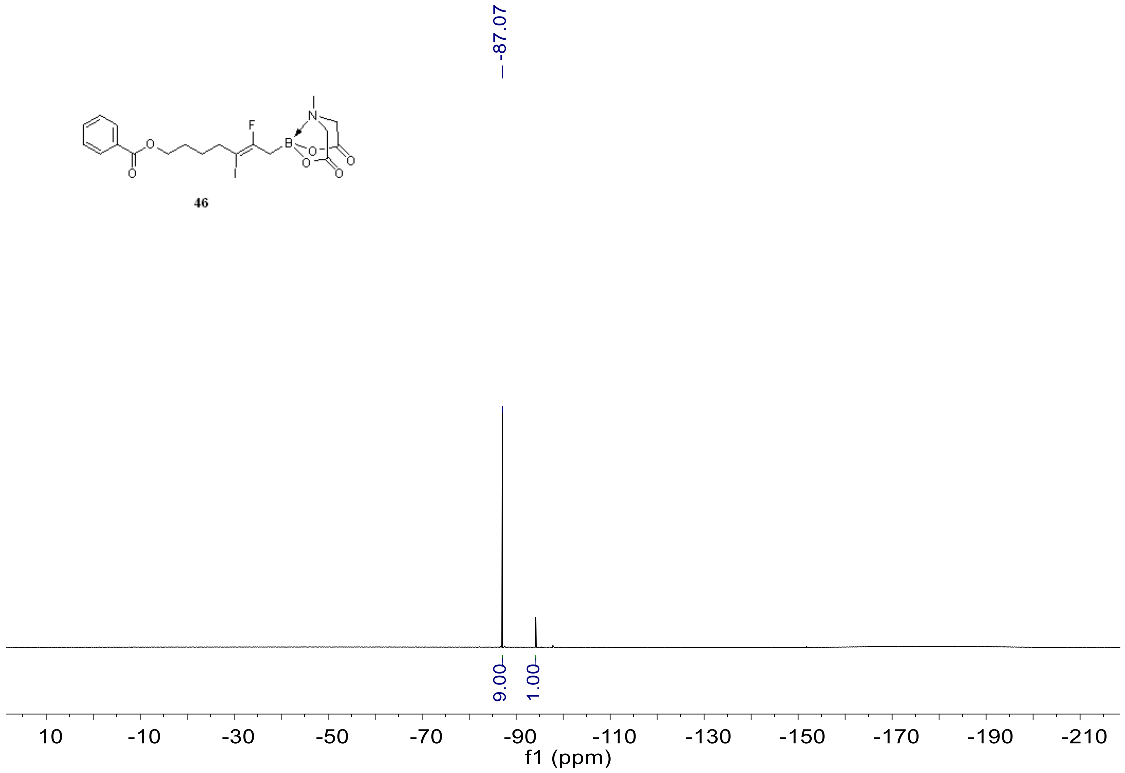

-87.07
46
9.00
1.00
10 -10 -30 -50 -70 -90 -110 -130 -150 -170 -190 -210
f1 (ppm)

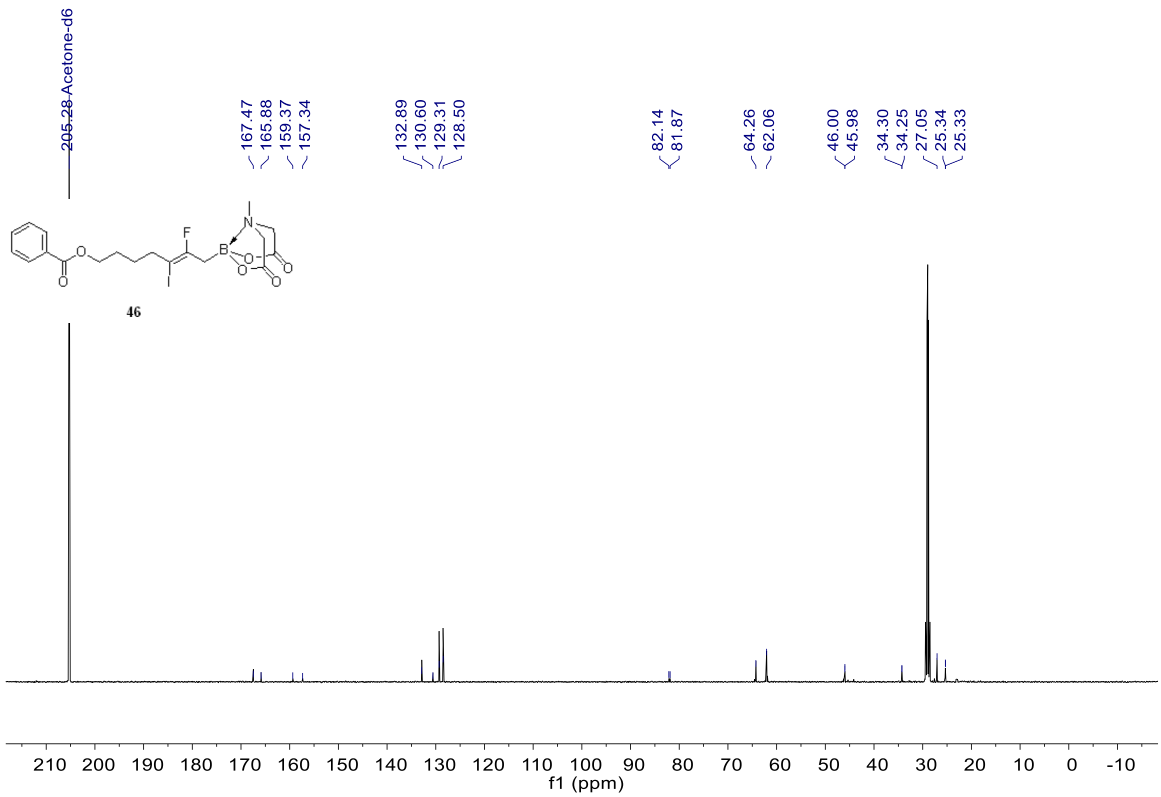
205.28 Acetone-d6
167.47
165.88
159.37
157.34
132.89
130.60
129.31
128.50
82.14
81.87
64.26
62.06
46.00
45.98
34.30
34.25
27.05
25.34
25.33
F
B
N
O
I
46
210 200 190 180 170 160 150 140 130 120 110 100 90 80 70 60 50 40 30 20 10 0 -10
f1 (ppm)

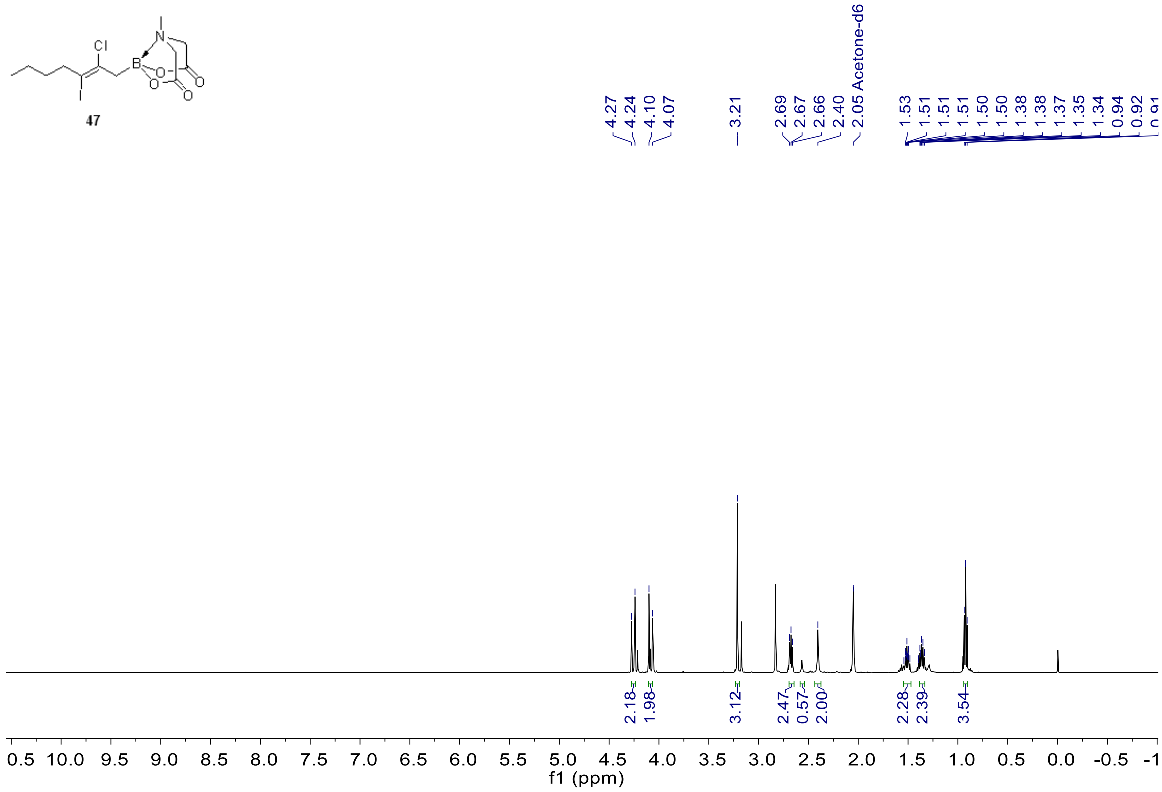
Cl
B
N
O
I
47
4.27
4.24
4.10
4.07
3.21
2.69
2.67
2.66
2.40
2.05 Acetone-d6
1.53
1.51
1.51
1.51
1.50
1.50
1.38
1.38
1.37
1.35
1.34
0.94
0.92
2.18
1.98
3.12
2.47
0.57
2.00
2.28
2.39
3.54
0.5 10.0 9.5 9.0 8.5 8.0 7.5 7.0 6.5 6.0 5.5 5.0 4.5 4.0 3.5 3.0 2.5 2.0 1.5 1.0 0.5 0.0 -0.5 -1
f1 (ppm)

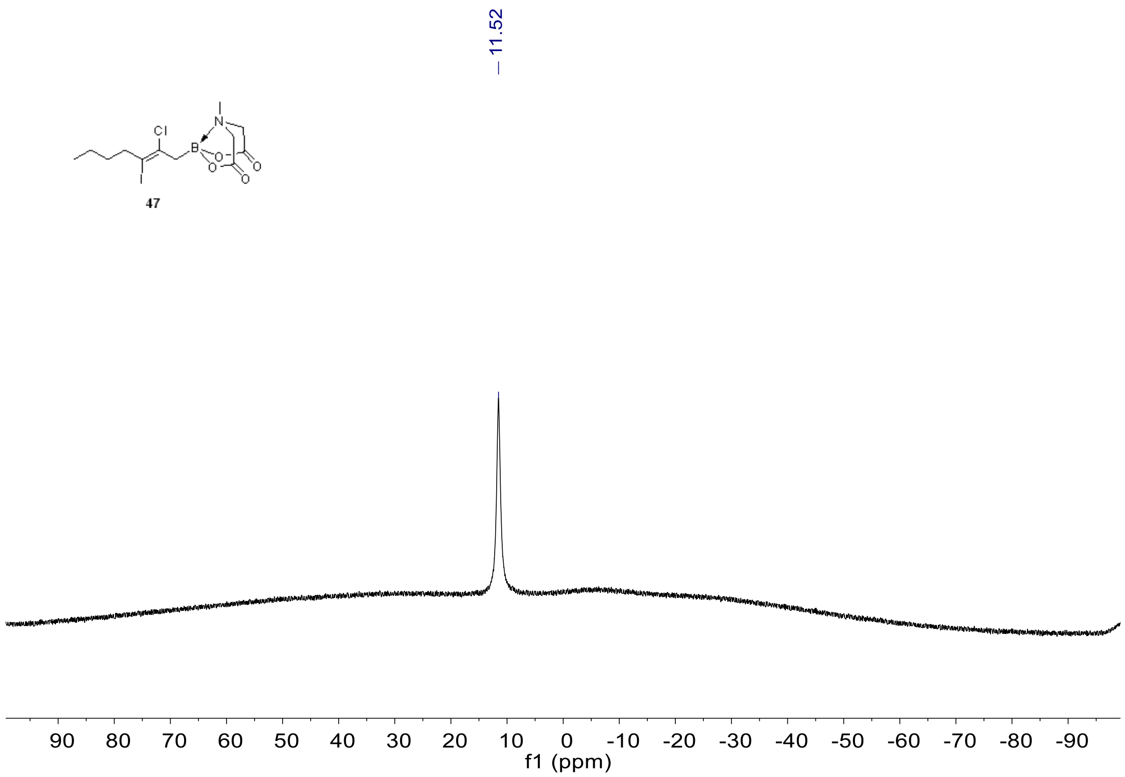
11.52
Cl
N
B
O
I
47
90 80 70 60 50 40 30 20 10 0 -10 -20 -30 -40 -50 -60 -70 -80 -90
f1 (ppm)

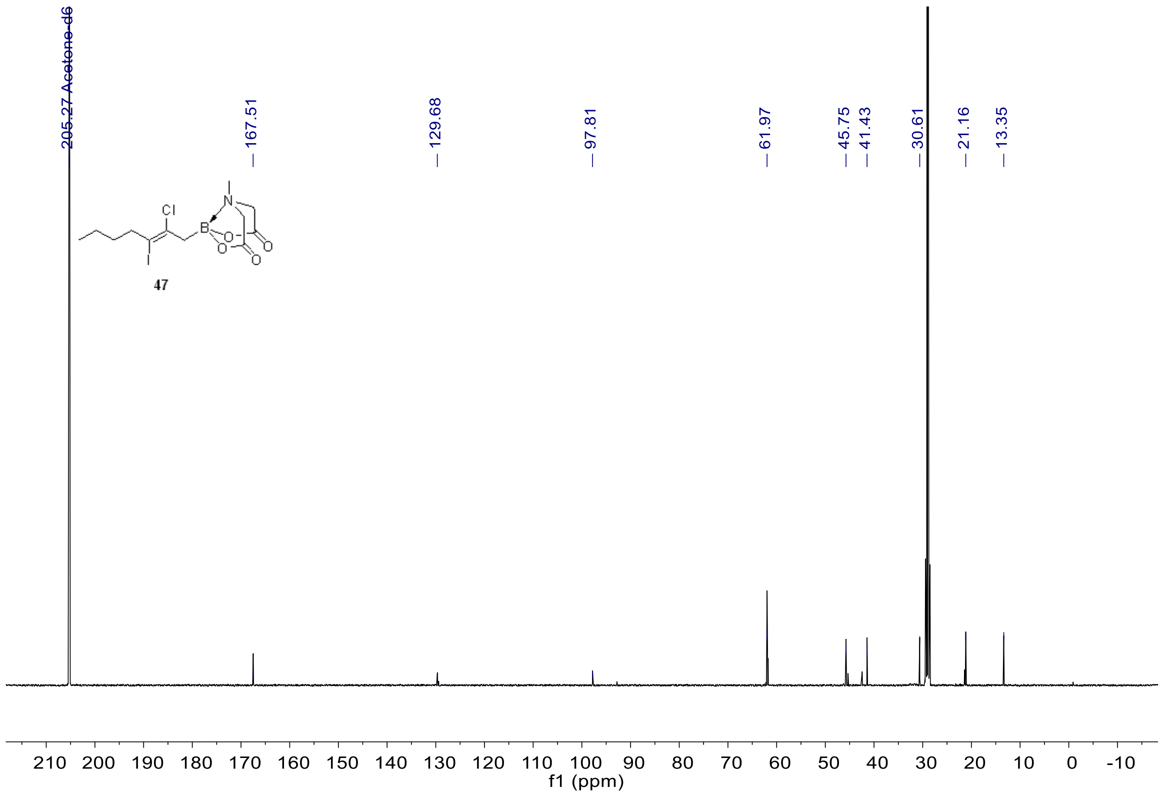
205.27 Acetone-d6
167.51
129.68
97.81
61.97
45.75
41.43
30.61
21.16
13.35
Cl
N
B
O
I
47
210 200 190 180 170 160 150 140 130 120 110 100 90 80 70 60 50 40 30 20 10 0 -10
f1 (ppm)

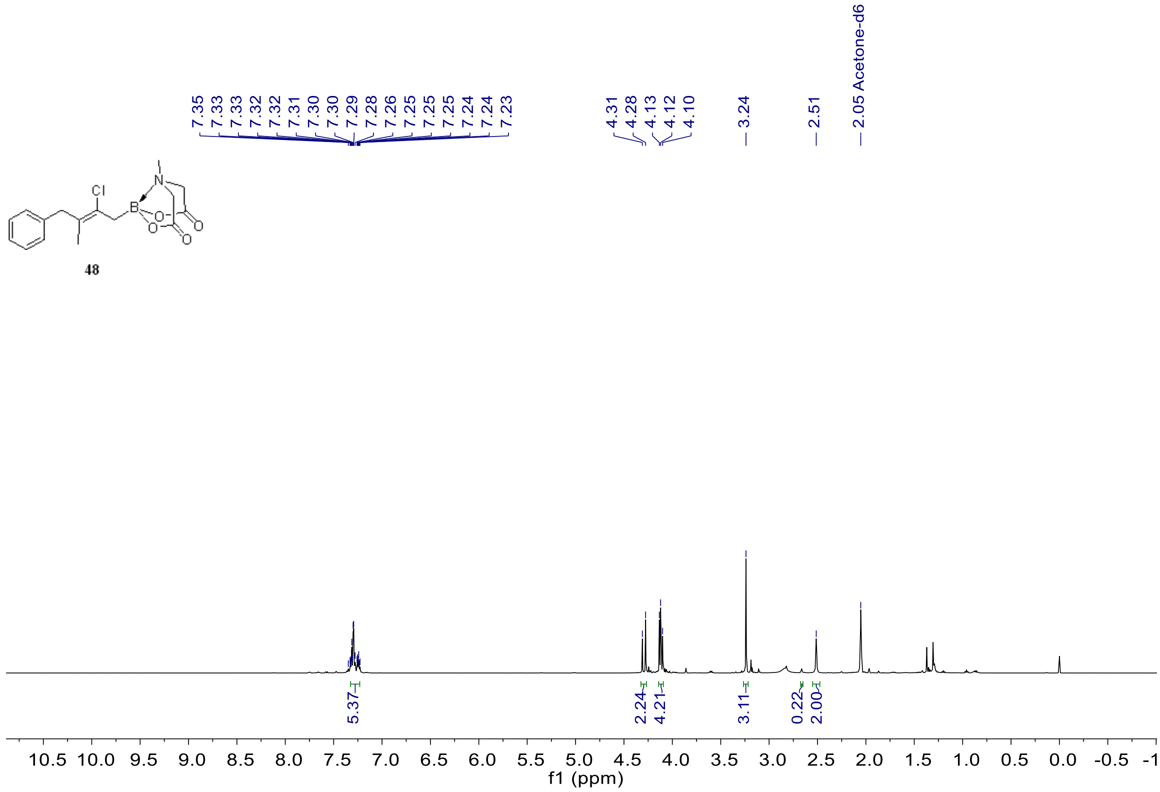
48
7.35 7.33 7.33 7.32 7.32 7.31 7.30 7.30 7.29 7.28 7.26 7.25 7.25 7.25 7.24 7.24 7.23
4.31 4.28 4.13 4.12 4.10
3.24
2.51
2.05 Acetone-d6
5.37
2.24
4.21
3.11
0.22
2.00
f1 (ppm)

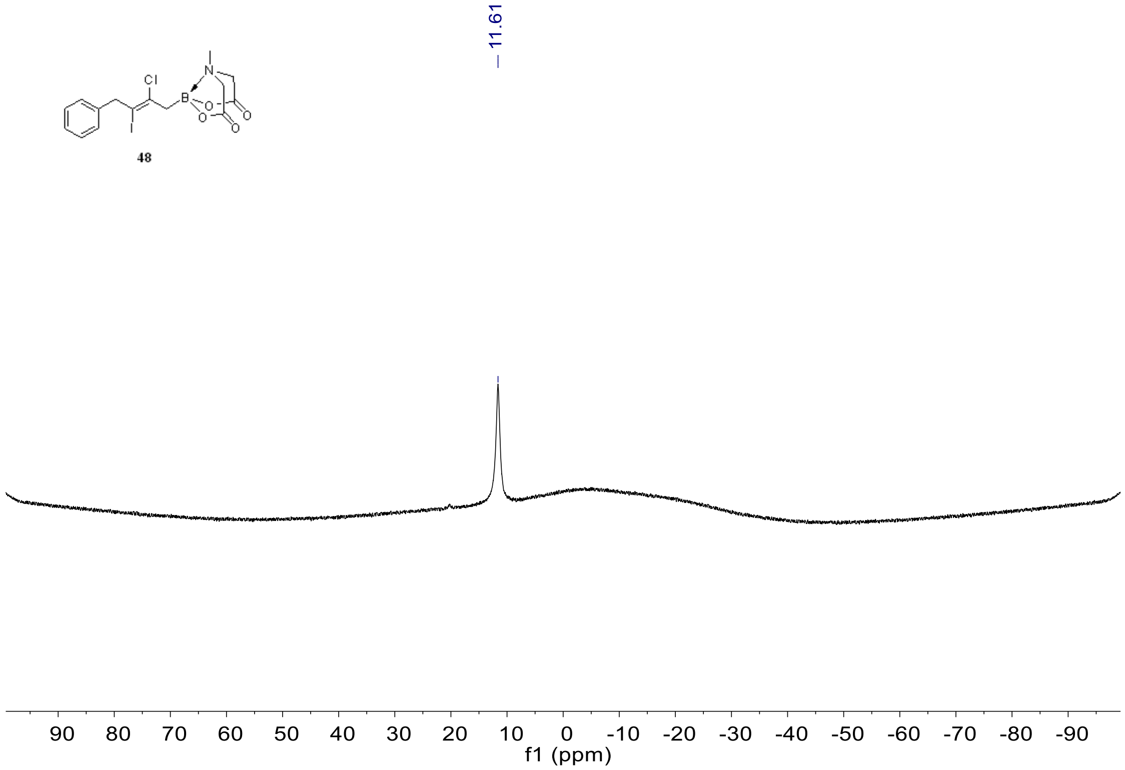
48
11.61
f1 (ppm)

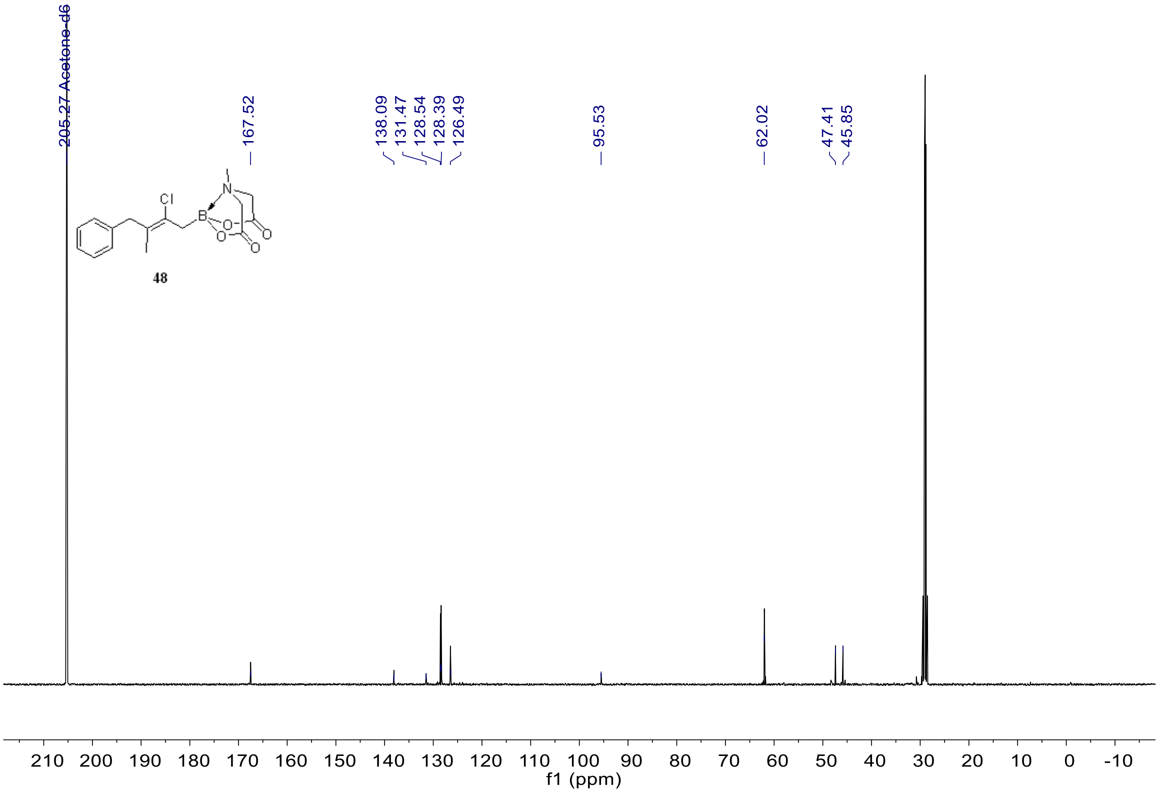
205.27 Acetone-d6
167.52
138.09
131.47
128.54
128.39
126.49
95.53
62.02
47.41
45.85
Cl
N
B
O
O
O
O
48
210 200 190 180 170 160 150 140 130 120 110 100 90 80 70 60 50 40 30 20 10 0 -10
f1 (ppm)

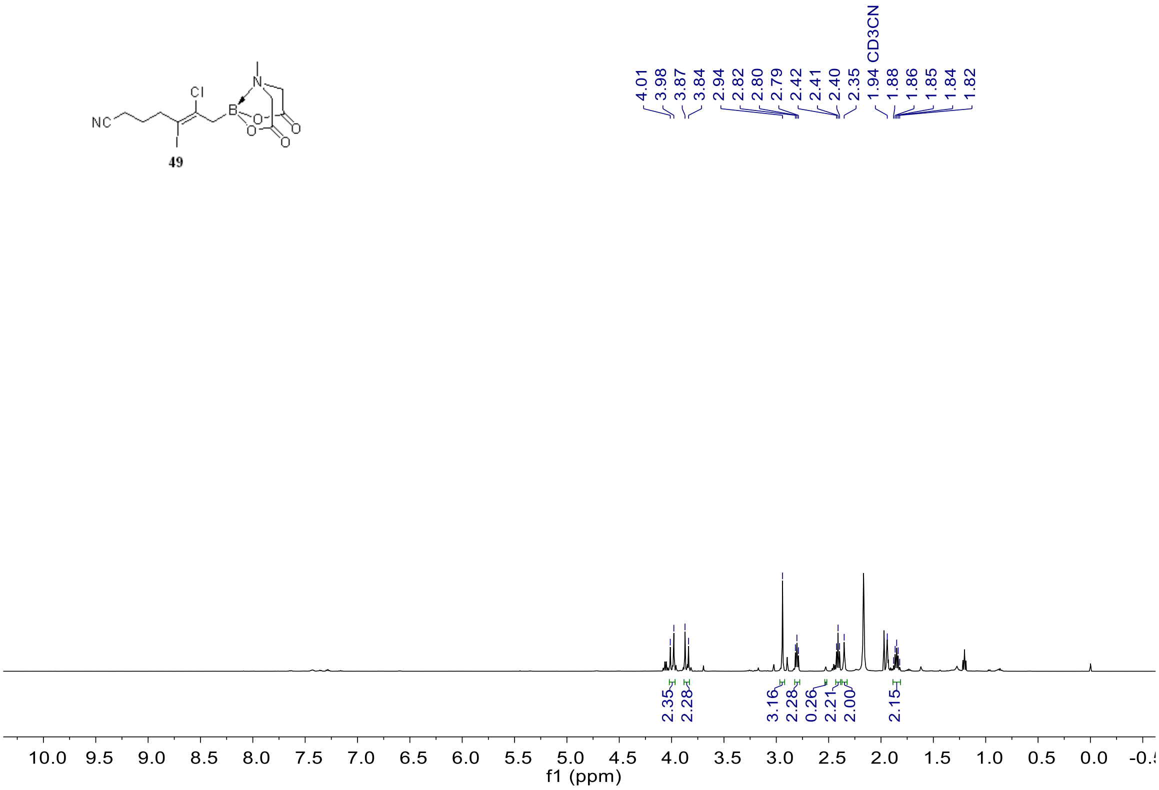
NC
Cl
N
B
O
O
O
O
49
4.01
3.98
3.87
3.84
2.94
2.82
2.80
2.79
2.42
2.41
2.40
2.35
1.94 CD3CN
1.88
1.86
1.85
1.84
1.82
2.35
2.28
3.16
2.28
0.26
2.21
2.00
2.15
10.0 9.5 9.0 8.5 8.0 7.5 7.0 6.5 6.0 5.5 5.0 4.5 4.0 3.5 3.0 2.5 2.0 1.5 1.0 0.5 0.0
f1 (ppm)

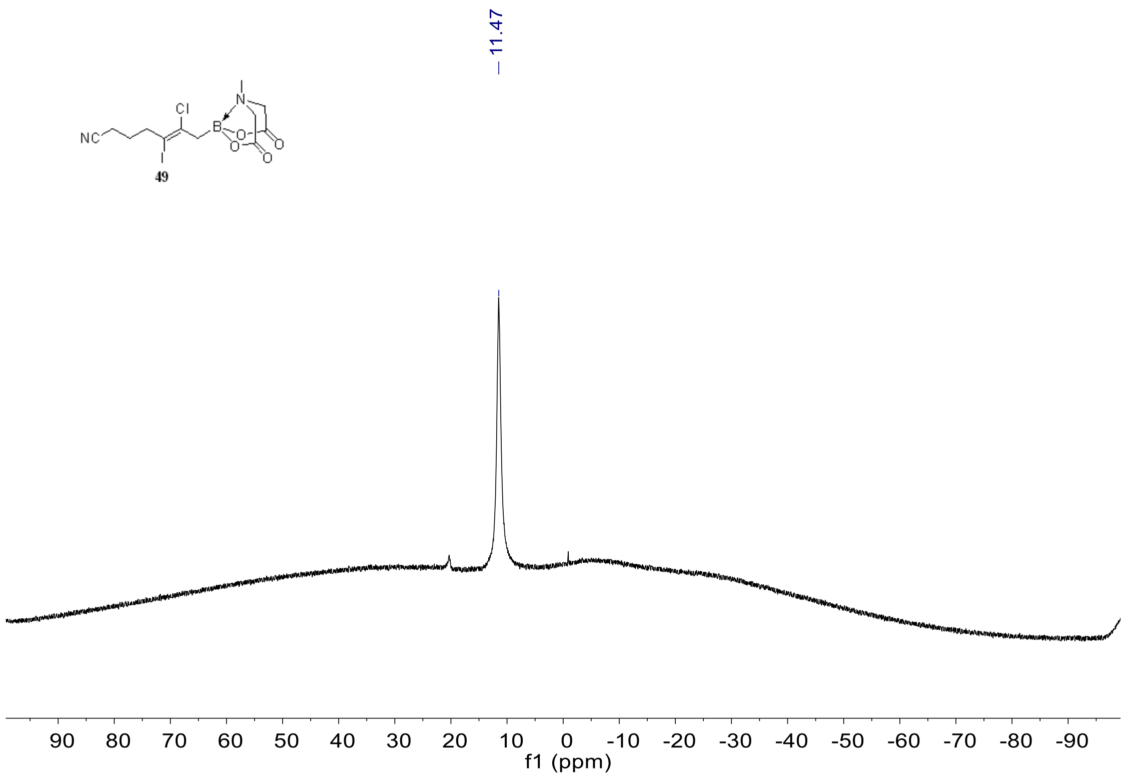
11.47
NC
Cl
N
B
O
49
90 80 70 60 50 40 30 20 10 0 -10 -20 -30 -40 -50 -60 -70 -80 -90
f1 (ppm)

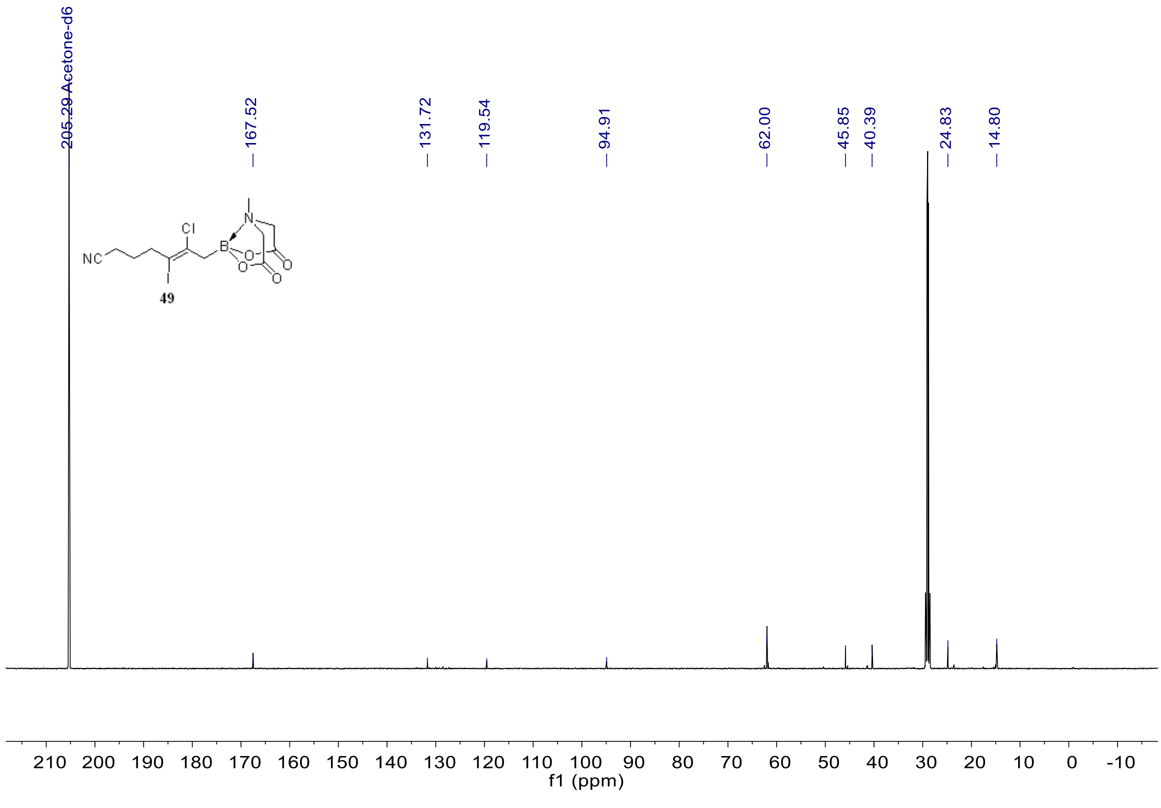
205.29 Acetone-d6
167.52
131.72
119.54
94.91
62.00
45.85
40.39
24.83
14.80
NC
Cl
N
B
O
49
210 200 190 180 170 160 150 140 130 120 110 100 90 80 70 60 50 40 30 20 10 0 -10
f1 (ppm)

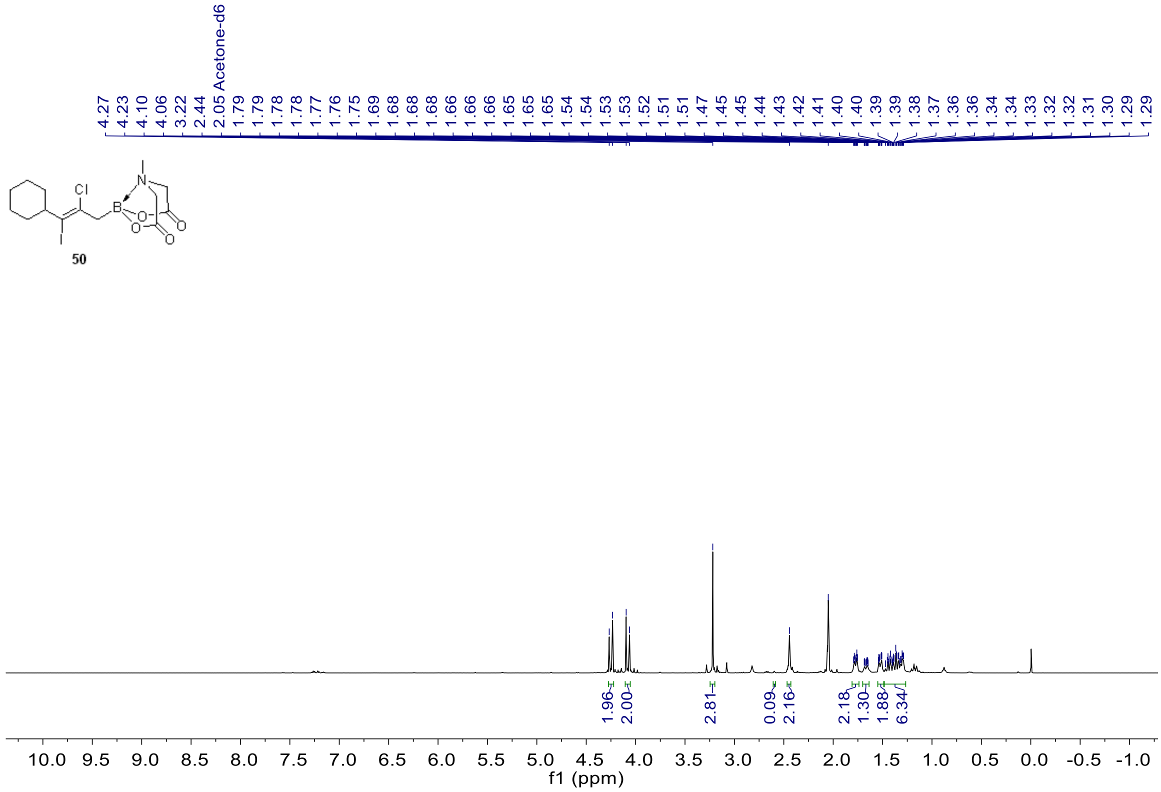
4.27
4.23
4.10
4.06
3.22
2.44
2.05 Acetone-d6
1.79
1.79
1.78
1.78
1.77
1.76
1.75
1.69
1.68
1.68
1.68
1.66
1.66
1.65
1.65
1.65
1.54
1.54
1.53
1.53
1.52
1.51
1.51
1.47
1.45
1.45
1.44
1.43
1.42
1.41
1.40
1.39
1.39
1.38
1.37
1.36
1.36
1.34
1.33
1.32
1.32
1.31
1.30
1.29
1.29
Cl
N
B
O
O
O
O
I
50
1.96
2.00
2.81
0.09
2.16
2.18
1.30
1.88
6.34
10.0 9.5 9.0 8.5 8.0 7.5 7.0 6.5 6.0 5.5 5.0 4.5 4.0 3.5 3.0 2.5 2.0 1.5 1.0 0.5 0.0 -0.5 -1.0
f1 (ppm)

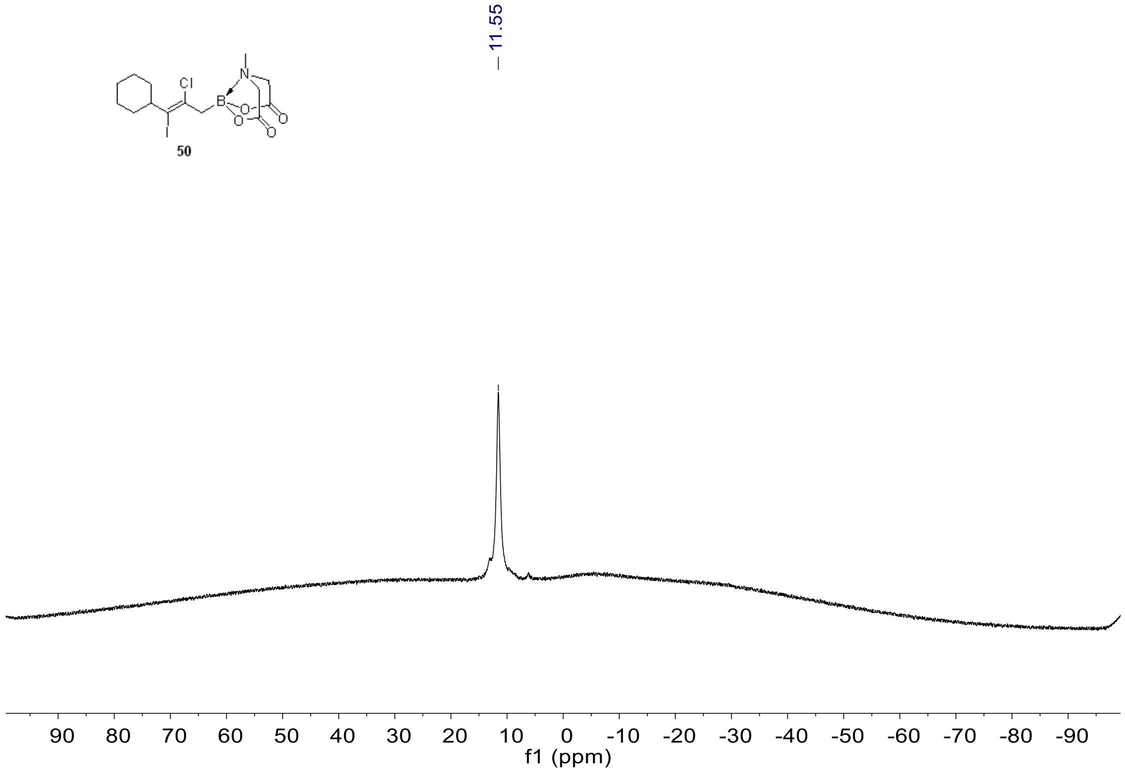
11.55
Cl
N
B
O
O
O
O
I
50
90 80 70 60 50 40 30 20 10 0 -10 -20 -30 -40 -50 -60 -70 -80 -90
f1 (ppm)

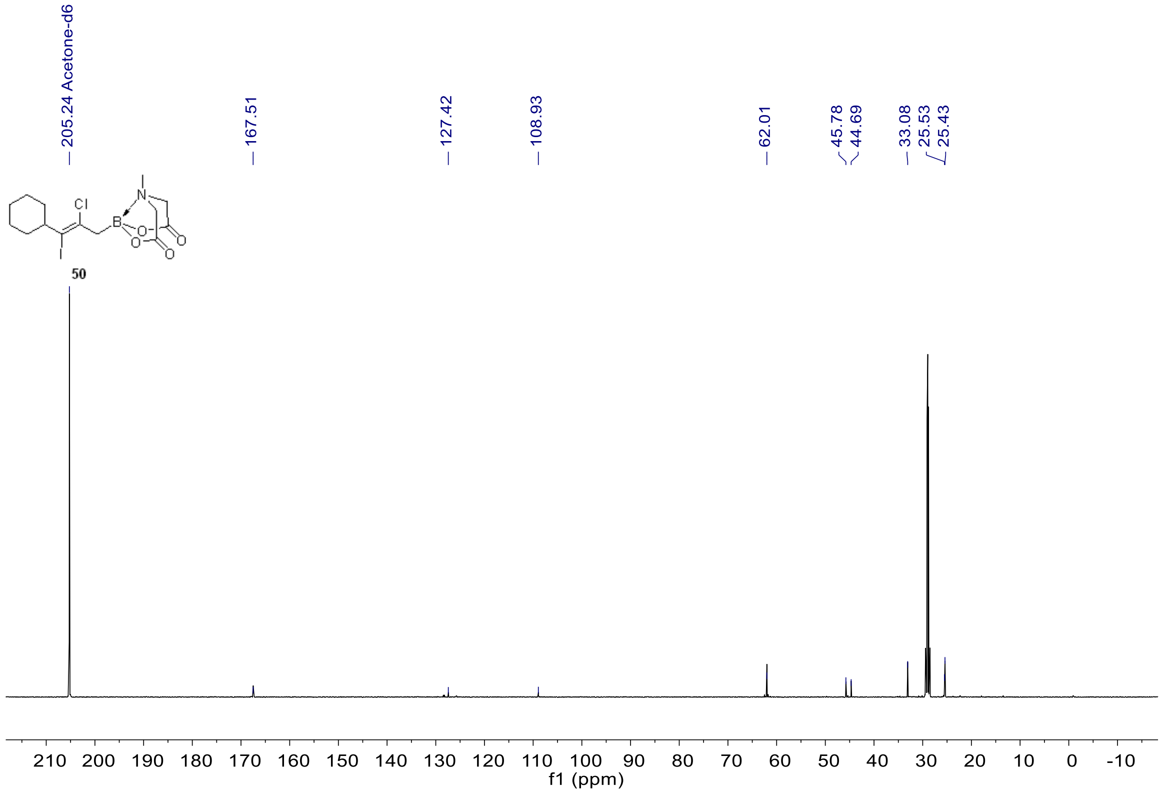
205.24 Acetone-d6
167.51
127.42
108.93
62.01
45.78
44.69
33.08
25.53
25.43
Cl
N
B
O
O
O
I
50
210 200 190 180 170 160 150 140 130 120 110 100 90 80 70 60 50 40 30 20 10 0 -10
f1 (ppm)

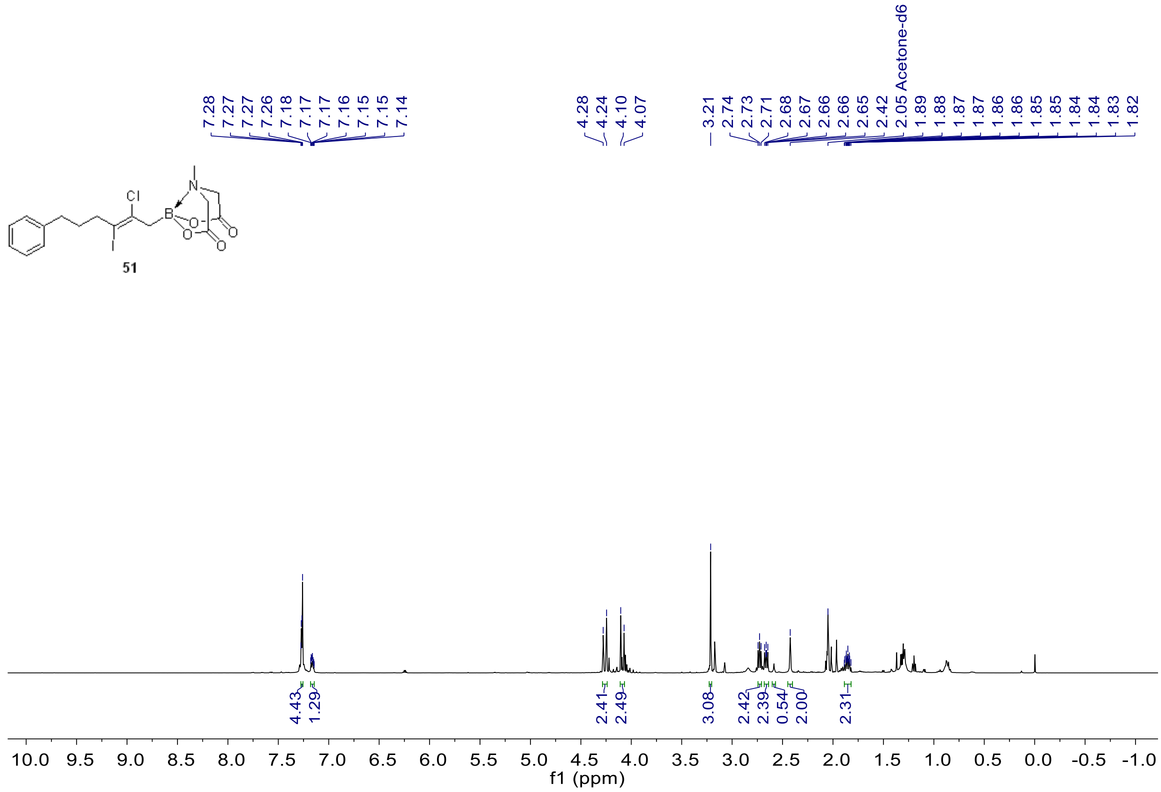
7.28
7.27
7.27
7.26
7.18
7.17
7.17
7.16
7.15
7.15
7.14
4.28
4.24
4.10
4.07
3.21
2.74
2.73
2.71
2.68
2.67
2.66
2.66
2.65
2.42
2.05 Acetone-d6
1.89
1.88
1.87
1.87
1.86
1.86
1.85
1.85
1.84
1.84
1.83
1.82
Cl
N
B
O
O
O
I
51
4.43
1.29
2.41
2.49
3.08
2.42
2.39
0.54
2.00
2.31
10.0 9.5 9.0 8.5 8.0 7.5 7.0 6.5 6.0 5.5 5.0 4.5 4.0 3.5 3.0 2.5 2.0 1.5 1.0 0.5 0.0 -0.5 -1.0
f1 (ppm)

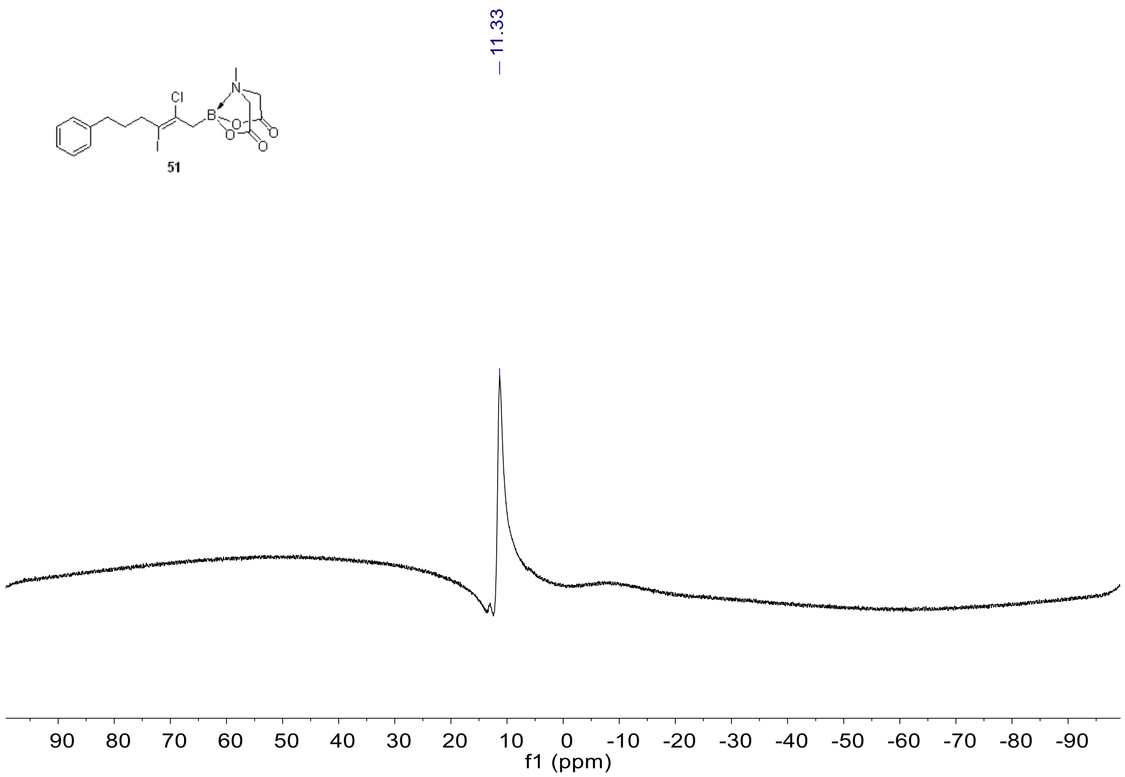
11.33
Cl
N
B
O
O
I
51
90 80 70 60 50 40 30 20 10 0 -10 -20 -30 -40 -50 -60 -70 -80 -90
f1 (ppm)

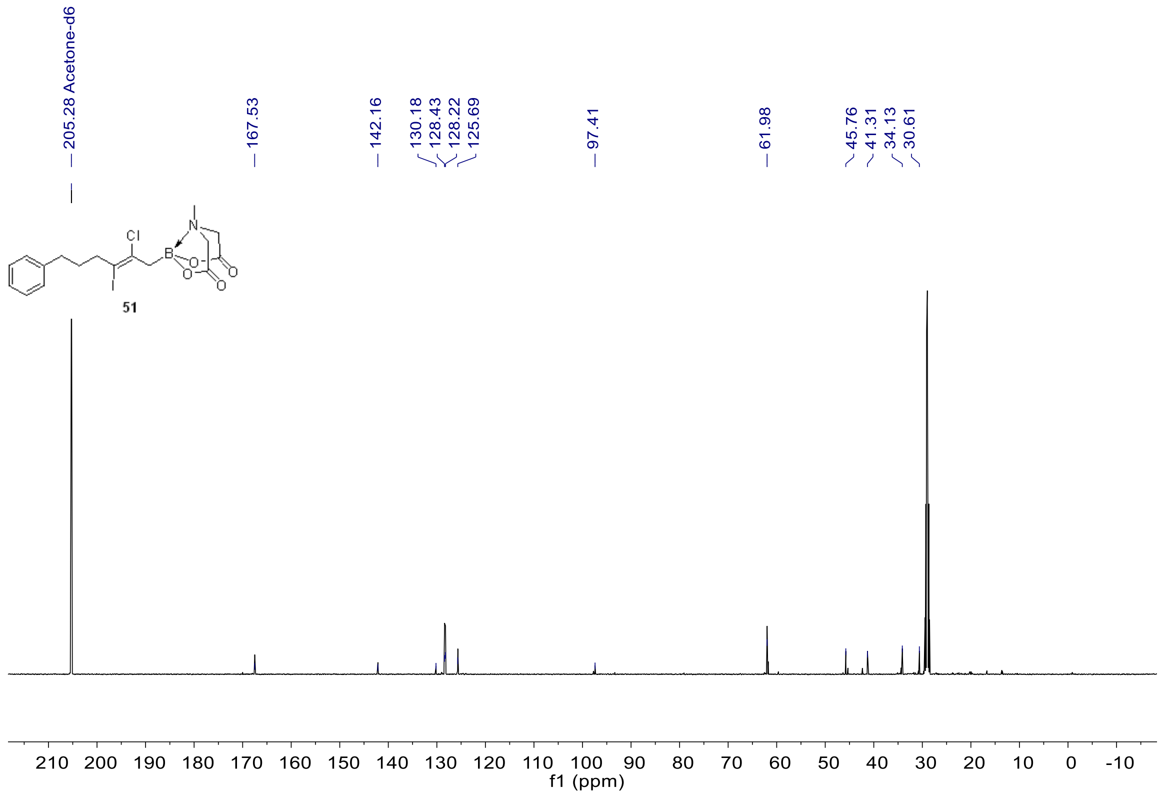
205.28 Acetone-d6
167.53
142.16
130.18
128.43
128.22
125.69
97.41
61.98
45.76
41.31
34.13
30.61
Cl
N
B
O
O
I
51
210 200 190 180 170 160 150 140 130 120 110 100 90 80 70 60 50 40 30 20 10 0 -10
f1 (ppm)

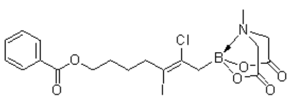

52

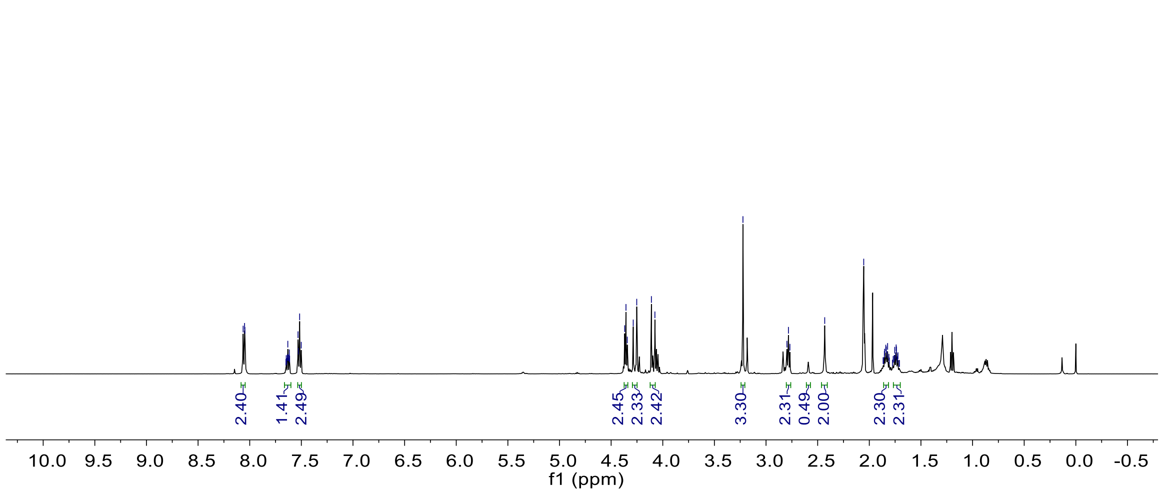
8.06
8.05
8.05
7.65
7.65
7.64
7.63
7.63
7.63
7.62
7.62
7.61
7.53
7.53
7.52
7.50
4.37
4.36
4.34
4.29
4.25
4.11
4.08
3.22
2.80
2.78
2.77
2.43
2.05 Acetone-d6
1.85
1.85
1.84
1.84
1.83
1.82
1.81
1.77
1.76
1.75
1.74
1.74
1.74
1.73
2.40
1.41
2.49
2.45
2.33
2.42
3.30
2.31
0.49
2.00
2.30
2.31
10.0 9.5 9.0 8.5 8.0 7.5 7.0 6.5 6.0 5.5 5.0 4.5 4.0 3.5 3.0 2.5 2.0 1.5 1.0 0.5 0.0 -0.5
f1 (ppm)

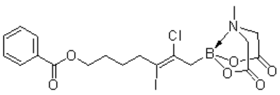

52

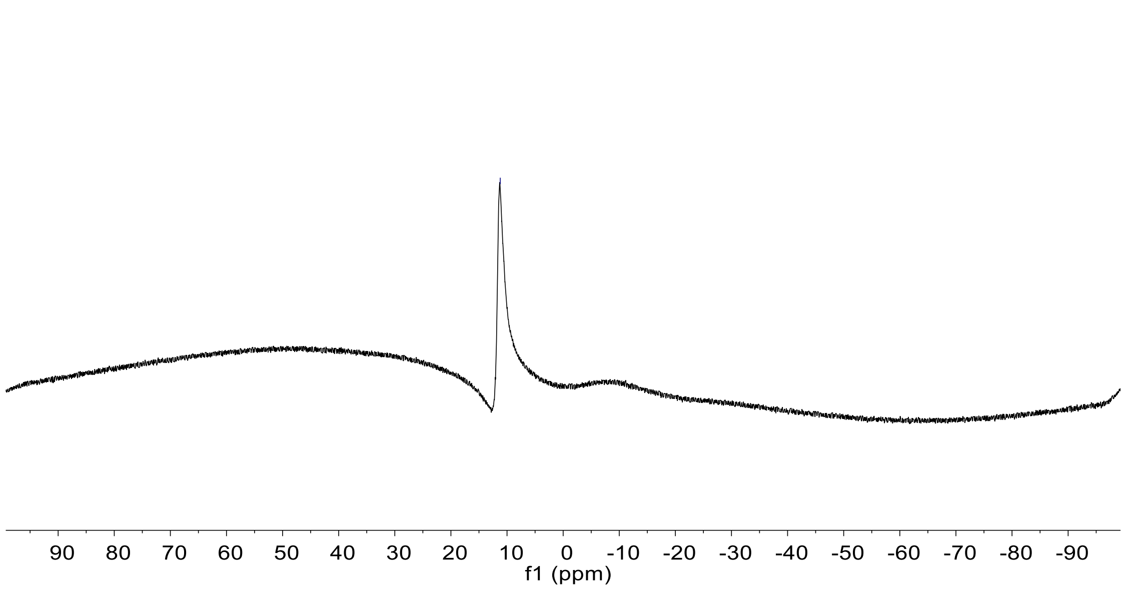
11.21
90 80 70 60 50 40 30 20 10 0 -10 -20 -30 -40 -50 -60 -70 -80 -90
f1 (ppm)

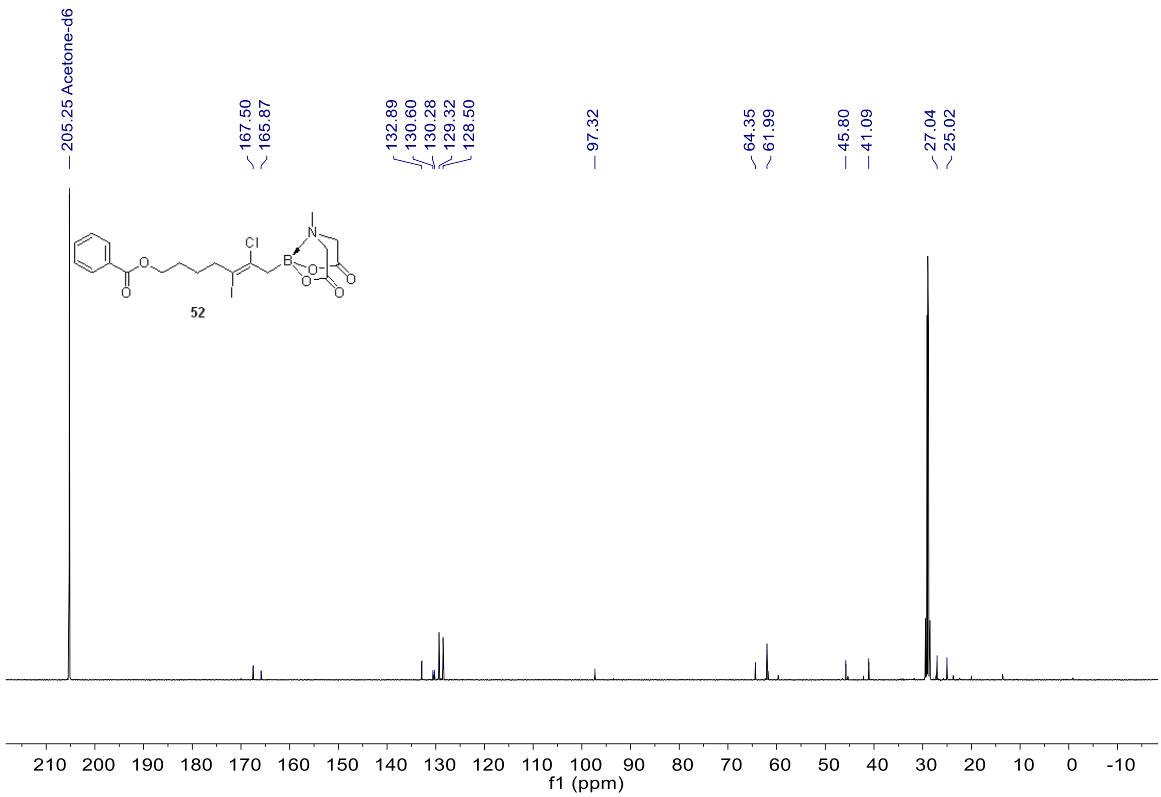
205.25 Acetone-d6
167.50
165.87
132.89
130.60
130.28
129.32
128.50
97.32
64.35
61.99
45.80
41.09
27.04
25.02
Cl
O
N
B
I
52
210 200 190 180 170 160 150 140 130 120 110 100 90 80 70 60 50 40 30 20 10 0 -10
f1 (ppm)

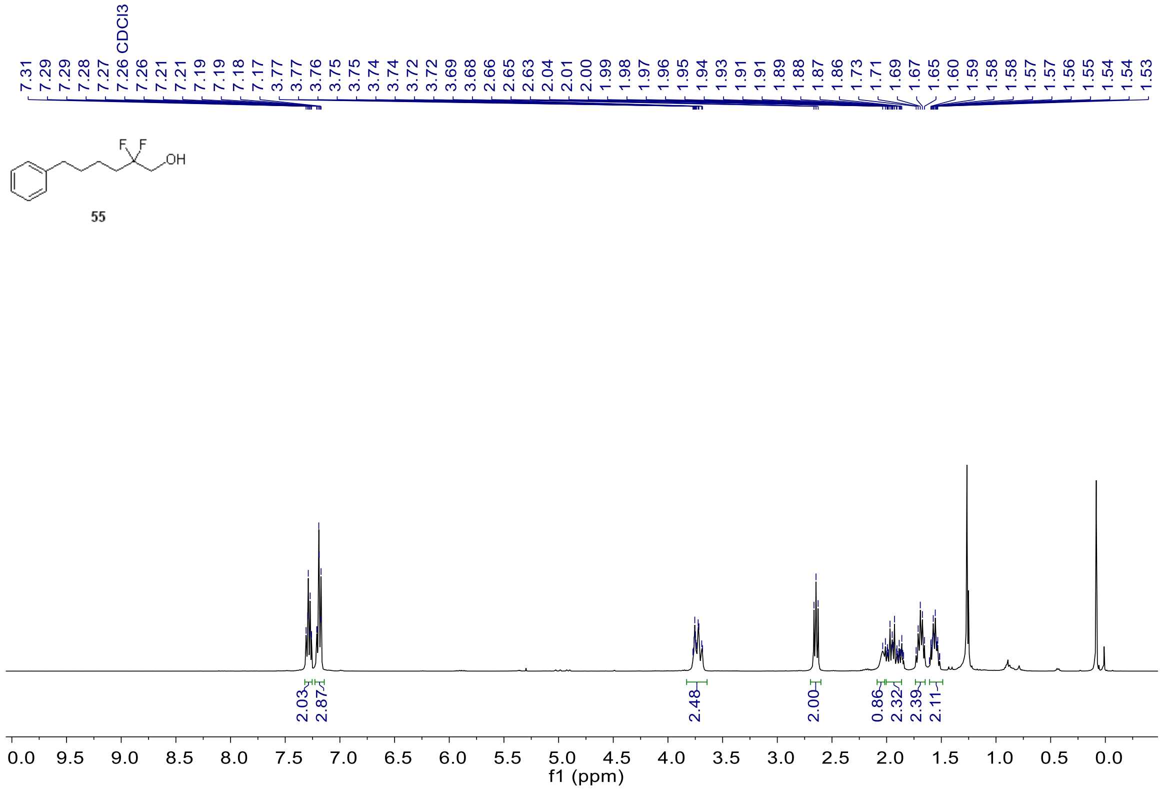
7.31 7.29 7.29 7.28 7.27 7.26 CDCl3 7.26 7.21 7.21 7.19 7.19 7.18 7.17 3.77 3.77 3.76 3.75 3.74 3.74 3.72 3.72 3.69 3.68 2.66 2.65 2.63 2.04 2.01 2.00 1.99 1.98 1.97 1.96 1.95 1.94 1.93 1.91 1.91 1.89 1.88 1.87 1.86 1.73 1.71 1.69 1.67 1.65 1.60 1.59 1.58 1.58 1.57 1.56 1.55 1.54 1.54 1.53
F F
OH
55
2.03
2.87
2.48
2.00
0.86
2.32
2.39
2.11
0.0 9.5 9.0 8.5 8.0 7.5 7.0 6.5 6.0 5.5 5.0 4.5 4.0 3.5 3.0 2.5 2.0 1.5 1.0 0.5 0.0
f1 (ppm)

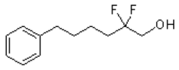

55

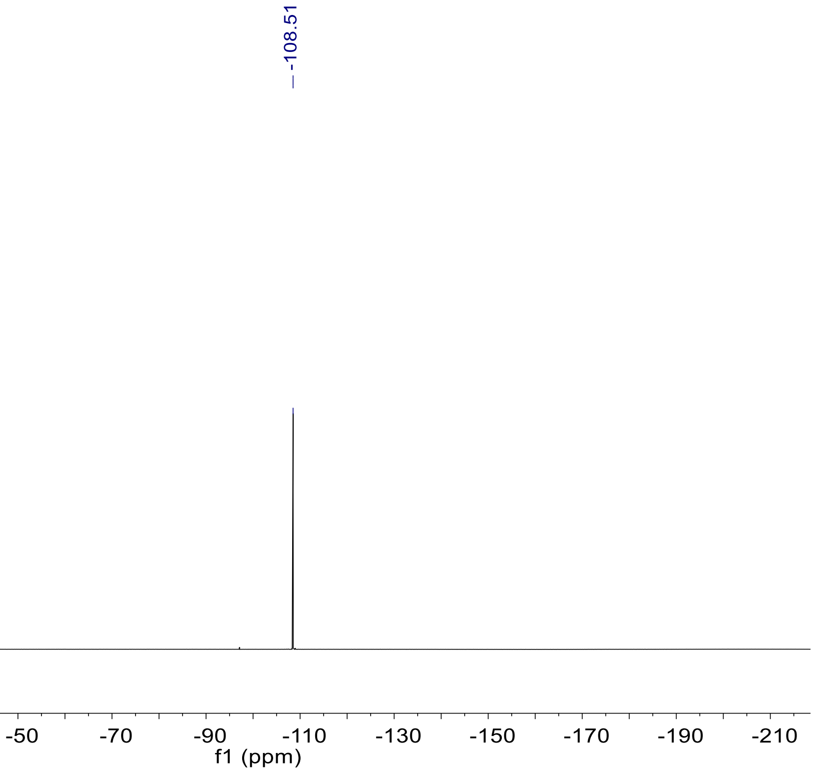
-108.51
10
-10
-30
-50
-70
-90
-110
-130
-150
-170
-190
-210
f1 (ppm)

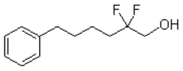

55

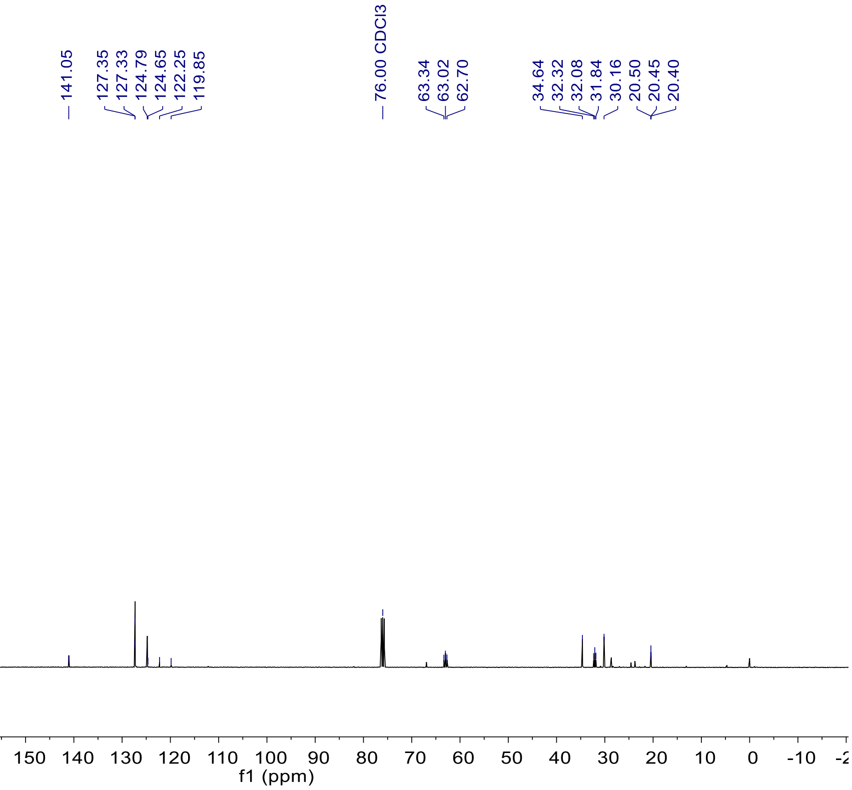
141.05
127.35
127.33
124.79
124.65
122.25
119.85
76.00 CDCl3
63.34
63.02
62.70
34.64
32.32
32.08
31.84
30.16
20.50
20.45
20.40
210 200 190 180 170 160 150 140 130 120 110 100 90 80 70 60 50 40 30 20 10 0 -10
f1 (ppm)

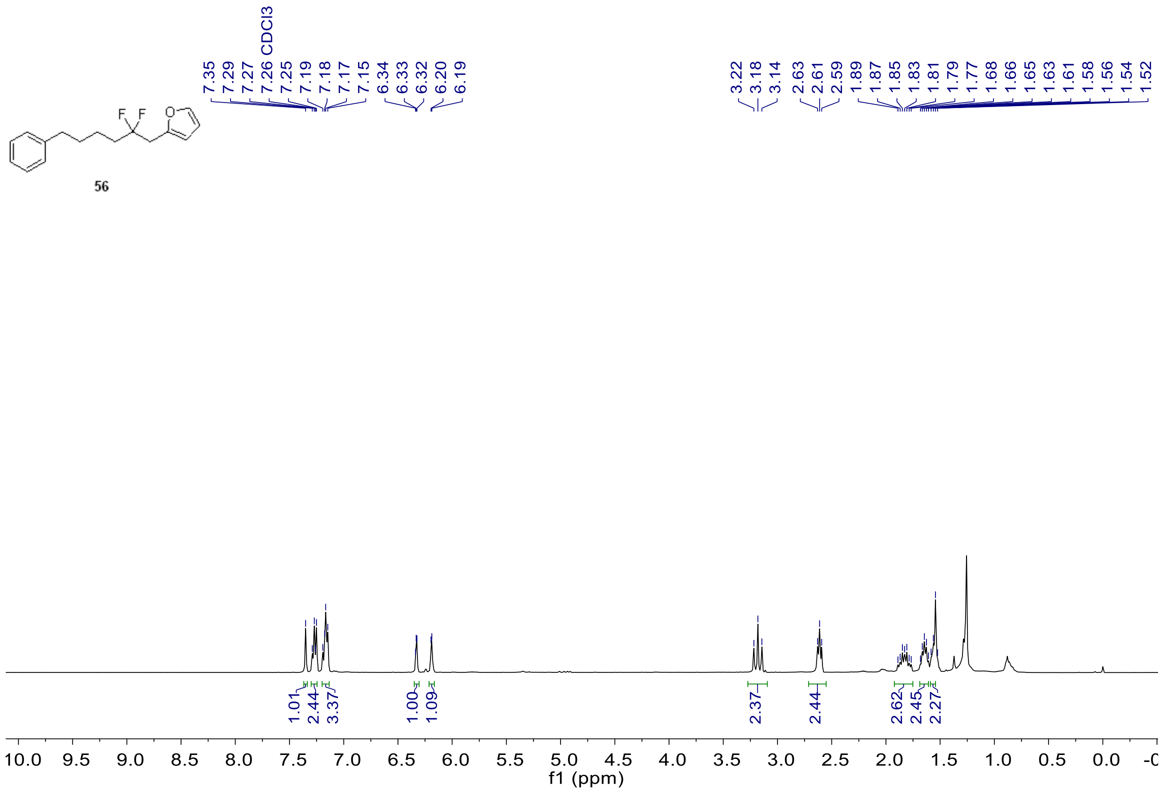

56
CDCl3
7.35 7.29 7.27 7.26 7.25 7.19 7.18 7.17 7.15 6.34 6.33 6.32 6.20 6.19
3.22 3.18 3.14 2.63 2.61 2.59 1.89 1.87 1.85 1.83 1.81 1.79 1.77 1.68 1.66 1.65 1.63 1.61 1.58 1.56 1.54 1.52
1.01 2.44 3.37 1.00 1.09 2.37 2.44 2.62 2.45 2.27
10.0 9.5 9.0 8.5 8.0 7.5 7.0 6.5 6.0 5.5 5.0 4.5 4.0 3.5 3.0 2.5 2.0 1.5 1.0 0.5 0.0
f1 (ppm)

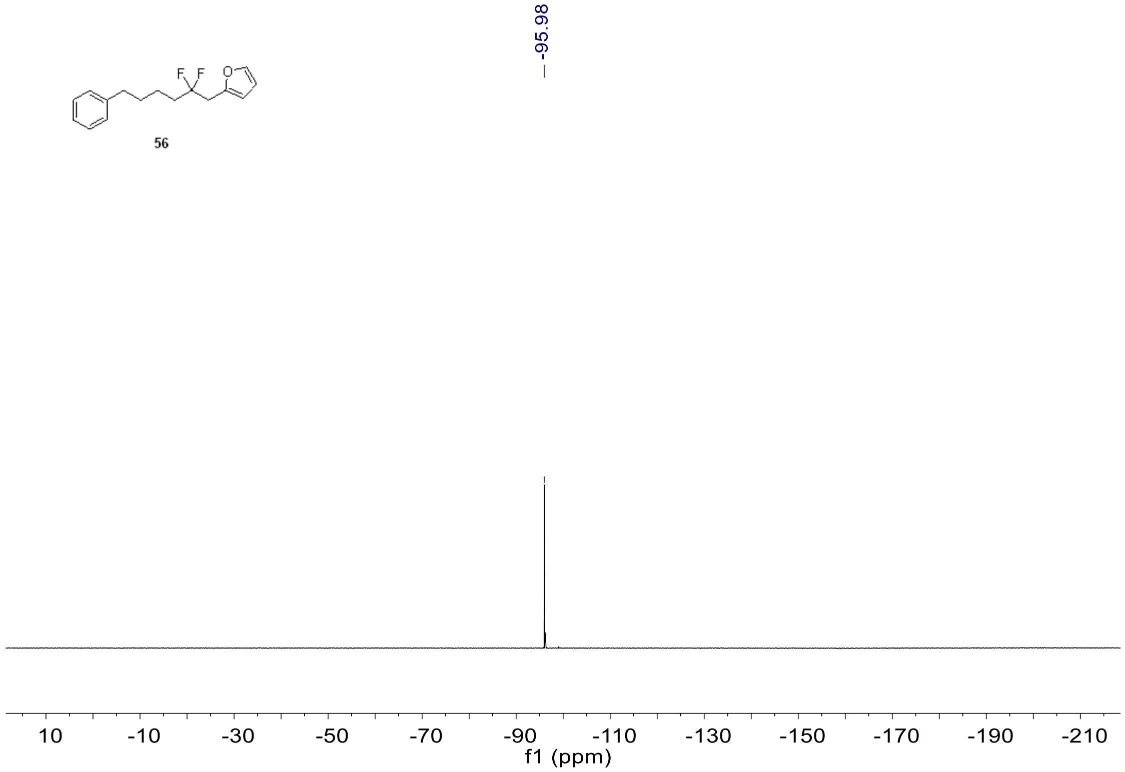

56
-95.98
10 -10 -30 -50 -70 -90 -110 -130 -150 -170 -190 -210
f1 (ppm)

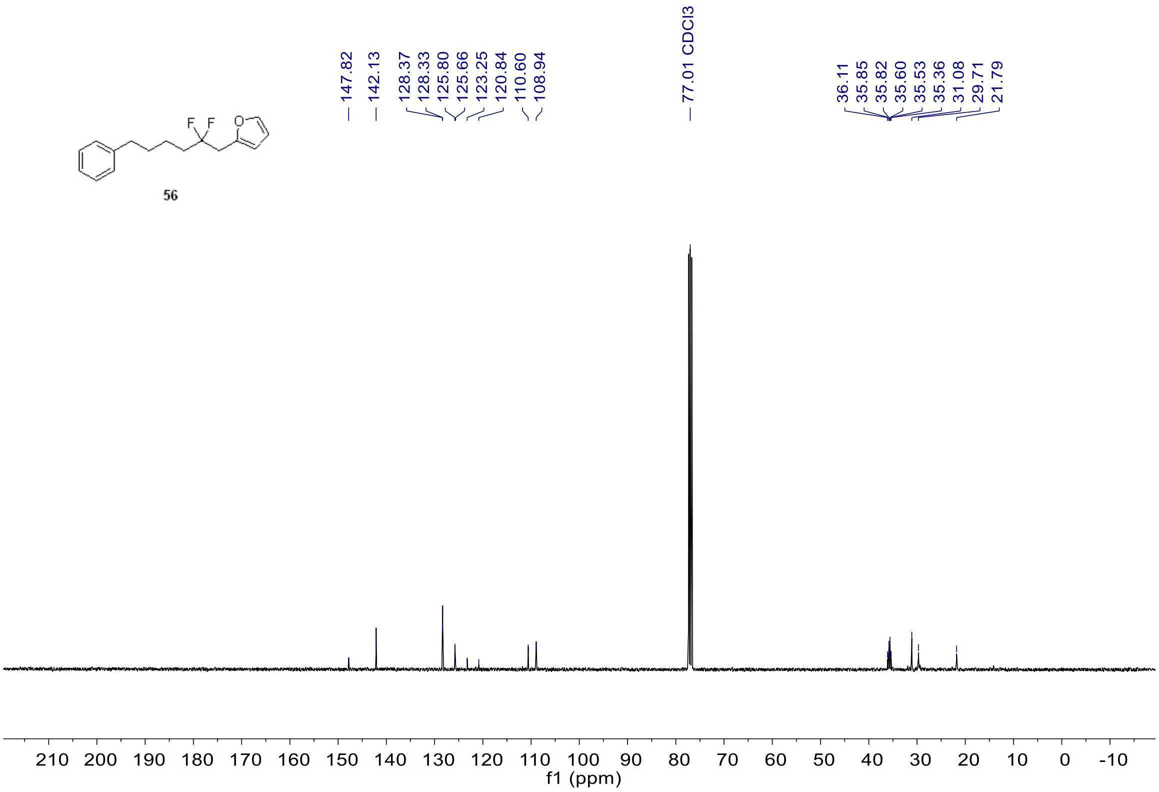
147.82
142.13
128.37
128.33
125.80
125.66
123.25
120.84
110.60
108.94
77.01 CDCl3
36.11
35.85
35.82
35.60
35.53
35.36
31.08
29.71
21.79
210 200 190 180 170 160 150 140 130 120 110 100 90 80 70 60 50 40 30 20 10 0 -10
f1 (ppm)
56

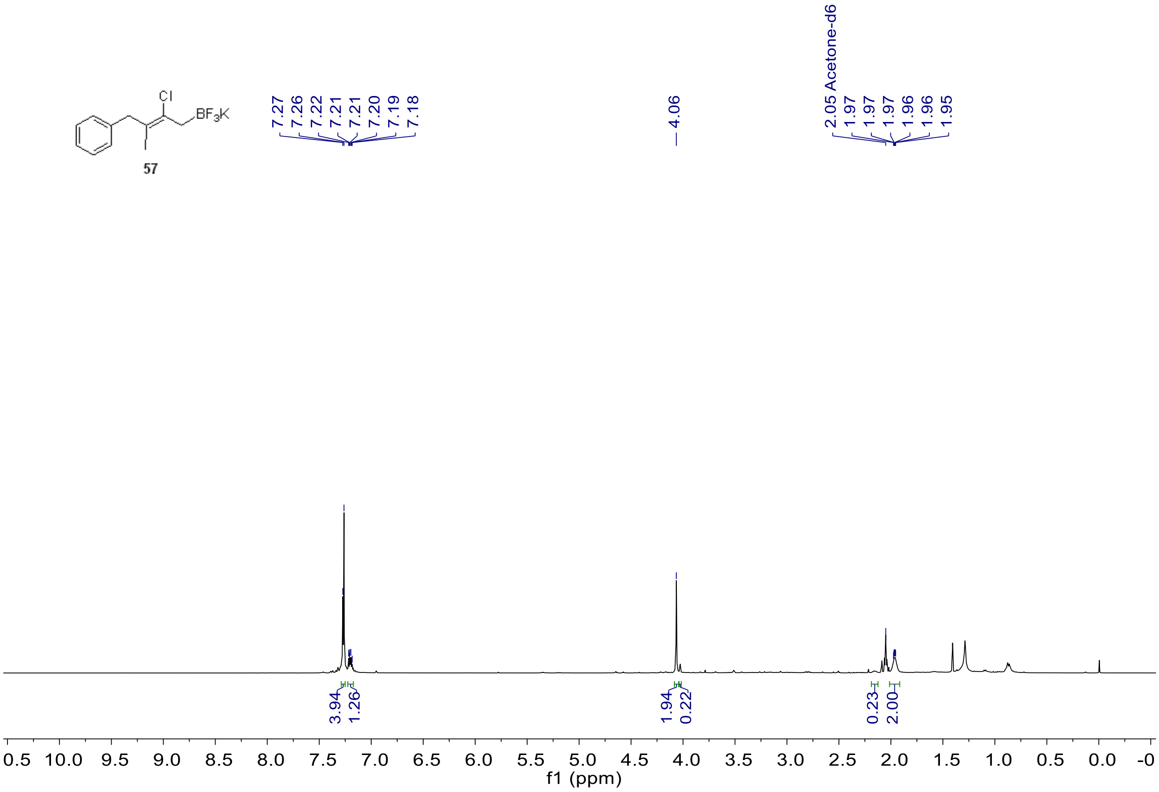
7.27
7.26
7.22
7.21
7.21
7.20
7.19
7.18
4.06
2.05 Acetone-d6
1.97
1.97
1.97
1.96
1.96
1.95
3.94
1.26
1.94
0.22
0.23
2.00
0.5 10.0 9.5 9.0 8.5 8.0 7.5 7.0 6.5 6.0 5.5 5.0 4.5 4.0 3.5 3.0 2.5 2.0 1.5 1.0 0.5 0.0 -0
f1 (ppm)
57

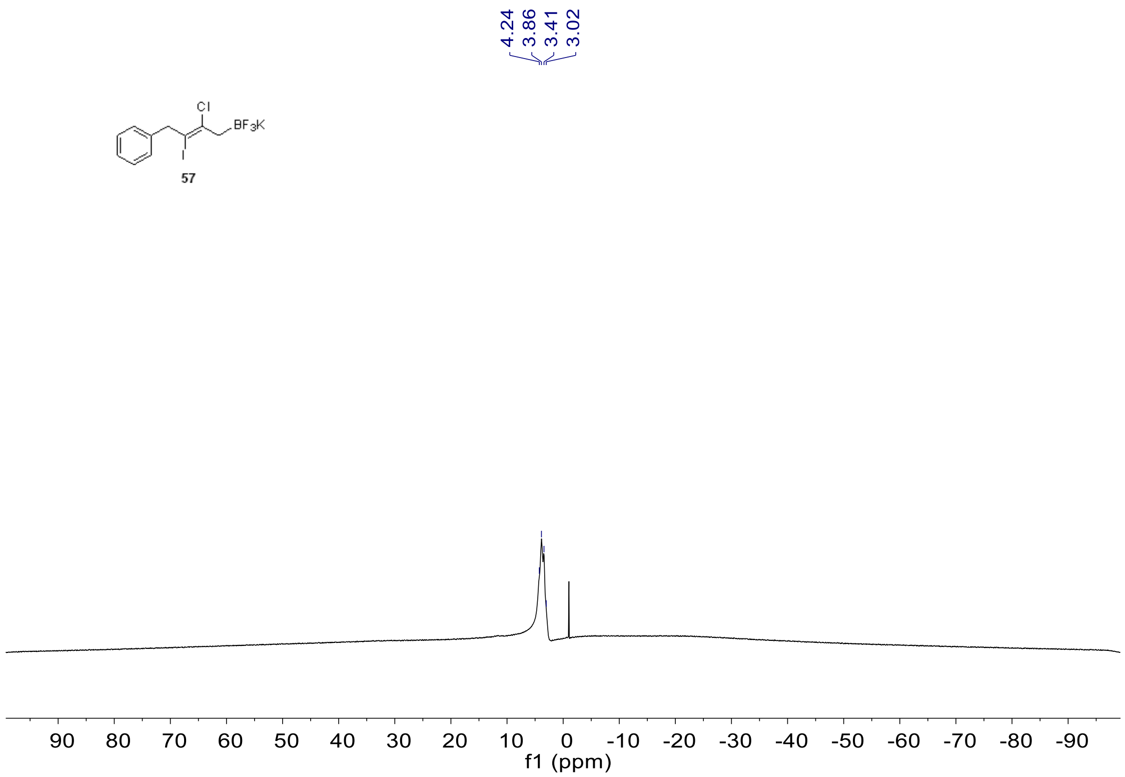
4.24
3.86
3.41
3.02
Cl
BF3K
I
57
90 80 70 60 50 40 30 20 10 0 -10 -20 -30 -40 -50 -60 -70 -80 -90
f1 (ppm)

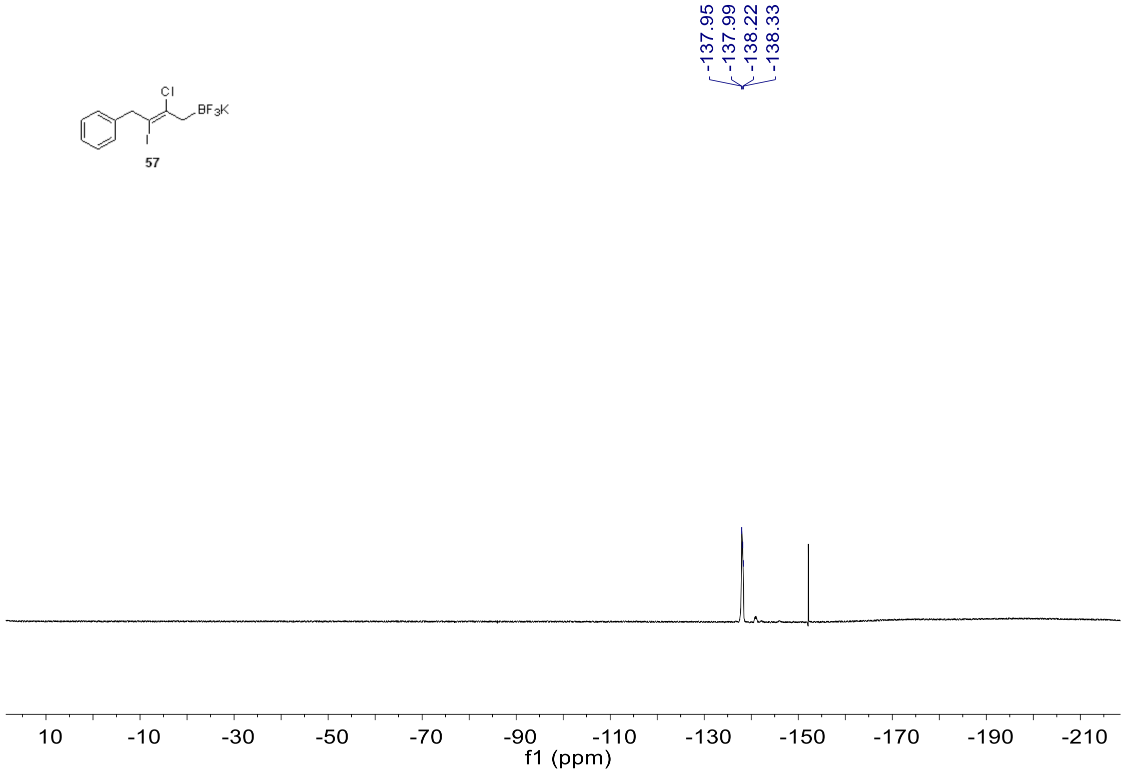
-137.95
-137.99
-138.22
-138.33
Cl
BF3K
I
57
10 -10 -30 -50 -70 -90 -110 -130 -150 -170 -190 -210
f1 (ppm)

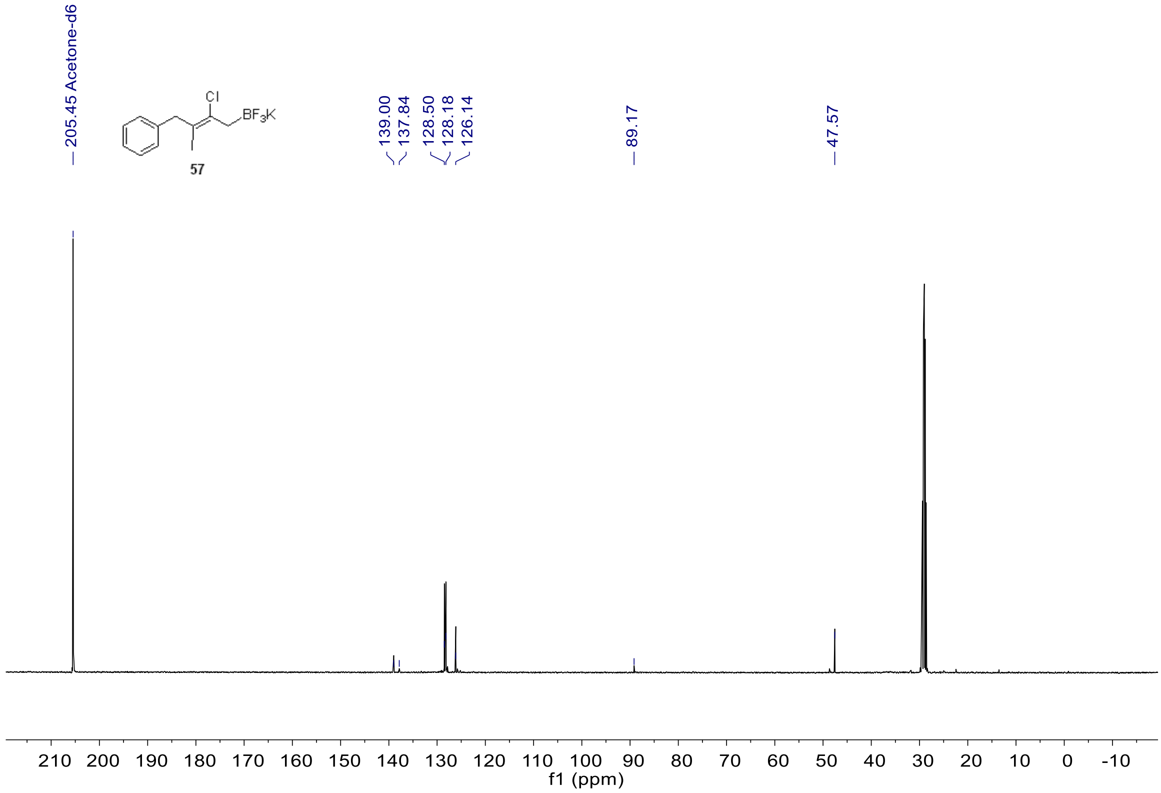
205.45 Acetone-d6
Cl
BF3K
57
139.00
137.84
128.50
128.18
126.14
89.17
47.57
210 200 190 180 170 160 150 140 130 120 110 100 90 80 70 60 50 40 30 20 10 0 -10
f1 (ppm)

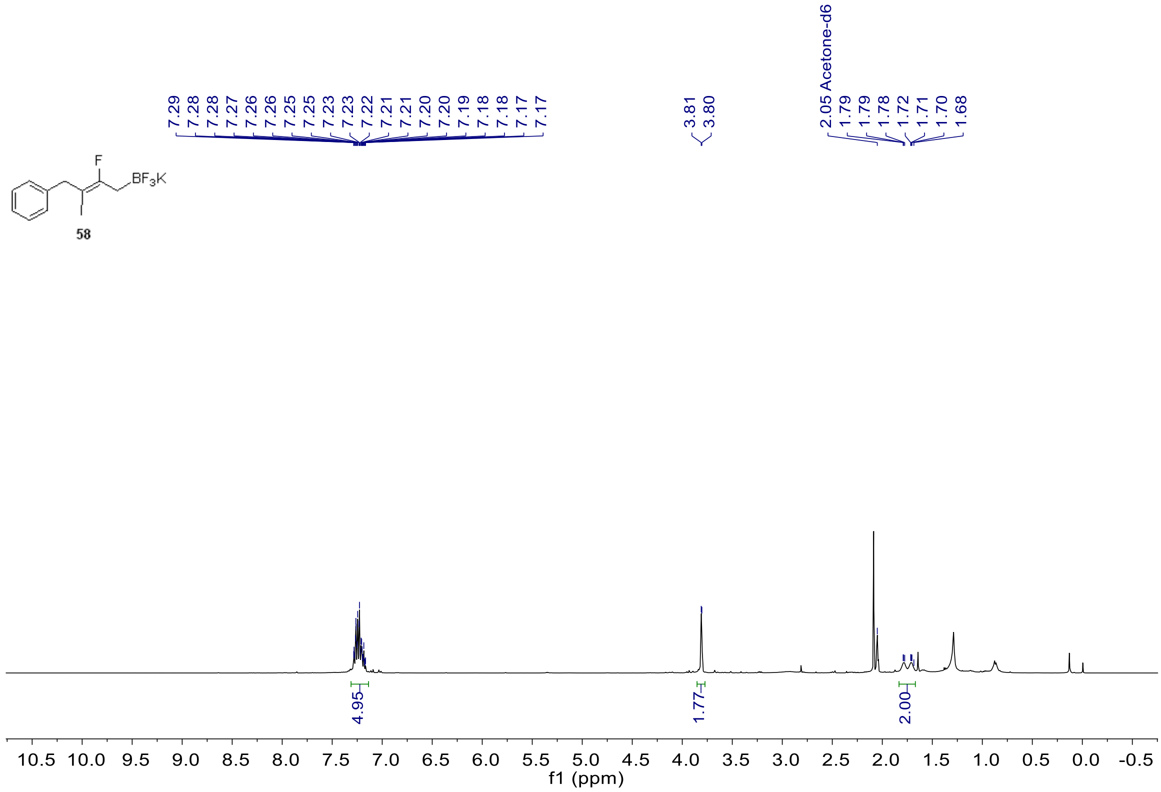
7.29
7.28
7.28
7.27
7.26
7.26
7.25
7.25
7.23
7.23
7.22
7.21
7.21
7.20
7.20
7.19
7.18
7.18
7.17
7.17
3.81
3.80
2.05 Acetone-d6
1.79
1.79
1.78
1.72
1.71
1.70
1.68
F
BF3K
58
4.95
1.77
2.00
10.5 10.0 9.5 9.0 8.5 8.0 7.5 7.0 6.5 6.0 5.5 5.0 4.5 4.0 3.5 3.0 2.5 2.0 1.5 1.0 0.5 0.0 -0.5
f1 (ppm)

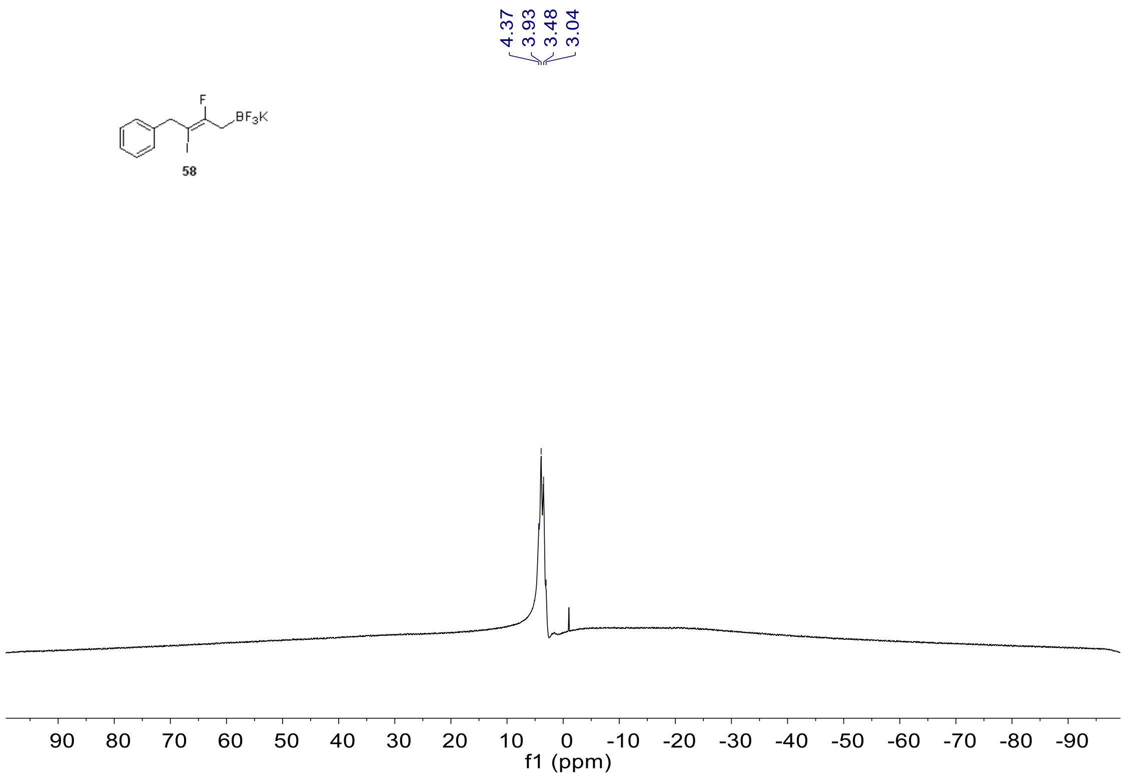
4.37
3.93
3.48
3.04
F
BF3K
I
58
90 80 70 60 50 40 30 20 10 0 -10 -20 -30 -40 -50 -60 -70 -80 -90
f1 (ppm)

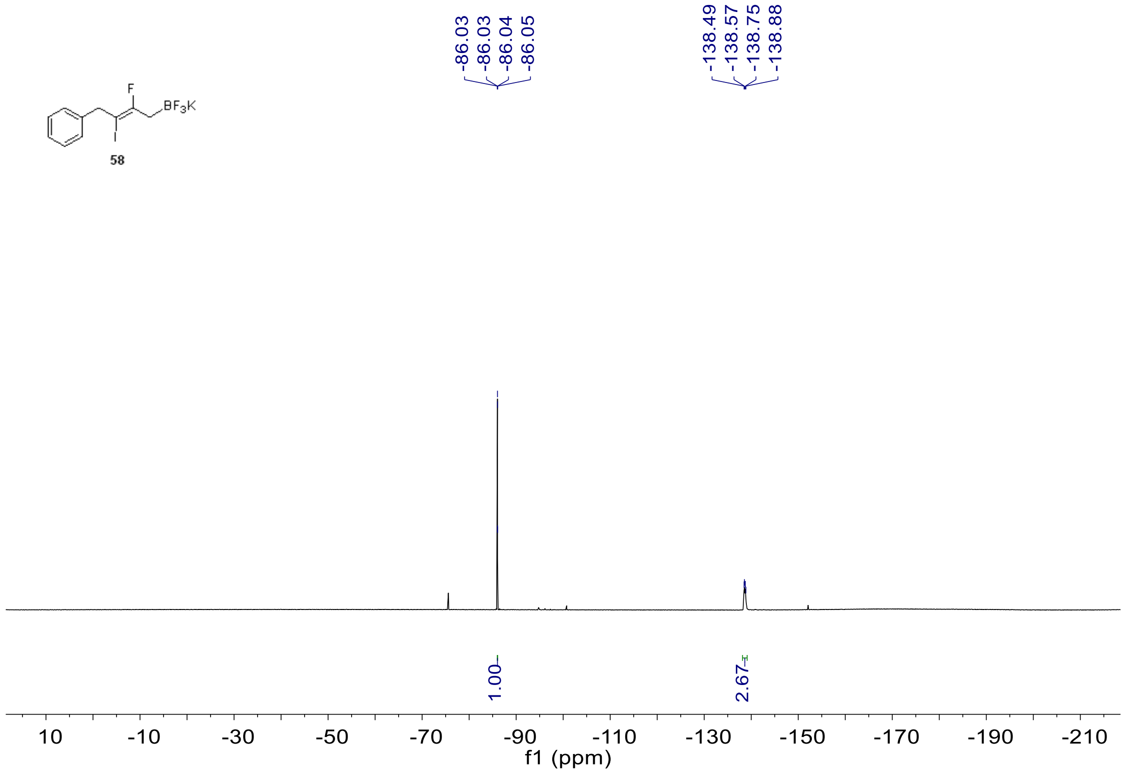
-86.03
-86.03
-86.04
-86.05
-138.49
-138.57
-138.75
-138.88
F
BF3K
I
58
1.00
2.67
10 -10 -30 -50 -70 -90 -110 -130 -150 -170 -190 -210
f1 (ppm)

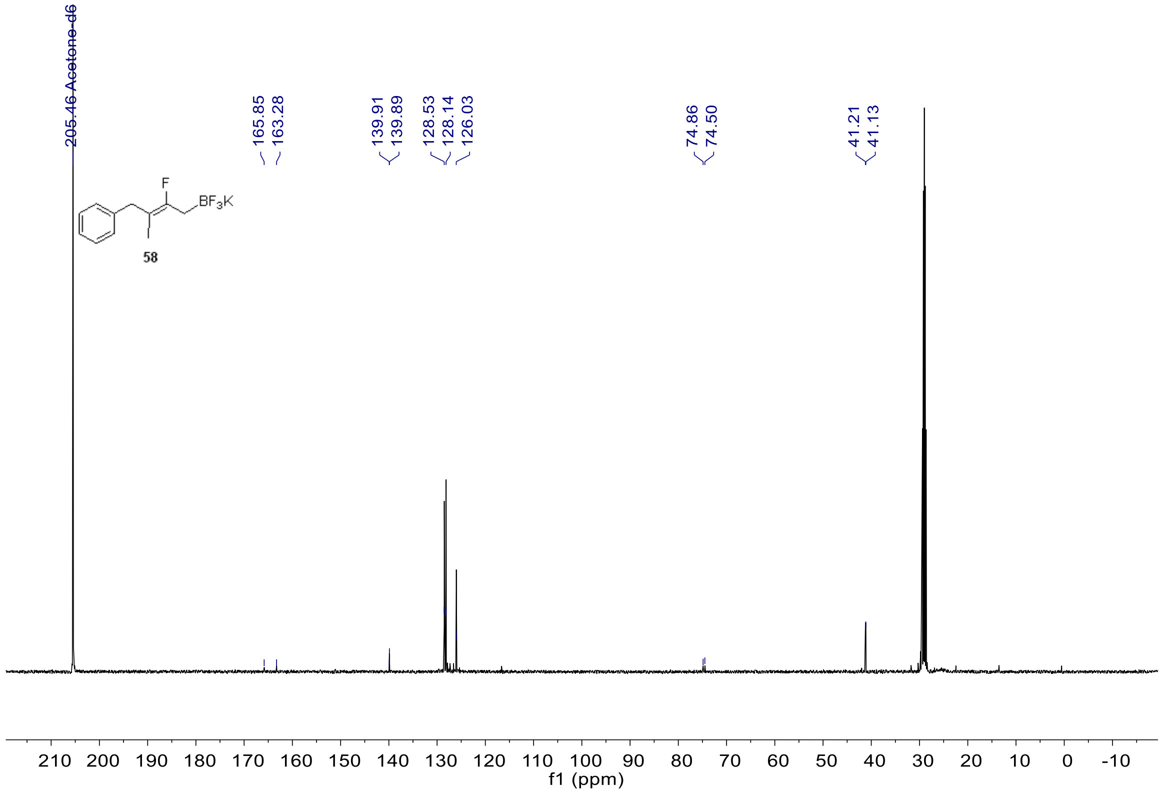
205.46 Acetone-d6
165.85
163.28
139.91
139.89
128.53
128.14
126.03
74.86
74.50
41.21
41.13
F
BF3K
58
210 200 190 180 170 160 150 140 130 120 110 100 90 80 70 60 50 40 30 20 10 0 -10
f1 (ppm)

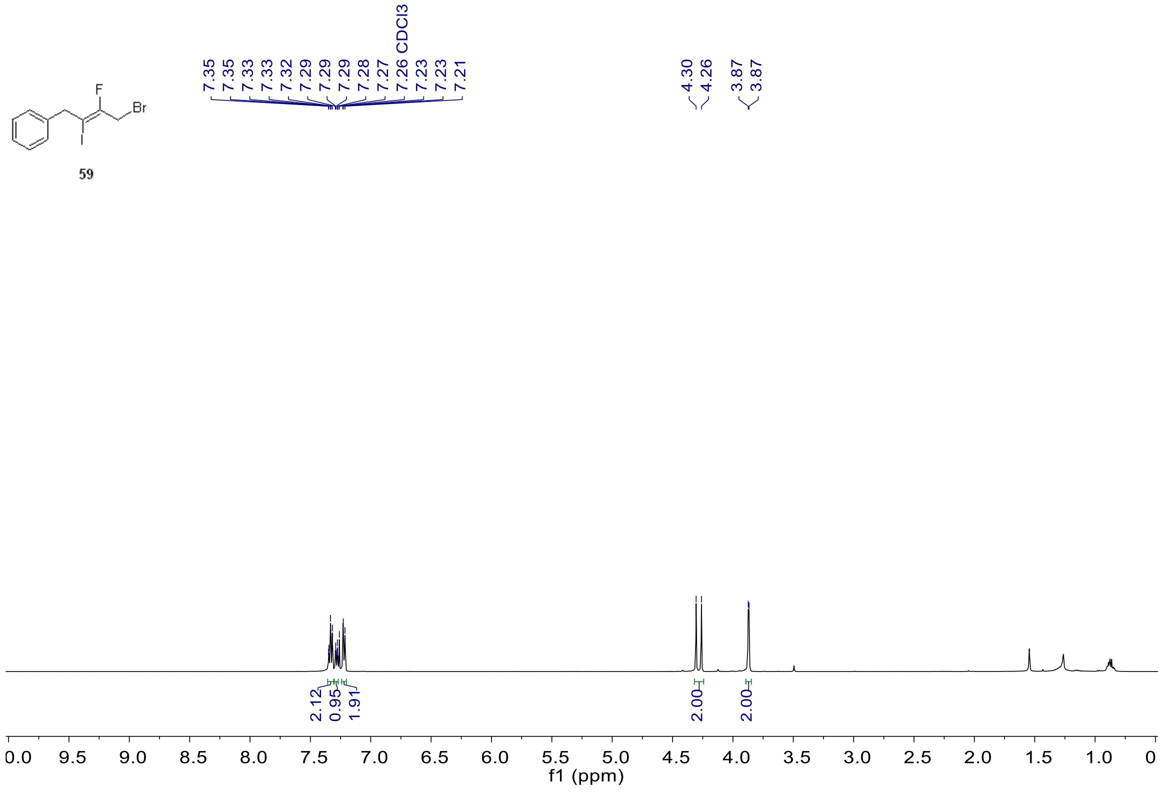
7.35
7.35
7.33
7.33
7.32
7.29
7.29
7.29
7.28
7.27
7.26 CDCl3
7.23
7.23
7.21
4.30
4.26
3.87
3.87
F
Br
59
2.12
0.95
1.91
2.00
2.00
0.0 9.5 9.0 8.5 8.0 7.5 7.0 6.5 6.0 5.5 5.0 4.5 4.0 3.5 3.0 2.5 2.0 1.5 1.0 0
f1 (ppm)

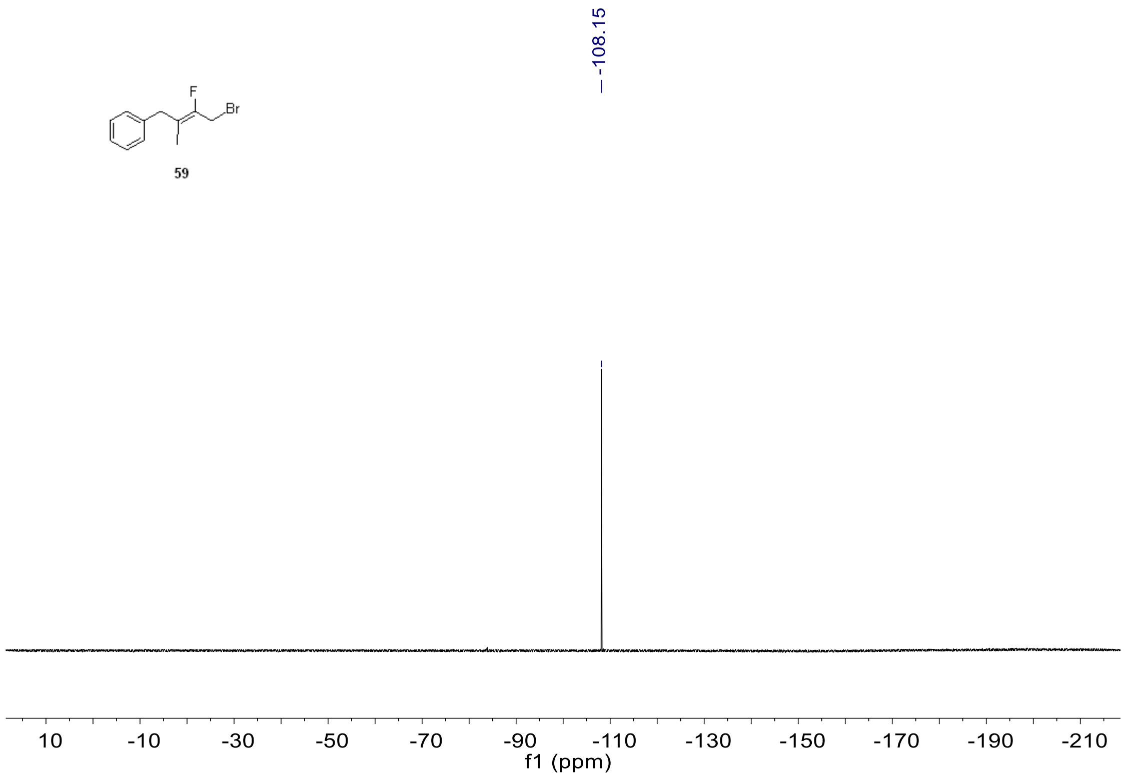
-108.15
F
Br
59
10 -10 -30 -50 -70 -90 -110 -130 -150 -170 -190 -210
f1 (ppm)

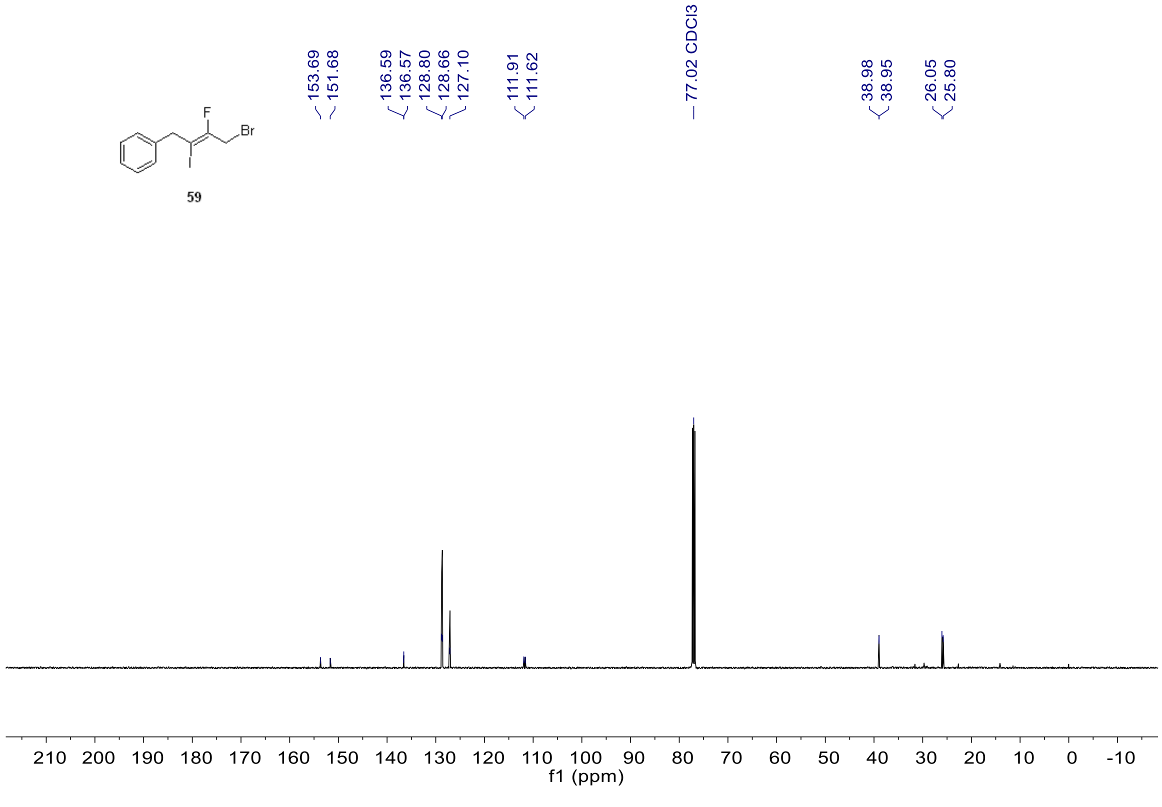
153.69
151.68
136.59
136.57
128.80
128.66
127.10
111.91
111.62
77.02 CDCl3
38.98
38.95
26.05
25.80
F
Br
59
210 200 190 180 170 160 150 140 130 120 110 100 90 80 70 60 50 40 30 20 10 0 -10
f1 (ppm)

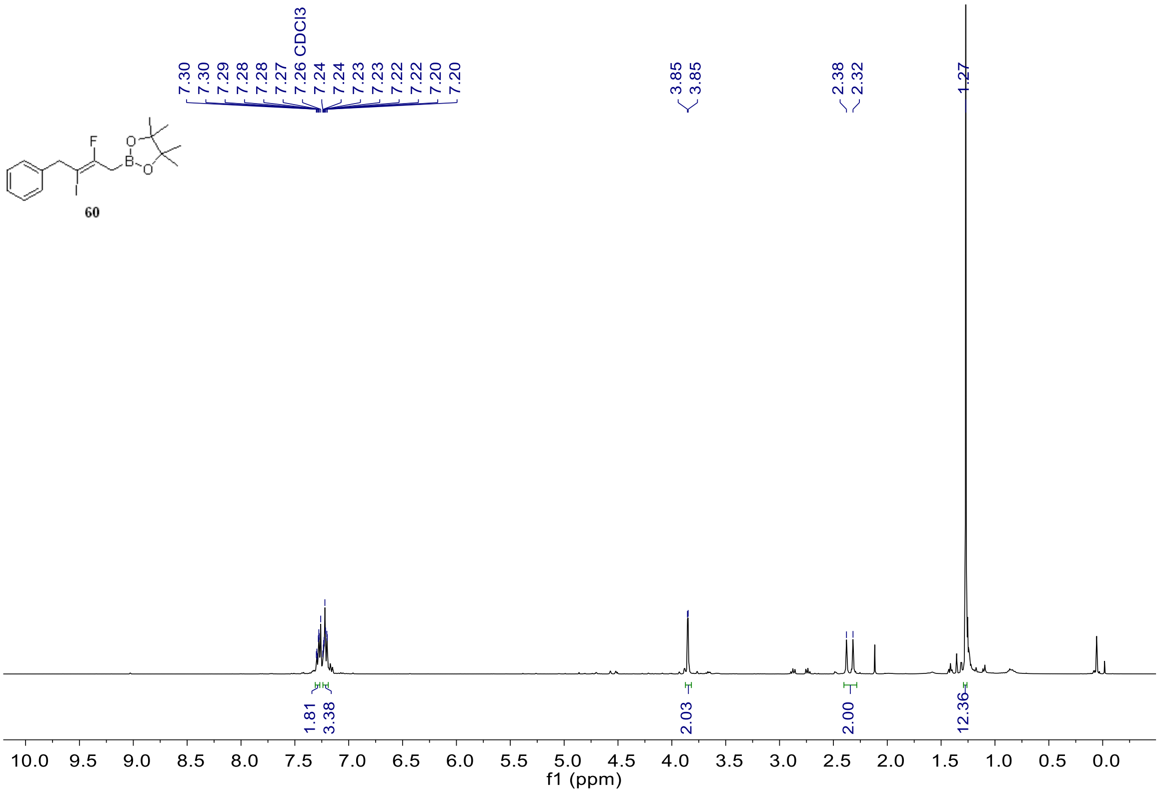
7.30
7.30
7.29
7.28
7.28
7.27
7.26 CDCl3
7.24
7.24
7.23
7.23
7.22
7.22
7.20
7.20
3.85
3.85
2.38
2.32
1.27
F
O
B
O
60
1.81
3.38
2.03
2.00
12.36
10.0 9.5 9.0 8.5 8.0 7.5 7.0 6.5 6.0 5.5 5.0 4.5 4.0 3.5 3.0 2.5 2.0 1.5 1.0 0.5 0.0
f1 (ppm)

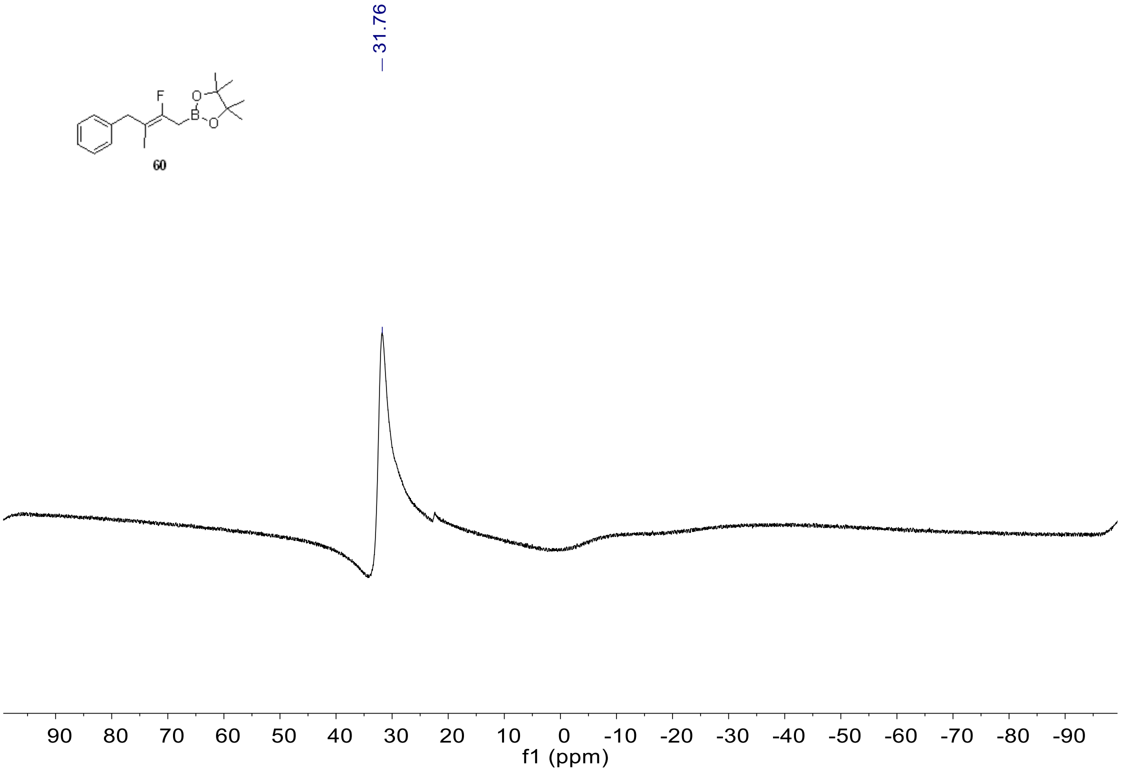
31.76
F
O
B
O
60
90 80 70 60 50 40 30 20 10 0 -10 -20 -30 -40 -50 -60 -70 -80 -90
f1 (ppm)

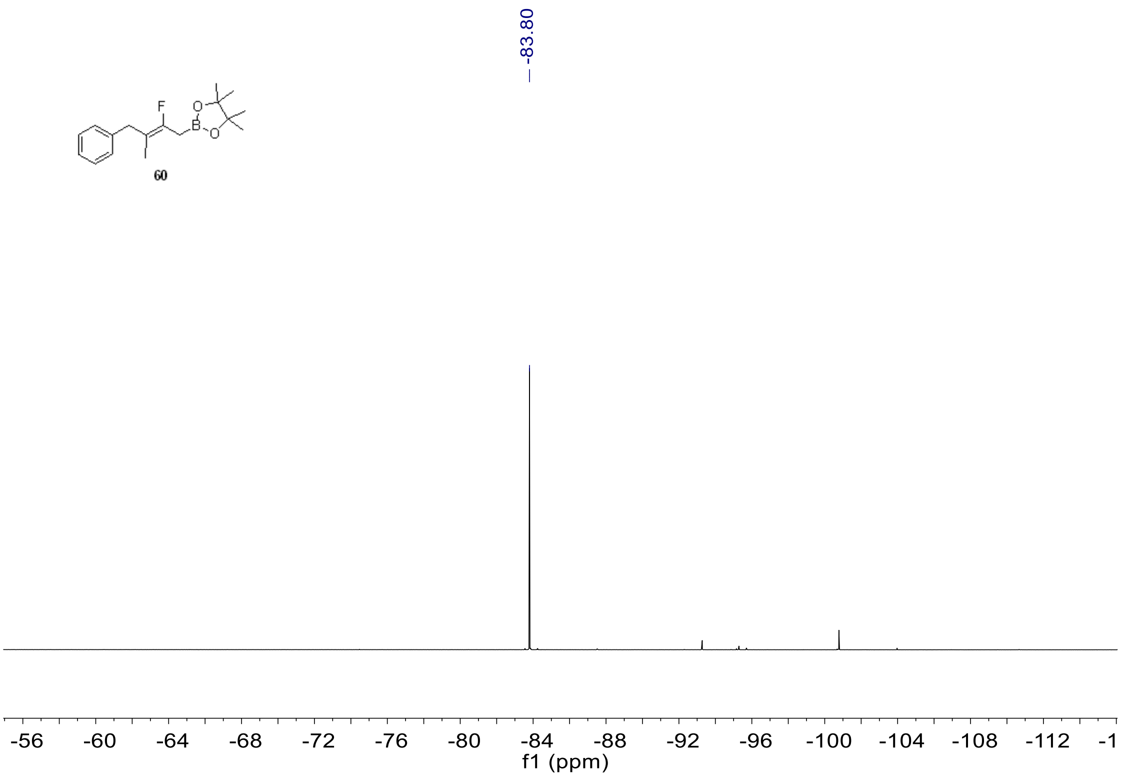
-83.80
F
O
B
O
60
-56 -60 -64 -68 -72 -76 -80 -84 -88 -92 -96 -100 -104 -108 -112 -1
f1 (ppm)

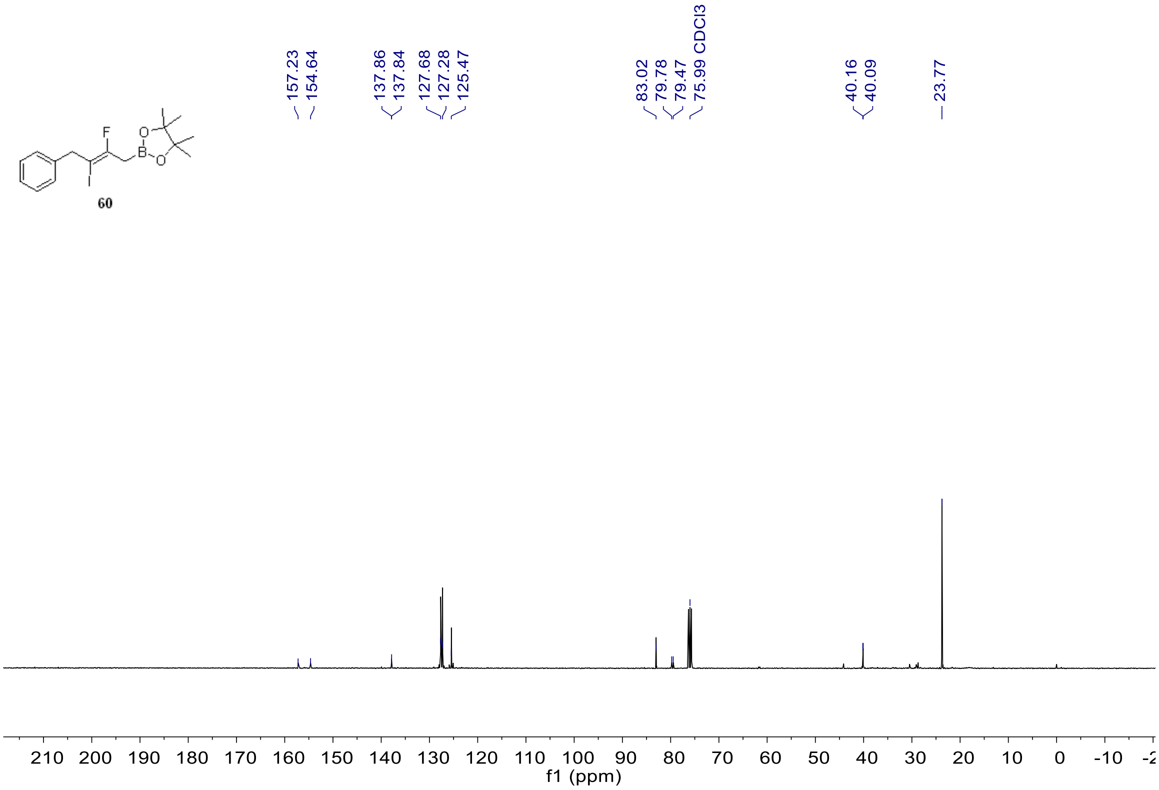
157.23
154.64
137.86
137.84
127.68
127.28
125.47
83.02
79.78
79.47
75.99 CDCl3
40.16
40.09
23.77
F
O
B
O
60
210 200 190 180 170 160 150 140 130 120 110 100 90 80 70 60 50 40 30 20 10 0 -10 -2
f1 (ppm)

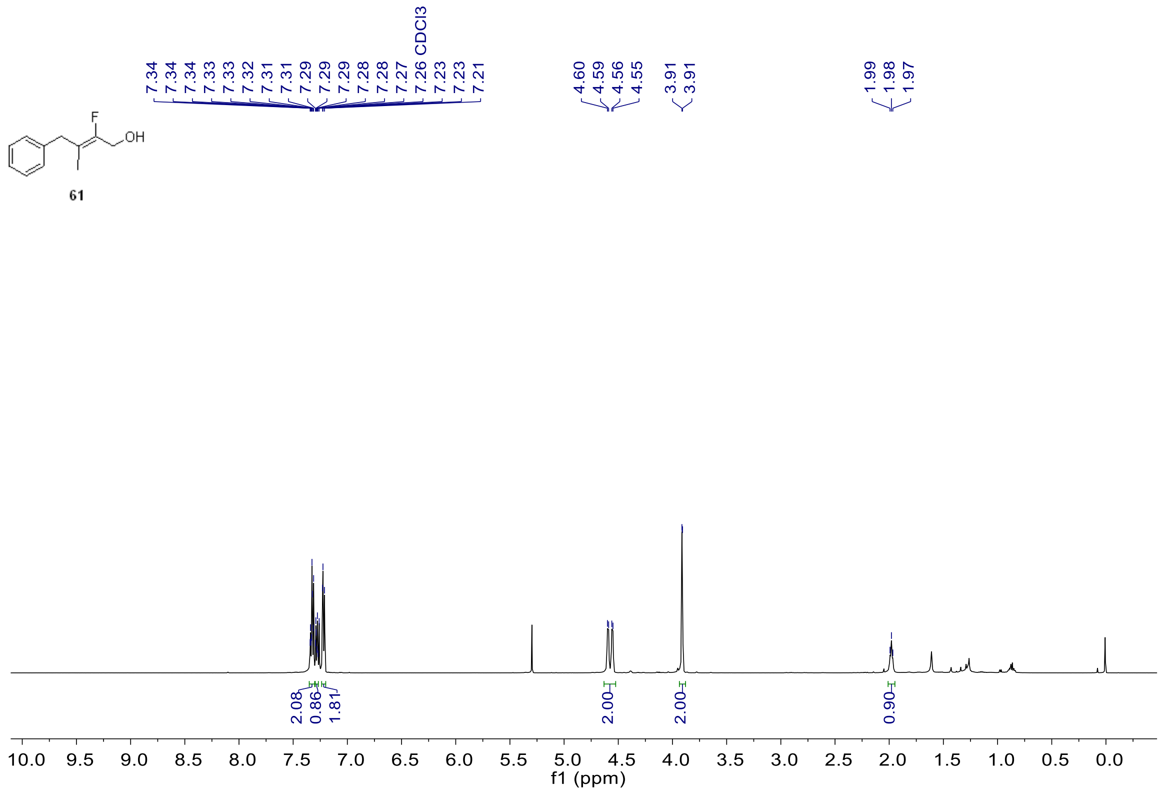
61
7.34 7.34 7.34 7.33 7.33 7.32 7.31 7.31 7.29 7.29 7.29 7.28 7.28 7.27 7.26 CDCl3 7.23 7.23 7.21
4.60 4.59 4.56 4.55
3.91 3.91
1.99 1.98 1.97
2.08 0.86 1.81
2.00
2.00
0.90
10.0 9.5 9.0 8.5 8.0 7.5 7.0 6.5 6.0 5.5 5.0 4.5 4.0 3.5 3.0 2.5 2.0 1.5 1.0 0.5 0.0
f1 (ppm)

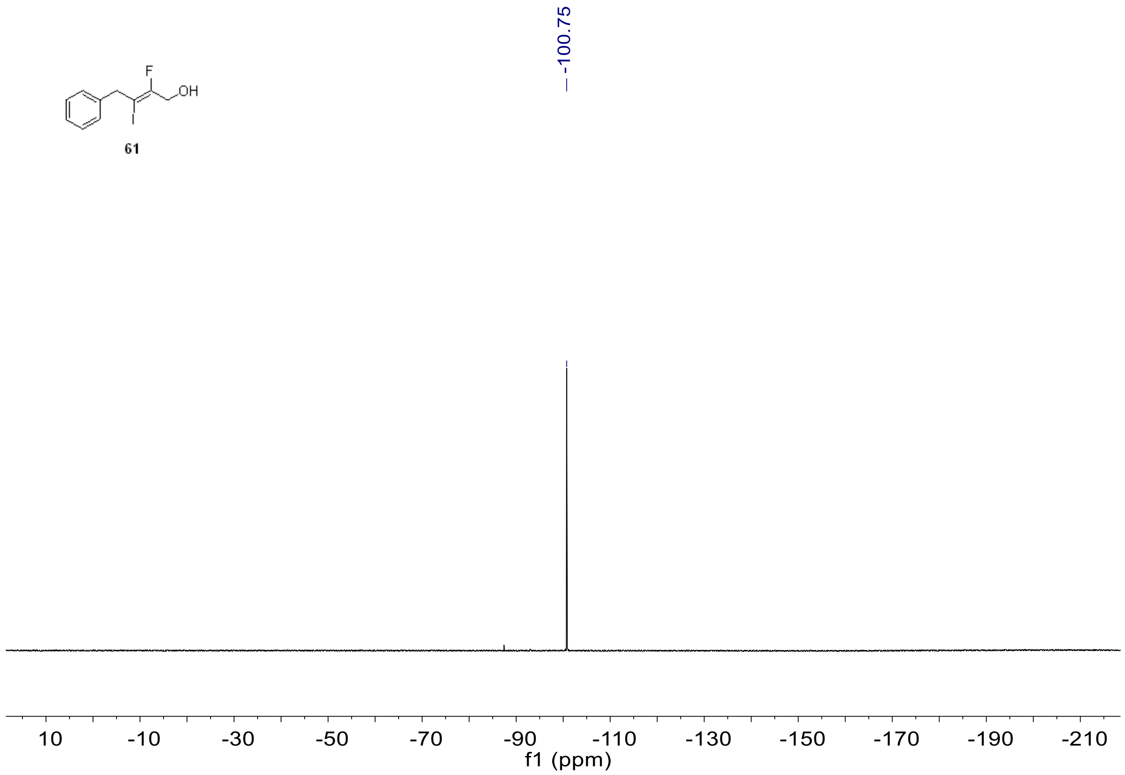
-100.75
61
10 -10 -30 -50 -70 -90 -110 -130 -150 -170 -190 -210
f1 (ppm)

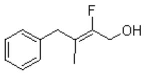
F
OH
I

61

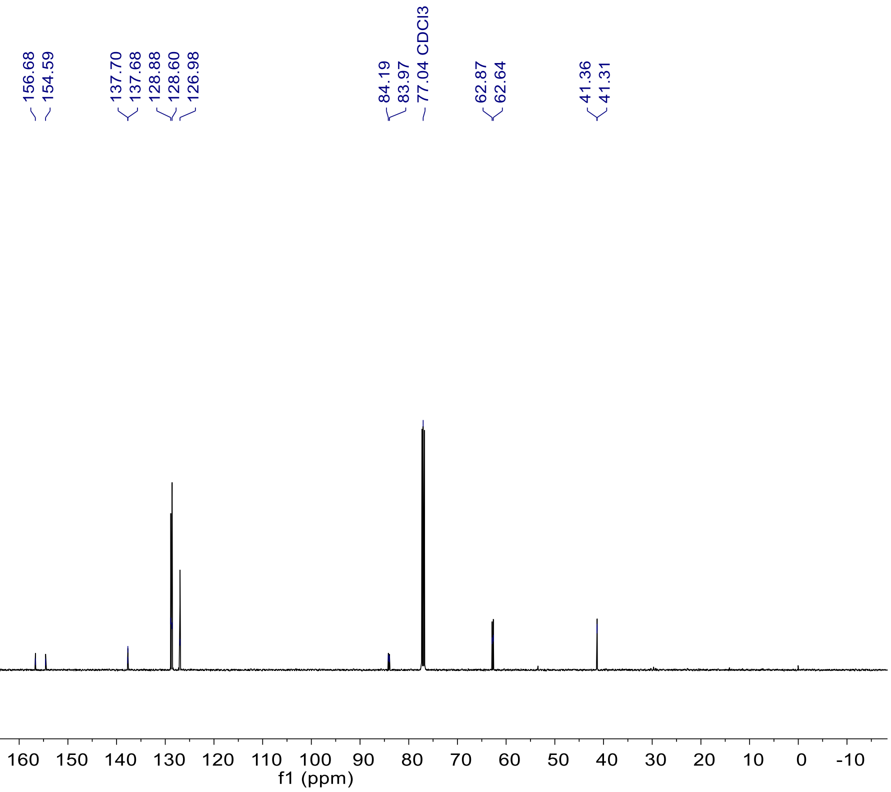
156.68
154.59
137.70
137.68
128.88
128.60
126.98
84.19
83.97
77.04 CDCl3
62.87
62.64
41.36
41.31
210 200 190 180 170 160 150 140 130 120 110 100 90 80 70 60 50 40 30 20 10 0 -10
f1 (ppm)

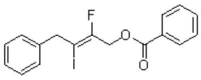
F
O
I
O

62

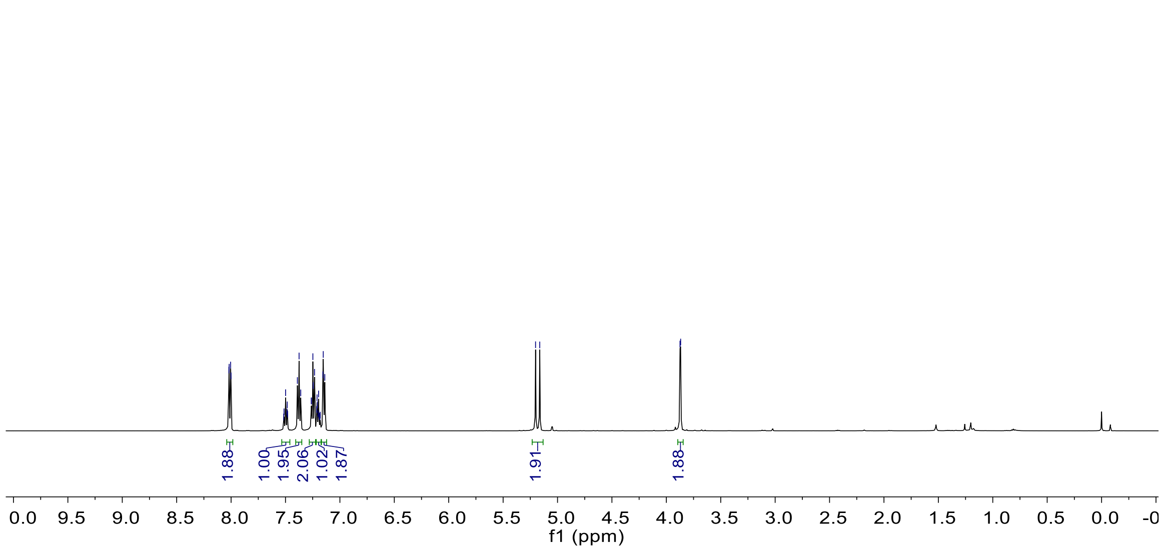
8.02
8.02
8.00
8.00
7.52
7.51
7.50
7.49
7.48
7.48
7.39
7.38
7.36
7.26 CDCl3
7.26
7.25
7.25
7.23
7.21
7.21
7.21
7.20
7.20
7.19
7.18
7.18
7.16
7.15
7.14
5.20
5.16
3.87
3.87
1.88
1.00
1.95
2.06
1.02
1.87
1.91
1.88
0.0 9.5 9.0 8.5 8.0 7.5 7.0 6.5 6.0 5.5 5.0 4.5 4.0 3.5 3.0 2.5 2.0 1.5 1.0 0.5 0.0 -0
f1 (ppm)

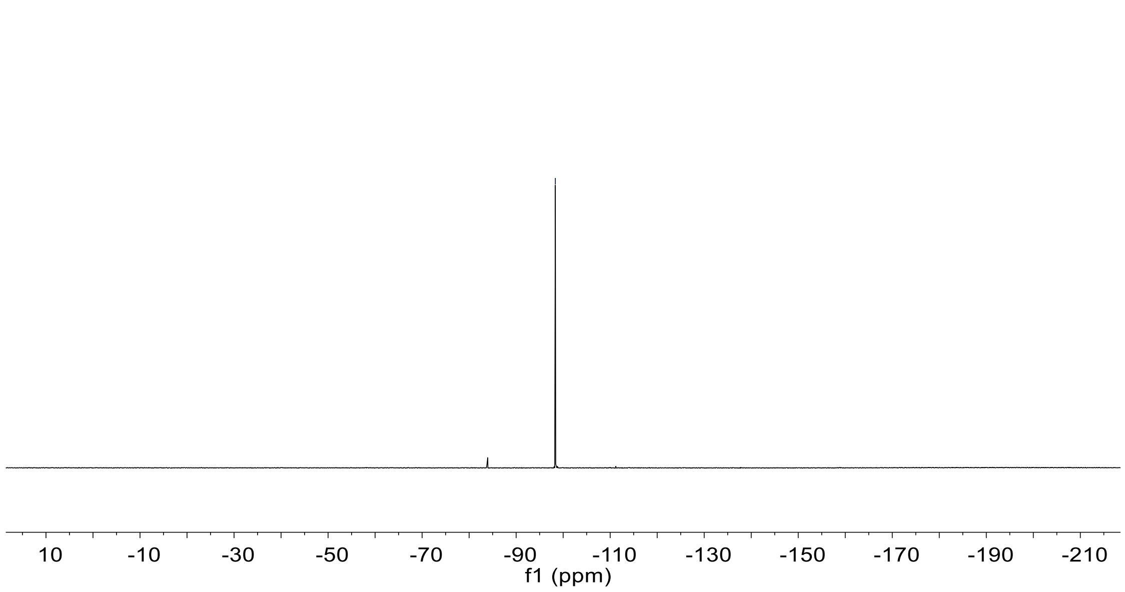
-98.34
10 -10 -30 -50 -70 -90 -110 -130 -150 -170 -190 -210
f1 (ppm)

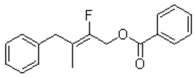
F
O
O

62

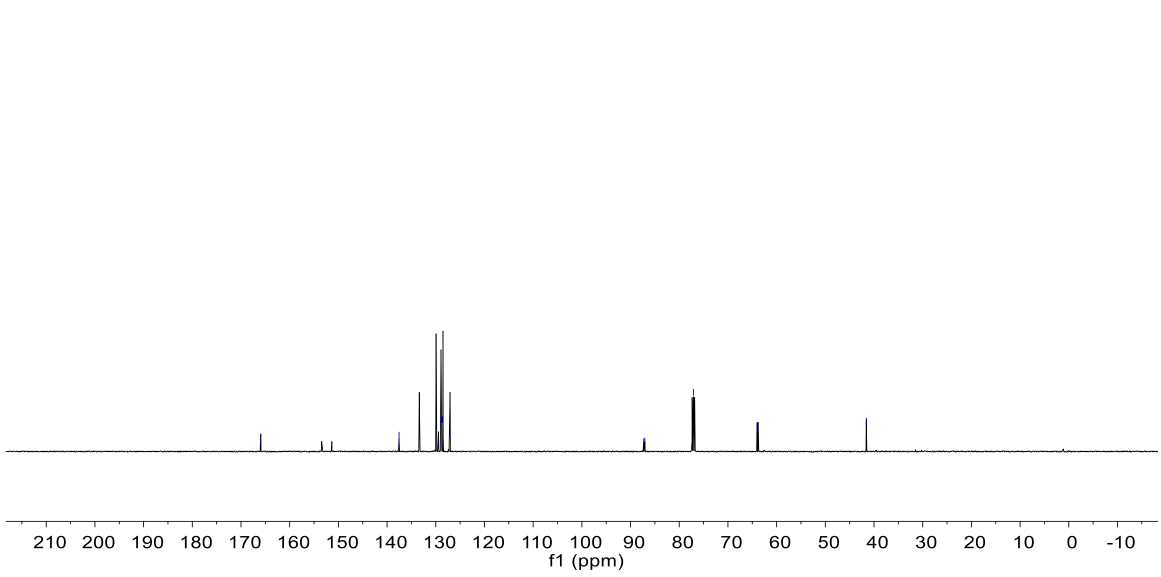
165.95
153.46
151.36
137.55
137.53
133.40
129.93
129.44
128.94
128.67
128.52
127.07
87.29
87.07
77.09 CDCl3
64.03
63.79
41.59
41.54
210 200 190 180 170 160 150 140 130 120 110 100 90 80 70 60 50 40 30 20 10 0 -10
f1 (ppm)

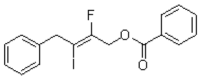
F
O
O

62

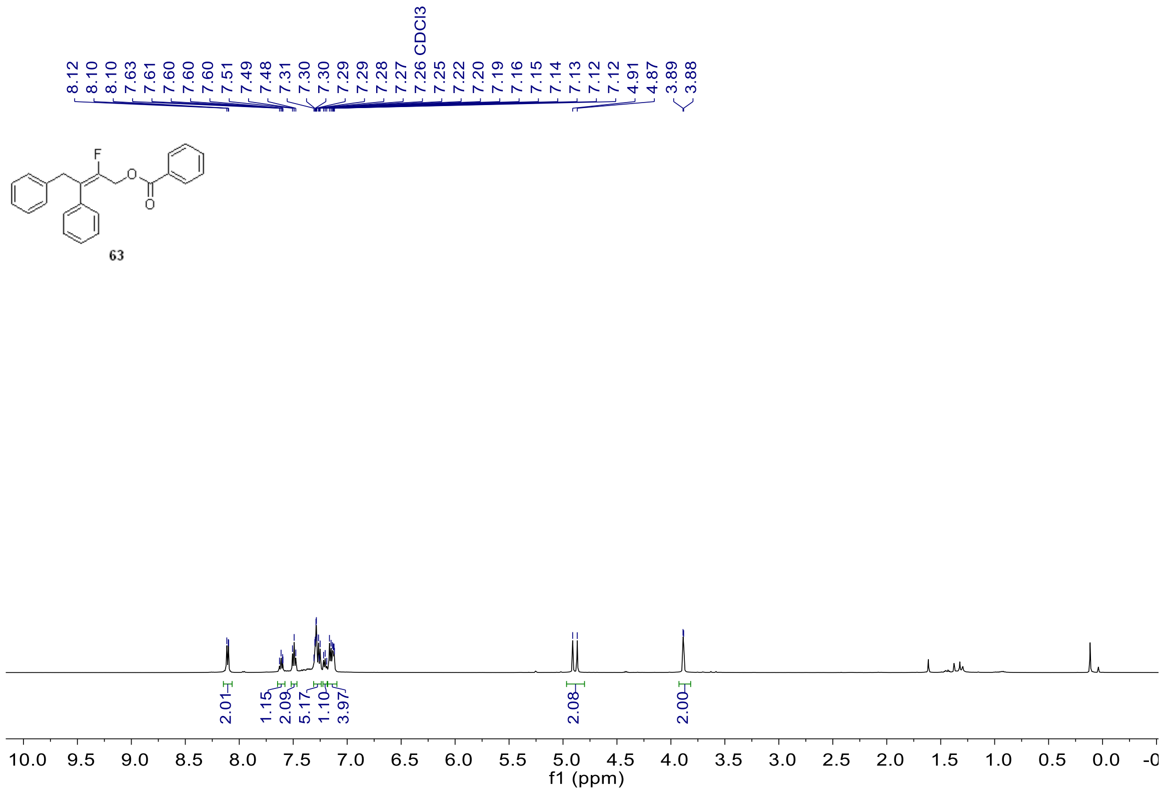
8.12
8.10
8.10
7.63
7.61
7.60
7.60
7.60
7.51
7.49
7.48
7.31
7.30
7.30
7.29
7.28
7.27
7.26 CDCl3
7.25
7.22
7.20
7.19
7.16
7.15
7.14
7.13
7.12
7.12
4.91
4.87
3.89
3.88
F
O
O
2.01
1.15
2.09
5.17
1.10
3.97
2.08
2.00
10.0 9.5 9.0 8.5 8.0 7.5 7.0 6.5 6.0 5.5 5.0 4.5 4.0 3.5 3.0 2.5 2.0 1.5 1.0 0.5 0.0
f1 (ppm)

63

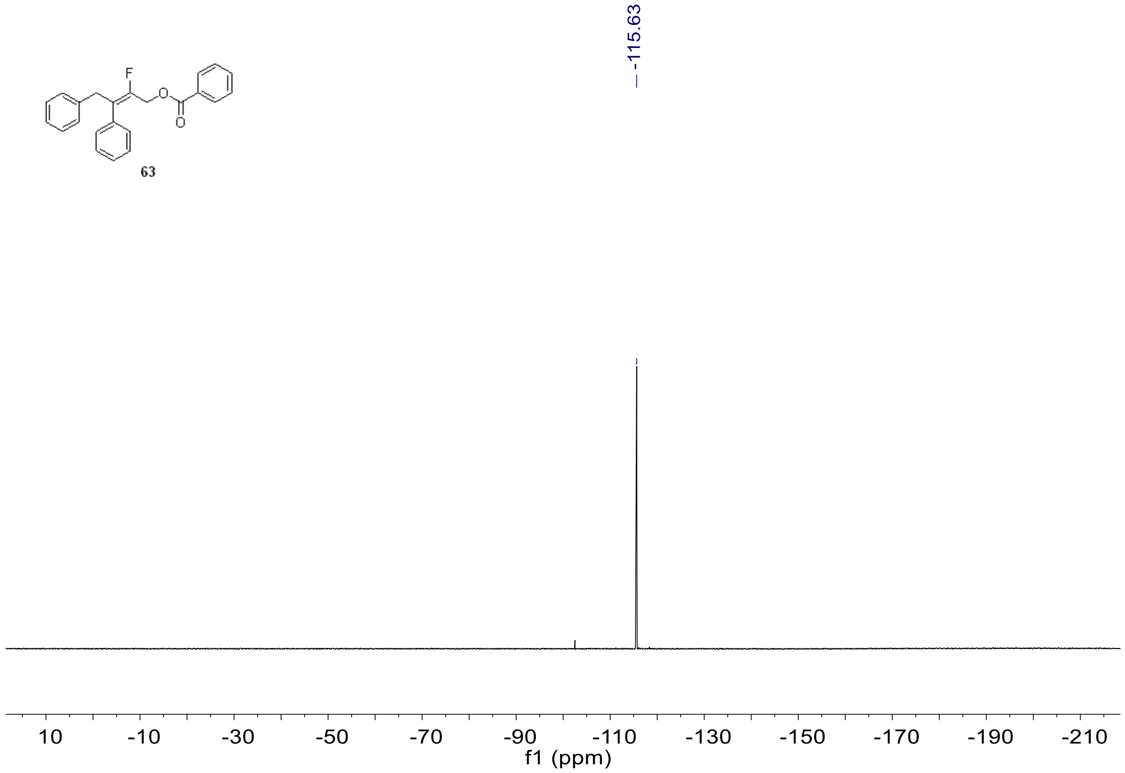
-115.63
F
O
O
10 -10 -30 -50 -70 -90 -110 -130 -150 -170 -190 -210
f1 (ppm)

63

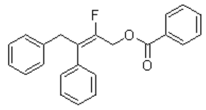

63

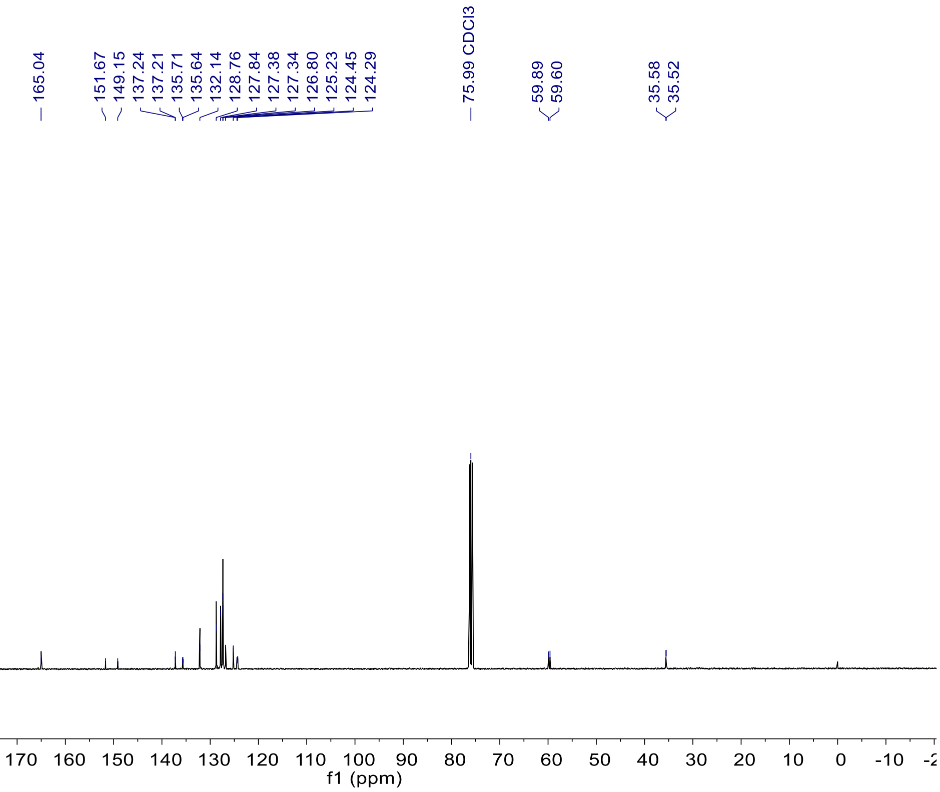
165.04
151.67
149.15
137.24
137.21
135.71
135.64
132.14
128.76
127.84
127.38
127.34
126.80
125.23
124.45
124.29
75.99 CDCl3
59.89
59.60
35.58
35.52
210 200 190 180 170 160 150 140 130 120 110 100 90 80 70 60 50 40 30 20 10 0 -10
f1 (ppm)

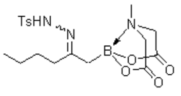

64

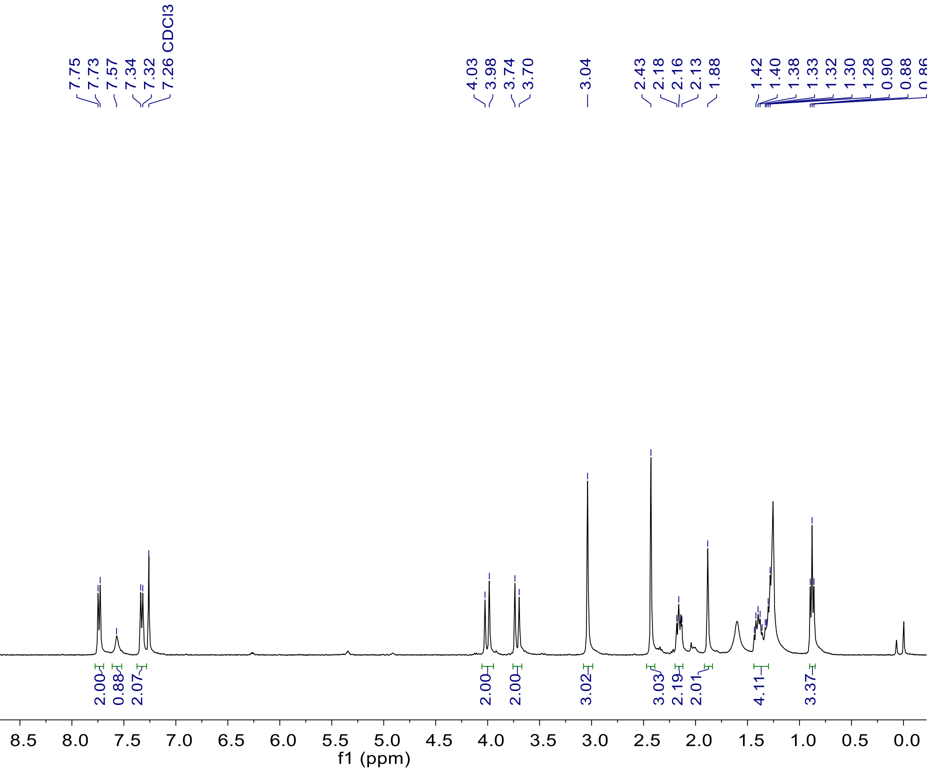
7.75
7.73
7.57
7.34
7.32
7.26 CDCl3
4.03
3.98
3.74
3.70
3.04
2.43
2.18
2.16
2.13
1.88
1.42
1.40
1.38
1.33
1.32
1.30
1.28
0.90
0.88
2.00
0.88
2.07
2.00
2.00
3.02
3.03
2.19
2.01
4.11
3.37
10.0 9.5 9.0 8.5 8.0 7.5 7.0 6.5 6.0 5.5 5.0 4.5 4.0 3.5 3.0 2.5 2.0 1.5 1.0 0.5 0.0
f1 (ppm)

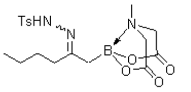

64

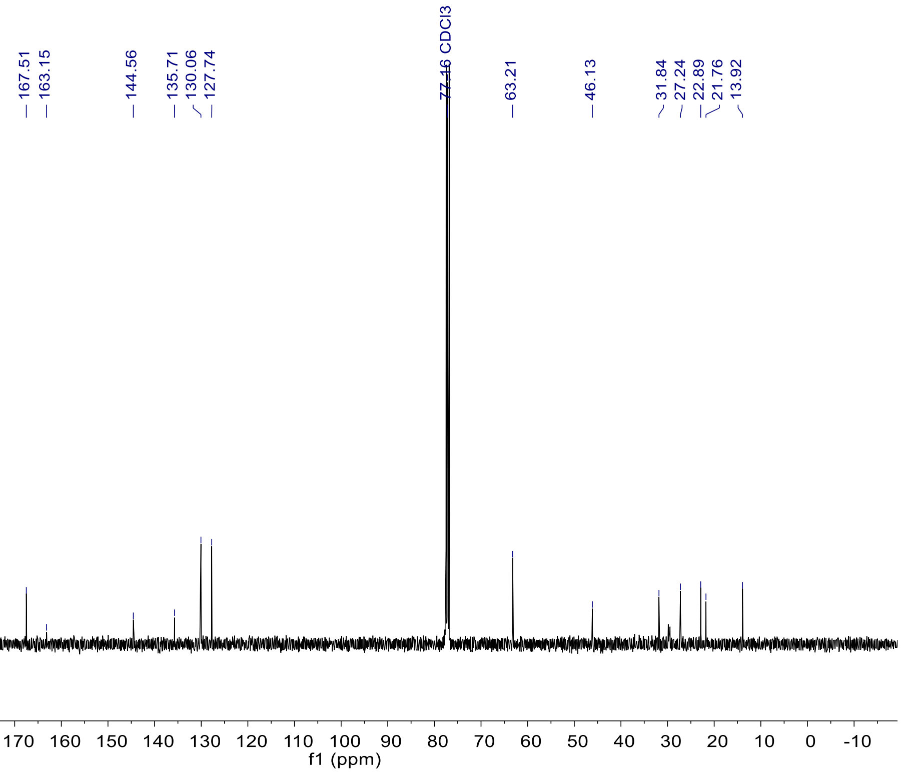

167.51
163.15
144.56
135.71
130.06
127.74
77.16 CDCl3
63.21
46.13
31.84
27.24
22.89
21.76
13.92
210 200 190 180 170 160 150 140 130 120 110 100 90 80 70 60 50 40 30 20 10 0 -10
f1 (ppm)

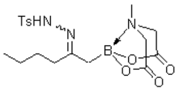

64

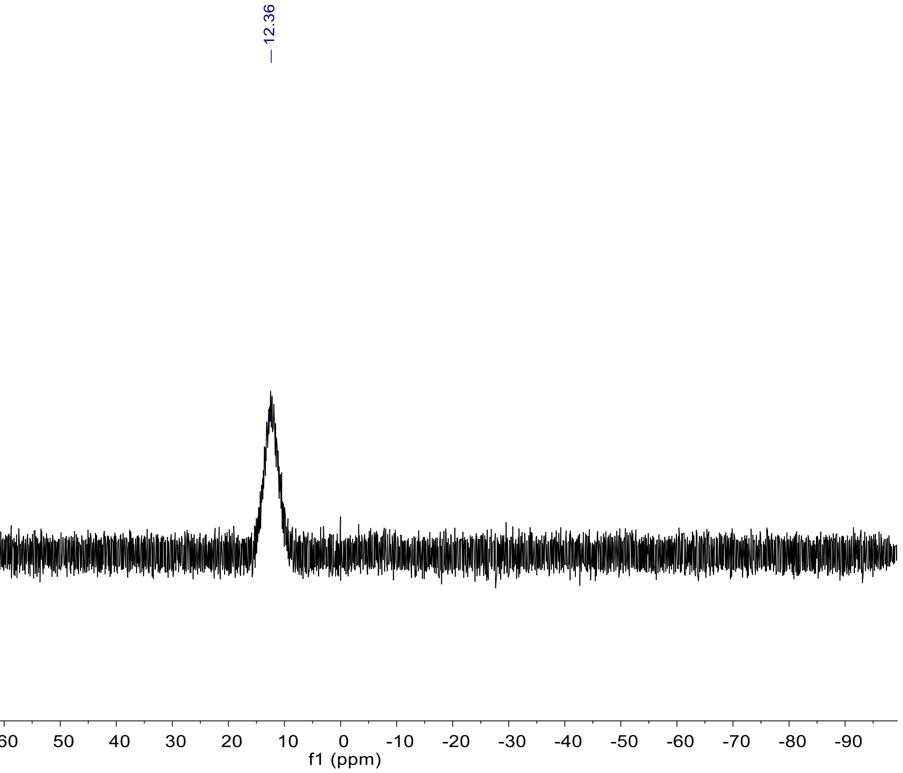

12.36
90 80 70 60 50 40 30 20 10 0 -10 -20 -30 -40 -50 -60 -70 -80 -90
f1 (ppm)

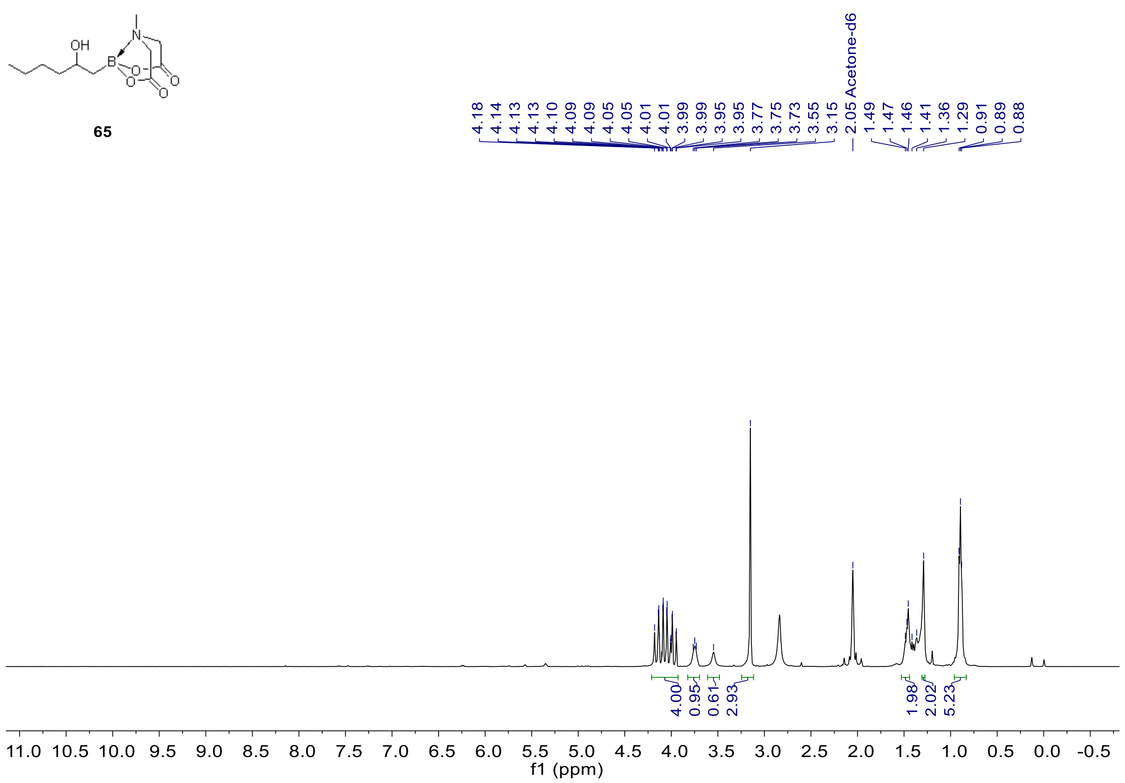
65
4.18 4.14 4.13 4.13 4.10 4.09 4.09 4.05 4.05 4.01 4.01 3.99 3.99 3.95 3.95 3.77 3.75 3.73 3.55 3.15
2.05 Acetone-d6
1.49 1.47 1.46 1.41 1.36 1.29 0.91 0.89 0.88
4.00 0.95 0.61 2.93 1.98 2.02 5.23
11.0 10.5 10.0 9.5 9.0 8.5 8.0 7.5 7.0 6.5 6.0 5.5 5.0 4.5 4.0 3.5 3.0 2.5 2.0 1.5 1.0 0.5 0.0 -0.5
f1 (ppm)

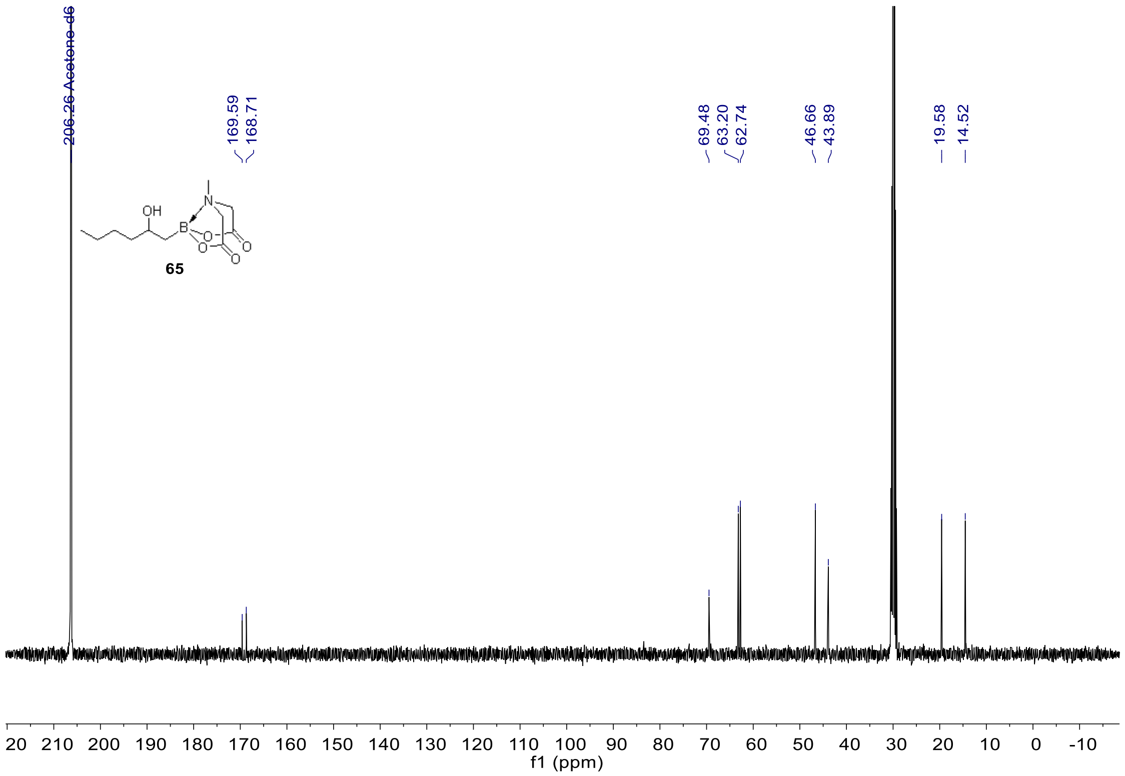
206.26 Acetone-d6
169.59 168.71
69.48 63.20 62.74
46.66 43.89
19.58 14.52
65
20 210 200 190 180 170 160 150 140 130 120 110 100 90 80 70 60 50 40 30 20 10 0 -10
f1 (ppm)

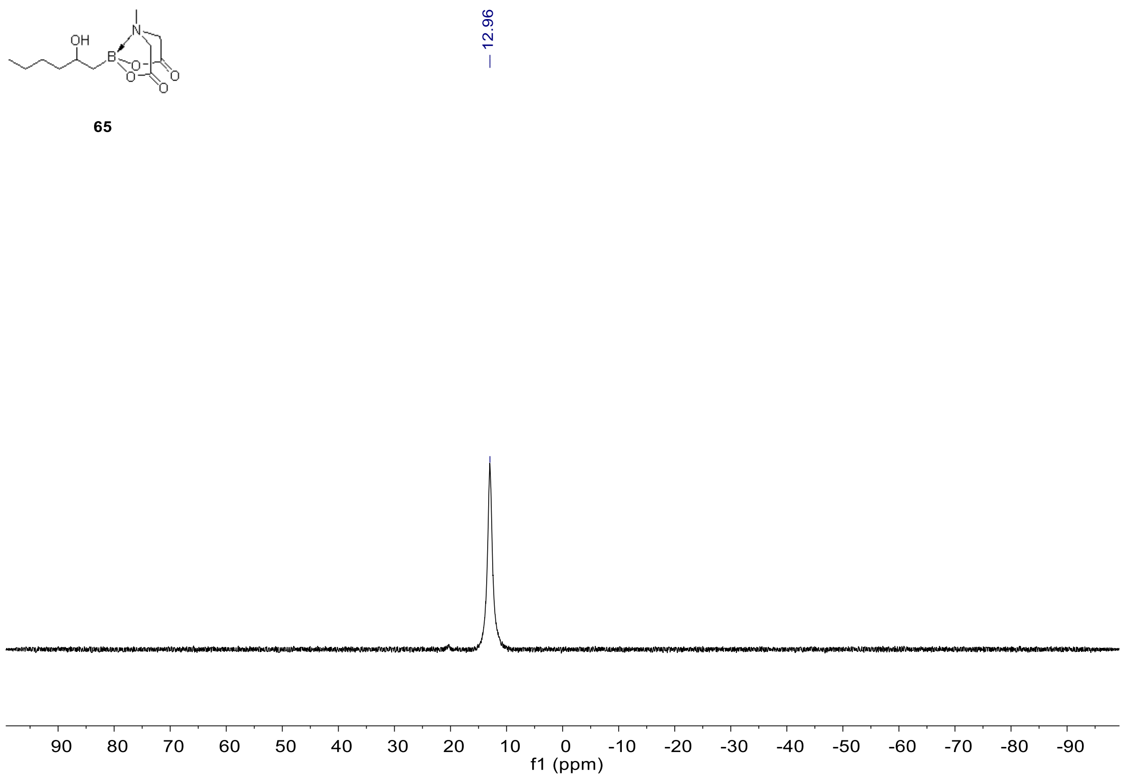
OH
B
N
O
O
O
O
12.96
90 80 70 60 50 40 30 20 10 0 -10 -20 -30 -40 -50 -60 -70 -80 -90
f1 (ppm)

65

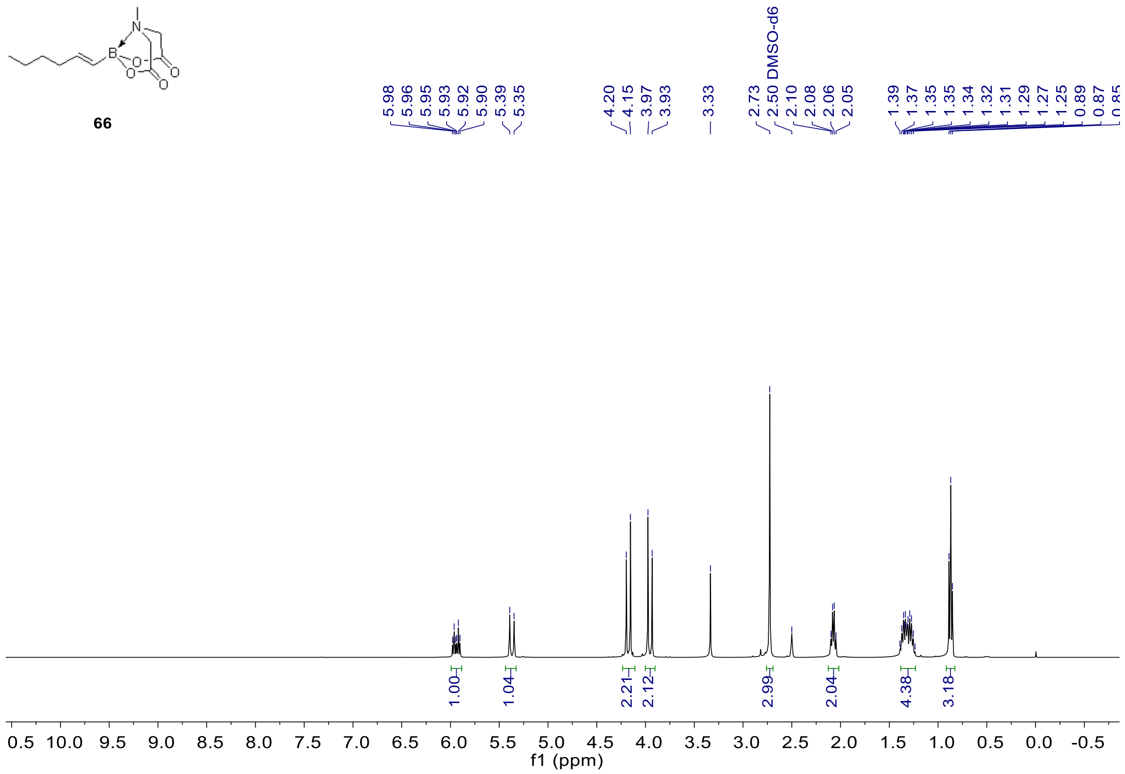
B
N
O
O
O
O
DMSO-d6
5.98 5.96 5.95 5.93 5.92 5.90 5.39 5.35
4.20 4.15 3.97 3.93
3.33
2.73 2.50 2.10 2.08 2.06 2.05
1.39 1.37 1.35 1.35 1.34 1.32 1.31 1.29 1.27 1.25 0.89 0.87
1.00 1.04 2.21 2.12 2.99 2.04 4.38 3.18
0.5 10.0 9.5 9.0 8.5 8.0 7.5 7.0 6.5 6.0 5.5 5.0 4.5 4.0 3.5 3.0 2.5 2.0 1.5 1.0 0.5 0.0 -0.5
f1 (ppm)

66

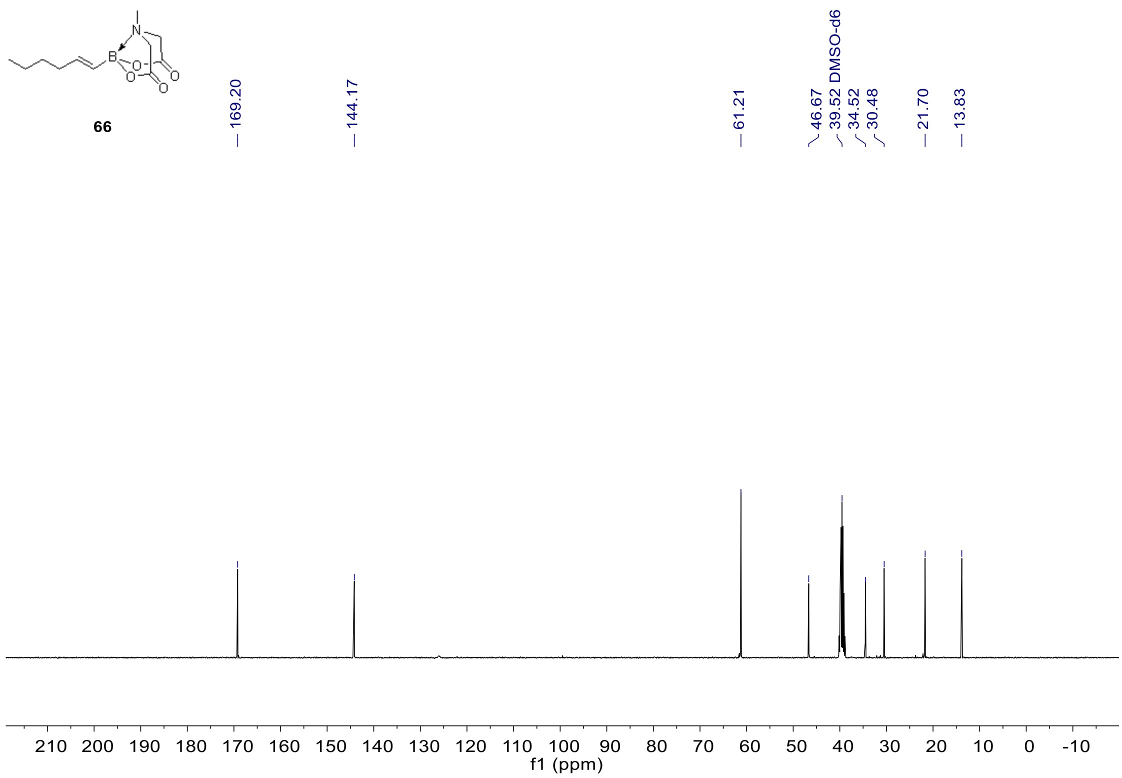
66
169.20
144.17
61.21
46.67
39.52 DMSO-d6
34.52
30.48
21.70
13.83
210 200 190 180 170 160 150 140 130 120 110 100 90 80 70 60 50 40 30 20 10 0 -10
f1 (ppm)

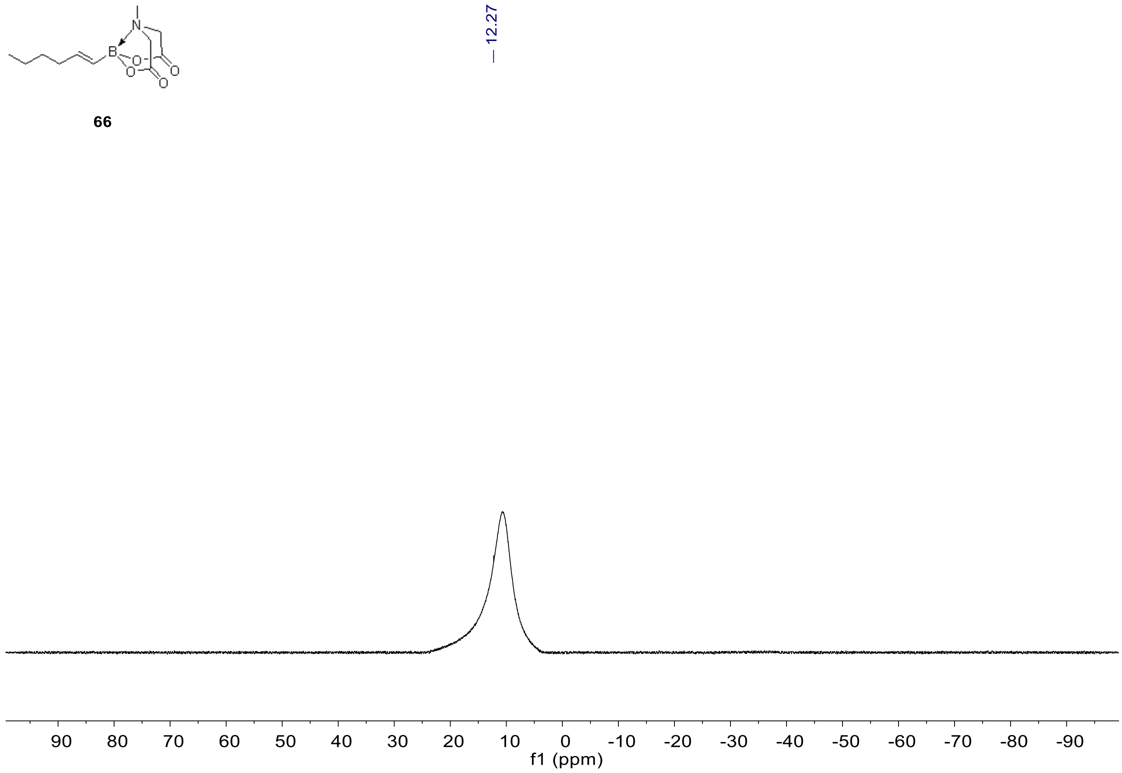
66
12.27
90 80 70 60 50 40 30 20 10 0 -10 -20 -30 -40 -50 -60 -70 -80 -90
f1 (ppm)

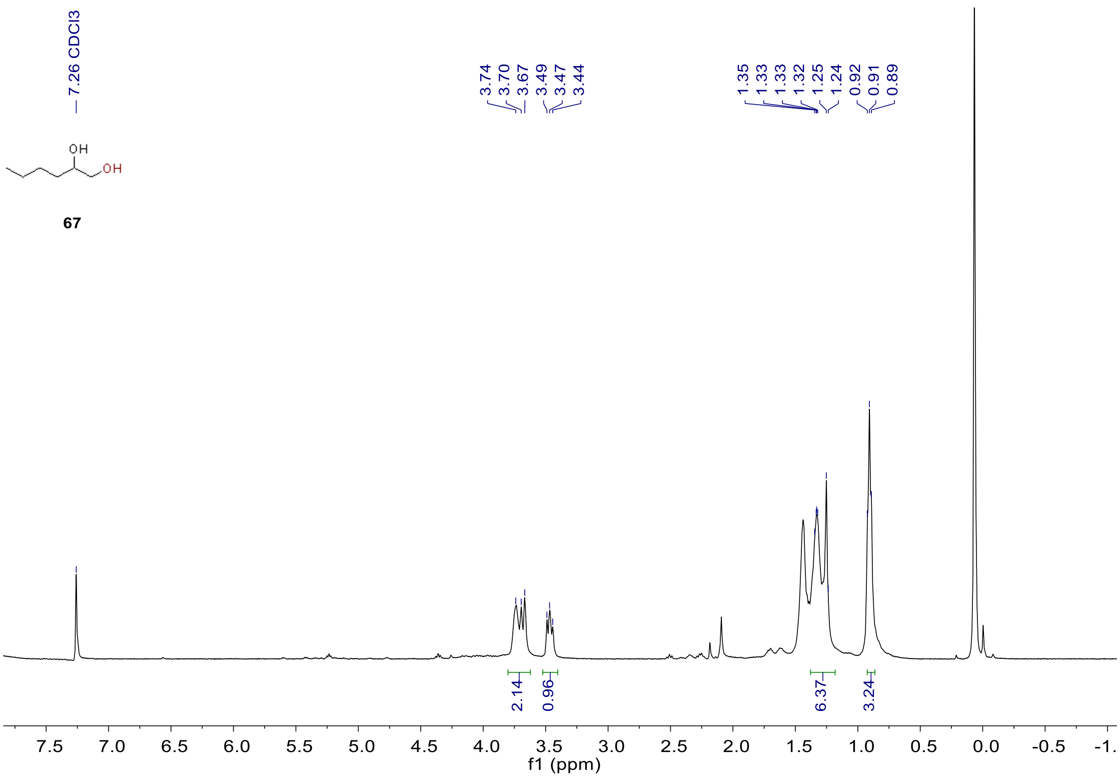

OH
OH
67
7.26 CDCl3
3.74
3.70
3.67
3.49
3.47
3.44
1.35
1.33
1.33
1.32
1.25
1.24
0.92
0.91
0.89
2.14
0.96
6.37
3.24
7.5 7.0 6.5 6.0 5.5 5.0 4.5 4.0 3.5 3.0 2.5 2.0 1.5 1.0 0.5 0.0 -0.5 -1.
f1 (ppm)
